# A Simple and Broadly Applicable C−N Bond Forming Dearomatization Protocol Enabled by Bifunctional Amino Reagents

**DOI:** 10.1002/anie.201708176

**Published:** 2017-10-11

**Authors:** Xiaofeng Ma, Joshua J. Farndon, Tom A. Young, Natalie Fey, John F. Bower

**Affiliations:** ^1^ School of Chemistry University of Bristol Bristol BS8 1TS UK

**Keywords:** C−N bond, dearomatization, spirocyclic pyrrolidine

## Abstract

A C−N bond forming dearomatization protocol with broad scope is outlined. Specifically, bifunctional amino reagents are used for sequential nucleophilic and electrophilic C−N bond formations, with the latter effecting the key dearomatization step. Using this approach, γ‐arylated alcohols are converted to a wide range of differentially protected spirocyclic pyrrolidines in just two or three steps.

Electrophile‐triggered dearomatization processes enable the direct conversion of readily prepared planar molecules to synthetically valuable three‐dimensional scaffolds,^1^ often with the concomitant provision of functionality primed for diversification. C−C bond forming processes of this type now offer exceptional utility,^1^, ^2^ but related C−N bond forming dearomatizations have not reached the same level of sophistication (Scheme 1 A). Within this context, predominant methods rely on nitrenium ions and are limited to lactam products equipped with specific N‐protecting groups.^3^ Oxime‐based processes, which provide dihydropyrroles, are also of note, although competing Beckmann rearrangement is often problematic.^4^ As far as we are aware, the direct synthesis of pyrrolidines using electrophilic nitrogen triggered dearomatizations has not been achieved. Ciufolini and co‐workers have pioneered a conceptually distinct approach involving oxidation of an arene to its corresponding cation in advance of trapping by a nitrogen nucleophile.^5^ Although often effective, the process requires specific N‐protecting groups (carbamates are not tolerated), is most efficient under strongly acidic conditions (e.g. TFA as solvent) and can suffer from competing oxidation processes involving the arene.5e Given the limitations associated with the state‐of‐the‐art, we reasoned that the development of a C−N bond forming dearomatization platform with broad scope would represent a significant and useful advance. Specifically, we sought to circumvent the requirement of a strong external oxidant, and, at the same time, provide a pyrrolidine synthesis that can accommodate a variety of N‐protecting groups (especially synthetically flexible carbamates) and a range of nucleophilic arenes (e.g. phenols, naphthols, indoles). As outlined below, we have been able to achieve this by developing a suite of bifunctional amino reagents **2** that allow the two or three‐step conversion of γ‐aryl alcohols (e.g. **1**) to protected pyrrolidines (e.g. **4**) (Scheme 1 B). This unique approach is efficient, mild (no external oxidant) and operationally simple, offering exceptionally wide scope with respect to both the arene and N‐protecting group. The spirocyclic pyrrolidine products obtained by this method are core motifs in many natural products and are also recognized as privileged scaffolds in drug discovery.^6^ Accordingly, we anticipate that the strategy described herein will find broad utility in synthetic settings.

**Scheme 1:**
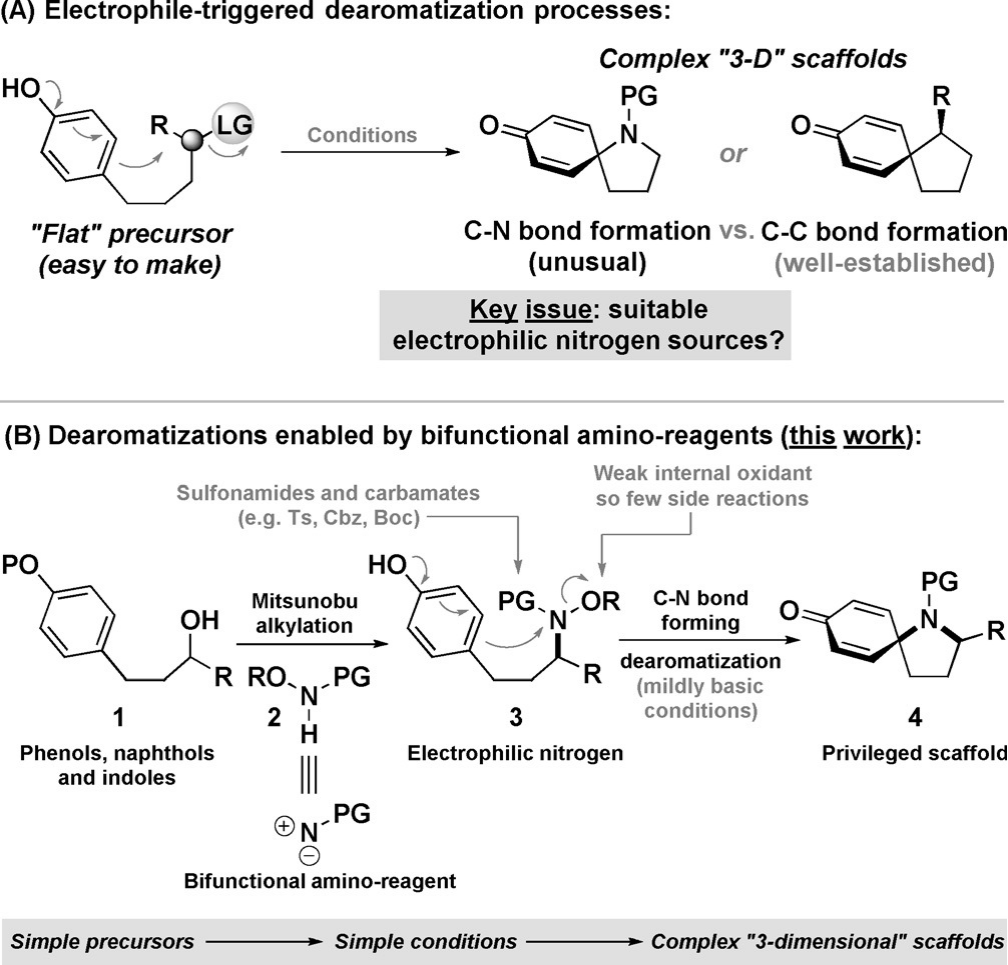


Our studies in this area stemmed from ongoing efforts to exploit electrophilic nitrogen sources for N‐heterocycle synthesis.^7^ Specifically, we envisaged that initial exploitation of the nucleophilic character of **2** for Mitsunobu alkylation with alcohols **1** would provide precursors **3**. At this stage, the electrophilic character of the nitrogen center would be harnessed to trigger the dearomatization step (to **4**). The overall approach offers several distinct advantages: 1) pre‐installation of the O‐based leaving group on nitrogen facilitates the Mitsunobu step and avoids protecting group manipulations,^8^ 2) readily prepared enantiopure secondary alcohols can potentially be used as a starting point,^7^ 3) the use of an N−O bond as a mild internal oxidant should minimize competing oxidation processes involving the arene, 4) the leaving group derived byproduct (HOR) should be easy to remove during work‐up, and 5) a wide range of N‐protecting groups should be tolerated because the dearomatization step relies on the electrophilicity of the nitrogen center, rather than on its nucleophilicity.

To evaluate the feasibility of the approach outlined in Scheme 1 B, we targeted initially a range of differentially protected reagents **2 a**–**f′**, equipped with either OTs or O^F^Bz leaving groups (see below) (see Table 1, box). In the event, these reagents were easily prepared on multi‐gram scale and showed good levels of stability (see the Supporting Information). Mitsunobu alkylation of **2 a**–**f′**, with 3‐(1*H*‐indol‐3‐yl)propan‐1‐ol as the pro‐electrophile, provided targets **3 a**–**f′** in an efficient manner (57–86 % yield, see the Supporting Information). Despite the lack of direct precedent, C−N bond forming dearomatization of O^F^Bz system **3 a** was achieved using a very simple protocol: exposure to catalytic quantities of K_2_HPO_4_ (15 mol %) at 140 °C in *n*‐BuCN resulted in spirocyclization to provide **4 a** in 44 % yield (Conditions A). Under the same conditions, spirocyclization of Mbs protected system **3 b** generated **4 b** in 65 % yield. With the aim of accessing *N*‐tosyl protected pyrrolidines more efficiently, we investigated the cyclization of OTs system **3 a′**. Optimization studies revealed that the desired process could be achieved under much milder conditions (K_2_CO_3_, TFE, 80 °C) than for **3 a**, and this enabled access to **4 a** in 75 % yield (Conditions B). The relative facility of Conditions A vs. B is reflective of the leaving group ability of pentafluorobenzoate vs. tosylate (p*K*_a_ values in H_2_O at 25 °C: ^F^BzOH 1.75, TsOH 0.7).^9^ Although OTs systems offer higher reactivity, we have found that an O^F^Bz leaving group can be beneficial in certain cases as the substrates are more stable, and this increased stability can translate into enhanced dearomatization yields. Indeed, O^F^Bz precursor **3 c** was more efficient than OTs precursor **3 c′** for accessing *N*‐Cbz system **4 c** (82 % vs. 70 % yield). Conversely, an OTs leaving group was most effective for the formation of methyl and *tert*‐butyl carbamate products **4 d** and **4 e**. Satisfactory results were achieved for Alloc‐protected system **4 f** using Conditions B. The results in Table 1 are significant as they show that the method tolerates a wide range of synthetically useful *N*‐protecting groups and that it is also mild enough for efficient dearomatization of the oxidatively sensitive indole core; C−N bond forming dearomatizations of this unit to provide spirocyclic pyrrolidine products have not been reported previously.^4^, ^10^, ^11^, ^12^

**Table 1:** Evaluation of electrophilic aminating agents for the C−N bond forming dearomatization process (^F^Bz=pentafluorobenzoyl, Mbs= *p*‐methoxybenzenesulfonyl).

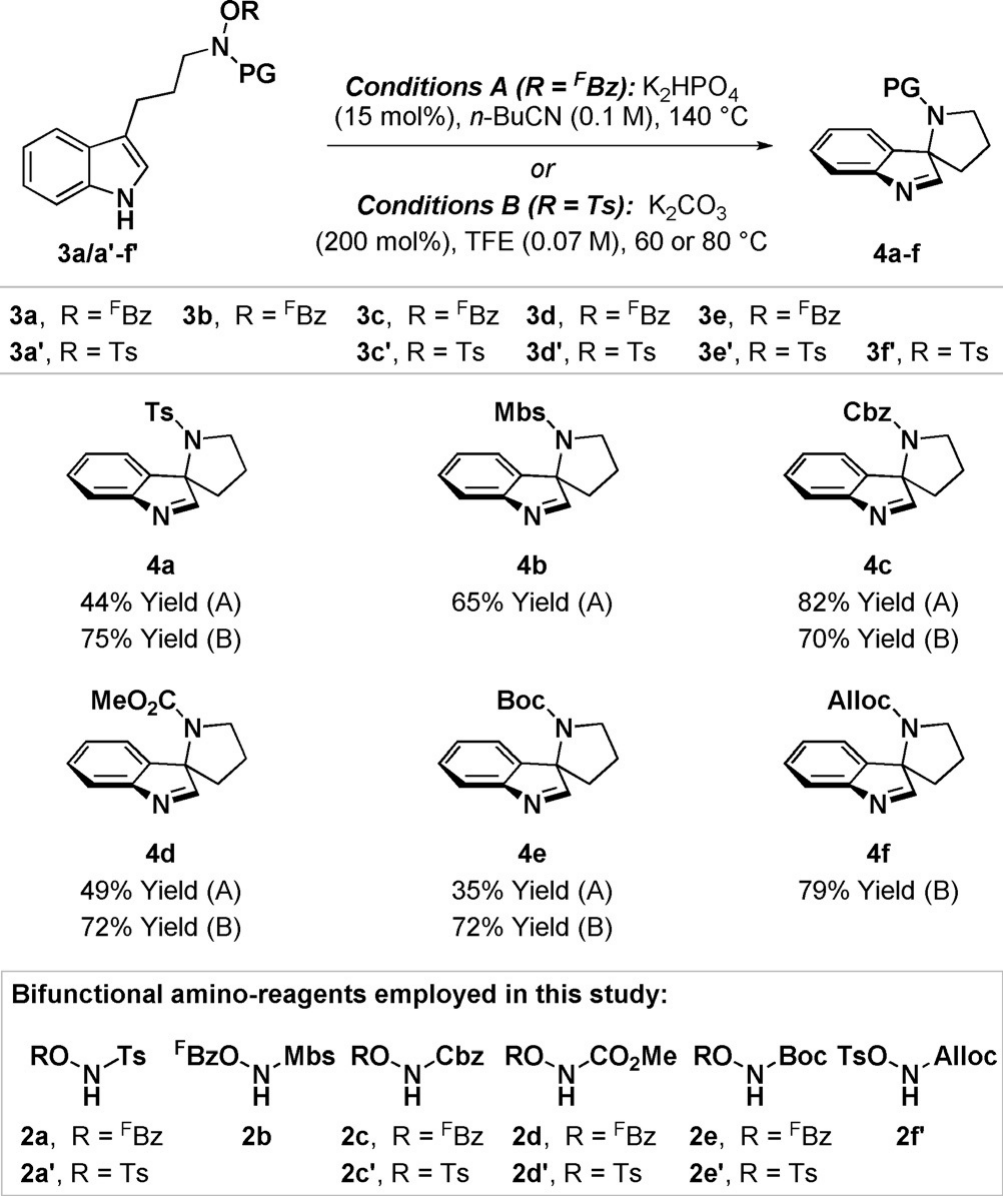

With efficient protocols in hand, we chose initially to evaluate scope with respect to indole‐based systems, primarily because of the lack of complementary methods for achieving this type of dearomatization (Table 2). The method is largely insensitive to substitution on the indole ring, with a variety of substrates **3 g**–**p** undergoing dearomatization to provide spirocyclic imines **4 g**–**p** in moderate to excellent yield. The exact reaction conditions were selected on a case‐by‐case basis, although, in general, Conditions B were preferable for processes involving Boc‐protected amines. The method also provides access to systems with additional substitution on the pyrrolidine ring. Modest diastereocontrol was observed for C‐3 substituted system **4 q**, but high levels of diastereoselectivity were achieved for C‐2 (**4 r**) and C‐4 (**4 s**–**u**) substituted variants. The alcohol precursor for **3 s** was accessed in high enantiopurity via chiral iminium ion catalyzed enantioselective addition of indole to crotonaldehyde (see the Supporting Information),^13^ and this allowed access to product **4 s** in 92 % *ee*. The stereochemical assignments of the major diastereomers of **4 r**–**u** were determined by nOe experiments, and the structure of **4 u** was confirmed unambiguously by single crystal X‐ray diffraction.^14^

**Table 2:** C−N bond forming dearomatizations of indole‐based systems.

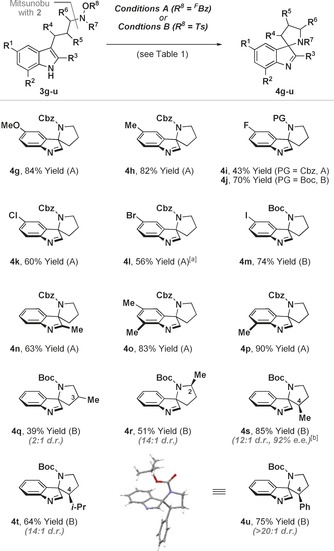

[a] K_2_HPO_4_ (200 mol %) was used. [b] Synthesized from enantioenriched **3 s** (92 % *ee*).

Further scope studies revealed that the approach can be extended to *para*‐phenol and *para*‐naphthol systems **5 a**–**e**, providing carbamate protected products **6 a**–**e** that are not accessible using the oxidative C−N bond forming dearomatization approach of Ciufolini and co‐workers (Table 3 A).5a–5e, 15 In each case, dearomatizing spirocyclization occurred cleanly and **6 a**–**e** were isolated in uniformly high yields. Similar efficiencies were observed for C−N bond forming dearomatizations of *ortho*‐phenols and naphthols; this provides access to enone products where the spirocyclic pyrrolidine is located adjacent to the newly unveiled ketone (Table 3 B). Thus, the method is generally applicable to the dearomatization of a key range of aromatic nucleophiles, with excellent efficiencies observed in all cases.

**Table 3:** C−N bond forming dearomatizations of phenols and naphthols.

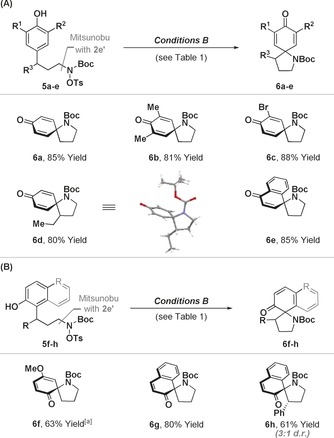

[a] K_2_CO_3_ (150 mol %) used in 1,2‐DCE.

The spirocyclic products accessed in this study are diverse, with each embodying electrophilic functionality that can be modified further (Figure 1). For example, reduction (NaBH_4_) of the imine moiety of **4 u** proceeded smoothly to provide indoline **7** in quantitative yield. C−C bond formations are also readily achieved; allyl‐Grignard addition to imine **4 u** occurred primarily from the face opposite the *N*‐Boc moiety to deliver **8** in 6:1 d.r. Vinyl‐cuprate addition to **6 e** was also diastereoselective (5:1 d.r.) and generated **9** in 77 % yield. The processes in Figure 1 are representative, and many other diversity oriented transformations can easily be envisaged. Note that in each case the products are accessed in only a few steps from the corresponding “planar” alcohol (see Scheme 1 B), which highlights the rapid complexity generation that can be achieved by harnessing bifunctional amino reagents **2** for dearomatizing heteroannulations.

**Figure 1:**
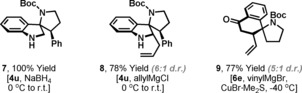
Derivatizations of the dearomatization products.

Our proposed mechanism for the processes described here is supported by a series of observations. Cross‐over experiments have confirmed that the N−O unit must be tethered to the aromatic undergoing dearomatization (Scheme 2 A); cyclization of *N*‐OTs system **3 j** in the presence of NH system **10** led to the formation of **4 j**, and **4 e** was not observed. Thus, the N−O bond acts as an internal oxidant only, and is not effective as an external oxidant. The conversion of **3 e′** to **4 e** is not affected by addition of TEMPO or BHT, which suggests that potential radical‐based pathways involving initial N−O homolysis are not operative.^16^ Additionally, we examined the behavior of alkenyl systems **11 a** and **11 b** because N‐centered radicals are known to undergo fast 5‐*exo* cyclization onto alkene acceptors (Scheme 2 B).^16^, ^17^ When **11 a** and **11 b** were exposed to optimized dearomatization conditions (in the presence or absence of 1,4‐cyclohexadiene (CHD) as a hydrogen atom donor) pyrrolidine product **12** was not observed. Although these results seemingly discount the intermediacy of an N‐centered radical, it is important to note that **11 a** and **11 b** lack a pendant arene, and so the results in Scheme 2 B do not rule out the possibility of N−O homolysis by intramolecular electron transfer from the indole or phenol unit.17a, 18 Nevertheless, at the current stage we favor a mechanistic pathway in line with that depicted in Scheme 1 B, wherein dearomatization occurs by direct nucleophilic attack of the arene onto the electrophilic nitrogen center. Nucleophilic substitution at N(sp^2^)−OR and N(sp^3^)−OR centers is unusual but has been invoked in other contexts.^4^, ^19^ Of particular relevance is the recent work of Wang and co‐workers, who disclosed intermolecular C3 aminations of indole anions using O‐sulfonyl activated *N*‐hydroxy amides and carbamates.19c In the current processes, the mild base (K_2_CO_3_ or K_2_HPO_4_) presumably enhances the nucleophilicity of the aromatic unit by deprotonating it before or during the C−N bond forming dearomatization step. Preliminary density functional theory (DFT) calculations support the viability of an S_N_2‐like mechanism. The dearomatizing spirocyclization of **5 a‐Me** (the methyl‐carbamate equivalent of **5 a**) to **6 a**‐**Me** was modelled and the computed free energy barrier (Δ*G*_solv_^≠^=25.1 kcal mol^−1^) is consistent with the reaction time required for complete conversion under Conditions B (93 % yield after 9 hours at 60 °C, see the Supporting Information) (Scheme 2 C).^20^ For Conditions A, fast protodecarboxylation of the pentafluorobenzoate leaving group to afford pentafluorobenzene regenerates the base (K_2_HPO_4_) and allows catalytic quantities of this component to be used. We have confirmed the formation of pentafluorobenzene by GCMS analysis of crude reaction mixtures, and our previous studies have shown that the protodecarboxylation process is facile.^21^ Under both Conditions A and B, minimal conversions are observed in the absence of base and hindered organic variants (e.g. 2,6‐di‐*tert*‐butylpyridine) are not suitable. These observations suggest an involved role for the base beyond simply sequestering the acid byproduct (TsOH, ^F^BzOH) and this may provide an avenue for the future realization of enantioselective processes.

**Scheme 2:**
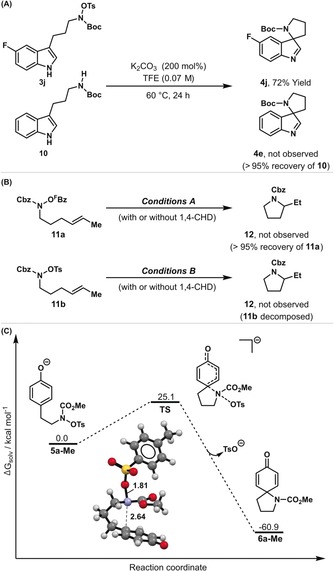
Preliminary mechanistic studies.^20^

In summary, we outline a conceptually simple approach to C−N bond forming dearomatization, wherein nucleophilic attack of an arene onto an electrophilic nitrogen center generates differentially protected spirocyclic pyrrolidines. The protocol offers unprecedented scope, encompassing indoles, phenols and naphthols as the nucleophilic component, and tolerating a range of N‐protecting groups; the ability to use synthetically flexible carbamates is particularly significant. The substrates are accessed directly using bifunctional amino reagents that incorporate the functionality required for the subsequent dearomatization step; this occurs under mildly basic conditions and avoids external oxidants. The spirocyclic pyrrolidine products obtained by this method are well recognized as privileged scaffolds in drug discovery, and the ability to access them in a flexible and direct manner is likely to be of wide interest.^22^

*Dedicated to the memory of István E. Markó*

## Conflict of interest

The authors declare no conflict of interest.

## Supporting information

As a service to our authors and readers, this journal provides supporting information supplied by the authors. Such materials are peer reviewed and may be re‐organized for online delivery, but are not copy‐edited or typeset. Technical support issues arising from supporting information (other than missing files) should be addressed to the authors.

Supplementary
