# Supplementary material for: A Simple and Broadly Applicable C−N Bond Forming Dearomatization Protocol Enabled by Bifunctional Amino Reagents
Source: Angew Chem Int Ed Engl. 2017 Oct 11;56(46):14531–5. doi: 10.1002/anie.201708176 (PMC5698738; doi:10.1002/anie.201708176)

Angewandte Chemie
Eine Zeitschrift der Gesellschaft Deutscher Chemiker

Supporting Information

A Simple and Broadly Applicable C−N Bond Forming Dearomatization Protocol Enabled by Bifunctional Amino Reagents

*Xiaofeng Ma, Joshua J. Farndon, Tom A. Young, Natalie Fey, and John F. Bower**

anie_201708176_sm_miscellaneous_information.pdf

Table of contents

1. General experimental details 2
2. Experimental procedures and data 3
3. Control experiments 96
4. Computational methods 98
5. References 106
6. Spectra of novel compounds 108

1. General experimental details

Unless stated, all materials were purchased from commercial sources (Acros, Aldrich, Alfa Aesar, Fluorochem and Strem) and used without any further treatment. Anhydrous 2,2,2-trifluoroethanol was obtained by drying over 4Å molecular sieves while other anhydrous solvents were obtained by passage through drying columns supplied by Anhydrous Engineering Ltd. Catalytic reactions were carried out in Young-type re-sealable tubes. Anhydrous solvents were obtained by distillation using standard procedures or by passage through drying columns supplied by Anhydrous Engineering Ltd. High-boiling solvents were removed from the reaction crudes employing rotary evaporators connected with high-vacuum pumps. Flash column chromatography (FCC) was performed using silica gel (Aldrich 40-63 µm, 230-400 mesh). Thin layer chromatography was performed using aluminium backed $60F_{254}$ silica plates. Visualization was achieved by UV fluorescence or a basic $KMnO_4$ solution and heat. Proton nuclear magnetic resonance spectra (NMR) were recorded at 400 MHz or 500 MHz. ^{13}C NMR spectra were recorded at 100 MHz or 125 MHz as stated. Chemical shifts (δ) are given in parts per million (ppm). Peaks are described as singlets (s), doublets (d), triplets (t), quartets (q), multiplets (m) and broad (br.). Coupling constants (J) are quoted to the nearest 0.5 Hz. All assignments of NMR spectra were based on 2D NMR data (COSY, HSQC and HMBC). *Where mixtures of isomers (e.g. diastereomers and/or rotamers) have been characterized together, they are referred to as A and B. Numbering systems for NMR signal assignments are specified on the structure and are not related to those used for the compound names.* In situ yields were determined by employing 1,3,5-trimethoxybenzene as internal standard. Mass spectra were recorded using a Brüker Daltonics FT-ICR-MS Apex 4e 7.0T FT-MS (ESI^+ mode). Infrared spectra were recorded in the range 4000-600 cm^{-1} on a Perkin Elmer Spectrum Two FTIR spectrometer as thin films or solids compressed on a diamond plate. Abbreviations used are: w (weak), m (medium) or s (strong). Melting points were determined using Stuart SMP30 melting point apparatus and are reported uncorrected. Enantiomeric excess was determined by integration of chromatograms peaks. Chiral SFC was performed on an Agilent 1260 Infinity SFC Control Module system equipped with a quaternary pump, diode array detector and column thermostat under the conditions specified.

2. Experimental procedures and data

General procedure A: Reduction of carboxylic acids or esters

To a solution of carboxylic acid/ester (1.0 eq.) in anhydrous THF (2-5 mL/mmol) at 0 °C was added $LiAlH_4$ (*equivalents specified*) dropwise. The reaction mixture was warmed to room temperature or refluxed overnight. Upon completion, the reaction mixture was cooled to 0 °C before addition of water (1 mL/g of $LiAlH_4$), 15 % aq. NaOH (1 mL/g of $LiAlH_4$) and a final portion of water (3.0 mL/g of $LiAlH_4$), the resulting mixture was dried over Na_2SO_4 and filtered through celite® and washed with Et_2O. The filtrate was concentrated *in vacuo* to afford the product.

General procedure B: TBS protection of phenol

To a solution of alcohol (1.0 eq.) in DMF (*approx*. 2mL/mmol) at 0 °C was added imidazole (3.3 eq.) and *tert*-butyldimethylsilyl chloride (2.2 eq.). The reaction was stirred at room temperature and monitored by TLC. Upon completion, the reaction was quenched by addition of H_2O and the organic phase extracted with hexane, dried over $MgSO_4$ and concentrated *in vacuo*. To the crude reaction mixture was added MeOH (1 mL/mmol), THF (1 mL/mmol) and aq. K_2CO_3 (2.0 eq.) After stirring for 12 h the reaction was quenched with aq. 1 M HCl at 0 °C (until pH *approx*. 3). The mixture was extracted with Et_2O (3 x 20 mL), dried over $MgSO_4$, filtered and concentrated *in vacuo*. The crude reaction mixture was purified by flash column chromatography.

General procedure C: Mitsunobu reaction using THF/PhMe as solvent

To a solution of alcohol (1.0 eq.), hydroxylamine-derived pronucleophile (1.2 eq.) and PPh_3 (1.2 – 1.5 eq.) in anhydrous THF:PhMe (1:4, 1 mL/mmol) at 0 °C was added a solution of DIAD (1.2 – 1.5 eq.) in anhydrous PhMe (0.3 mL/mmol) dropwise. The reaction mixture was stirred at room temperature overnight before being concentrated *in vacuo* and loaded directly onto silica gel for purification of the product by flash column chromatography.

General procedure D: Mitsunobu procedure using THF as solvent

Following a literature procedure[1], diisopropyl azodicarboxylate (1.2 eq.) was added at 0 ^{o}C to a stirring solution of triphenylphosphine (1.2 eq.) in anhydrous THF (*approx*. 2 mL/mmol) under a nitrogen atmosphere. After 30 min stirring at this temperature, a solution of alcohol (1.0 eq.) and hydroxylamine-derived pronucleophile (1.2 eq.) in anhydrous THF (*approx*. 2 mL/mmol) were added. The reaction was stirred at 0 ^{o} C for 1 h after which it was stirred at room temperature until compelition. The reaction mixture was concentrated *in vacuo* and purified by flash column chromatography.

General procedure E: Removal of silyl protecting group with TBAF/AcOH

To a solution of silyl ether (1.0 eq.) in THF (*approx*. 20mL/mmol) at 0 ^{o}C was added a solution of 1:1 TBAF/AcOH (*equivalents specified*, 0.1 M in THF). The reaction mixture was stirred at room temperature and monitored by TLC. Upon completion, the reaction mixture was quenched with water (10 mL), extracted with EtOAc (2 x 10 mL), washed with sat. aq. $NaHCO_3$ (10 mL) and brine (10 mL), dried over Na_2SO_4, filtered and the concentrated *in vacuo*. The crude product was purified by flash column chromatography.

General procedure F (‘Conditions A’): Dearomatization procedure for OBz^{F} substrates

A flame-dried re-sealable tube, fitted with a rubber septum, was charged with cyclization substrate and base (K_2HPO_4, 15 mol% or 200 mol%). The tube was purged with nitrogen and anhydrous nBuCN (0.1 M) was added via syringe. The tube was sealed and heated at 140 °C for the time noted. The reaction mixture was concentrated *in vacuo* and the crude mixture was purified by flash column chromatography to afford the pure product.

General procedure G (‘Conditions B’): Dearomatization procedure for OTs substrates

A flame-dried re-sealable tube, fitted with a rubber septum, was charged with fresh prepared cyclization substrate, base (K_2CO_3, 200 mol%). The tube was purged with nitrogen, anhydrous 2,2,2-Trifluoroethanol (TFE) (0.067 M) was added via syringe. The tube was sealed and heated at 60 °C or 80 °C for the time noted. The reaction mixture was concentrated *in vacuo* and the crude mixture was purified by flash column chromatography to afford the pure product.

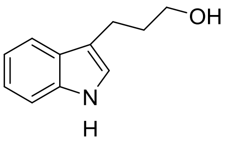

S1

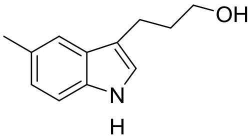

S2

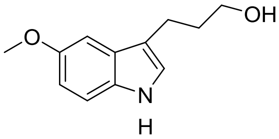

S3

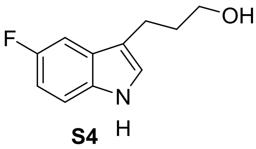

S4

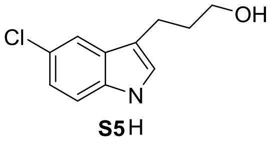

S5

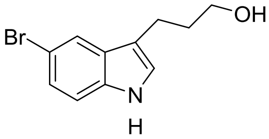

S6

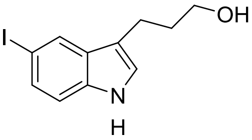

S7

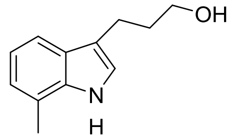

S8

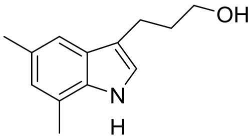

S9

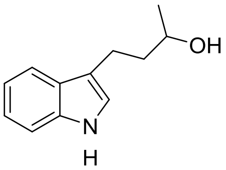

S10

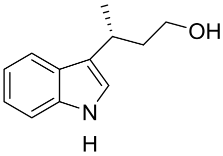

S11

Alcohols **S1-S9** were synthesized according to a literature procedure.[2] **S10** was synthesized according to a literature procedure.[3] **S11** was synthesized according to a literature procedure (92% ee).[4]

The enantiomeric purity of compound **S11** was determined by chiral SFC (Chiralpak IE, CO_2:*i*-PrOH 95:5, 3 mL/min, 25 °C, P_{outlet} 150 Bar, Sample in solution with 100% CH_2Cl_2) against a racemic standard prepared under another method (see below); t_R (minor) = 14.4 min and t_R (major) = 16.6 min.

```
Method           : C:\CHEM32\2\METHODS\MXF.M
Last changed     : 16/06/2017 16:59:03 by SYSTEM
Sample Info      : CO2: IPA 95:5 20MIN

Additional Info : Peak(s) manually integrated
```

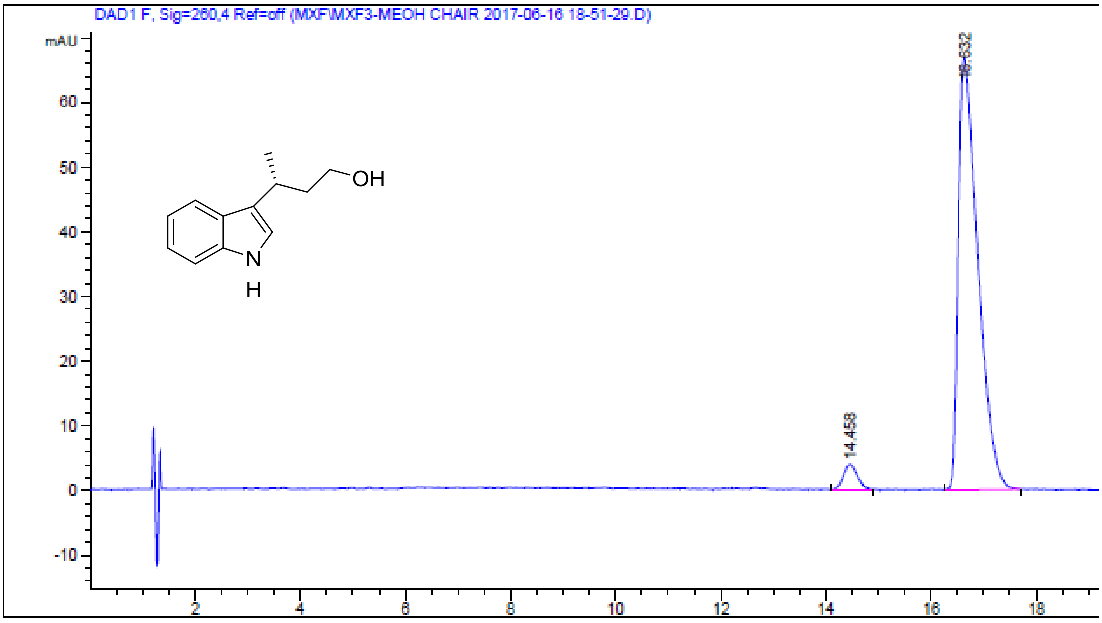


```
=====================================================================
                        Area Percent Report
=====================================================================

Sorted By             :      Signal
Multiplier            :      1.0000
Dilution              :      1.0000
Sample Amount:               :      30.00000  [ng/ul]   (not used in calc.)
Use Multiplier & Dilution Factor with ISTDs


Signal 1: DAD1 F, Sig=260,4 Ref=off
```

Peak #	RetTime [min]	Type	Width [min]	Area [mAU*s]	Height [mAU]	Area %
1	14.458	BB	0.2687	70.64447	3.87548	3.9335
2	16.632	BB	0.3795	1725.33679	66.75536	96.0665

Totals : 1795.98126 70.63083

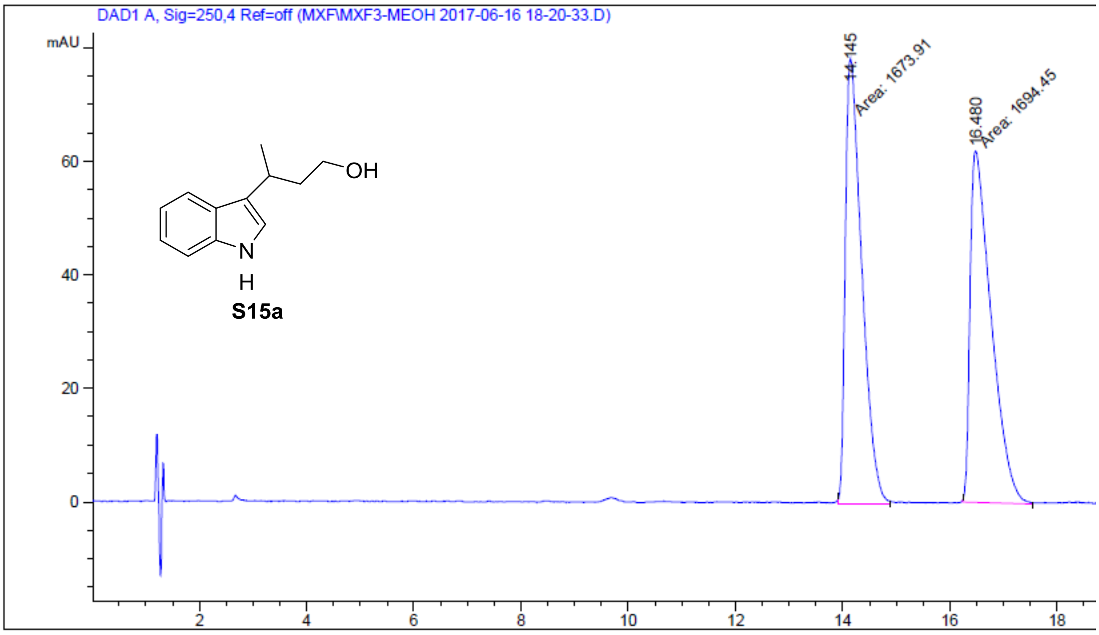


```
=====================================================================
                         Area Percent Report
=====================================================================

Sorted By             :      Signal
Multiplier            :      1.0000
Dilution              :      1.0000
Sample Amount:               :      30.00000  [ng/ul]   (not used in calc.)
Use Multiplier & Dilution Factor with ISTDs


Signal 1: DAD1 A, Sig=250,4 Ref=off

Peak RetTime Type  Width     Area      Height     Area
  #   [min]        [min]   [mAU*s]     [mAU]        %
----|-------|----|-------|----------|----------|--------|
   1  14.145 MM    0.3554 1673.91235   78.49419  49.6951
   2  16.480 MM    0.4556 1694.45081   61.98769  50.3049

Totals :                  3368.36316  140.48188
```

3-(5,7-Dimethyl-1*H*-indol-3-yl)propan-1-ol (S9)

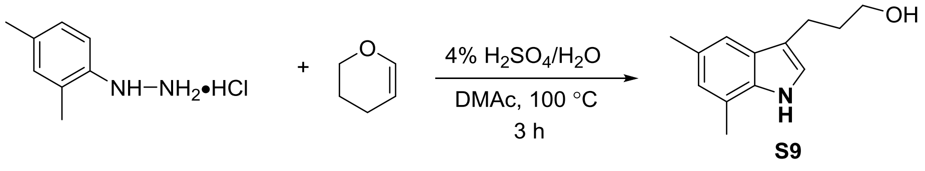

3-(5,7-Dimethyl-1*H*-indol-3-yl)propan-1-ol **S9** was synthesized according to a literature procedure[1]: To a solution of phenyhydrazine-HCl (1.00 g, 6.92 mmol) in 4% H_2SO_4 (aq) (10 mL) and *N*,*N*-dimethyl acetamide (DMAc,10 mL) at 100 °C was added dihydrofuran (630 μL, 6.92 mmol) dropwise over 2 minutes. The reaction was aged for 3 hours, then cooled to room

temperature, extracted with ethyl acetate (20 mL) and washed with water (20 mL × 3). The crude material was purified by flash chromatography (33–50% EtOAc/Hexane) to afford **S9** (1.28 g, 91%) as a light red oil.

IR (film) ν_{max} / cm^{-1}: 3411 (br, s), 3312 (br, m), 2917 (s), 1708 (m), 1441 (s), 1035 (s), 843 (s).

^{1}H NMR (400 MHz, $CDCl_3$) δ 7.86 (s, 1H), 7.28 (s, 1H), 6.94 (d, *J* = 2.3 Hz, 1H), 6.87 (s, 1H), 3.74 (t, *J* = 6.4 Hz, 2H), 2.85 (t, *J* = 7.5 Hz, 2H), 2.47 (s, 3H), 2.45 (s, 3H), 2.00 (p, *J* = 6.8 Hz, 2H) 1.76 (br. s, 1H).

^{13}C NMR (101 MHz, $CDCl_3$) δ 134.39, 128.73, 127.30, 124.33, 121.35, 120.09, 116.26, 115.92, 77.48, 77.16, 76.84, 62.79, 33.04, 21.63, 21.57, 16.61.

ESI-HRMS: m/z calculated for $C_{13}H_{17}NNaO[M+Na]^+$: 226.1202; found: 226.1207.

3-(2-Methyl-1*H*-indol-3-yl)propan-1-ol (S13)

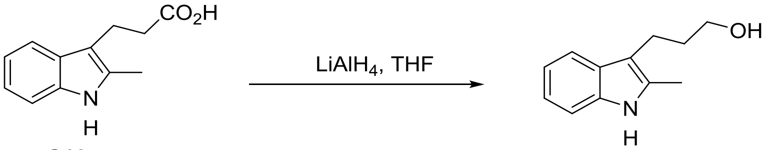

S12 **S13**

Carboxylic acid **S13** was synthesized according to a literature procedure[2]. The spectroscopic properties of **S13** was consistent with the data available in the literature.[5]

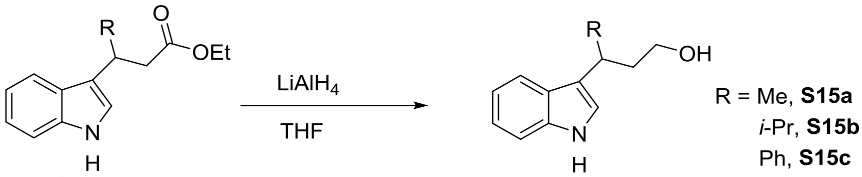

S14

Esters **S14a-S14c** were synthesized according to a literature procedure.[6] The spectroscopic properties of **S15a** and **S15c** were consistent with the data available in the literature.[7]

3-(1*H*-Indol-3-yl)-4-methylpentan-1-ol (S15b)

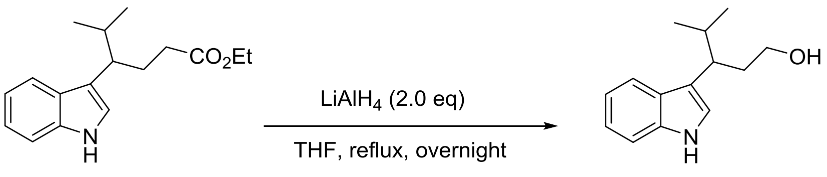

S14b **S15b**

General procedure A: Ethyl 4-(1*H*-indol-3-yl)-5-methylhexanoate **S14b** (6.69 g, 25.8 mmol), $LiAlH_4$ (1.0 M in THF, 51.6 mL) and 50 mL THF were employed to afford the title compound **S15b** (4.77 g, 85%) as light yellow oil.

IR (film) ν_{max} / cm^{-1}: 3412 (s), 3303 (br, m), 2954 (m), 1456 (s), 1010 (s), 737 (s).

^{1}H NMR (400 MHz, $CDCl_3$) δ 8.22 (s, 1H), 7.68 (d, *J* = 8.0 Hz, 1H), 7.42 – 7.28 (m, 1H), 7.23 (t, *J* = 7.6 Hz, 1H), 7.15 (t, *J* = 7.5 Hz, 1H), 6.86 (d, *J* = 2.5 Hz, 1H), 3.68 – 3.43 (m, 2H), 2.84 (ddt, *J* = 8.5, 5.9, 2.8 Hz, 1H), 2.23 – 1.78 (m, 4H), 0.99 (dd, *J* = 6.8, 1.9 Hz, 3H), 0.97 – 0.82 (m, 3H).

^{13}C NMR (101 MHz, $CDCl_3$) δ 136.54, 127.74, 121.94, 121.69, 119.74, 119.20, 118.18, 111.27, 77.48, 77.16, 76.84, 62.30, 40.34, 35.43, 33.12, 20.95, 20.70.

ESI-HRMS: m/z calculated for $C_{14}H_{19}NNaO[M+Na]^+$: 240.1359; found: 240.1364.

4-Methyl-*N*-((perfluorobenzoyl)oxy)benzenesulfonamide (2a)

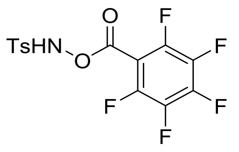

This compound was prepared according to our reported procedure with a slight modification.[8] To a suspension of *N*-tosylhydroxylamine (9.68 g, 51.7 mmol) and pentafluorobenzoic acid (10.9 g, 51.4 mmol) in CH_2Cl_2 (240 mL) at 0 °C was added a solution of *N*-*N'*-dicyclohexylcarbodiimide (11.6 g, 56.2 mmol) in CH_2Cl_2 (240 mL) dropwise. The resulting mixture was stirred at 0 °C overnight before filtration to remove the white precipitate. The filtrate was concentrated *in vacuo* and the crude mixture was purified by FCC (33–50% EtOAc/Hexane) to afford **2a** (15.3 g, yield 78%) as a colorless crystalline solid.

The spectroscopic properties were consistent with the data available in the literature.[8]

4-Methyl-*N*-(tosyloxy)benzenesulfonamide (2a')

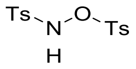

This compound was prepared according to a literature reported procedure.[9]

The spectroscopic properties were consistent with the data available in the literature.[9]

4-Methoxy-*N*-((perfluorobenzoyl)oxy)benzenesulfonamide (2b)

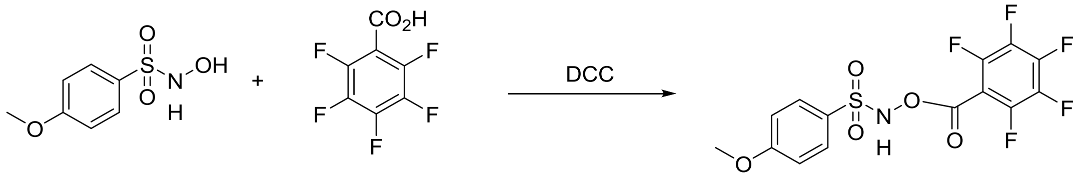

To a suspension of *N*-hydroxy-4-methoxybenzenesulfonamide[10] (1.55 g, 7.65 mmol) and pentafluorobenzoic acid (1.67 g, 8.42 mmol) in CH_2Cl_2 (30 mL) at 0 °C was added a solution of *N*-*N*'-dicyclohexylcarbodiimide (1.74 g, 8.42 mmol) in CH_2Cl_2 (30 mL) dropwise. The resulting mixture was stirred at 0 °C overnight before filtration to remove the white precipitate. The filtrate was concentrated *in vacuo* and the crude mixture was purified by flash column chromatography (20–33% EtOAc/Hexane) to give the title compound (2.68 g, 88%) as a colorless solid.

m.p.: 101 - 103 ° C (EtOAc/Hexane).

IR (film) ν_{max} / cm^{-1}: 2987 (m), 1780 (m), 1596 (m), 1499 (s), 1165 (s), 1007 (m).

^{1}H NMR (400 MHz, $CDCl_3$) δ 8.96 (br s, 1H), 7.96 – 7.87 (m, 2H), 7.08 – 6.93 (m, 2H), 3.90 (s, 3H).

^{13}C NMR (101 MHz, $CDCl_3$) δ 164.88, 131.46, 126.29, 114.80, 55.97.

The aromatic signals corresponding to the pentafluorobenzoyl group could not be resolved due to their weak intensity.

ESI-HRMS: m/z calculated for $C_{14}H_8F_5NNaO_5S[M+Na]^+$: 419.9936; found: 419.9934.

^{19}F NMR (377 MHz, $CDCl_3$) δ -132.52 – -139.03 (m, 2F), -144.10 (tt, *J* = 21.0, 6.3 Hz, 1F), -158.53 – -158.98 (m, 2F).

Benzyl (perfluorobenzoyl)oxycarbamate (2c)

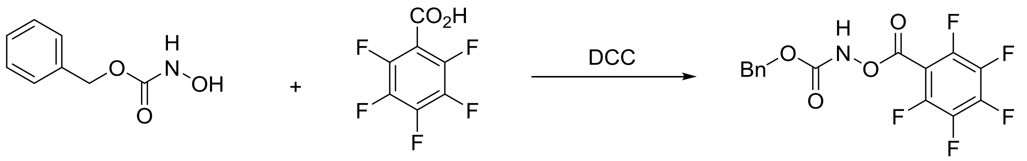

To a suspension of benzyl hydroxycarbamate[11] (9.72 g, 58.2 mmol) and pentafluorobenzoic acid (12.33 g, 58.2 mmol) in CH_2Cl_2 (200 mL) at 0 °C was added a solution of *N*-*N*'-dicyclohexylcarbodiimide (13.2 g, 64.0 mmol) in CH_2Cl_2 (200 mL) dropwise. The resulting

mixture was stirred at 0 °C overnight before filtration to remove the white precipitate. The filtrate was concentrated *in vacuo* and the crude mixture was purified by flash column chromatography (20% EtOAc/Hexane) to give the title compound (15.6 g, 74%) as a colorless solid.

m.p.: 112 - 113 °C (EtOAc/Hexane).

IR (solid) ν_{max}/cm^{-1}: 3237 (s, br), 2971 (m), 2987 (m), 2901 (m), 1779 (m), 1755 (s), 1494 (s).

^{1}H NMR (400 MHz, $CDCl_3$) δ 8.35 (1H, br s), 7.37 (5H, s), 5.26 (2H, s).

^{13}C NMR (101 MHz, $CDCl_3$) δ 155.7, 134.6, 128.8, 128.7, 128.4, 69.0, 68.9.

The aromatic signals corresponding to the pentafluorobenzoyl group could not be resolved due to their weak intensity.

^{19}F NMR (377 MHz, $CDCl_3$) δ -135.15 (dq, *J* = 18.9, 7.2, 6.6 Hz, 2F), -145.10 (tt, *J* = 20.9, 6.0 Hz, 1F), -158.49 – -159.91 (m, 2F).

ESI-HRMS: m/z calculated for $C_{15}H_{18}F_5NNaO_4[M+Na]^+$: 384.0266; found: 384.0276.

Benzyl (tosyloxy)carbamate (2c')

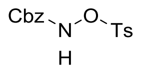

This compound was prepared according to a literature reported procedure.[11]

The spectroscopic properties were consistent with the data available in the literature.[11]

Methyl hydroxycarbamate

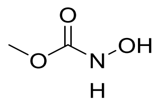

This compound was prepared according to a literature reported procedure.[12]

The spectroscopic properties were consistent with the data available in the literature.[13]

Methyl (perfluorobenzoyl)oxycarbamate (2d)

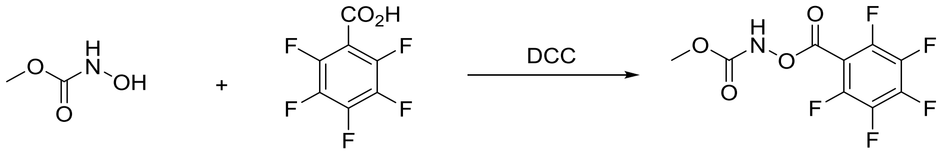

To a suspension of methyl hydroxycarbamate (1.00 g, 11.0 mmol) and pentafluorobenzoic acid (2.3 g, 11.0 mmol) in CH_2Cl_2 (11 mL) at 0 ºC was added a solution of *N*-*N*'-dicyclohexylcarbodiimide (2.50 g, 12.1 mmol) in CH_2Cl_2 (11 mL) dropwise. The resulting mixture was stirred at 0 ºC overnight before filtration to remove the white precipitate. The filtrate was concentrated *in vacuo* and the crude mixture was purified by flash column chromatography (20% EtOAc/Hexane) to give the title compound (2.13 g, 68%) as a colorless oil.

IR (film) ν_{max} / cm^{-1}: 3258 (br, m), 1783 (m), 1747 (s), 1499 (s), 1328 (m), 1181 (s), 997 (s).

^{1}H NMR (400 MHz, $CDCl_3$) δ 6.16 (br s, 1H), 3.82 (s, 3H).

^{13}C NMR (101 MHz, $CDCl_3$) δ 158.62, 156.78, 147.43, 147.32, 145.77, 145.72, 145.59, 144.84, 144.78, 144.71, 143.16, 143.12, 139.43, 139.28, 139.21, 139.14, 139.08, 136.90, 136.83, 136.77, 136.70, 136.56, 105.04, 104.88, 104.84, 104.69, 54.03.

^{19}F NMR (377 MHz, $CDCl_3$) δ -146.00, -156.66, -162.25.

ESI-HRMS: m/z calculated for $C_9H_4F_5NNaO_4$$[M+Na]^+$: 307.9953; found: 307.9963.

Methyl (tosyloxy)carbamate (2d')

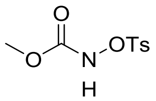

This compound was prepared according to a literature reported procedure.[14]

The spectroscopic properties were consistent with the data available in the literature.[14]

***tert*-Butyl hydroxycarbamate**

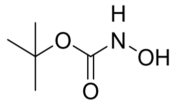

This compound was prepared according to a literature reported procedure.[15]

The spectroscopic properties were consistent with the data available in the literature.[15]

***tert*-Butyl (perfluorobenzoyl)oxycarbamate (2e)**

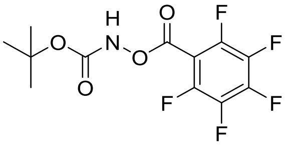

This compound was prepared according to a literature reported procedure.[11]

The spectroscopic properties were consistent with the data available in the literature.[11]

tert-Butyl tosyloxycarbamate (2e')

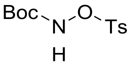

This compound was prepared according to a literature reported procedure.[11]

The spectroscopic properties were consistent with the data available in the literature.[11]

Allyl tosyloxycarbamate (2f')

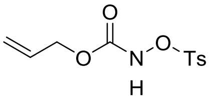

This compound was prepared according to a literature reported procedure.[16]

The spectroscopic properties were consistent with the data available in the literature.[16]

***N*-(3-(1*H*-Indol-3-yl)propyl)-4-methyl-*N*-((perfluorobenzoyl)oxy)benzenesulfonamide (3a)**

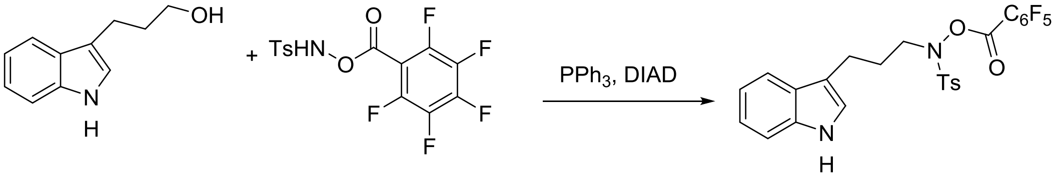

__General procedure C__**:** 3-(1*H*-Indol-3-yl)propan-1-ol (1.46 g, 8.33 mmol), 4-methyl-*N*-((perfluorobenzoyl)oxy)benzenesulfonamide **2a** (3.8 g, 9.97 mmol), PPh_3 (2.53 g, 12.50 mmol), DIAD (2.5 mL, 12.50 mmol), toluene (40 mL) and THF (10 mL) were employed. Purification of the product by flash column chromatography (1% EtOAc/PhMe) afforded **3a** (2.99 g, 67%) as a crystalline colorless solid.

m.p.: 149 -151 °C (EtOAc/Hexane).

IR (film) ν_{max} / cm^{-1}: 3675 (m), 2988 (s), 1394 (m), 1260 (m), 1057 (s), 1066 (s), 750 (s).

^{1}H NMR (500 MHz, $CDCl_3$) δ 7.99 (s, 1H), 7.76 (d, *J* = 8.0 Hz, 2H), 7.55 (d, *J* = 7.9 Hz, 1H), 7.35 (t, *J* = 7.7 Hz, 3H), 7.19 (t, *J* = 7.5 Hz, 1H), 7.14 – 7.04 (m, 2H), 3.24 (s, 2H), 2.97 (t, *J* = 7.1 Hz, 2H), 2.45 (s, 3H), 1.95 (p, *J* = 6.9 Hz, 2H).

^{13}C NMR (126 MHz, $CDCl_3$) δ 145.94, 136.52, 130.08, 130.00, 129.72, 127.37, 122.42, 122.08, 119.34, 118.84, 114.71, 111.29, 52.21, 26.97, 21.87, 21.82.

The aromatic signals corresponding to the pentafluorobenzoyl group could not be resolved due to their weak intensity.

^{19}F NMR (377 MHz, $CDCl_3$) δ -135.75 (s, 2F), -145.93 (s, 1F), -158.94 (s, 2F).

ESI-HRMS: m/z calculated for $C_{25}H_{19}FN_2NaO_4S$[M+Na]$^+$: 561.0878; found: 561.0883.

N-(3-(1*H*-Indol-3-yl)propyl)-4-methyl-*N*-(tosyloxy)benzenesulfonamide (3a')

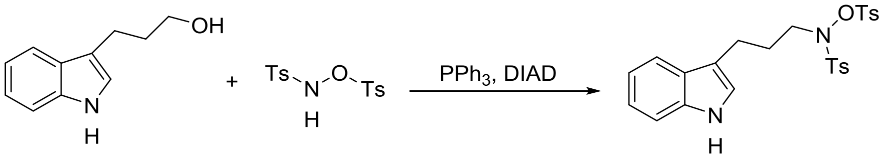

General procedure D: 3-(1*H*-Indol-3-yl)propan-1-ol (0.50 g, 2.85 mmol), TsNHOTs **2a'** (1.17 g, 3.42 mmol), PPh_3 (1.12 g, 4.27 mmol), DIAD (0.84 mL, 4.27 mmol) and THF (20.0 mL) were employed. Purification of the product by flash column chromatography (1% EtOAc/PhMe) afforded **3a'** (1.01 g, 71%) as a colorless foam.

IR (film) ν_{max} / cm^{-1}: 3425 (w), 2988 (s), 1380 (s), 1178 (s),1166 (s), 1157 (s), 748 (s).

^{1}H NMR (400 MHz, CD_2Cl_2) δ 8.14 (s, 1H), 7.88 (d, *J* = 7.7 Hz, 2H), 7.54 (t, *J* = 5.6 Hz, 3H), 7.38 (d, *J* = 7.7 Hz, 3H), 7.29 (d, *J* = 7.8 Hz, 2H), 7.19 (t, *J* = 7.6 Hz, 1H), 7.10 (t, *J* = 7.4 Hz, 1H), 7.01 (s, 1H), 3.18 (s, 2H), 2.80 (t, *J* = 7.2 Hz, 2H), 2.45 (s, 3H), 2.43 (s, 3H), 2.09 – 1.82 (m, 2H).

^{13}C NMR (101 MHz, CD_2Cl_2) δ 146.99, 146.63, 136.95, 131.73, 130.35, 130.24, 130.17, 130.10, 129.94, 127.90, 122.36, 119.62, 119.23, 115.40, 111.65, 56.12, 27.96, 22.67, 22.11, 22.00.

ESI-HRMS: m/z calculated for $C_{25}H_{26}N_2NaO_5S$[M+Na]$^+$: 521.1169; found: 521.1175.

N-(3-(1H-Indol-3-yl)propyl)-4-methoxy-N-((perfluorobenzoyl)oxy)benzenesulfonamide (3b)

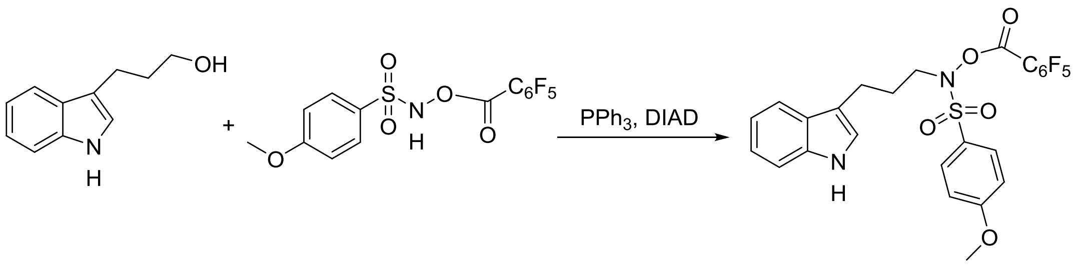

<u>General procedure D</u>: 3-(1*H*-Indol-3-yl)propan-1-ol (0.25 g, 1.43 mmol), 4-methoxy-*N*-((perfluorobenzoyl)oxy)benzenesulfonamide **2b** (679 mg, 1.71 mmol), PPh_3 (449 mg, 1.71 mmol), DIAD (0.36 mL, 1.71 mmol) and THF (12.0 mL) were employed. Purification of the product by flash column chromatography (20% Hexane/PhMe then 1% EtOAc/PhMe) afforded **3b** (561 mg, 71%) as a crystalline colorless solid.

m.p.: 166 - 168 °C (EtOAc/Hexane).

IR (film) ν_{max} / cm^{-1}: 3675 (m), 2988 (s), 1394 (m), 1166 (s), 1157 (s), 750 (m).

^{1}H NMR (400 MHz, $CDCl_3$) δ 7.98 (s, 1H), 7.81 (d, *J* = 9.0 Hz, 2H), 7.55 (d, *J* = 7.9 Hz, 1H), 7.36 (d, *J* = 8.1 Hz, 1H), 7.19 (t, *J* = 7.1 Hz, 1H), 7.09 (dd, *J* = 12.5, 4.6 Hz, 2H), 7.00 (d, *J* = 9.0 Hz, 2H), 3.89 (s, 3H), 3.23 (s, 2H), 2.97 (t, *J* = 7.1 Hz, 2H), 2.01 – 1.89 (m, 2H).

^{13}C NMR (101 MHz, $CDCl_3$) δ 164.59, 136.51, 133.44, 131.97, 127.38, 124.34, 122.40, 122.08, 119.35, 118.85, 114.74, 114.58, 111.29, 66.43, 63.93, 52.13, 26.97, 21.84.

The aromatic signals corresponding to the pentafluorobenzoyl group could not be resolved due to their weak intensity.

^{19}F NMR (377 MHz, $CDCl_3$) δ -134.99 – -136.45 (m, 2F), -146.18 (tt, *J* = 20.9, 5.2 Hz, 1F), -158.26 – -160.00 (m, 2F).

ESI-HRMS: m/z calculated for $C_{25}H_{19}F_5N_2NaO_5S[M+Na]^+$: 577.0827; found: 577.0831.

Benzyl (3-(1H-Indol-3-yl)propyl)((perfluorobenzoyl)oxy)carbamate (3c)

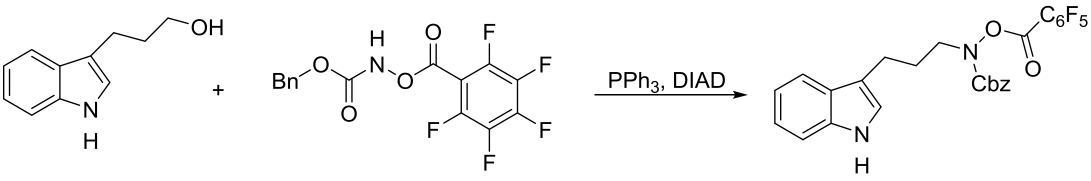

<u>General procedure C</u>: 3-(1*H*-Indol-3-yl)propan-1-ol (1.22 g, 6.99 mmol), benzyl (perfluorobenzoyl)oxycarbamate **2c** (3.03 g, 8.39 mmol), PPh_3 (2.75 g, 10.49 mmol), DIAD (2.10 mL, 10.49 mmol), toluene (33.0 mL) and THF (8.4 mL) were employed. Purification of the product by flash column chromatography (0–1%EtOAc/PhMe) afforded **3c** (2.06 g, 57%) as a crystalline colorless solid.

m.p.: 96 - 98 °C (EtOAc/Hexane).

IR (film) ν_{max} / cm^{-1}: 3416 (br, w), 1780 (m), 1721 (m), 1499 (s), 1173 (s), 1003 (m).

^{1}H NMR (400 MHz, $CDCl_3$) δ 7.96 (s, 1H), 7.56 (d, *J* = 7.8 Hz, 1H), 7.38 – 7.33 (m, 6H), 7.21 – 7.14 (m, 1H), 7.12 – 7.05 (m, 1H), 6.94 (d, *J* = 2.0 Hz, 1H), 5.23 (s, 2H), 3.84 (t, *J* = 6.9 Hz, 2H), 2.87 (t, *J* = 7.3 Hz, 2H), 2.08 (p, *J* = 7.1 Hz, 2H).

^{13}C NMR (101 MHz, $CDCl_3$) δ 155.65, 153.99, 135.33, 131.59, 128.70, 128.60, 128.27, 127.76, 122.52, 114.77, 112.20, 111.91, 100.68, 68.90, 55.97, 50.85, 27.10, 22.00.

The aromatic signals corresponding to the pentafluorobenzoyl group could not be resolved due to their weak intensity.

^{19}F NMR (377 MHz, $CDCl_3$) δ -135.40 – -136.18 (m, 2F), -145.96 (tt, *J* = 20.9, 5.4 Hz, 1F), -158.44 – -160.01 (m, 2F).

ESI-HRMS: m/z calculated for $C_{26}H_{19}F_5N_2NaO_4$[M+Na]$^+$: 541.1157; found: 541.1172.

Benzyl (3-(1*H*-indol-3-yl)propyl)(tosyloxy)carbamate (3c’)

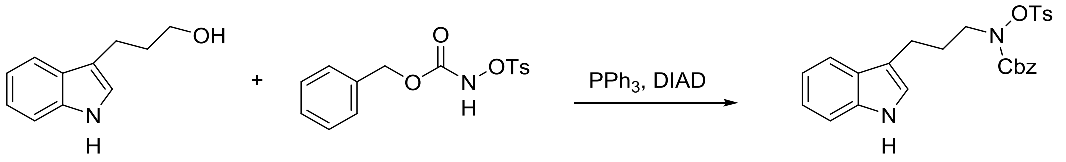

<u>General procedure D</u>: 3-(1*H*-Indol-3-yl)propan-1-ol (0.50 g, 2.85 mmol), CbzNHOTs **2c’** (1.12 g, 3.42 mmol), PPh_3 (1.12 g, 4.27 mmol), DIAD (0.84 mL, 4.27 mmol) and THF (20.0 mL) were employed. Purification of the product by flash column chromatography (PhMe) afforded **3c’** (0.79 g, 58%) as a colorless viscous oil.

IR (film) ν_{max} / cm^{-1}: 3420 (br, m), 1723 (s), 1457 (m), 1378 (s), 1191 (s), 1178 (s), 745 (s).

^{1}H NMR (400 MHz, CD_2Cl_2) δ 7.71 (d, *J* = 8.3 Hz, 2H), 7.56 – 7.48 (m, 1H), 7.40 – 7.33 (m, 3H), 7.23 – 7.13 (m, 5H), 7.12 – 7.05 (m, 1H), 6.98 (s, 1H), 4.92 (s, 2H), 3.67 (s, 2H), 2.73 (t, *J* = 7.5 Hz, 2H), 2.39 (d, *J* = 1.9 Hz, 3H), 2.13 – 1.91 (m, 2H).

^{13}C NMR (101 MHz, CD_2Cl_2) δ 157.20, 146.66, 136.92, 135.69, 131.36, 130.14, 129.82, 129.69, 128.98, 128.94, 128.63, 127.88, 126.65, 122.36, 122.02, 119.60, 119.16, 115.46, 111.63, 69.15, 53.49, 26.75, 22.55, 22.08.

ESI-HRMS: m/z calculated for $C_{26}H_{27}N_2O_5S[M+H]^+$: 479.1635; found: 479.1634.

Methyl (3-(1*H*-indol-3-yl)propyl)((perfluorobenzoyl)oxy)carbamate (3d)

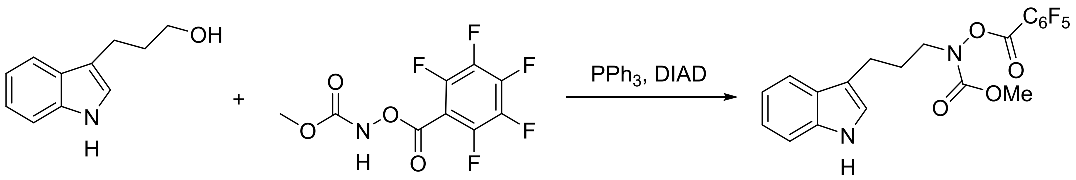

General procedure C: 3-(1*H*-Indol-3-yl)propan-1-ol (0.5 g, 2.85 mmol), methyl (perfluorobenzoyl)oxycarbamate **2d** (976 mg, 3.42 mmol), PPh_3 (1.12 g, 4.27 mmol), DIAD (0.84 mL, 4.27 mmol), THF (3.0 mL) and toluene (12.0 mL) were employed. Purification of the product by flash column chromatography (PhMe) afforded **3d** (1.0 g, 80%) as a crystalline colorless solid.

m.p.: 135 - 137 ° C (EtOAc/Hexane).

IR (film) ν_{max} / cm^{-1}: 3417 (br, w), 1779 (s), 1721 (s), 1524 (m), 1497 (s), 1325 (m), 1171 (s), 1000 (s), 741 (s).

^{1}H NMR (400 MHz, $CDCl_3$) δ 7.96 (s, 1H), 7.57 (d, *J* = 7.9 Hz, 1H), 7.34 (d, *J* = 8.1 Hz, 1H), 7.17 (t, *J* = 7.5 Hz, 1H), 7.08 (dd, *J* = 7.7, 7.2 Hz, 1H), 7.00 (d, *J* = 2.1 Hz, 1H), 3.83 – 3.78 (m, 5H), 2.86 (t, *J* = 7.4 Hz, 2H), 2.14 – 1.95 (m, 2H).

^{13}C NMR (101 MHz, $CDCl_3$) δ 156.33, 136.47, 127.41, 122.10, 121.64, 119.32, 118.84, 115.19, 111.22, 77.48, 77.16, 76.84, 54.11, 50.97, 27.26, 22.05.

The aromatic signals corresponding to the pentafluorobenzoyl group could not be resolved due to their weak intensity.

^{19}F NMR (377 MHz, $CDCl_3$) δ -135.87 (qd, *J* = 12.5, 6.6 Hz, 2F), -145.98 (tt, *J* = 21.0, 5.4 Hz, 1F), -159.16 – -159.36 (m, 2F).

ESI-HRMS: m/z calculated for $C_{20}H_{15}F_5N_2NaO_4[M+Na]^+$: 465.0844; found: 465.0844.

Methyl (3-(1*H*-indol-3-yl)propyl)(tosyloxy)carbamate (3d')

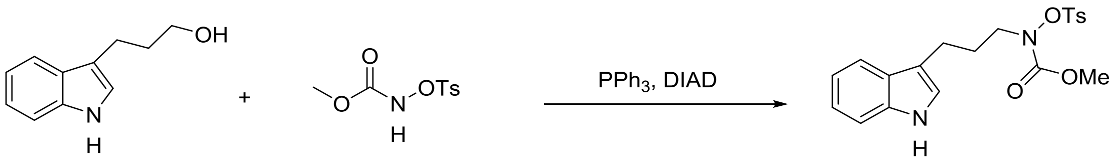

<u>**General procedure C**</u>**:** 3-(1*H*-Indol-3-yl)propan-1-ol (0.5 g, 2.85 mmol), methyl (tosyloxy) carbamate **2d'** (839 mg, 3.42 mmol), PPh_3 (1.12 g, 4.27 mmol), DIAD (0.84 mL, 4.27 mmol), THF (3.0 mL) and toluene (12.0 mL) were employed. Purification of the product by flash column chromatography (PhMe) afforded **3d'** (588 mg, 51%) as a light red viscous oil.

IR (film) ν_{max} / cm^{-1}: 3322 (br, w), 1723 (m), 1701 (m), 1448 (m), 1379 (m), 1191 (m), 1178 (s), 747 (s).

^{1}H NMR (400 MHz, CD_2Cl_2) δ 8.28 (s, 1H), 7.83 (d, *J* = 8.3 Hz, 2H), 7.58 (dt, *J* = 7.9, 1.0 Hz, 1H), 7.39 (dt, *J* = 8.1, 0.9 Hz, 1H), 7.30 (dd, *J* = 18.6, 7.8 Hz, 2H), 7.23 – 7.18 (m, 1H), 7.12 (ddd, *J* = 8.0, 7.0, 1.1 Hz, 1H), 7.00 (s, 1H), 3.76 – 3.56 (m, 2H), 3.50 (s, 3H), 2.76 (t, *J* = 7.5 Hz, 2H), 2.44 (s, 3H), 2.06 (p, *J* = 7.5 Hz, 2H).

^{13}C NMR (101 MHz, CD_2Cl_2) δ 157.76, 146.82, 142.48, 136.96, 131.43, 130.29, 130.19, 130.15, 129.87, 129.84, 129.65, 129.51, 128.71, 127.84, 126.62, 125.79, 122.24, 122.13, 119.48, 119.08, 115.22, 111.72, 54.14, 53.31, 26.77, 22.55, 21.98.

ESI-HRMS: m/z calculated for $C_{20}H_{22}N_2NaO_5S[M+Na]^+$: 425.1142; found: 425.1127.

***tert*-Butyl (3-(1*H*-indol-3-yl)propyl)((perfluorobenzoyl)oxy)carbamate (3e)**

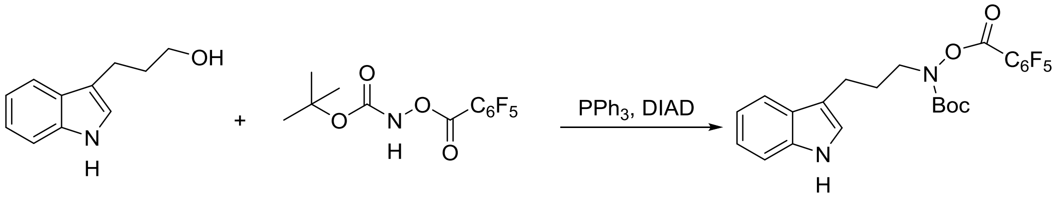

<u>**General procedure C:**</u> 3-(1*H*-Indol-3-yl)propan-1-ol (0.50 g, 2.86 mmol), *tert*-butyl (perfluorobenzoyl)oxycarbamate **2e** (1.21 g, 3.71 mmol), PPh_3 (1.12 g, 4.27 mmol), DIAD (0.84 mL, 4.27 mmol), THF (3.0 mL) and toluene (12.0 mL) were employed. Purification of

the product by flash column chromatography (PhMe) afforded **3e** (1.04 g, 75%) as a crystalline colorless solid.

m.p.: 102 - 104 ° C (EtOAc/Hexane).

IR (film) ν_{max} / cm^{-1}: 3412 (br, w), 1779 (m), 1710 (m), 1524 (m), 1501 (s), 1326 (m), 1151 (s), 739 (s).

^{1}H NMR (400 MHz, $CDCl_3$) δ 8.00 (s, 1H), 7.58 (d, *J* = 7.9 Hz, 1H), 7.35 (d, *J* = 8.1 Hz, 1H), 7.17 (dd, *J* = 11.1, 3.9 Hz, 1H), 7.13 – 7.06 (m, 1H), 7.01 (s, 1H), 3.77 (t, *J* = 7.0 Hz, 2H), 2.87 (t, *J* = 7.4 Hz, 2H), 2.11 – 2.00 (m, 2H), 1.48 (s, 9H).

^{13}C NMR (101 MHz, $CDCl_3$) δ 154.85, 136.49, 127.46, 122.08, 121.60, 119.31, 118.89, 111.21, 83.42, 50.69, 28.15, 27.33, 22.11.

The aromatic signals corresponding to the pentafluorobenzoyl group could not be resolved due to their weak intensity.

^{19}F NMR (377 MHz, $CDCl_3$) δ -136.37 (d, *J* = 18.2 Hz, 2F), -146.56 (tt, *J* = 20.9, 5.1 Hz, 1F), -158.80 – -159.98 (m, 2F).

ESI-HRMS: m/z calculated for $C_{23}H_{21}F_5N_2NaO_4$[M+Na]$^+$: 507.1314; found: 507.1318.

***tert*-Butyl (3-(1*H*-indol-3-yl)propyl)(tosyloxy)carbamate (3e')**

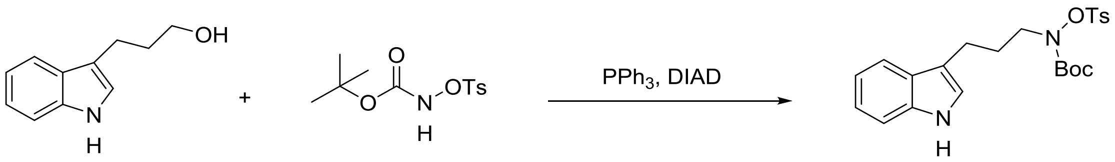

General procedure C: 3-(1*H*-Indol-3-yl)propan-1-ol (0.50 g, 2.86 mmol), BocNHOTs **2e'** (984 mg, 3.42 mmol), PPh_3 (1.12 g, 4.27 mmol), DIAD (0.84 mL, 4.27 mmol), THF (3.0 mL) and toluene (12.0 mL) were employed. Purification of the product by flash column chromatography (0–2%EtOAc/PhMe) afforded afforded **3e'** (0.81 g, 64%) as a colorless viscous oil.

IR (film) ν_{max} / cm^{-1}: 3675 (m), 2988 (s), 1719 (m), 1381 (m), 1066 (s), 1057 (s), 749 (s).

^{1}H NMR (400 MHz, CD_2Cl_2) δ 8.18 (s, 1H), 7.83 (d, *J* = 8.3 Hz, 2H), 7.64 – 7.49 (m, 1H), 7.37 (d, *J* = 8.2 Hz, 1H), 7.34 (d, *J* = 8.0 Hz, 2H), 7.23 – 7.14 (m, 1H), 7.10 (ddd, *J* = 8.2, 7.1, 1.2

Hz, 1H), 7.01 (s, 1H), 3.66 (s, 2H), 2.75 (t, *J* = 7.5 Hz, 2H), 2.44 (s, 3H), 2.14 – 1.93 (m, 2H), 1.22 (s, 9H).

^{13}C NMR (101 MHz, CD_2Cl_2) δ 156.00, 146.57, 136.97, 131.68, 130.13, 130.08, 127.92, 122.35, 122.00, 119.60, 119.18, 115.65, 111.65, 83.74, 53.42, 27.90, 26.73, 22.66, 21.97, 15.66.

ESI-HRMS: m/z calculated for $C_{23}H_{28}N_2NaO_5S$[M+Na]$^+$: 467.1611; found: 467.1614.

Allyl (3-(1*H*-indol-3-yl)propyl)(tosyloxy)carbamate 3f

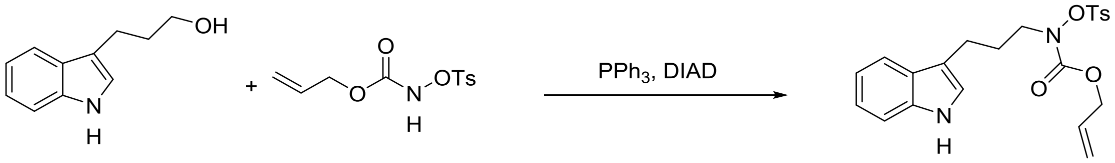

__General procedure D__**:** 3-(1*H*-Indol-3-yl)propan-1-ol (0.5 g, 2.85 mmol), allyl (tosyloxy) carbamate **2f'** (928 mg, 3.42 mmol), PPh_3 (1.12 g, 4.27 mmol), DIAD (0.84 mL, 4.27 mmol) and THF (20.0 mL) were employed. Purification of the product by flash column chromatography (1% EtOAc/PhMe) afforded **3f** (1.05 g, 86%) as a colorless foam.

IR (film) ν_{max} / cm^{-1}: 3417 (w), 2987 (m), 1726 (m), 1382 (m), 1191 (m), 1178 (s), 750 (s).

^{1}H NMR (400 MHz, $CDCl_3$) δ 7.84 – 7.72 (m, 2H), 7.53 (dt, *J* = 7.9, 1.0 Hz, 1H), 7.36 (dt, *J* = 8.1, 0.9 Hz, 1H), 7.31 (d, *J* = 8.1 Hz, 2H), 7.19 – 7.12 (m, 1H), 7.08 (ddd, *J* = 8.0, 7.0, 1.1 Hz, 1H), 7.00 (s, 1H), 5.65 (ddt, *J* = 16.7, 10.6, 5.7 Hz, 1H), 5.24 – 5.09 (m, 2H), 4.36 (d, *J* = 5.8 Hz, 2H), 3.64 (s, 2H), 2.87 – 2.64 (m, 2H), 2.43 (s, 3H), 2.03 (t, *J* = 7.5 Hz, 2H).

^{13}C NMR (101 MHz, $CDCl_3$) δ 156.99, 146.77, 136.94, 131.95, 131.48, 130.23, 129.91, 129.67, 127.88, 126.65, 122.36, 122.02, 119.59, 119.15, 118.98, 115.47, 111.63, 68.04, 53.38, 26.73, 22.57, 22.02.

ESI-HRMS: m/z calculated for $C_{22}H_{24}N_2NaO_5S$ [M+Na]$^+$: 451.1298; found: 451.1305.

Benzyl (3-(5-methoxy-1*H*-indol-3-yl)propyl)((perfluorobenzoyl)oxy)carbamate (3g)

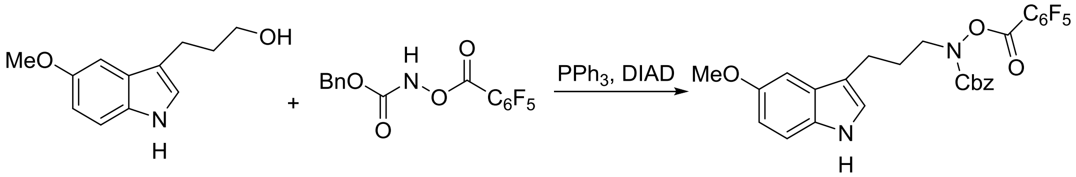

General procedure C: 3-(5-Methoxy-1*H*-indol-3-yl)propan-1-ol (0.585 g, 2.85 mmol), benzyl (perfluorobenzoyl)oxycarbamate **2c** (1.24 g, 3.42 mmol), PPh_3 (1.12 g, 4.27 mmol), DIAD (0.84 mL, 4.27 mmol), toluene (12.0 mL) and THF (3.0 mL) were employed. Purification of the product by flash column chromatography (0–1% EtOAc/PhMe) afforded **3g** (889 mg, 57%) as a crystalline colorless solid.

m.p.: 135 - 137 ° C (EtOAc/Hexane).

IR (film) ν_{max} / cm^{-1}: 3675 (br, w), 2988 (s), 1781 (m), 1722 (m), 1524 (m), 1499 (m), 1176 (m), 1066 (s), 750 (m).

^{1}H NMR (400 MHz, $CDCl_3$) δ 7.85 (s, 1H), 7.41 – 7.29 (m, 5H), 7.21 (d, *J* = 8.8 Hz, 1H), 7.00 (d, *J* = 2.2 Hz, 1H), 6.93 (d, *J* = 1.9 Hz, 1H), 6.84 (dd, *J* = 8.8, 2.4 Hz, 1H), 5.23 (s, 2H), 3.92 – 3.76 (m, 5H), 2.83 (t, *J* = 7.4 Hz, 2H), 2.13 – 1.95 (m, 2H).

^{13}C NMR (101 MHz, $CDCl_3$) δ 157.47, 155.65, 153.99, 135.33, 131.59, 128.70, 128.60, 128.26, 127.76, 122.52, 114.77, 112.20, 111.91, 100.68, 68.90, 55.97, 50.85, 27.10, 22.00.

The aromatic signals corresponding to the pentafluorobenzoyl group could not be resolved due to their weak intensity.

^{19}F NMR (377 MHz, $CDCl_3$) δ -135.87 (d, *J* = 18.7 Hz, 2F), -145.94 (tt, *J* = 21.0, 5.1 Hz, 1F), -159.28 (ddd, *J* = 26.8, 12.9, 6.2 Hz, 2F).

ESI-HRMS: m/z calculated for $C_{27}H_{21}F_5N_2NaO_5$ $[M+Na]^+$: 571.1263; found: 571.1258.

Benzyl (3-(5-methyl-1*H*-indol-3-yl)propyl)((perfluorobenzoyl)oxy)carbamate (3h)

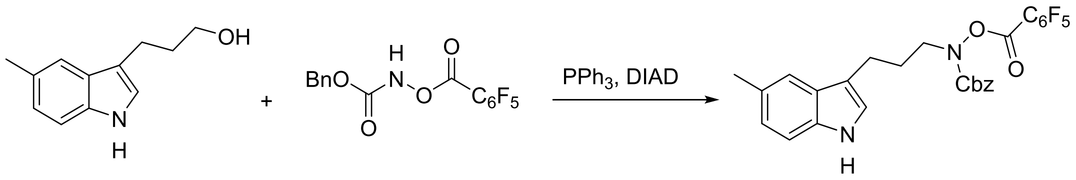

General procedure C: 3-(5-Methyl-1*H*-indol-3-yl)propan-1-ol (0.54 g, 2.85 mmol), benzyl (perfluorobenzoyl)oxycarbamate **2c** (1.24 g, 3.42 mmol), PPh_3 (1.12 g, 4.27 mmol), DIAD (0.84 mL, 4.27 mmol), toluene (12.0 mL) and THF (3.0 mL) were employed. Purification of the product by flash column chromatography (PhMe) afforded **3h** (810 mg, 53%) as a colorless solid.

m.p.: 143 - 145 °C (EtOAc/Hexane).

IR (film) ν_{max} / cm^{-1}: 3675 (m), 2988 (s), 1494 (m), 1260 (m), 1175 (s), 1066 (s), 1056 (s), 750 (s).

^{1}H NMR (400 MHz, $CDCl_3$) δ 7.82 (s, 1H), 7.37 (dd, *J* = 9.9, 5.7 Hz, 6H), 7.22 (d, *J* = 8.3 Hz, 1H), 7.01 (d, *J* = 8.1 Hz, 1H), 6.91 (s, 1H), 5.23 (s, 2H), 3.85 (t, *J* = 6.9 Hz, 2H), 2.84 (t, *J* = 7.4 Hz, 2H), 2.45 (s, 3H), 2.13 – 1.99 (m, 2H).

^{13}C NMR (101 MHz, $CDCl_3$) δ 155.65, 135.36, 134.79, 128.71, 128.60, 128.56, 128.28, 127.61, 123.67, 121.85, 118.48, 114.56, 110.87, 68.89, 50.89, 27.20, 22.03, 21.55.

The aromatic signals corresponding to the pentafluorobenzoyl group could not be resolved due to their weak intensity.

^{19}F NMR (377 MHz, $CDCl_3$) δ -135.98 (tt, *J* = 10.6, 5.8 Hz, 2F), -145.58 (ddt, *J* = 26.4, 21.1, 5.4 Hz, 1F), -158.97 – -159.15 (m, 2F).

ESI-HRMS: m/z calculated for $C_{27}H_{21}F_5N_2NaO_4[M+Na]^+$: 555.1314; found: 555.1319.

Benzyl (3-(5-fluoro-1*H*-indol-3-yl)propyl)((perfluorobenzoyl)oxy)carbamate (3i)

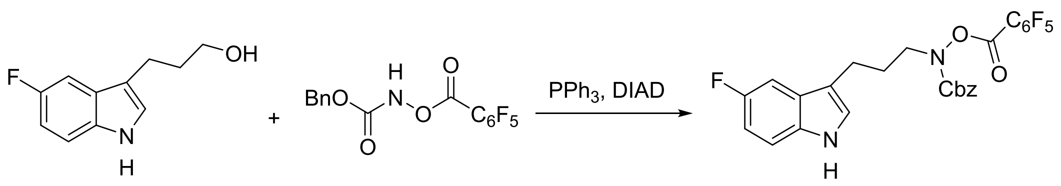

__General procedure C:__ 3-(5-Fluoro-1*H*-indol-3-yl)propan-1-ol (0.55 g, 2.85 mmol), benzyl (perfluorobenzoyl)oxycarbamate **2c** (1.24 g, 3.42 mmol), PPh_3 (1.12 g, 4.27 mmol), DIAD (0.84 mL, 4.27 mmol), toluene (12.0 mL) and THF (3.0 mL) were employed. Purification of the product by flash column chromatography (PhMe) afforded **3i** (874 mg, 55%) as a colorless foam.

IR (film) ν_{max} / cm^{-1}: 3430 (br, m), 2944 (br, m), 1781 (s), 1721 (s), 1524 (m), 1499 (s), 1327 (m), 1177 (s), 1092 (m), 1003 (s).

^{1}H NMR (400 MHz, $CDCl_3$) δ 7.93 (s, 1H), 7.40 – 7.29 (m, 6H), 7.25 – 7.20 (m, 1H), 7.17 (dt, *J* = 10.6, 5.3 Hz, 1H), 6.98 (t, *J* = 6.5 Hz, 1H), 6.92 (td, *J* = 9.0, 2.5 Hz, 1H), 5.22 (s, 2H), 3.82 (t, *J* = 6.9 Hz, 2H), 2.81 (t, *J* = 7.4 Hz, 2H), 2.04 (dq, *J* = 14.3, 7.1 Hz, 2H).

^{13}C NMR (101 MHz, $CDCl_3$) δ 155.61, 135.33, 132.95, 128.73, 128.65, 128.32, 123.55, 111.86, 111.76, 110.59, 110.33, 103.88, 103.64, 68.94, 50.76, 27.07, 21.92.

The aromatic signals corresponding to the pentafluorobenzoyl group could not be resolved due to their weak intensity.

^{19}F NMR (377 MHz, $CDCl_3$) δ -123.49 – -126.74 (m, 1F), -135.32 – -136.25 (m, 2F), -145.13 – -146.47 (m, 1F), -158.68 – -159.52 (m, 2F).

ESI-HRMS: m/z calculated for $C_{26}H_{18}F_6N_2NaO_4$ $[M+Na]^+$: 559.1063; found: 559.1058.

tert-Butyl (3-(5-fluoro-1*H*-indol-3-yl)propyl)(tosyloxy)carbamate (3j)

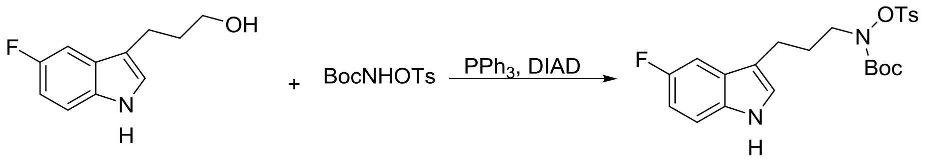

<u>**General procedure D:**</u> 3-(5-Fluoro-1*H*-indol-3-yl)propan-1-ol (0.55 g, 2.85 mmol), BocNHOTs **2e'** (984 mg, 3.42 mmol), PPh_3 (1.12 g, 4.27 mmol), DIAD (0.84 mL, 4.27 mmol) and THF (20 mL) were employed. Purification of the product by flash column chromatography (0–2% EtOAc/PhMe) afforded **3j** (986 mg, 75%) as a colorless solid.

m.p.: 84 - 86 ° C (EtOAc/Hexane).

IR (film) ν_{max} / cm^{-1}: 3418 (m), 2988 (m), 1689 (s), 1369 (m), 1156 (s), 750 (s).

^{1}H NMR (400 MHz, $CDCl_3$) δ 8.00 (s, 1H), 7.84 (d, *J* = 8.2 Hz, 2H), 7.31 (d, *J* = 7.9 Hz, 2H), 7.25 (q, *J* = 4.1 Hz, 1H), 7.17 (d, *J* = 9.3 Hz, 1H), 7.05 (s, 1H), 6.92 (td, *J* = 9.0, 2.4 Hz, 1H), 3.68 (s, 2H), 2.69 (t, *J* = 7.5 Hz, 2H), 2.44 (s, 3H), 2.04 (d, *J* = 8.6 Hz, 2H), 1.21 (s, 9H).

^{13}C NMR (101 MHz, $CDCl_3$) δ 158.96, 155.66, 145.83, 132.94, 131.34, 129.79, 129.65, 123.32, 115.58, 115.53, 111.85, 111.76, 110.55, 110.29, 103.89, 103.66, 83.40, 52.83, 27.72, 26.12, 22.22, 21.82.

^{19}F NMR (377 MHz, CD_2Cl_2) δ -116.62 (td, *J* = 8.6, 4.6 Hz).

ESI-HRMS: m/z calculated for $C_{23}H_{27}FN_2NaO_5S$$[M+H]^+$: 481.1517; found: 481.1511.

Benzyl (3-(5-chloro-1*H*-indol-3-yl)propyl)((perfluorobenzoyl)oxy)carbamate (3k)

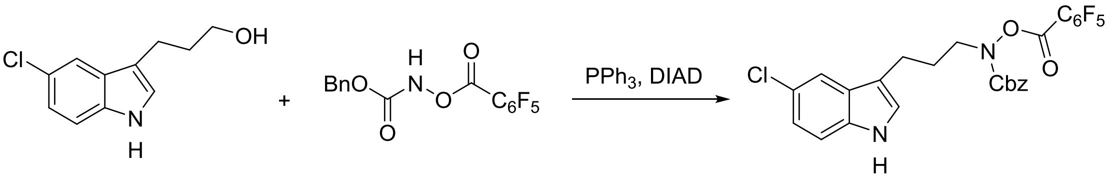

General procedure C: 3-(5-Chloro-1*H*-indol-3-yl)propan-1-ol (598 mg, 2.85 mmol), benzyl (perfluorobenzoyl)oxycarbamate **2c** (1.24 g, 3.42 mmol), PPh_3 (1.12 g, 4.27 mmol), DIAD (0.84 mL, 4.27 mmol), toluene (12.0 mL) and THF (3.0 mL) were employed. Purification of the product by flash column chromatography (PhMe) afforded **3k** (1.49 g, 94%) as a colorless solid.

m.p.: 96 - 97 °C (EtOAc/Hexane).

IR (film) ν_{max} / cm^{-1}: 3428 (br, w), 2941 (br, m), 1779 (m), 1720 (m), 1497 (s), 1326 (m), 1169 (s), 1077 (m), 1001 (s), 749 (m).

^{1}H NMR (400 MHz, $CDCl_3$) δ 7.96 (s, 1H), 7.50 (d, *J* = 2.0 Hz, 1H), 7.41 – 7.30 (m, 5H), 7.24 (dd, *J* = 8.6, 0.5 Hz, 1H), 7.11 (dd, *J* = 8.6, 2.0 Hz, 1H), 6.97 (d, *J* = 2.3 Hz, 1H), 5.23 (s, 2H), 3.82 (t, *J* = 6.9 Hz, 2H), 2.88 – 2.74 (m, 2H), 2.04 (p, *J* = 7.1 Hz, 2H).

^{13}C NMR (101 MHz, $CDCl_3$) δ 155.61, 135.33, 134.81, 128.73, 128.65, 128.53, 128.32, 125.15, 123.16, 122.39, 118.38, 114.92, 112.22, 68.96, 50.70, 27.10, 21.83.

The aromatic signals corresponding to the pentafluorobenzoyl group could not be resolved due to their weak intensity.

^{19}F NMR (377 MHz, $CDCl_3$) δ -135.99 (dq, *J* = 18.6, 6.5 Hz, 2F), -145.97 (tt, *J* = 20.9, 5.5 Hz, 1F), -157.94 – -160.41 (m, 2F).

ESI-HRMS: m/z calculated for $C_{26}H_{18}ClF_5N_2NaO_4$ $[M+Na]^+$: 575.0767; found: 575.0763.

Benzyl (3-(5-bromo-1*H*-indol-3-yl)propyl)((perfluorobenzoyl)oxy)carbamate (3l)

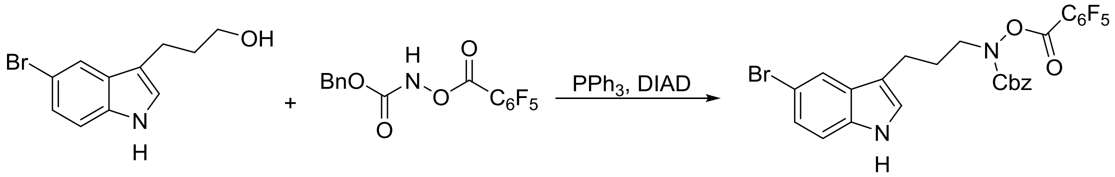

General procedure C: 3-(5-Bromo-1*H*-indol-3-yl)propan-1-ol (0.725 g, 2.85 mmol), benzyl (perfluorobenzoyl)oxycarbamate **2c** (1.24 g, 3.42 mmol), PPh_3 (1.12 g, 4.27 mmol), DIAD (0.84 mL, 4.27 mmol), toluene (12.0 mL) and THF (3.0 mL) were employed. Purification of the product by flash column chromatography (PhMe) afforded **3l** (1.35 g, 79%) as a light red solid.

m.p.: 93 - 94° C (EtOAc/Hexane).

IR (film) ν_{max} / cm^{-1}: 3428 (br, w), 2944 (br, w), 1781 (m), 1721 (m), 1524 (m), 1499 (s), 1327 (m), 1172 (s), 1003 (m).

^{1}H NMR (400 MHz, $CDCl_3$) δ 7.97 (s, 1H), 7.67 (d, *J* = 1.7 Hz, 1H), 7.41 – 7.30 (m, 6H), 7.24 (d, *J* = 1.7 Hz, 1H), 7.20 (d, *J* = 8.6 Hz, 1H), 6.95 (d, *J* = 2.1 Hz, 1H), 5.23 (s, 2H), 3.82 (t, *J* = 6.8 Hz, 2H), 2.81 (t, *J* = 7.4 Hz, 2H), 2.09 – 1.94 (m, 3H).

^{13}C NMR (101 MHz, $CDCl_3$) δ 155.61, 135.31, 135.07, 129.19, 128.73, 128.65, 128.32, 124.93, 123.00, 121.49, 114.83, 112.67, 112.64, 68.96, 50.66, 27.07, 21.81.

The aromatic signals corresponding to the pentafluorobenzoyl group could not be resolved due to their weak intensity.

^{19}F NMR (377 MHz, $CDCl_3$) δ -135.12 – -136.24 (m, 2F), -145.17 – -146.07 (m, 1F), -158.25 – -159.99 (m, 2F).

ESI-HRMS: m/z calculated for $C_{26}H_{18}F_5N_2NaO_4[M+Na]^+$: 619.0262; found: 619.0266.

tert-Butyl (3-(5-iodo-1*H*-indol-3-yl)propyl)(tosyloxy)carbamate (3m)

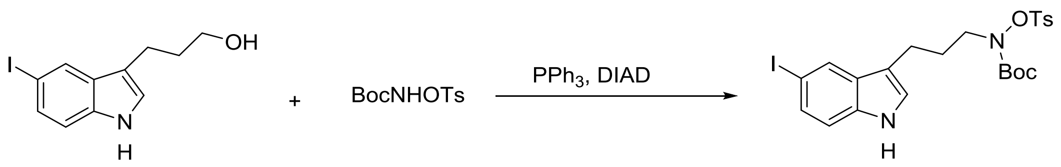

<u>General procedure D</u>: 3-(5-Iodo-1*H*-indol-3-yl)propan-1-ol (0.858 g, 2.85 mmol), BocNHOTs **2e'** (984 mg, 3.42 mmol), PPh_3 (1.12 g, 4.27 mmol), DIAD (0.84 mL, 4.27 mmol), and THF (20.0 mL) were employed. Purification of the product by flash column chromatography (PhMe) afforded **3m** (1.15 g, 71%) as a light yellow viscous oil.

IR (film) ν_{max} / cm^{-1}: 3420 (br, s), 1721 (s), 1456 (m), 1369 (m), 1192 (s), 1178 (s), 1155 (s), 748 (m).

^{1}H NMR (400 MHz, CD_2Cl_2) δ 8.30 (s, 1H), 7.89 (d, *J* = 1.7 Hz, 1H), 7.85 – 7.75 (m, 2H), 7.41 (dd, *J* = 8.5, 1.7 Hz, 1H), 7.34 (d, *J* = 8.0 Hz, 2H), 7.17 (d, *J* = 8.5 Hz, 1H), 6.99 (s, 1H), 3.63 (s, 2H), 2.79 – 2.56 (m, 2H), 2.43 (s, 3H), 2.00 (t, *J* = 7.5 Hz, 2H), 1.22 (s, 9H).

^{13}C NMR (101 MHz, CD_2Cl_2) δ 155.94, 146.60, 135.97, 131.64, 130.60, 130.58, 130.14, 130.08, 128.17, 123.02, 115.20, 113.74, 83.81, 82.90, 53.30, 27.91, 26.66, 22.45, 22.00.

ESI-HRMS: m/z calculated for $C_{23}H_{27}IN_2NaO_5S[M+Na]^+$: 593.0578; found: 593.0583.

Benzyl (3-(2-methyl-1*H*-indol-3-yl)propyl)((perfluorobenzoyl)oxy)carbamate (3n)

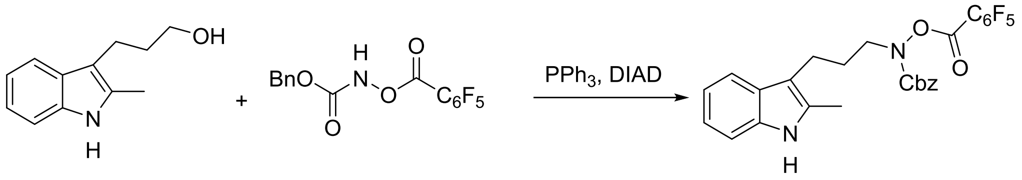

General procedure C: 3-(2-Methyl-1*H*-indol-3-yl)propan-1-ol (0.540 g, 2.85 mmol), benzyl (perfluorobenzoyl)oxycarbamate **2c** (1.24 g, 3.42 mmol), PPh_3 (1.12 g, 4.27 mmol), DIAD (0.84 mL, 4.27 mmol), toluene (12.0 mL) and THF (3.0 mL) were employed. Purification of the product by flash column chromatography (PhMe) afforded **3n** (787 mg, 52%) as a light red viscous oil.

IR (film) ν_{max} / cm^{-1}: 3365 (br, w), 2936 (br, w), 1781 (m), 1720 (m), 1524 (m), 1498 (s), 1326 (m), 1174 (s), 1002 (m), 742 (m).

^{1}H NMR (400 MHz, $CDCl_3$) δ 7.76 (s, 1H), 7.46 (d, *J* = 7.6 Hz, 1H), 7.40 – 7.30 (m, 5H), 7.24 (d, *J* = 7.6 Hz, 1H), 7.12 – 7.00 (m, 2H), 5.22 (s, 2H), 3.79 (t, *J* = 7.0 Hz, 2H), 2.80 (t, *J* = 7.5 Hz, 2H), 2.32 (s, 3H), 2.04 – 1.93 (m, 2H), 1.28 (dd, *J* = 9.8, 4.4 Hz, 2H).

^{13}C NMR (101 MHz, $CDCl_3$) δ 155.78, 135.39, 135.33, 131.34, 129.16, 128.68, 128.57, 128.54, 128.35, 128.22, 121.02, 119.18, 117.97, 110.45, 110.28, 68.87, 50.96, 27.51, 21.06, 11.60.

The aromatic signals corresponding to the pentafluorobenzoyl group could not be resolved due to their weak intensity.

^{19}F NMR (377 MHz, $CDCl_3$) δ -135.81 (dd, *J* = 19.4, 5.1 Hz, 2F), -146.04 (tt, *J* = 20.9, 5.3 Hz, 1F), -158.56 – -159.66 (m, 2F).

ESI-HRMS: m/z calculated for $C_{27}H_{21}F_5N_2NaO_4$[M+Na]$^+$: 555.1314; found: 555.1333.

Benzyl (3-(5,7-dimethyl-1*H*-indol-3-yl)propyl)((perfluorobenzoyl)oxy)carbamate (3o)

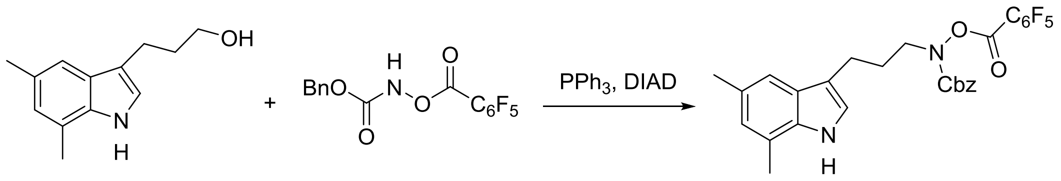

General procedure C: 3-(5,7-Dimethyl-1*H*-indol-3-yl)propan-1-ol (0.31 g, 1.52 mmol), benzyl (perfluorobenzoyl)oxycarbamate **2c** (0.66 g, 1.82 mmol), PPh_3 (0.44 g, 2.28 mmol), DIAD (0.33 mL, 2.28 mmol), toluene (6.0 mL) and THF (1.5 mL) were employed. Purification

of the product by flash column chromatography (17% Hexane/PhMe, then PhMe) afforded **3o** (682 mg, 82%) as a colorless solid.

m.p.: 130 - 132 ° C (EtOAc/Hexane).

IR (film) ν_{max} / cm^{-1}: 3413 (br, m), 2936 (br, m), 1781 (m, 1721 (m), 1524 (m), 1499 (s), 1325 (s), 1171 (s), 1002 (s).

^{1}H NMR (400 MHz, $CDCl_3$) δ 7.72 (s, 1H), 7.35 (q, *J* = 4.5 Hz, 5H), 7.19 (s, 1H), 6.92 (s, 1H), 6.82 (s, 1H), 5.22 (s, 2H), 3.83 (t, *J* = 6.9 Hz, 2H), 2.82 (t, *J* = 7.4 Hz, 2H), 2.43 (s, 3H), 2.41 (s, 3H), 2.06 (p, *J* = 7.1 Hz, 2H).

^{13}C NMR (101 MHz, $CDCl_3$) δ 155.64, 135.39, 134.39, 128.86, 128.71, 128.60, 128.30, 127.18, 124.39, 121.59, 120.06, 116.17, 115.09, 68.88, 50.86, 27.21, 22.17, 21.51, 16.64.

The aromatic signals corresponding to the pentafluorobenzoyl group could not be resolved due to their weak intensity.

^{19}F NMR (377 MHz, $CDCl_3$) δ -135.94 (dt, *J* = 20.3, 6.6 Hz, 2F), -146.16 (tt, *J* = 20.9, 5.4 Hz, 1F), -159.16 – -159.90 (m, 2F).

ESI-HRMS: m/z calculated for $C_{28}H_{23}F_5N_2NaO_4[M+Na]^+$: 569.1470; found: 569.1475.

Benzyl (3-(7-methyl-1*H*-indol-3-yl)propyl)((perfluorobenzoyl)oxy)carbamate (3p)

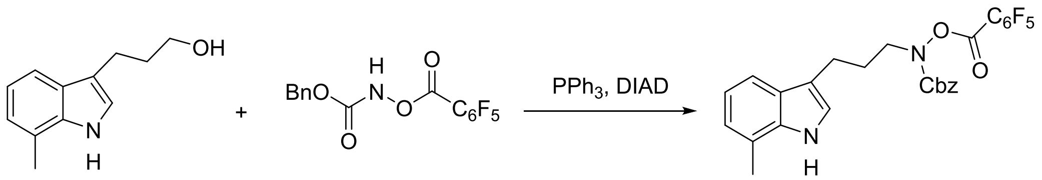

General procedure C: 3-(7-Methyl-1*H*-indol-3-yl)propan-1-ol (0.54 g, 2.85 mmol), benzyl (perfluorobenzoyl)oxycarbamate **2c** (1.24 g, 3.42 mmol), PPh_3 (1.12 g, 4.27 mmol), DIAD (0.84 mL, 4.27 mmol), toluene (12.0 mL) and THF (3.0 mL) were employed. Purification of the product by flash column chromatography (17% Hexane/PhMe, then PhMe only) afforded afforded **3p** (1.1 g, 73%) as a colorless solid.

m.p.: 97 - 99 ° C (EtOAc/Hexane).

IR (film) ν_{max} / cm^{-1}: 3414 (br, w), 1780 (m), 1721 (m), 1497 (s), 1325 (m), 1172 (s), 1000 (s), 745 (s).

^{1}H NMR (400 MHz, $CDCl_3$) δ 7.90 (s, 1H), 7.42 (d, *J* = 7.7 Hz, 1H), 7.39 – 7.36 (m, 5H), 7.06 – 6.96 (m, 2H), 6.95 (d, *J* = 1.8 Hz, 1H), 5.24 (s, 2H), 3.85 (t, *J* = 6.9 Hz, 2H), 2.87 (t, *J* = 7.3 Hz, 2H), 2.47 (s, 3H), 2.12 – 2.03 (m, 2H).

^{13}C NMR (101 MHz, $CDCl_3$) δ 155.64, 135.99, 135.35, 128.69, 128.62, 128.58, 128.28, 126.88, 122.56, 121.46, 120.40, 119.52, 116.51, 115.49, 68.88, 50.86, 27.20, 22.09, 16.62.

The aromatic signals corresponding to the pentafluorobenzoyl group could not be resolved due to their weak intensity.

^{19}F NMR (377 MHz, $CDCl_3$) δ -135.82 (d, *J* = 19.1 Hz, 2F), -145.97 (tt, *J* = 21.0, 5.2 Hz, 1F), -158.36 – -159.72 (m, 2F).

ESI-HRMS: m/z calculated for $C_{27}H_{21}F_5N_2NaO_4$[M+Na]$^+$: 555.1314; found: 555.1303.

***tert*-Butyl (4-(1*H*-indol-3-yl)butan-2-yl)(tosyloxy)carbamate (3r)**

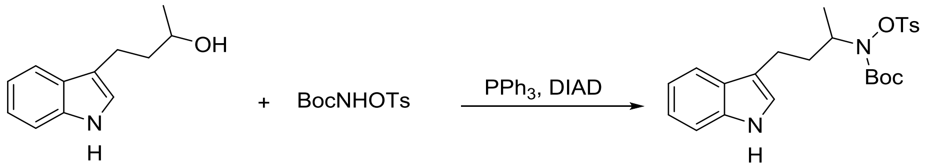

<u>**General procedure D**</u>**:** 4-(1*H*-Indol-3-yl)butan-2-ol (0.54 g, 2.85 mmol), BocNHOTs **2e'** (985 mg, 3.42 mmol), PPh_3 (823 mg, 3.14 mmol), DIAD (0.62 mL, 3.14 mmol) and THF (20.0 mL) were employed. Purification of the product by flash column chromatography (PhMe) afforded **3r** (0.94 g, 72%) as a light yellow viscous oil.

IR (film) ν_{max} / cm^{-1}: 2979 (m), 2970 (m), 1722 (m), 1370 (m), 1192 (m), 1178 (s), 1010 (m), 815 (s).

^{1}H NMR (400 MHz, CD_2Cl_2) δ 8.10 (s, 1H), 7.84 (d, *J* = 8.3 Hz, 2H), 7.56 (d, *J* = 7.9 Hz, 1H), 7.34 (dd, *J* = 9.7, 8.2 Hz, 3H), 7.16 (ddd, *J* = 8.1, 7.0, 1.2 Hz, 1H), 7.08 (ddd, *J* = 8.0, 7.0, 1.1 Hz, 1H), 7.00 (d, *J* = 2.2 Hz, 1H), 4.03 (h, *J* = 6.9 Hz, 1H), 2.79 (t, *J* = 7.8 Hz, 2H), 2.41 (s, 3H), 2.18 – 2.02 (m, 1H), 1.90 – 1.72 (m, 1H), 1.25 (s, 9H).

^{13}C NMR (101 MHz, CD_2Cl_2) δ 156.85, 146.43, 136.96, 132.22, 130.11, 130.07, 127.98, 122.31, 121.98, 119.56, 119.27, 116.15, 111.60, 83.88, 61.51, 34.86, 27.97, 22.69, 21.95, 17.64.

ESI-HRMS: m/z calculated for $C_{24}H_{30}N_2NaO_5S$[M+Na]$^+$: 481.1768; found: 481.1775.

tert-Butyl (R)- (3-(1H-indol-3-yl)butyl)(tosyloxy)carbamate (3s)

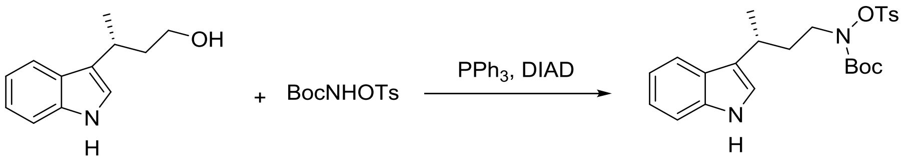

General procedure D: 3-(1*H*-Indol-3-yl)butan-1-ol **S11**(540 mg, 2.85 mmol), BocNHOTs **2e'** (985 mg, 3.42 mmol), PPh_3 (1.12 g, 4.27 mmol), DIAD (0.84 mL, 4.27 mmol) and THF (20.0 mL) were employed. Purification of the product by flash column chromatography (17% Hexane/PhMe, then PhMe only) afforded **3s** (829 mg, 63%) as a colorless viscous oil.

$[\alpha]_D^{22.3}$ = -35.8 (*c* = 1.20, $CHCl_3$)

IR (film) ν_{max} / cm^{-1}: 3675 (m), 2988 (s), 1712 (m), 1394 (m), 1259 (m),1166 (s), 1157 (s), 750 (s).

^{1}H NMR (400 MHz, CD_2Cl_2) δ 8.39 (s, 1H), 7.79 (dd, *J* = 8.3, 2.3 Hz, 2H), 7.61 (dd, *J* = 8.0, 2.1 Hz, 1H), 7.39 (dd, *J* = 8.2, 2.2 Hz, 1H), 7.35 – 7.26 (m, 2H), 7.22 – 7.15 (m, 1H), 7.10 (td, *J* = 7.5, 2.0 Hz, 1H), 6.99 (d, *J* = 2.2 Hz, 1H), 3.55 (s, 2H), 3.04 (qd, *J* = 7.0, 2.1 Hz, 1H), 2.43 (d, *J* = 2.3 Hz, 3H), 2.04 (d, *J* = 50.3 Hz, 2H), 1.37 (dd, *J* = 7.0, 2.3 Hz, 3H), 1.23 (s, 9H).

^{13}C NMR (101 MHz, CD_2Cl_2) δ 156.00, 146.51, 137.20, 131.63, 130.10, 130.01, 129.53, 127.13, 126.47, 122.25, 121.04, 120.92, 119.57, 119.47, 111.85, 83.75, 52.40, 33.56, 29.15, 27.91, 21.98, 21.95.

ESI-HRMS: m/z calculated for $C_{24}H_{30}N_2NaO_5S$ $[M+Na]^+$: 481.1768; found: 481.1764.

tert-Butyl (3-(1H-indol-3-yl)-4-methylpentyl)(tosyloxy)carbamate (3t)

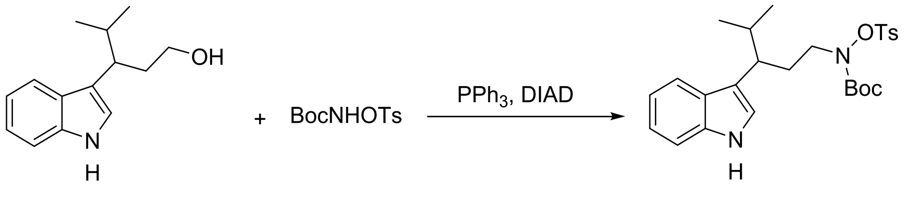

General procedure D: 3-(1*H*-Indol-3-yl)-4-methylpentan-1-ol (0.619 g, 2.85 mmol), BocNHOTs **2e'** (985 mg, 3.42 mmol), PPh_3 (1.12 g, 4.27 mmol), DIAD (0.84 mL, 4.27 mmol) and THF (20.0 mL) were employed. Purification of the product by flash column chromatography (0–1% EtOAc/PhMe) afforded **3t** (0.838 g, 60%) as a light red viscous oil.

IR (film) ν_{max} / cm^{-1}: 2959 (w), 1698 (m), 1368 (m), 1178 (s), 743 (s).

^{1}H NMR (400 MHz, CD_2Cl_2) δ 8.31 (s, 1H), 7.73 (dd, *J* = 28.9, 7.8 Hz, 2H), 7.55 (t, *J* = 7.9 Hz, 1H), 7.39 (d, *J* = 8.2 Hz, 1H), 7.23 (t, *J* = 9.3 Hz, 2H), 7.16 (t, *J* = 7.7 Hz, 1H), 7.07 (t, *J* = 7.7 Hz, 1H), 6.98 (s, 1H), 3.52 – 3.20 (m, 2H), 2.63 (dt, *J* = 11.0, 5.5 Hz, 1H), 2.38 (d, *J* = 5.3 Hz, 3H), 1.98 (dd, *J* = 12.9, 6.3 Hz, 3H), 1.20 (s, 9H), 0.94 – 0.82 (m, 6H).

^{13}C NMR (101 MHz, CD_2Cl_2) δ 155.92, 146.42, 137.02, 131.62, 130.03, 129.97, 129.46, 128.21, 126.41, 122.33, 122.17, 119.91, 119.48, 117.61, 111.67, 83.63, 53.06, 41.52, 33.54, 28.63, 27.91, 21.93, 20.90, 20.67.

ESI-HRMS: m/z calculated for $C_{26}H_{34}N_2O_5S[M+Na]^+$: 509.2081; found: 509.2090.

***tert*-Butyl (3-(1*H*-indol-3-yl)-3-phenylpropyl)(tosyloxy)carbamate (3u)**

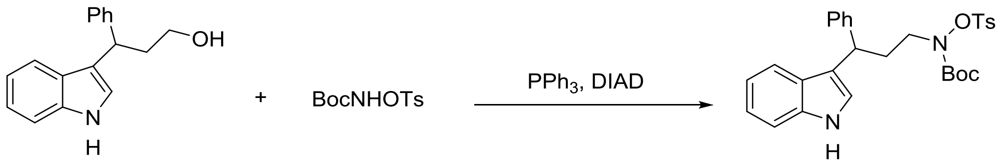

General procedure D: 3-(1*H*-Indol-3-yl)-3-phenylpropan-1-ol (0.72 g, 2.85 mmol), BocNHOTs **2e’** (985 mg, 3.42 mmol), PPh_3 (1.12 g, 4.27 mmol), DIAD (0.84 mL, 4.27 mmol) and THF (20.0 mL) were employed. Purification of the product by flash column chromatography (20% Hexane/PhMe, then 1% EtOAc/PhMe) afforded **3u** (1.01 g, 68%) as a light yellow form.

IR (film) ν_{max} / cm^{-1}: 3416 (m), 2980 (m), 1722 (m), 1370 (s), 1179 (s), 745 (s).

^{1}H NMR (400 MHz, $CDCl_3$) δ 7.95 (s, 1H), 7.74 – 7.66 (m, 2H), 7.31 (dt, *J* = 8.0, 1.0 Hz, 1H), 7.25 (dt, *J* = 8.1, 0.9 Hz, 1H), 7.22 – 7.14 (m, 5H), 7.13 – 7.03 (m, 3H), 7.01 (d, *J* = 1.0 Hz, 1H), 6.93 (ddd, *J* = 8.0, 7.1, 1.0 Hz, 1H), 4.07 (td, *J* = 7.7, 1.0 Hz, 1H), 3.52 (d, *J* = 46.2 Hz, 2H), 2.44 (s, 1H), 2.33 (s, 3H), 2.26 – 2.17 (m, 1H), 1.14 (s, 9H).

^{13}C NMR (101 MHz, CD_2Cl_2) δ 155.32, 145.96, 144.49, 136.55, 131.00, 129.55, 129.44, 128.35, 127.63, 126.69, 126.20, 121.93, 121.13, 119.12, 119.05, 111.11, 83.30, 52.04, 40.29, 31.68, 27.35, 21.38.

ESI-HRMS: m/z calculated for $C_{29}H_{32}N_2O_5S[M+H]^+$: 543.1924; found: 543.1947.

1'-Tosylspiro[indole-3,2'-pyrrolidine] (4a)

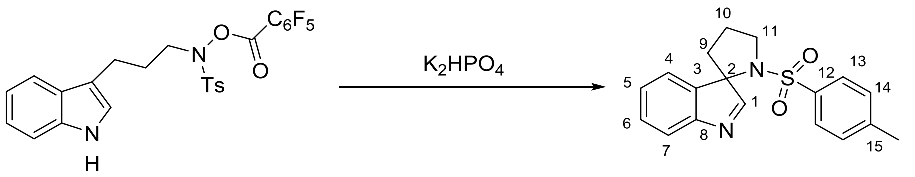

General procedure F: *N*-(3-(1*H*-Indol-3-yl)propyl)-4-methyl-*N*-((perfluorobenzoyl) oxy) benzenesulfonamide **3a** (80.8 mg, 0.15 mmol) and K_2HPO_4 (4 mg, 0.025 mmol) were employed. After stirring at 140 °C for 48 h, purification of the product by flash column chromatography (29%–50% EtOAc/Hexane) afforded **4a** (21.5 mg, 44%) as a light red foam.

IR (film) ν_{max} / cm^{-1}: 2975 (br, w), 1597 (m), 1566 (m), 1342 (m), 1158 (s), 1007 (m).

^{1}H NMR (400 MHz, CD_2Cl_2) δ 8.02 (s, 1H, C**1**-H), 7.53 (d, *J* = 7.7 Hz, 1H, C**4**-H), 7.29 (td, *J* = 7.6, 1.2 Hz, 1H, C**5**-H), 7.23 (d, *J* = 8.3 Hz, 2H, TsArCH × 2), 7.11 (d, *J* = 8.0 Hz, 2H, TsArCH × 2), 6.95 (td, *J* = 7.5, 1.0 Hz, 1H, C**6**-H), 6.73 (d, *J* = 7.4 Hz, 1H, C**7**-H), 3.86 (dt, *J* = 9.4, 7.3 Hz, 1H, C**11**-H), 3.74 (ddd, *J* = 9.4, 7.4, 5.4 Hz, 1H, C**11**-H’), 2.43 (dt, *J* = 8.3, 3.9 Hz, 1H, C**9**-H), 2.38 (s, 3H, CH_3), 2.30 – 2.15 (m, 2H, C**9**-H’, C**10**-H), 1.85 (ddd, *J* = 12.0, 6.4, 5.0 Hz, 1H, C**10**-H’).

^{13}C NMR (101 MHz, CD_2Cl_2) δ 174.76 (C**1**), 154.39 (C**15**), 144.01 (C**8**), 138.51(C**12**), 136.40 (C**3**), 129.80 (C**14**), 129.49 (C**5**), 127.69 (C**13**), 126.75 (C**6**), 122.61 (C**7**), 121.94 (C**4**), 77.72 (C**2**), 50.07 (C**11**), 35.92 (C**10**), 24.44 (C**9**), 21.75 (CH_3).

ESI-HRMS: m/z calculated for $C_{18}H_{18}N_2NaO_2S$ $[M+Na]^+$: 349.0981; found: 349.0983.

1'-((4-Methoxyphenyl)sulfonyl)spiro[indole-3,2'-pyrrolidine] (4a)

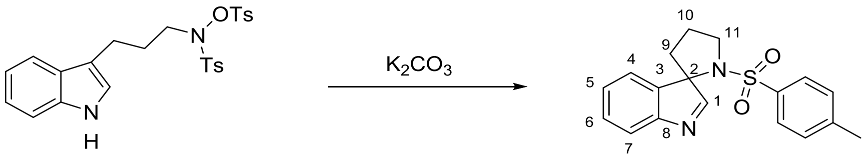

General procedure G: *N*-(3-(1*H*-indol-3-yl)propyl)-4-methyl-*N*-(tosyloxy) benzenesulfonamide (74.8 mg, 0.15 mmol) and K_2CO_3 (41.5 mg, 0.3 mmol) were employed. After stirring at 60 °C for 48 h, purification of the product by flash column chromatography (29–50 % EtOAc/Hexane) afforded **4a** (36.9 mg, 75%) as a light red viscous oil, whose spectral characteristics were concordant with values described above.

1'-((4-Methoxyphenyl)sulfonyl)spiro[indole-3,2'-pyrrolidine] (4b)

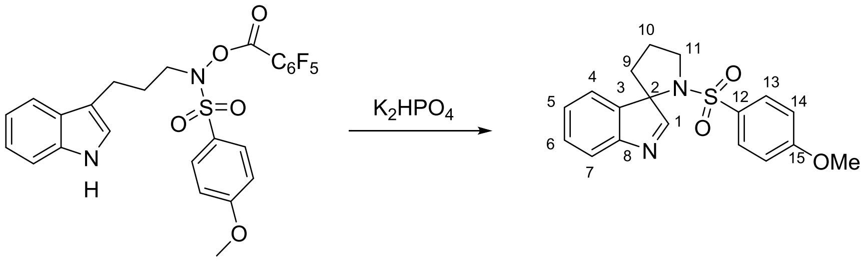

<u>**General procedure F**</u>**:** *N*-(3-(1*H*-Indol-3-yl)propyl)-4-methoxy-*N*-((perfluorobenzoyl) oxy) benzenesulfonamide **3b** (83.2 mg, 0.15 mmol) and K_2HPO_4 (4 mg, 0.025 mmol) were employed. After stirring at 140 °C for 48 h, purification of the product by flash column chromatography (33% EtOAc/Hexane) afforded **4b** (33.3 mg, 65%) as a light red oil.

IR (film) ν_{max} / cm^{-1}: 2988 (m), 1596 (m), 1260 (s), 1152 (s), 1076 (s), 750 (s).

^{1}H NMR (400 MHz, $CDCl_3$) δ 8.06 (s, 1H, C**1**-<u>H</u>), 7.55 (d, *J* = 7.6 Hz, 1H, C**4**-<u>H</u>), 7.32 – 7.24 (m, 3H, C**5**-<u>H</u>, C**13**-<u>H</u> × 2), 6.94 (t, *J* = 7.4 Hz, 1H, C**6**-<u>H</u>), 6.71 (d, *J* = 8.6 Hz, 3H, C**7**-<u>H</u>, C**14**-<u>H</u> × 2), 3.89 (dt, *J* = 15.6, 7.8 Hz, 1H, C**11**-<u>H</u>), 3.80 (s, 3H, **C<u>H</u>$_3$**O-<u>H</u>), 3.77 – 3.68 (m, 1H, C**11**-<u>H</u>’), 2.41 (dt, *J* = 12.4, 8.3 Hz, 1H, C**10**-<u>H</u>), 2.21 (td, *J* = 15.4, 7.9 Hz, 2H, C**10**-<u>H</u>’, C**9**-<u>H</u>), 1.92 – 1.74 (m, 1H, C**9**-<u>H</u>’).

^{13}C NMR (101 MHz, $CDCl_3$) δ 174.48 (C**1**), 162.86 (C**15**), 153.75 (C**8**), 137.78 (C**12**), 130.33 (C**3**), 129.48 (C**14**), 129.16 (C**5**), 126.38 (C**13**), 122.08 (C**6**), 121.81 (C**7**), 113.84 (C**4**), 55.67 (<u>C</u>H$_3$O), 49.46 (C**9**), 35.44 (C**10**), 23.89 (C**11**).

ESI-HRMS: m/z calculated for $C_{18}H_{19}N_2O_3S[M+H]^+$: 343.1111; found: 343.1125.

Benzyl spiro[indole-3,2'-pyrrolidine]-1'-carboxylate (4c)

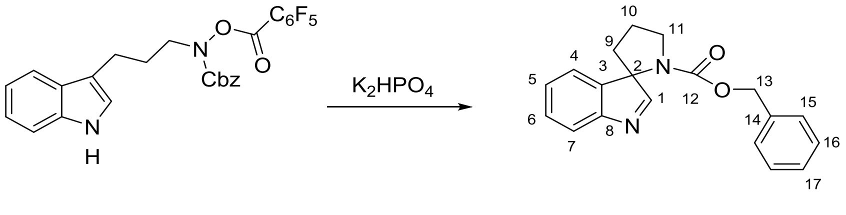

<u>**General procedure F**</u>**:** Benzyl (3-(1*H*-Indol-3-yl)propyl)((perfluorobenzoyl)oxy) carbamate **3c** (38.9 mg, 0.075 mmol) and K_2HPO_4 (2 mg, 0.011 mmol) were employed. After stirring at 140 °C for 48 h, purification of the product by flash column chromatography (29%–50% EtOAc/Hexane) afforded **4c** (18.9 mg, 82%, 1:2 mixture of rotamers *A:B*) as a light red oil.

IR (film) ν_{max} / cm^{-1}: 2972 (m), 1693 (s), 1407 (s), 1166 (s), 750 (s).

^{1}H NMR (400 MHz, $CDCl_3$) δ 7.99 (s, 0.33 H, C**1**-H, *A*), 7.93 (s, 0.62H, C**1**-H, *B*), 7.60 (d, *J* = 7.7 Hz, 0.37H, C**4**-H, *A*), 7.49 (d, *J* = 7.7 Hz, 0.74 H, C**4**-H, *B*), 7.37 – 7.03 (m, 6H, C**15**-H × 2, C**16**-H × 2, C**17**-H, C**6**-H), 6.68 (d, *J* = 7.2 Hz, 1H, C**7**-H), 5.06 (d, *J* = 12.3 Hz, 0.39H, C**13**-H, *A*), 4.96 (d, *J* = 12.3 Hz, 0.43H, C**13**-H, *B*), 4.86 (d, *J* = 12.5 Hz, 0.67H, C**13**-H, *A'*), 4.70 (d, *J* = 12.4 Hz, 0.66H, C**13**-H, *B'*), 3.97 – 3.86 (m, 1H, C**11**-H), 3.80 (dt, *J* = 10.8, 7.1 Hz, 1H, C**11'**-H), 2.30 (ddp, *J* = 26.8, 20.6, 7.0 Hz, 2H, C**10**-H, C**9**-H), 2.14 (dt, *J* = 12.3, 5.8 Hz, 1H, C**10**-H'), 2.00 (dt, *J* = 11.8, 5.8 Hz, 1H, C**9**-H').

^{13}C NMR (101 MHz, $CDCl_3$) δ 174.48 (C**1**, *B*), 174.32 (C**1**, *A*), 153.86 (C**12**), 153.72 (C**8**), 140.85 (C**14**), 135.78 (C**3**), [129.10, 128.88, 128.56, 128.18, 127.66, 127.53, 126.87 (C**5**+C**7**+C**15**+C**16**+C**17**)], 121.70 (C**4**, *A*), 121.65 (C**4**, *B*), 120.90 (C**6**, *B*) , 120.82 (C**6**, *A*), 75.70 (C**2**, *A*+*B*), 67.08 (C**13**, *A*), 67.02 (C**13**, *B*), 48.73 (C**11**, *A*), 48.02 (C**11**, *B*), 35.46 (C**10**, *A*), 34.28 (C**10**, *B*), 24.51 (C**9**, *A*), 23.86 (C**9**, *B*).

ESI-HRMS: m/z calculated for $C_{19}H_{18}N_2NaO_2$[M+Na]$^+$: 329.1260; found: 329.1266.

Benzyl spiro[indole-3,2'-pyrrolidine]-1'-carboxylate (4c)

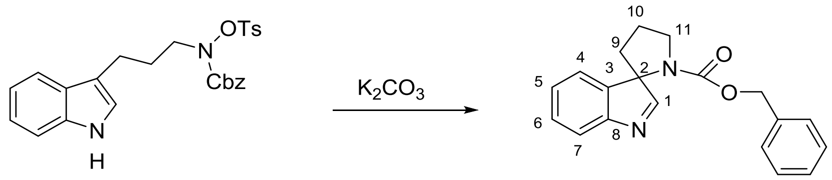

General procedure G: Benzyl (3-(1*H*-indol-3-yl)propyl)(tosyloxy)carbamate **3c'** (71.8 mg, 0.15 mmol) and K_2CO_3 (41.5 mg, 0.3 mmol) were employed. After stirring at 60 °C for 18 h, purification of the product by flash column chromatography (29%–50% EtOAc/Hexane) afforded **4c** (32.0 mg, 70%) as a light red viscous oil, whose spectral characteristics were concordant with values described above.

Methyl spiro[indole-3,2'-pyrrolidine]-1'-carboxylate (4d)

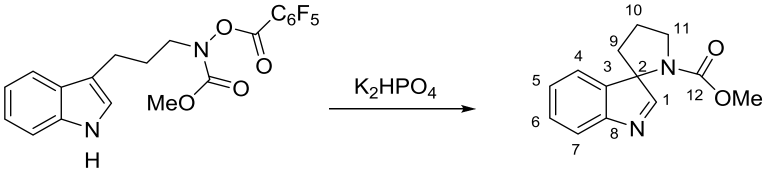

General procedure F: Methyl (3-(1*H*-indol-3-yl)propyl)((perfluorobenzoyl)oxy) carbamate **3d** (66.4 mg, 0.15 mmol) and K_2HPO_4 (4 mg, 0.025 mmol) were employed. After stirring at 140 °C for 48 h, purification of the product by flash column chromatography (33% EtOAc/Hexane) afforded **4d** (17 mg, 49%, 1:1 mixture of rotamers *A:B*) as a colorless viscous oil.

IR (film) ν_{max} / cm^{-1}:1691 (s), 1518 (m), 1451 (m), 1378 (m).

^{1}H NMR (400 MHz, $CDCl_3$) δ 7.96 (s, 0.5 H, C**1**-H, *A*), 7.92 (s, 1H C**1**-H, *B*), 7.56 (dd, *J* = 13.2, 7.6 Hz, 1H, C**4**-H, *A+B*), 7.34 (td, *J* = 7.4, 1.6 Hz, 1H, C**5**-H, *A+B*), 7.29 – 7.17 (m, 2H, C**6**-H, C**7**-H, *A+B*), 3.96 – 3.70 (m, 2H, C**11**-H, *A+B*), 3.59 (s, 1.5 H, CH_3O, *A*), 3.30 (s, 1.5H, CH_3O, *B*), 2.42 – 2.21 (m, 2H, C**10**-H, *A+B,* C**9**-H, *A+B*), 2.20 – 2.06 (m, 1H, C**10**-H', *A+B*), 1.96 (dq, *J* = 9.0, 5.6, 4.8 Hz, 1H, C**9'**-H, *A+B*).

^{13}C NMR (101 MHz, $CDCl_3$) δ 174.57 (C**1**, *A*) , 174.34 (C**1**, *B*), 153.63 (C**8**), 153.46 (C**12**) 129.11 (C**5**,*A*), 128.92 (C**5**, *B*), 126.82 (C**7**, A), 126.78 (C**7**, B), 121.70 (C**4**, A), 121.46 (C**4**, B), 120.82 (C**6**), 77.12 (C**2**), 52.50 (CH_3O), 48.67 (C**11**, *A*), 47.93 (C**11**, *B*), 35.38 (C**10**, *A*), 34.28 (C**10**, *B*), 24.58 (C**9**, *A*), 23.80 (C**9**, *B*).

ESI-HRMS: m/z calculated for $C_{13}H_{14}N_2NaO_2$ $[M+Na]^+$: 253.0947; found: 253.0951.

Methyl spiro[indole-3,2'-pyrrolidine]-1'-carboxylate (4d)

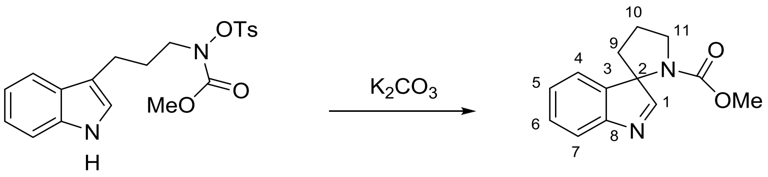

General procedure G: Methyl (3-(1*H*-indol-3-yl)propyl)(tosyloxy)carbamate **3d'** (60.4 mg, 0.15 mmol) and K_2CO_3 (41.5 mg, 0.3 mmol) were employed. After stirring at 60 °C for 48 h, purification of the product by flash column chromatography (29–50% EtOAc/Hexane) afforded **7ba** (25 mg, 72%) as a light red viscous oil, whose spectral characteristics were concordant with values described above.

***tert*-Butyl spiro[indole-3,2'-pyrrolidine]-1'-carboxylate (4e)**

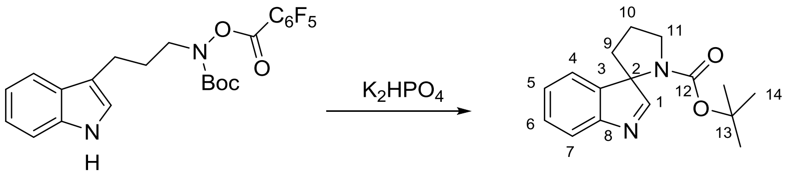

General procedure F: *tert*-Butyl (3-(1*H*-indol-3-yl)propyl)((perfluorobenzoyl)oxy) carbamate **4e** (72.7 mg, 0.15 mmol) and K_2HPO_4 (4 mg, 0.025 mmol) were employed. After stirring at 140 °C for 48 h, purification of the product by flash column chromatography (33% EtOAc/Hexane) afforded **4e** (14.4 mg, 35%) as a colorless viscous oil.

IR (film) ν_{max} / cm^{-1}: 2973 (m), 1680 (s), 1387 (s), 1366 (s), 1153 (s), 738 (s).

^{1}H NMR (400 MHz, $CDCl_3$) δ 7.92 (s, 1H, C**1**-H), 7.50 (d, *J* = 7.6 Hz, 1H, C**4**-H), 7.32 (td, *J* = 7.4, 1.7 Hz, 1H, C**5**-H), 7.26 – 7.18 (m, 2H, C**6**-H, C**7**-H), 3.84 (dt, *J* = 10.7, 6.4 Hz, 1H, C**11**-H), 3.71 (dt, *J* = 10.7, 6.8 Hz, 1H, C**11**-H'), 2.35 – 2.24 (m, 1H, C**10**-H), 2.20 (dt, *J* = 13.5, 6.6 Hz, 1H, C**9**-H), 2.09 (dt, *J* = 12.5, 6.2 Hz, 1H, C**10**-H'), 2.00 (dt, *J* = 12.1, 6.1 Hz, 1H, C**9**-H'), 0.97 (s, 9H, *t*Bu, C**14**-H_3× 3).

^{13}C NMR (101 MHz, $CDCl_3$) δ 175.04 (C**1**), 153.94 (C**12**), 153.35 (C**8**), 141.55 (C**3**), 128.63 (C**5**), 126.66 (C**7**), 121.36 (C**4**), 120.72 (C**6**), 80.37 (C**2**),75.84 (C**13**), 47.96 (C**11**), 35.22 (C**9**), 27.70 (C**14**), 23.91 (C**10**).

ESI-HRMS: m/z calculated for $C_{16}H_{20}N_2NaO_2$[M+Na]$^+$: 295.1417; found: 295.1422.

***tert*-Butyl spiro[indole-3,2'-pyrrolidine]-1'-carboxylate (4e)**

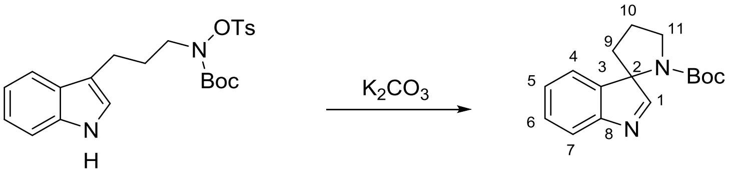

General procedure G: *tert*-Butyl (3-(1*H*-indol-3-yl)propyl)(tosyloxy)carbamate **3e'** (66.7 mg, 0.15 mmol) and K_2CO_3 (41.5 mg, 0.3 mmol) were employed. After stirring at 60 °C for 17 h, purification of the product by flash column chromatography (33% EtOAc/Hexane) afforded **4e** (29.5 mg, 72%) as a colorless viscous oil, whose spectral characteristics were concordant with values described above.

Allyl spiro[indole-3,2'-pyrrolidine]-1'-carboxylate (4f)

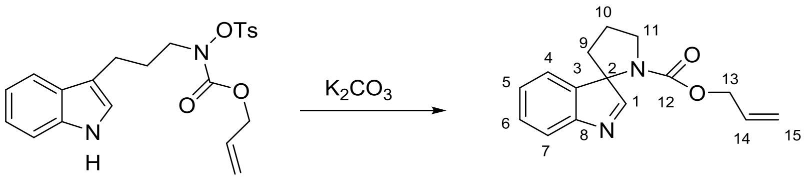

General procedure G: Allyl (3-(1*H*-indol-3-yl)propyl)(tosyloxy)carbamate **3f** (64.3 mg, 0.15 mmol) and K_2CO_3 (41.5 mg, 0.3 mmol) were employed. After stirring at 60 °C for 24 h, purification of the product by flash column chromatography (40% EtOAc/Hexane) afforded **4f** (30.4 mg, 79%, 1:1 mixture of rotamers *A*:*B*) as a colorless viscous oil.

IR (film) ν_{max} / cm^{-1}: 2974 (w), 2882 (w), 1695 (s), 1395 (s), 1332 (m), 1134 (m), 751 (s).

^{1}H NMR (500 MHz, CD_2Cl_2) δ 7.94 (s, 1H, C**1**-H, *A*+*B*), 7.51 (t, *J* = 8.6 Hz, 1H, C**4**-H, *A*+*B*), 7.35 (q, *J* = 5.5, 3.7 Hz, 1H, C**5**-H, *A*+*B*), 7.31 (d, *J* = 7.4 Hz, 1H, C**7**-H, *A*+*B*), 7.23 (t, *J* = 7.4 Hz, 1H, C**6**-H, *A*+*B*), 5.90 (ddt, *J* = 16.4, 10.8, 5.5 Hz, 0.5H, C**14**-H, *A*), 5.38 (ddt, *J* = 16.2, 10.4, 5.1 Hz, 0.5H, C**14**-H, *B*), 5.27 (d, *J* = 17.4 Hz, 0.5H, C**15**-H, *A*), 5.18 (d, *J* = 10.5 Hz, 0.5H, C**15**-H, *B*), 4.87 (d, *J* = 10.7 Hz, 0.5H, C**15**-H', *A*), 4.70 (d, *J* = 10.7 Hz, 0.5H, C**15**-H', *A*), 4.45 (qd, *J* = 13.6, 5.4 Hz, 1H, C**13**-H, *A*+*B*), 4.21 (qd, *J* = 14.0, 4.9 Hz, 1H, C**13**-H', *A*+*B*), 3.95 – 3.81 (m, 1H, C**11**-H, *A*+*B*), 3.75 (q, *J* = 7.7 Hz, 1H, C**11**-H', *A*+*B*), 2.29 (tq, *J* = 20.8, 7.8, 7.0 Hz, 2H, C**10**-H, *A*+*B*, C9-H, *A*+*B*), 2.14 (dq, *J* = 12.3, 5.6 Hz, 1H, C**10**-H', *A*+*B*), 1.95 (ddd, *J* = 29.6, 12.0, 6.0 Hz, 1H, C**9**-H', *A*+*B*).

^{13}C NMR (126 MHz, CD_2Cl_2) δ 175.12 (C**1**, *A*), 175.06 (C**1**, *B*), 154.71 (C**12**, *A*), 154.44 (C**12**, *B*), 154.02 (C**8**, *A*), 153.03 (C**8**, *B*), 141.60 (C**3**, *A*), 141.00 (C**3**, *B*), 133.62 (C**14**, *A*), 132.72 (C**14**, *B*), 129.26 (C**5**, *A*), 129.18 (C**5**, *B*), 127.10 (C**7**, *A*), 127.00 (C**7**, *B*), 121.69 (C**4**, *A*+*B*), 121.40 (C**6**, *A*+*B*), 117.46 (C**15**, *A*), 116.61 (C**15**, *B*), 76.64 (C**2**, *A*), 76.18 (C**2**, *B*), 66.34 (C**13**, *A*), 65.92 (C**13**, *B*), 49.15 (C**11**, *A*) , 48.41 (C**11**, *B*), 35.91 (C**9**, *A*), 34.71 (C**9**, *B*), 24.97 (C**10**, *A*), 24.23 (C**10**, *B*).

ESI-HRMS: m/z calculated for $C_{15}H_{17}N_2O_2[M+H]^+$: 257.1285; found: 257.1288.

Benzyl 5-methoxyspiro[indole-3,2'-pyrrolidine]-1'-carboxylate (4g)

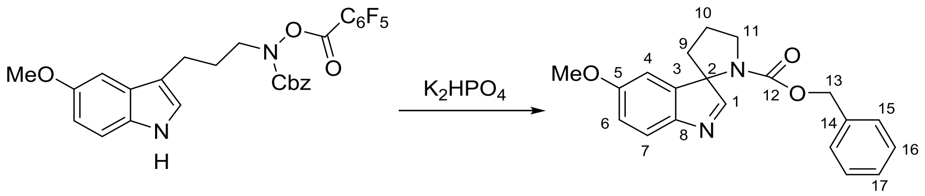

General procedure F: Benzyl (3-(5-methoxy-1*H*-indol-3-yl)propyl)((perfluorobenzoyl)oxy)carbamate **3g** (82.3 mg, 0.15 mmol) and K_2HPO_4 (4 mg, 0.025 mmol) were employed. After stirring at 140 °C for 24 h, purification of the product by flash column chromatography (33% EtOAc/Hexane) afforded **4g** (42.4 mg, 84%, 1:1 mixture of rotamers *A:B*) as a colorless viscous oil.

IR (film) ν_{max} / cm^{-1}: 2957 (br, m), 1695 (s), 1404 (s), 1351 (s), 1212 (m), 1026 (m), 731 (s).

^{1}H NMR (400 MHz, CD_2Cl_2) δ 7.82 (s, 1H, C**1**-H, *A*+*B*), 7.43 (d, *J* = 8.2 Hz, 0.5H, C**4**-H, *A*), 7.35 (q, *J* = 6.9, 5.7 Hz, 2.5H, C**4**-H, *B*; C**15**-H × 2), 7.17 (q, *J* = 7.3, 6.4 Hz, 2H, C**16**-H × 2,), 6.89 – 6.80 (m, 2H, C**17**-H, C**7**-H), 6.72 (d, *J* = 7.1 Hz, 1H, C**6**-H,), 5.04 (d, *J* = 12.6 Hz, 0.5H, C**13**-H, *A*), 4.96 (d, *J* = 12.4 Hz, 0.5H, C**13**-H', *A*), 4.89 (d, *J* = 12.6 Hz, 0.5H, C**13**-H, *B*), 4.67 (d, *J* = 12.6 Hz, 0.5H, C**13**-H', *B*), 3.88 – 3.72 (m, 5H, CH_3O, C**11**-H), 2.28 (dtt, *J* = 33.3, 14.1, 7.4 Hz, 2H, C**10**-H, C**9**-H), 2.11 (dd, *J* = 12.3, 6.2 Hz, 1H, C**10**-H'), 2.01 – 1.90 (m, 1H, C**9**-H').

^{13}C NMR (101 MHz, CD_2Cl_2) δ 173.01 (C**1**, *A*+*B*) , 159.72 (C**12**, *A*), 159.54 (C**12**, *B*), 154.08 (C**5**, ***A***), 153.18 (C**5**, ***B***) , 148.22 (C**8**, ***A***), 147.90 (C**8**, ***B***), 143.34 (C**14**, ***A***), 142.67 (C**14**, B), 137.31 (C**3**, ***A***), 136.76 (C**3**, ***B***), 128.94 (C**15**, ***A***), 128.52 (C**15**, ***B***), 128.49 (C**17**, ***A***), 128.42 (C**17**, ***B***), 128.01 (C**16**, ***A***), 127.81 (C**16**, ***B***), 122.14 (C**7**, ***A***), 121.93 (C**7**, ***B***), 113.45 (C**4**, ***A***), 113.04 (C**4**, *B*), 108.76 (C**6**, *A*), 108.28 (C**6**, *B*), 76.67 (C**2**, *A*), 76.20 (C**2**, *B*), 67.42 (C**13**, *A*), 67.11 (C**13**, *B*), 56.20 (CH_3**O**, *A* + *B*), 49.22 (C**11**, *A*), 48.49 (C**11**, *B*), 36.30 (C**10**, *A*), 35.01 (C**10**, *B*), 24.86 (C**9**, *A*), 24.18 (C**9**, *B*).

ESI-HRMS: m/z calculated for $C_{20}H_{21}N_2O_3$[M+H]$^+$: 337.1547; found: 337.1553.

Benzyl 5-methylspiro[indole-3,2'-pyrrolidine]-1'-carboxylate (4h)

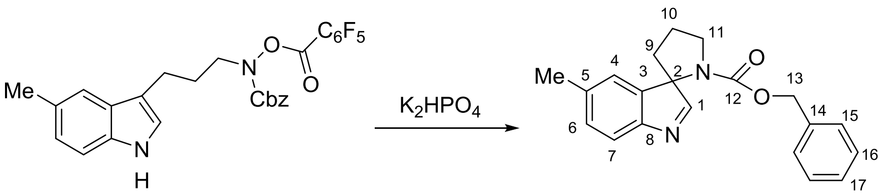

General procedure F: Benzyl (3-(5-methyl-1*H*-indol-3-yl)propyl) ((per fluorobenzoyl)oxy) carbamate **3h** (80.0 mg, 0.15 mmol) and K_2HPO_4 (4 mg, 0.025 mmol) were employed. After stirring at 140 °C for 24 h, purification of the product by flash column chromatography (33% EtOAc/Hexane) afforded **4h** (39.6 mg, 82%, 1:1 mixture of rotamers *A:B*) as a colorless viscous oil.

IR (film) ν_{max} / cm^{-1}: 2954(w), 1701 (s), 1408 (m), 1352 (m).

^{1}H NMR (400 MHz, CD_2Cl_2) δ 7.88 (d, *J* = 4.2 Hz, 1H, C**1**-H, *A*+*B*), 7.42 – 7.35 (m, 3H, C**4**-H, C**15**-H × 2), 7.28 – 7.05 (m, 4H, C**16**-H × 2, C**17**-H, C**7**-H), 6.68 (d, *J* = 6.9 Hz, 1H, C**6**-H), 5.05 (d, *J* = 12.5 Hz, 0.5H, C**13**-H, *A*), 4.96 (d, *J* = 12.4 Hz, 0.5H, C**13**-H', *A*), 4.89 (d, *J* = 12.7 Hz, 0.5H, C**13**-H, *B*), 4.66 (d, *J* = 12.7 Hz, 0.5H, C**13**-H', *B*), 3.88 (dd, *J* = 14.1, 8.6 Hz, 1H, C**11**-H, *A*), 3.83 – 3.68 (m, 1H, C**11**-H, *B*), 2.38 (t, *J* = 7.0 Hz, 3H, CH_3), 2.35 – 2.24 (m, 2H C**10**-H, C**9**-H, *A*+*B*), 2.17 – 1.89 (m, 2H, C**9**-H', C**9**-H', *A*+*B*).

^{13}C NMR (101 MHz, CD_2Cl_2) δ 174.13 (C**1**, *A*+*B*), 154.08 (C**12**, *A*), 153.18 (C**12** *B*), 152.55 (C**8, *A***), 152.29 (C**8, *B***), 141.80 (C**5,** *A*), 141.08 (C**5,** *B*), 137.35 (C1**4, *A***), 137.25 (C**14, *B***), 137.08 (C**3, *A***), 136.81 (C**3, *B***), 129.72 (C**16, *A***), 129.58 (C**16, *B***), 128.94 (C**4**, *A*), 128.52 (C**4**, *B*), 128.49 (C1**5, *A***), 128.42 (C1**5, *B***), 127.97 (C**17, *A***), 127.72 (C**17, *B***), 122.38 (C**7, *A* + *B***), 121.37 (C**6**, *A*), 121.21 (C**6**, *B*), 76.50 (C**2**, *A*), 76.01 (C**2**, *B*), 67.39 (C**13**, *A*), 67.02 (C**13**, *A*), 49.21 (C**11**, *A*), 48.47 (C**11**, *B*), 36.05 (C**10**, *A*) , 34.82 (C**10**, *B*), 24.91 (C**9**, *A*), 24.19 (C**9**, *B*), 21.71 (CH_3).

ESI-HRMS: m/z calculated for $C_{20}H_{20}N_2NaO_2$[M+Na]$^+$: 343.1417; found: 343.1425.

Benzyl 5-fluorospiro[indole-3,2'-pyrrolidine]-1'-carboxylate (4i)

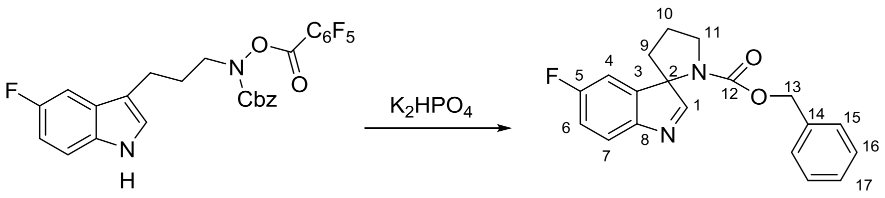

<u>General procedure F</u>: Benzyl (3-(5-fluoro-1*H*-indol-3-yl)propyl)((perfluorobenzoyl) oxy)carbamate **3i** (80.5 mg, 0.15 mmol) and K_2HPO_4 (4 mg, 0.025 mmol) were employed. After stirring at 140 °C for 48 h, purification of the product by flash column chromatography (33% EtOAc/Hexane) afforded **4i** (20.8 mg, 43%) as a colorless viscous oil.

IR (film) ν_{max} / cm^{-1}: 2972 (m), 1693 (s), 1487 (s),1404 (s), 1351 (s), 1128 (m), 733 (s).

^{1}H NMR (400 MHz, CD_2Cl_2, 1:1 mixture of rotamers *A:B*) δ 7.91 (d, *J* = 5.9 Hz, 1H, C**1**-<u>H</u>, *A*+*B*), 7.54 – 7.42 (m, 0.5H, C**4**-<u>H</u>, *A*), 7.42 – 7.30 (m, 2.5H, C**4**-<u>H</u>, *B*, C**15**-<u>H</u> × 2, *A*+*B*), 7.19 (t, *J* = 7.1 Hz, 2H, C**16**-<u>H</u> × 2, *A*+*B*), 7.06 – 6.98 (m, 2H, C**17**-<u>H</u>, C**6**-<u>H</u>, *A*+*B*), 6.73 (d, *J* = 6.7 Hz, 1H, C**7**-<u>H</u>, *A*+*B*), 5.04 (d, *J* = 12.5 Hz, 0.5H, C**13**-<u>H</u>, *A*), 4.96 (d, *J* = 12.5 Hz, 0.5H, C**13**-<u>H</u>, *B*), 4.86 (d, *J* = 12.4 Hz, 0.5H, C**13**-<u>H</u>’, *A*), 4.67 (d, *J* = 12.5 Hz, 0.5H, C**13**-<u>H</u>’, *B*), 3.94 – 3.81 (m, 1H, C**11**-<u>H</u>, *A*+*B*), 3.81 – 3.69 (m, 1H, C**11**-<u>H</u>’, *A*+*B*), 2.42 – 2.10 (m, 3H, C**10**-<u>H</u>, *A*+*B*; C**9**-<u>H</u>, *A*+*B*), 1.96 (ddd, *J* = 22.5, 12.0, 6.2 Hz, 1H, C**9**-<u>H</u>’, *A*+*B*).

^{13}C NMR (101 MHz, CD_2Cl_2) δ 175.01 (C**1**, *A*), 174.97 (C**1**, *B*), 163.76 (C**5**, *A*), 161.34 (C**5**, *B*), 153.93 (C**12**), 150.70 (C**8**, *A*), 150.41 (C**8**, *B*), 143.75 (C**3**), 137.21 (C**14**, *A*), 136.53 (C**14**, *B*), [128.98, 128.60, 128.56, 128.44, 128.18, , 127.95 (C**15**, C**16,** C**17,** *A*+*B*)], 122.54 (dd, *J* = 15.3, 9.1 Hz), C**7**, *A*+*B*) , 115.47 (dd, *J* = 23.8, 10.9 Hz, C**4**, *A*+*B*), 109.51 (dd, *J* = 25.5, 2.1 Hz, C**6**, *A*+*B*), 67.56 (C**13**, *A*), 67.32 (C**13**, *B*), 49.18 (C**11**, *A*), 48.48 (C**11**, *B*), 35.96 (C**10**, *A*), 34.71 (C**10**, *B*), 24.97 (C**9**, *A*), 24.25 (C**9**, *B*).

^{19}F NMR (377 MHz, $CDCl_3$) δ -125.00 (td, *J* = 9.5, 4.4 Hz).

ESI-HRMS: m/z calculated for $C_{19}H_{17}FN_2NaO_2$ $[M+Na]^+$: 347.1166; found: 347.1175.

tert-Butyl 5-fluorospiro[indole-3,2'-pyrrolidine]-1'-carboxylate (4j)

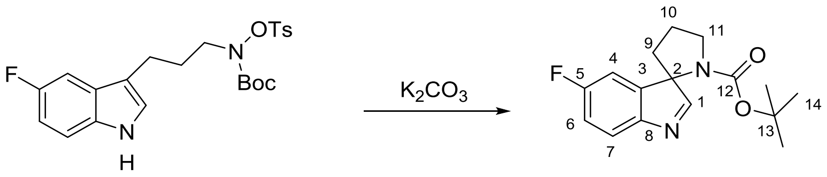

General procedure G: *tert*-Butyl (3-(5-fluoro-1*H*-indol-3-yl)propyl)(tosyloxy)carbamate **3j** (69.4 mg, 0.15 mmol) and K_2CO_3 (41.5 mg, 0.3 mmol) were employed. After stirring at 60 °C for 24 h, purification of the product by flash column chromatography (33% EtOAc/Hexane) afforded **4j** (30.6 mg, 70%) as a colorless viscous oil.

IR (film) ν_{max} / cm^{-1}: 2976 (m), 1693 (m), 1464 (m), 1385 (s), 1151 (s).

^{1}H NMR (400 MHz, $CDCl_3$) δ 7.90 (s, 1H, C**1**-H), 7.45 (dd, *J* = 8.4, 4.6 Hz, 1H, C**7**-H), 7.07 – 6.92 (m, 2H, C**4**-H, C**6**-H), 3.83 (dt, *J* = 10.9, 6.5 Hz, 1H, C**11**-H), 3.71 (dt, *J* = 10.8, 6.9 Hz, 1H, C**11**-H'), 2.30 (dt, *J* = 12.4, 7.1 Hz, 1H, C**9**-H), 2.18 (dt, *J* = 13.8, 6.8 Hz, 1H, C**10**-H), 2.10 (dt, *J* = 11.4, 5.8 Hz, 1H, C**10**-H'), 2.00 (dt, *J* = 12.6, 6.3 Hz, 1H, C**9**-H'), 1.02 (s, 9H, *t*Bu, C**14**-$H_3 \times 3$).

^{13}C NMR (101 MHz, $CDCl_3$) δ 174.84 (d, *J* = 3.8 Hz, C**1**), 162.01 (d, *J* = 246.2 Hz, C**5**), 153.02 (C**12**), 149.72 (d, *J* = 2.1 Hz, C**8**), 143.63 (d, *J* = 8.7 Hz, C**3**), 121.97 (d, *J* = 8.7 Hz, C**7**), 114.87 (d, *J* = 23.7 Hz, C**6**), 108.66 (d, *J* = 25.1 Hz, C**4**), 80.67 (C**13**), 76.09 (d, *J* = 1.8 Hz, C**2**), 48.00 (C**11**), 35.31 (C**10**), 27.77 (C**9**), 23.91 (C**14**).

ESI-HRMS: m/z calculated for $C_{17}H_{19}F_3N_2NaO_2$[M+Na]$^+$: 363.1291; found: 363.1300.

Benzyl 5-chlorospiro[indole-3,2'-pyrrolidine]-1'-carboxylate (4k)

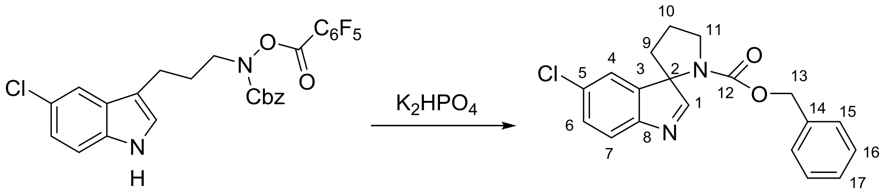

General procedure F: Benzyl (3-(5-chloro-1*H*-indol-3-yl)propyl) ((perfluorobenzoyl)oxy) carbamate **3k** (83.0 mg, 0.15 mmol) and K_2HPO_4 (4 mg, 0.025 mmol) were employed. After stirring at 140 °C for 48 h, purification of the product by flash column chromatography (33%

EtOAc/Hexane) afforded **4k** (33.7 mg, 66%, 1:2 mixture of rotamers *A:B*) as a colorless viscous oil.

IR (film) ν_{max} / cm^{-1}: 2953 (w), 2882 (w), 1689 (s), 1498 (m), 1482 (m), 1407 (s), 1353 (m).

^{1}H NMR (400 MHz, $CDCl_3$) δ 7.97 (s, 0.34H, C**1**-H, *A*), 7.91 (s, 0.60H, C**1**-H, *B*), 7.51 (d, *J* = 8.2 Hz, 0.35H, C**7**-H, *A*), 7.32 (ddd, *J* = 17.6, 14.2, 5.1 Hz, 3.6H, C**7**-H, *B*; C**15**-H × 2, *A*+*B*; C**17**-H, *A*+*B*), 7.25 – 7.13 (m, 3H, C**16**-H × 2, *A*+*B*; C**4**-H, *A*+*B*), 6.73 (dd, *J* = 7.1, 2.1 Hz, 1H, C**6**-H, *A*+*B*), 5.07 (d, *J* = 12.3 Hz, 0.38H, C**13**-H, *A*), 4.97 (d, *J* = 12.3 Hz, 0.40H, C**13**-H', *A*), 4.91 (d, *J* = 12.3 Hz, 0.63H, C**13**-H, *B*), 4.67 (d, *J* = 12.3 Hz, 0.61H, C**13**-H', *B*), 3.96 – 3.83 (m, 1H, C**11**-H, *A*+*B*), 3.78 (dt, *J* = 10.9, 7.2 Hz, 1H, C**11**-H', *A*+*B*), 2.39 – 2.28 (m, 1H, C**10**-H, *A*+*B*), 2.18 (ddp, *J* = 31.1, 12.6, 6.6 Hz, 2H, C**9**-H_2, *A*+*B*), 1.99 (qd, *J* = 15.4, 13.4, 5.7 Hz, 1H, C**10**-H', *A*+*B*).

^{13}C NMR (101 MHz, $CDCl_3$) δ 174.80 (C**1**, *B*), 174.69 (C**1**, *A*), 153.63 (C**12,** *A*+*B*), 152.22 (C**8**, *A*+*B*), 142.74 (C**3**, *B*), 141.93 (C**3**, *A*), 136.34 (C**14**, *A*) , 135.58 (C**14**, *B*), 132.78 (C**5**, *B*), 132.71 (C**5**, *A*), [129.19, 128.94, 128.61, 128.29, 128.18, 127.92, 127.76, 122.55, 122.43, 121.59 (C**4**, C**15**, C**16**, C**17**, *A*+*B*)], 76.36 (C**13**, *A*), 75.84 (C**13**, *B*), 67.36 (C**2**, *A*), 67.22 (C**2**, *B*), 48.68 (C**11**, *B*), 48.01 (C**11**, *A*), 35.32 (C**10**, *B*), 34.18 (C**10**, *A*), 24.56 (C**9**, *A*), 23.90 (C**9**, *B*).

ESI-HRMS: m/z calculated for $C_{19}H_{18}ClN_2O_2$ $[M+H]^+$: 341.1051; found: 341.1062.

Benzyl 5-bromospiro[indole-3,2'-pyrrolidine]-1'-carboxylate (4l)

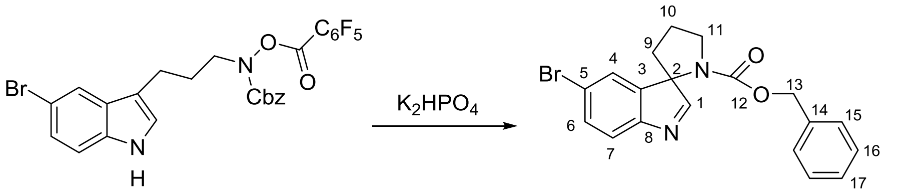

__General procedure F__: Benzyl (3-(5-bromo-1*H*-indol-3-yl)propyl)((perfluorobenzoyl) oxy)carbamate **3l** (90.0 mg, 0.15 mmol) and K_2HPO_4 (52.2 mg, 0.30 mmol) were employed. After stirring at 140 °C for 48 h, purification of the product by flash column chromatography (33% EtOAc/Hexane) afforded **4l** (32.5 mg, 56%, 1:2 mixture of rotamers *A:B*) as a colorless viscous oil.

IR (film) ν_{max} / cm^{-1}: 2925 (m), 1701 (s), 1407 (s), 1353 (s).

^{1}H NMR (400 MHz, CD_2Cl_2) δ 7.95 (s, 0.33H, C**1**-H, *A*), 7.90 (s, 0.56H, C**1**-H, *B*), 7.67 – 7.21 (m, 5H, C**4**-H, *A*+*B*; C**7**-H, *A*+*B*; C**15**-H × 2, *A*+*B*; C**17**-H, *A*+*B*), 7.25 – 7.10 (m, 2H, C**16**-H, *A*+*B*), 6.73 (dd, *J* = 6.8, 2.3 Hz, 1H, C**6**-H, *A*+*B*), 5.07 (d, *J* = 12.4 Hz, 0.36H, C**13**-H, *A*), 4.97 (d, *J* = 12.3 Hz, 0.38H, C**13**-H', *A*), 4.91 (d, *J* = 12.3 Hz, 0.57H, C**13**-H, *B*), 4.66 (d, *J* = 12.3 Hz, 0.56H, C**13**-H, *B*), 3.89 (dtd, *J* = 17.3, 11.9, 9.6, 5.8 Hz, 1H, C**11**-H, *A*+*B*), 3.78 (dt, *J* = 10.7, 7.0 Hz, 1H, C**11**-H', *A*+*B*), 2.34 (dq, *J* = 16.8, 6.0, 4.5 Hz, 1H, C**10**-H, *A*+*B*), 2.26 – 2.10 (m, 2H, C**9**-H_2, *A*+*B*), 1.99 (dq, *J* = 16.3, 10.7, 8.4 Hz, 1H, C**10**-H', *A*+*B*).

^{13}C NMR (101 MHz, CD_2Cl_2) δ 174.76 (C**1**, *B*), 174.68 (C**1**, *A*), 153.65 (C**12**, *A*+*B*), 152.70 (C**8**, *A*+*B*), 143.09 (C**3**, *A*+*B*), 135.60 (C**14**, *A*+*B*), 132.17 (C**4**, *A*), 131.94 (C**4**, *B*), 128.63, 128.34, 128.20 (C**5**, C**16**, *A*+*B*)], 127.95 (C**6**-H, *A*, 127.78 (C**6**-H, *B*), 124.44 (C**15**, *A*+*B*), 122.93 (C**17**, *A*+*B*), 120.74 (C**7**, *A*+*B*), 75.89 (C**13**, *A*+*B*), 67.41 (C**2**, *A*), 67.26 (C**2**, *B*), 48.71(C**11**, *B*), 48.03 (C**11**, *A*), 35.32 (C**10**, *B*), 34.19 (C**10**, *A*), 24.62 (C**9**, *A*), 23.94 (C**9**, *B*).

ESI-HRMS: m/z calculated for $C_{19}H_{18}BrN_2O_2$[M+H]$^+$: 385.0546; found: 385.0557.

tert-Butyl 5-iodospiro[indole-3,2'-pyrrolidine]-1'-carboxylate (4m)

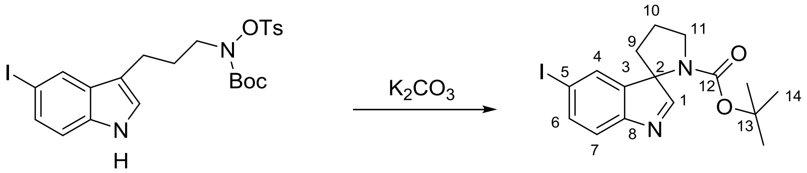

General procedure G: *tert*-Butyl (3-(5-iodo-1*H*-indol-3-yl)propyl)(tosyloxy)carbamate **3m** (85.6 mg, 0.15 mmol) and K_2CO_3 (41.5 mg, 0.3 mmol) were employed. After stirring at 60 °C for 18 h, purification of the product by flash column chromatography (29% EtOAc/Hexane) afforded **4m** (44.2 mg, 74%) as a colorless viscous oil.

IR (film) ν_{max} / cm^{-1}: 2975 (m), 1682 (s), 1503 (m), 1477 (m), 1389 (s), 1366 (s), 1159 (s).

^{1}H NMR (400 MHz, $CDCl_3$) δ 7.80 (s, 1H, C**1**-H), 7.61 (d, *J* = 8.1 Hz, 1H, C**7**-H), 7.52 (s, 1H, C**4**-H), 7.21 (d, *J* = 7.9 Hz, 1H, C**6**-H), 3.78 (dt, *J* = 12.5, 6.4 Hz, 1H, C**11**-H), 3.64 (dt, *J* = 13.6, 8.2 Hz, 1H, C**11**-H'), 2.24 (dt, *J* = 12.8, 7.1 Hz, 1H, C**9**-H), 2.13 (dt, *J* = 13.6, 6.8 Hz, 1H, C**10**-H), 2.04 (h, *J* = 5.7 Hz, 1H, C**10**-H'), 1.94 (dt, *J* = 12.5, 6.2 Hz, 1H, C**9**-H'), 0.96 (s, 9H, *t*Bu, C**14**-H_3 × 3).

^{13}C NMR (101 MHz, $CDCl_3$) δ 175.20 (C**1**), 153.63 (C**8**), 153.10 (C**12**), 144.03 (C**3**), 137.72 (C**7**), 130.04 (C**4**), 123.20 (C**6**), 91.54 (C**5**), 80.75 (C**13**), 75.94 (C**2**), 47.99 (C**11**), 35.10 (C**10**), 27.75 (C**14**), 23.93 (C**9**).

ESI-HRMS: m/z calculated for $C_{16}H_{19}IN_2NaO_2[M+Na]^+$: 421.0383; found: 421.0398.

Benzyl 2-methylspiro[indole-3,2'-pyrrolidine]-1'-carboxylate (4n)

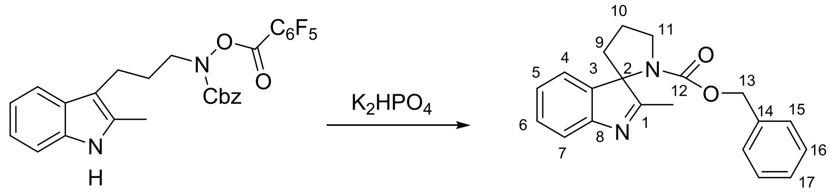

__General procedure F__**:** Benzyl (3-(2-methyl-1*H*-indol-3-yl)propyl)((perfluorobenzoyl)oxy) carbamate **3n** (80.0 mg, 0.15 mmol) and K_2HPO_4 (4.0 mg, 0.025 mmol) were employed. After stirring at 140 °C for 23 h, purification of the product by flash column chromatography (33% EtOAc/Hexane) afforded **4n** (30.2 mg, 63%, 1:1 mixture of rotamers *A:B*) as a colorless viscous oil.

IR (film) ν_{max} / cm^{-1}: 2974 (w), 1698 (s), 1589 (m), 1403 (s), 1350 (s), 1126 (m), 769 (m), 750 (s).

^{1}H NMR (400 MHz, CD_2Cl_2) δ 7.47 – 7.40 (m, 1H, C**5**-H), 7.41 – 7.31 (m, 3H, Ph-H_3), 7.28 (d, *J* = 7.5 Hz, 1H, C**6**-H), 7.25 – 7.06 (m, 3H, C**4**-H, Ph-H_2), 6.70 (dd, *J* = 7.5, 1.8 Hz, 1H, C**7**-H), 5.08 (d, *J* = 12.5 Hz, 0.5H, C**13**-H, *A*), 4.97 (d, *J* = 12.5 Hz, 0.5H, C**13**-H', *A*), 4.86 (d, *J* = 12.7 Hz, 0.5H, C**13**-H, *B*), 4.73 (d, *J* = 12.7 Hz, 0.5H, C**13**-H', *B*), 3.94 (dddd, *J* = 14.6, 10.9, 7.3, 3.4 Hz, 1H, C**11**-H, *A*+*B*), 3.77 (tdd, *J* = 10.6, 7.8, 2.6 Hz, 1H, C**11**-H', *A*+*B*), 2.33 – 2.15 (m, 6H, C**10**-H, *A*+*B*, C**9**-H_2, *A*+*B*, CH_3), 2.01 – 1.83 (m, 1H, C**10**-H', *A*+*B*).

^{13}C NMR (126 MHz, $CDCl_3$) δ 183.52 (C**1**, *A*), 183.05 (C**1**, *B*), 162.75 (C**12**, *A*+*B*) , 153.79 (C**8**, *A*), 153.33 (C**8**, *B*), 148.36 (C**3**, *A*+*B*), 135.89 (C**14**, *A*+*B*), [129.01, 128.80, 128.60, 128.26, 128.19, 128.06, (C1**5**, *A*+*B*); (C1**6**, *A*+*B*), C**17** (*A*+*B*)], 127.77 (C**7**, *A*), 127.67 (C**7**, *B*), 125.77 (C**6**, *A*), 125.68 (C**6**, *B*), 120.83 (C**5**, *A*), 120.72 (C**5**, *B*), 120.55 (C**4**, *A*), 120.44 (C**4**, *B*), 73.64 (C**2**, *A*+*B*), 67.16 (C**13**, *A*), 67.07 (C**13**, *B*), 48.83 (C**11**, *A*), 48.12 (C**11**, *B*), 37.13 (C**10**, *A*), 35.90 (C**10**, *B*), 24.07 (C**9**, *A*), 23.36 (C**9**, *B*), 15.44 (CH_3, *A*), 15.31 (CH_3, *B*).

ESI-HRMS: m/z calculated for $C_{20}H_{20}N_2NaO_2$ $[M+Na]^+$: 343.1417; found: 343.1428.

Benzyl 5,7-dimethylspiro[indole-3,2'-pyrrolidine]-1'-carboxylate (4o)

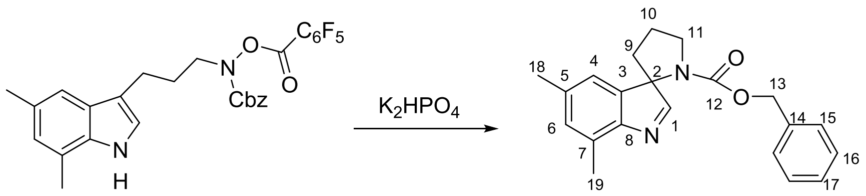

__General procedure F:__ Benzyl (3-(5,7-dimethyl-1*H*-indol-3-yl)propyl)((perfluorobenzoyl)oxy)carbamate **3o** (82.0 mg, 0.15 mmol) and K_2HPO_4 (4.0 mg, 0.025 mmol) were employed. After stirring at 140 °C for 20 h, purification of the product by flash column chromatography (33%–50% EtOAc/Hexane) afforded **4o** (41.8 mg, 83%, 3:7 mixture of rotamers *A:B*) as a colorless viscous oil.

IR (film) ν_{max} / cm^{-1}: 2951 (w), 2921 (w), 1698 (s), 1405 (s), 1351 (s),1148 (m), 1128 (m).

^{1}H NMR (400 MHz, $CDCl_3$) δ 7.89 (s, 0.3H, C**1**-H, *A*), 7.84 (s, 0.7H, C**1**-H, *B*), 7.34 – 7.30 (m, 1H, Ph-H, *A*+*B*), 7.19 – 7.10 (m, 2H, Ph-H_2, *A*+*B*), 6.96 (s, 0.3H, C**4**-H, *A*), 6.94 (s, 0.7H, C**4**-H, *B*), 6.89 (s, 0.3H, C**6**-H, *A*), 6.86 (s, 0.7H, C**6**-H, *B*), 6.67 (d, *J* = 7.1 Hz, 1H, Ph-H, *A*+*B*), 5.08 (d, *J* = 12.4 Hz, 0.3H, C**13**-H, *A*), 4.96 (d, *J* = 12.4 Hz, 0.3H, C**13**-H’, *A*), 4.90 (d, *J* = 12.5 Hz, 0.7H, C**13**-H’, *A*), 4.68 (d, *J* = 12.5 Hz, 0.7H, C**13**-H’, *B*), 3.95 – 3.82 (m, 1H, C**11**-H, *A*+*B*), 3.78 (dt, *J* = 10.9, 7.0 Hz, 1H, C**11**-H’, *A*+*B*), 2.53 (s, 0.9H, CH_3, C**18**-H_3, *A*), 2.42 (s, 2.1H, CH_3, C**18**-H_3, *B*), 2.33 (s, 0.9H, C**19**-H_3, *A*), 2.31 (s, 2.1H, C**19**-H_3, *B*), 2.29 – 2.19 (m, 2H, C**10**-H, *A*+*B*, C**9**-H, *A*+*B*), 2.10 (dt, *J* = 12.5, 6.1 Hz, 1H, C**9**-H’, *A*+*B*), 1.96 (ddd, *J* = 23.8, 11.8, 6.0 Hz, 1H, C**10**-H’, *A*+*B*).

^{13}C NMR (101 MHz, $CDCl_3$) δ 172.28 (C**1**, *B*), 172.12 (C**1**, *A*), 153.96 (C**12**, *B*), 152.75 (C**12**, *A*), 150.20 (C**5**, *A*), 149.89 (C**5**, *B*), 140.91 (C**3**, *B*), 140.05 (C**3**, *A*), 136.62 (C**8**, *B*) , 136.42 (C**8**, *A*), 135.76 (C**7**, *A*+*B*), 131.29 (C**4**, *A*),, 130.84 (C**4**, *B*), 130.65 (C**14**, *A*+*B*), [128.52, 128.08, 127.57, 127.41 (C1**5**, *A*+*B*); (C1**6**, *A*+*B*), C**17** (*A*+*B*)], 119.19 (C**6**, *A*+*B*), 76.16 (C**2**, *A*), 75.68 (C**2**, *B*), 67.06 (C**13**, *A*) 67.01 (C**13**, *B*), 48.70 (C**11**, *B*), 48.00 (C**11**, *A*), 35.61 (C**10**, *A*), 34.53 (C**10**, *B*), 24.43 (C**9**, *A*), 23.80 (C**9**, *B*), 21.53 (C**19**, *A*), 21.45 (C**19**, *B*), 16.57 (C**18**, *A*), 16.42 (C**18**, *B*).

ESI-HRMS: m/z calculated for $C_{21}H_{22}N_2NaO_2[M+Na]^+$: 357.1573; found: 357.1588.

Benzyl 7-methylspiro[indole-3,2'-pyrrolidine]-1'-carboxylate (4p)

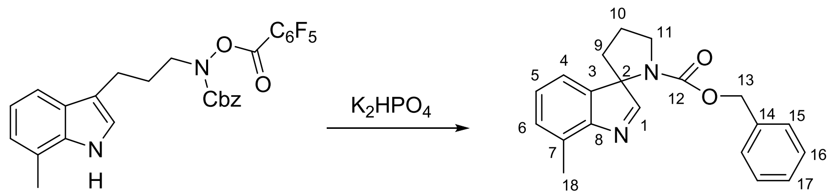

<u>**General procedure F**</u>**:** Benzyl (3-(7-methyl-1*H*-indol-3-yl)propyl)((perfluorobenzoyl) oxy)carbamate **3p** (80.0 mg, 0.15 mmol) and K_2HPO_4 (4.0 mg, 0.025 mmol) were employed. After stirring at 140 °C for 21 h, purification of the product by flash column chromatography (33%–50% EtOAc/Hexane) afforded **4p** (43.3 mg, 90%, 1:2 mixture of rotamers *A:B*) as a colorless viscous oil.

IR (film) ν_{max} / cm^{-1}: 2954 (m), 1698 (s), 1406 (s), 1352 (s), 1128 (m).

^{1}H NMR (400 MHz, $CDCl_3$) δ 7.96 (s, 0.31H, C**1**-<u>H</u>, *A*), 7.90 (s, 0.69H, C**1**-<u>H</u>, *B*), 7.34 – 7.30 (m, 2H, Ph-<u>H</u>, *A*+*B*; C**5**-<u>H</u>, *A*+*B*), 7.17 – 7.06 (m, 5H, Ph-<u>H</u>$_4$, *A*+*B*; C**6**-<u>H</u>, *A*+*B*), 6.66 (d, *J* = 7.2 Hz, 1H, C**4**-<u>H</u>, *A*+*B*), 5.07 (d, *J* = 12.4 Hz, 0.34H, C**13**-<u>H</u>, *A*), 4.96 (d, *J* = 12.5 Hz, 0.35H, C**13**-<u>H</u>’, *A*), 4.85 (d, *J* = 12.5 Hz, 0.76H, C**13**-<u>H</u>, *B*), 4.70 (d, *J* = 12.4 Hz, 0.75H, C**13**-<u>H</u>’, *B*), 3.92 (dt, *J* = 12.3, 6.3 Hz, 1H, C**11**-<u>H</u>, *A*+*B*), 3.80 (dt, *J* = 10.2, 6.8 Hz, 1H, C**11**-<u>H</u>’, *A*+*B*), 2.57 (s, 1H, C**18**-<u>H</u>, *A*), 2.44 (s, 2H, C**18**-<u>H</u>, *B*), 2.37 – 2.21 (m, 2H, C**10**-<u>H</u>, *A*+*B*, C**9**-<u>H</u>, *A*+*B*), 2.13 (dq, *J* = 12.5, 6.3, 5.7 Hz, 1H, C**9**-<u>H</u>’, *A*+*B*), 2.03 – 1.91 (m, C**10**-<u>H</u>’, *A*+*B*).

^{13}C NMR (101 MHz, $CDCl_3$) δ 173.21 (C**1**, *B*), 173.09 (C**1**, *A*), 153.97 (C**12**, *B*), 152.81(C**12**, *A*), 152.38 (C**5**, *A*), 152.06 (C**5**, *B*), 140.75 (C**3**, *B*), 139.93 (C**3**, *A*), 136.62 (C**8**, *A*+*B*), 135.67 (C**7**, *A*+*B*), 131.27 (C**4**, *A*+*B*), 130.67 (C1**4**, *A*), 130.33 (C1**4**, *B*), [128.57, 128.16, 127.64, 127.46, 126.77, 126.68 (C**15**, *A*+*B*); (C**16**, *A*+*B*), C**17** (*A*+*B*)], 118.31 (C**6**, *A*+*B*), 75.91 (C**2**, *A*+*B*), 67.16 (C**13**, *A*+*B*), 48.74 (C**11**, *B*), 48.02 (C**11**, *A*), 35.55 (C**10**, *B*), 34.45 (C**10**, *A*), 24.47 (C**9**, *A*), 23.86 (C**9**, *B*), 16.67 (C**18**, *A*), 16.51 (C**18**, *B*).

ESI-HRMS: m/z calculated for $C_{20}H_{20}N_2NaO_2$ $[M+Na]^+$: 343.1417; found: 343.1433.

tert-Butyl 4'-methylspiro[indole-3,2'-pyrrolidine]-1'-carboxylate (4q)

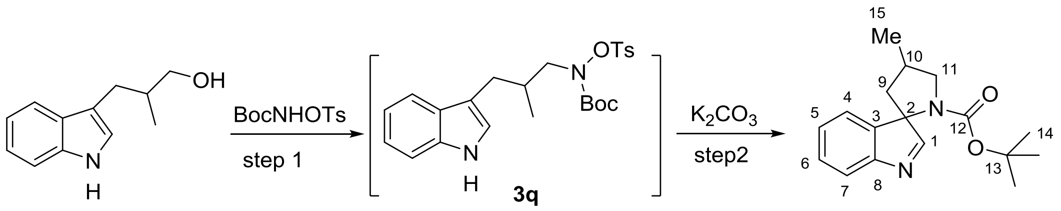

<u>Step 1. General procedure D</u>: 3-(1*H*-Indol-3-yl)-2-methylpropan-1-ol (0.54 g, 2.85 mmol), BocNHOTs **2e'** (984 mg, 3.42 mmol), PPh_3 (897 g, 3.42 mmol), DIAD (0.72 mL, 3.42 mmol) and THF (25 mL) were employed. Purification of the product by flash column chromatography (PhMe) afforded *tert*-butyl (3-(1*H*-indol-3-yl)-2-methylpropyl)(tosyloxy)carbamate **3q** (740 mg), *in which containing another by-product. We tried diffierent methods but failed to obtain a pure **3q**, so we used the mixture directly for the next dearomatization process.*

The by-product from this step is 3-(1H-indol-3-yl)-2-methylpropyl 4-methylbenzenesulfonate (280 mg), which can be easily removed after the second step (see below).

ESI-HRMS for **3q**: m/z calculated for $C_{17}H_{22}N_2NaO_2[M+Na]^+$: 481.1768; found: 481.1772.

<u>Step 2. General procedure G</u>: *tert*-Butyl (3-(1*H*-indol-3-yl)-2-methylpropyl)(tosyloxy) carbamate **3q** (728 mg, containing 280 mg of *3-(1H-indol-3-yl)-2-methylpropyl 4-methylbenzenesulfonate, see below*), K_2CO_3 (506 mg, 3.67 mmol) and TFE (30 mL) were employed. After stirring at 60 °C for 18 h, purification of the product by flash column chromatography (20%–50% EtOAc/Hexane) afforded **4q** (110 mg, 39%, 2:1 d.r, *A:B*) as a colorless viscous oil.

*The yield for step 2 is calculated as following: SM = 728 mg – 280 mg = 528 mg (0.977 mmol); The yield of **4q** = 110 ÷ 286.375 ÷ 0.977 × 100% = 39%.*

Data for **3q**:

IR (film) ν_{max} / cm^{-1}: 2965 (m), 2928 (w), 1690 (s), 1389 (s), 1364 (s), 1158 (s), 753 (s).

^{1}H NMR (500 MHz, CD_3CN, 65 °C) δ 7.98 (d, *J* = 1.6 Hz, 1H, C**1**-<u>H</u>, *A*), 7.94 (d, *J* = 1.6 Hz, 0.5H, C**1**-<u>H</u>, *B*), 7.44 (dd, *J* = 7.7, 4.7 Hz, 1H, C**4**-<u>H</u>, *A+B*), 7.39 – 7.30 (m, 3H, C**5**-<u>H</u>, *A+B;* C**7**-<u>H</u>, *A+B*), 7.29 – 7.21 (m, 1H, C**6**-<u>H</u>, *A+B*), 4.01 – 3.88 (m, 1.5H, C**11**-<u>H</u>, *A+B*), 3.23 (td, *J* = 10.4, 2.0 Hz, 1H, C**11**-<u>H'</u>, *A*), 3.16 (t, *J* = 10.0 Hz, 0.5H, C**11**-<u>H'</u>, *B*), 2.79 – 2.69 (m, 0.5H, C**10**-<u>H</u>, *B*), 2.53 (dddd, *J* = 13.7, 12.3, 10.3, 6.6 Hz, 1H, C**10**-<u>H</u>, *A*), 2.14 (dd, *J* = 13.1, 6.3 Hz,

1.5H, C**9**-H, *A*+*B*), 1.80 (s, 1H, C**9**-H', *A*+*B*), 1.42 – 1.22 (m, 4.5H, C**14**-H, *B*), 1.17 – 1.14 (m, 5H, C**15**-H, *A*+*B*), 1.07 – 0.85 (m, 9H, C**14**-H, *A*).

^{13}C NMR (126 MHz, CD_3CN, 65 °C) δ 176.99 (C**1**, *B*), 175.56 (C**1**, *A*), 155.75 (C**12**, *A*), 155.49 (C**12**, *B*), 129.55 (C**5**, *B*), 129.38 (C**5**, *A*), 127.65 (C**6**, *A*), 127.47 (C**6**, *B*), 122.35 (C**4**, *A*), 122.11 (C**4**, *B*), 122.01 (C**7**, *A*), 121.94 (C**7**, *B*), 80.61 (C**13**, *B*), 80.35 (C**13**, *B*), 77.84 (C**2**, *A*), 77.78 (C**2**, *B*), 56.15 (C**11**, *A* + *B*), 45.00 (C**10**, *A* + *B*), 28.33 (C**14**, *A* + *B*), 17.55 (C**15**, *B*), 17.08 (C**15**, *A*). *The signals corresponding to C3, C8 could not be resolved due to their weak intensity*.

ESI-HRMS: m/z calculated for $C_{17}H_{22}N_2NaO_2$[M+Na]$^+$: 309.1573; found: 309.1583.

We also isolated 280 mg of **3-(1*H*-Indol-3-yl)-2-methylpropyl 4-methylbenzenesulfonate**.

IR (film) ν_{max} / cm^{-1}: 3414 (br m), 2925 (w), 1352 (m), 1173 (s), 963 (s), 742 (s).

^{1}H NMR (400 MHz, $CDCl_3$) δ 7.98 (s, 1H), 7.80 – 7.71 (m, 2H), 7.52 – 7.44 (m, 1H), 7.35 (dt, *J* = 8.1, 0.9 Hz, 1H), 7.31 (s, 2H), 7.22 – 7.14 (m, 1H), 7.08 (ddd, *J* = 8.0, 7.1, 1.0 Hz, 1H), 6.92 (d, *J* = 2.3 Hz, 1H), 3.99 – 3.84 (m, 2H), 2.81 (dd, *J* = 14.5, 6.9 Hz, 1H), 2.61 (dd, *J* = 14.5, 7.1 Hz, 1H), 2.26 – 2.13 (m, 1H), 0.95 (d, *J* = 6.8 Hz, 3H).

^{13}C NMR (101 MHz, $CDCl_3$) δ 144.73, 136.39, 133.24, 129.91, 128.00, 127.68, 122.53, 122.07, 119.40, 118.94, 113.41, 111.24, 74.72, 33.91, 28.46, 21.78, 16.91.

ESI-HRMS: m/z calculated for $C_{19}H_{21}NnaO_3$[M+Na]$^+$: 366.1140; found: 366.1157.

***tert*-Butyl (3*S**,5'*R**)-5'-methylspiro[indole-3,2'-pyrrolidine]-1'-carboxylate (4r)**

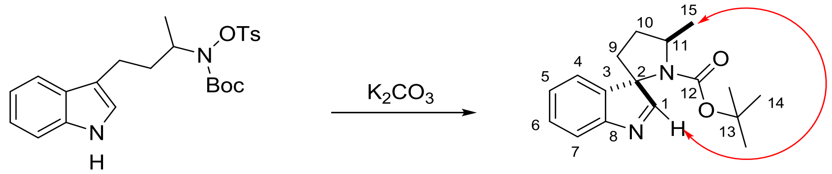

General procedure G: *tert*-Butyl (4-(1*H*-indol-3-yl)butan-2-yl)(tosyloxy)carbamate **3r** (68.8 mg, 0.15 mmol) and K_2CO_3 (41.5 mg, 0.3 mmol) were employed. After stirring at 80 °C for 25 h, purification of the product by flash column chromatography (20%–50% EtOAc/Hexane) afforded **4r** (22.0 mg, 51%, 14:1 d.r) as a colorless viscous oil.

IR (film) ν_{max} / cm^{-1}: 2974 (m), 1694 (s), 1454 (m), 1366 (s), 1163 (m), 751 (m).

^{1}**H NMR** (400 MHz, $CDCl_3$) δ 7.90 (s, 1H, C**1**-H), 7.50 (d, *J* = 7.5 Hz, 1H, C**4**-H), 7.32 (t, *J* = 7.4 Hz, 1H, C**5**-H), 7.24 (d, *J* = 7.6 Hz, 1H, C**7**-H), 7.19 (t, *J* = 7.2 Hz, 1H, C**6**-H), 4.42 (p, *J* = 6.4 Hz, 1H, C**11**-H), 2.58 – 2.47 (m, 1H, C**10**-H), 2.47 – 2.36 (m, 1H, C**9**-H), 1.75 (ddd, *J* = 19.0, 12.0, 6.1 Hz, 2H, C**9**-H'; C**10**-H'), 1.35 (s, 3H, C**15**-H_3), 0.96 (s, 9H, *t*Bu, C**14**-H_3 × 3).

^{13}**C NMR** (101 MHz, $CDCl_3$) δ 176.65 (C**1**), 153.66 (C**12**), 153.18 (C**8**), 141.87 (C**3**), 128.66 (C**5**), 126.58 (C**7**), 121.35 (C**4**), 120.51 (C**6**), 80.39 (C**13**), 76.72 (C**2**), 54.98 (C**11**), 31.69 (C**10**), 30.41 (C**9**), 27.74 (C**14**), 20.66 (C**15**).

ESI-HRMS: m/z calculated for $C_{17}H_{22}N_2NaO_2[M+Na]^+$: 309.1573; found: 309.1571.

The relative stereochemistry of this compound was corroborated by nOe experiments (as indicated on the compound structure). An nOe was observed from CH$_3$ to C1-H.

tert-Butyl (3*R*,3'*R*)-3'-methylspiro[indole-3,2'-pyrrolidine]-1'-carboxylate (4s)

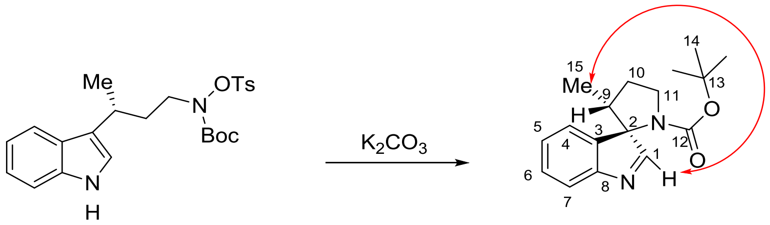

General procedure G: *tert*-Butyl (*R*)-(3-(1*H*-indol-3-yl)butyl)(tosyloxy)carbamate **3s** (68.8 mg, 0.15 mmol) and K_2CO_3 (41.5 mg, 0.3 mmol) were employed. After stirring at 60 °C for 17 h, purification of the product by flash column chromatography (33% EtOAc/Hexane) afforded **4s** (36.7 mg, 85%, 12:1 d.r., *A*:*B*) as a colorless viscous oil.

$[\alpha]_D^{21.9}$ = + 103.5 (*c* = 0.75, $CHCl_3$)

IR (film) ν_{max} / cm^{-1}: 2971 (m), 1691 (s), 1455 (m), 1392 (s), 1152 (s), 746 (s).

^{1}**H NMR** (400 MHz, $CDCl_3$) δ 7.87 (s, 1H, C**1**-H), 7.52 (d, *J* = 7.6 Hz, 1H, C**4**-H), 7.32 (t, *J* = 7.1 Hz, 1H, C**5**-H), 7.25 – 7.14 (m, 2H, C**7**-H, C**6**-H), 3.89 (t, *J* = 9.5 Hz, 1H, C**11**-H), 3.69 (td, *J* = 10.8, 6.1 Hz, 1H, C**11**-H'), 2.58 (dt, *J* = 12.7, 6.5 Hz, 1H, C**9**-H), 2.24 (dt, *J* = 12.6, 6.2 Hz, 1H, C**10**-H), 1.82 (qd, *J* = 11.8, 8.3 Hz, 1H, C**10**-H'), 0.94 (s, 9H, *t*Bu, C**14**-H_3 × 3), 0.74 (d, *J* = 6.9 Hz, 3H, C**15**-H_3).

^{13}**C NMR** (101 MHz, $CDCl_3$) δ 175.70 (C**1**, *B*), 173.21 (C**1**, *A*), 154.48 (C**12**, *A*+ *B*), 153.45 (C**8**, *A*+ *B*), 140.60 (C**3**, *A*+ *B*), 128.77 (C**5**, *B*), 128.59 (C**5**, *A*), 126.63 (C**6**, *A*), 126.13 (C**6**, *B*), 122.20 (C**4**, *B*) , 121.34 (C**4**, *A*), 120.82 (C**7**, *A*+ *B*), 80.13 (C**13**, *A* + *B*), 79.26 (C**2**, *A*+ *B*),

47.14 (C**11**, *A*), 46.59 (C**11**, *B*), 45.71 (C**9**, *A*), 39.79 (C**9**, *B*) 32.70 (C**10**, *A*), 31.23 (C**10**, *B*), 27.77 (C**14**, *A*), 27.72 (C**14**, *B*), 13.36 (C**15**, *A*), 13.11 (C**15**, *B*).

ESI-HRMS: m/z calculated for $C_{17}H_{23}N_2O_2[M+H]^+$: 287.1754; found: 287.1765.

*The relative stereochemistry of this compound was corroborated by nOe experiments (as indicated on the compound structure). nOe was observed from C$\underline{H}_3$ (C**15**) to C**1**-H.*

The enantiomeric purity of this compound was determined by chiral SFCC (Chiralpak IE, CO_2:*i*-PrOH 95:5, 3 mL/min, 25 °C, P_{outlet} 150 Bar, Sample in solution with 100% CH_2Cl_2) against a racemic standard prepared under same conditions; t_R (major) = 9.1 min and t_R (minor) = 10.6 min.

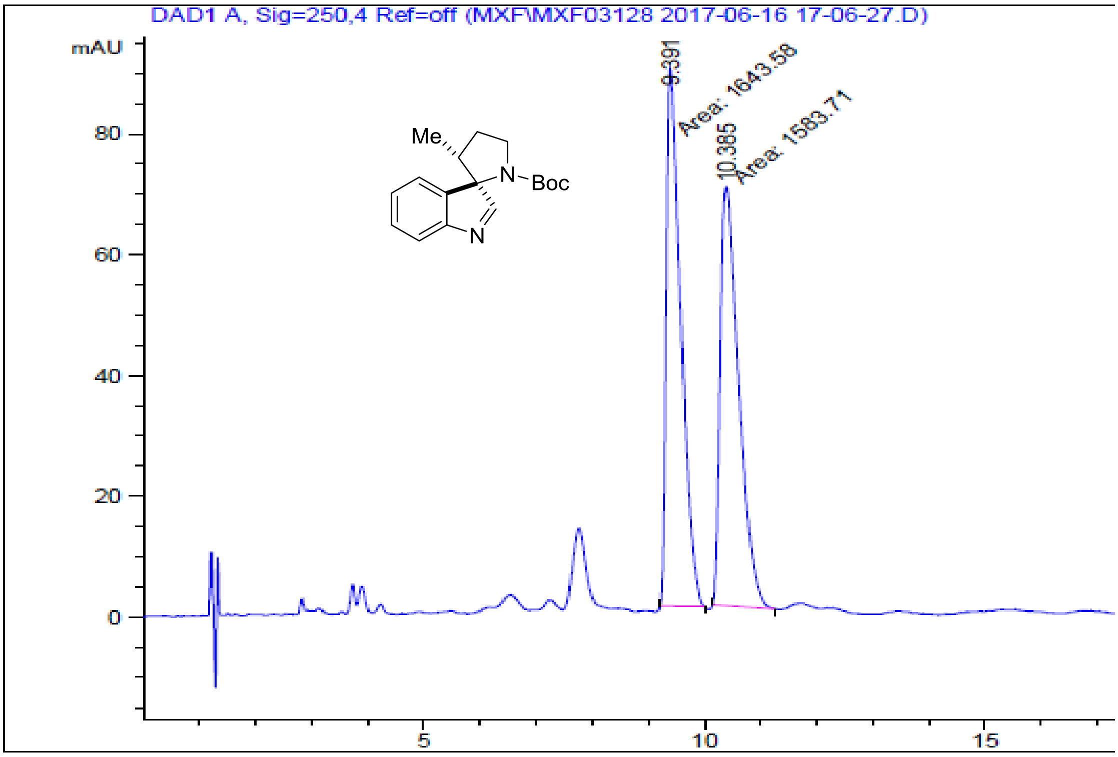

==

Area Percent Report

==

Sorted By : Signal
Multiplier : 1.0000
Dilution : 1.0000
Sample Amount: : 30.00000 [ng/ul] (no
Use Multiplier & Dilution Factor with ISTDs

Signal 1: DAD1 A, Sig=250,4 Ref=off

Peak #	RetTime [min]	Type	Width [min]	Area [mAU*s]	Height [mAU]	Area %
1	9.391	PP	0.3077	1643.57727	89.01918	50.9275
2	10.385	PP	0.3812	1583.71387	69.24937	49.0725

Additional Info : Peak(s) manually integrated

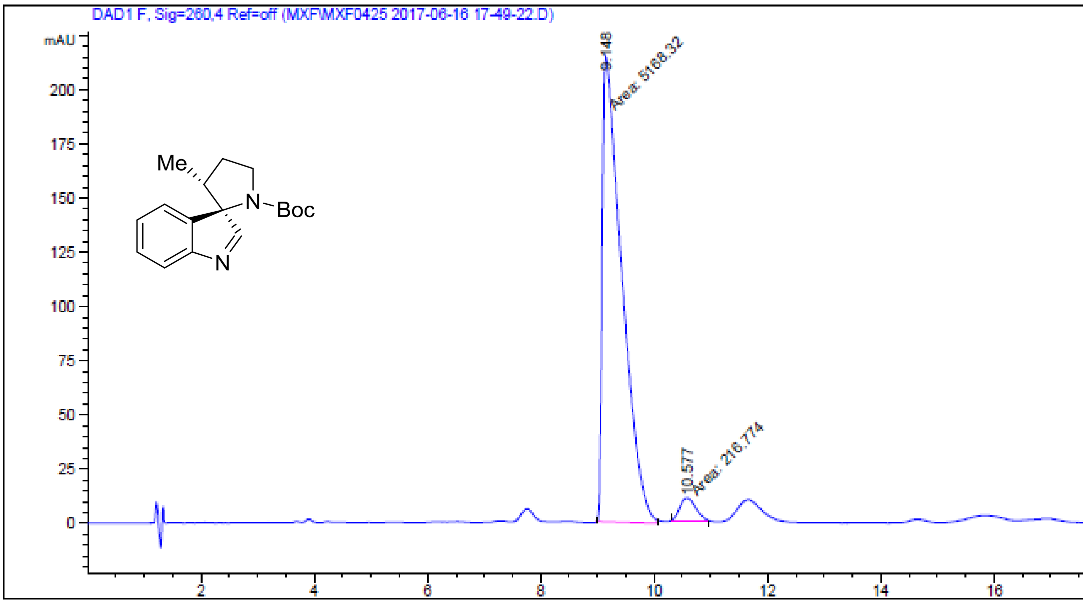

===

Area Percent Report

===

Sorted By : Signal
Multiplier : 1.0000
Dilution : 1.0000
Sample Amount: : 30.00000 [ng/ul] (not used in calc.)
Use Multiplier & Dilution Factor with ISTDs

Signal 1: DAD1 F, Sig=260,4 Ref=off

Peak #	RetTime [min]	Type	Width [min]	Area [mAU*s]	Height [mAU]	Area %
1	9.148	MM	0.4020	5168.32324	214.26555	95.9746
2	10.577	MM	0.3352	216.77437	10.77885	4.0254

tert-Butyl (3_R_*,3'_R_*)-3'-methylspiro[indole-3,2'-pyrrolidine]-1'-carboxylate (4s) (achirality)

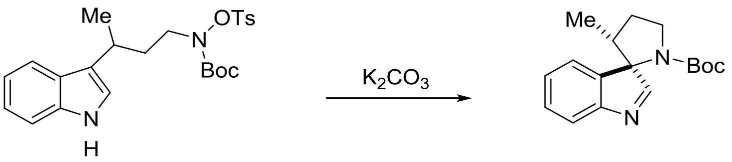

<u>General procedure G</u>: _tert_-Butyl (3-(1_H_-indol-3-yl)butyl)(tosyloxy)carbamate **3s** (achirality) (68.8 mg, 0.15 mmol) and K_2CO_3 (41.5 mg, 0.3 mmol) were employed. After stirring at 60 °C for 17 h, purification of the product by flash column chromatography (33% EtOAc/Hexane) afforded **4s** (37.5 mg, 87%, 12:1 d.r.) as a colorless viscous oil, whose spectral characteristics were concordant with values described above.

tert-Butyl (3_R_*,3'_R_*)-3'-isopropylspiro[indole-3,2'-pyrrolidine]-1'-carboxylate (4t)

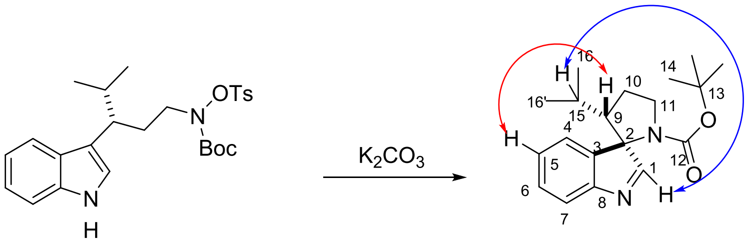

<u>General procedure G</u>: _tert_-Butyl (3-(1_H_-indol-3-yl)-4-methylpentyl)(tosyloxy) carbamate **3t** (73.0 mg, 0.15 mmol) and K_2CO_3 (41.5 mg, 0.3 mmol) were employed. After stirring at 80 °C for 48 h, purification of the product by flash column chromatography (20%–50% EtOAc/Hexane) afforded **4t** (30.3 mg, 64%, 14:1 d.r.) as a colorless solid.

m.p.: 68 - 70 ° C (EtOAc/hexane).

IR (film) ν_{max} / cm^{-1}: 2973 (m), 1689 (s), 1670 (s), 1392 (s), 1367 (s), 1145 (s).

^{1}H NMR (400 MHz, $CDCl_3$) δ 7.94 (s, 1H, C**1**-<u>H</u>), 7.47 (d, _J_ = 7.4 Hz, 1H, C**4**-<u>H</u>), 7.37 – 7.26 (m, 1H, C**5**-<u>H</u>), 7.18 (d, _J_ = 2.7 Hz, 2H, C**7**-<u>H</u>, C**6**-<u>H</u>), 3.86 (t, _J_ = 9.4 Hz, 1H, C**11**-<u>H</u>), 3.58 (dt, _J_ = 16.8, 8.5 Hz, 1H, C**11**-<u>H</u>’), 2.31 (dd, _J_ = 12.1, 6.5 Hz, 1H, C**9**-<u>H</u>), 2.19 (dt, _J_ = 11.3, 5.5 Hz, 1H, C**10**-<u>H</u>), 1.94 – 1.72 (m, 1H, C**10**-<u>H</u>’), 1.52 – 1.39 (m, 1H, C**15**-<u>H</u>), 0.92 (s, 9H, _t_Bu, C**14**-$\underline{H_3}$ × 3), 0.84 (d, _J_ = 6.6 Hz, 3H, C**16**-$\underline{H_3}$), 0.29 (d, _J_ = 6.5 Hz, 3H, C**16**-$\underline{H_3}$’).

^{13}C NMR (101 MHz, $CDCl_3$) δ 172.54 (C**1**), 155.03 (C**12**), 153.30 (C**8**), 141.95 (C**3**), 128.62 (C**5**), 126.58 (C**6**), 121.43 (C**4**), 120.97 (C**7**), 80.17 (C**13**), 78.31 (C**2**), 58.01 (C**9**), 46.87 (C**11**), 28.75 (C**10**), 28.16 (C**15**), 27.74 (C**14**), 22.14 (C**16**), 21.84 (C**16**’).

ESI-HRMS: m/z calculated for $C_{19}H_{26}N_2NaO_2$[M+Na]$^+$: 337.1886; found: 337.1901.

The relative stereochemistry of this compound was corroborated by nOe experiments (as indicated on the compound structure). nOes were observed from C$\underline{H_3}$ <u>(C**15**)</u> to C**1**-H and from C**9**-H to C**5**-H.

tert-Butyl (3_R_*,3'_R_*)-3'-phenylspiro[indole-3,2'-pyrrolidine]-1'-carboxylate (4u)

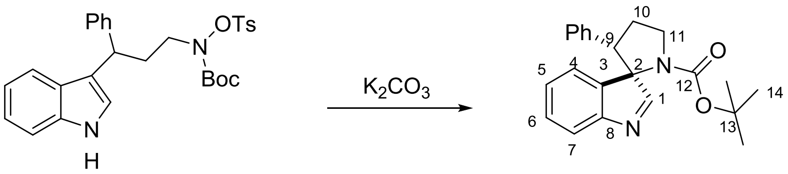

General procedure G: *tert*-Butyl (3-(1*H*-indol-3-yl)-3-phenylpropyl)(tosyloxy) carbamate **3u** (78.1 mg, 0.15 mmol) and K_2CO_3 (41.5 mg, 0.3 mmol) were employed. After stirring at 60 °C for 24 h, purification of the product by flash column chromatography (33%–50% EtOAc/Hexane) afforded **4u** (39.4 mg, 75%, > 20:1 d.r.) as a colorless solid.

m.p.: 126 - 128 °C (EtOAc/Hexane).

IR (film) ν_{max} / cm^{-1}: 2976 (m), 1689 (s), 1392 (s), 1168 (m), 1140 (m).

^{1}H NMR (400 MHz, $CDCl_3$) δ 7.71 (s, 1H, C**1**-H), 7.42 – 7.36 (m, 1H, Ar-H), 7.36 – 7.28 (m, 3H, Ar-H × 3), 7.18 – 7.06 (m, 3H, Ar-H × 3), 6.76 (d, *J* = 7.3 Hz, 2H, Ar-H × 2), 4.11 (t, *J* = 9.4 Hz, 1H, C**11**-H), 3.83 (td, *J* = 10.6, 6.0 Hz, 1H, C**11**-H'), 3.75 (dd, *J* = 12.5, 5.6 Hz, 1H, C**10**-H), 2.72 – 2.52 (m, 1H, C**9**-H), 2.42 (dt, *J* = 12.1, 5.9 Hz, 1H, C**9**-H'), 0.95 (s, 9H, *t*Bu, C**14**-H_3 × 3).

^{13}C NMR (101 MHz, $CDCl_3$) δ 172.27 (C**1**), 154.87 (C**12**), 151.66 (C**8**), 148.61 (C**3**), 135.15 (Ar-C), 128.82 (Ar-C), 128.35 (Ar-C), 127.87 (Ar-C), 121.44 (Ar-C), 120.93 (Ar-C), 80.43 (C**13**), 78.98 (C**2**), 55.46 (C**9**), 47.01 (C**11**), 28.99 (C**10**), 27.74 (C**14**).

ESI-HRMS: m/z calculated for $C_{22}H_{24}N_2NaO_2[M+Na]^+$: 371.1730; found: 371.1737.

The structure and relative stereochemistry of this compound was determined unambiguously by X-ray crystallography.

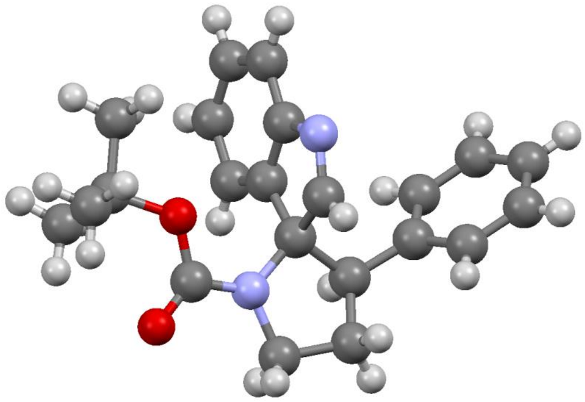

3-(4-((*tert*-Butyldimethylsilyl)oxy)phenyl)propanoic[17]

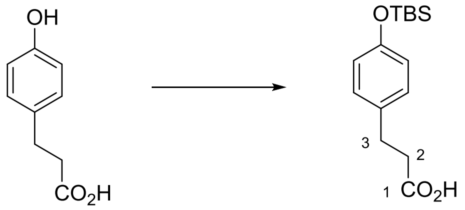

<u>General procedure B</u>: 3-(4-Hydroxyphenyl)propanoic acid (8.30 g, 50.0 mmol), *tert*-butyldimethylsilyl chloride (16.5 g, 110.0 mmol), imidazole (11.25 g, 165.0 mmol) in DMF (100 mL) were employed. Purification of the product by flash column chromatography (25% EtOAc/Hexane) afforded the title compound (10.8 g, 77%) as a colorless solid.

IR (solid) ν_{max} / cm^{-1}: 2926 (m), 2882, (m, br), 2855 (m), 1714 (s), 1509 (s), 1249 (s), 1213 (s).

^{1}H NMR (400 MHz, $CDCl_3$) δ 7.05 (d, *J* = 8.3 Hz, 2H, ArC<u>H</u>), 6.76 (d, *J* = 8.3 Hz, 2H, ArC<u>H</u>), 2.89 (t, *J* = 7.6 Hz, 2H, C**3**-<u>H</u>$_2$), 2.64 (t, *J* = 7.3 Hz, 2H, C**2**-<u>H</u>$_2$), 0.97 (s, 9H, TBS (C<u>H</u>$_3$)$_3$), 0.18 (s, 6H, TBS (C<u>H</u>$_3$)$_2$).

^{13}C NMR (101 MHz, $CDCl_3$) δ 179.0 (C**1**), 154.1 (Ar<u>C</u>), 132.8 (Ar<u>C</u>), 129.1 (2 × Ar<u>C</u>H), 120.0 (2 × Ar<u>C</u>H), 35.8 (C**2**), 29.8 (C**3**), 25.7 (TBS (<u>C</u>H$_3$)$_3$), 18.2 (TBS Si<u>C</u>(CH$_3$)$_3$), -4.5 (TBS Si(C<u>H</u>$_3$)$_2$).

ESI-HRMS: m/z calculated for $C_{15}H_{25}O_3Si[M+H]^+$: 281.1567; found: 281.1580.

The title compound has been described only in a patent.[17]

3-(4-((*tert*-Butyldimethylsilyl)oxy)phenyl)propan-1-ol[18]

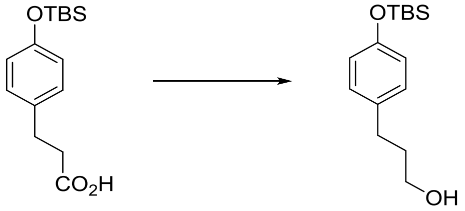

<u>General procedure A</u>: 3-(4-((*tert*-Butyldimethylsilyl)oxy)phenyl)propanoic acid (1.40 g, 5.0 mmol) and 2.0 eq. $LiAlH_4$ (1M in THF) in anhydrous Et_2O were employed. Purification of the product by flash column chromatography (25% EtOAc/Hexane) afforded the title compound (0.99 g, 74%) as a colorless oil.

IR (film) ν_{max} / cm^{-1}: 3339 (br m), 2929 (s), 2885 (s), 2858 (s), 1609 (m), 1508 (s), 1250 (s).

¹H NMR (400 MHz, $CDCl_3$) δ 7.04 (d, *J* = 8.0 Hz, 2H), 6.75 (d, *J* = 8.0 Hz, 2H), 3.64 – 3.68 (m, 2H), 2.64 (t, *J* = 7.4 Hz, 2H), 1.86 (m, 2H), 1.35, (br s, 1H), 0.98 (s, 9H), 0.18 (s, 6H).

¹³C NMR (101 MHz, $CDCl_3$) δ 153.7, 134.4, 129.2, 119.9, 62.3, 34.4, 31.2, 25.7, 18.2, -4.4.

Spectroscopic properties were consistent with the data available in the literature.[18]

***tert*-Butyl (3-(4-((*tert*-butyldimethylsilyl)oxy)phenyl)propyl)(tosyloxy)carbamate**

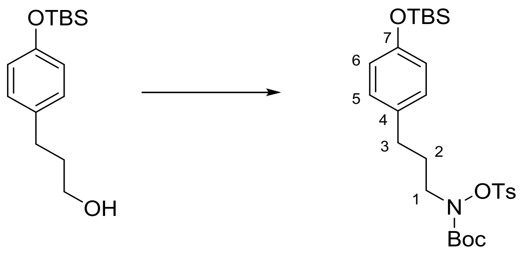

General procedure D: 3-(4-((*tert*-Butyldimethylsilyl)oxy)phenyl)propan-1-ol (0.79 g, 3.00 mmol), PPh_3 (0.94 g, 3.60 mmol), DIAD (0.71 mL, 3.60 mmol) and TsONHBoc (1.03 g, 3.60 mmol) in THF (12 mL) were employed. Purification of the product by flash column chromatography (10% EtOAc/Hexane) afforded the title compound (1.49 g, 93%) as a pale yellow oil.

IR (film) ν_{max} / cm^{-1}: 2955 (m), 2930 (m), 2858 (m), 1753 (m), 1720 (s), 1509 (s), 1382 (s), 1368 (s), 1251 (s), 1178 (s), 1154 (s), 1093 (m).

¹H NMR (400 MHz, $CDCl_3$) δ 7.84 (d, *J* = 8.3 Hz, 2H, Ts ArC<u>H</u>), 7.33 (d, *J* = 8.3 Hz, 2H, Ts ArC<u>H</u>), 7.00 (d, *J* = 8.4 Hz, 2H, C**5**-<u>H</u>), 6.74 (d, *J* = 8.4 Hz, 2H, C**6**-<u>H</u>), 3.60 (app. br s, 2H, C**1**-<u>H</u>$_2$), 2.52 (t, *J* = 7.8 Hz, 2H, C**3**-<u>H</u>$_2$), 2.45 (s, 3H, Ts C<u>H</u>$_3$), 1.95-1.85 (m, 2H, C**2**-<u>H</u>$_2$), 1.22 (s, 9H, Boc (C<u>H</u>$_3$)$_3$), 0.98 (s, 9H, TBS (C<u>H</u>$_3$)$_3$), 0.18 (s, 6H, TBS (C<u>H</u>$_3$)$_2$).

¹³C NMR (101 MHz, $CDCl_3$) δ 155.5 (C=O), 153.8 (C**7**), 145.6 (Ts Ar<u>C</u>), 133.7 (C**4**), 131.3 (Ts Ar<u>C</u>), 129.6 (Ts Ar<u>C</u>H), 129.5(Ts Ar<u>C</u>H), 129.1 (C**5**), 120.0 (C**6**), 83.2 (Boc <u>C</u>(CH_3)$_3$), 52.6 (C**1**), 32.0 (C**3**), 27.6 (Boc (<u>C</u>H_3)$_3$), 27.5 (C**2**), 25.7 (TBS (<u>C</u>H_3)$_3$), 21.7 (Ts <u>C</u>H_3), 18.2 (TBS Si<u>C</u>(CH_3)$_3$), -4.4 (TBS Si(<u>C</u>H_3)$_2$).

ESI-HRMS: m/z calculated for $C_{27}H_{41}NNaO_6SSi[M+Na]^+$: 558.2316; found: 558.2313.

***tert*-Butyl (3-(4-hydroxyphenyl)propyl)(tosyloxy)carbamate (5a)**

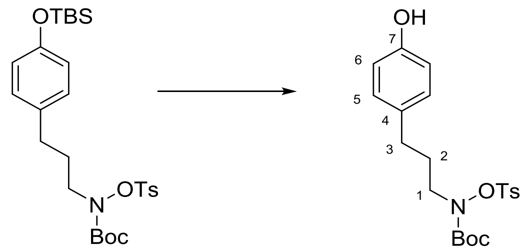

<u>General procedure E</u>: *tert*-Butyl (3-(4-((*tert*-butyldimethylsilyl)oxy)phenyl)propyl)(tosyloxy)carbamate (0.69 g, 1.28 mmol) and 1.0 eq. [1:1 TBAF/HOAc solution (0.1 M in THF)] in THF (20 mL) were employed. Purification of the product by flash column chromatography (20% EtOAc/Hexane) afforded the title compound (0.35 g, 60%) as a colorless solid.

m.p.: 63 - 65 ^{o}C (EtOAc/hexane).

IR (solid) ν_{max} / cm^{-1}: 3426 (m, br), 2982 (m), 2934 (m), 1721 (s), 1515 (s), 1369 (s), 1191 (s), 1177 (s), 1152 (s), 1092 (m).

^{1}H NMR (400 MHz, $CDCl_3$) δ 7.83 (d, *J* = 8.3 Hz, 2H, Ts ArC<u>H</u>)), 7.32 (d, *J* = 8.3 Hz, 2H, Ts ArC<u>H</u>), 6.98 (d, *J* = 8.4 Hz, 2H, C**5**-<u>H</u>), 6.75 (d, *J* = 8.4 Hz, 2H, C**6**-<u>H</u>), 5.75 (br s, 1H, O<u>H</u>), 3.60 (app. br s, 2H, C**1**-<u>H</u>$_2$), 2.50 (t, *J* = 7.8 Hz, 1H, C**3**-<u>H</u>$_2$), 2.44 (s, 3H, Ts C<u>H</u>$_3$), 1.97 – 1.80 (m, 1H, C**2**-<u>H</u>$_2$), 1.23 (s, 9H, Boc (C<u>H</u>$_3$)$_3$).

^{13}C NMR (101 MHz, $CDCl_3$) δ 155.7 (C=O), 154.1 (C**7**), 145.9 (Ts Ar<u>C</u>), 132.8 (C**4**), 131.0 (Ts Ar<u>C</u>), 129.6 (2 × Ts Ar<u>C</u>H), 129.5 (2 × Ts Ar<u>C</u>H), 129.3 (C**5**), 115.3 (C**6**), 83.6 (Boc <u>C</u>(CH$_3$)$_3$), 52.6 (C**1**), 31.9 (C**3**), 27.7 (C**2**), 27.6 (Boc (<u>C</u>H$_3$)$_3$), 21.7 (Ts, <u>C</u>H$_3$).

ESI-HRMS: m/z calculated for $C_{21}H_{27}NnaO_6S$[M+Na]$^+$: 444.1451; found: 444.1434.

Methyl (*E*)-3-(4-(benzyloxy)-3,5-dimethylphenyl)acrylate

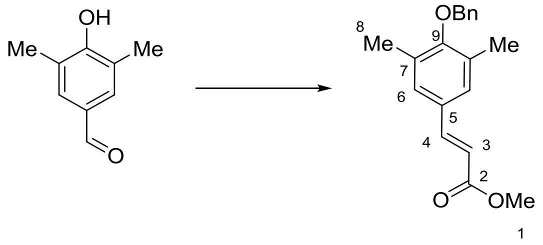

4-(Benzyloxy)-3,5-dimethylbenzaldehyde (2.40 g, 10.0 mmol) and methyl 2-(triphenyl-phosphaneylidene) acetate (5.00 g, 15.0 mmol) in CH_2Cl_2 (15 mL) were were stirred at r.t. overnight and monitored by TLC. Upon completion, the reaction mixture was concentrated *in vacuo* and purified by flash column chromatography (10–20% EtOAc/Hexane) afforded the title compound (2.85 g, 96%) as a colorless oil.

IR (film) ν_{max}/ cm^{-1}: 3030 (w), 2949 (w), 2920 (w), 1713 (s), 1632 (m), 1434 (m), 1265 (s), 1143 (s).

^{1}H NMR (400 MHz, $CDCl_3$) δ 7.63 (d, *J* = 16.0 Hz, 1H, C**4**-$\underline{H}_2$), 7.50 – 7.46 (m, 2H, PhC$\underline{H}$), 7.46 – 7.33 (m, 3H, PhC$\underline{H}$), 7.23 (s, 2H, C**6**-$\underline{H}$), 6.36 (d, *J* = 16.0 Hz, 1H, C**3**-$\underline{H}$), 4.83 (s, 2H, OC$\underline{H}_2$), 3.81 (s, 3H, C**1**-$\underline{H}_3$), 2.32 (s, 6H, C**8**-$\underline{H}_3$).

^{13}C NMR (101 MHz, $CDCl_3$) δ 167.7 (C**2**), 157.9 (C**9**), 144.8 (C**4**), 137.4 (Ph$\underline{C}$), 131.9 (2 × C**7**), 130.2 (C**5**), 129.0 (2 × C**6**), 128.7 (2 × Ph$\underline{C}$H), 128.2 (Ph$\underline{C}$H), 127.9(2 × Ph$\underline{C}$H), 116.6 (C**3**), 74.2 (O$\underline{C}H_2$), 51.7 (C**1**), 16.6 (C**8**).

ESI-HRMS: m/z calculated for $C_{19}H_{20}NaO_3$ $[M+Na]^+$: 319.1305; found: 319.1311.

Methyl 3-(4-hydroxy-3,5-dimethylphenyl)propanoate[19]

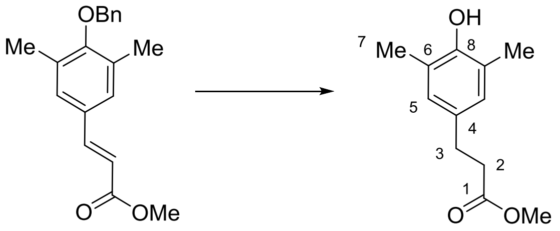

A solution of methyl (*E*)-3-(4-(benzyloxy)-3,5-dimethylphenyl) acrylate (2.37 g, 8.00 mmol) in MeOH (50 mL) was purged with argon before the addition of 10 wt. % Pd/C (10 mol%). The flask was fitted with a balloon of hydrogen and stirred at room temperature overnight and monitored by TLC. Upon completion, the reaction mixture was filtered over a bed of Celite® washing with the appropriate solvent and concentrated *in vacuo* to afford the title compound (1.66 g, 99%) as a colorless solid, which was used without further purification.

m.p.: 66 - 68 °C (EtOAc/hexane).

IR(solid) ν_{max}/ cm^{-1}: 3492 (s, br), 2952 (m), 2928 (m), 1723 (s), 1277 (s), 1174 (s), 1151 (s).

^{1}H NMR (400 MHz, $CDCl_3$) δ 6.81 (s, 2H, C**5**-$\underline{H}$), 4.55 (br s, 1H, O$\underline{H}$), 3.67 (s, 3H, OC$\underline{H}_3$), 2.82 (t, *J* = 7.8 Hz, 2H, C**2**-$\underline{H}_2$), 2.58 (t, *J* = 7.8 Hz, 2H, C**3**-$\underline{H}_2$), 2.22 (s, 6H, C**7**-$\underline{H}_3$).

^{13}C NMR (101 MHz, $CDCl_3$) 173.6, 150.6, 132.1, 128.4, 123.0, 51.6, 36.1, 30.1, 15.9.

ESI-HRMS: m/z calculated for $C_{12}H_{16}NaO_3$ $[M+Na]^+$: 231.0992; found: 231.1002.

The title compound has been described only in a patent.[19]

Methyl 3-(4-((*tert*-butyldimethylsilyl)oxy)-3,5-dimethylphenyl)propanoate

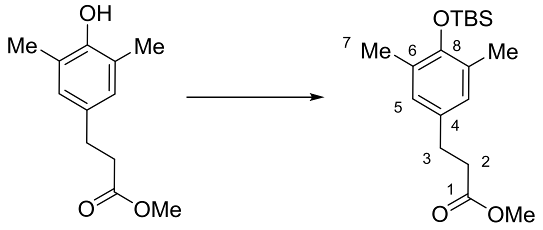

To a solution of methyl 3-(4-hydroxy-3,5-dimethylphenyl)propanoate (1.33 g, 6.00 mmol) in CH_2Cl_2 (10 mL) and DMF (12 mL) was added *tert*-butyldimethylsilyl chloride (1.8 g, 12.0 mmol) and imidazole (0.82 g, 12.0 mmol) at 0 ^{o}C. The reaction was stirred at rt overnight and quenched with H_2O (50 mL) and the organic phase extracted with CH_2Cl_2 (3 × 15 mL), washed with brine (15 mL), dried over $MgSO_4$, filtered and concentrated *in vacuo*. Purification of the product by flash column chromatography (10% EtOAc/Hexane) afforded the title compound (1.22 g, 63%) as a colorless oil.

IR (film) ν_{max}/ cm^{-1}: 2953 (m), 2930 (m), 1740 (s), 1484 (m), 1473 (m), 1253 (s), 1228 (s), 1153 (s).

^{1}H NMR (400 MHz, $CDCl_3$) δ 6.78 (s, 2H, C**5**-H), 3.66 (s, 3H, OCH$_3$), 2.81 (t, *J* = 7.8 Hz, 2H, C**3**-H$_2$), 2.58 (*J* = 7.8 Hz, 2H, t, C**2**-H$_2$), 2.17 (s, 6H, C**7**-H$_3$), 1.02 (s, 9H, TBS (CH$_3$)$_3$), 0.17 (s, 6H, TBS (CH$_3$)$_2$).

^{13}C NMR (101 MHz, $CDCl_3$) δ 173.6 (C**1**), 150.4 (C**8**), 133.0 (C**4**), 128.5 (C**5**), 128.4 (C**6**), 51.5 (OCH$_3$), 36.0 (C**2**), 30.1 (C**3**), 26.1 (TBS (CH$_3$)$_3$), 18.7 (TBS C(CH$_3$)$_3$), 17.8 (C**7**), -3.0 (TBS Si(CH$_3$)$_2$).

ESI-HRMS: m/z calculated for $C_{18}H_{30}NaO_3Si$ $[M+Na]^+$: 345.1856; found: 345.1870.

3-(4-((*tert*-Butyldimethylsilyl)oxy)-3,5-dimethylphenyl)propan-1-ol[20]

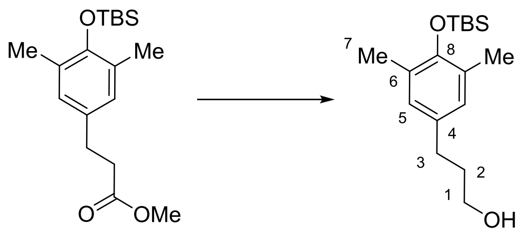

<u>General procedure A</u>: Methyl 3-(4-((*tert*-butyldimethylsilyl)oxy)-3,5-dimethylphenyl) propanoate (0.96 g, 3.0 mmol) and 2.0 eq. $LiAlH_4$ (1 M in THF) in anhydrous Et_2O were employed. The crude product was filtered through a plug of silica and washed with EtOAc to afford the title compound (0.69 g, 80%) as a pale yellow oil.

IR (film) ν_{max}/ cm^{-1}: 3345 (br m), 2929 (m), 2858 (m), 1483 (s), 1472 (s), 1252 (s), 1227 (s), 1152 (s).

^{1}H NMR (400 MHz, $CDCl_3$) δ 6.79 (s, 2H), 3.66 (t, *J* = 6.4 Hz, 2H), 2.57 (t, *J* = 7.5 Hz 2H), 2.18 (s, 6H), 1.89 – 1.81 (m, 2H), 1.29 (br s, 1H), 1.03 (s, 9H), 0.18 (s, 6H).

^{13}C NMR (101 MHz, $CDCl_3$) δ 150.1, 134.2, 128.6, 128.3, 62.5, 34.3, 31.2, 26.1, 18.7, 17.8, -3.0.

Spectroscopic data were consistent with the data available in the literature[20]

tert-Butyl (3-(4-((*tert*-butyldimethylsilyl)oxy)-3,5-dimethylphenyl)propyl)(tosyloxy) carbamate

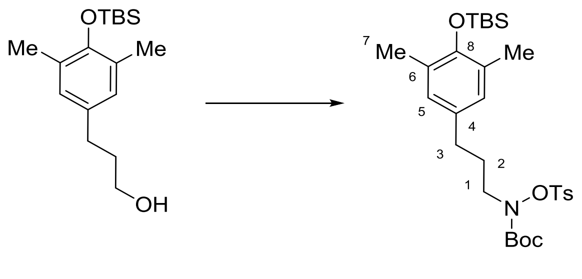

<u>General procedure D</u>: 3-(4-((*tert*-Butyldimethylsilyl)oxy)-3,5-dimethylphenyl)propan-1-ol (0.53 g, 1.80 mmol), PPh_3 (0.58 g, 2.20 mmol), DIAD (0.43 mL, 2.20 mmol) and TsONHBoc (0.63 g, 2.20 mmol) in anhydrous THF (8 mL) were employed. Purification of the product by flash column chromatography (10% EtOAc/Hexane) afforded the title compound (0.88 g, 87%) as a colorless oil.

IR (film) ν_{max}/ cm^{-1}: 2955 (m), 2930 (m), 1722 (s), 1473 (m), 1483 (m), 1383 (s), 1369 (s), 1230 (s), 1191 (s), 1179 (s), 1154 (s).

^{1}H NMR (400 MHz, $CDCl_3$) δ 7.84 (d, *J* = 8.3 Hz, 2H, Ts ArCH), 7.32 (d, *J* = 8.3 Hz, 2H, Ts ArCH), 6.75 (s, 2H, C**5**-H), 3.59 (app. br s, 2H, C**1**-H_2), 2.46 – 2.42 (m, 5H, overlapping C**3**-H_2 and Ts CH_3), 2.16 (s, 6H, C**7**-H_3) 1.93 – 1.81 (m, 2H, C**2**-H_2), 1.21 (s, 9H, Boc $(CH_3)_3$), 1.02 (s, 9H, TBS $(CH_3)_3$), 0.17 (s, 6H, TBS $Si(CH_3)_2$).

^{13}C NMR (101 MHz, $CDCl_3$) δ 155.6 (Boc C=O), 150.4 (C**8**), 145.7 (Ts ArC), 133.7 (C**4**), 131.4 (Ts ArC), 129.8 (2 × Ts ArCH), 129.6 (2 × Ts ArCH), 128.7 (C**5**), 128.5 (C**6**), 83.2 (Boc $C(CH_3)_3$), 52.9 (C**1**), 32.1 (C**3**), 27.8 (C**2**), 27.7 (Boc $(CH_3)_3$), 26.3 (TBS $(CH_3)_3$), 21.9 (Ts CH_3), 18.9 (TBS $C(CH_3)_3$), 18.0 (C**7**), -2.8 (TBS $(CH_3)_2$).

ESI-HRMS: m/z calculated for $C_{29}H_{45}NNaO_6SSi$ $[M+Na]^+$: 586.2629; found: 586.2648.

***tert*-Butyl (3-(4-hydroxy-3,5-dimethylphenyl)propyl)(tosyloxy)carbamate (5b)**

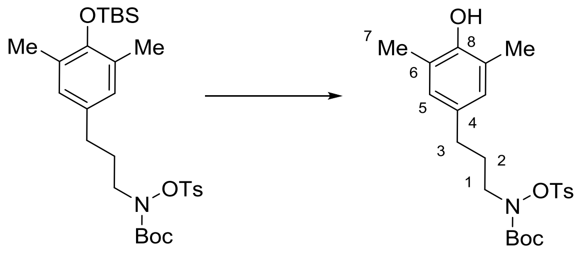

General procedure E: *tert*-Butyl (3-(4-((*tert*-butyldimethylsilyl)oxy)-3,5-dimethylphenyl) propyl) (tosyloxy)carbamate (0.45 g, 0.80 mmol) and 1.1 eq. [1:1 TBAF/HOAc solution (0.1 M in THF)] in THF (16 mL) were employed. Purification of the product by flash column chromatography (20–33% EtOAc/Hexane) afforded the title compound (0.31 g, 87%) as a colorless, viscous oil.

IR (film) ν_{max}/ cm^{-1}: 3530 (br m), 2979 (m), 2930 (m), 1721 (s), 1597 (m), 1489 (m), 1370 (s), 1192 (s), 1177 (s), 1152 (s).

^{1}H NMR (400 MHz, $CDCl_3$) δ 7.84 (d, *J* = 8.3 Hz, 2H, Ts ArCH), 7.32 (d, *J* = 8.3 Hz, 2H, Ts ArCH), 6.77 (s, 2H, C**5**-H), 4.49 (s, 1H, OH), 3.62 (app. br s, 2H, C**1**-H_2), 2.44 (m, 5H, overlapping C**3**-H_2 and Ts CH_3), 2.21 (s, 6H, C**7**-H_3), 1.93 – 1.85 (m, 2H, C**2**-H_2), 1.20 (s, 9H, Boc $(CH_3)_3$).

^{13}C NMR (101 MHz, $CDCl_3$) δ 155.4 (Boc C=O), 150.4 (C**8**), 145.6 (Ts ArC), 132.6 (C**4**), 131.2 (Ts ArC), 129.7 (2 × Ts ArCH), 129.5 (2 × Ts ArCH), 128.4 (C**5**), 122.9 (C**6**), 83.2 (Boc $C(CH_3)_3$), 52.7 (C**1**), 31.9 (C**3**), 27.7 (C**2**), 27.6 (Boc $(CH_3)_3$), 21.7 (Ts CH_3), 15.9 (C**7**).

ESI-HRMS: m/z calculated for $C_{23}H_{31}NNaO_6S$ $[M+Na]^+$: 472.1764; found: 472.1767.

Ethyl 3-(3-bromo-4-hydroxyphenyl)propanoate[21]

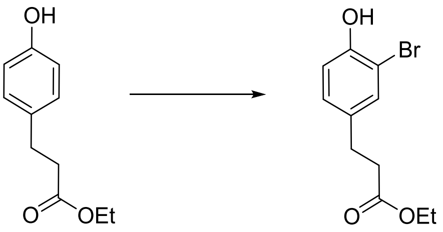

A solution of bromine (0.25 mL, 4.75 mmol) in acetic acid (20 mL) was slowly added to a stirring solution of ethyl 3-(4-hydroxyphenyl)propionate (1.84 g, 9.50 mmol) at room temperature. The reaction mixture was stirred for 6 h then diluted with EtOAc (80 mL) and washed with brine (2 × 30 mL). The organic extracts were dried over anhydrous $MgSO_4$, filtered and concentrated *in vacuo*. Purification of the product by flash column chromatography (5% EtOAc/PhMe) afforded the title compound (1.14 g, 44%) as a pale yellow solid.

IR (solid) ν_{max}/ cm^{-1}: 3357 (br m), 2977 (m), 2936 (m), 1727 (s), 1704 (s), 1496 (s), 1289 (s), 1254 (s), 1180 (s), 1156 (s), 1039 (s).

^{1}H NMR (400 MHz, $CDCl_3$) 7.29 (d, *J* = 2.0 Hz 1H), 7.03 (dd, *J* = 8.2, 2.0 Hz, 1H,), 6.91 (d, *J* = 8.2 Hz, 1H), 4.11 (q, *J* = 7.2 Hz, 2H,), 2.85 (t, *J* = 8.5 Hz, 2H,), 2.56 (t, *J* = 7.6 Hz, 2H), 1.22 (t, *J* = 7.2 Hz, 3H,).

^{13}C NMR (101 MHz, $CDCl_3$) δ 172.7, 150.7, 134.2, 131.6, 129.1, 116.0, 110.0, 60.5, 36.0, 29.8, 14.2.

Spectroscopic properties are consistent the data available in the literature.[21]

Ethyl 3-(3-bromo-4-((*tert*-butyldimethylsilyl)oxy)phenyl)propanoate

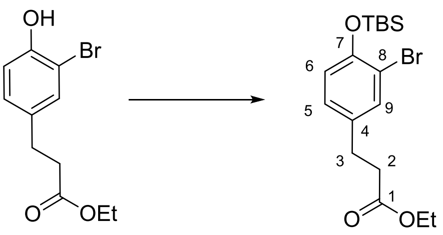

To a solution of ethyl 3-(3-bromo-4-hydroxyphenyl)propanoate (1.08 g, 3.95 mmol) in DMF (5 mL) was added *tert*-butyldimethylsilyl chloride (0.71 g, 4.74 mmol) and imidazole (0.67 g, 9.88 mmol) and the reaction was stirred at room temperature overnight. To the reaction was added water (25 mL) and the organic phase extracted with CH_2Cl_2 (2 x 20 mL). The combined organic extracts were washed with brine (20 mL), dried over $MgSO_4$, filtered and concentrated *in vacuo*. Purification of the product by flash column chromatography (5% EtOAc/Hexane) afforded the title compound (1.43 g, 93%) as a colorless oil.

IR (film) ν_{max}/ cm^{-1}: 2956 (m), 2930 (m), 1734 (s), 1493 (s), 1287 (s), 1253 (s).

^{1}H NMR (400 MHz, $CDCl_3$) δ 7.35 (d, *J* = 2.2 Hz, 1H, C**9**-H), 6.98 (dd, *J* = 8.4, 2.2 Hz, 1H, C**5**-H), 6.77 (d, *J* = 8.4 Hz, 1H, C**6**-H), 4.11 (q, *J* = 7.1 Hz, 2H, O$C\underline{H}_2$), 2.84 (t, *J* = 7.7 Hz, 2H, C**3**-$\underline{H}_2$), 2.56 (t, *J* = 7.7 Hz, 2H, C**2**-$\underline{H}_2$), 1.22 (t, *J* = 7.1 Hz, 3H, $CH_2C\underline{H}_3$), 1.03 (s, 9H, TBS $(C\underline{H}_3)_3$), 0.22 (s, 6H, TBS $(C\underline{H}_3)_2$.

^{13}C NMR (101 MHz, $CDCl_3$) δ 172.8 (C**1**), 151.1 (C**7**), 134.9 (C**4**), 133.2 (C**9**), 128.2 (C**5**), 120.2 (C**6**), 115.2 (C**8**), 60.6 (O$\underline{C}H_2CH_3$), 36.1 (C**2**), 29.9 (C**3**), 25.9 (TBS $(\underline{C}H_3)_3$), 18.5 (TBS Si$\underline{C}(CH_3)_3$), 14.4 ($OCH_2\underline{C}H_3$), -4.11 (TBS Si$(\underline{C}H_3)_2$).

ESI-HRMS: m/z calculated for $C_{17}H_{27}BrNaO_3Si$ $[M+Na]^+$: 409.0805; found: 409.0816.

3-(3-Bromo-4-((*tert*-butyldimethylsilyl)oxy)phenyl)propan-1-ol

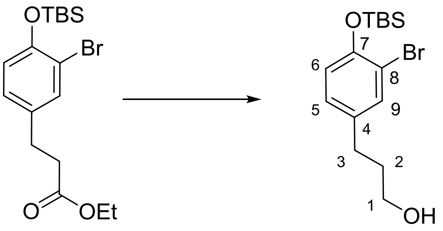

To a solution of ethyl 3-(3-bromo-4-((*tert*-butyldimethylsilyl)oxy)phenyl)propanoate (1.03 g, 2.66 mmol) in anhydrous THF (15 mL) at -15 °C was added 0.75 eq. $LiAlH_4$ (1M in THF) and the reaction was stirred at the same temperature for 25 min. Then to the reaction mixture was added water (0.5 mL), aq. 1 M NaOH (0.2 mL) and water (1 mL). The reaction mixture was

warmed to room temperature, filtered through Celite® and washed with CH_2Cl_2. The filtrate was dried over Na_2SO_4, filtered and concentrated *in vacuo*. Purification of the product by flash column chromatography (20% EtOAc/Hexane) afforded the title compound (0.80 g, 87%) as a colorless oil.

IR (film) ν_{max}/ cm^{-1}: 3327 (br m), 2930 (m), 2858 (m), 1492 (s), 1280 (s), 1253 (s).

^{1}H NMR (400 MHz, $CDCl_3$) δ 7.34 (d, *J* = 2.2 Hz, 1H, C**9**-H), 6.97 (dd, *J* = 8.2, 2.2 Hz, 1H, C**5**-H), 6.77 (dd, *J* = 8.2, 0.8 Hz, 1H, C**6**-H), 3.65 (t, *J* = 6.5 Hz, 2H, C**1**-H_2), 2.61 (t, *J* = 7.5 Hz, 2H, C**3**-H_2), 1.87 – 1.80 (m, 2H, C**2**-H_2), 1.41 (br s, 1H, OH), 1.03 (s, 9H, TBS $(CH_3)_3$), 0.23 (s, 6H, TBS $(CH_3)_2$).

^{13}C NMR (101 MHz, $CDCl_3$) δ 150.8 (C**7**), 136.1 (C**4**), 133.2 (C**9**), 128.3 (C**5**), 120.2 (C**6**), 115.2 (C**8**), 62.1 (C**1**), 34.2 (C**2**), 31.0 (C**3**), 25.9 (TBS $(CH_3)_3$), 18.5 (TBS $C(CH_3)_3$), -4.1 (TBS $Si(CH_3)_2$).

ESI-HRMS: m/z calculated for $C_{15}H_{25}BrNaO_2Si$ $[M+Na]^+$: 367.0699; found: 367.0701.

tert-Butyl (3-(3-bromo-4-((tert-butyldimethylsilyl)oxy)phenyl)propyl) (tosyloxy) carbamate

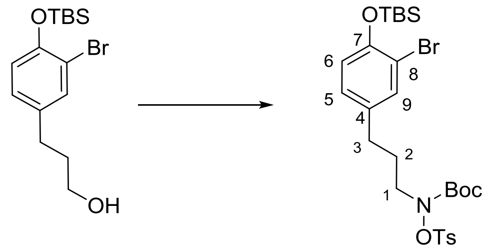

<u>General procedure D</u>: 3-(3-Bromo-4-((*tert*-butyldimethylsilyl)oxy)phenyl)propan-1-ol (0.69 g, 2.00 mmol), PPh_3 (0.63 g, 2.40 mmol), DIAD (0.47 mL, 2.40 mmol) and TsONHBoc (0.69 g, 2.40 mmol) in anhydrous THF were employed. Purification of the product by flash column chromatography (5%–10% EtOAc/Hexane) afforded the title compound (1.12 g, 91%) as a colorless oil.

IR (film) ν_{max}/ cm^{-1}: 2955 (m), 2930 (m), 2858 (m), 1720 (s), 1493 (s), 1381 (s), 1368 (s), 1288 (s), 1254 (s), 1178 (s).

^{1}H NMR (400 MHz, $CDCl_3$) δ 7.83 (d, *J* = 8.2 Hz, 2H, Ts ArCH), 7.32 (d, *J* = 8.0 Hz, 2H, Ts ArCH), 7.29 (d, *J* = 2.1 Hz, 1H, C**9**-H), 6.94 (dd, *J* = 8.3, 2.2 Hz, 1H, C**5**-H), 6.77 (d, *J* = 8.6

Hz, 1H, C**6**-H), 3.58 (app. br s, 2H, C**1**-H_2), 2.49 (t, *J* = 7.8 Hz, 2H, C**3**-H_2), 2.44 (s, 3H, Ts CH_3), 1.95 – 1.82 (m, 2H, C**2**-H_2), 1.22 (s, 9H, Boc $(CH_3)_3$), 1.03 (s, 9H, TBS $(CH_3)_3$), 0.23 (s, 6H, $Si(CH_3)_2$).

^{13}C NMR (101 MHz, $CDCl_3$) δ 155.6 (C=O), 150.9 (C**7**), 145.8 (Ts ArC), 135.4 (C**4**), 133.1 (C**9**), 131.4 (Ts ArC), 129.8 (Ts ArCH), 129.7 (Ts ArCH), 128.2 (C**5**), 120.2 (C**6**), 115.2 (C**8**), 83.4 (Boc $C(CH_3)_3$), 52.6 (C**1**), 31.8 (C**3**), 27.8 (Boc $(CH_3)_3$), 27.5 (C**2**), 25.9 (TBS $(CH_3)_3$), 21.8 (Ts CH_3), 18.5 (TBS $SiC(CH_3)_3$), -4.1 (TBS $Si(CH_3)_2$).

ESI-HRMS: m/z calculated for $C_{27}H_{40}BrNNaO_6SSi$ $[M+Na]^+$: 636.1421; found: 636.1422.

***tert*-Butyl (3-(3-bromo-4-hydroxyphenyl)propyl)(tosyloxy)carbamate (5c)**

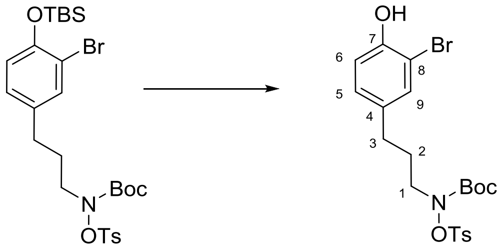

<u>**General procedure E**</u>**:** *tert*-Butyl (3-(3-bromo-4-((*tert*-butyldimethylsilyl)oxy)phenyl) propyl) (tosyloxy)carbamate (0.61 g, 1.00 mmol) and 1.0 eq. [1:1 solution of TBAF/AcOH (0.1 M in THF)] in THF (20 mL) were employed. Purification of the product by flash column chromatography (20% EtOAc/Hexane) afforded the title compound (0.48 g, 96%) as a colorless solid.

m.p.: 93 - 95 °C (EtOAc/hexane).

IR (solid) ν_{max}/ cm^{-1}: 3416 (br s), 2945 (m), 1682 (s), 1371 (s), 1361 (s), 1180 (s), 1158 (s).

^{1}H NMR (400 MHz, $CDCl_3$) δ 7.83 (d, *J* = 8.3 Hz, 2H, Ts ArCH), 7.32 (d, *J* = 8.1 Hz, 2H, Ts ArCH), 7.25 (d, *J* = 2.2 Hz, 1H, C**9**), 7.00 (dd, *J* = 8.3, 2.1 Hz, 1H, C**5**), 6.91 (d, *J* = 8.3 Hz, 1H, C**6**), 5.42 (1H, s, OH), 3.59 (app. br s, 2H, C**1**-H_2), 2.50 (t, *J* = 7.8 Hz, 2H, C**3**-H_2), 2.44 (s, 3H, Ts CH_3), 1.91 – 1.87 (m, 2H, C**2**-H_2), 1.21 (s, 9H, Boc $(CH_3)_3$).

^{13}C NMR (101 MHz, $CDCl_3$) δ 155.6 (C=O), 150.6 (C**7**), 145.9 (Ts ArC), 134.9 (C**4**), 131.6 (C**9**), 131.3 (Ts ArC), 129.8 (2 × Ts ArCH), 129.7 (2 × Ts ArCH), 129.2 (C**5**), 116.1 (C**6**),

110.1 (C**8**), 83.5 (Boc C(CH3)3), 52.5(C**1**), 31.7 (C**3**), 27.8 (Boc (CH3)3), 27.6 (C**2**), 21.8 (Ts CH3).

ESI-HRMS: m/z calculated for $C_{21}H_{26}BrNO_6S$ $[M+Na]^+$: 522.0556; found: 522.0555.

Ethyl (*E*)-3-(4-hydroxyphenyl)acrylate[22]

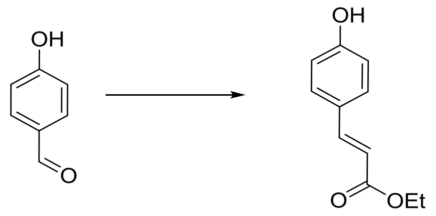

4-Hydroxybenzaldehyde (4.88 g, 40.0 mmol) and ethyl (triphenylphosphoranylidene)acetate (20.9 g, 60.0 mmol) in CH_2Cl_2 (40 mL) were stirred at r.t. overnight and monitored by TLC. Upon completion, the reaction mixture was concentrated *in vacuo* and purified by flash column chromatography (20% EtOAc/Hexane) afforded the title compound (6.56 g, 85%) as a colorless solid.

^{1}H NMR (400 MHz, $CDCl_3$) δ 7.64 (d, *J* = 16.0 Hz, 1H), 7.42 (d, *J* = 8.6 Hz, 2H), 6.86 (d, *J* = 8.3 Hz, 2H), 6.30 (d, *J* = 16.0 Hz, 1H), 6.14 (br s, 1H), 4.27 (q, *J* = 7.1 Hz, 2H), 1.34 (t, *J* = 7.1 Hz, 3H).

^{13}C NMR (101 MHz) δ 168.1, 158.1, 144.9, 132.4, 130.1, 127.2, 116.1, 115.5, 115.1, 60.8, 14.5.

Spectroscopic properties were consistent with the data available in the literature.[22]

Ethyl (*E*)-3-(4-((tert-butyldimethylsilyl)oxy)phenyl)acrylate[23]

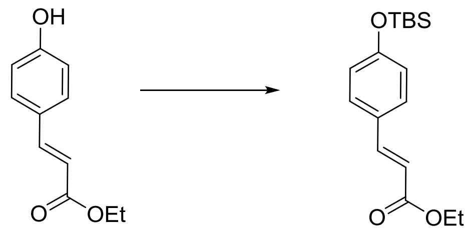

To a solution of ethyl (*E*)-3-(4-hydroxyphenyl)acrylate (3.84 g, 20.0 mmol) in DMF (20 mL) were added *tert*-butyldimethylsilyl chloride (3.60 g, 24.0 mmol) and imidazole (3.40 g, 50.0 mmol) and the reaction was stirred overnight at room temperature until completion by TLC

analysis. Purification of the product by flash column chromatography (20% EtOAc/Hexane) afforded the title compound (5.13 g, 84%) as a colorless oil.

^{1}H NMR (400 MHz, $CDCl_3$) δ 7.63 (d, *J* = 16.0 Hz, 1H), 7.41 (d, *J* = 8.6 Hz, 1H), 6.83 (d, *J* =8.5 Hz, 2H), 6.30 (d, *J* = 16.0 Hz, 1H), 4.25 (q, *J* = 7.3 Hz, 2H), 1.33 (t, *J* = 7.3 Hz, 3H), 0.98 (s, 9H, TBS (C<u>H</u>$_3$)$_3$), 0.22 (s, 6H, TBS Si(C<u>H</u>$_3$)$_2$).

^{13}C NMR (101 MHz, $CDCl_3$) δ 167.5, 157.9, 144.4, 129.8, 127.9, 120.6, 116.1, 60.4, 25.8, 18.4, 14.5, -4.2.

Spectroscopic properties were consistent with the data available in the literature.[23]

Ethyl 3-(4-((*tert*-butyldimethylsilyl)oxy)phenyl)pentanoate

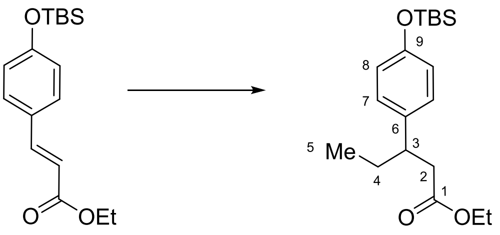

CuI (2.86 g, 15.0 mmol) in anhydrous Et_2O (60 mL) was stirred under nitrogen at room temperature until a suspension was observed. The mixture was cooled to -20 ^{o}C and EtMgBr (3.0 M solution in Et_2O, 37.5 mmol) was added. After stirring for 5 min, a solution of ethyl (*E*)-3-(4-((*tert*-butyldimethylsilyl)oxy)phenyl)acrylate (4.6 g, 15.0 mmol) in anhydrous Et_2O (15 mL) was added dropwise over 1 h. After stirring at -20 ^{o}C for 4 h, MeOH (15 mL) and sat. aq. NH_4Cl (60 mL) were sequentially added and the mixture was warmed to room temperature. After extracting with Et_2O (3 × 20 mL), the combined organic extracts were dried over anhydrous Na_2SO_4, filtered and concentrated *in vacuo*. Purification of the product by flash column chromatography (20% EtOAc/Hexane) afforded the title compound (4.36 g, 86%) as a pale yellow oil.

IR (film) ν_{max}/ cm^{-1}: 2958 (m), 2930 (m), 2858 (m), 1735 (s), 1509 (s), 1252 (s), 1165 (m);

^{1}H NMR (400 MHz, $CDCl_3$) δ 7.02 (d, *J* = 8.4 Hz, 2H, C**7**-H), 6.75 (d, *J* = 8.6 Hz, 2H, C**8**-H), 4.02 (q, *J* = 7.1 Hz, 2H, OCH_2CH_3), 2.97 – 2.89 (m, 1H, C**3**-H), 2.59 (dd, *J* = 14.9, 7.0 Hz, 1H, C**2**-H), 2.50 (dd, *J* = 14.8, 8.3 Hz, 1H, C**2**-H'), 1.73 – 1.61 (m, 1H, C**4**-H), 1.59 – 1.49 (m, 1H, C**4**-H'), 1.12 (t, *J* = 7.1 Hz, 3H, OCH_2CH_3), 0.97 (s, 9H, TBS $(CH_3)_3$), 0.77 (t, *J* = 7.3 Hz, 3H, C**5**-H_3), 0.18 (s, 6H, TBS $Si(CH_3)_2$).

^{13}C NMR (101 MHz, $CDCl_3$) δ 172.7 (C**1**), 154.1 (C**9**), 136.7 (C**6**), 128.5 (C**7**), 119.9 (C**8**), 60.2 (OCH_2CH_3), 43.4 (C**3**), 41.9 (C**2**) 29.4 (C**4**), 25.8 (TBS $(CH_3)_3$), 18.3 (TBS $SiC(CH_3)_3$), 14.3 (OCH_2CH_3), 12.0 (C**5**), -4.4 (TBS $Si(CH_3)_2$).

ESI-HRMS: m/z calculated for $C_{19}H_{32}NaO_3Si$ $[M+Na]^+$: 359.2013; found: 359.2016.

3-(4-((*tert*-Butyldimethylsilyl)oxy)phenyl)pentan-1-ol

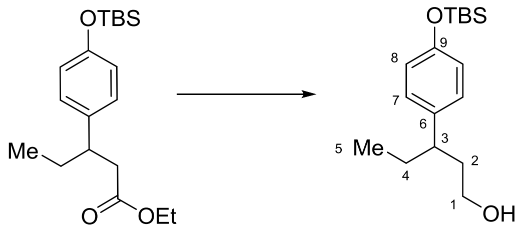

General procedure A: Ethyl 3-(4-((*tert*-butyldimethylsilyl)oxy)phenyl)pentanoate (4.10 g, 12.2 mmol), 1.0 eq. $LiAlH_4$ (1M in THF) and anhydrous Et_2O were employed. The title compound (3.03 g, 84%) was obtained as a colorless oil which was used without further purification.

IR (film) ν_{max}/ cm^{-1}: 3354 (br m), 2956 (m), 2929 (m), 2858 (m), 1607 (m), 1508 (s), 1253 (s).

^{1}H NMR (400 MHz, $CDCl_3$) δ 6.99 (d, *J* = 8.4 Hz, 2H, C**7**-H), 6.76 (d, *J* = 8.5 Hz, 2H, C**8**-H), 3.55 – 3.42 (m, 2H, C**1**-H_2), 2.55 – 2.47 (m, 1H, C**3**-H), 1.94 – 1.87 (m, 1H, C**2**-H), 1.79 – 1.72 (m, 1H, C**2**-H'), 1.70 – 1.61 (m, 1H, C**4**-H), 1.57 – 1.49 (m, 1H, C**4**-H'), 0.98 (s, 9H, TBS $(CH_3)_3$), 0.76 (t, *J* = 7.4 Hz, 3H, C**5**-H_3), 0.18 (s, 6H, TBS $Si(CH_3)_2$).

^{13}C NMR (101 MHz, $CDCl_3$) δ 153.8 (C**9**), 137.5 (C**6**), 128.4 (C**7**-H), 119.9 (C**8**-H), 61.4 (C**1**), 43.6 (C**3**), 39.4 (C**2**), 29.9 (C**4**), 25.7 (TBS C($\underline{C}H_3)_3$), 18.2 (TBS $\underline{C}(CH_3)_3$), 12.1 (C**5**), -4.4 (TBS Si($\underline{C}H_3)_2$).

ESI-HRMS: m/z calculated for $C_{17}H_{30}NaO_2Si$ $[M+Na]^+$: 317.1907; found: 317.1917.

***tert*-Butyl (3-(4-((*tert*-butyldimethylsilyl)oxy)phenyl)pentyl)(tosyloxy)carbamate**

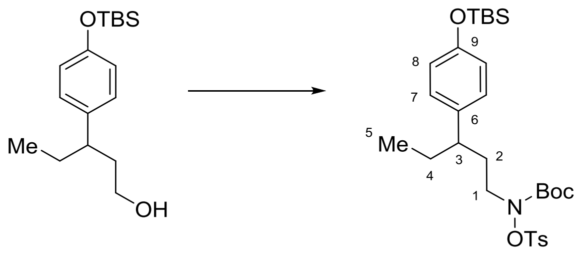

<u>**General procedure D:**</u> 3-(4-((*tert*-Butyldimethylsilyl)oxy)phenyl)pentan-1-ol (2.94 g, 10.0 mmol), PPh_3 (3.15 g, 12.0 mmol mmol), DIAD (2.36 mL, 12.0 mmol) and TsONHBoc (3.44 g, 12.0 mmol) in anhydrous THF (40 mL) were employed. Purification of the product by flash column chromatography (5% EtOAc/Hexane) afforded the title compound (5.35 g, 95%) as a colorless oil.

IR (film) ν_{max}/ cm^{-1}: 2962 (m), 2931 (m), 1721 (s), 1509 (s), 1382 (s), 1369 (s), 1253 (s), 1191 (s), 1155 (s).

^{1}H NMR (400 MHz, $CDCl_3$) δ 7.78 (d, *J* = 8.2 Hz, 2H, Ts ArC<u>H</u>), 7.28 (d, *J* = 8.2 Hz, 2H, Ts ArC<u>H</u>), 6.93 (d, *J* = 8.4 Hz, 2H, C**7**-<u>H</u>), 6.74 (d, *J* = 8.4 Hz, 2H, C**8**-<u>H</u>), 3.48 – 3.19 (m, 2H, C**1**-$\underline{H}_2$), 2.42 (s, 3H, Ts C$\underline{H}_3$), 2.33 – 2.26 (m,1H, C**3**-<u>H</u>), 1.94 (br s, 1H, C**2**-<u>H</u>), 1.76 (br s, 1H, C**2**-<u>H</u>'), 1.66 – 1.57 (m, 1H, C**4**-<u>H</u>), 1.53 – 1.43 (m, 1H, C**4**-<u>H</u>'), 1.22 (s, 9H, Boc (C$\underline{H}_3)_3$), 0.98 (s, 9H, TBS (C$\underline{H}_3)_3$), 0.73 (t, *J* = 7.3 Hz, 3H, C**5**-$\underline{H}_3$).

^{13}C NMR (101 MHz, $CDCl_3$) 155.5 (Boc C=O), 154.0 (C**9**), 145.7 (Ts Ar<u>C</u>), 136.9 (C**6**), 131.4 (Ts Ar<u>C</u>), 129.7 (2 × Ts ArC<u>H</u>), 129.6 (2 × Ts ArC<u>H</u>), 128.4 (C**7**), 120.0 (C**8**) 83.2 (Boc $\underline{C}(CH_3)_3$), 52.0 (C**1**), 44.6 (C**3**), 32.0 (C**2**), 30.1 (C**4**), 27.8 (Boc ($\underline{C}H_3)_3$), 25.8 (TBS C($\underline{C}H_3)_3$), 21.8 (Ts $\underline{C}H_3$), 18.3 (TBS $\underline{C}(CH_3)_3$), 12.1 (C**5**) , -4.3 (TBS ($\underline{C}H_3)_2$).

ESI-HRMS: m/z calculated for $C_{29}H_{45}NO_6SSi$ $[M+Na]^+$: 586.2629; found: 586.2628.

***tert*-Butyl (3-(4-hydroxyphenyl)pentyl)(tosyloxy)carbamate (5d)**

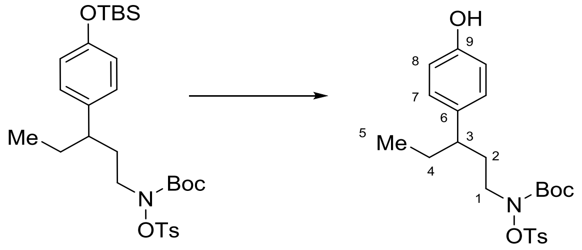

General procedure E: *tert*-Butyl (3-(4-((*tert*-butyldimethylsilyl)oxy)phenyl)pentyl) (tosyloxy)carbamate (2.82 g, 5.0 mmol) and 1.0 eq. 1:1 TBAF/HOAc (0.1 M in THF) in THF (50 mL) were employed. Purification of the product by flash column chromatography (20% EtOAc/Hexane) afforded the title compound (1.80 g, 80%) as a colorless solid.

m.p.: 93 - 95 °C (EtOAc/hexane).

IR (film) ν_{max}/ cm^{-1}: 3436 (br m), 2965 (m), 2930 (m), 1720 (s), 1514 (s), 1368 (s), 1191 (s), 1177 (s), 1153 (s).

^{1}H NMR (400 MHz, $CDCl_3$) δ 7.77 (d, *J* = 8.0 Hz, 2H, Ts ArCH), 7.28 (d, *J* = 8.1 Hz, 2H, Ts ArCH), 6.95 (d, *J* = 8.5 Hz, 2H, C**7**-H), 6.74 (d, *J* 8.5 Hz, 2H, C**8**-H), 4.93 (br s, 1H, OH), 3.51 – 3.16 (app. br s, 2H, C**1**-H_2), 2.42 (s, 3H, Ts CH_3), 2.34 – 2.27 (m, 1H, C**3**-H), 1.95 (br s, 1H, C**2**-H), 1.77 (br s, 1H, C**2**-H'), 1.66 – 1.54 (m, 1H, C**4**-H), 1.52 – 1.43 (m, 1H, C**4**-H'), 1.22 (s, 9H, Boc $(CH_3)_3$), 0.73 (t, *J* = 7.3 Hz, 3H, C**5**-H_3).

^{13}C NMR (101 MHz, $CDCl_3$) δ 155.6 (C=O), 154.1 (C**9**), 145.8 (Ts ArC), 136.3 (C**6**), 131.3 (Ts ArC), 129.7 (2 × Ts ArCH), 129.6 (2 × Ts ArCH), 128.7 (C**7**), 115.4 (C**8**), 83.4 (Boc $C(CH_3)_3$), 51.9 (C**1**), 44.5 (C**3**), 32.0 (C**2**), 30.1 (C**4**), 27.8 (Boc $(CH_3)_3$), 21.8 (Ts CH_3), 12.05 (C**5**).

ESI-HRMS: m/z calculated for $C_{23}H_{31}NNaO_6S$ $[M+H]^+$: 472.1764; found: 472.1763.

3-(4-((*tert*-Butyldimethylsilyl)oxy)naphthalen-1-yl)propan-1-ol

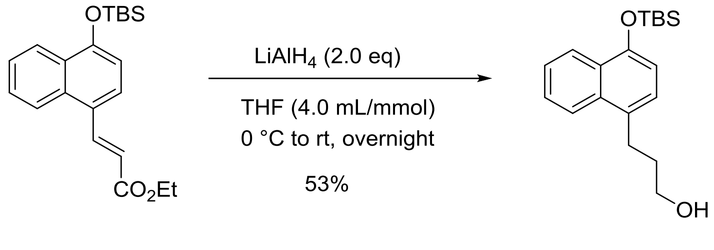

General procedure A: Ethyl (*E*)-3-(4-((*tert*-butyldimethylsilyl)oxy)naphthalen-1-yl) acrylate[23] (1.64 g, 4.59 mmol, 1.0 eq.), $LiAlH_4$ (9.2 mL, 1.0 M in THF) and 10 mL THF were

employed. Purification of the product by flash column chromatography (20–33% EtOAc/Hexane) to afford the title compound (0.77 g, 53%) as a colorless oil.

IR (film) ν_{max}/cm^{-1}: 2988 (s), 2901 (s), 1394 (m), 1275 (m), 1260 (m), 1075 (s), 1066 (s), 1057 (s), 750 (s).

^{1}H NMR (400 MHz, $CDCl_3$) δ 8.27 – 8.19 (m, 1H), 7.99 (dd, *J* = 8.1, 1.5 Hz, 1H), 7.53 – 7.45 (m, 2H), 7.18 (d, *J* = 7.7 Hz, 1H), 6.79 (d, *J* = 7.7 Hz, 1H), 3.74 (t, *J* = 6.4 Hz, 2H), 3.10 (dd, *J* = 8.6, 6.7 Hz, 2H), 2.08 – 1.90 (m, 2H), 1.10 (s, 9H), 0.29 (s, 6H).

^{13}C NMR (101 MHz, $CDCl_3$) δ 150.44, 133.14, 130.58, 128.38, 126.17, 125.99, 124.86, 123.87, 123.45, 112.16, 62.73, 33.75, 28.91, 26.06, 26.00, 18.59, -4.09.

ESI-HRMS: m/z calculated for $C_{19}H_{28}NaO_2Si[M+Na]^+$: 339.1751; found: 339.1763.

tert-Butyl (3-(4-((_tert_-butyldimethylsilyl)oxy)naphthalen-1-yl)propyl)(tosyloxy) carbamate

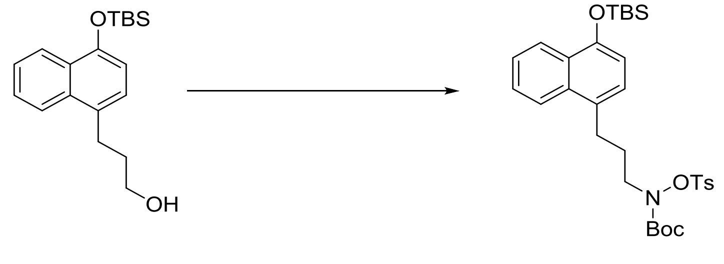

General procedure D: 3-(4-((*tert*-Butyldimethylsilyl)oxy)naphthalen-1-yl)propan-1-ol (0.75 g, 2.36 mmol), BocNHOTs (815 mg, 2.84 mmol, 1.2 eq.), PPh_3 (0.93 g, 2.84 mmol, 1.2 eq.) THF (21 mL) and DIAD (0.7 mL, 2.84 mmol, 1.2 eq.) were employed. Purification of the product by flash column chromatography (25–0% Hexane/PhMe) to afford title compound (1.12 g, 81%) as a white solid.

m.p.: 87 - 89 °C (EtOAc/hexane).

IR (film) ν_{max} / cm^{-1}: 2972(s), 1722 (m), 1393 (s), 1259 (m), 1156 (m), 1075 (s), 750 (s).

^{1}**H NMR** (400 MHz, $CDCl_3$) δ 8.25 – 8.15 (m, 1H), 7.95 – 7.87 (m, 1H), 7.84 (d, *J* = 8.4 Hz, 2H), 7.48 (dddd, *J* = 16.6, 8.1, 6.8, 1.5 Hz, 2H), 7.30 (d, *J* = 7.8 Hz, 2H), 7.13 (d, *J* = 7.7 Hz, 1H), 6.77 (d, *J* = 7.7 Hz, 1H), 3.70 (s, 2H), 2.97 (t, *J* = 7.8 Hz, 2H), 2.43 (s, 3H), 2.16 – 1.89 (m, 2H), 1.19 (s, 9H), 1.10 (s, 9H), 0.28 (s, 6H).

^{13}**C NMR** (101 MHz, $CDCl_3$) δ 155.64, 150.55, 145.77, 133.04, 131.41, 129.84, 129.78, 129.63, 128.37, 126.26, 125.89, 124.87, 123.69, 123.45, 112.12, 83.36, 53.06, 29.68, 27.72, 27.05, 26.06, 21.82, 18.59, -4.09.

ESI-HRMS: m/z calculated for $C_{31}H_{43}NNaO_6SSi$[M+Na]$^+$: 608.2473; found: 608.2473.

tert-Butyl (3-(4-hydroxynaphthalen-1-yl)propyl)(tosyloxy)carbamate (5e)

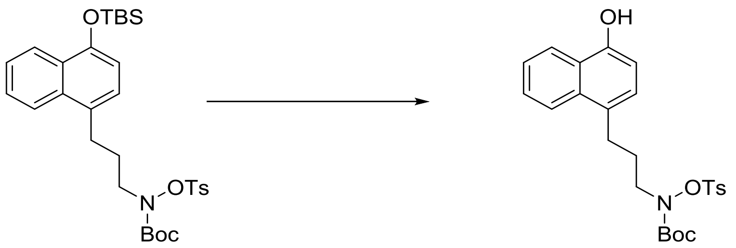

<u>General procedure E</u>: *tert*-Butyl (3-(4-((*tert*-butyldimethylsilyl)oxy)naphthalen-1-yl)propyl) (tosyloxy)carbamate (0.97 g, 1.66 mmol) and 1.0 eq. 1:1 TBAF/AcOH solution (1.0 M in THF) in 17 mL THF were employed. Purification of the product by flash column chromatography (20–30% EtOAc/Hexane) to afford the title compound (0.53 g, 67%) as a viscous colorless oil.

IR (film) ν_{max} / cm^{-1}: 3417 (br, m), 2980 (m), 2871 (m), 1720 (s), 1589 (m), 1370 (s), 1191 (s), 1178 (s), 1151 (s), 763 (s).

^{1}**H NMR** (400 MHz, $CDCl_3$) δ 8.23 (d, *J* = 7.9 Hz, 1H), 7.91 (d, *J* = 8.2 Hz, 1H), 7.83 (d, *J* = 8.1 Hz, 2H), 7.54 – 7.43 (m, 2H), 7.29 (d, *J* = 8.1 Hz, 2H), 7.08 (d, *J* = 7.6 Hz, 1H), 6.73 (d, *J* = 7.6 Hz, 1H), 3.70 (s, 2H), 2.95 (t, *J* = 7.9 Hz, 2H), 2.42 (s, 3H), 2.03 (q, *J* = 12.1, 8.4 Hz, 2H), 1.19 (s, 9H).

^{13}**C NMR** (101 MHz, $CDCl_3$) δ 155.69, 150.52, 145.83, 132.77, 131.29, 129.76, 129.64, 126.51, 125.81, 124.98, 124.92, 123.71, 122.55, 108.15, 83.48, 53.01, 29.61, 27.71, 27.06, 21.81.

ESI-HRMS: m/z calculated for $C_{25}H_{29}NNaO_6S$ [M+Na]$^+$: 494.1608; found: 494.1603.

Ethyl (*E*)-3-(2-hydroxy-4-methoxyphenyl)acrylate[25]

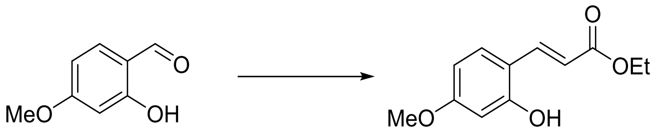

2-Hydroxy-4-methoxybenzaldehyde (1.80 g, 12.0 mmol) and ethyl 2-(triphenyl-phosphaneylidene) acetate (6.27 g, 18.0 mmol) in CH_2Cl_2 (15 mL) were stirred at r.t. overnight and monitored by TLC. Upon completion, the reaction mixture was concentrated *in vacuo* and purified by flash column chromatography (20% EtOAc/Hexane) afforded the title compound (2.73 g, quant.) as a colorless solid.

^{1}H NMR (400 MHz, $(CD_3)_2CO$) δ 9.13 (s, 1H), 7.93 (d, *J* = 16.1 Hz, 1H), 7.54 (d, *J* = 8.4 Hz, 1H), 6.53 – 6.50 (m, 2H), 6.48 (d, *J* = 16.1 Hz, 1H), 4.18 (q, *J* = 7.1 Hz, 2H), 3.79 (s, 3H), 1.27 (t, *J* = 7.1 Hz, 3H).

^{13}C NMR (101 MHz, $CDCl_3$) δ 170.0, 163.6, 158.9, 140.7, 131.2, 116.1, 115.6, 107.2, 102.3, 60.4, 55.7, 14.8.

Spectroscopic properties were consistent with the data available in the literature.[24]

Ethyl 3-(2-hydroxy-4-methoxyphenyl)propanoate[26]

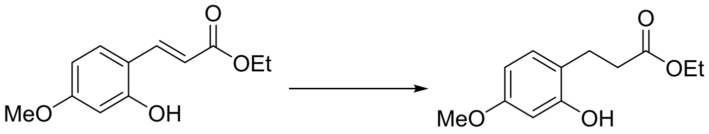

A solution of ethyl (*E*)-3-(2-hydroxy-4-methoxyphenyl)acrylate (2.22 g, 10.0 mmol) in EtOH (30 mL) was purged with argon before the addition of 10 wt. % Pd/C (5 mol%). The flask was fitted with a balloon of hydrogen and stirred at room temperature overnight and monitored by TLC. Upon completion, the reaction mixture was filtered over a bed of Celite® washing with the appropriate solvent and concentrated *in vacuo* to afford the title compound (2.21 g, 99%) as an off-white solid.

^{1}H NMR (400 MHz, $CDCl_3$) δ 7.50 (s, 1H), 6.97 (d, *J* = 8.3 Hz, 1H), 6.48 – 6.40 (m, 2H), 4.14 (q, *J* = 7.2 Hz, 2H), 3.75 (s, 3H), 2.79 – 2.89 (m, 2H), 2.63 – 2.73 (m, 2H), 1.24 (t, *J* = 7.2 Hz, 3H).

^{13}C NMR (101 MHz, $CDCl_3$) δ 176.1, 159.7, 155.4, 131.1, 119.7, 106.9, 102.9, 61.5, 55.4, 35.6, 24.1, 14.2.

Spectroscopic properties were consistent with the data available in the literature.[26]

Ethyl 3-(2-((*tert*-butyldimethylsilyl)oxy)-4-methoxyphenyl)propanoate

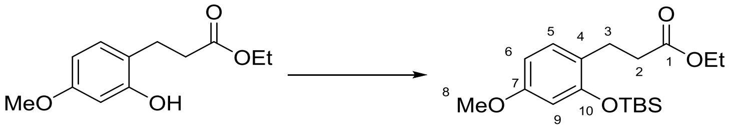

General procedure B: Ethyl 3-(2-hydroxy-4-methoxyphenyl)propanoate (1.68 g, 7.50 mmol), *tert*-butyldimethylsilyl chloride (1.36 g, 9.00 mmol), and imidazole (1.28 g, 18.75 mmol) in DMF (15 mL) were employed. Purificaition by flash column chromatography (20% EtOAc/Pentane) afforded the title compound (1.59 g, 63%) as a colorless oil.

IR (film) ν_{max}/cm^{-1}: 2955 (m), 2931 (m), 2858 (m), 1733 (s), 1611 (s), 1505 (s).

^{1}H NMR (400 MHz, $CDCl_3$) δ 7.04 (d, *J* = 8.3 Hz, 1H, C**5**-H), 6.44 (dd, *J* = 8.3, 2.5 Hz, 1H, C**6**-H), 6.38 (d, *J* = 2.5 Hz, 1H, C**9**-H), 4.12 (q, *J* = 7.1 Hz, 2H, OCH_2), 3.75 (s, 3H, C**8**-H_3), 2.84 (dd, *J* = 8.9, 7.0 Hz, 2H, C**3**-H_2), 2.54 (dd, *J* = 8.9, 7.0 Hz, 2H, C**2**-H_2), 1.23 (t, *J* = 7.1 Hz, 3H, CH_2CH_3), 1.02 (s, 9H, TBS $(CH_3)_3$), 0.25 (s, 6H, TBS $Si(CH_3)_2$).

^{13}C NMR (101 MHz, $CDCl_3$) δ 173.4 (C=O), 159.1 (C**7**), 154.5 (C**10**), 130.4 (C**5**), 123.7 (C**4**), 105.7 (C**6**/C**9**), 105.6 (C**6**/C**9**), 60.3 (OCH_2), 55.4 (C**8**), 34.9 (C**2**), 25.9 (TBS $(CH_3)_3$), 25.8 (C**3**), 18.3 (TBS $SiC(CH_3)_3$), 14.4 (CH_2CH_3), -4.0 (TBS $Si(CH_3)_2$).

ESI-HRMS: m/z calculated for $C_{18}H_{30}NaO_4Si[M+Na]^+$: 361.1806; found: 361.1820.

3-(2-((*tert*-Butyldimethylsilyl)oxy)-4-methoxyphenyl)propan-1-ol[26]

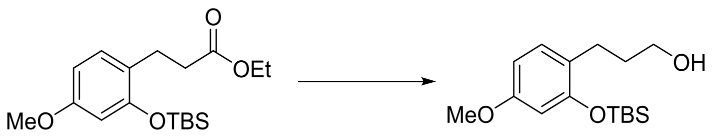

A solution of ethyl 3-(2-((*tert*-butyldimethylsilyl)oxy)-4-methoxyphenyl)propanoate (1.01 g, 3.0 mmol) in anhydrous THF (15 mL) was cooled to -78 °C before 2.0 eq. DIBALH (1 M in CH_2Cl_2) was added dropwise to maintain the temperature of the reaction mixture below -75 °C. The reaction was stirred at this temperature for 4 h and then warmed to 0 °C and stirred for an

additional 2 h. The reaction mixture was diluted with EtOAc (10 mL) and quenched with Rochelle's salt (10 mL). The mixture was filtered through Celite® and washed with EtOAc. The phases were separated and the aqueous phase extracted with EtOAc (10 mL). The combined organic extracts were dried over Na_2SO_4, filtered and concentrated *in vacuo*. Purification of the product by flash column chromatography (33% EtOAc/Hexane) afforded the title compound (0.44 g, 50%) as a colorless oil.

^{1}H NMR (400 MHz, $CDCl_3$) δ 7.03 (d, *J* = 8.4 Hz, 1H), 6.47 (dd, *J* = 8.4, 2.4 Hz, 1H), 6.39 (d, *J* = 2.4 Hz, 1H), 3.76 (s, 3H), 3.61 (t, *J* = 6.4 Hz, 2H), 2.62 (t, *J* = 7.2 Hz, 2H), 1.85 – 1.77 (m, 2H), 1.64 (br s, 1H), 1.01 (s, 9H), 0.25 (s, 6H).

^{13}C NMR (101 MHz, $CDCl_3$) δ 158.8, 154.4, 130.6, 124.7, 106.1, 105.7, 62.4, 55.4, 33.4, 25.9, 25.8, 18.4, -4.0.

Spectroscopic properties were consistent with the data available in the literature.[26]

tert-Butyl (3-(2-((tert-butyldimethylsilyl)oxy)-4-methoxyphenyl)propyl)(tosyloxy) carbamate

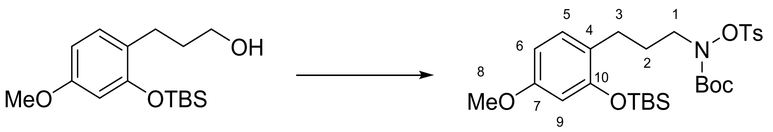

<u>General procedure D</u>: 3-(2-((*tert*-Butyldimethylsilyl)oxy)-4-methoxyphenyl)propan-1-ol (0.44 g, 1.50 mmol), PPh_3 (0.47 g, 1.80 mmol), DIAD (0.35 mL, 1.80 mmol) and TsONHBoc (0.52 g, 1.80 mmol) in anhydrous THF (6 mL) were employed. Purification by flash column chromatography (10% EtOAc/Hexane) afforded the title compound (0.82 g, 96%) as a colorless oil.

IR (film) ν_{max}/ cm^{-1}: 2955 (m), 2931 (m), 1720 (s), 1504 (s), 1160 (s).

^{1}H NMR (400 MHz, $CDCl_3$) δ 7.84 (d, *J* = 8.3 Hz, 2H, Ts ArC<u>H</u>), 7.31 (d, *J* = 8.3 Hz, 2H, Ts ArC<u>H</u>), 7.00 (d, *J* = 8.3 Hz, 1H, C**5**-<u>H</u>), 6.44 (dd, *J* = 8.3, 2.5 Hz, 1H, C**6**-<u>H</u>), 6.36 (d, *J* = 2.5 Hz, 1H, C**9**-<u>H</u>), 3.75 (s, 3H, C**8**-<u>H</u>$_3$), 3.71 – 3.49 (m, 2H, C**1**-<u>H</u>$_2$), 2.49 (t, *J* = 7.7 Hz, 2H, C**3**-

H_2), 2.43 (s, 3H, Ts CH_3), 1.95 – 1.81 (m, 2H, C**2**-H_2), 1.21 (s, 9H, Boc (CH_3)$_3$), 1.01 (s, 9H, TBS (CH_3)$_3$), 0.24 (s, 6H, TBS Si(CH_3)$_2$).

^{13}C NMR (101 MHz, $CDCl_3$) δ 158.8 (C**7**), 155.5 (C=O), 154.3 (C**10**), 145.6 (Ts ArC), 131.4 (Ts ArC), 130.3 (C**5**), 129.7 (2 × Ts ArCH), 129.6 (2 × Ts ArCH), 124.1 (C**4**), 105.7 (C**6**), 105.5 (C**9**), 83.1 (Boc C(CH_3)$_3$), 55.3 (C**8**), 52.8 (C**1**), 27.7 (Boc (CH_3)$_3$), 27.0 (C**3**), 26.2 (C**2**), 25.9 (TBS (CH_3)$_3$), 21.8 (Ts CH_3), 18.3 (TBS SiC(CH_3)$_3$), -4.1 (TBS Si(CH_3)$_2$).

ESI-HRMS: m/z calculated for $C_{28}H_{43}NNaO_7SSi[M+Na]^+$: 588.2422; found: 588.2426.

***tert*-Butyl (3-(2-hydroxy-4-methoxyphenyl)propyl)(tosyloxy)carbamate (5f)**

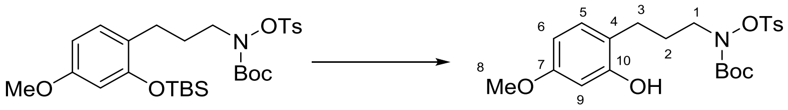

General procedure E: *tert*-Butyl (3-(2-((*tert*-butyldimethylsilyl)oxy)-4-methoxyphenyl) propyl) (tosyloxy)carbamate (0.56 g, 1.00 mmol) and 1.0 eq. 1:1 TBAF/AcOH solution (0.1 M in THF) in THF (20 mL) were employed. Purification by flash column chromatography (33% EtOAc/Hexane) afforded the title compound (0.30 g, 68%) as a colorless, viscous oil. **IR** (film) ν_{max}/ cm^{-1}: 3422 (br s), 2936 (m), 1720 (m), 1508 (m), 1368 (s).

^{1}H NMR (400 MHz, $CDCl_3$) δ 7.85 (d, *J* = 8.0 Hz, 2H, Ts ArCH), 7.33 (d, *J* = 8.0 Hz, 2H, Ts ArCH), 6.98 (d, *J* =8.3 Hz, 1H, C**5**-H), 6.42 (dd, *J* = 8.3, 2.4 Hz, 1H, C**6**-H), 6.36 (d, *J* = 2.4 Hz, 1H, C**9**-H), 5.33 (br s, 1H, OH), 3.75 (s, 3H, C**8**-H_3), 3.72 – 3.53 (m, 2H, C**1**-H_2), 2.54 (t, *J* = 7.7 Hz, 2H, C**3**-H_2), 2.44 (s, 3H, Ts CH_3), 1.98 – 1.85 (m, 2H, C**2**-H_2), 1.22 (s, 9H, Boc (CH_3)$_3$).

^{13}C NMR (101 MHz, $CDCl_3$) δ 159.3 (C**7**), 156.0 (C=O), 154.6 (C**10**), 145.9 (Ts ArC), 131.4 (Ts ArC), 130.7 (C**5**), 130.0 (2 × Ts ArCH), 129.7 (2 × Ts ArCH), 119.6 (C**4**), 106.1 (C**6**), 102.1 (C**9**), 83.6, 55.5 (C**8**), 53.0 (C**1**), 27.8 (Boc (CH_3)$_3$), 26.6 (C**2**/C**3**), 26.5 (C**2**/C**3**), 21.8 (Ts CH_3).

ESI-HRMS: m/z calculated for $C_{22}H_{29}NNaO_7S[M+Na]^+$: 474.1557; found: 474.1560.

1,2-Dihydro-3*H*-naphtho[2,1-*b*]pyran-3-one[27]

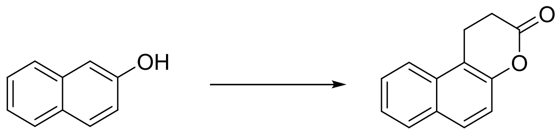

The compound was prepared according to a literature procedure.[27]

Spectroscopic properties were consistent with the data available in the literature.[28]

3-(2-((*tert*-Butyldimethylsilyl)oxy)naphthalen-1-yl)propanoic acid

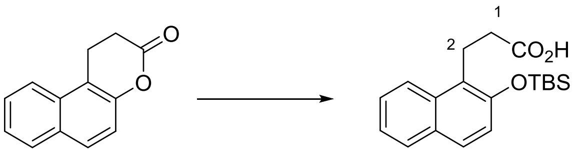

To a solution of 1,2-dihydro-3*H*-naphtho[2,1-*b*]pyran-3-one (7.90 g, 37.0 mmol) in THF (200 mL) was added 1 M LiOH (125 mL). After stirring at room temperature overnight the pH was adjusted to approx. 3 with 1M HCl. The product was extracted with EtOAc (50 mL), dried over Na_2SO_4, filtered and concentrated *in vacuo*. The crude product was dissolved in DMF (20 mL) and *tert*-butyldimethylsilyl chloride (12.2 g, 81.4 mmol) and imidazole (8.30 g, 122.1 mmol) were added at 0 °C. After being stirred at r.t. overnight the reaction was quenched by addition of H_2O and the product was extracted with hexane, dried over $MgSO_4$, filtered and concentrated *in vacuo*. To the crude product in MeOH (30 mL) and THF (30 mL) was added aq. K_2CO_3 (74.0 mmol, 10.2 g in 100 mL H_2O). After stirring for 5 h the reaction was quenched with aq. 1M HCl (100 mL) at 0 °C. The mixture was extracted with Et_2O, dried over Na_2SO_4, filtered and concentrated *in vacuo*. Purification of the product by flash column chromatography (20% EtOAc/Hexane) afforded the title compound (8.10 g, 66%) as a pale yellow solid.

m.p.: 94 - 96 °C (EtOAc/hexane).

IR (solid) ν_{max}/ cm^{-1}: 2957 (m), 2928 (m), 2900 (m), 2857 (m), 1699 (s).

^{1}H NMR (400 MHz, $CDCl_3$) δ 7.97 (d, *J* = 8.5 Hz, 1H, ArCH), 7.79 (d, *J* = 7.8 Hz, 1H, ArCH), 7.65 (d, *J* = 8.5 Hz, 1H, ArCH), 7.51 (t, *J* = 7.7 Hz, 1H, ArCH), 7.36 (t, *J* = 7.5 Hz, 1H, ArCH), 7.11 (d, *J* = 8.9 Hz, 1H, ArCH), 3.43 (t, *J* = 8.5 Hz, 2H, C**2**-H_2), 2.67 (t, *J* = 8.5 Hz, 2H, C**3**-H_2), 1.08 (s, 9H, TBS $(CH_3)_3$), 0.31 (s, 6H, TBS $Si(CH_3)_2$).

^{13}C NMR (101 MHz, $CDCl_3$) δ 179.3 (C=O), 150.8 (ArC), 133.0 (ArC), 129.5 (ArC), 128.6 (ArCH), 127.9 (ArCH), 126.5 (ArCH), 123.4 (ArCH), 123.1 (ArC), 122.8 (ArCH), 120.2 (ArCH), 33.9 (C**1**), 25.8 (TBS (CH_3)$_3$), 21.0 (C**2**), 18.3 (TBS SiC(CH_3)$_3$), 3.9 (TBS Si(CH_3)$_2$).
ESI-HRMS: m/z calculated for $C_{19}H_{26}NaO_3Si[M+Na]^+$: 353.154342; found: 353.155141.

3-(2-(*tert*-Butyldimethylsilyloxy)naphthalen-1-yl)propan-1-ol

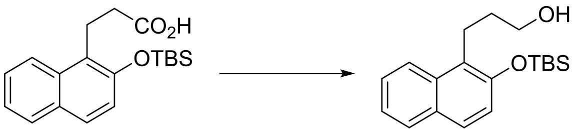

To a solution of carboxylic acid 3-(2-((*tert*-butyldimethylsilyl)oxy)naphthalen-1-yl)propanoic acid (1.65 g, 5.00 mmol) and Et_3N (0.70 mL, 5.00 mmol), in THF (50 mL) at -5 °C was added a solution of ethyl chloroformate (0.54 g, 5.00 mmol) in THF (5 mL/mmol) dropwise maintaining a temperature below 0 °C. The reaction was stirred at the same temperature for 1 h and filtered to remove the white precipitate that formed, washing with THF (10 mL). The filtrate was added dropwise to a solution of $NaBH_4$ (0.47 g, 12.5 mmol) in H_2O (20 mL) at -5 °C. The reaction was stirred at r.t. overnight and monitored by TLC. Upon completion, the reaction was acidified to approx. pH 3 with aq. 1 M HCl. The layers were separated and the aqueous layer extracted with Et_2O (3 × 10 mL). The combined organic extracts were washed with aq. 1 M NaOH (10 mL) and H_2O (10 mL), dried over $MgSO_4$, filtered and concentrated *in vacuo*. Purification of the product by flash column chromatography (20% EtOAc/Hexane) afforded the title compound (1.19 g, 75%) as a colorless oil.
IR (film) ν_{max} / cm^{-1}: 3336 (m, br), 2953 (m), 2929 (m), 2882 (m), 2857 (m), 1622 (m), 1594 (m), 1465 (m), 1264 (m), 1241 (s).
^{1}H NMR (400 MHz, $CDCl_3$) δ 8.00 (d, *J* = 8.1 Hz, 1H), 7.79 (d, *J* = 8.4 Hz, 1H), 7.63 (d, *J* = 8.6 Hz, 1H), 7.48 (t, *J* = 7.8 Hz, 1H), 7.35 (t, *J* = 7.2 Hz, 1H), 7.12 (d, *J* = 9.0 Hz, 1H), 3.63 (t, *J* = 5.9 Hz, 2H), 3.20 (t, *J* = 6.7 Hz, 2H), 2.53 (br s, 1H), 1.95 (p, *J* = 7.4 Hz, 2H), 1.10 (s, 9H), 0.31 (s, 6H).
^{13}C NMR (101 MHz, $CDCl_3$) δ 150.6, 133.4, 129.9, 128.6, 127.4, 126.3, 124.7, 123.6, 123.5, 120.6, 62.2, 32.5, 26.0, 21.5, 18.5, -3.8.
Spectroscopic properties were consistent with the data available in the literature.[28]

tert-Butyl (3-(2-((tert-butyldimethylsilyl)oxy)naphthalen-1-yl)propyl) (tosyloxy) carbamate

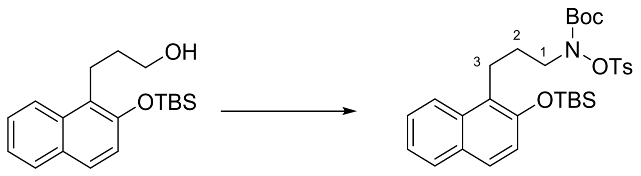

To a solution of 3-(2-(*tert*-butyldimethylsilyloxy)naphthalen-1-yl)propan-1-ol (0.63 g, 2.00 mmol), *tert*-butyl (tosyloxy)carbamate (0.56 g, 3.00 mmol) and PPh_3 (1.05 g, 4.00 mmol) in anhydrous PhMe:THF (3:1, 8 mL/mmol) at 0 °C was added a solution of DIAD (0.78 mL, 4.00 mmol) in anhydrous PhMe (2 mL/mmol) dropwise. The reaction was stirred at room temperature until completion by TLC analysis (4 h). The reaction mixture was concentrated *in vacuo.* Purification of the product by flash column chromatography (20–25% EtOAc/Hexane) afforded the title compound (0.74 g, 63%) as a colorless solid.

m.p.: 79 - 80 °C (EtOAc/hexane).

IR (film) ν_{max} / cm^{-1}: 2961 (m), 2927 (m), 2857 (m), 1709 (s), 1596 (m), 1466 (m), 1368 (s), 1240 (s), 1174 (s), 1164 (s), 1153 (s), 1087 (m).

^{1}H NMR (400 MHz, $CDCl_3$) δ 7.86 (d, *J* = 8.6 Hz, 1H, ArC<u>H</u>), 7.82 (d, *J* = 8.3 Hz, 2H, ArC<u>H</u>), 7.75 (d, *J* = 8.2 Hz, 1H, ArC<u>H</u>), 7.59 (d, *J* = 8.8 Hz, 1H, ArC<u>H</u>), 7.47 – 7.43 (m,1H, ArC<u>H</u>), 7.33 (ddd, *J* = 8.1, 6.8, 1.1 Hz, 1H, ArC<u>H</u>), 7.27 (d, *J* = 8.2 Hz, 2H, ArC<u>H</u>), 7.05 (d, *J* = 8.8 Hz, 1H, ArC<u>H</u>), 3.71 (br s, 2H, C**1**-$\underline{H}_2$), 3.02 (t, *J* = 7.9 Hz, 2H, C**3**-$\underline{H}_2$), 2.42 (s, 3H, Ts C$\underline{H}_3$), 1.98 – 1.89 (m, 2H, C**2**-$\underline{H}_2$), 1.16 (s, 9H, Boc (C$\underline{H}_3$)$_3$), 1.07 (s, 9H, TBS (C$\underline{H}_3$)$_3$), 0.27 (s, 6H, Si(C$\underline{H}_3$)$_2$).

^{13}C NMR (101 MHz, $CDCl_3$) δ 155.5 (C=O), 150.6 (Ar<u>C</u>), 145.6 (Ts Ar<u>C</u>), 133.3 (Ar<u>C</u>), 131.5 (Ts Ar<u>C</u>), 129.7 (2 × Ts Ar<u>C</u>H), 129.6 (Ar<u>C</u>), 129.5 (2 × Ts ArC<u>H</u>), 128.6 (Ar<u>C</u>H), 127.5 (Ar<u>C</u>H), 126.4 (Ar<u>C</u>H), 124.2 (Ar<u>C</u>), 123.3 (Ar<u>C</u>H), 123.2 (Ar<u>C</u>H), 120.3 (Ar<u>C</u>H), 83.1 (Boc <u>C</u>(CH_3)$_3$, 53.1 (C**1**), 27.6 (Boc (<u>C</u>H_3)$_3$), 26.1 (C**2**), 26.0 (TBS (C$\underline{H}_3$)$_3$), 22.7 (C**3**), 21.8 (Ts <u>C</u>H_3), 18.4 (TBS <u>C</u>(CH_3)$_3$), -3.8 (TBS Si(<u>C</u>H_3)$_2$).

ESI-HRMS: m/z calculated for $C_{31}H_{43}NNaO_6SSi$[M+Na]$^+$: 608.2473; found: 608.2456.

tert-Butyl (3-(2-hydroxynaphthalen-1-yl)propyl)(tosyloxy)carbamate (5g)

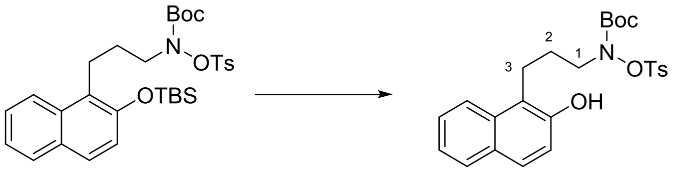

General procedure E: *tert*-Butyl (3-(2-((*tert*-butyldimethylsilyl)oxy)naphthalen-1-yl) propyl)(tosyloxy) carbamate (0.16 g, 0.279 mmol) and 1.0 eq., 1:1 TBAF/HOAc solution (0.1 M in THF) in THF were employed. Purification of the product by flash column chromatography (33% EtOAc/Hexane) afforded the title compound (0.11 g, 84%) as a pale yellow solid.

m.p.: 53 - 55 °C (EtOAc/hexane).

IR (film) ν_{max} / cm^{-1}: 3359 (m, br), 2931 (m), 1721 (s), 1369 (s), 1191 (s), 1178 (s), 1154 (s).

^{1}H NMR (400 MHz, $CDCl_3$) δ 7.88 – 7.83 (m, 3H, Ts ArCH, ArCH), 7.77 (d, *J* = 8.1 Hz, 1H, ArCH), 7.62 (d, *J* = 8.8 Hz, 1H, ArCH), 7.47 (ddd, *J* = 8.3, 6.9, 1.4 Hz, 1H, ArCH), 7.35 – 7.29 (m, 3H, Ts ArCH, ArCH), 7.07 (d, *J* = 8.8 Hz, 1H, ArCH), 5.77 (br s, 1H, OH), 3.79 – 3.70 (m, 2H, C**1**-H_2), 3.07 (t, *J* = 7.8 Hz, 2H, C**3**-H_2), 2.43 (s, 3H, Ts CH_3), 2.08 – 1.97 (m, 2H, C**2**-H_2), 1.20 (s, 9H, Boc $(CH_3)_3$).

^{13}C NMR (101 MHz, $CDCl_3$) δ 156.1 (C=O), 151.1 (ArC), 145.9 (Ts ArC), 133.2 (ArC), 131.3 (Ts ArC), 129.7 (2 × Ts ArCH), 129.7 (2 × Ts ArCH), 129.5 (ArC), 128.8 (ArCH), 128.1 (ArCH), 126.6 (ArCH), 123.1 (ArCH), 122.8 (ArCH), 118.8 (ArC), 118.1 (ArCH), 83.6 (Boc $C(CH_3)_3$), 53.2 (C**1**), 27.7 (Boc $(CH_3)_3$), 26.4 (C**2**), 22.2 (C**3**) 21.8 (Ts CH_3).

ESI-HRMS: m/z calculated for $C_{25}H_{29}NNaO_6S[M+Na]^+$: 494.1608; found: 494.1598.

2-(Cinnamyloxy)naphthalene[29]

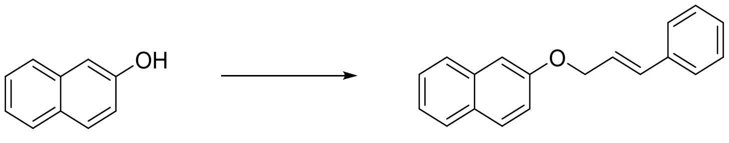

The title compound was prepred according to a literature procedure.[29]

Spectroscopic properties were consistent with the data available in the literature.[29]

1-(1-Phenylallyl)naphthalen-2-ol[30]

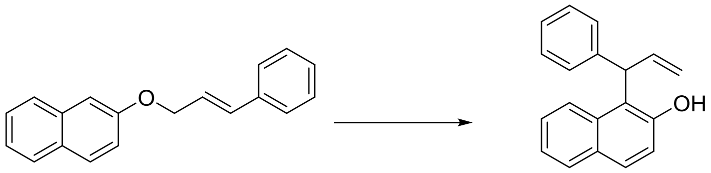

The title compound was prepared according to a literature procedure.[29]

Spectroscopic properties were consistent with the data available in the literature.[30]

tert-Butyldimethyl((1-(1-phenylallyl)naphthalen-2-yl)oxy)silane

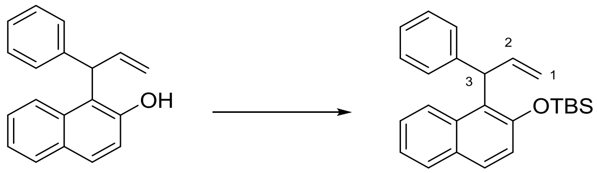

To a solution of 1-(1-phenylallyl)naphthalen-2-ol (1.40 g, 5.30 mmol), in DMF (10 mL) was added *tert*-butyldimethylsilyl chloride (0.97 g, 6.45 mmol) and imidazole (0.91 g, 13.4 mmol) and the reaction mixture was stirred at room temperature overnight until completion by TLC analysis. The reaction was quenched with water (50 mL) and extracted with CH_2Cl_2 (3 × 20 mL). The organic phase was washed with brine (20 mL), dried over Na_2SO_4, filtered and concentrated *in vacuo*. Purification of the product by flash column chromatography (4% EtOAc/Hexane) afforded the title compound (1.37 g, 69%) as a pale-yellow oil.

IR (film) ν_{max}/cm^{-1}: 2955 (m), 2928 (m), 1622 (m), 1586 (m), 1463 (m), 1253 (m), 1236 (m).

^{1}H NMR (400 MHz, $CDCl_3$) δ 7.75 (d, *J* = 7.9 Hz, 1H, ArC<u>H</u>), 7.70 – 7.63 (m, 2H, ArC<u>H</u>), 7.25 – 7.13 (m, 8H, ArC<u>H</u>), 6.64 (ddd, *J* = 17.3, 10.1, 7.5 Hz, 1H, C**2**-<u>H</u>), 5.91 (d, *J* = 7.6 Hz, 1H, C**3**-<u>H</u>) 5.28 – 5.16 (m, 2H, C**1**-<u>H</u>$_2$), 0.99 (s, 9H, TBS (C<u>H</u>$_3$)$_3$), 0.23 (d, *J* = 7.3 Hz, 6H, TBS Si(C<u>H</u>$_3$)$_2$).

^{13}C NMR (101 MHz, $CDCl_3$) δ 151.4 (Ar<u>C</u>), 143.7 (Ar<u>C</u>), 138.9 (C**2**), 132.9 (Ar<u>C</u>), 130.4 (Ar<u>C</u>), 128.7 (Ar<u>C</u>H), 128.6 (Ar<u>C</u>H), 128.3 (2 × Ar<u>C</u>H), 127.6 (2 × Ar<u>C</u>H), 126.0 (Ar<u>C</u>), 125.7 (2 × Ar<u>C</u>H), 125.5 (Ar<u>C</u>H), 123.2 (Ar<u>C</u>H), 120.6 (Ar<u>C</u>H), 117.5 (C**1**), 45.4 (C**3**), 26.0 (TBS (<u>C</u>H$_3$)$_3$), 18.5 (TBS Si<u>C</u>(CH$_3$)$_3$), -3.6 (TBS Si(<u>C</u>H$_3$)$_2$).

ESI-HRMS: m/z calculated for $C_{25}H_{31}NaOSi[M+Na]^+$: 397.1958; found: 397.1972.

3-(2-((_tert_-Butyldimethylsilyl)oxy)naphthalen-1-yl)-3-phenylpropan-1-ol

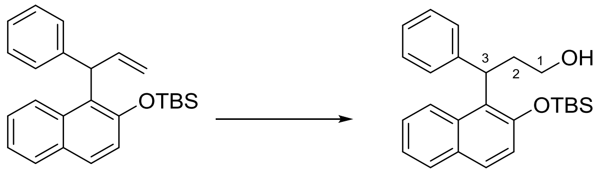

This compound was prepared according to a literature procedure.[28]

IR (film) ν_{max}/cm^{-1}: 3447 (m, br), 2952 (m), 2929 (m), 2857 (m), 1595 (m), 1463 (m), 1237 (m).

^{1}H NMR (400 MHz, $CDCl_3$) δ 7.78 – 7.74 (m, 1H), 7.76-7.68 (m, 1H), 7.66 – 7.57 (m, 1H), 7.32 – 7.24 (m, 5H), 7.21 – 7.15 (m, 3H), 5.37 (dd, *J* = 11.4, 4.7 Hz, 1H, C**3**-H), 3.62 – 3.53 (m, 1H, C**1**-H), 3.37 – 3.27 (m, 1H, C**1**-H'), 2.87 – 2.79 (m, 1H, C**2**-H), 2.55 – 2.46 (m, 1H, C**2**-H'), 2.28 (br s, 1H, OH), 1.03 (s, 9H, TBS $(CH_3)_3$), 0.37 (s, 3H, TBS Si(CH_3)), 0.25 (s, 3H, TBS Si(CH_3)).

^{13}C NMR (101 MHz, $CDCl_3$) δ 151.6 (ArC), 144.6 (ArC), 132.8 (ArC), 130.8 (ArC), 128.5 (ArC), 128.8 (ArCH), 128.7 (ArCH), 128.3 (2 × ArCH), 127.1 (2 × ArCH), 125.7 (ArCH), 125.7 (ArCH), 125.5 (ArCH), 123.5 (ArCH), 120.2 (ArCH) 61.5 (C**1**), 36.4 (C**3**), 34.9 (C**2**) 25.9 (TBS $(CH_3)_3$), 18.4 (TBS Si$C(CH_3)_3$), -3.44 (TBS Si(CH_3)), -3.83 (TBS Si(CH_3)).

ESI-HRMS: m/z calculated for $C_{25}H_{33}NaO_2Si$[M+Na]$^+$: 415.2064; found: 415.2061.

***tert*-Butyl (3-(2-hydroxynaphthalen-1-yl)-3-phenylpropyl)(tosyloxy)carbamate (5h)**

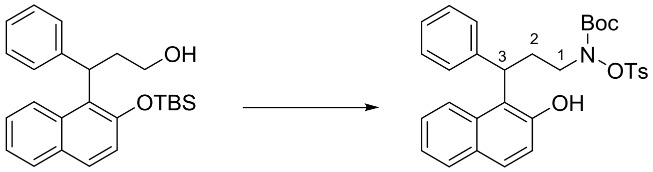

General procedure D and E: 3-(2-((*tert*-Butyldimethylsilyl)oxy)naphthalen-1-yl)-3-phenylpropan-1-ol (0.27 g, 0.69 mmol), PPh_3 (0.22 g, 0.832 mmol), DIAD (0.16 mL, 0.83 mmol) and TsONHBoc (0.24 g, 0.83 mmol) in anhydrous THF (4 mL) were employed. The product was purified by flash column chromatography (10% EtOAc/hexane) to afford the desired product (0.40 g) which could not be obtained cleanly so was used crude in the next step using 1:1 TBAF/AcOH solution (0.1 M in THF, 0.60 mmol) in THF (12 mL). Purification of the product by flash column chromatography (33% EtOAc/Hexane) afforded the title compound (0.26 g, 71% over 2 steps) as an off-white solid.

m.p.: 75 - 78 °C (EtOAc/hexane).

IR (film) ν_{max}/ cm^{-1}: 3410 (m, br), 2978 (m), 1721 (m), 1373 (s).

^{1}H NMR (400 MHz, $CDCl_3$) δ 7.99 – 7.89 (m, 1H, ArC<u>H</u>), 7.79 – 7.70 (m, 3H, ArC<u>H</u>), 7.66 (d, *J* = 8.8 Hz, 1H, ArC<u>H</u>), 7.46 – 7.37 (m, 1H, ArC<u>H</u>), 7.35 – 7.26 (m, 9H, ArC<u>H</u>), 7.00 (d, *J* = 8.8 Hz, 1H, ArC<u>H</u>), 5.11 (br s, 1H, O<u>H</u>), 4.99 (t, *J* = 8.1 Hz, 1H, C**3**-<u>H</u>), 3.73 – 3.61 (m, 1H, C**1**-<u>H</u>), 3.42 – 3.15 (m, 1H, C**1**-<u>H</u>'), 2.84 – 2.51 (m, 2H, C**2**-$\underline{H_2}$), 2.38 (s, 3H, Ts C$\underline{H_3}$), 1.14 (s, 9H, Boc (C$\underline{H_3}$)$_3$).

^{13}C NMR (101 MHz, $CDCl_3$) δ 155.6 (C=O), 151.9 (Ar<u>C</u>), 145.7 (Ts Ar<u>C</u>), 142.9 (Ar<u>C</u>), 133.2 (Ar<u>C</u>), 131.3 (Ts Ar<u>C</u>), 129.8 (Ar<u>C</u>), 129.7 (2 x Ts Ar<u>C</u>H), 129.6 (2 × Ts ArC<u>H</u>), 129.2 (ArC<u>H</u>), 129.0 (ArC<u>H</u>), 128.9 (Ar<u>C</u>H), 127.5 (Ar<u>C</u>H), 126.8 (Ar<u>C</u>H), 126.6 (Ar<u>C</u>H), 123.3 (Ar<u>C</u>H), 121.3 (Ar<u>C</u>), 119.2 (Ar<u>C</u>H), 83.6 (Boc <u>C</u>(CH_3)$_3$), 52.3 (C**1**), 38.4 (C**3**), 28.0 (C**2**), 27.7 (Boc (<u>C</u>H_3)$_3$), 21.8 (Ts <u>C</u>H_3)$_3$.

ESI-HRMS: m/z calculated for $C_{31}H_{33}NNaO_6S$[M+Na]$^+$: 570.1920; found: 570.1912.

***tert*-Butyl 8-oxo-1-azaspiro[4.5]deca-6,9-diene-1-carboxylate (6a)**

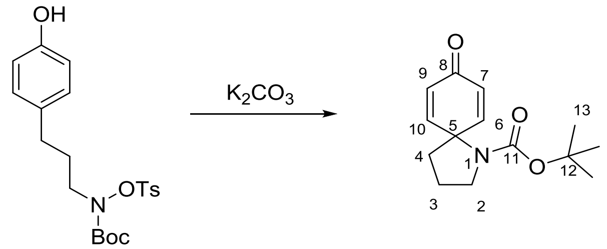

<u>**General procedure G**</u>**:** *tert*-Butyl (3-(4-hydroxyphenyl)propyl)(tosyloxy)carbamate **5a** (63.2 mg, 0.15 mmol) and K_2CO_3 (41.5 mg, 0.3 mmol) were employed. After stirring at 80 °C for 48 h, purification of the product by flash column chromatography (25% EtOAc/Hexane) afforded **6a** (31.9 mg, 85%) as a colorless solid.

m.p.: 128.7 - 130.4 °C (EtOAc/Hexane).

IR (film) ν_{max} / cm^{-1}: 2976 (w), 1689 (s), 1665 (s), 1389 (s), 1377 (s), 1365 (s), 1162 (s), 1129 (m), 847 (m).

^{1}H NMR (400 MHz, $CDCl_3$) δ 6.77 (d, *J* = 9.5 Hz, 2H, C**7**-H, C9-H), 6.17 (d, *J* = 9.4 Hz, 2H, C6-H, C**10**-H), 3.65 (t, *J* = 6.2 Hz, 2H, C**2**-H_2), 2.09 (d, *J* = 6.2 Hz, 2H, C**3**-H_2), 2.00 (dd, *J* = 12.5, 6.2 Hz, 2H, C**4**-H_2), 1.35 (s, 9H, Boc, C**13**-H_3 × 3).

^{13}C NMR (101 MHz, $CDCl_3$) δ 185.72 (C**8**), 153.78 (C**11**), 151.91 (C**6** + **C10**), 127.62 (C**7** + C**9**) , 80.66 (C**12**), 61.14 (C**5**), 47.78 (C**2**), 39.84 (C**3**), 28.13 (C**13**), 23.17 (C**4**).

ESI-HRMS: m/z calculated for $C_{14}H_{19}NNaO_3[M+Na]^+$: 272.1257; found: 272.1255.

***tert*-Butyl 7,9-dimethyl-8-oxo-1-azaspiro[4.5]deca-6,9-diene-1-carboxylate (6b)**

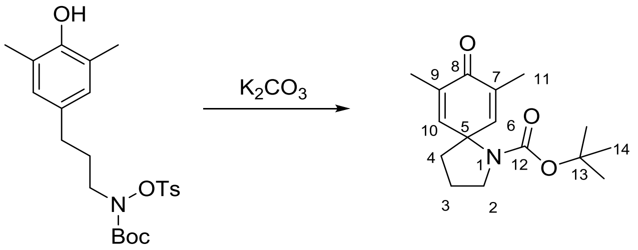

General procedure G: *tert*-Butyl (3-(4-hydroxy-3,5-dimethylphenyl)propyl)(tosyloxy) carbamate **5b** (35 mg, 0.078 mmol) and K_2CO_3 (21.6 mg, 0.156 mmol) were employed. After stirring at 80 °C for 42 h, purification of the product by flash column chromatography (10%–50% EtOAc/Hexane) afforded **6b** (17.4 mg, 81%) as a colorless solid.

m.p.: 90 - 92 °C (EtOAc/Hexane).

IR (film) ν_{max} / cm^{-1}: 2975 (m), 1690 (s), 1638 (s), 1388 (s), 1160 (m).

^{1}H NMR (400 MHz, $CDCl_3$) δ 6.53 (s, 2H, C**6**-H, C**10**-H), 3.63 (t, *J* = 6.6 Hz, 2H, C**2**-H_2), 2.04 (d, *J* = 6.4 Hz, 2H, C**3**-H_2), 2.01 – 1.91 (m, 2H, C**4**-H_2), 1.88 (s, 6H, C**11**-H_3 × 2), 1.24 (s, 9H, Boc, C**13**-H_3 × 3).

^{13}C NMR (101 MHz, $CDCl_3$) δ 187.11 (C**8**), 154.02 (C**12**), 146.83 (C**6** + **C10**), 133.51 (C**7** + C**9**), 79.71 (C**13**), 61.01 (C**5**), 47.64 (C**2**) , 39.55 (C**3**), 28.22 (C**14**), 23.12 (C**4**), 15.93 (C**11**) .

ESI-HRMS: m/z calculated for $C_{16}H_{23}NNaO_3[M+Na]^+$: 300.1570; found: 300.1575.

***tert*-Butyl 7-bromo-8-oxo-1-azaspiro[4.5]deca-6,9-diene-1-carboxylate (6c)**

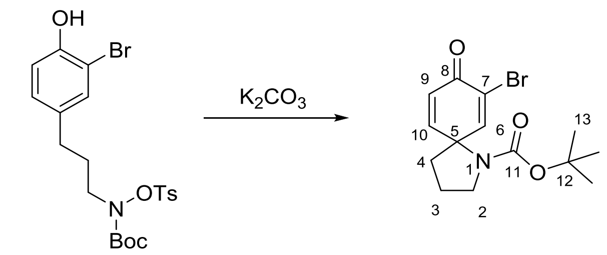

General procedure G: *tert*-Butyl (3-(3-bromo-4-hydroxyphenyl)propyl)(tosyloxy) carbamate **5c** (75.0 mg, 0.15 mmol) and K_2CO_3 (43.3 mg, 0.3 mmol) were employed. After stirring at 80 °C for 15 h, purification of the product by flash column chromatography (10%–50%) afforded **6c** (43.3 mg, 88%) as a colorless viscous oil.

IR (film) ν_{max} / cm^{-1}: 2974 (m), 1689 (s), 1666 (s), 1380 (s), 1365 (s), 1157 (m).

^{1}H NMR (400 MHz, $CDCl_3$) δ 7.22 (s, 1H, C**6**-H), 6.80 (d, *J* = 9.8 Hz, 1H, C**10**-H), 6.29 (d, *J* = 9.9 Hz, 1H, C**9**-H), 3.65 (d, *J* = 5.9 Hz, 2H, C**2**-H_2), 2.15 (dt, *J* = 19.6, 10.0 Hz, 2H, C**3**-H_2), 2.08 – 1.90 (m, 2H, C**4**-H_2), 1.46 – 1.18 (m, 9H, Boc, $C(CH_3)_3$).

^{13}C NMR (101 MHz, $CDCl_3$) δ 178.55 (C**8**), 153.44 (C**11**), 152.04 (C**10**), 151.73 (C**6**), 125.94 (C**7**), 123.21 (C**9**), 80.94 (C**12**), 63.66 (C**5**), 47.72 (C**2**), 39.19 (C**3**), 28.13 (C**13**), 23.13 (C**4**).

ESI-HRMS: m/z calculated for $C_{14}H_{18}BrNNaO_3[M+Na]^+$: 350.0362; found: 350.0368.

***tert*-Butyl 4-ethyl-8-oxo-1-azaspiro[4.5]deca-6,9-diene-1-carboxylate (6d)**

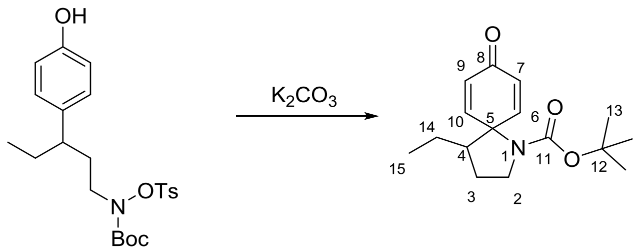

General procedure G: *tert*-Butyl (3-(4-hydroxyphenyl)pentyl)(tosyloxy)carbamate **5d** (67.4 mg, 0.15 mmol) and K_2CO_3 (41.5 mg, 0.3 mmol) were employed. After stirring at 60 °C for 48 h, purification of the product by flash column chromatography (25% EtOAc/Hexane) afforded **6d** (33.3 mg, 80%) as a colorless solid.

m.p.: 91.6 - 93.4 °C (EtOAc/Hexane).

IR (film) ν_{max} / cm^{-1}: 2967 (m), 1689 (s), 1665 (s), 1392 (s), 1378 (s), 1365 (s), 1175 (m), 1145 (s).

^{1}H NMR (400 MHz, $CDCl_3$) δ 6.77 – 6.57 (m, 2H, C**6**-H, C**10**-H), 6.23 (t, *J* = 8.5 Hz, 2H, C**7**-H, C**9**-H), 3.82 (t, *J* = 9.6 Hz, 1H, C**2**-H), 3.47 (td, *J* = 11.1, 6.5 Hz, 1H, C**2**-H'), 2.21 (dt, *J* = 12.3, 6.2 Hz, 1H, C**3**-H), 2.14 – 1.91 (m, 1H, C**3**-H'), 1.62 (dd, *J* = 20.7, 11.9 Hz, 1H, C**4**-H), 1.35 (s, 9H, Boc, C**13**-H_3 × 3), 1.26 – 1.17 (m, 1H, C**14**-H), 1.11 – 0.94 (m, 1H, C**14**-H'), 0.88 (t, *J* = 7.3 Hz, 3H, C**15**-H_3).

^{13}C NMR (101 MHz, $CDCl_3$) δ 186.19 (C**8**), 153.87 (C**11**), 153.31 (C**6**), 148.24 (C**10**), 129.25 (C**8**), 128.48 (C**9**), 80.68 (C**12**), 64.51 (C**5**), 52.48 (C**2**), 46.54 (C**4**), 28.76 (C**3**), 28.16 (C**13**), 22.10 (C**14**), 12.69 (C**15**).

ESI-HRMS: m/z calculated for $C_{16}H_{23}NNaO_3[M+Na]^+$: 300.1570; found: 300.1578.

The structure of this compound was determined unambiguously by X-ray crystallography.

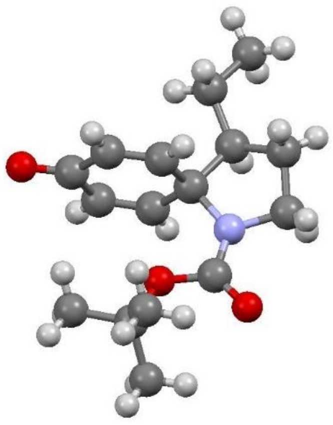

***tert*-Butyl 4-oxo-4*H*-spiro[naphthalene-1,2'-pyrrolidine]-1'-carboxylate (6e)**

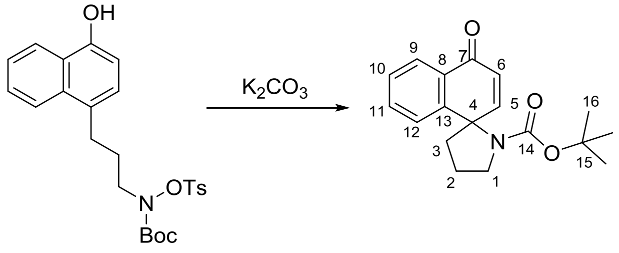

General procedure G: *tert*-Butyl (3-(4-hydroxynaphthalen-1-yl)propyl)(tosyloxy) carbamate **5e** (47.2 mg, 0.10 mmol) and K_2CO_3 (27.6 mg, 0.2 mmol) were employed. After stirring at 80

°C for 24 h, purification of the product by flash column chromatography (33% EtOAc/Hexane) afforded **6e** (25.5 mg, 85%, 1:8 mixture of rotamers *A:B*) as a colorless solid.

m.p.: 141 - 143 °C (EtOAc/Hexane).

IR (film) ν_{max} / cm^{-1}: 2974 (m), 1686 (s), 1663 (s), 1382 (s), 1364 (m), 1303 (m), 1159 (m), 1137 (m), 932 (m), 765 (s).

^{1}H NMR (400 MHz, $CDCl_3$) δ 8.09 (d, *J* = 7.7 Hz, 1H, C**9**-H), 7.54 (t, *J* = 7.9 Hz, 1H, C**11**-H), 7.38 (q, *J* = 7.5 Hz, 2H, C**10**-H, C**12**-H), 6.95 (d, *J* = 10.2 Hz, 1H, C**6**-H), 6.32 (d, *J* = 10.4 Hz, 1H, C**5**-H), 3.94 – 3.69 (m, 2H, C**1**-H_2), 2.26 (qd, *J* = 13.1, 6.8 Hz, 2H, C**3**-H_2), 2.20 – 1.92 (m, 2H, C**2**-H_2), 1.37 (s, 1H, C**16**-H, *A*), 0.94 (s, 8H, C**16**-H, *B*).

^{13}C NMR (101 MHz, $CDCl_3$) δ 184.61 (C**7**), 153.99 (C**6**, *A*), 153.47 (C**14**), 152.98 (C**6**, *B*), 148.77 (C**13**, *A* + *B*), 132.94 (C**11**, *B*), 132.64 (C**11**, *A*), 130.91 (C**8**, *A* + *B*), 127.35 (C**10**, *B*), 127.19 (C**10**, *A*), 126.88 (C**9**, *B*), 126.52 (C**9**, *A*), 126.30 (C**5**, *A* + *B*), 124.92 (C**12**, *B*), 124.05 (C**12**, *A*), 80.14 (C**15**, *A* + *B*), 62.59 (C**4**, *A* + *B*), 48.93 (C**1**, *A*), 48.53 (C**1**, *B*), 43.92 (C**3**, *B*), 43.48 (C**3**, *A*), 28.49 (C**16**, *B*), 27.83 (C**16**, *A*), 23.19 (C**2**, *B*), 22.96 (C**2**, *A*).

ESI-HRMS: m/z calculated for $C_{18}H_{21}NNaO_3[M+Na]^+$: 322.1414; found: 322.1429.

***tert*-Butyl 8-methoxy-10-oxo-1-azaspiro[4.5]deca-6,8-diene-1-carboxylate (6f)**

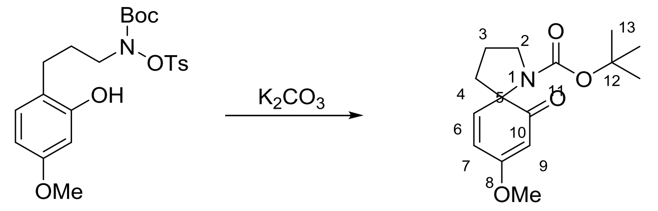

General procedure G: *tert*-Butyl (3-(2-hydroxy-4-methoxyphenyl)propyl)(tosyloxy) carbamate **5f** (45.2 mg, 0.10 mmol), K_2CO_3 (20.7 mg, 0.15 mmol) and 1,2-Dichloroethene (1.5 mL) were employed. After stirring at 60 °C for 48 h, purification of the product by flash column chromatography (33% EtOAc/Hexane) afforded **6f** (17.7 mg, 63%, 2:3 mixture of rotamers *A:B*) as a colorless solid.

m.p.: 71 - 73 ºC (EtOAc/Hexane).

IR (film) ν_{max} / cm^{-1}: 2971 (m), 1693 (s), 1656 (s), 1576 (s), 1386 (s), 1233 (m), 1168 (s), 1143 (s), 821 (m).

^{1}H NMR (400 MHz, $CDCl_3$) δ 6.38 (d, *J* = 10.0 Hz, 0.4H, C**6**-H, *A*), 6.34 (d, *J* = 10.0 Hz, 0.6H, C**6**-H, *B*), 6.08 (dd, *J* = 10.0, 2.4 Hz, 0.4H, C**7**-H, *A*), 6.02 (dd, *J* = 10.0, 2.4 Hz, 0.6H, C**7**-H, *B*), 5.48 (d, *J* = 2.4 Hz, 0.4H, C**9**-H, *A*), 5.44 (d, *J* = 2.4 Hz, 0.6H, C**9**-H, *B*), 3.75 (d, *J* = 2.6 Hz, 4H, CH_3O, C**2**-H, *A* + *B*), 3.63 – 3.50 (m, 1H, C**2**-H', *A* + *B*), 2.21 – 2.01 (m, 2H, C**4**-H, *A* + *B*; C**3**-H, *A* + *B*), 1.98 – 1.77 (m, 2H, C**4**-H', *A* + *B*; C**3**-H', *A* + *B*), 1.42 (s, 3.55H, Boc $(CH_3)_3$, *A*), 1.28 (s, 5.45H, Boc $(CH_3)_3$, *B*).

^{13}C NMR (101 MHz, $CDCl_3$) δ 200.49 (C**10**, *A*), 200.45 (C**10**, *A*), 170.51 (C**8**, *A*), 170.41 (C**8**, *B*), 153.94 (C**11**, *A*), 153.60 (C**10**, *B*), 146.45 (C**6**, *B*), 145.83 (C**6**, *A*), 120.76 (C**7**, *A*), 120.01 (C**7**, *B*), 99.20 (C**9**, *A*), 98.87 (C**9**, *B*), 80.35 (C**12**, *B*), 80.17 (C**12**, *A*), 68.46 (C**5**, *A*), 68.11 (C**5**, *B*), 55.99 (CH_3O, *B*), 55.87 (CH_3O, *A*), 48.63 (C**2**, *A*), 48.25 (C**2**, *B*), 39.33 (C**4**, *B*), 38.35 (C**4**, *A*), 28.60 (C**13**, *A*), 28.16 (C**13**, *B*), 22.93 (C**3**, *B*), 22.31 (C**3**, *A*).

ESI-HRMS: m/z calculated for $C_{15}H_{21}NNaO_4[M+Na]^+$: 302.1363; found: 302.1375.

tert-Butyl 2-oxo-2*H*-spiro[naphthalene-1,2'-pyrrolidine]-1'-carboxylate (6g)

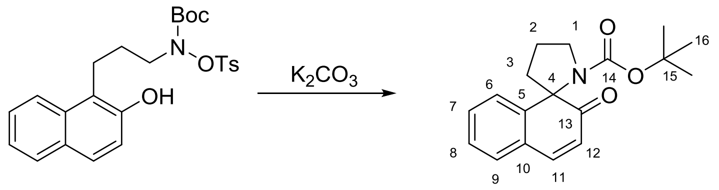

<u>General procedure G</u>: *tert*-Butyl (3-(2-hydroxynaphthalen-1-yl)propyl) (tosyloxy)carbamate **5g** (70.7 mg, 0.15 mmol) and K_2CO_3 (41.5 mg, 0.3 mmol) were employed. After stirring at 80 °C for 24 h, purification of the product by flash column chromatography (33%–50% EtOAc/Hexane) afforded **6g** (35.7 mg, 80%, 2:7 mixture of rotamers *A:B*) as a colorless solid.

m.p.: 86 - 87 °C (EtOAc/Hexane).

IR (film) ν_{max} / cm^{-1}: 2974 (m), 1693 (s), 1674 (s), 1385 (s), 1365 (m), 1159 (s).

^{1}H NMR (400 MHz, $CDCl_3$) δ 7.44 – 7.31 (m, 3H, C**11**-H, C**6**-H, C**7**-H), 7.31 – 7.20 (m, 2H, C**8**-H, C**9**-H), 6.15 (d *J* = 8.0 Hz, 1H, C**12**-H), 4.05 – 3.85 (m, 1H, C**1**-H), 3.84 – 3.71 (m, 1H, C**12**-H'), 2.40 – 2.19 (m, 1H, C**3**-H), 2.09 (tdd, *J* = 10.6, 9.1, 4.2 Hz, 1H, C**3**-H'), 2.00 – 1.76 (m, 2H, C**4**-H_2), 1.37 (s, 2H, C**15**-H, *A*), 0.98 (s, 7H, C**15**-H, *B*).

^{13}C NMR (101 MHz, $CDCl_3$) δ 200.76 (C**13**, *B*), 200.67 (C**13**, *A*), 153.59 (C**14**, *A*), 153.25 (C**14**, *B*), 147.32 (C**5**, *B*), 146.43 (C**5**, *A*), 145.03 (C**11**, *A*), 144.83 (C**11**, *B*), 130.33 (C**7**, *A* +

B), 129.64 (C**10**, *B*), 129.25 (C**10**, *A*), [129.09, 127.26, 127.24, 124.90, 124.49, (C**6**, *A* + *B*) , (C**8**, *A* + *B*), (C**9**, *A* + *B*)], 124.35 (C**12**, *A*), 124.07 (C**12**, *B*), 80.04 (C**15**, *A*), 79.88 (C**15**, *B*), 71.88 (C**4**, *A*), 71.82 (C**4**, *B*), 49.34 (C**1**, *B*), 48.86 (C**1**, *A*), 42.19 (C**3**, *B*), 41.76 (C**3**, *A*), 28.50 (C**16**, *A*), 27.86 (C**16**, *B*), 22.56 (C**2**, *A*), 21.68 (C**2**, *B*).

ESI-HRMS: m/z calculated for $C_{18}H_{21}NNaO_3[M+Na]^+$: 322.1414; found: 322.1425.

tert-Butyl (1*R,3'*R**)-2-oxo-3'-phenyl-2*H*-spiro[naphthalene-1,2'-pyrrolidine]-1'-carboxylate (6h)**

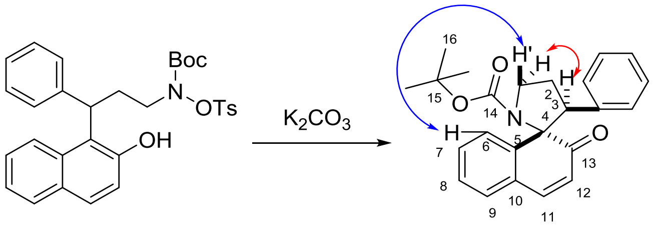

<u>**General procedure G**</u>**:** *tert*-Butyl (3-(2-hydroxynaphthalen-1-yl)-3- phenylpropyl) (tosyloxy) carbamate **5h** (82.1 mg, 0.15 mmol) and K_2CO_3 (41.5 mg, 0.3 mmol) were employed. After stirring at 80 °C for 48 h, purification of the product by flash column chromatography (20%–50% EtOAc/Hexane) afforded **6h** (34.3 mg, 61%, *d.r.* = 1:3, *A:B*) as a colorless solid.

m.p.: 166 - 168 °C (EtOAc/Hexane).

IR (film) ν_{max} / cm^{-1}: 2974 (m), 1694 (s), 1673 (s), 1391 (s), 1365 (s), 1170 (m), 1140 (m), 764 (m).

^{1}H NMR (400 MHz, $CDCl_3$) δ 7.39 – 7.35 (m, 2H, C**6**-<u>H</u>, C**7**-<u>H</u>), 7.21 – 7.14 (m, 1H, C**8**-<u>H</u>), 7.07 – 6.97 (m, 1H, ArC<u>H</u>), 6.98 – 6.86 (m, 4H, C**9**-<u>H</u>, C**11**-<u>H</u>, ArC<u>H</u>$_2$), 6.62 – 6.49 (m, 2H, ArC<u>H</u>$_2$), 6.13 (d, *J* = 9.9 Hz, 0.24H, C**12**-<u>H</u>, *A*), 6.09 (d, *J* = 9.9 Hz, 0.70H, C**12**-<u>H</u>, *B*), 4.17 (dd, *J* = 10.3, 8.3 Hz, 0.74H, C**1**-<u>H</u>, *B*), 4.06 (dd, *J* = 10.2, 8.4 Hz, 0.25H, C**1**-<u>H</u>, *A*), 3.86 (tdd, *J* = 10.8, 6.1, 4.2 Hz, 1H, C**1**-<u>H</u>’, *A* + *B*), 3.64 (dd, *J* = 13.1, 6.0 Hz, 0.75H, C**3**-<u>H</u>, *B*), 3.60 – 3.54 (m, 0.25H, C**3**-<u>H</u>, *A*), 2.71 – 2.42 (m, 1H, C**2**-<u>H</u>, *A* + *B*), 2.22 – 2.04 (m, 1H, C**2**-<u>H</u>’, *A* + *B*), 1.43 (s, 2H, C**1**-<u>H</u>, *A*), 1.04 (s, 7H, C**1**-<u>H</u>, *B*).

^{13}C NMR (101 MHz, $CDCl_3$) δ 201.35 (C**13**, *B*), 200.76 (C**13**, *A*), 153.34 (C**14**, *A*), 153.20 (C**14**, *B*), 145.50 (Ar<u>C</u>, *A*), 145.27 (Ar<u>C</u>, *B*), 143.64 (C**10**, *B*), 142.74 (C**10**, *A*), 135.21 (C**5**, *A* + *B*), 131.02 (C**17**, *A*), 130.72 (C**17**, *B*), [129.45, 129.29, 128.96, 128.93, 128.90, 128.15, 127.85, 127.61, 127.54, 127.42, 127.39, 127.23, 127.20, 126.31, 125.95, (Ar<u>C</u>, *A* + *B*)] 125.16

(C**12**, *A*), 124.89 (C**12**, *B*), 80.30 (C**15**, *A*) , 80.23 (C**15**, *B*), 75.64 (C**4**, *B*) , 75.51 (C**4**, *A*), 61.04 (C**3**, *B*), 60.27 (C**3**, *A*), 47.79 (C**1**, *A*), 47.21 (C**1**, *B*), 29.18 (C**2**, *A*), 28.62 (C**16**, *A*), 28.37 (C**2**, *B*), 28.00 (C**16**, *B*).

ESI-HRMS: m/z calculated for $C_{24}H_{25}NNaO_3[M+Na]^+$: 398.1727; found: 398.1741.

*The relative stereochemistry of this compound was corroborated by nOe experiments (as indicated on the compound structure). nOes were observed from C**3**-H to C**1**-H and from C**6**-H to C**1**-H'.*

tert-Butyl (3*S,3'*R**)-3'-phenylspiro[indoline-3,2'-pyrrolidine]-1'-carboxylate (7)**

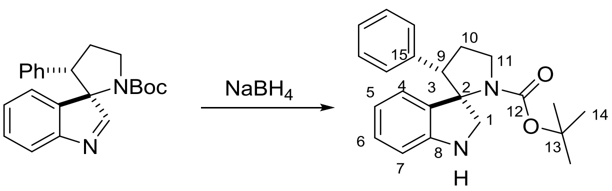

To a solution of *tert*-butyl 3'-phenylspiro[indole-3,2'-pyrrolidine]-1'-carboxylate **4u** (40 mg, 0.115 mmol) in EtOH (2.0 mL) at 0 ˚C was added $NaBH_4$ (26 mg, 0.69 mmol). The resulted mixture was stirred at 0 ˚C to room temperature for 16.5 h. The reaction mixture was then concentrated *in vacuo* to give the crude product, which was purified by flash column chromatography (33% EtOAc/Hexane) to afford **7** (40.4 mg, 100%, 1:2 mixture of rotamers *A:B*) as a white powder.

IR (film) ν_{max} / cm^{-1}: 3418 (br, s), 2926 (s), 1691 (s), 1514 (m), 1456 (m), 1366 (m), 1252 (m), 1169 (s), 743 (s).

^{1}H NMR (500 MHz, CD_2Cl_2) δ 7.39 – 7.26 (m, 0.74H, ArCH, *A*) 7.22 – 7.12 (m, 4H, ArCH, *B*), 7.10 – 7.03 (m, 1H, C**6**-H), 6.90 – 6.77 (m, 3H, ArCH), 6.44 (m, 1H, C**7**-H), 3.75 (dd, *J* = 10.4, 8.0 Hz, 1H, C**11**-H), 3.59 (td, *J* = 11.0, 5.9 Hz, 1H, C**11**-H'), 3.56 – 3.41 (m, 2H, C**1**-H, C**9**-H), 3.25 (d *J* = 10.0 Hz, 0.63H, C**1**-H', *B*), 3.20 (d *J* = 10.0 Hz, 0.21H, C**1**-H', *A*), 2.35 – 2.21 (m, 1H, C**10**-H), 2.16 – 2.02 (m, 1H, C**10**-H'), 1.41 (d, *J* = 2.5 Hz, 3H, C**14**-H, *A*), 1.06 (s, 6H, C**14**-H, *B*).

^{13}C NMR (126 MHz, CD_2Cl_2) δ 154.26 (C**12**, *B*), 154.22 (C**12**, *A*), 152.50 (C**8**, *B*), 152.35 (C**8**, *A*), 137.80 (C**3**, *B*), 137.17 (C**3**, *A*), 133.76 (C**15**), [128.92, 128.81 , 128.59, 128.35 128.33 , 127.51 , 126.68, 122.92, 122.73, 119.69, 119.65, 119.07, 119.00, 111.66, (ArC (*A* +*B*) × 4); C**4**

(*A* +*B*), C**5** (*A* +*B*), C**6** (*A* +*B*)], 110.55 (C**7**, *A*), 110.42 (C**7**, *B*), 79.47 (C**14**), 73.81 (C**2**, *A*), 57.24 (C**9**, *B*), 56.86 (C**9**, *A*), 53.53 (C**1**), 47.08 (C**11**, *A*), 46.51 (C**11**, *B*), 28.69 (C**14**, *A*), 28.19 (C**14**, *B*), 27.55 (C**10**, *A*), 27.01 (C**10**, *B*).

ESI-HRMS: m/z calculated for $C_{22}H_{26}N_2NaO_2[M+Na]^+$: 373.1886; found: 373.1896.

tert-Butyl (2*S,3*S**,3'*R**)-2-allyl-3'-phenylspiro[indoline-3,2'-pyrrolidine]-1'-carboxylate (8)**

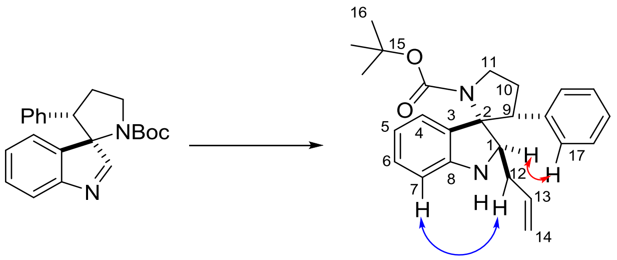

To a solution of *tert*-butyl 3'-phenylspiro[indole-3,2'-pyrrolidine]-1'-carboxylate **4u** (40 mg, 0.115 mmol) in THF (1.2 mL) at 0 ˚C was added allylmagnesium chloride in THF (2.0 M, 0.173 mmol) dropwise. The reaction mixture was stirred for 15 hours during which time it warmed to room temperature. The reaction was quenched by addition of saturated aq. NH_4Cl (3 mL) and extracted with Et_2O (3 × 10 mL). The combined organic phases were washed with brine (3 mL), dried (Na_2SO_4) and concentrated *in vacuo*. The residue was then purified by flash column chromatography (33% EtOAc/Hexane) to afford **8** (35 mg, 78%, d.r. = 6:1) as a colorless oil.

IR (film) ν_{max} / cm^{-1}: 3356 (br, m), 2974 (m), 1680 (s), 1609 (m), 1489 (m), 1391 (s), 1149 (m), 744 (m).

^{1}H NMR (400 MHz, $CDCl_3$) δ 7.21 – 7.10 (m, 4H, C**7**-H, ArCH × 3), 7.06 (td, *J* = 7.7, 1.2 Hz, 1H, C**6**-H), 6.85 – 6.75 (m, 3H, C**5**-H, ArCH × 2), 6.47 (d, *J* = 7.8 Hz, 0.13H), 6.41 (d, *J* = 7.8 Hz, 0.83H, C**7**-H), 5.84 – 5.63 (m, 1H, C**13**-H), 5.09 – 4.98 (m, 2H, C**14**-H_2), 3.80 – 3.64 (m, 3H, C**1**-H, C**11**-H_2), 3.38 (dd, *J* = 9.8, 4.4 Hz, 1H, C**9**-H), 2.42 – 2.20 (m, 3H, C**10**-H, C**12**-H_2), 2.16 (d, *J* = 6.1 Hz, 1H, C**10**-H'), 1.41 (s, 1H, C**17**-H, *A*), 1.10 (s, 8H, C**17**-H, *B*).

^{13}C NMR (101 MHz, $CDCl_3$) δ 154.59 (C**15**), 150.74 (C**8**), 137.45 (C**3**), 136.23 (C**13**), 131.54 (ArC), 128.67 (ArC), 128.40 (ArC), 128.20 (C**6**), 127.21 (ArC), 123.34 (C**5**), 118.33 (ArC), 117.06 (C**14**), 109.77 (C**7**), 79.63 (C**16**), 74.94 (C**2**), 61.02 (C**9**), 57.03 (C**1**), 46.49 (C**11**), 38.51 (C**12**), 28.01 (C**17**), 25.17 (C**10**).

ESI-HRMS: m/z calculated for $C_{25}H_{30}N_2NaO_2[M+Na]^+$: 413.2199; found: 413.2219.

*The relative stereochemistry of this compound was corroborated by nOe experiments (as indicated on the compound structure). nOes were observed from C**12**-H to C**7**-H and from C**1**-H to C**17**-H.*

tert-Butyl 4-oxo-2-vinyl-3,4-dihydro-2H-spiro[naphthalene-1,2'-pyrrolidine]-1'-carboxylate (9)

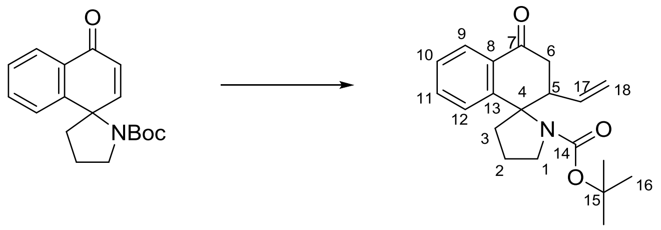

A solution of *tert*-butyl 4-oxo-4*H*-spiro[naphthalene-1,2'-pyrrolidine]-1'-carboxylate **6e** (30 mg, 0.1 mmol) in dry THF (0.3 mL) was added through a syring to a solution of CuBr·Me_2S (6.2 mg, 0.03 mmol) and Me_2S (0.05 mL) in the same solvent (0.2 mL) and cooled to –40 °C. Then a 1 M solution of vinylmagnesium bromide in THF (0.3 mL, 0.3 mmol) was added dropwise with stirring whilst keeping the reaction temperature below –40 °C. The reaction mixture (which changed from brown to orange during the addition) was stirred at –40 °C for 3 h and then decomposed in ice and aqueous hydrochloride (ca. 1 M, 2.0 mL), extracted with diethyl ether (3 × 10 mL) and dried. The crude product was left to evaporation to dryness under reduced pressure was subjected to flash column chromatography (33% EtOAc/Hexane) to afford **9** (25.3 mg, 77%, d.r. = 5:1, *B:A*).

m.p.: 91 - 93 °C (EtOAc/Hexane).

IR (film) ν_{max} / cm^{-1}: 2974 (m), 1678 (s), 1383 (s), 1364 (s), 1151 (s), 764 (s).

^{1}H NMR (400 MHz, $CDCl_3$) δ 7.95 (dd, *J* = 7.8, 1.4 Hz, 1H, C**9**-H), 7.54 (t, *J* = 8.0 Hz, 1H, C**11**-H), 7.34 – 7.24 (m, 2H, C**10**-H, C**12**-H), 6.05 – 5.76 (m, 1H, C**17**-H), 5.17 – 5.04 (m, 1H, C**18**-H), 5.00 (d, *J* = 10.1 Hz, 1H, C**18**-H'), 3.87 (dt, *J* = 11.0, 8.3 Hz, 1H, C**1**-H), 3.69 (dt, *J* = 11.5, 5.8 Hz, 1H, C**1**-H'), 2.89 (dt, *J* = 16.8, 5.4 Hz, 3H, C**5**-H, C**3**-H_2), 2.56 (dt, *J* = 12.8, 5.0 Hz, 1H, C**6**-H), 2.20 (dt, *J* = 12.7, 9.1 Hz, 1H, C**6**-H'), 2.06 (td, *J* = 8.3, 4.6 Hz, 2H, C**2**-H_2), 1.35 (s, 2.67H, rotamer of Boc), 1.25 (s, 0.67H), 1.03 (s, 1H, rotamer of Boc), 0.98 (s, 6.21H).

^{13}C NMR (101 MHz, $CDCl_3$) δ 197.74 (C**7**, *A*), 197.20 (C**7**, *B*), 153.87 (C**14**, *A* + *B*), 147.76 (C**13**, *A* + *B*), 138.02 (C**17**, *A* + *B*), 133.92 (C**11**, *A* + *B*), 131.83 (C**8**, *A* + *B*), [128.11, 127.27, 126.89 (C**10**, *A* + *B or A/B*), (C**12**, *A* + *B, or A/B*)], 126.09 (C**9**, *A* + *B*), 116.63 (C**18**, *B*), 112.81 (C**18**, *A*), 80.07 (C**15**, *A* + *B*), 66.30 (C**15**, *A* + *B*), 50.05 (C**5**, *A* + *B*), 49.03 (C**1**, *A* + *B*), 44.74 (C**6**, *A* + *B*) , 42.80 (C**3**, *A* + *B*), 28.59, 28.50, 28.12, 27.93 (C**16**, *A* + *B and* rotamer), 22.45 (C**2**, *A* + *B*).

ESI-HRMS: m/z calculated for $C_{20}H_{26}NO_3[M+H]^+$: 328.1907; found: 328.1919.

tert-Butyl (3-(1*H*-indol-3-yl)propyl)carbamate (10)

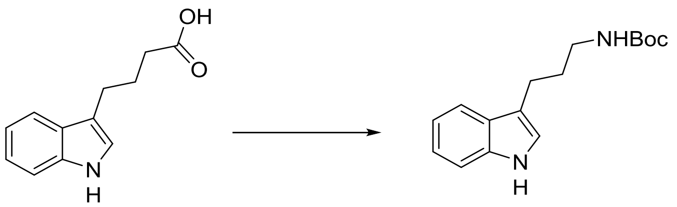

To a solution of 4-(1*H*-indol-3-yl)butanoic acid (1.26 g, 6.21 mmol) and Et_3N (0.9 mL, 6.5 mmol) in anhydrous *t*-BuOH (10 mL) was added diphenylphosphoryl azide (1.31 g, 6.5 mmol) dropwise. The reaction was heated at 80 °C for 48 h before cooling to r.t. and concentrating *in vacuo*. Et_2O (50 mL) and water (100 mL) were added. The organic portion was isolated and the aqueous layer was extracted with Et_2O (2 × 30 mL). The organics extracts were combined, washed with sat. aq. $NaHCO_3$ (50 mL) and brine (50 mL), dried over Na_2SO_4 and concentrated *in vacuo*. The residue was purified by flash column chromatography (10%–25% EtOAc/Hexane) to yield the title compound (0.66 g, 39%) as a colorless solid.

m.p.: 90 - 92 °C (EtOAc/Hexane).

IR (film) ν_{max} / cm^{-1}: 3392 (br, m), 3331 (br, m), 1667(s), 1281 (m), 1172 (m), 733 9s).

^{1}H NMR (400 MHz, $CDCl_3$) δ 8.03 (s, 1H), 7.59 (dt, *J* = 7.9, 1.0 Hz, 1H), 7.35 (dt, *J* = 8.2, 0.9 Hz, 1H), 7.19 (ddd, *J* = 8.2, 7.1, 1.2 Hz, 1H), 7.11 (ddd, *J* = 8.0, 7.0, 1.1 Hz, 1H), 6.99 (d, *J* = 2.3 Hz, 1H), 4.57 (s, 1H), 3.21 (q, *J* = 6.7 Hz, 2H), 2.91 – 2.69 (m, 2H), 1.90 (p, *J* = 7.3 Hz, 2H), 1.46 (s, 9H).

^{13}C NMR (101 MHz, $CDCl_3$) δ 156.20, 136.51, 127.50, 122.05, 121.51, 119.29, 118.91, 115.76, 111.25, 79.25, 77.48, 77.36, 77.16, 76.84, 40.51, 30.43, 28.58, 22.46.

ESI-HRMS: m/z calculated for $C_{16}H_{22}N_2NaO_2$ $[M+Na]^+$: 297.1573; found: 297.1574.

Benzyl (*E*)-hex-4-en-1-yl((pentafluorobenzoyl)oxy)carbamate (11a)

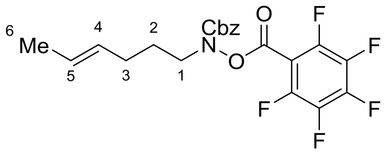

General procedure C: (*E*)-Hex-4-en-1-ol (0.33 mL, 2.80 mmol), benzyl (perfluorobenzoyl) oxycarbamate **2c** (1.22 g, 3.36 mmol), PPh_3 (1.10 g, 4.20 mmol), DIAD (0.83 mL, 4.20 mmol), THF (3.0 mL) and toluene (12.0 mL) were employed. Purification of the product by flash column chromatography (67% Hexane/PhMe) afforded **11a** (1.04 g, 75 %) as a crystalline colorless oil.

IR (film) ν_{max} / cm^{-1}: 2940 (br, m), 1786 (s), 1730 (s), 1524 (s), 1500 (s), 1327 (s), 1175 (s), 1003 (s).

^{1}H NMR (400 MHz, $CDCl_3$) δ 7.39 – 7.30 (m, 5H, ArCH), 5.50 – 5.33 (m, 2H, C**4**-H and C**5**-H), 5.22 (s, 2H, OCH_2Ph), 3.75 (t, *J* = 7.0 Hz, 2H, C**1**-H_2), 2.06 (td, *J* = 7.5, 6.5 Hz, 2H, C**3**-H_2), 1.71 (tt, *J* = 7.5, 7.0 Hz, 2H, C**2**-H_2), 1.63 (dq, *J* = 6.0, 1.0 Hz, 3H, C**6**-H_3).

^{13}C NMR (101 MHz, $CDCl_3$) δ 155.5 (Cbz C=O), 135.4 (ArC), 129.7 (C**4**), 128.7 (ArCH), 128.6 (ArCH), 128.2 (ArCH), 126.3 (C**5**), 68.9 (OCH_2Ph), 50.8 (C**1**), 29.4 (C**3**), 26.8 (C**2**), 18.0 (C**6**).

The signals corresponding to the pentafluorobenzoyl group could not be resolved due to their weak intensity.

^{19}F NMR (377 MHz, $CDCl_3$) δ -135.8 – -136.1 (2F, m), -145.9 – -146.1 (1F, m), -159.2 – -159.4 (2F, m).

ESI-HRMS: m/z calculated for $C_{21}H_{18}F_5NNaO_4$ $[M+Na]^+$: 466.1048; found: 466.1050.

***tert*-Butyl (*E*)-hex-4-en-1-yl(tosyloxy)carbamate (11b)**

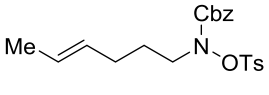

<u>General procedure D</u>: (*E*)-hex-4-en-1-ol (0.34 g, 2.85 mmol), PPh_3 (1.12 g, 2.40 mmol), DIAD (0.47 mL, 4.27 mmol) and methyl (tosyloxy)carbamate (0.59 g, 4.27 mmol) in anhydrous THF (25 mL) were employed. Purification by flash column chromatography (toluene) afforded the title compound **11b** (1.11 g, 96 %) as a colorless oil.

IR (film) ν_{max} / cm^{-1}: 2957 (br, w), 1756 (m), 1725 (s), 1383 (s), 1191 (s), 1179 (s), 750 (s).

^{1}H NMR (400 MHz, $CDCl_3$) δ 7.81 – 7.70 (m, 2H), 7.33 (q, *J* = 2.9 Hz, 3H), 7.17 (dd, *J* = 7.0, 4.7 Hz, 4H), 5.48 – 5.26 (m, 2H), 4.91 (s, 2H), 3.64 (s, 2H), 2.39 (s, 3H), 1.98 – 1.89 (m, 2H), 1.70 (q, *J* = 7.3 Hz, 2H), 1.62 (dd, *J* = 6.0, 1.4 Hz, 3H).

^{13}C NMR (101 MHz, $CDCl_3$) δ 156.70, 145.82, 135.02, 131.04, 129.74, 129.61, 129.56, 128.56, 128.54, 128.22, 126.05, 68.70, 52.85, 29.54, 25.74, 21.92, 18.01.

ESI-HRMS: m/z calculated for $C_{21}H_{25}NNaO_5S$ $[M+Na]^+$: 426.1346; found: 426.1337.

Methyl (3-(4-((*tert*-butyldimethylsilyl)oxy)phenyl)propyl(tosyloxy)carbamate

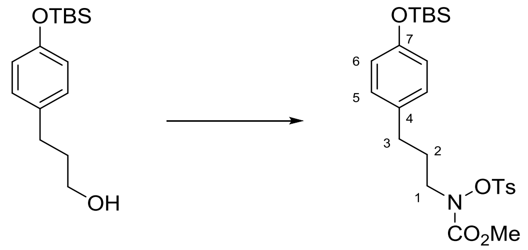

<u>General procedure D</u>: 3-(4-((*tert*-Butyldimethylsilyl)oxy)phenyl)propan-1-ol (0.53 g, 2.00 mmol), PPh_3 (0.63 g, 2.40 mmol), DIAD (0.47 mL, 2.40 mmol) and methyl (tosyloxy)carbamate (0.59 g, 2.40 mmol) in anhydrous THF (8 mL) were employed. Purification by flash column chromatography (10% EtOAc/Hexane) afforded the title compound (0.93 g, 94%) as a colorless oil.

IR (film) ν_{max} / cm^{-1}: 2955 (m), 2930 (m), 2858 (m), 1728 (s), 1509 (s), 1253 (s).

^{1}H NMR (400 MHz, $CDCl_3$) δ 7.83 (2H, d, *J* = 8.1 Hz, Ts ArC<u>H</u>), 7.33 (2H, d, *J* = 8.1 Hz, Ts ArC<u>H</u>), 6.99 (2H, d, *J* = 8.1 Hz, C**5**-<u>H</u>), 6.74 (2H, d, *J* = 8.1 Hz, C**6**-<u>H</u>), 3.58 (2H, app. br s, C**1**-$\underline{H}_2$), 3.47 (3H, s, OC$\underline{H}_3$), 2.51 (2H, t, *J* = 7.8 Hz, C**3**-$\underline{H}_2$), 2.45 (3H, s, Ts C$\underline{H}_3$), 1.94 - 1.85 (2H, m, C**2**-$\underline{H}_2$), 0.98 (9H, s, TBS (C$\underline{H}_3$)$_3$), 0.18 (6H, s, TBS Si($\underline{C}H_3$)$_2$).

^{13}C NMR (101 MHz, $CDCl_3$) δ 157.2 (C=O), 153.9 (C**7**), 146.0 (Ts Ar<u>C</u>), 133.6 (C**4**), 131.2 (Ts Ar<u>C</u>), 129.7 (2 × Ts Ar<u>C</u>H), 129.6 (2 × Ts Ar<u>C</u>H), 129.2 (C**5**), 120.1 (C**6**), 53.7 (O<u>C</u>H$_3$),

52.7 (C**1**), 32.0 (C**3**), 27.7 (C**2**), 25.8 (TBS (CH_3)$_3$), 21.9 (Ts CH_3), 18.3 (TBS SiC(CH_3)$_3$), -4.3 (TBS Si(CH_3)$_2$).

ESI-HRMS: m/z calculated for $C_{24}H_{35}NNaO_6SSi$ $[M+Na]^+$: 516.1847; found: 516.1851.

Methyl (3-(4-hydroxyphenyl)propyl)(tosyloxy)carbamate (5a-Me)

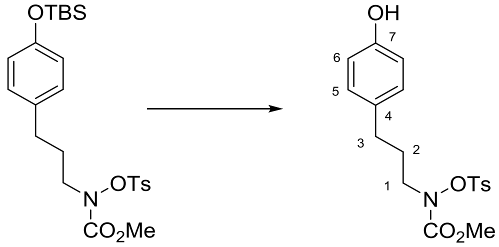

General procedure E: Methyl (3-(4-((*tert*-butyldimethylsilyl)oxy)phenyl)propyl)(tosyloxy) carbamate (490 mg, 1.00 mmol) and 1.1 eq. 1:1 TBAF/AcOH solution (0.1 M in THF, 11 mL, 1.1 mmol) in THF (20 mL) were employed. Purification by flash column chromatography (20–33% EtOAc/Hexane) afforded **5a-Me** (289 mg, 76%) as a viscous, colorless oil.

IR (film) ν_{max} / cm^{-1}: 3431 (m, br), 3023 (m), 2956 (m), 1726 (m), 1514 (m), 1175 (s).

^{1}H NMR (400 MHz, $CDCl_3$) δ 7.82 (2H, d, *J* = 8.2 Hz, Ts ArCH), 7.33 (2H, d, *J* = 8.2 Hz, Ts ArCH), 6.99 (2H, d, *J* = 8.1 Hz, C**5**-H), 6.74 (2H, d, *J* = 8.1 Hz, C**6**-H), 4.88 (1H, s, OH), 3.59 (2H, br s, C**1**-H_2), 2.51 (2H, t, *J* = 7.8 Hz, C**3**-H_2), 2.45 (3H, s, OCH_3), 1.95 – 1.85 (2H, m, C**2**-H_2).

^{13}C NMR (101 MHz, $CDCl_3$) δ 157.3 (C=O), 154.0 (C**7**), 146.1 (Ts ArC), 133.0 (C**4**), 131.1 (Ts ArC), 129.7 (2 × Ts ArCH), 129.6 (2 × Ts ArCH), 129.5 (C**5**), 115.4 (C**6**), 53.8 (OCH_3), 52.7 (C**1**), 31.9 (C**3**), 27.8 (C**2**), 21.9 (Ts CH_3).

ESI-HRMS: m/z calculated for $C_{18}H_{21}NNaO_6S$ $[M+Na]^+$: 402.0982; found: 402.0984.

Methyl 8-oxo-1-azaspiro[4.5]deca-6,9-diene-1-carboxylate (6a-Me)

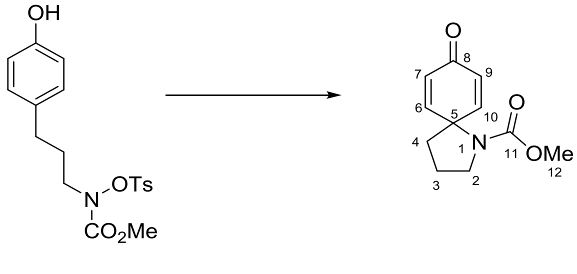

General procedure G: Methyl (3-(4-hydroxyphenyl)propyl)(tosyloxy)carbamate **5a-Me** (56.9 mg, 0.15 mmol) and K_2CO_3 (41.5 mg, 0.3 mmol) were employed. After stirring at 60 °C for 9 h, purification of the product by flash column chromatography (33%–50% EtOAc/Hexane) afforded **6a-Me** (29 mg, 93%, 1:1 mixture of rotamers *A:B*) as a colorless solid.

m.p.: 115.7 – 117.4 °C (EtOAc/Hexane).

IR (film) ν_{max} / cm^{-1}: 2956 (w), 1698 (s), 1666 (s), 1628 (m), 1447 (s), 1373 (s), 1191 (m), 860 (m)

^{1}H NMR (500 MHz, $CDCl_3$) δ 6.82 – 6.71 (m, 2H, C**6**-H, C**10**-H), 6.20 (dd, *J* = 33.9, 9.8 Hz, 2H, C**7**-H, C**9**-H), 3.80 – 3.46 (m, 5H, C**12**-H_3, C**2**-H), 2.19 – 1.93 (m, 4H, C**3**-H, C**4**-H).

^{13}C NMR (126 MHz, $CDCl_3$) δ 185.57 (C**8**, *A*) , 185.32 (C**8**, *B*), 155.17 (C**11**, *A*), 154.29 (C**11**, *B*), 151.17 (C**6** + **C10**, *A*), 150.67(C**6** + **C10**, *B*), 128.41 (C**7** + **C9**, *A*), 127.78 (C**7** + **C9**, *B*), 61.67 (C**5**, *A*), 61.19 (C**5**, *B*), 52.52 (C**12**, A + *B*), 48.43 (C**2**, *A*), 47.68 (C**2**, *B*), 40.05 (C**4**, *A*), 39.13 (C**4**, *B*), 23.94 (C**3**, *A*), 23.27 (C**3**, *B*).

MALDI-HRMS: m/z calculated for $C_{11}H_{13}NNaO_3$ $[M+Na]^+$: 230.0788; found: 230.0797.

3. Control experiments

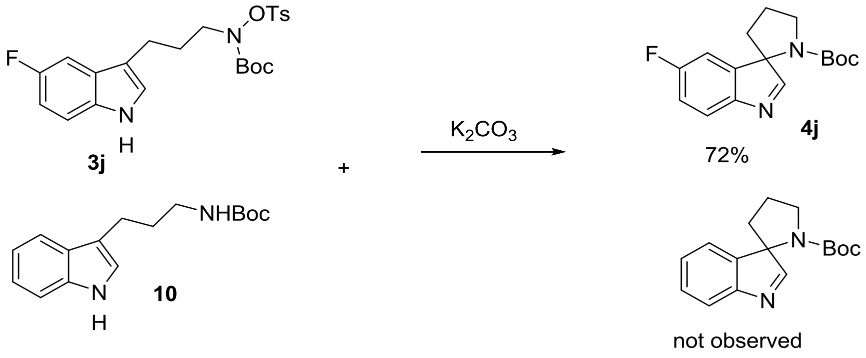

A flame-dried re-sealable tube, fitted with a rubber septum, was charged with fresh prepared *tert*-butyl (3-(5-fluoro-1*H*-indol-3-yl)propyl)(tosyloxy)carbamate (69.4 mg, 0.15 mmol), *tert*-butyl (3-(1*H*-indol-3-yl)propyl)carbamate (41.2 mg, 0.15 mmol), K_2CO_3 (41.5 mg, 0.3 mmol). The tube was purged with nitrogen, anhydrous 2,2,2-Trifluoroethanol (TFE) (2.3 mL) was added via syringe. The tube was sealed and heated at 60 °C for 24 h. The reaction mixture was concentrated *in vacuo* and the crude mixture was purified by flash column chromatography (16-33% EtOAc/Hexane) to afford **4j** (31.4 mg, 72%), **10** (40.3 mg, 98%).

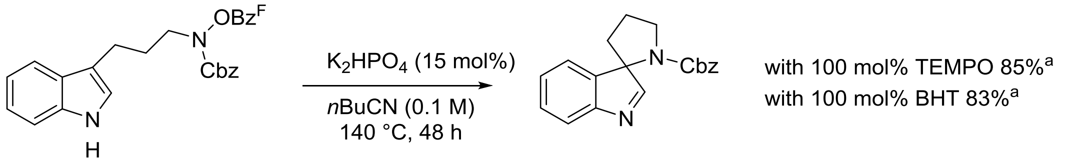

[a]In situ yield determined by ^{1}H NMR against 1,3,5-trimethoxybenzene internal standard.

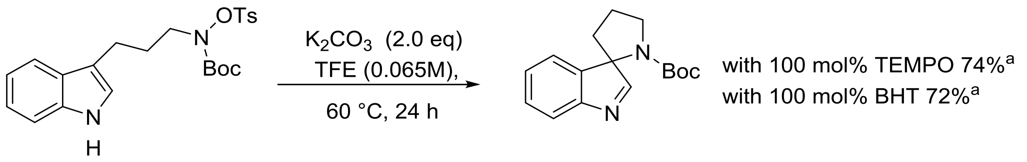

[a]Isolated yield.

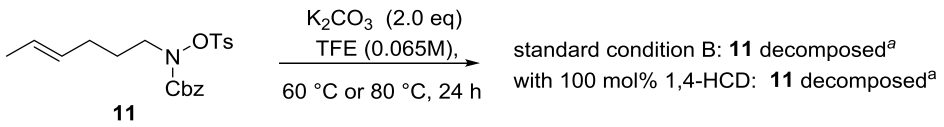

[a]No pyrrolidine product was observed by NMR, GC-MS.

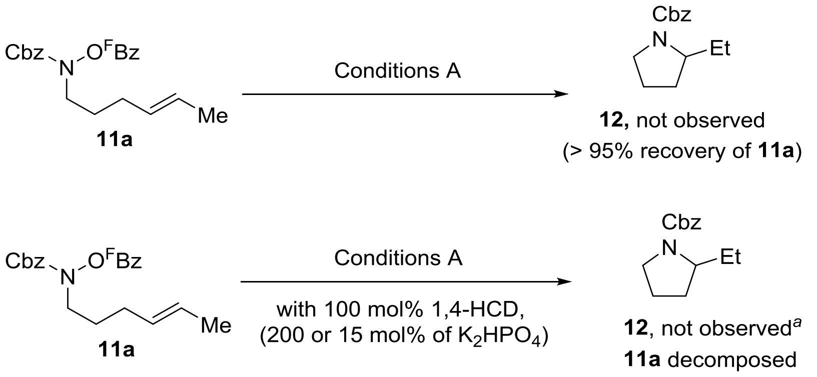

[a]No pyrrolidine product was observed by GC-MS.

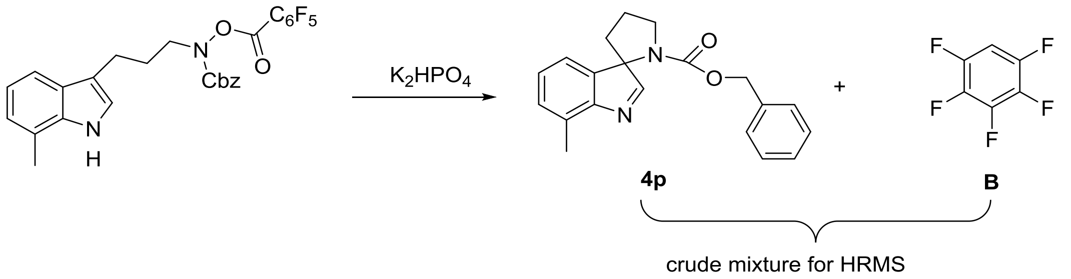

4p: **ESI-HRMS**: m/z calculated for $C_{20}H_{20}N_2NaO_2[M+Na]^+$: 343.1417; found: 343.1435.

C_6HF_5 **(B)**: **MALDI- HRMS**: m/z calculated for C_6F_5 $[M-H]^-$:166.9915; found: 166.9916.

---

4. Computational methods

An evaluation of the conformational freedom of the reactant, TS and products was performed using the GMMX routine implemented in PCMODEL v.8.5,[31] and subsequent DFT calculations using Jaguar v.8,[32,33] at the PBE/6-31+G(d) level of theory.[34,35] All subsequent geometry optimisations have been performed in Gaussian09[36] using the PBE0 hybrid functional,[37] the empirical dispersion correction of Grimme with Becke–Johnson damping (D3BJ)[38] and the 6-31+G(d) basis set (**BS1**), with five spherical harmonic components of the polarization functions, and including diffuse functions on heavy atoms. An ultrafine integration grid was used to minimise numerical error, and Gibbs free energy corrections were computed at 353 K. Stationary points on the potential energy hypersurface were characterised as minima or transition states by the presence of zero and one imaginary frequency from an analytical frequency calculation respectively.

Single point energy calculations were performed using the double hybrid functional of Grimme (B2PLYP-D3),[39] which has been shown to be more accurate than the standard hybrid functionals,[40] and the Minnesota class of meta-GGA functionals[41] for main-group thermochemistry, in conjunction with the 6-311++G(2d,p) triple-ζ basis set (**BS2**). 2,2,2-Trifluoroethanol (TFE) solvent effects were modelled implicitly with the SMD continuum solvent model (ε=26.726).[42]

The intrinsic reaction coordinate (IRC) scan[43,44] at the PBE-D3/6-31+G(d) level has been performed in Jaguar v.8, with 30 forward points and 8 backward points from an initial analytically calculated Hessian (0.1 Å step size).

Discussion

In the calculated transition state, the C–N–O(Ts) angle is 154.4°, consistent with an S_N2-like mechanism. Note that the calculations reported herein did not test the viability of an SET mechanism. Further multireference *ab initio* calculations would need to be performed to investigate the possibility of an SET mechanism, but these are outside the scope of the current study.

In modelling the reaction of **5a-Me** to **6a-Me** the following approximations have been made to afford a computationally tractable problem. (1) The reactant arene is assumed to be deprotonated, and to be sufficiently stable to exist as the free ion in solution. (2) Implicit solvation is sufficient to model the interaction between the reacting species and the solvent. Clearly, this approach does not capture any hydrogen bonding interactions present in the real system. However, we have no reason to suggest that the stabilisation of the reactant and TS will be significantly different, thus the calculated barrier is unlikely to differ considerably if explicit solvent molecules were included in the calculation.

Table S1. Energies for the species along the calculated energy pathway quoted in kcal mol^{-1} and relevant distances in Å.

Structure	$\Delta E_{PBE0/BS1}$	$\Delta H_{PBE0/BS1}$	$\Delta G_{PBE0/BS1}$	$\Delta E_{B2PLY\text{-}D3/BS2, SMD(TFE)}$	$\Delta G_{(B2PLYP\text{-}D3/BS2, SMD(TFE)}$	$d_{C(\mathit{para})–N}$	$d_{N–O(Ts)}$
**5a-Me**	0.0	0.0	0.0	0.0	0.0	4.08	1.40
**TS**	24.2	22.5	24.0	25.3	25.1	2.64	1.81
**6a-Me**	-32.0	-33.3	-45.3	-47.6	-60.9	1.47	-

IRC calculation

The IRC calculation has been performed to confirm that the TS links the reactant and product geometries. The pure GGA PBE functional was used in Jaguar to increase computational efficiency. Figure S1 depicts the relative potential (SCF) energy change over the IRC scan in addition to pertinent bond distances. See Table S2 for SCF and relative energies. The TS is very early along the reaction coordinate (d(C(*para*)–N) = 2.95 Å, 0.3 Å longer than that computed at the PBE0/6-31+G(d) level of theory), consistent with the highly exergonic reaction.

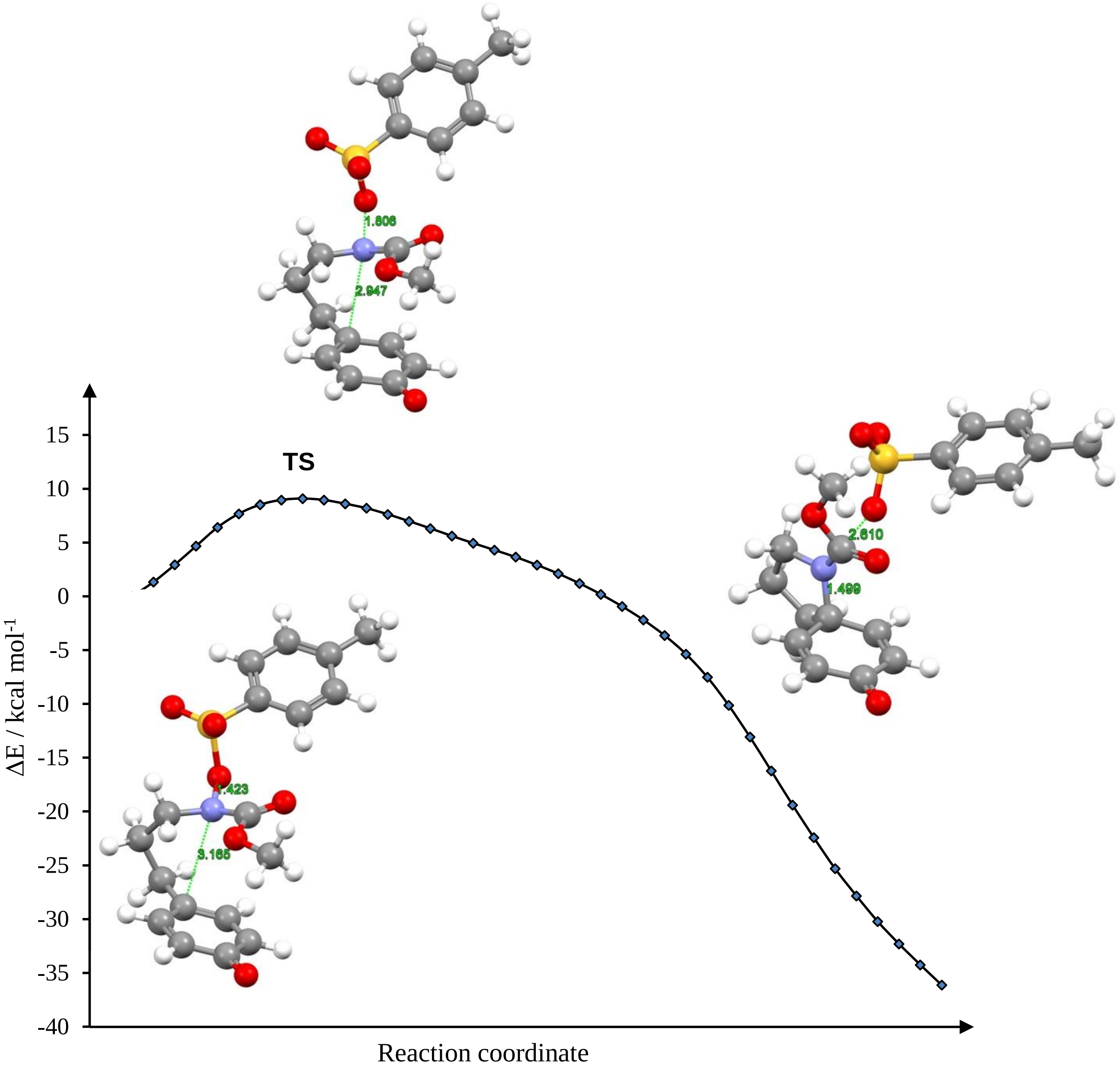

Figure S1. Relative energy plotted as a function of the reaction coordinate. Structures are shown for the IRC points -8 (reactant-like), 0 (TS) and +30 (product-like).

Table S2. SCF energies computed at the PBE-D3/6-31+G(d) level at each IRC scan point.

IRC scan point	E / Ha	ΔE / kcal mol^{-1}	IRC scan point	E / Ha	ΔE / kcal mol^{-1}
-8	-1600.685439	0.0	+12	-1600.682082	2.1
-7	-1600.683308	1.3	+13	-1600.683527	1.2
-6	-1600.680749	2.9	+14	-1600.685139	0.2
-5	-1600.677982	4.7	+15	-1600.686936	-0.9
-4	-1600.675231	6.4	+16	-1600.688936	-2.2
-3	-1600.673208	7.7	+17	-1600.691250	-3.6
-2	-1600.671865	8.5	+18	-1600.694005	-5.4
-1	-1600.671156	9.0	+19	-1600.697419	-7.5
**0 (TS)**	**-1600.670955**	**9.1**	+20	-1600.701586	-10.1
+1	-1600.671166	9.0	+21	-1600.706276	-13.1
+2	-1600.671724	8.6	+22	-1600.711292	-16.2
+3	-1600.672385	8.2	+23	-1600.716335	-19.4
+4	-1600.673289	7.6	+24	-1600.721181	-22.4
+5	-1600.674315	7.0	+25	-1600.725785	-25.3
+6	-1600.675375	6.3	+26	-1600.729797	-27.8
+7	-1600.676490	5.6	+27	-1600.733601	-30.2
+8	-1600.677553	4.9	+28	-1600.736928	-32.3
+9	-1600.678578	4.3	+29	-1600.740014	-34.2
+10	-1600.679623	3.6	+30	-1600.743009	-36.1
+11	-1600.680792	2.9			

Cartesian Coordinates

5a-Me

E (PBE0/6-31+G(d)) = -1600.7773946

H (PBE0/6-31+G(d), 353K) = -1600.379792

G (PBE0/6-31+G(d), 353K) = -1600.490465

E (B2PLYP-D3/6-311++G(2d,p), SMD(TFE)) = -1601.5483333

C	-3.87818	-1.07989	0.13531
C	-4.17558	-0.36329	-1.03614
C	-4.52179	0.97800	-1.01637
C	-4.55671	1.75057	0.20074
C	-4.33236	0.97074	1.38983
C	-3.99354	-0.37471	1.34320
H	-4.11414	-0.87909	-1.99872
H	-4.73830	1.50652	-1.94449
O	-4.73921	3.00828	0.21493
H	-4.39603	1.49063	2.34506
H	-3.79062	-0.90161	2.28005
C	-3.27536	-2.45582	0.06355
C	-1.80808	-2.40526	-0.40453
H	-3.83153	-3.10243	-0.63217
H	-3.32808	-2.94579	1.04815
C	-0.93310	-1.64572	0.59203
N	0.19192	-0.93495	-0.00684
C	0.00402	0.25543	-0.72314
O	0.68598	0.60421	-1.66285
O	-0.97307	0.95009	-0.15679
C	-1.30269	2.19937	-0.77187
H	-1.40738	-3.41415	-0.57387
H	-1.78027	-1.88390	-1.37030
H	-0.52034	-2.30515	1.36145
H	-1.52604	-0.87050	1.08443
O	1.05645	-1.79549	-0.69870
H	-2.14963	2.59108	-0.20926
H	-1.59570	2.03468	-1.81072
H	-0.43856	2.86994	-0.72893
S	2.47493	-1.96246	0.14277
O	2.17332	-2.42374	1.48840
O	3.29270	-2.75122	-0.76045
C	3.87162	2.35853	0.28479
C	3.13994	1.81292	1.34483
C	2.73305	0.48380	1.32569
C	3.06860	-0.30303	0.22793

C	3.79844	0.20875	-0.84067
C	4.20418	1.53586	-0.79747
C	4.25960	3.80967	0.28968
H	2.87363	2.43990	2.19275
H	2.15733	0.05678	2.14024
H	4.02878	-0.42517	-1.69080
H	4.77218	1.94778	-1.62858
H	5.19807	3.97627	-0.24970
H	3.48620	4.41459	-0.20111
H	4.37630	4.19098	1.3093

TS

E (PBE0/6-31+G(d)) = -1600.7387925
H (PBE0/6-31+G(d), 353K) = -1600.343966
G (PBE0/6-31+G(d), 353K) = -1600.452226
Imaginary frequency = -397.4 cm^{-1}
E (B2PLYP-D3/6-311++G(2d,p), SMD(TFE)) = -1601.5079502

C	3.396936	0.649011	1.128588
C	4.296300	0.701268	0.038544
C	4.848521	-0.431605	-0.507889
C	4.550589	-1.757757	0.003468
C	3.669777	-1.780575	1.149014
C	3.129917	-0.629320	1.674038
H	4.546863	1.673387	-0.389427
H	5.522626	-0.374085	-1.360471
O	5.010017	-2.799068	-0.532002
H	3.413832	-2.754363	1.560614
H	2.435273	-0.694463	2.511154
C	2.873947	1.912123	1.753993
C	2.184214	2.829013	0.730848
H	3.677595	2.470525	2.260533
H	2.145629	1.637805	2.524519
C	1.418436	1.998898	-0.301499
N	0.877242	0.822755	0.364924
C	0.849453	-0.424947	-0.291793
O	0.209290	-1.368997	0.124670
O	1.658418	-0.482234	-1.348548
C	1.769325	-1.761397	-1.963896
H	2.913088	3.455139	0.197740
H	1.504298	3.513317	1.253276
H	2.056946	1.682377	-1.129904
H	0.609227	2.588034	-0.742873

O	-0.871530	1.202470	0.597645
H	2.563833	-1.655969	-2.703231
H	2.047232	-2.518922	-1.228756
H	0.818776	-2.024440	-2.438766
S	-1.817603	1.324044	-0.644031
O	-1.184902	0.731809	-1.826167
O	-2.336483	2.690457	-0.771312
C	-5.331792	-1.343257	0.558508
C	-4.017519	-1.829465	0.589559
C	-2.940502	-1.025054	0.240218
C	-3.184211	0.291594	-0.150493
C	-4.474808	0.805143	-0.183973
C	-5.542756	-0.018923	0.168887
C	-6.480817	-2.232852	0.945563
H	-3.834030	-2.856987	0.899197
H	-1.917376	-1.393247	0.280854
H	-4.628817	1.838529	-0.479767
H	-6.556315	0.377439	0.143266
H	-6.387202	-2.573170	1.984478
H	-7.439541	-1.712347	0.848680
H	-6.519500	-3.129434	0.314508

6a-Me

E (PBE0/6-31+G(d)) = -706.6526583
H (PBE0/6-31+G(d), 353K) = -706.402427
G (PBE0/6-31+G(d), 353K) = -706.473746
E (B2PLYP-D3/6-311++G(2d,p), SMD(TFE)) = -707.0293749

C	0.38970	0.72988	0.14833
C	1.04784	0.41913	-1.16862
C	2.21061	-0.23370	-1.27352
C	2.91173	-0.76016	-0.08703
C	2.21990	-0.60697	1.20465
C	1.04713	0.02938	1.29959
H	0.53376	0.78773	-2.05536
H	2.68482	-0.41745	-2.23387
O	4.00362	-1.30863	-0.17593
H	2.69860	-1.06385	2.06628
H	0.52785	0.11134	2.25375
C	0.40925	2.26587	0.39942
C	-0.85754	2.77116	-0.28346
H	1.33393	2.71493	0.02864
H	0.35183	2.44879	1.47957

C	-1.87563	1.68296	0.03850
N	-1.05631	0.47498	0.09313
C	-1.53671	-0.79307	0.01295
O	-0.85378	-1.79967	-0.00767
O	-2.88835	-0.79366	-0.03600
C	-3.47247	-2.09050	-0.12384
H	-0.70845	2.84543	-1.36706
H	-1.16849	3.75517	0.07956
H	-2.65840	1.58274	-0.71781
H	-2.36472	1.86090	1.00621
H	-4.54940	-1.92125	-0.15606
H	-3.13720	-2.60418	-1.02883
H	-3.20464	-2.69318	0.74808

TsO$^-$

E (PBE0/6-31+G(d)) = -894.1756736

H (PBE0/6-31+G(d), 353K) = -894.030493

G (PBE0/6-31+G(d), 353K) = -894.088947

E (B2PLYP-D3/6-311++G(2d,p), SMD(TFE)) = -894.5947753

O	2.338169	-0.815590	-1.190446
S	1.978341	-0.004524	-0.003311
O	2.353557	1.426103	-0.098376
O	2.324590	-0.644608	1.287816
C	-2.644796	0.000797	0.002263
C	-1.928074	1.201102	0.023196
C	-0.534031	1.207583	0.016368
C	0.170255	0.007121	-0.012110
C	-0.529762	-1.197671	-0.033694
C	-1.921992	-1.197012	-0.026427
C	-4.150245	0.001536	0.005345
H	-2.469561	2.147158	0.044485
H	0.030632	2.135653	0.029189
H	0.027631	-2.130178	-0.060438
H	-2.461490	-2.143783	-0.044156
H	-4.547347	-1.019702	0.027386
H	-4.551404	0.533506	0.877863
H	-4.555310	0.495846	-0.887702

5. References

1. M. P. Paudyal, A. M. Adebensin, S. R. Burt, D. H. Ess, Z. Ma, L. Kurti, J. R. Falck, *Science*. **2016**, *353*, 1144-1147.
2. K.R. Campos, J. C.Woo, S. Lee, R. D. Tillyer, *Org. Lett.* **2004**, *6*, 79-82.
3. A. V. Novikov, A. Sabahi, A. M. Nyong, J. D. Rainier, *Tetrahedron: Asymmetry* **2003**, *14*, 911-915.
4. J. F. Austin, D. W. C. MacMillan, *J. Am. Chem. Soc.* **2002**, *124*, 1172-1173.
5. N. T. Patil, A. Konala, *Eur. J. Org. Chem.* **2010**, 6831-6839.
6. Y. Oikawa, H. Hirasawa, O. Yonemitsu, *Chem. Pharm. Bulletin*, **1982**, *30*, 3092-3096.
7. X. Liang, S. Li, W. Su, *Tetrahedron Lett.* **2012**, *53*, 289-291.
8. I. R. Hazelden, X. Ma, T. Langer, J. F. Bower, *Angew. Chem. Int. Ed.* **2016**, *55*, 11198-11202.
9. Ł. Albrecht, H. Jiang, G. Dickmeiss, B. Gschwend, S. G. Hansen, K. A. Jørgensen, *J. Am. Chem. Soc.* **2010**, *132*, 9188-9196.
10. D. E. Olson, J. Du Bois, *J. Am. Chem. Soc.* **2008**, *130*, 11248-11249.
11. Masuri, A. C. Willis, M. D. McLeod, *J. Org. Chem.* **2012**, *77*, 8480-849.
12. A. Defoin, H. Fritz, C. Schmidlin, J. Streith, *Helv. Chim. Acta* **1987**, *70*, 554-569.
13. R. K. Varshnaya, P. Banerjee, *Eur. J. Org. Chem.* **2016**, 4059-4066.
14. J. P. Lajiness, W. M. Robertson, I. Dunwiddie, M. A. Broward, G. A.Vielhauer, S. J. Weir, D. L. Boger, *J. Med. Chem.* **2010**, *53*, 7731-7738.
15. J. B. Behr, C. Chevrier, A. Defoin, C. Tarnus, J. Streith, *Tetrahedron* **2003**, *59*, 543-553.
16. C. Greck, L. Bischoff, F. Ferreira, J. P. Genbt, *J. Org. Chem.* **1995**, *60*, 7010-7012.
17. W. T. Ashton, L. L. Chang, J. Hannah, G. H. Rasmusson, R. L. Tolman, **1991**, *EP438873* A1.
18. V. Rauniyar, D. G. Hall, *J. Org. Chem.* **2009**, *74*, 4236-4241.
19. M. Dexter, J. D. Spivack, D. H. Steinberg, **1972**, US3642868.
20. S. R. Angle, D. O. Arnaiz, J. P. Boyce, R. P. Frutos, M. S. Louie, H. L. Mattson-Arnaiz, J. D. Rainier, K. D. Turnbull, W. Yang, *J. Org. Chem.* **1994**, *59*, 6322-6337.
21. X. Xing, D. Padmanaban, L.-A. Yeh, G. D. Cuny, *Tetrahedron*, **2002**, *58*, 7903-7910.
22. H. Zhang, X. Huang, *Adv. Synth. Catal.* **2016**, *358*, 3736-3742.
23. D. Boschi, G. C. Tron, L. Lazzarato, K. Chegaev, C. Cena, A. Di Stilo, M. Giorgis, M. Bertinaria, R. Fruttero, A. Gasco, *J. Med. Chem.* **2006**, *49*, 2886-2897.
24. A. Rudolph, P. H. Bos, A. Meetsma, A. J. Minnaard, B. L. Feringa, *Angew. Chem., Int. Ed.* **2011**, *50*, 5834-5838.
25. S. Chaabouni, F. Simonet, A. François, S. Abid, C. Galaup, S. Chassaing, *Eur. J. Org. Chem.* **2017**, 271-277.
26. H. Lu, F.-M. Zhang, J.-L. Pan, T. Chen, Y.-F. Li, *J. Org. Chem.* **2014**, *79*, 546-558.
27. A. Sudo, Y. Zhang, T. Endo, *J. Polym. Sci., Part A: Polym. Chem.* **2011**, *49*, 619-624.

28. N. Jain, M. A. Ciufolini, *Synlett* **2015**, *26*, 631-634.
29. J. Huang, W.Wang, H-Y. He, L. Jian, H.-Y. Fu, X.-L. Zheng, H. Chen, R.-X. Li, *J. Org. Chem.* **2017**, *82*, 2523-2534.
30. C. Li, B. Breit, *Chem. Eur. J.* **2016**, *22*, 14655-14663.
31. K. Gilbert, PCModel; Serena Software: Bloomington, IN (**2004**).
32. Jaguar v. 8.5, Schrodinger, Inc., New York, NY (**2014**).
33. A. D. Bochevarov, E. Harder, T.F. Hughes, J.R. Greenwood, D. A. Braden, D. M. Philipp, D. Rinaldo, M. D. Halls, J. Zhang, R. A. Friesner, *Int. J. Quantum Chem.* **2013**, *113*, 2110-2142.
34. R. Ditchfield, W. J. Hehre, J. A. Pople, *J. Chem. Phys.* **1971**, *54*, 724-728.
35. M. M. Francl, W.J. Pietro, W. J. Hehre, J. S. Binkley, M. S. Gordon, D. J. DeFrees, J. A. Pople, *J. Chem. Phys.* **1982**, *77*, 3654-3665.
36. Gaussian 09, Revision D.01, M. J. Frisch, G. W. Trucks, H. B. Schlegel, G. E. Scuseria, M. A. Robb, J. R. Cheeseman, G. Scalmani, V. Barone, B. Mennucci, G. A. Petersson, H. Nakatsuji, M. Caricato, X. Li, H. P. Hratchian, A. F. Izmaylov, J. Bloino, G. Zheng, J. L. Sonnenberg, M. Hada, M. Ehara, K. Toyota, R. Fukuda, J. Hasegawa, M. Ishida, T. Nakajima, Y. Honda, O. Kitao, H. Nakai, T. Vreven, J. A. Montgomery, Jr., J. E. Peralta, F. Ogliaro, M. Bearpark, J. J. Heyd, E. Brothers, K. N. Kudin, V. N. Staroverov, R. Kobayashi, J. Normand, K. Raghavachari, A. Rendell, J. C. Burant, S. S. Iyengar, J. Tomasi, M. Cossi, N. Rega, J. M. Millam, M. Klene, J. E. Knox, J. B. Cross, V. Bakken, C. Adamo, J. Jaramillo, R. Gomperts, R. E. Stratmann, O. Yazyev, A. J. Austin, R. Cammi, C. Pomelli, J. W. Ochterski, R. L. Martin, K. Morokuma, V. G. Zakrzewski, G. A. Voth, P. Salvador, J. J. Dannenberg, S. Dapprich, A. D. Daniels, Ö. Farkas, J. B. Foresman, J. V. Ortiz, J. Cioslowski, and D. J. Fox, Gaussian, Inc., Wallingford CT, **2009**.
37. J. P. Perdew, M. Ernzerhof, K. Burke, *J. Chem. Phys.* **1996**, *105*, 9982-9985.
38. S. Grimme, S. Ehrlich, L. Goerigk, *J. Comput. Chem.* **2011**, *32*, 1456-1465.
39. S. Grimme, *J. Chem. Phys.* **2006**, *124*, 34108.
40. L. Goerigk, S. Grimme, *J. Chem. Theory Comput.* **2011**, *7*, 291-309.
41. L. Goerigk, S. Grimme, *Phys. Chem. Chem. Phys.* **2011**, *13*, 6670.
42. A. V. Marenich, C. J. Cramer, D. G. Truhlar, *J. Phys. Chem. B* **2009**, *113*, 6378–6396.
43. C. Gonzalez, H. B. Schlegel, *J. Chem. Phys.* **1989**, *90*, 2154-2161.
44. C. Gonzalez, H. B. Schlegel, *J. Phys. Chem.* **1990**, *94*, 5523-5527.

6. Spectra of novel compounds

3-(5,7-Dimethyl-1*H*-indol-3-yl)propan-1-ol (S9)

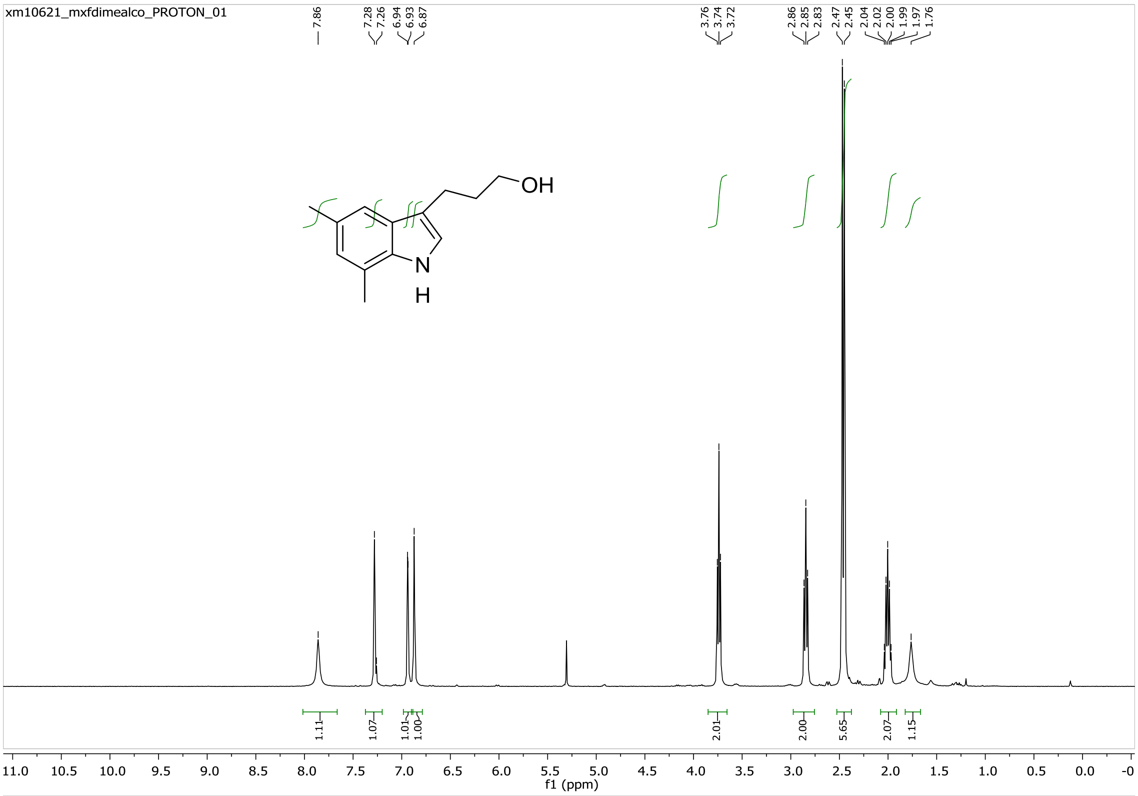

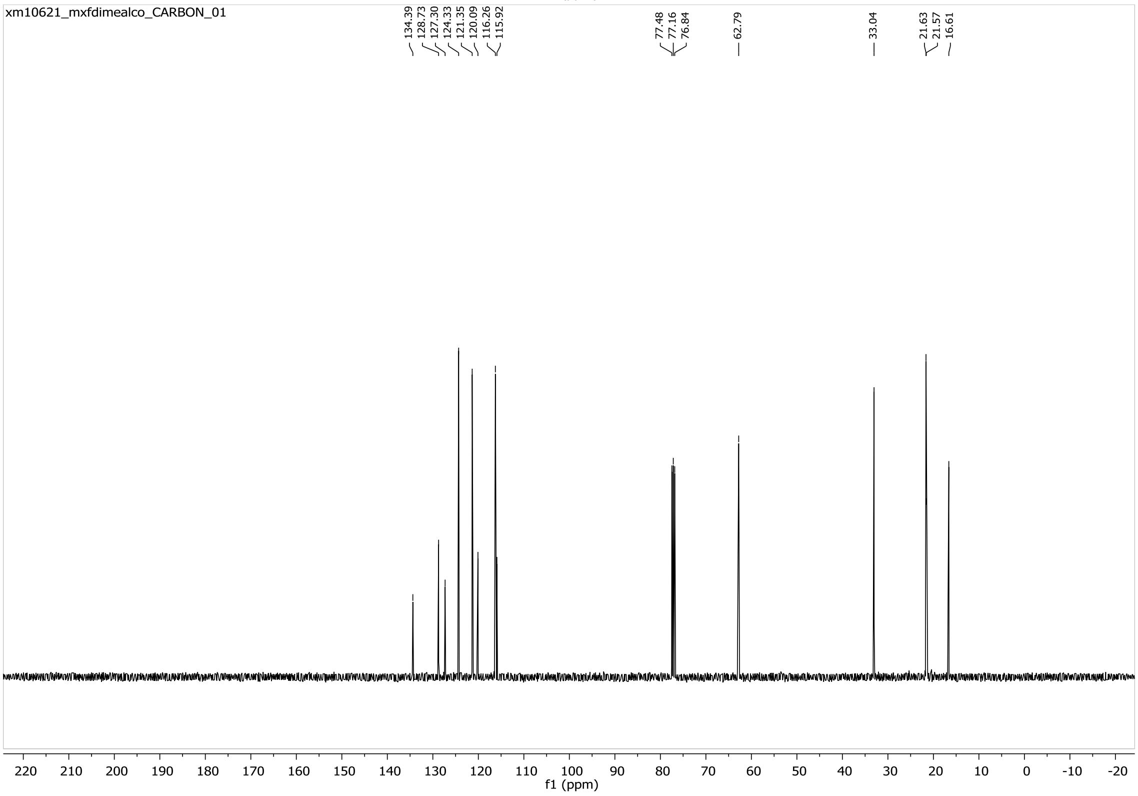

3-(1*H*-Indol-3-yl)-4-methylpentan-1-ol (S15b)

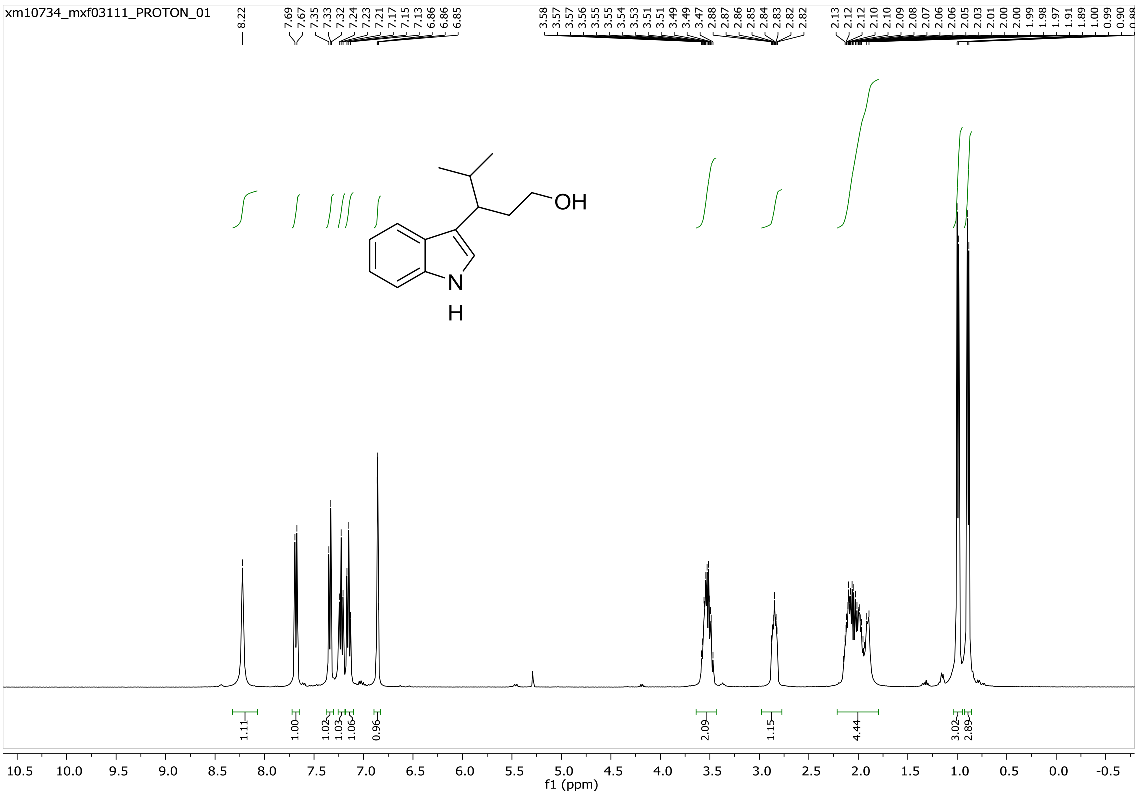

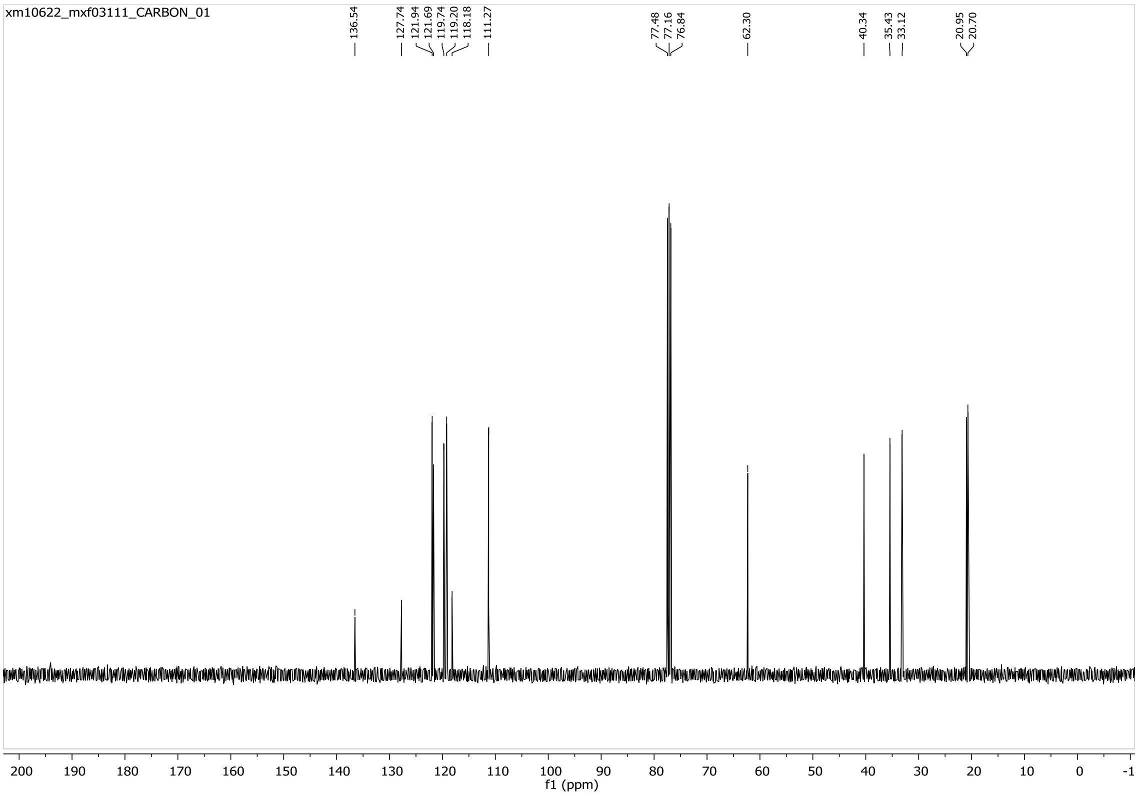

4-Methoxy-*N*-((perfluorobenzoyl)oxy)benzenesulfonamide (2b)

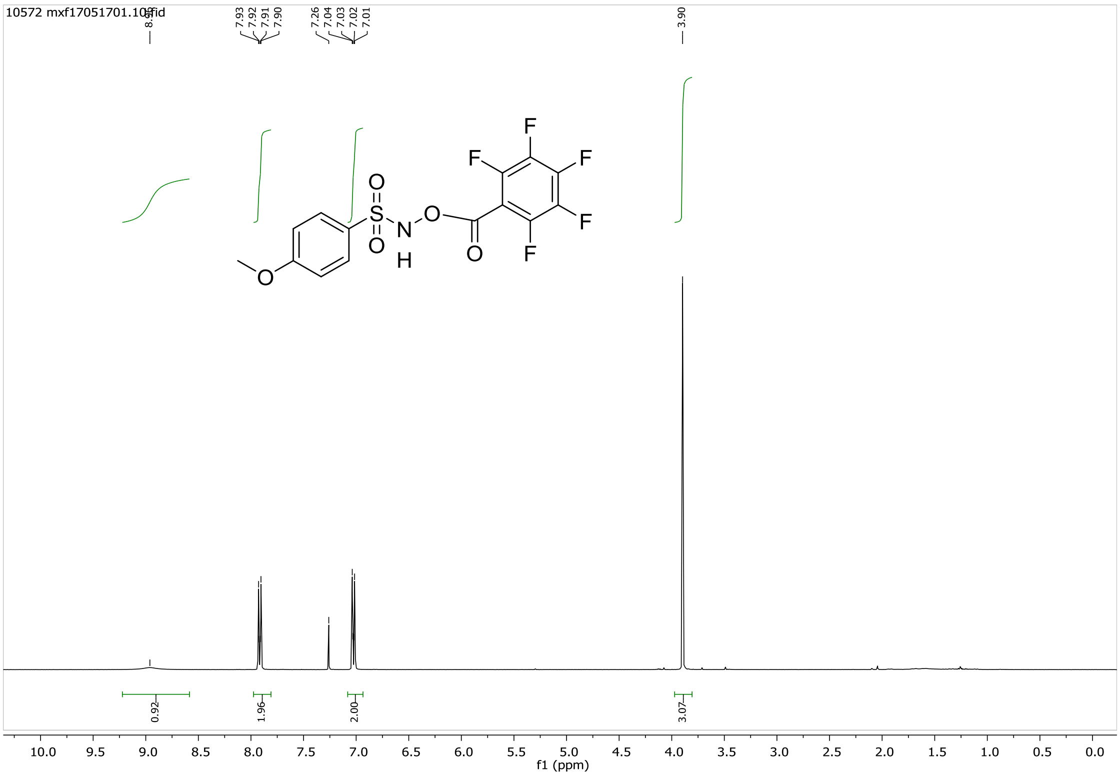

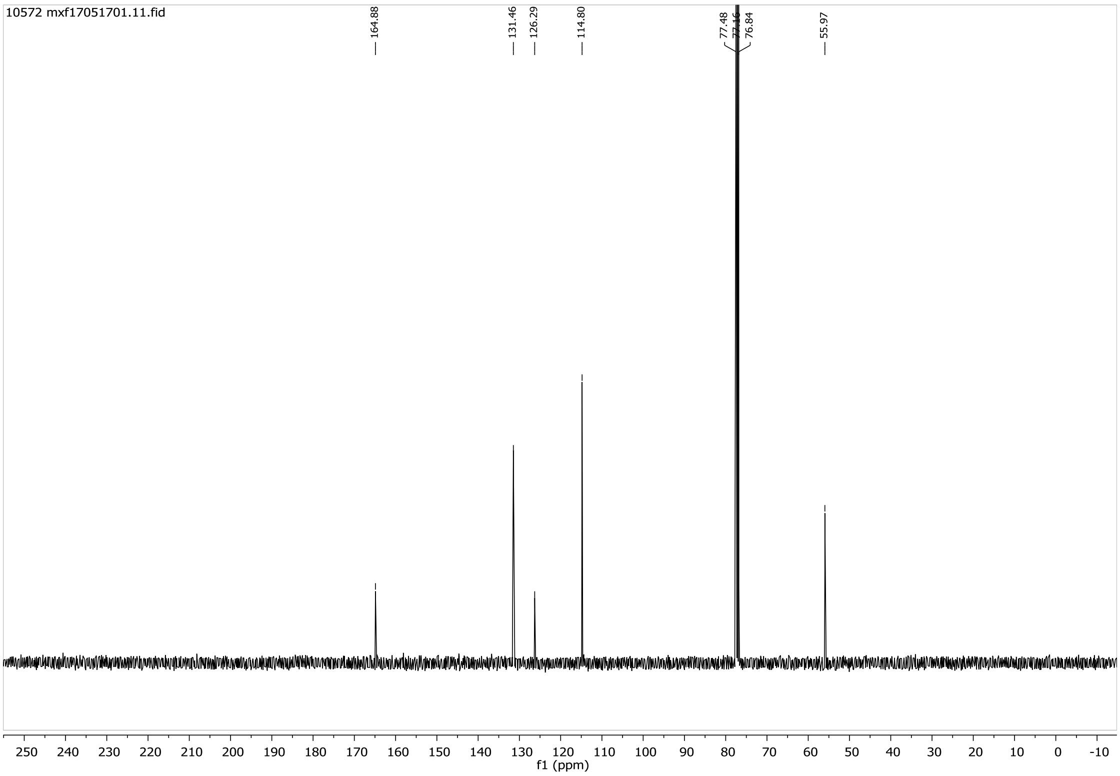

Benzyl (perfluorobenzoyl)oxycarbamate (2c)

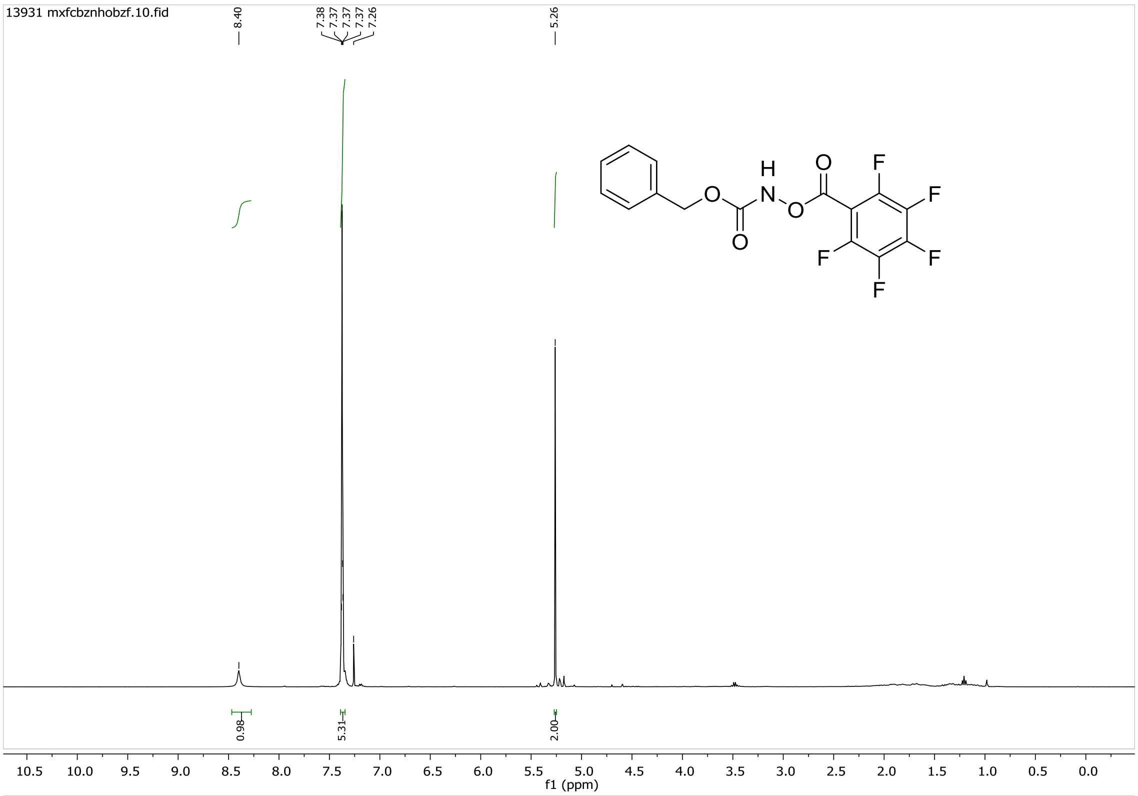

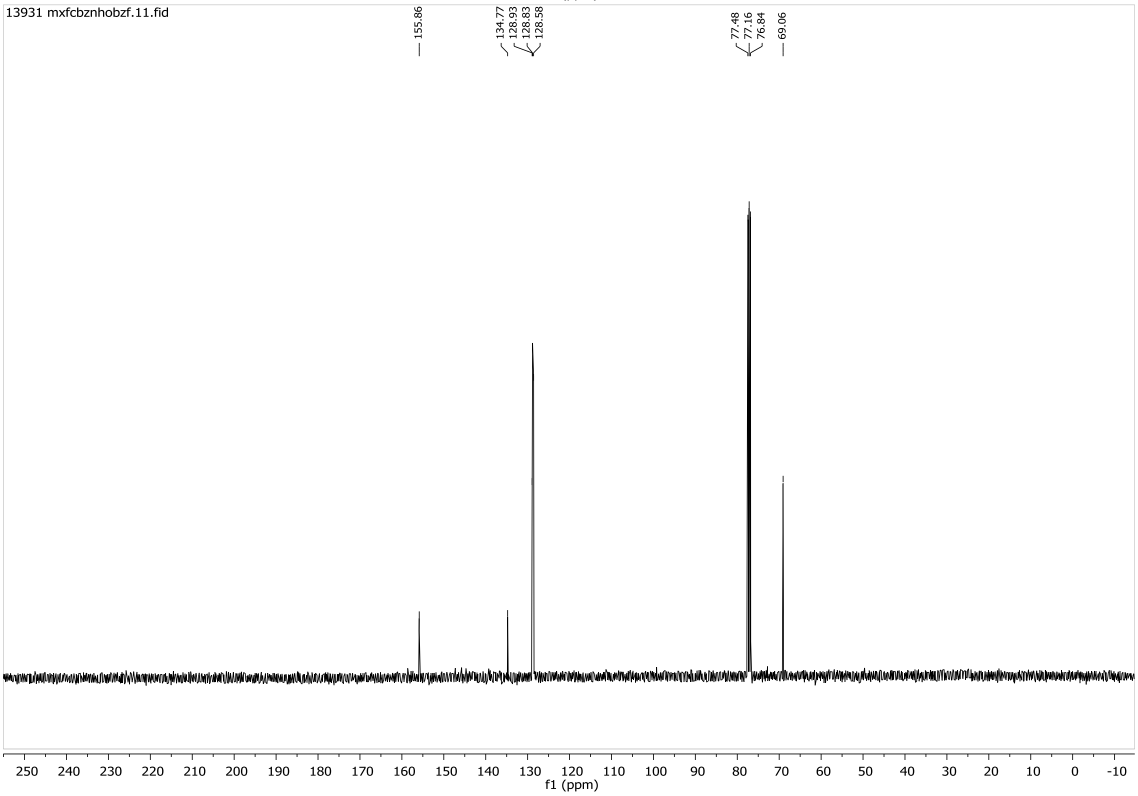

Methyl (perfluorobenzoyl)oxycarbamate (2d)

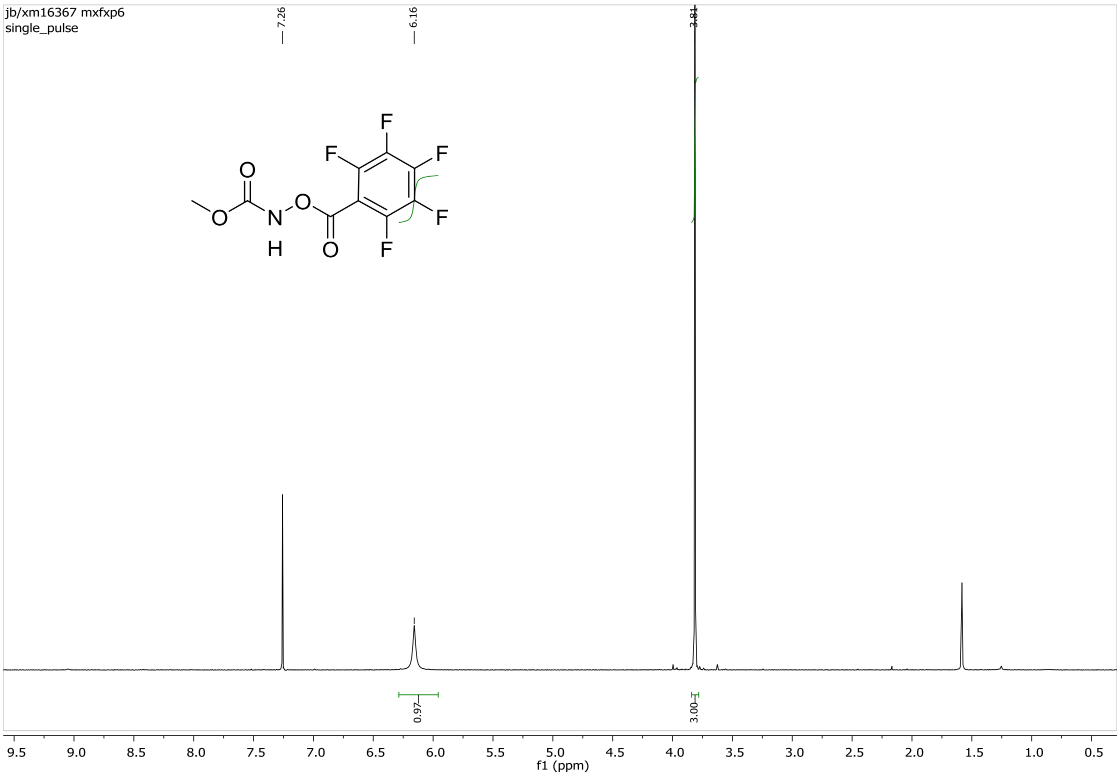

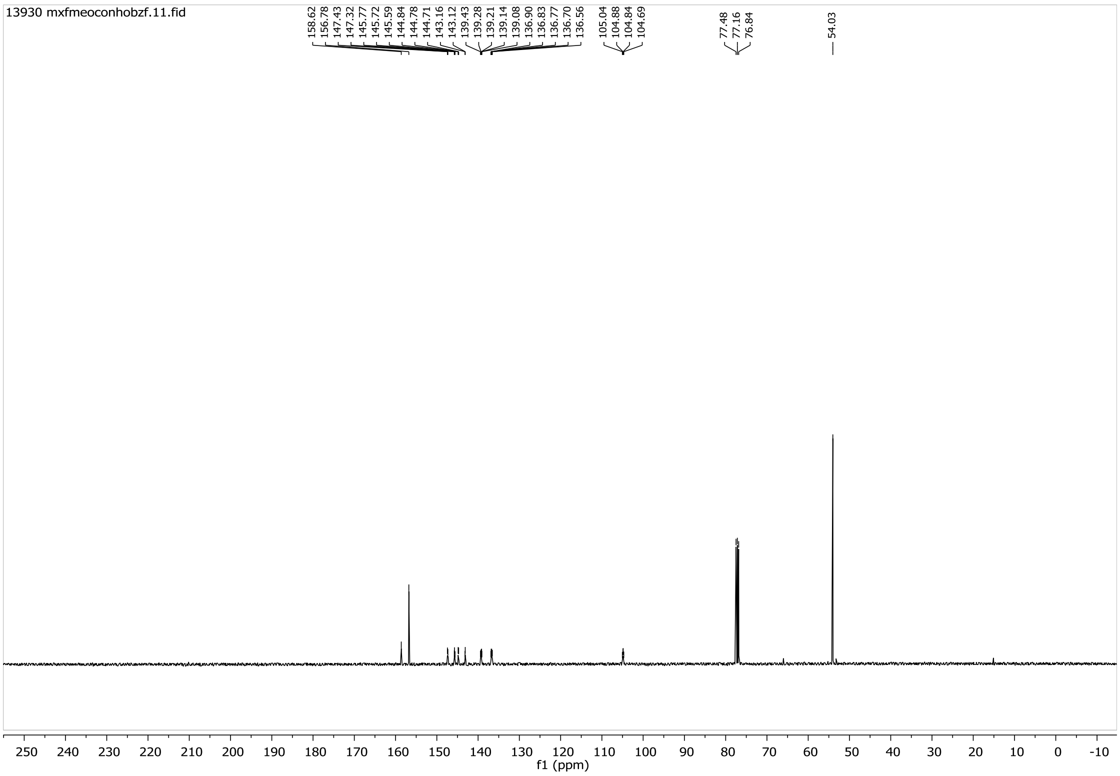

N-(3-(1*H*-Indol-3-yl)propyl)-4-methyl-*N*-((perfluorobenzoyl)oxy)benzenesulfonamide (3a)

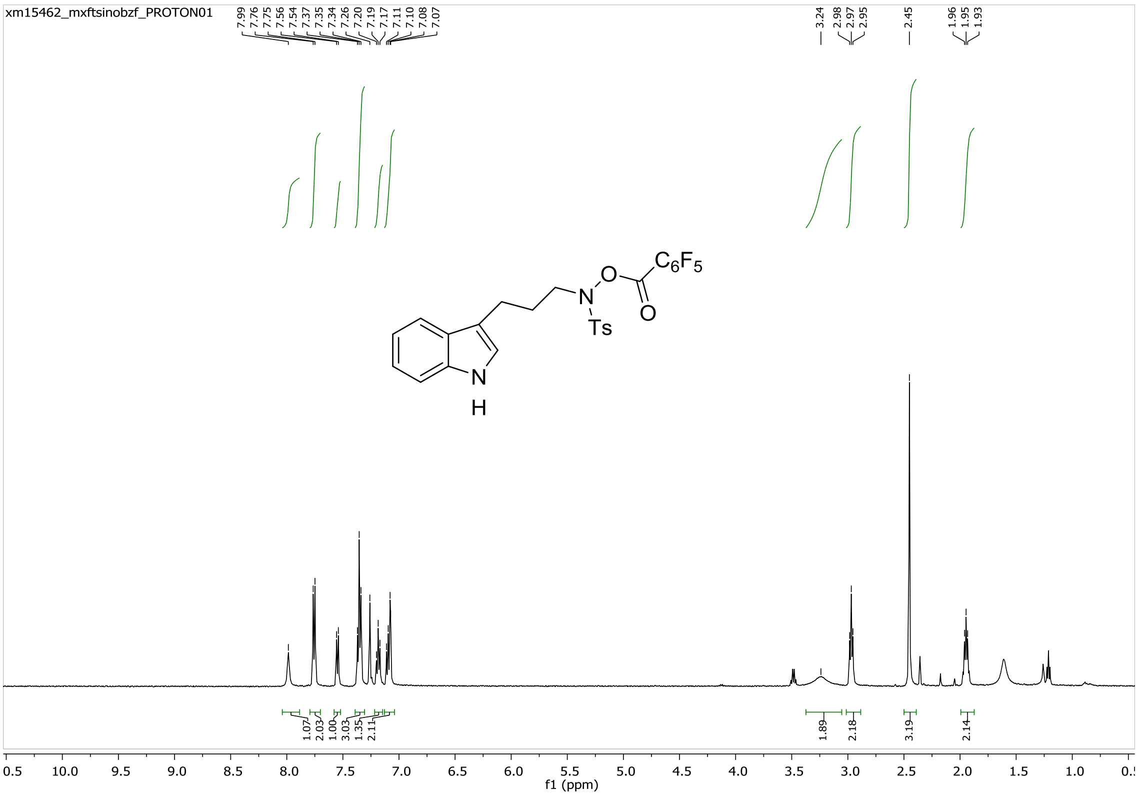

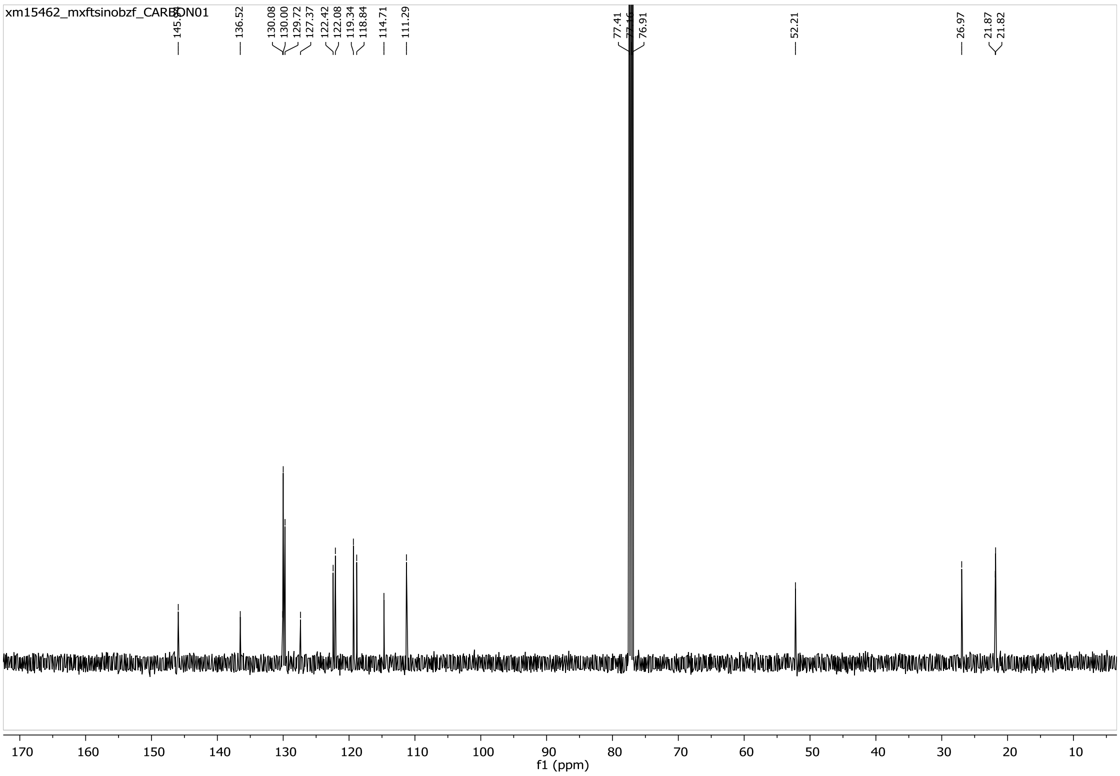

N-(3-(1*H*-Indol-3-yl)propyl)-4-methyl-*N*-(tosyloxy)benzenesulfonamide (3a')

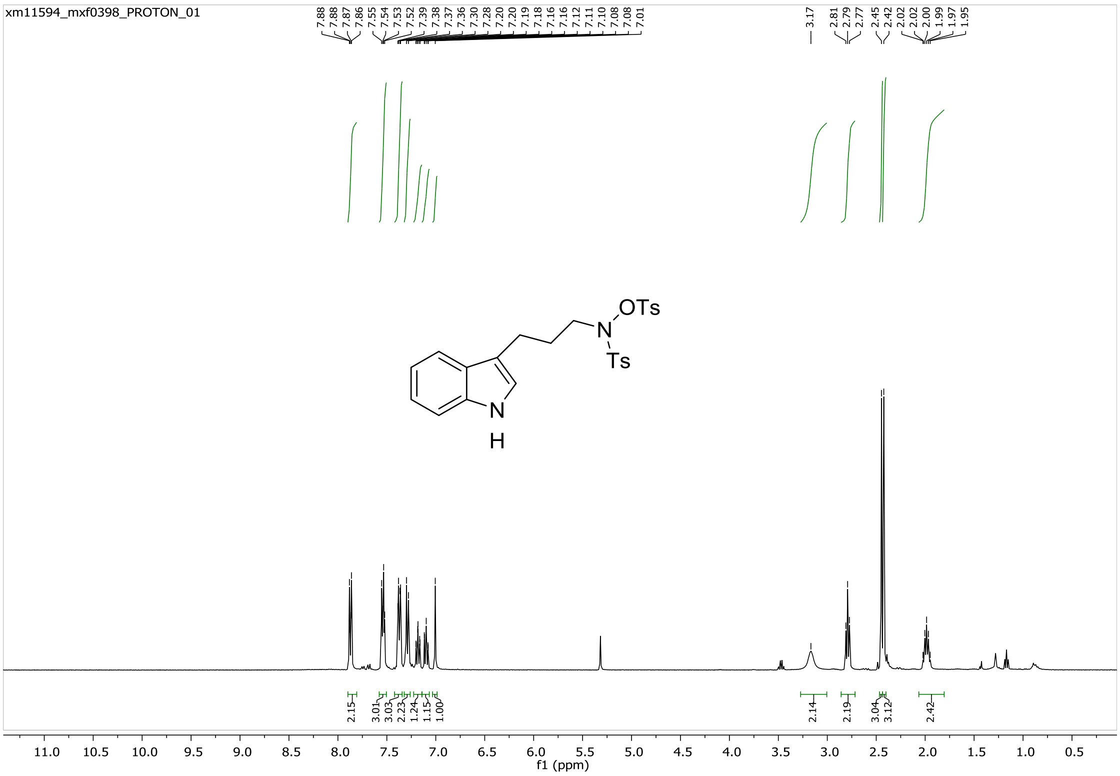

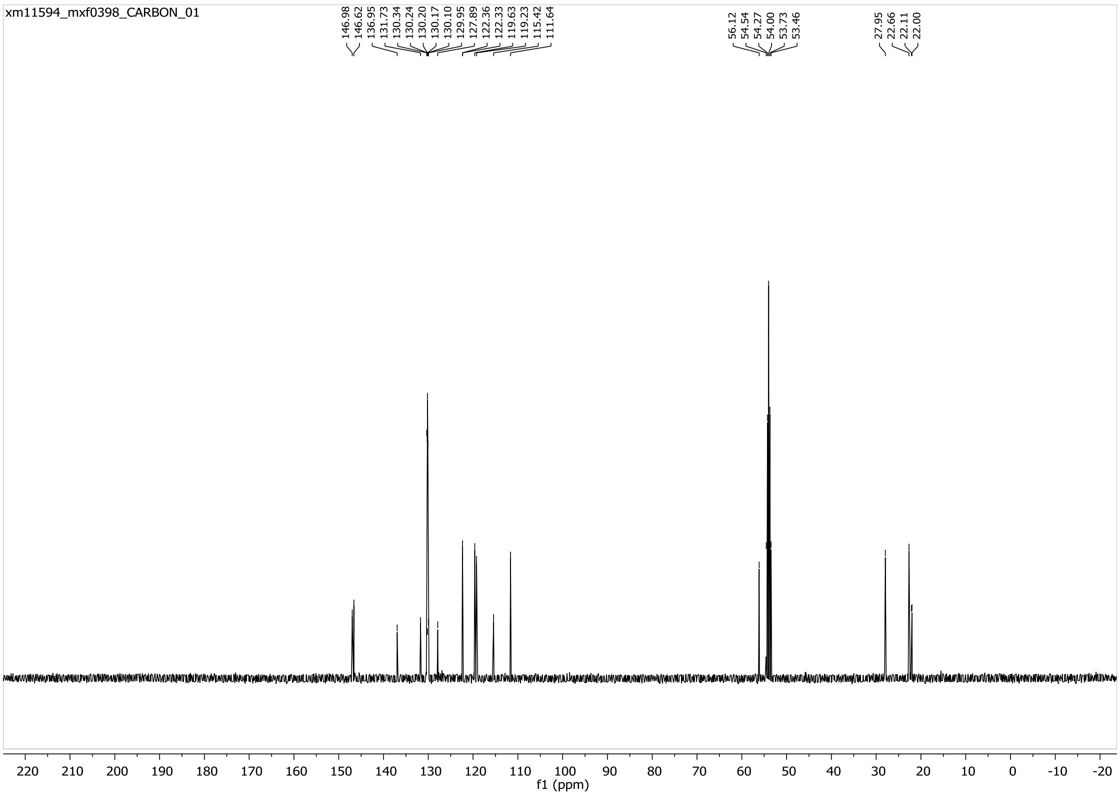

_N_-(3-(1_H_-Indol-3-yl)propyl)-4-methoxy-_N_-((perfluorobenzoyl)oxy)benzenesulfonamide

(3b)

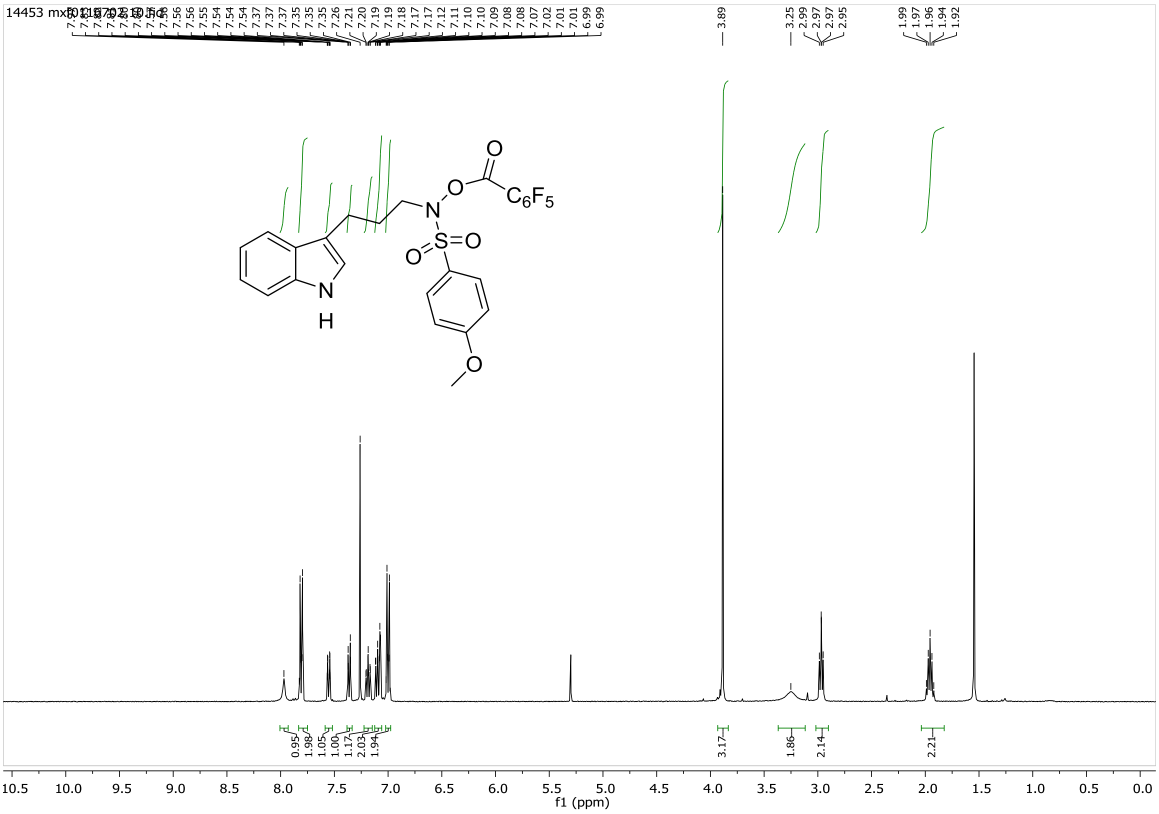

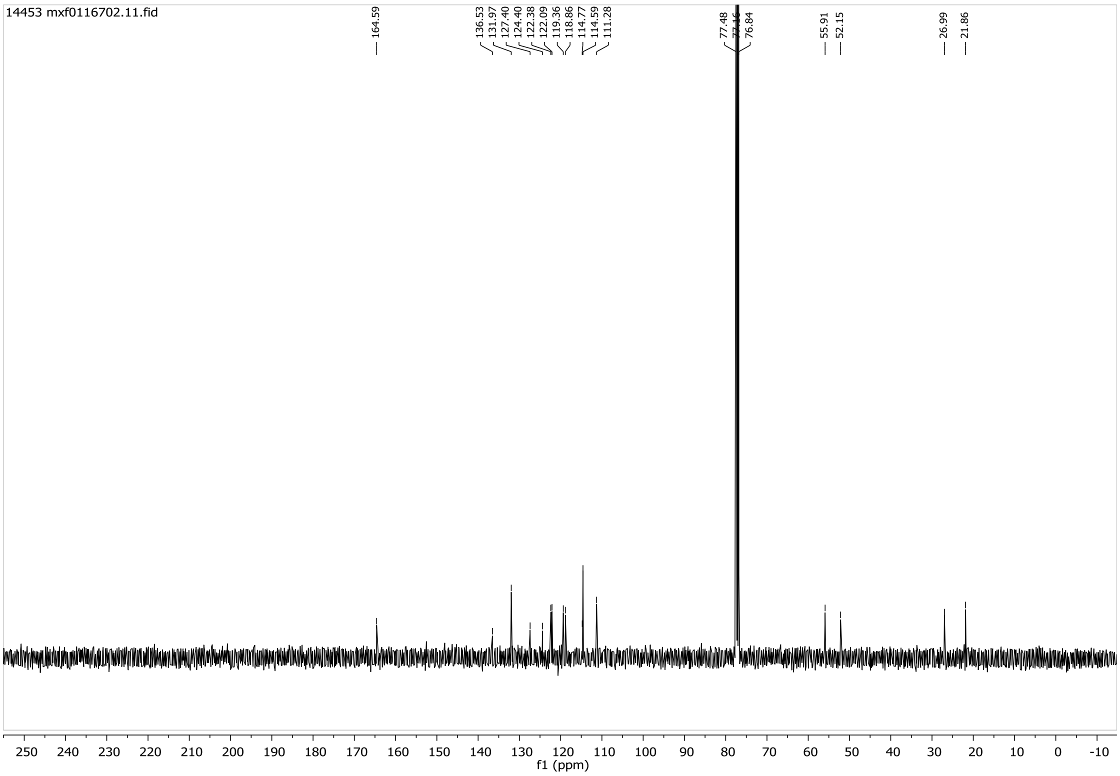

Benzyl (3-(1*H*-Indol-3-yl)propyl)((perfluorobenzoyl)oxy)carbamate (3c)

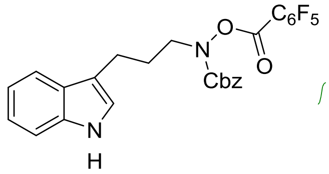

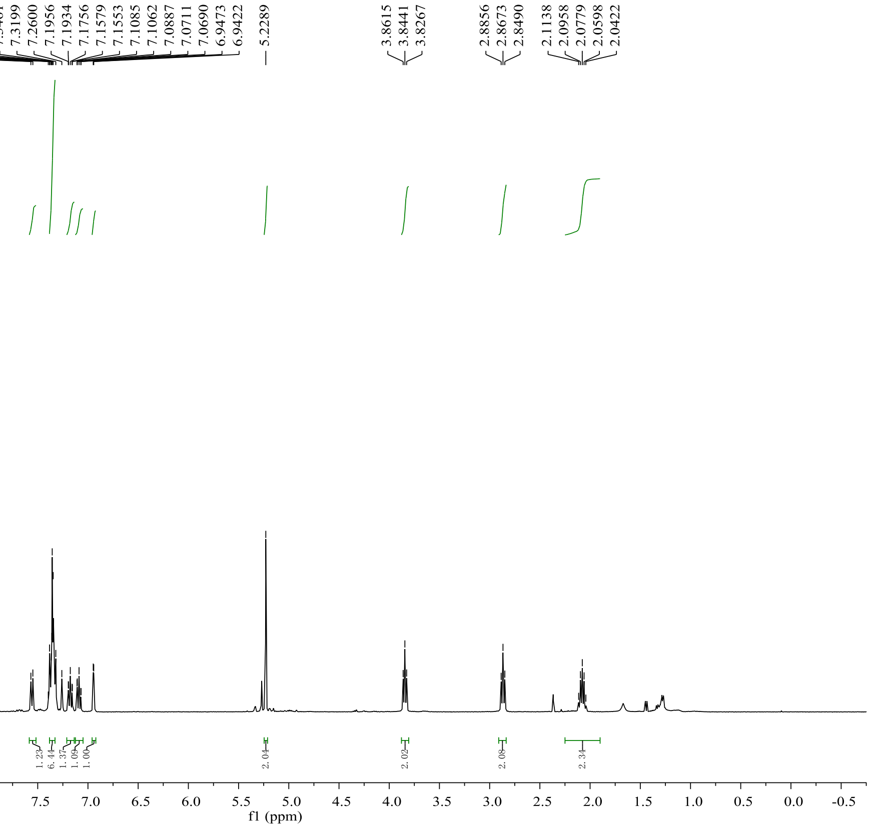

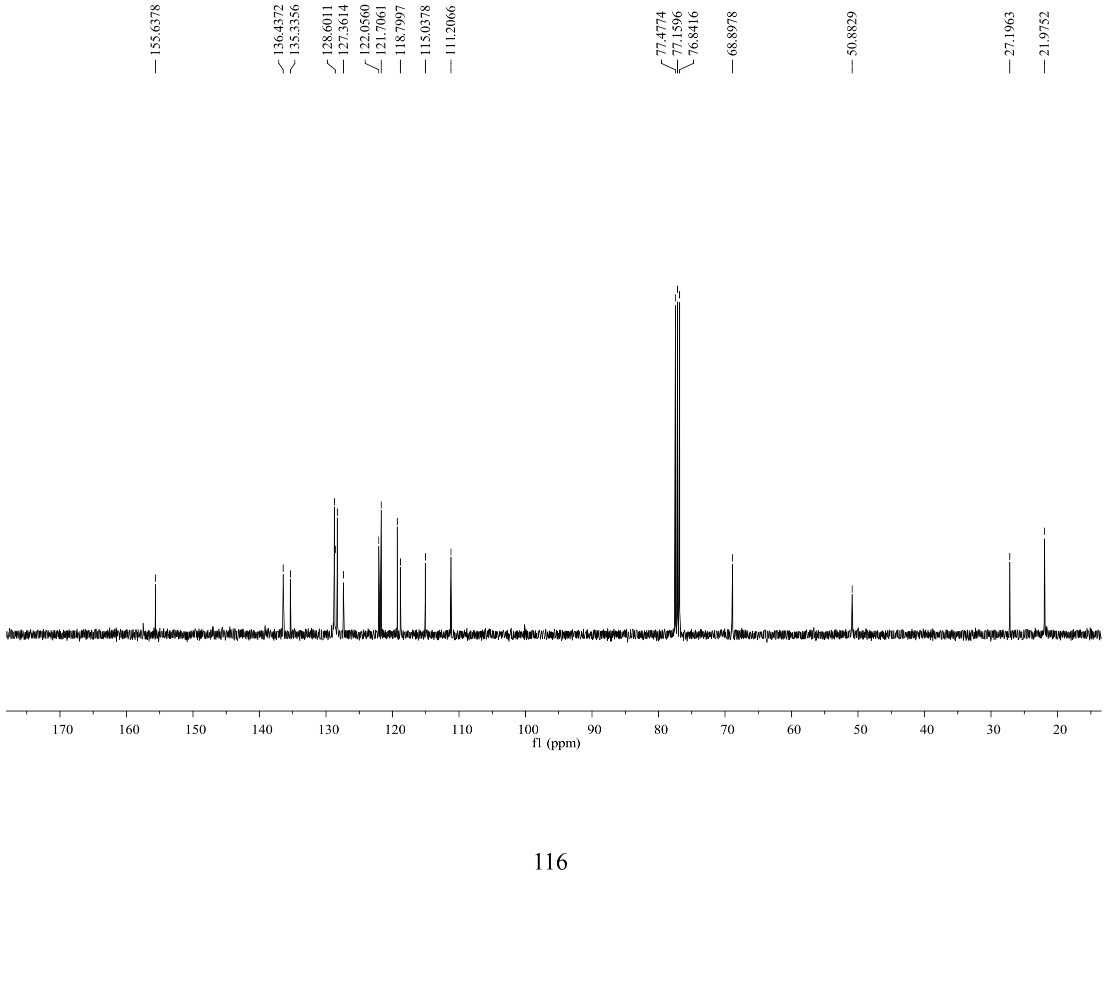

Benzyl (3-(1*H*-indol-3-yl)propyl)(tosyloxy)carbamate (3c’)

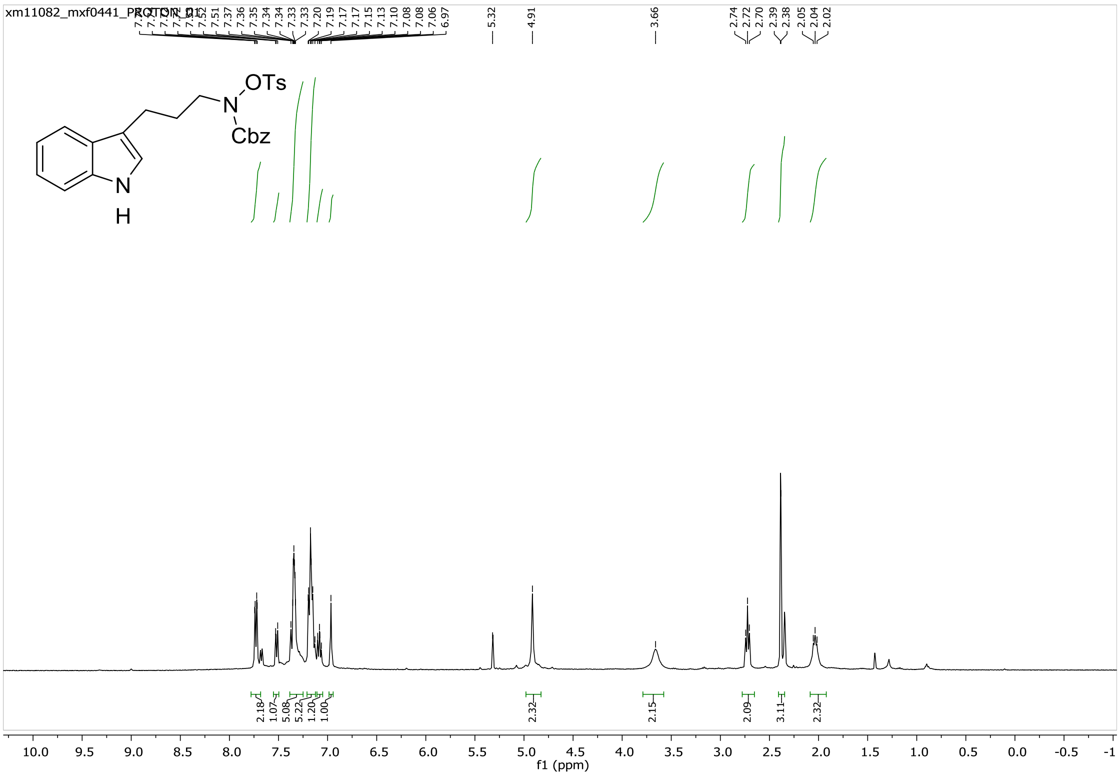

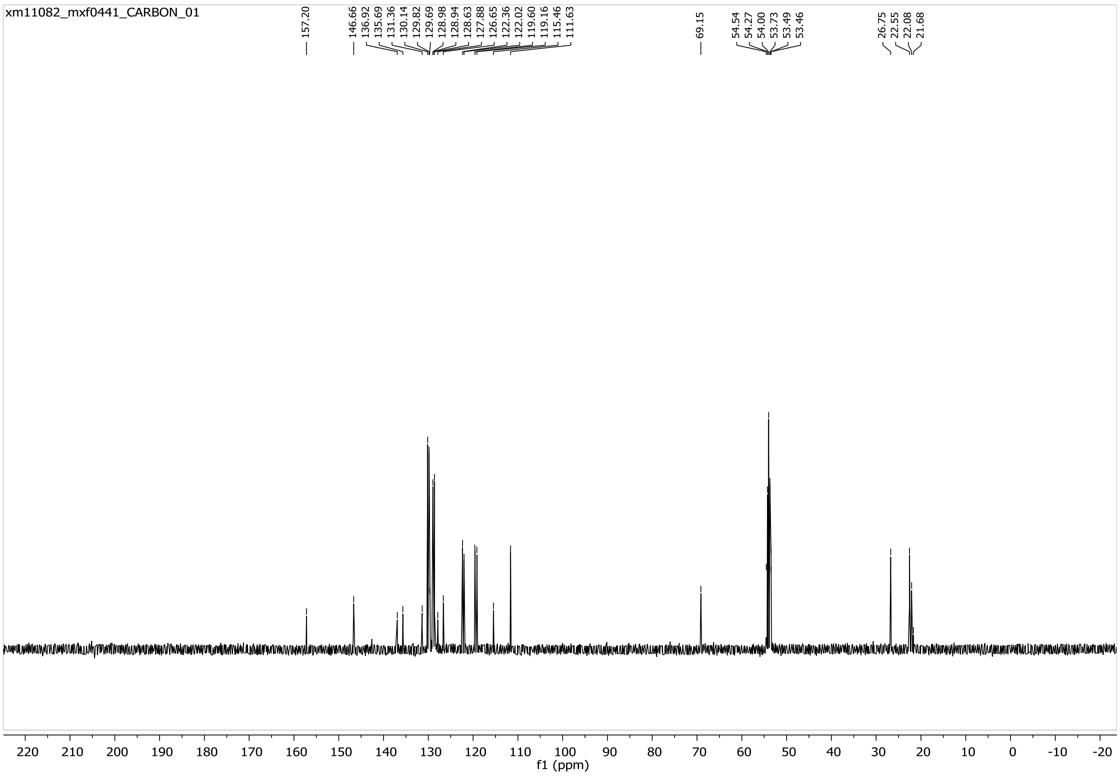

Methyl (3-(1*H*-indol-3-yl)propyl)((perfluorobenzoyl)oxy)carbamate (3d)

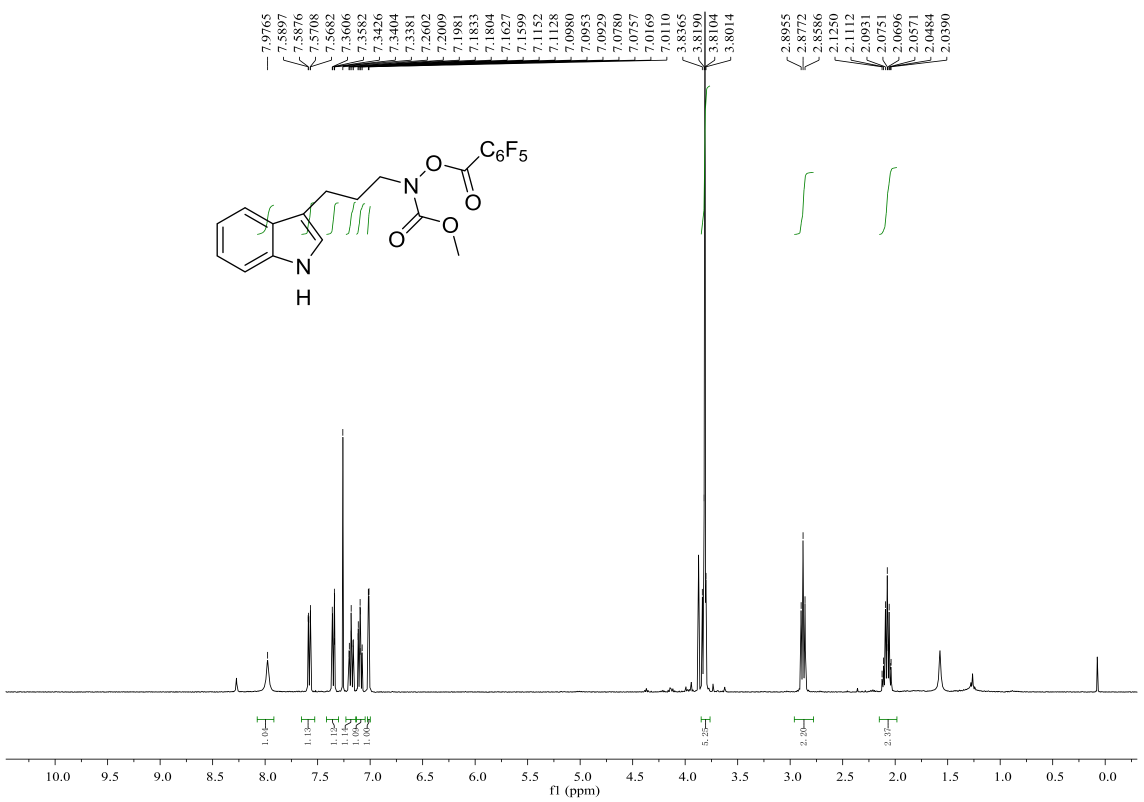

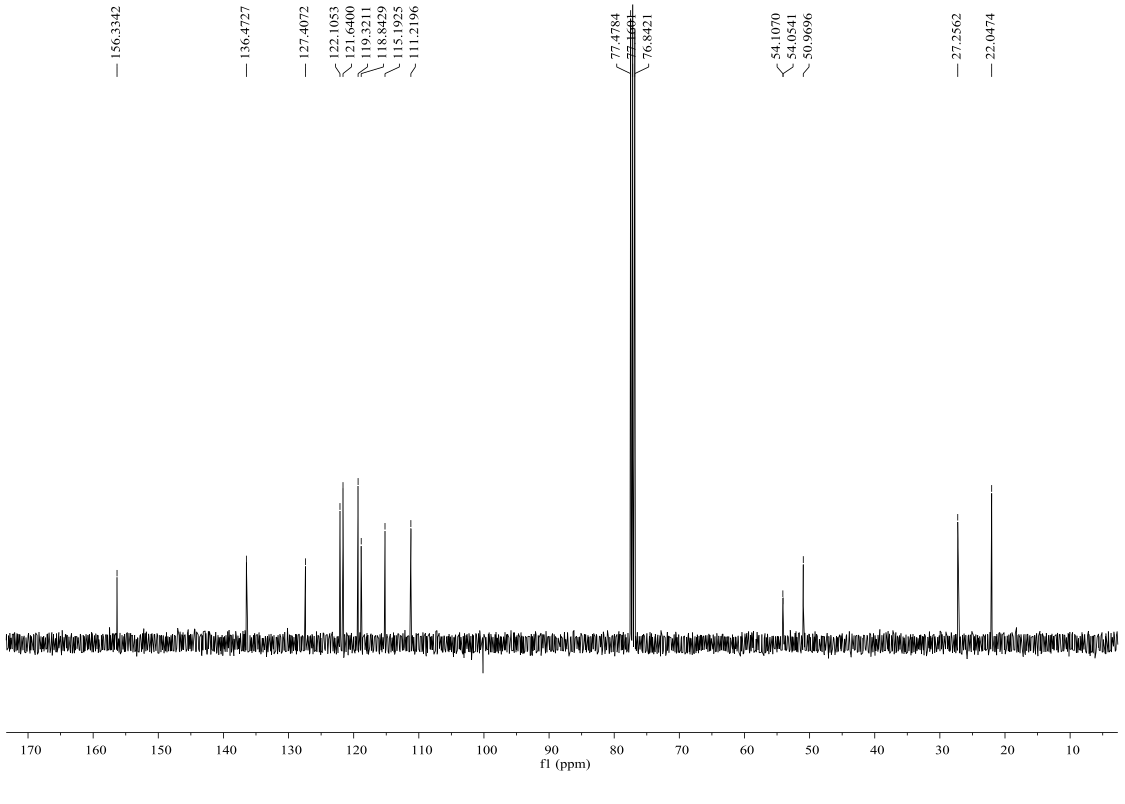

Methyl (3-(1*H*-indol-3-yl)propyl)(tosyloxy)carbamate (3d’)

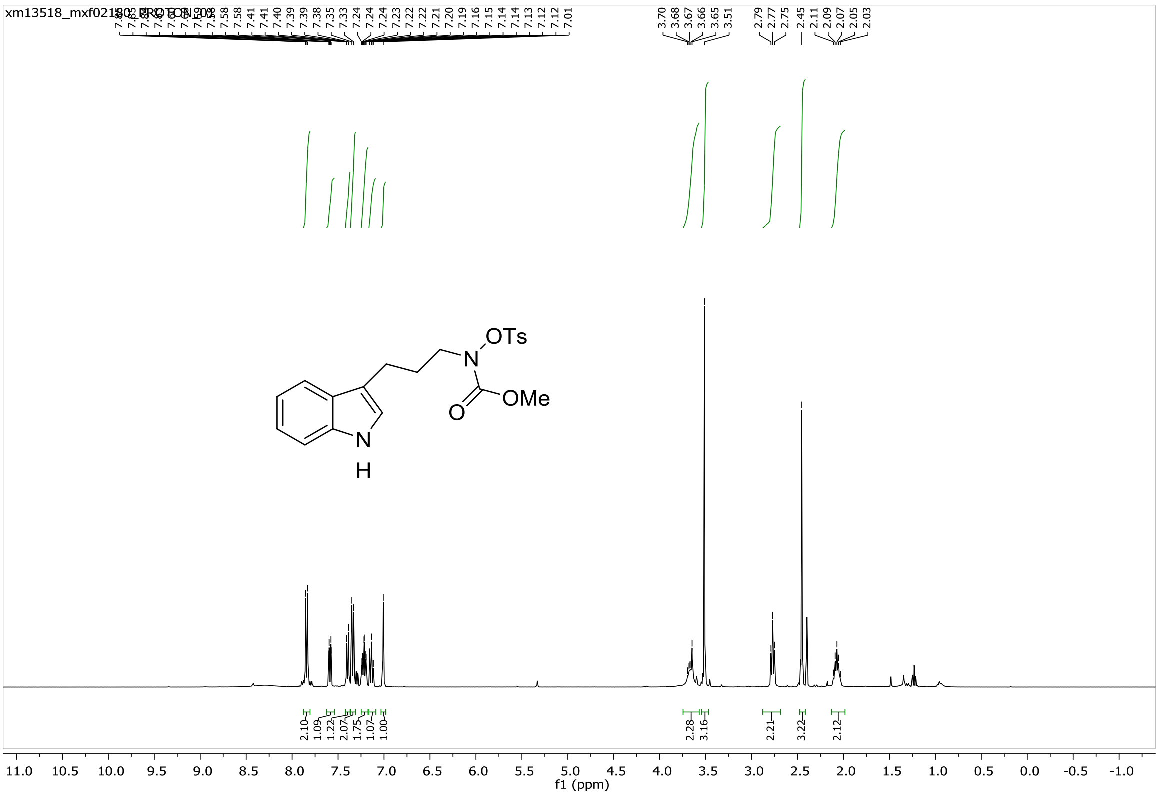

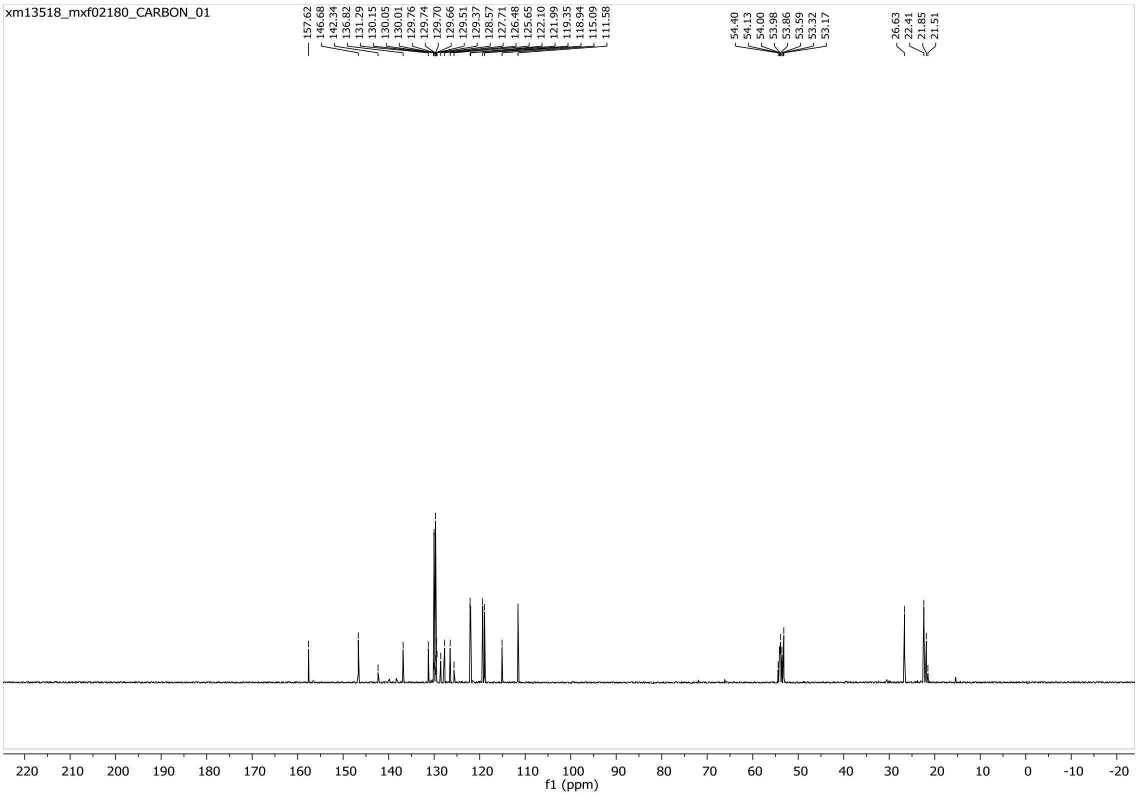

tert-Butyl (3-(1_H_-indol-3-yl)propyl)((perfluorobenzoyl)oxy)carbamate (3e)

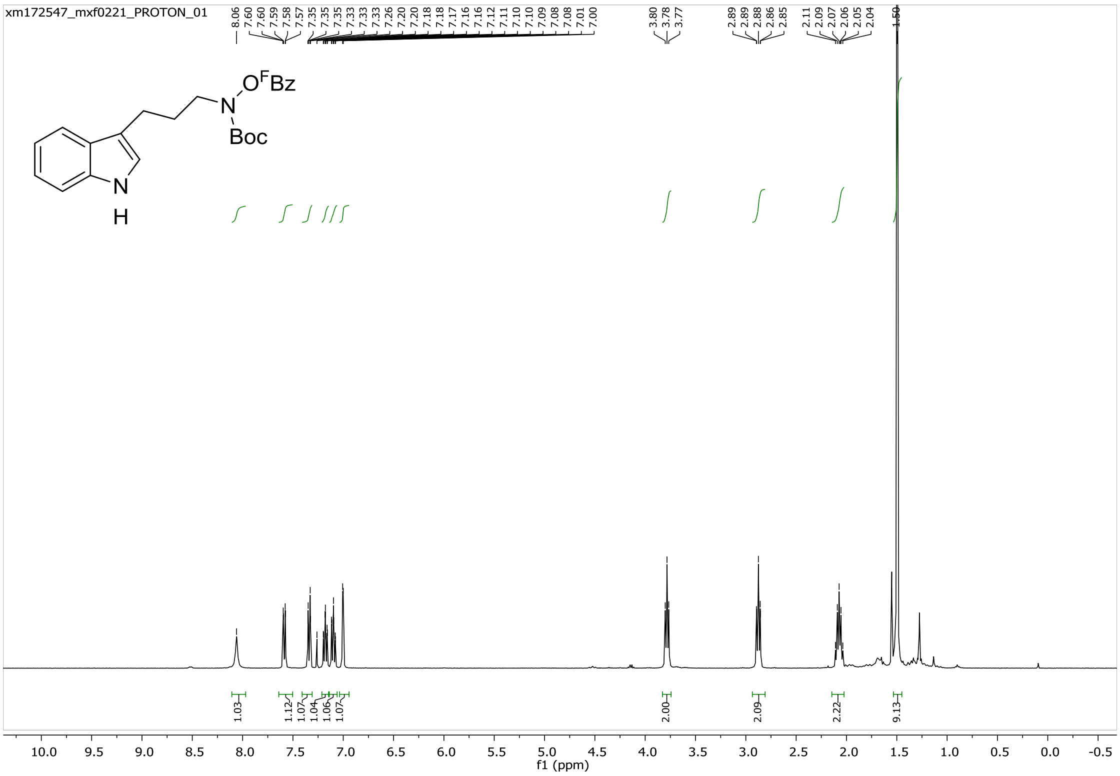

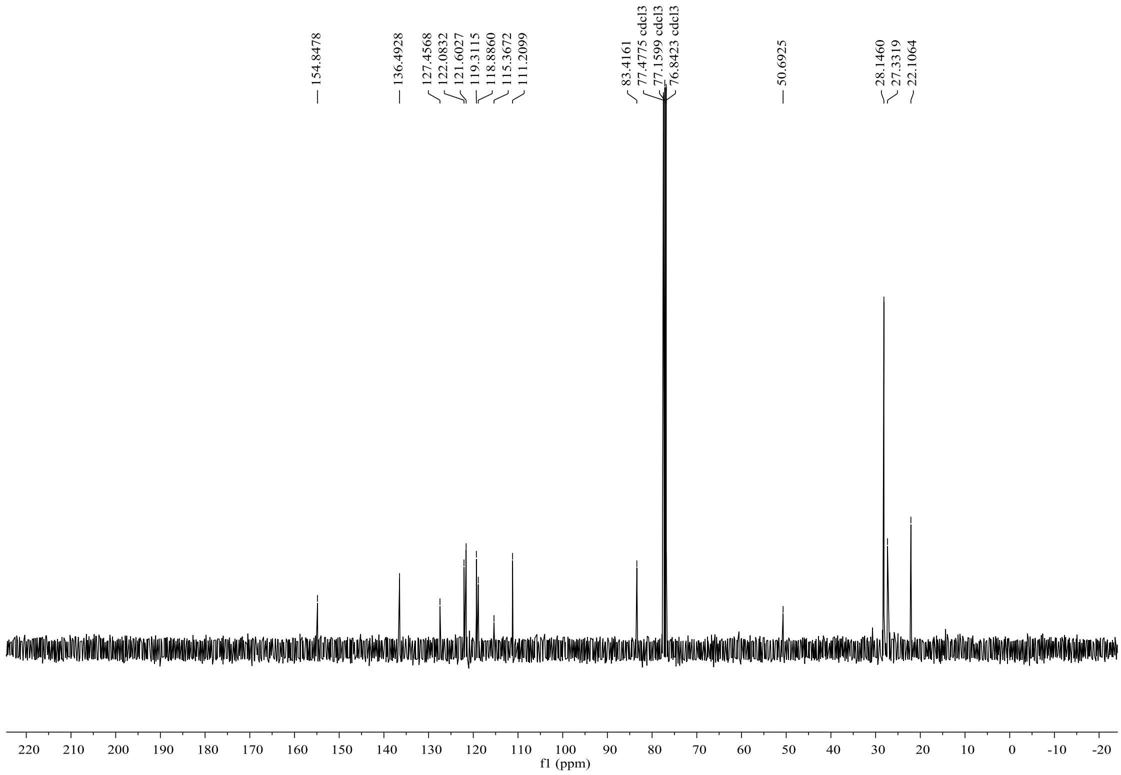

***tert*-Butyl (3-(1*H*-indol-3-yl)propyl)(tosyloxy)carbamate (3e')**

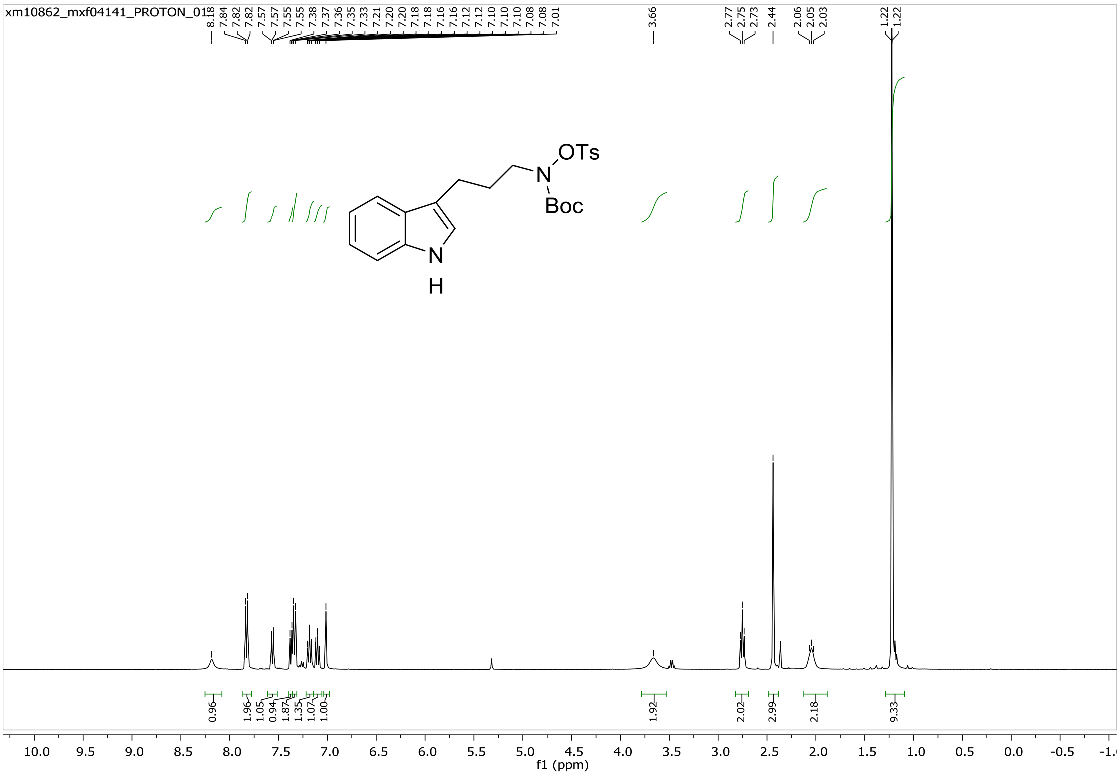

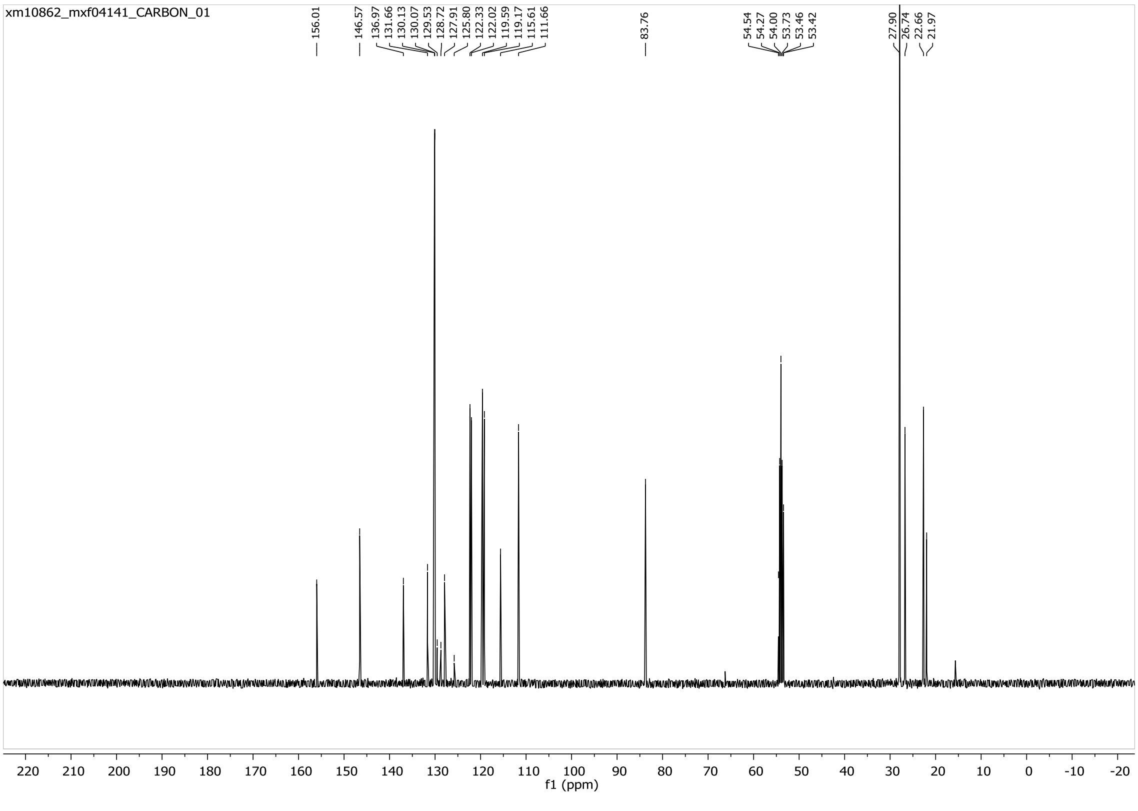

Allyl (3-(1*H*-indol-3-yl)propyl)(tosyloxy)carbamate (3f)

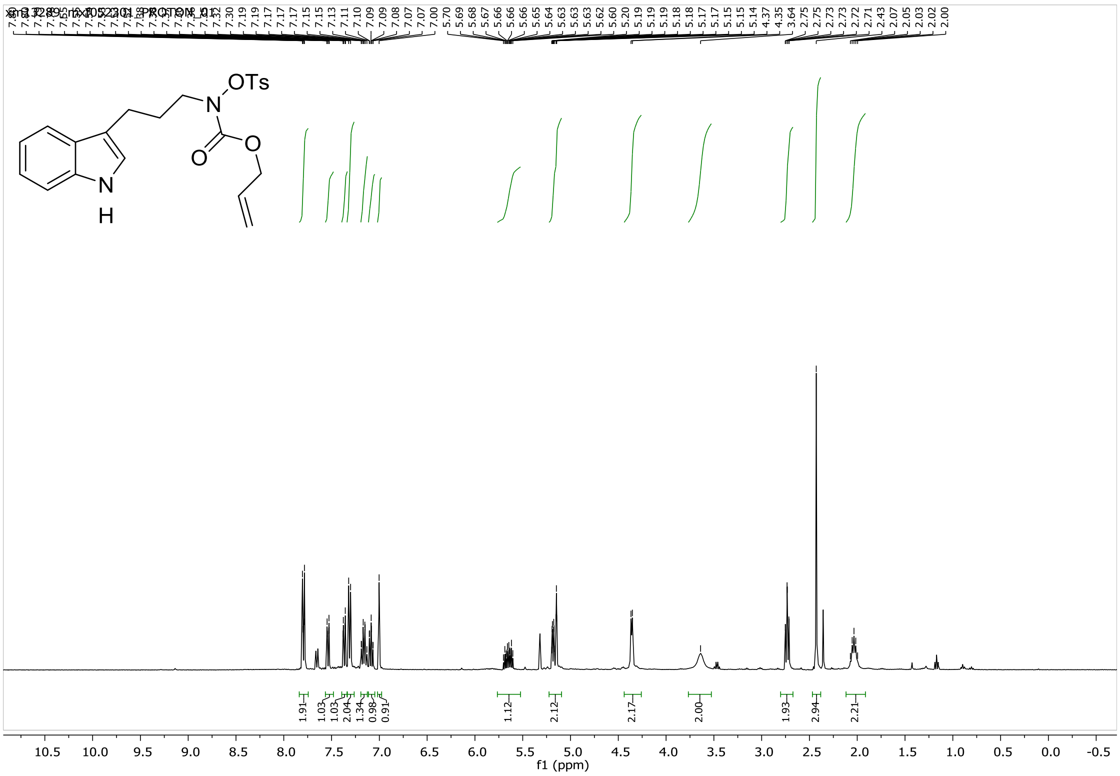

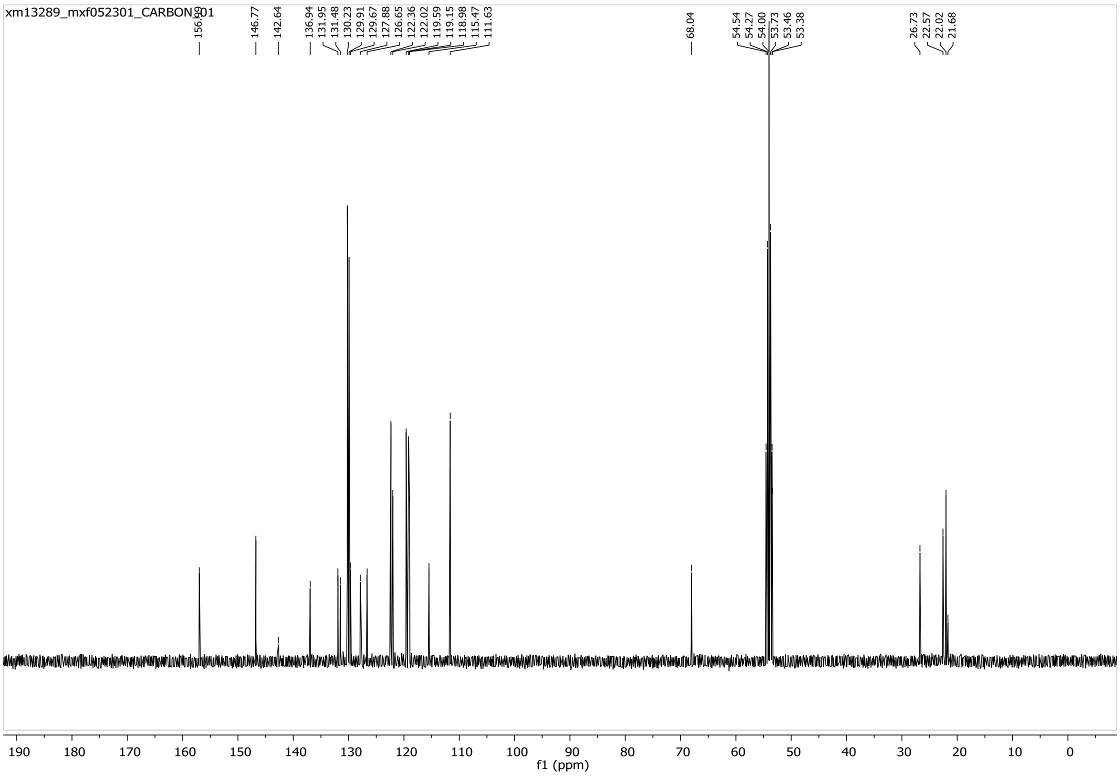

Benzyl (3-(5-methoxy-1*H*-indol-3-yl)propyl)((perfluorobenzoyl)oxy)carbamate (3g)

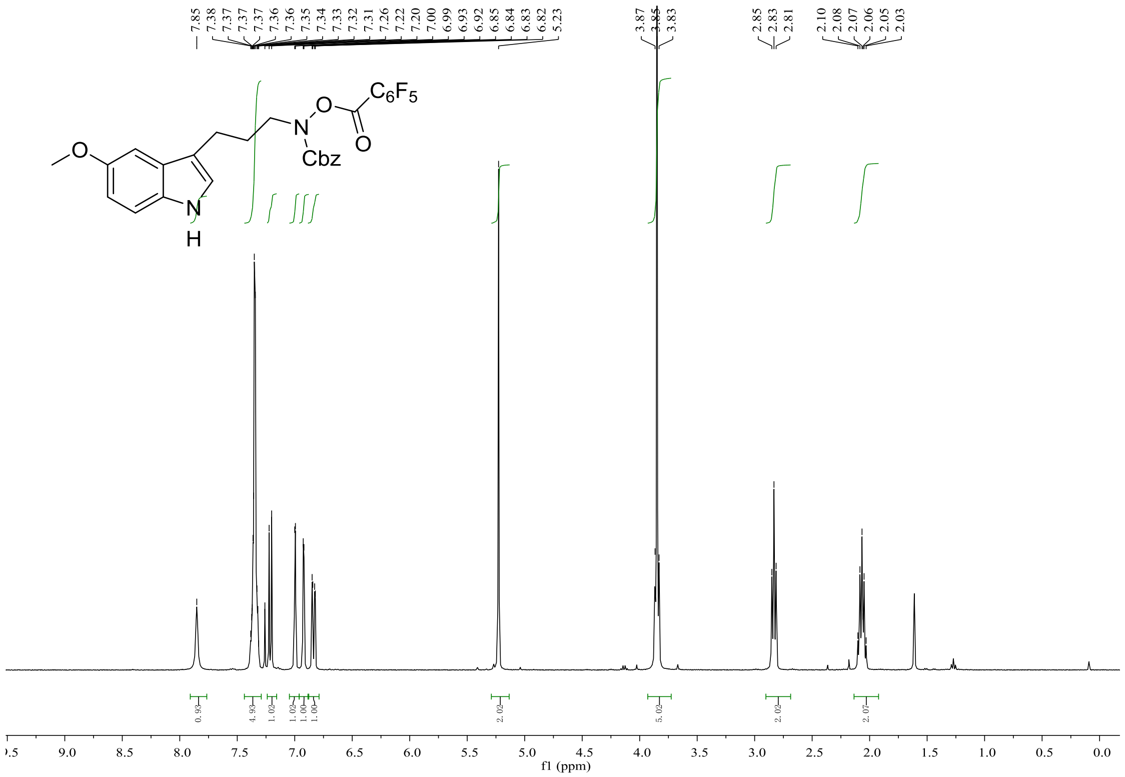

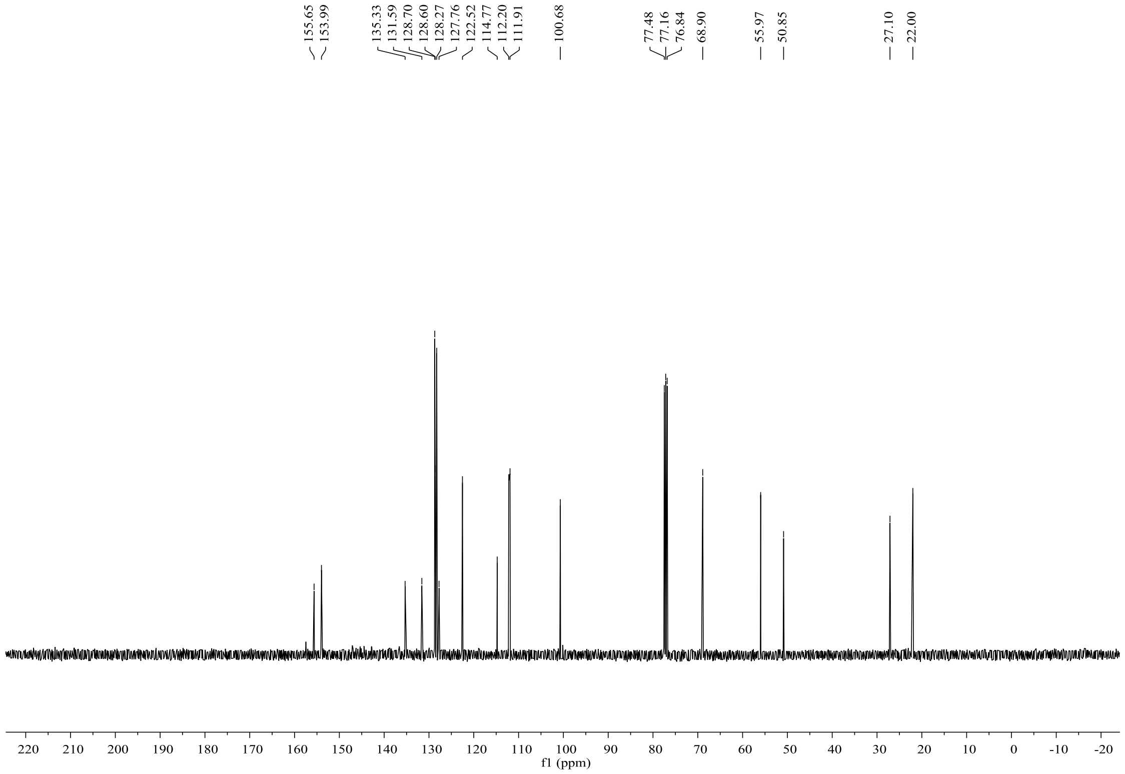

Benzyl (3-(5-methyl-1*H*-indol-3-yl)propyl)((perfluorobenzoyl)oxy)carbamate (3h)

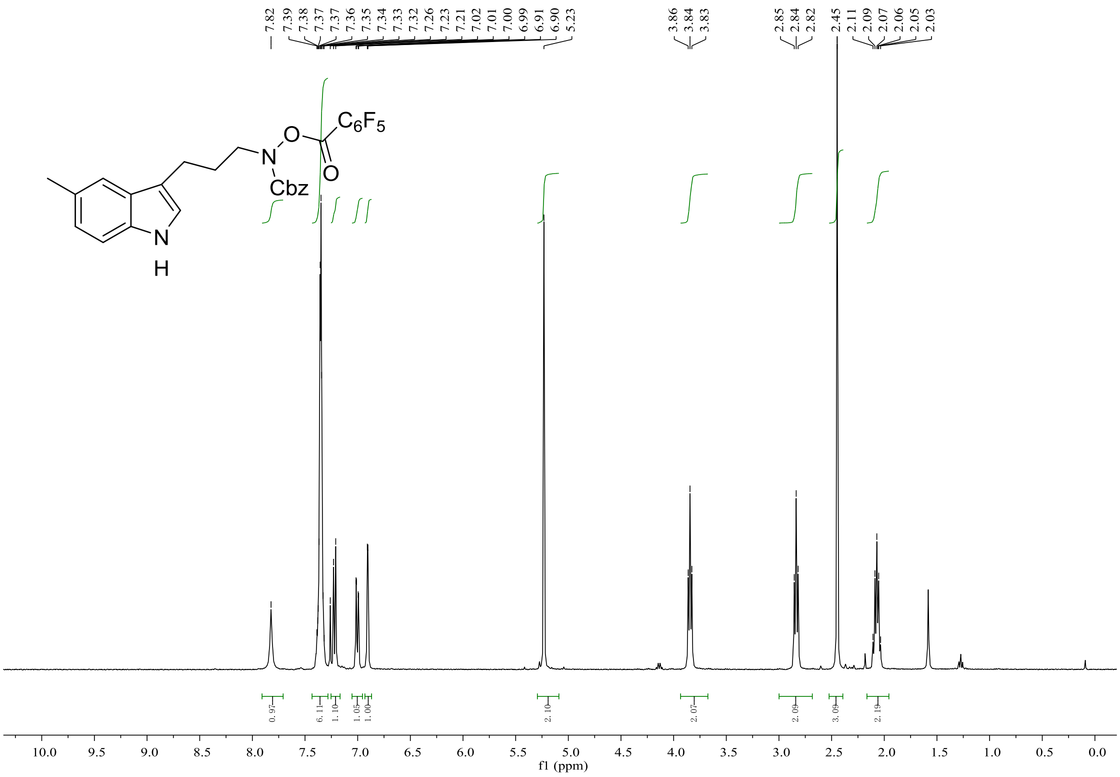

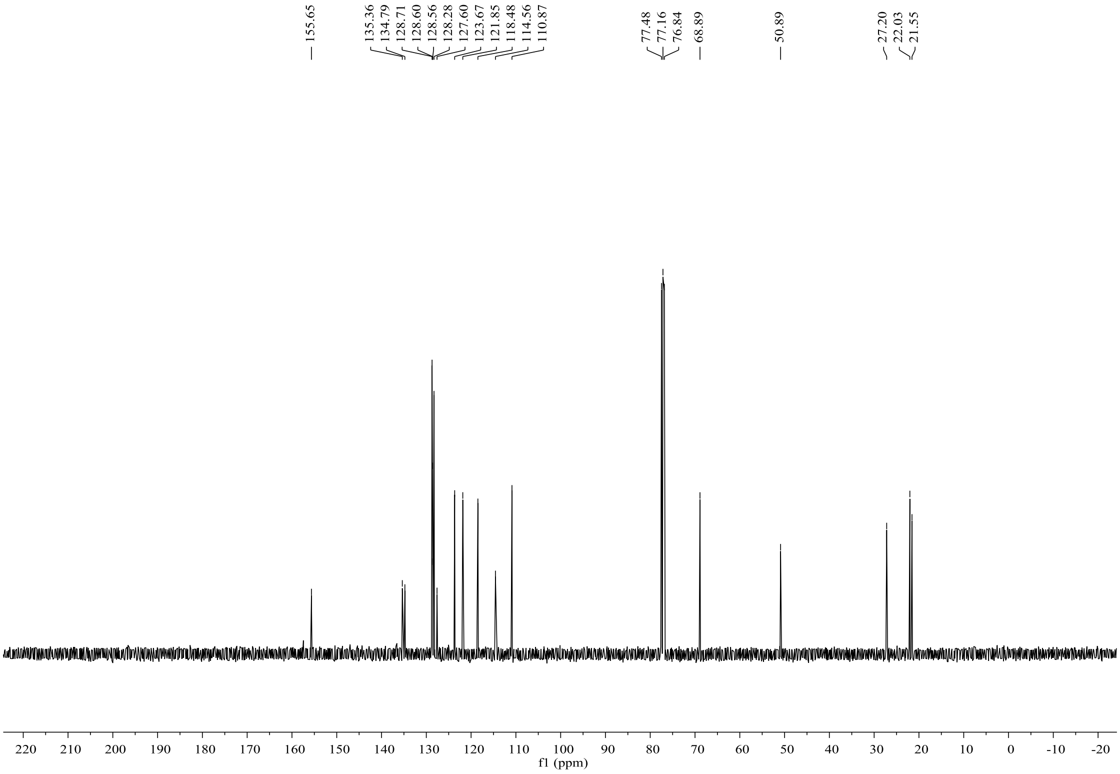

Benzyl (3-(5-fluoro-1*H*-indol-3-yl)propyl)((perfluorobenzoyl)oxy)carbamate (3i)

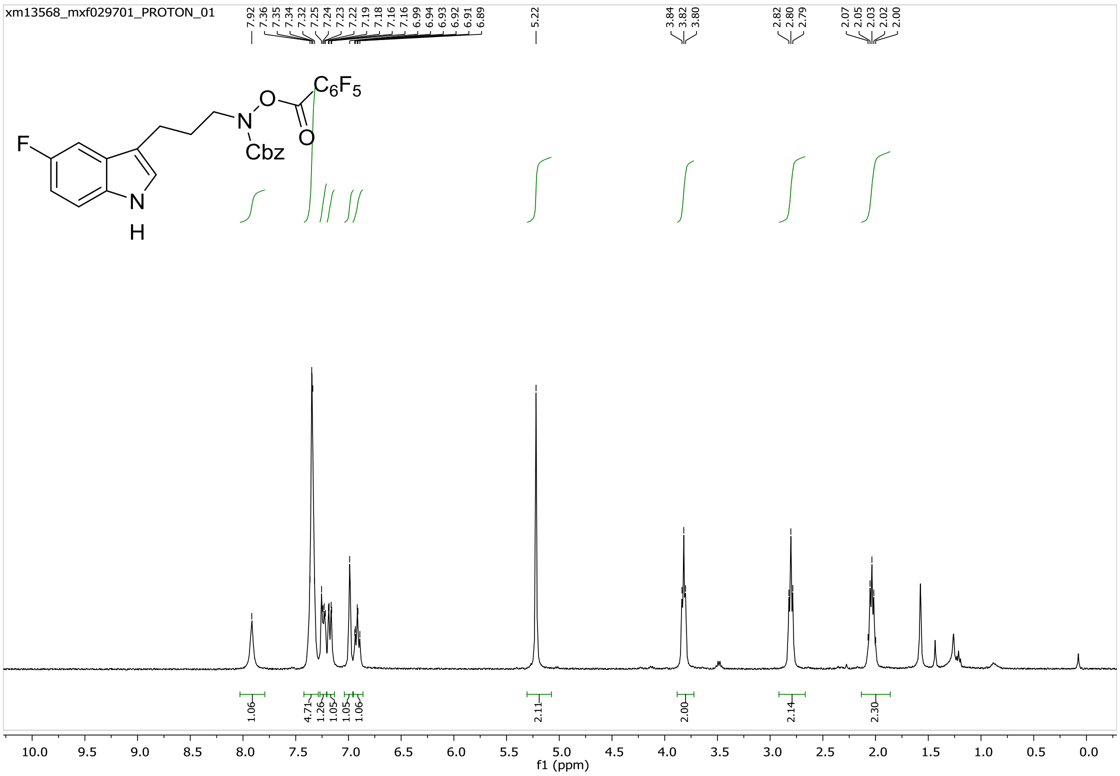

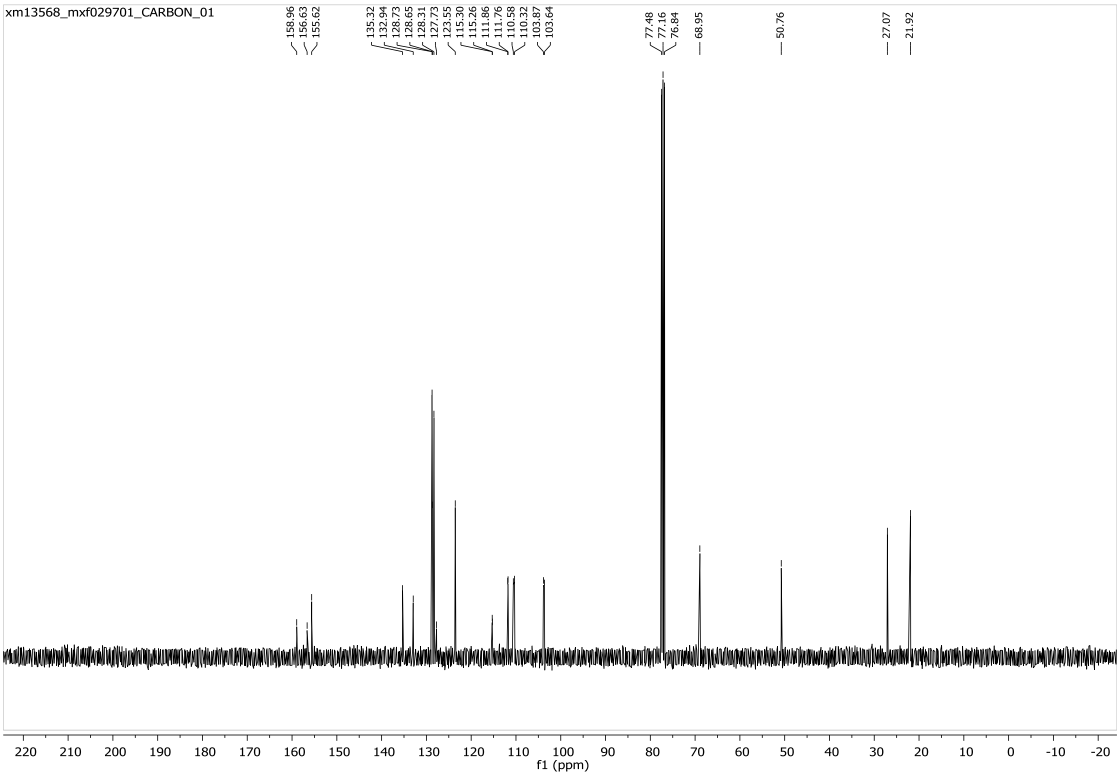

***tert*-Butyl (3-(5-fluoro-1*H*-indol-3-yl)propyl)(tosyloxy)carbamate (3j)**

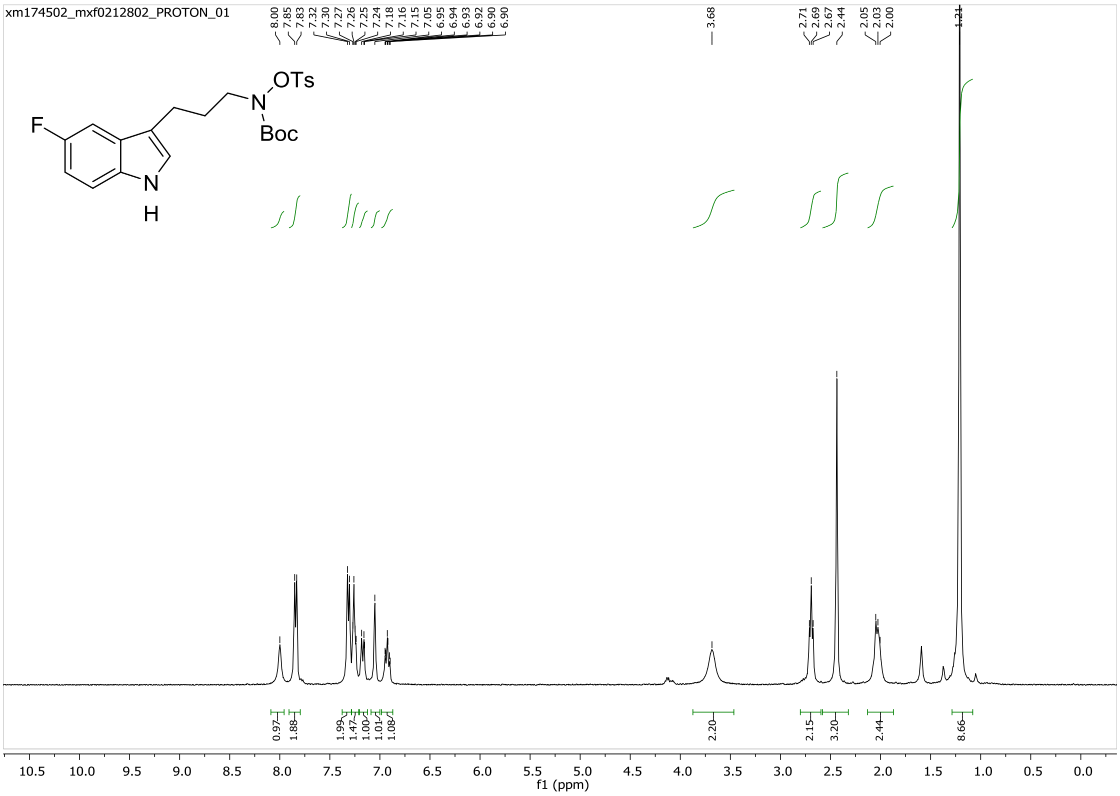

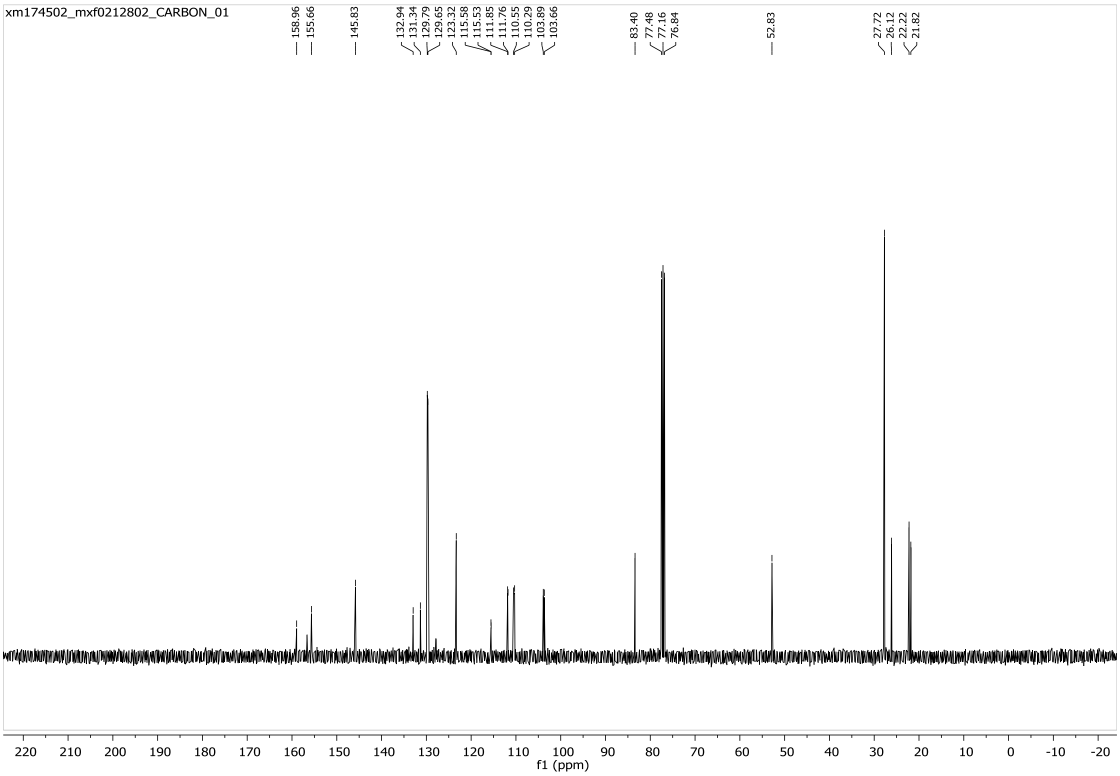

Benzyl (3-(5-chloro-1*H*-indol-3-yl)propyl)((perfluorobenzoyl)oxy)carbamate (3k)

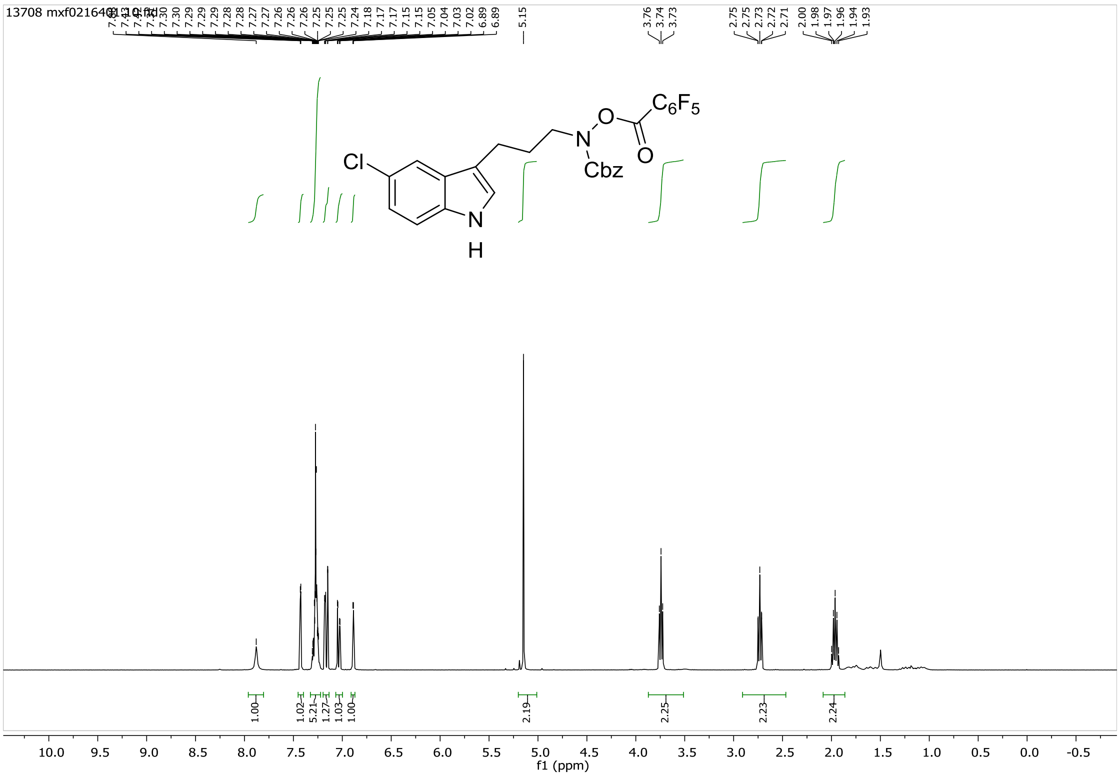

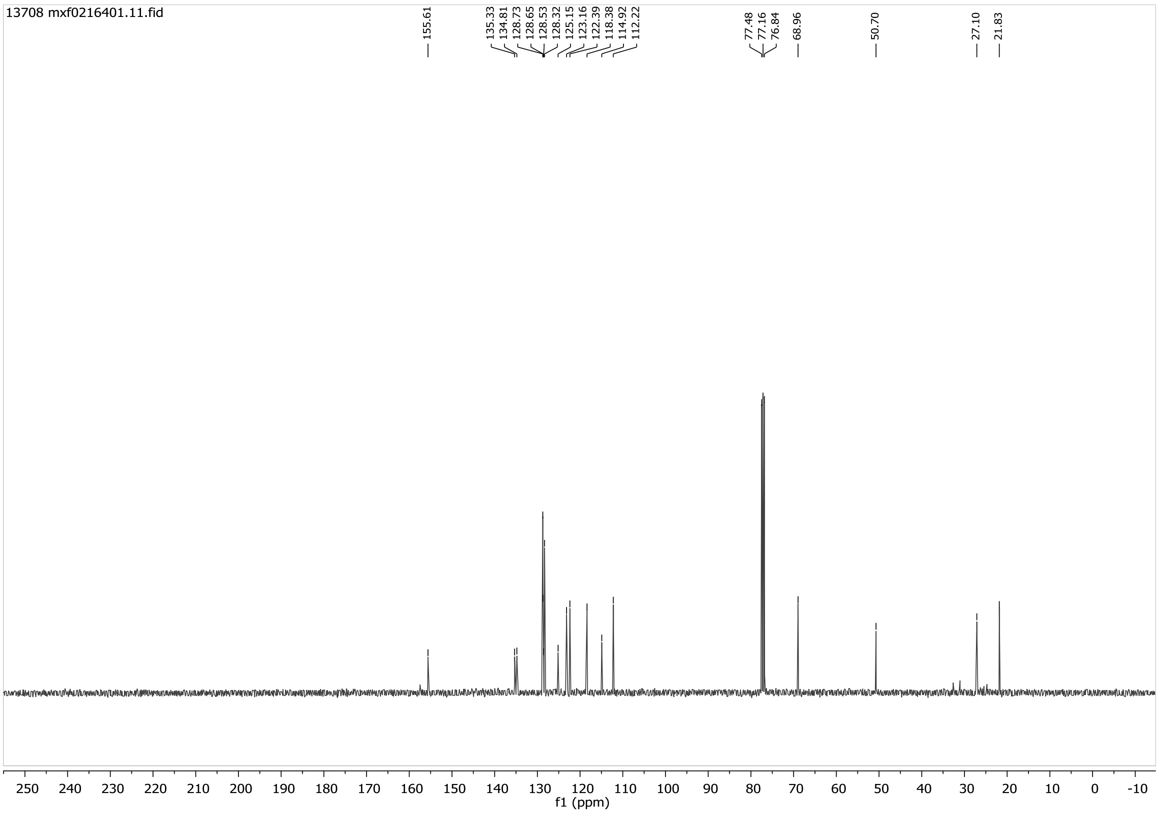

Benzyl (3-(5-bromo-1*H*-indol-3-yl)propyl)((perfluorobenzoyl)oxy)carbamate (3l)

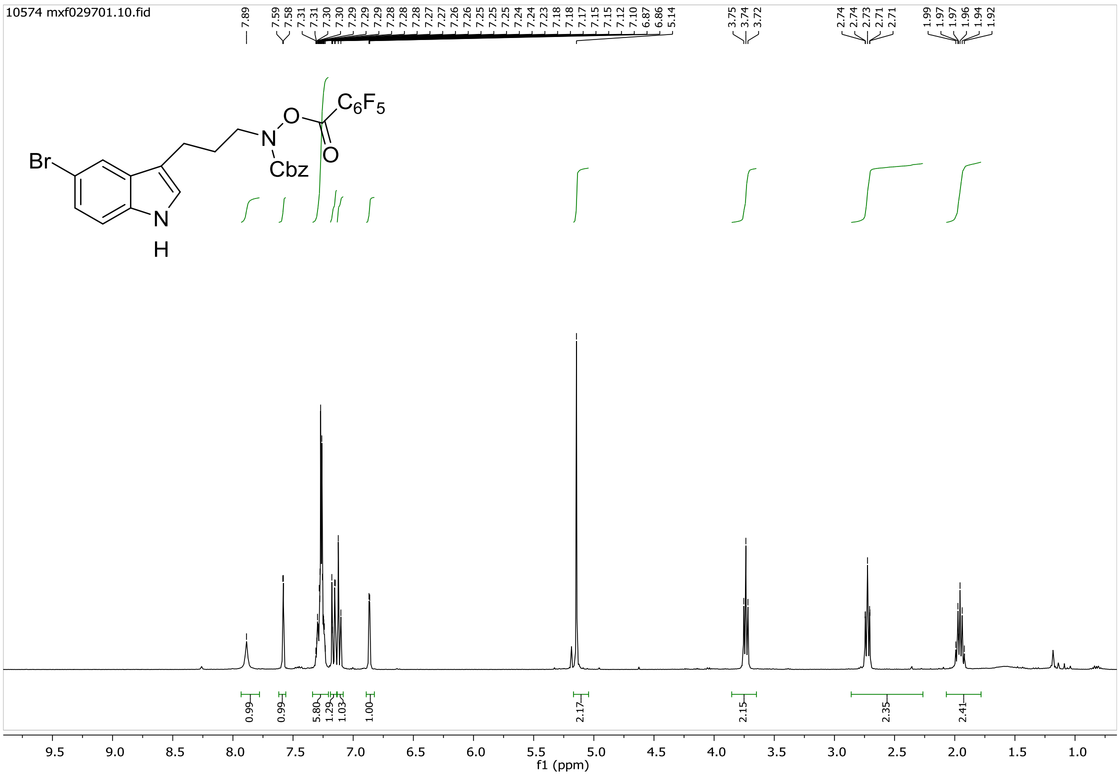

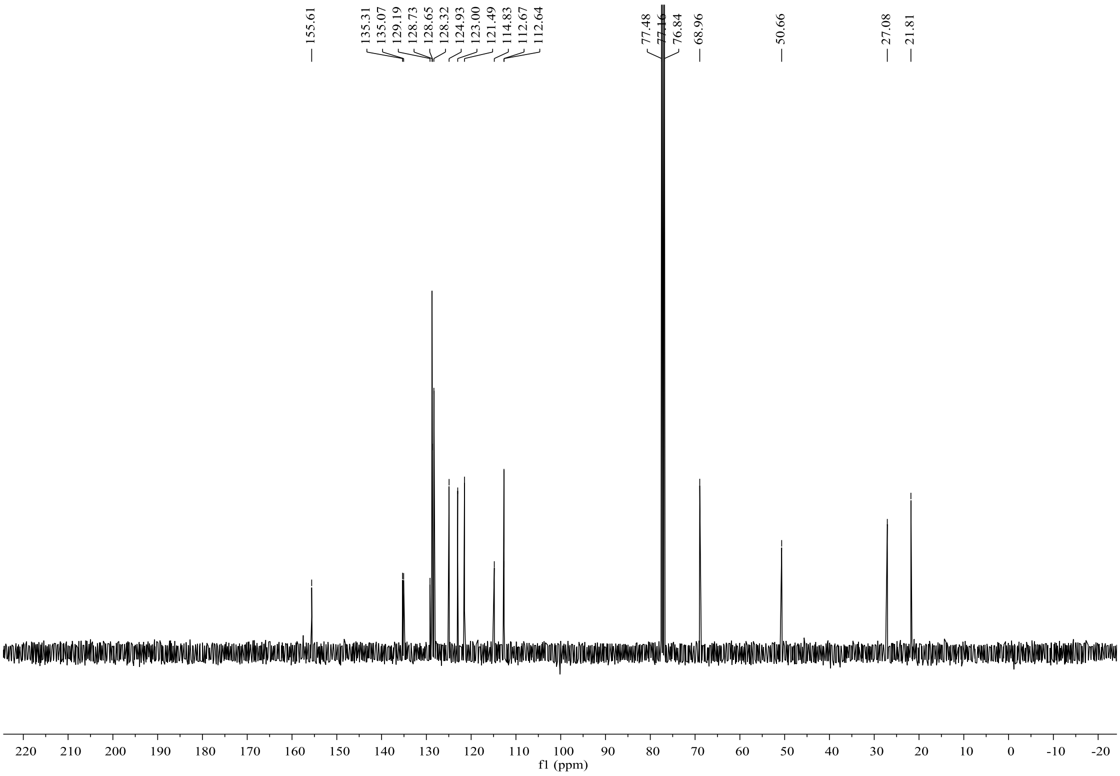

tert-Butyl (3-(5-iodo-1_H_-indol-3-yl)propyl)(tosyloxy)carbamate (3m)

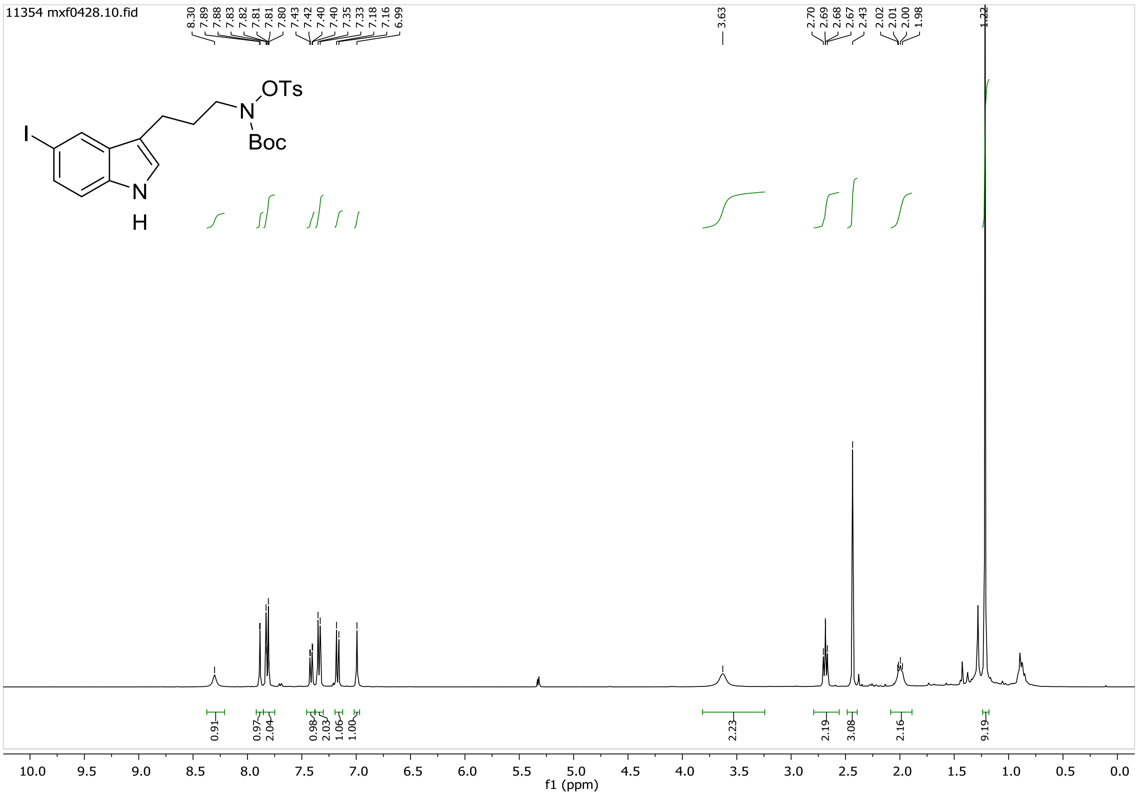

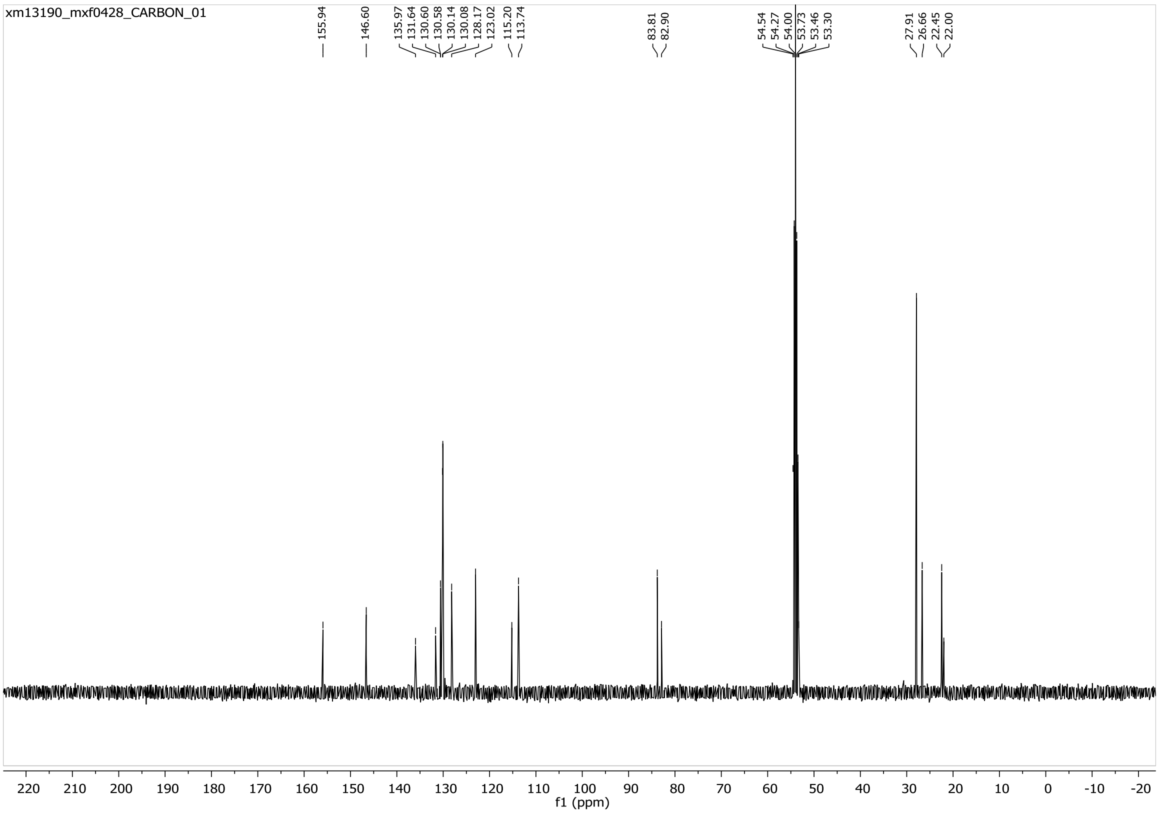

Benzyl (3-(2-methyl-1*H*-indol-3-yl)propyl)((perfluorobenzoyl)oxy)carbamate (3n)

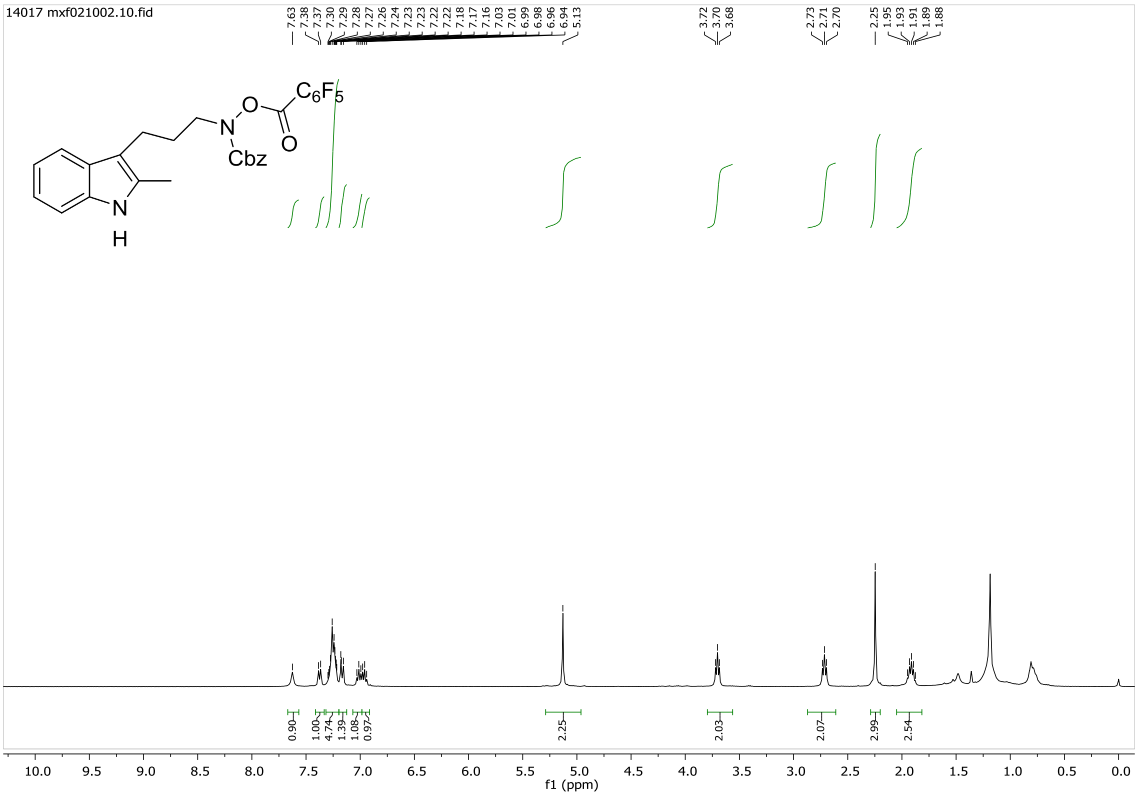

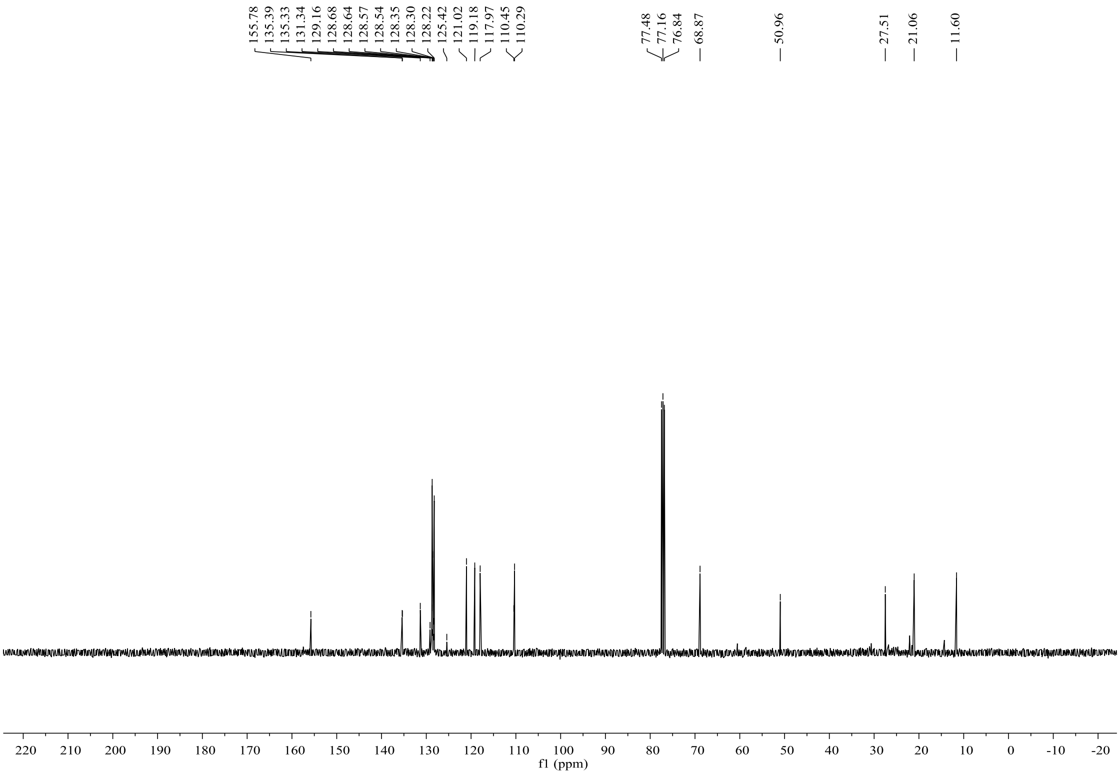

Benzyl (3-(5,7-dimethyl-1*H*-indol-3-yl)propyl)((perfluorobenzoyl)oxy)carbamate (3o)

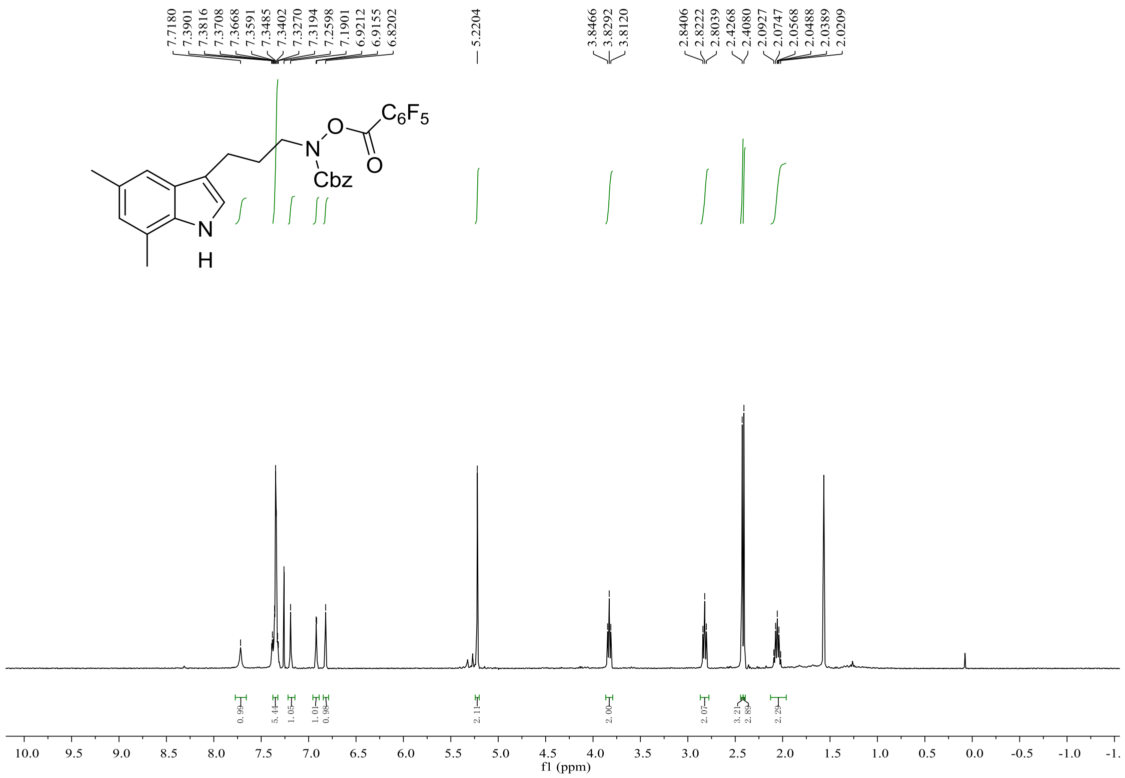

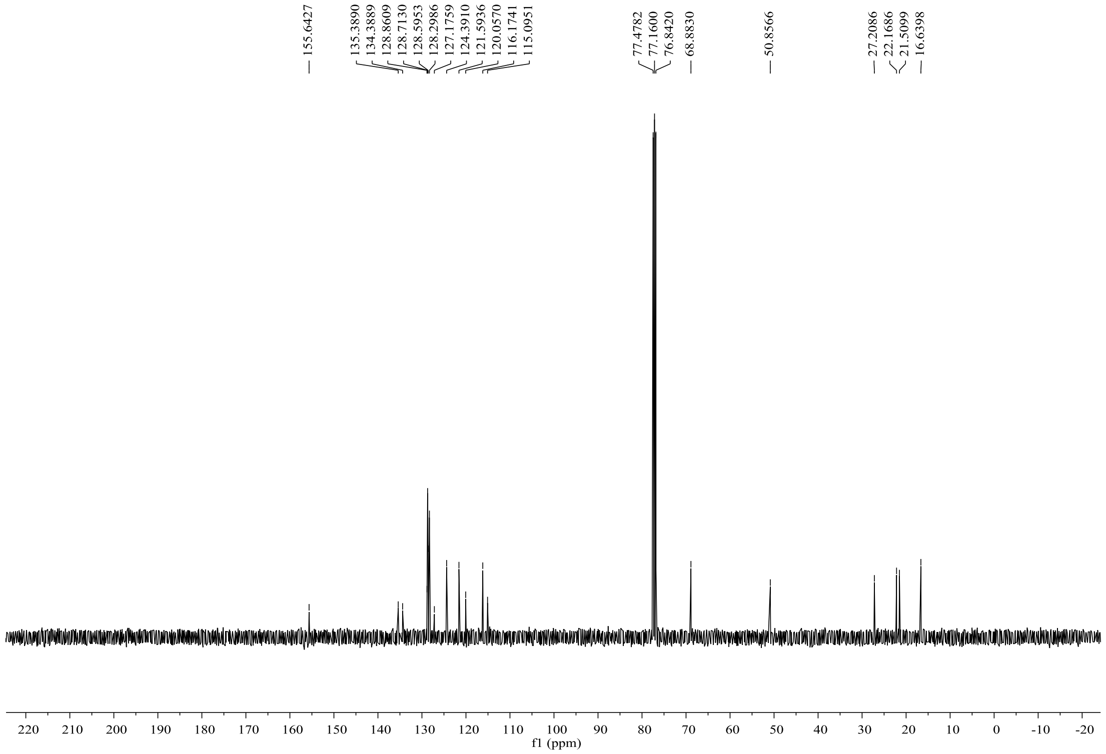

Benzyl (3-(7-methyl-1*H*-indol-3-yl)propyl)((perfluorobenzoyl)oxy)carbamate (3p)

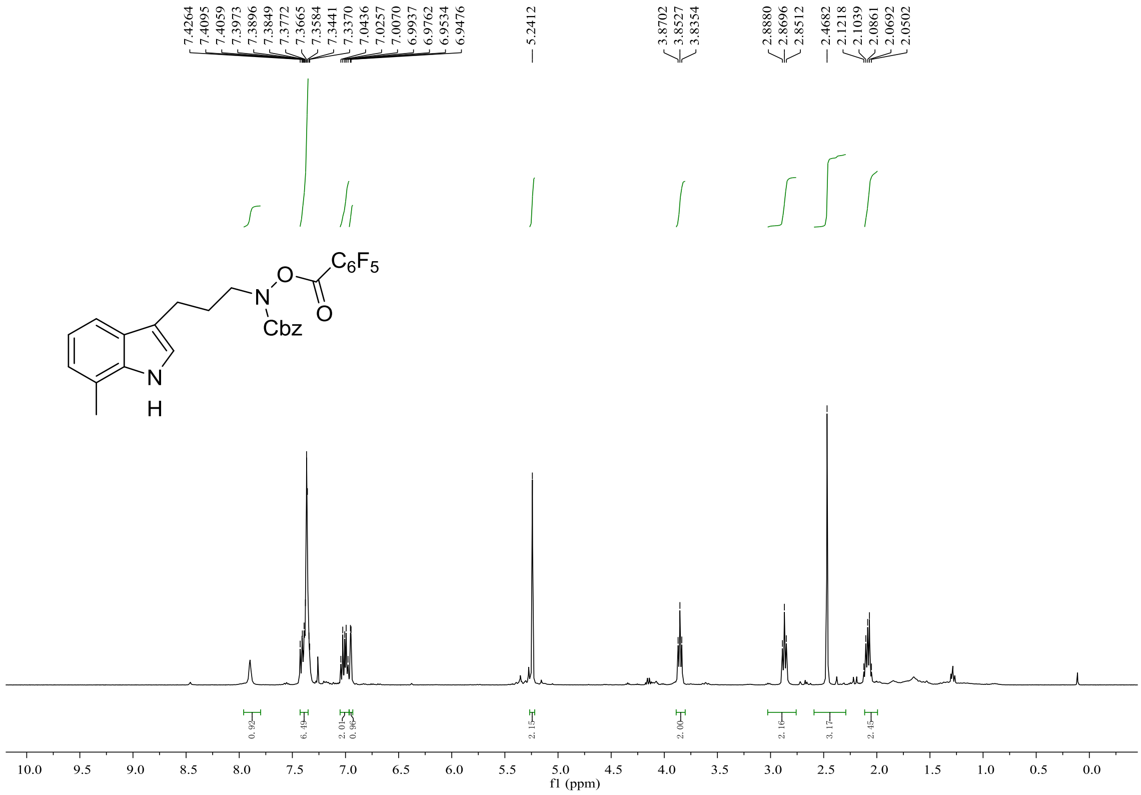

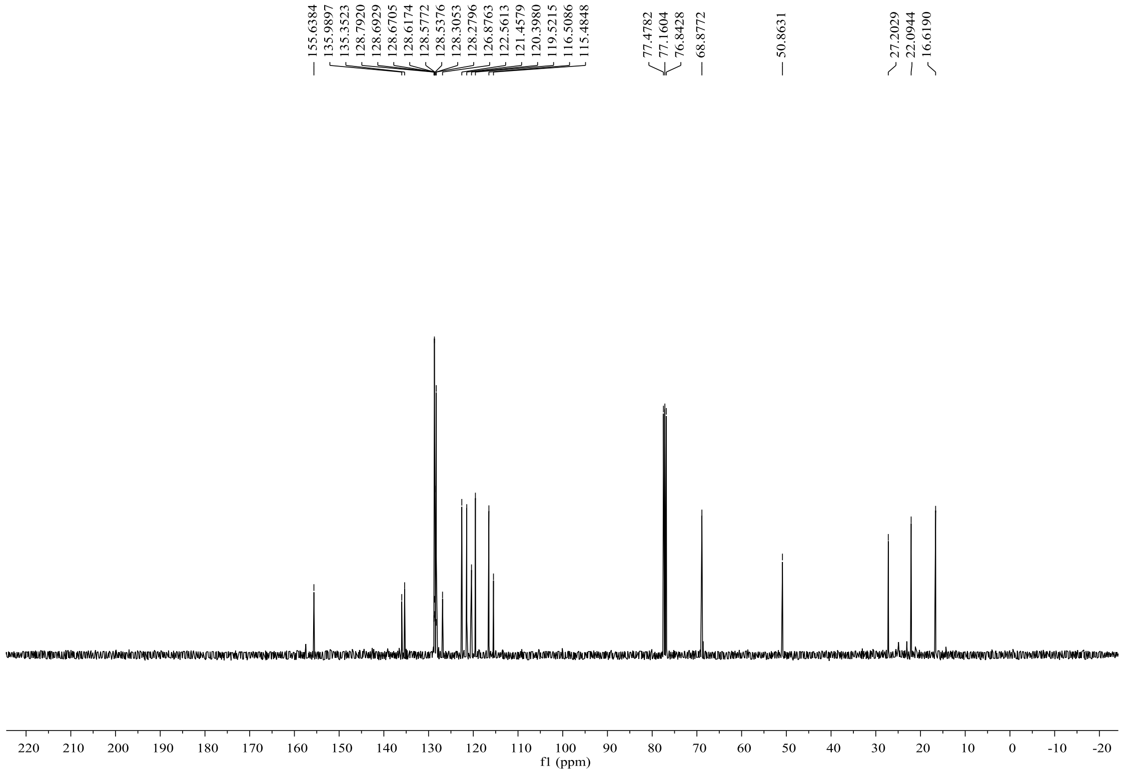

tert-Butyl (4-(1H-indol-3-yl)butan-2-yl)(tosyloxy)carbamate (3r)

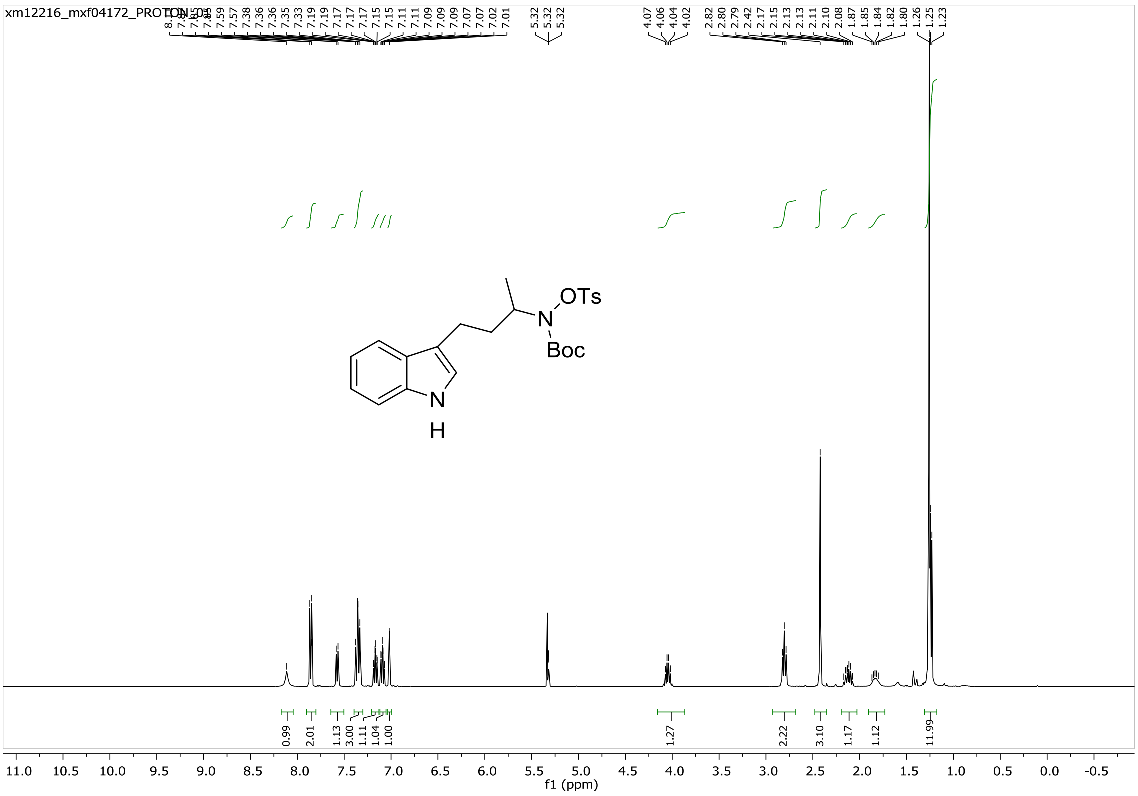

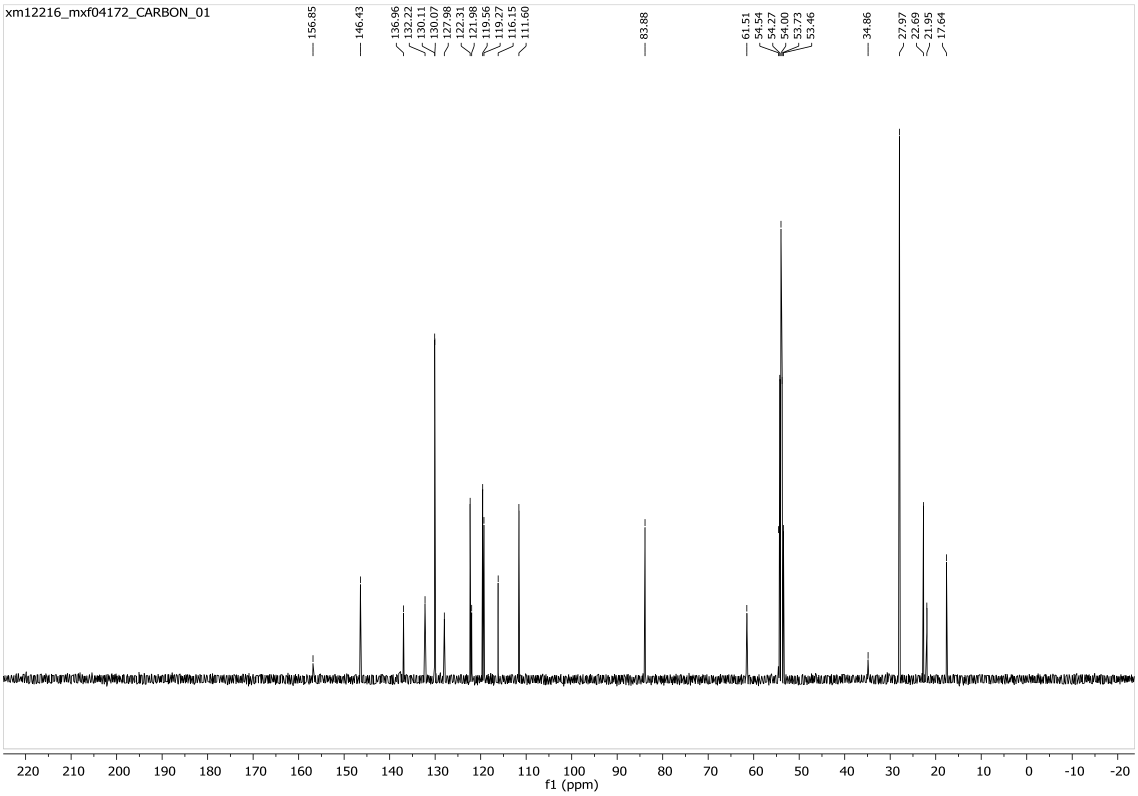

***tert*-Butyl (*R*)-(3-(1*H*-indol-3-yl)butyl)(tosyloxy)carbamate (3s)**

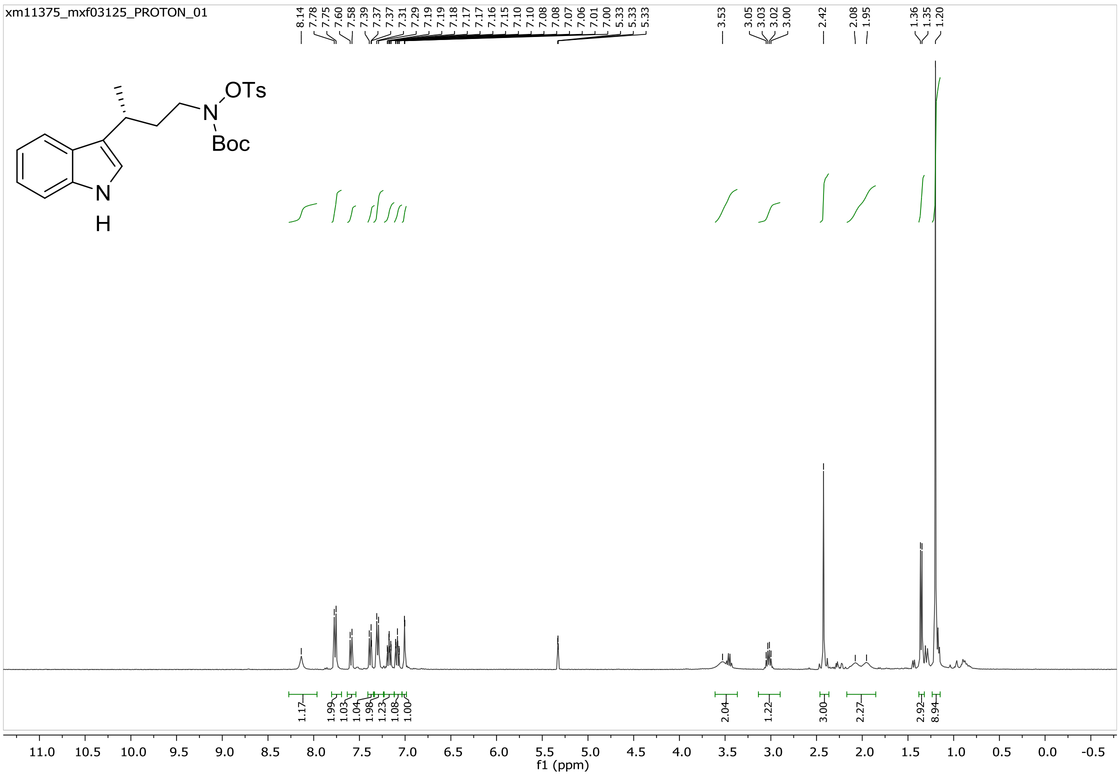

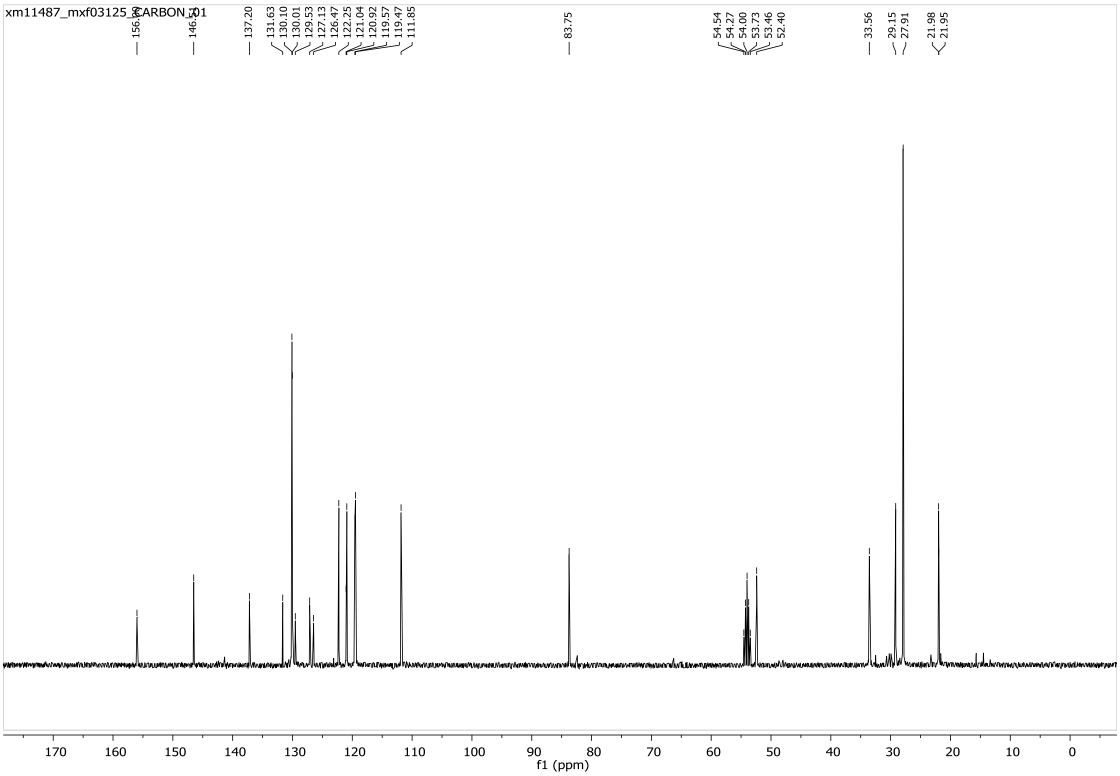

tert-Butyl (3-(1H-indol-3-yl)-4-methylpentyl)(tosyloxy)carbamate (3t)

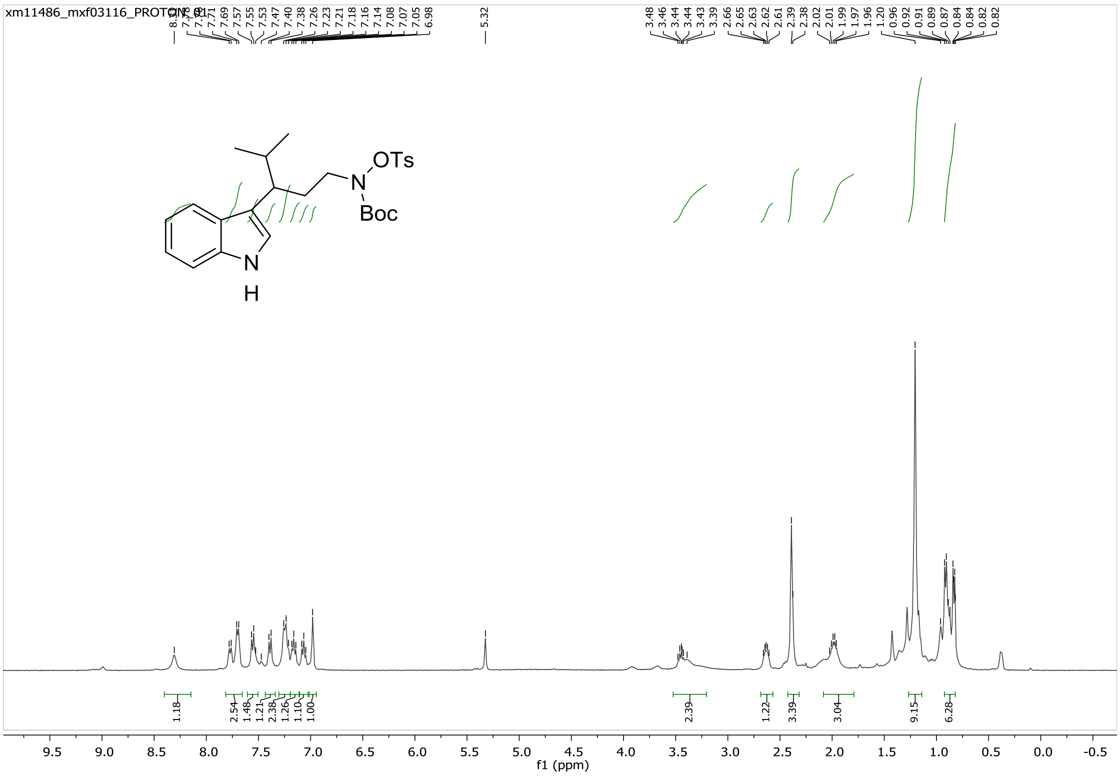

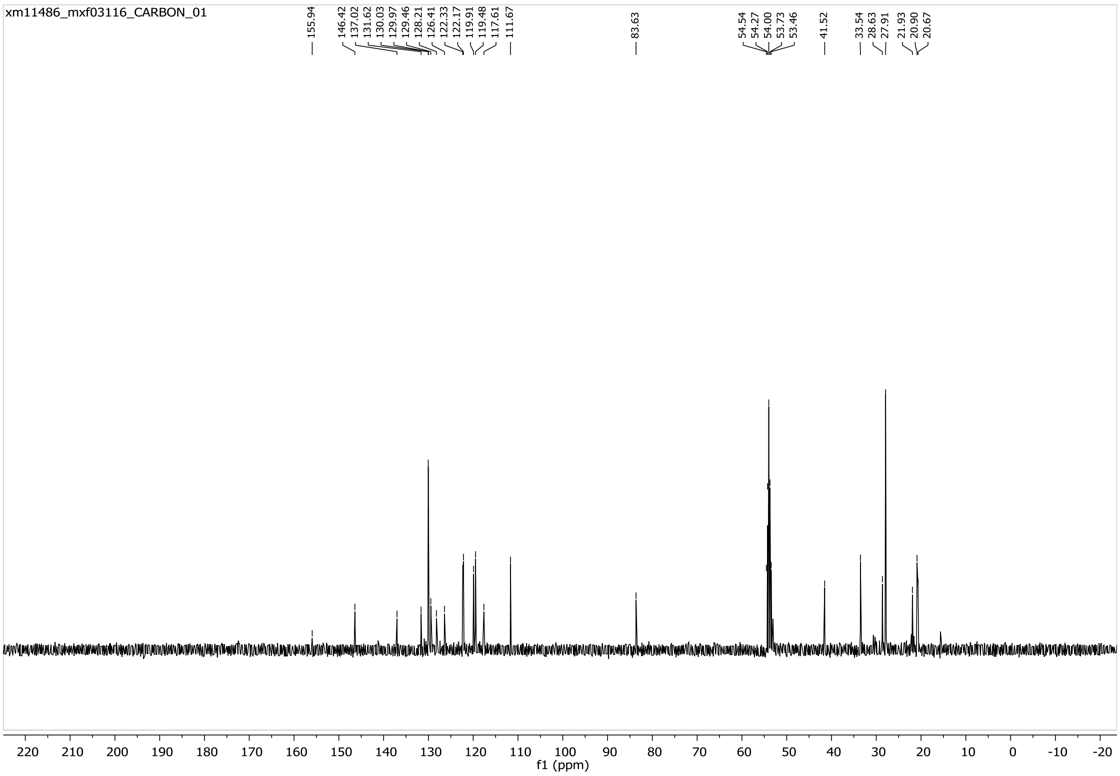

tert-Butyl (3-(1*H*-indol-3-yl)-3-phenylpropyl)(tosyloxy)carbamate (3u)

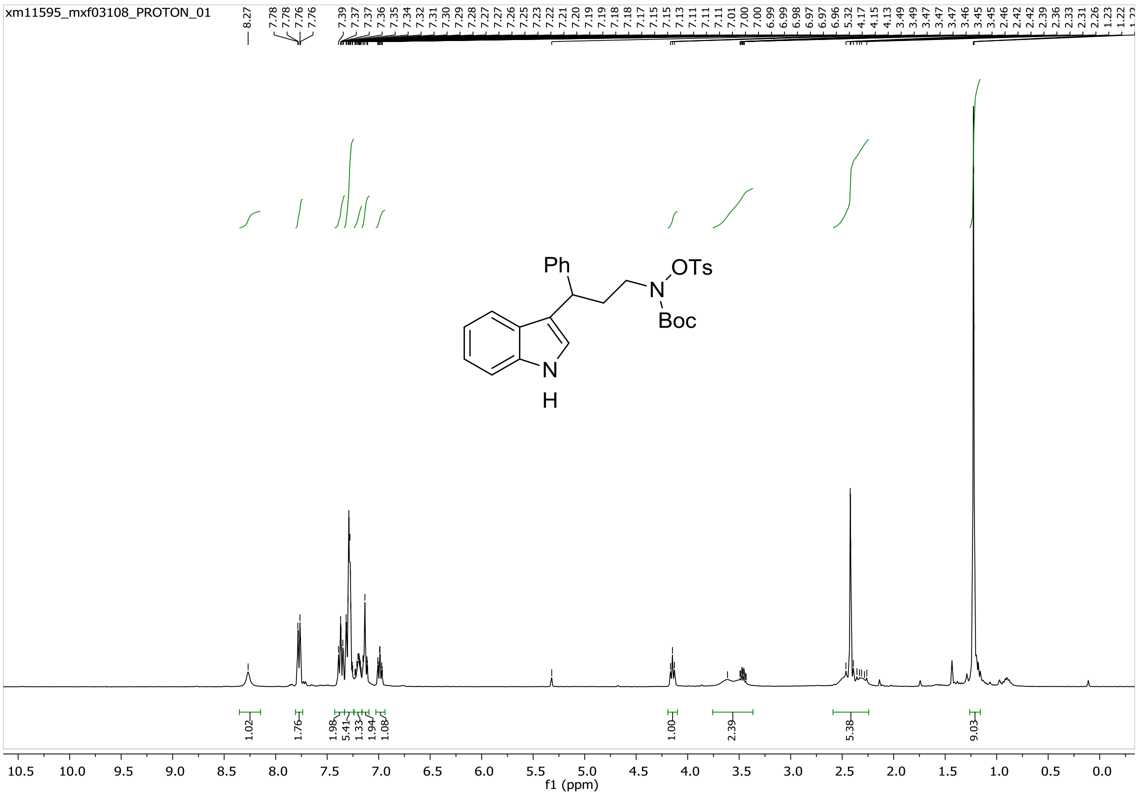

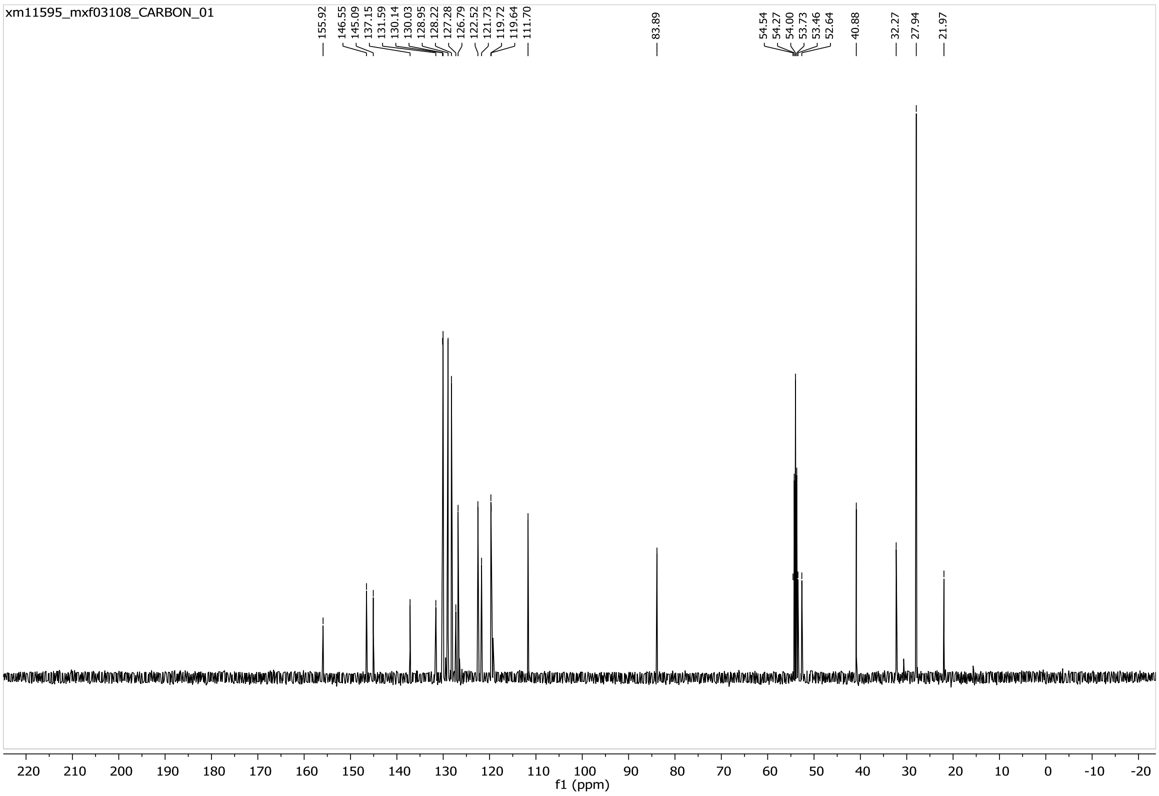

1'-Tosylspiro[indole-3,2'-pyrrolidine] (4a)

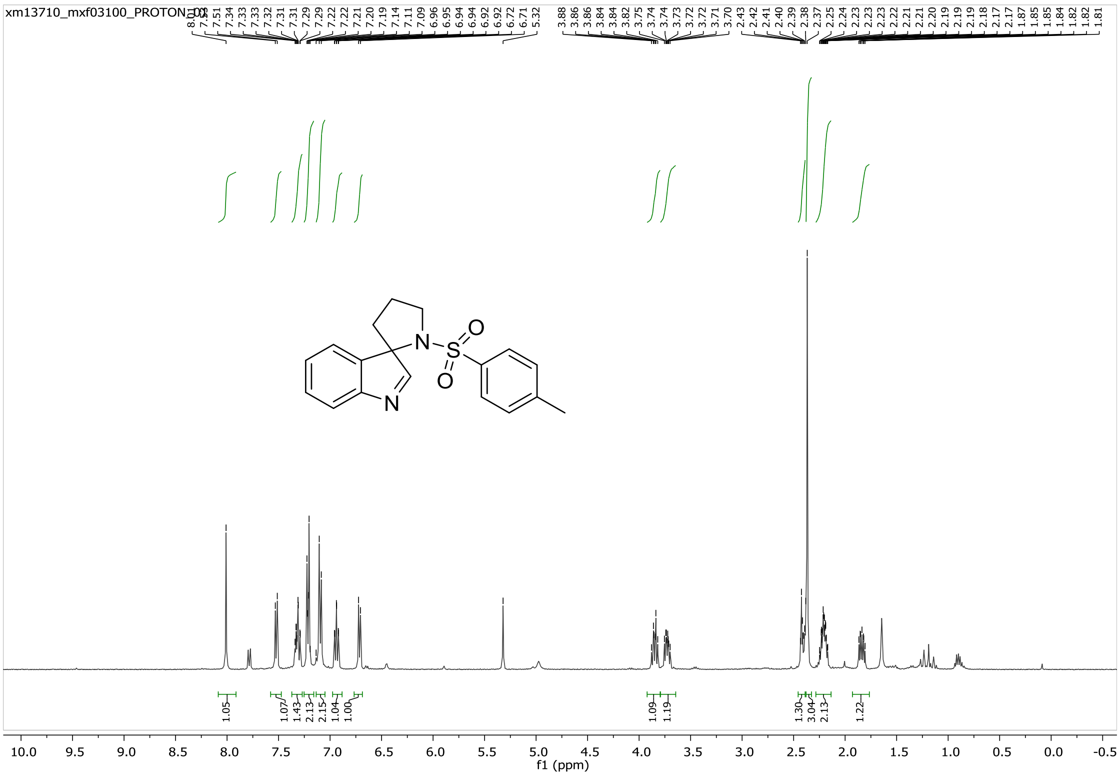

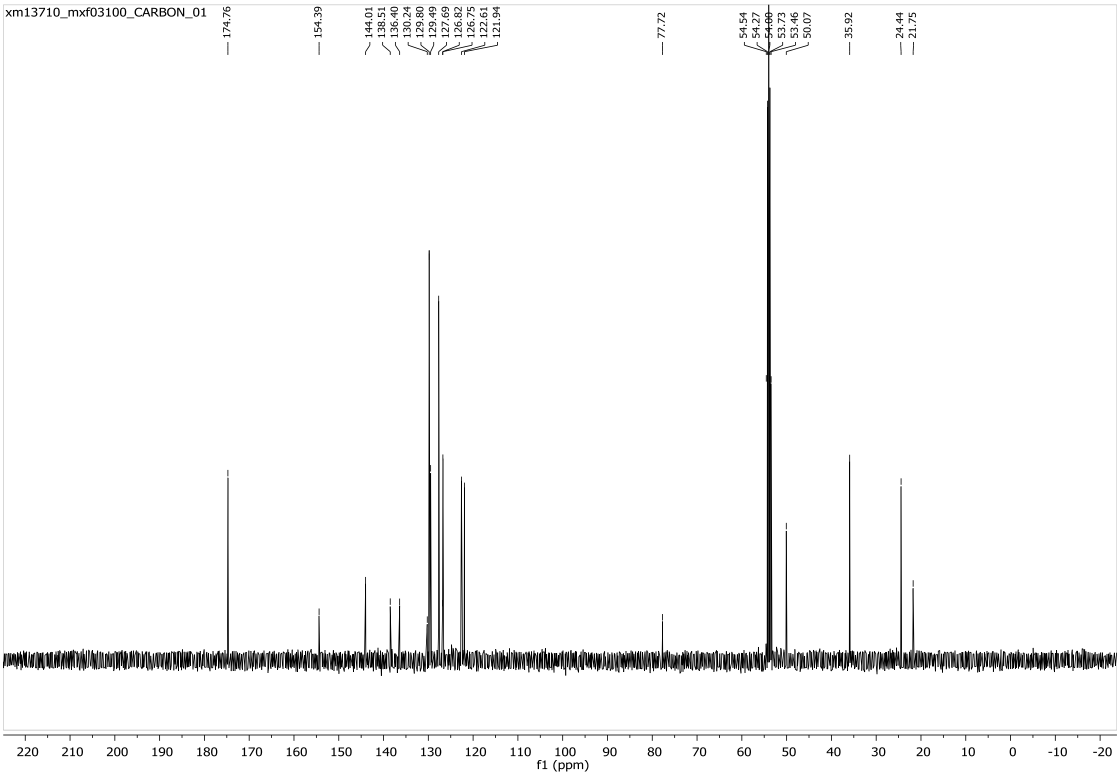

1'-((4-Methoxyphenyl)sulfonyl)spiro[indole-3,2'-pyrrolidine] (4b)

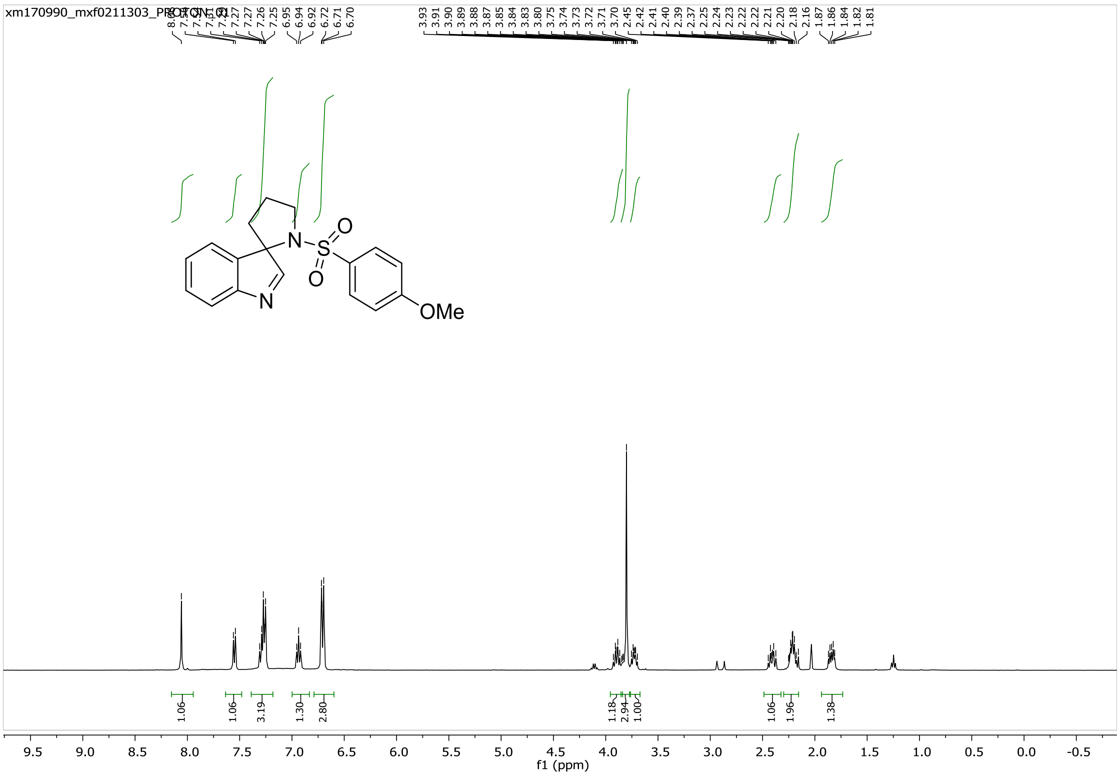

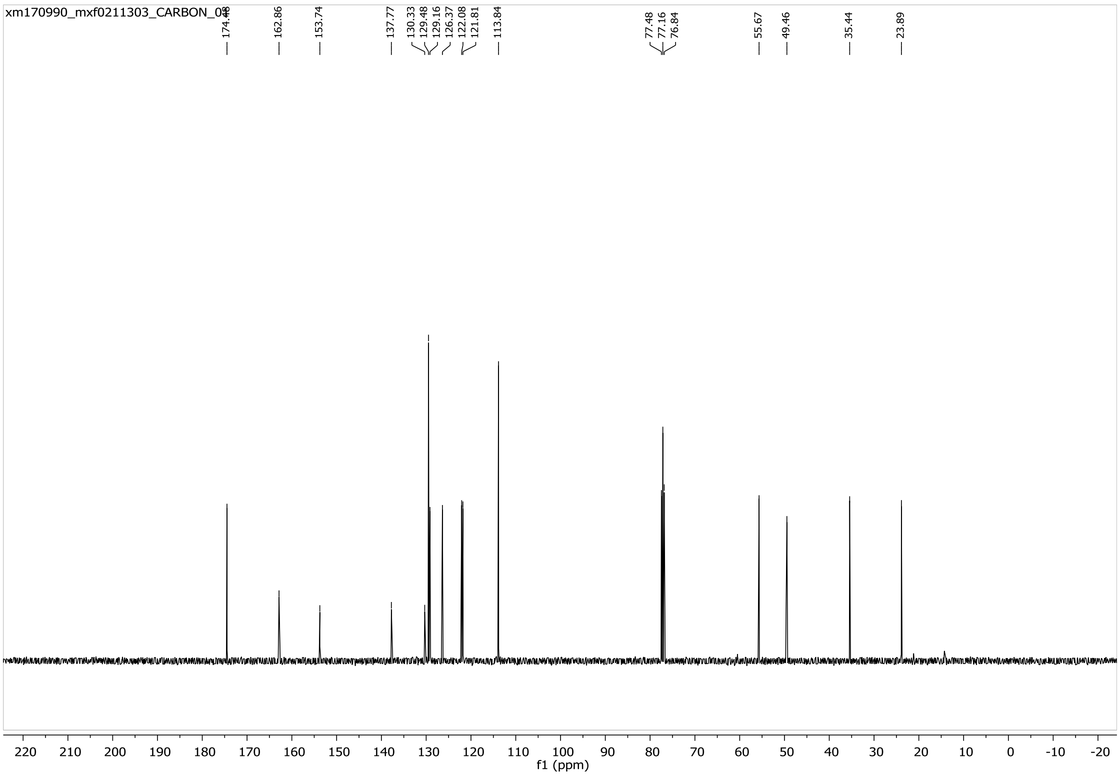

Benzyl spiro[indole-3,2'-pyrrolidine]-1'-carboxylate (4c)

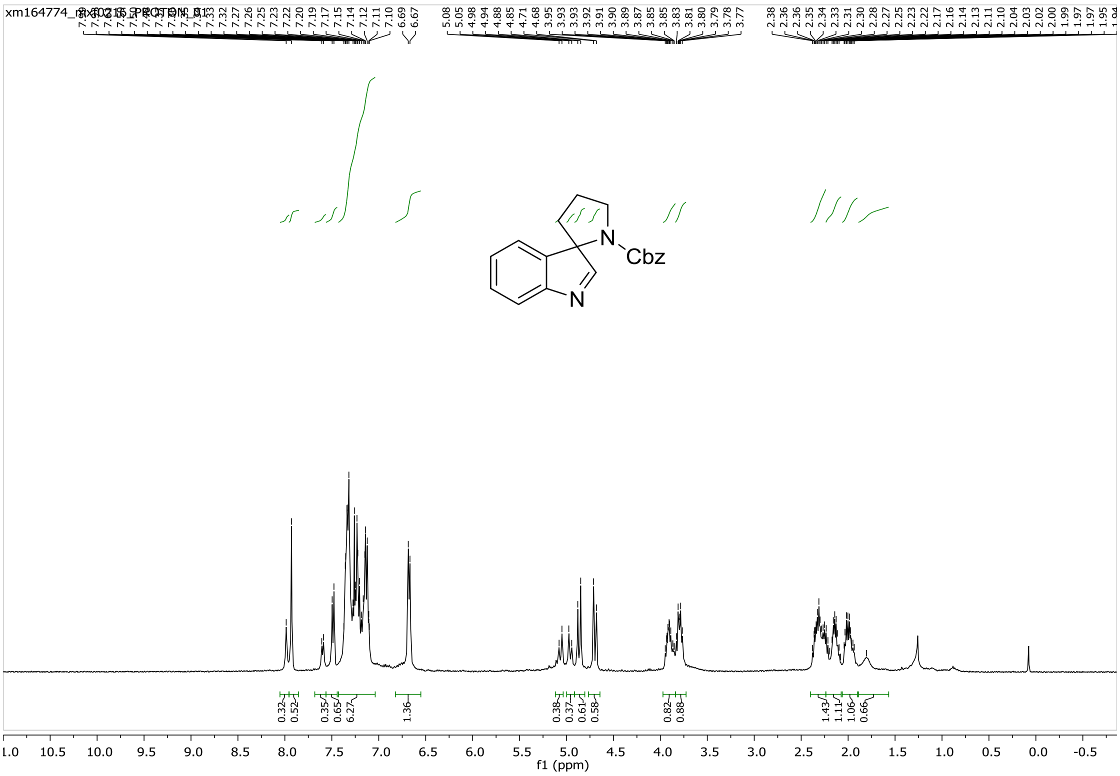

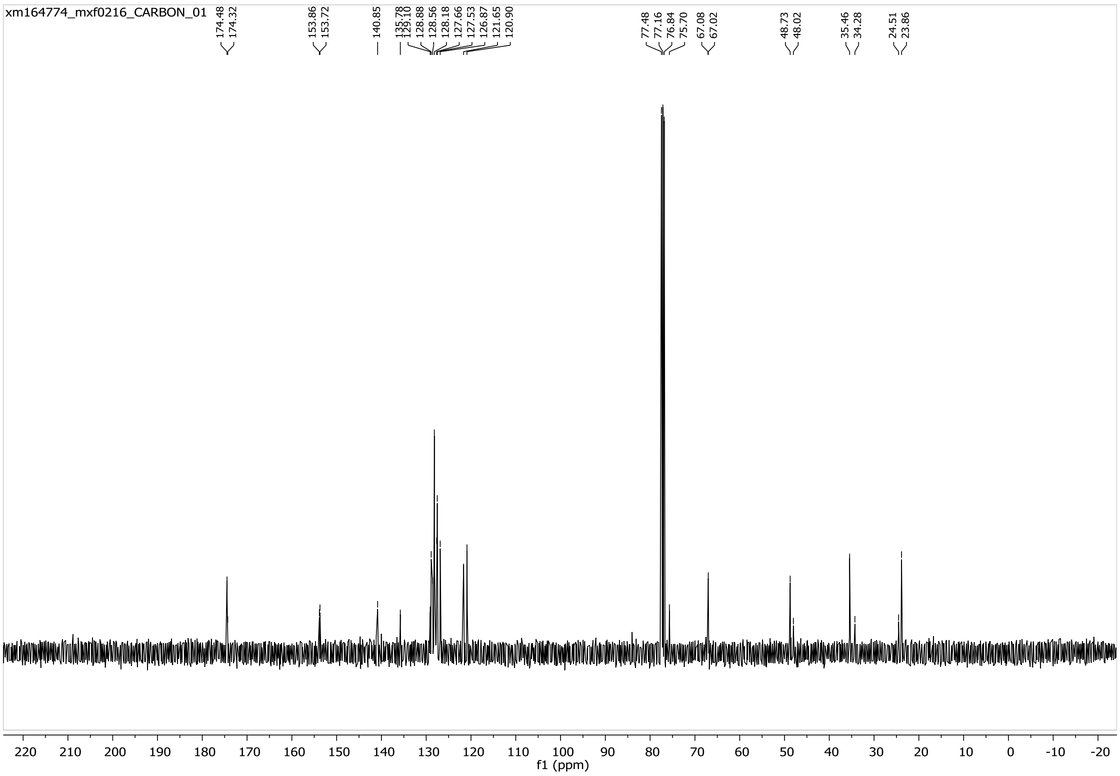

Methyl spiro[indole-3,2'-pyrrolidine]-1'-carboxylate (4d)

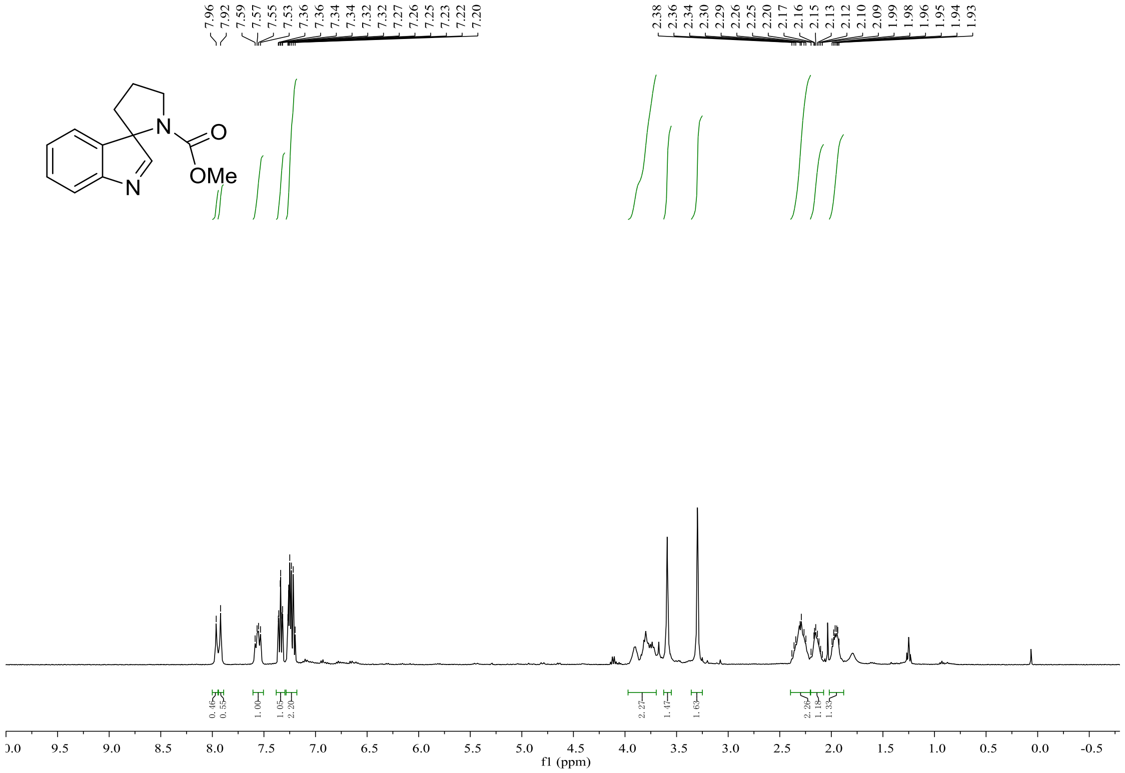

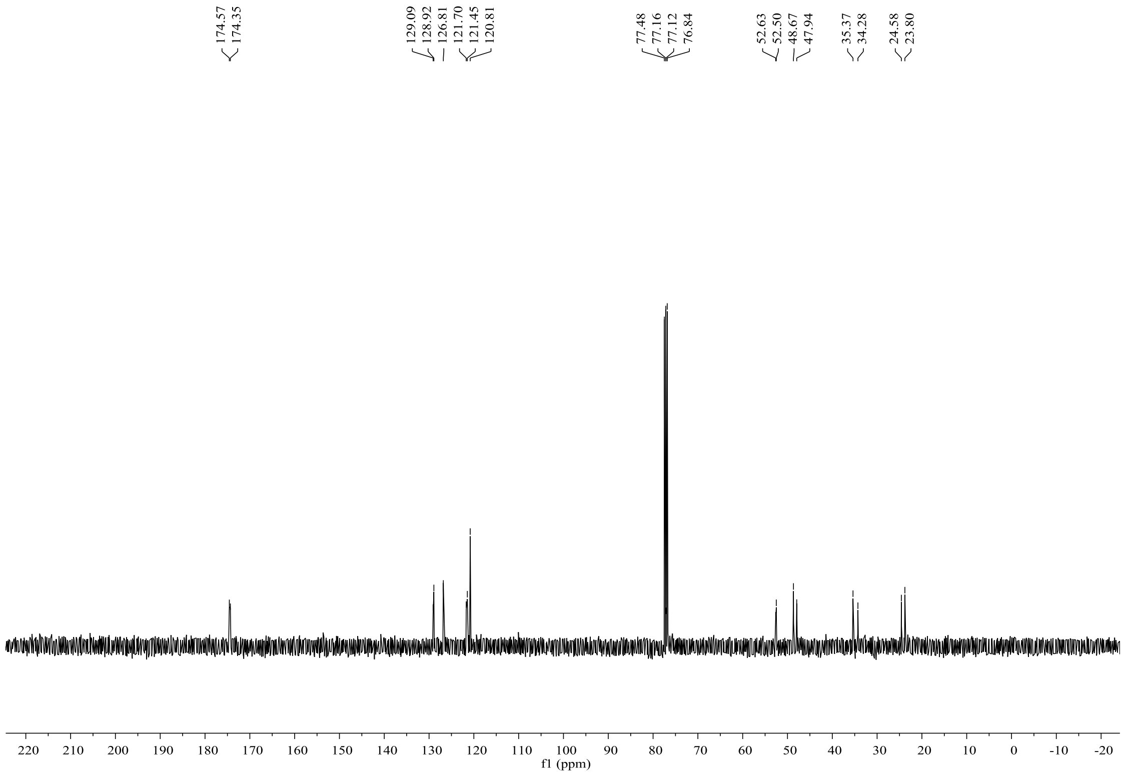

***tert*-Butyl spiro[indole-3,2'-pyrrolidine]-1'-carboxylate (4e)**

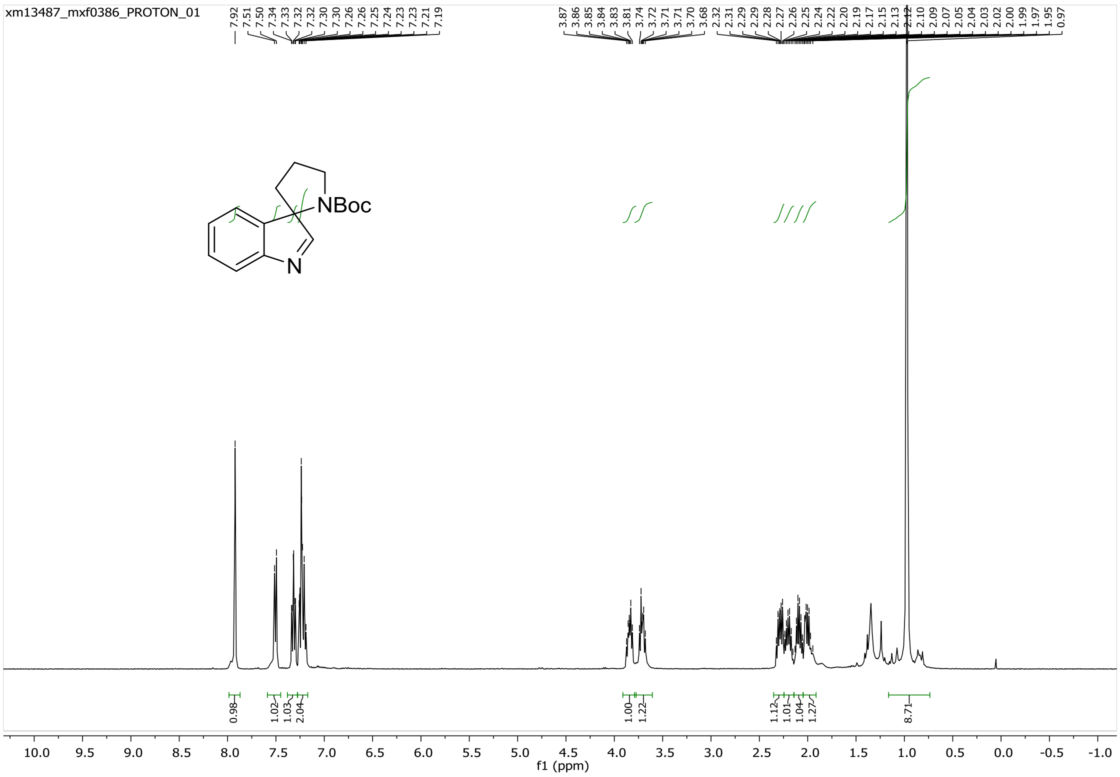

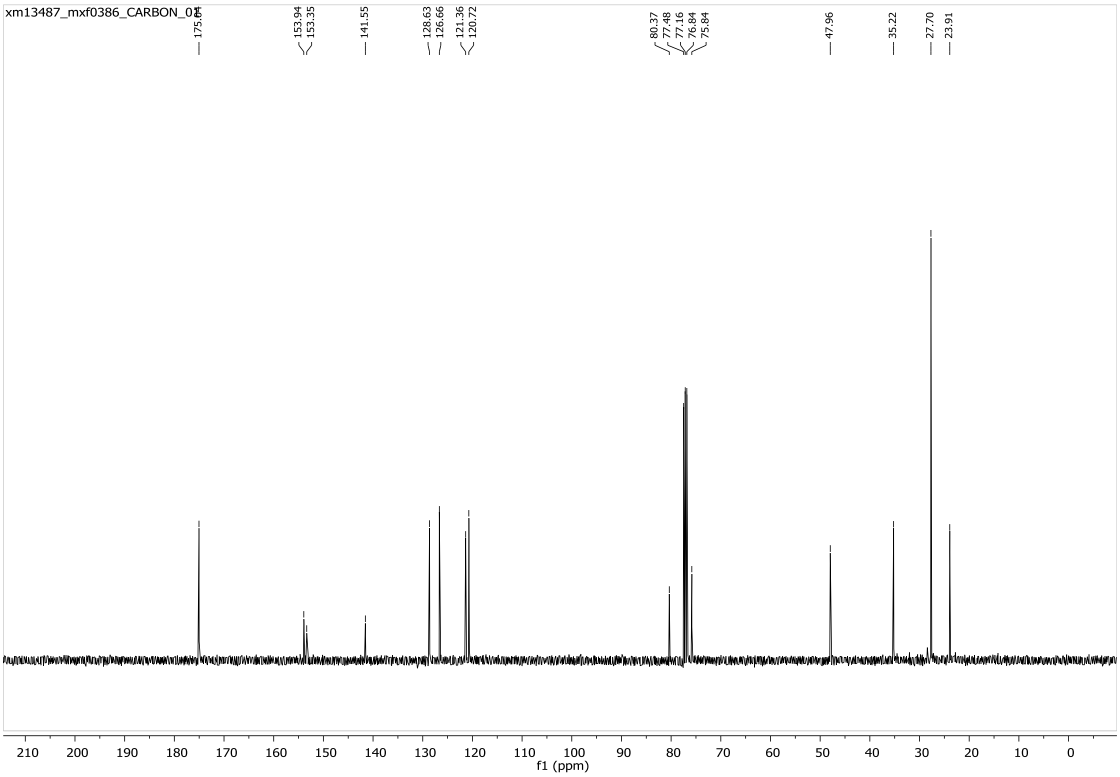

Allyl spiro[indole-3,2'-pyrrolidine]-1'-carboxylate (4f)

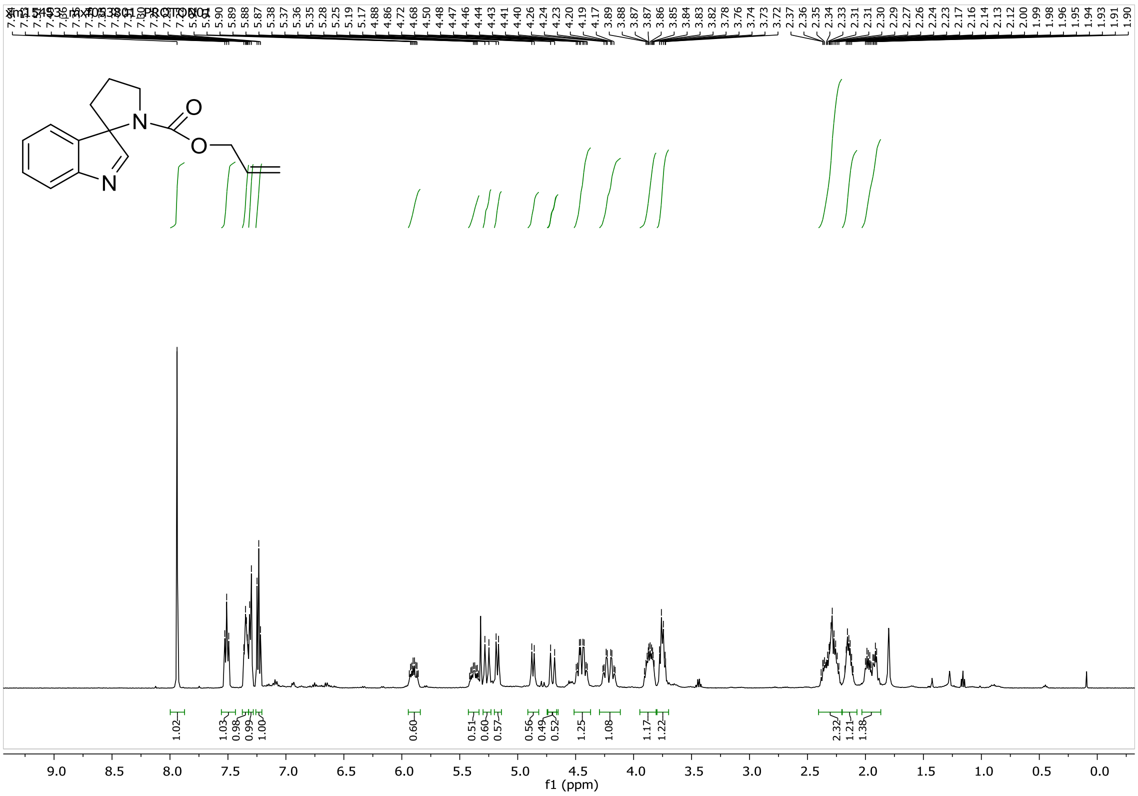

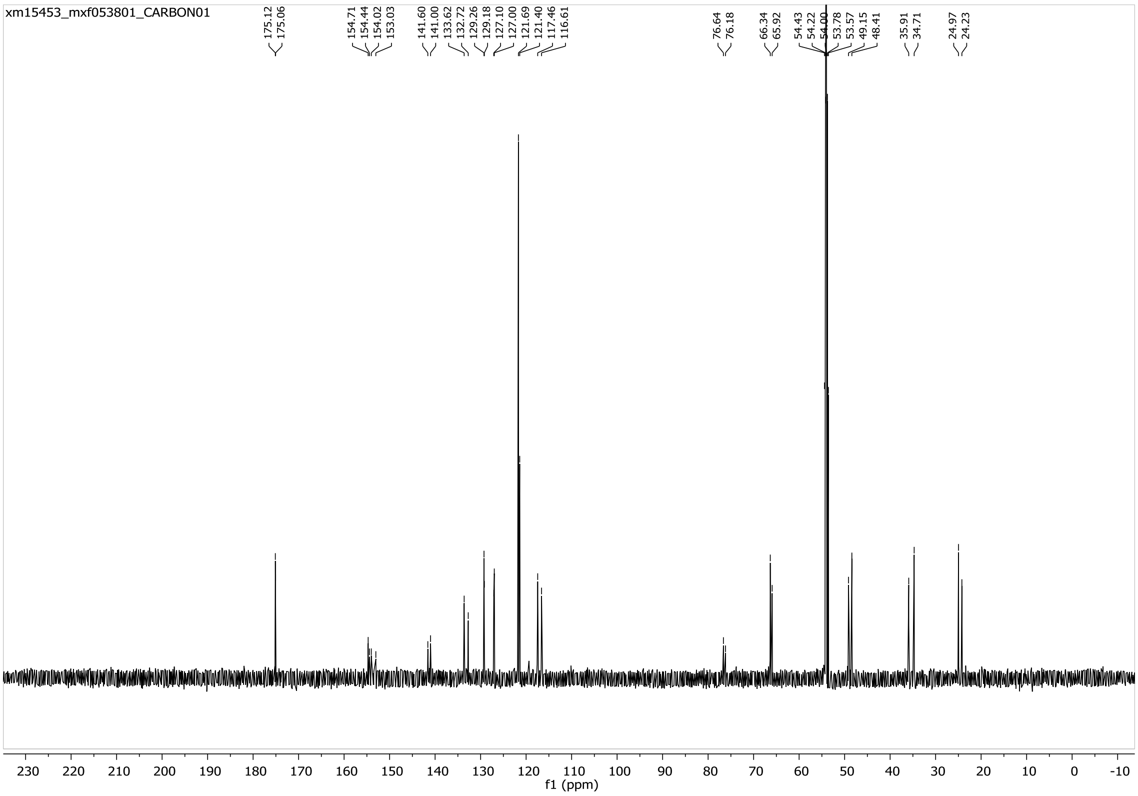

Benzyl 5-methoxyspiro[indole-3,2'-pyrrolidine]-1'-carboxylate (4g)

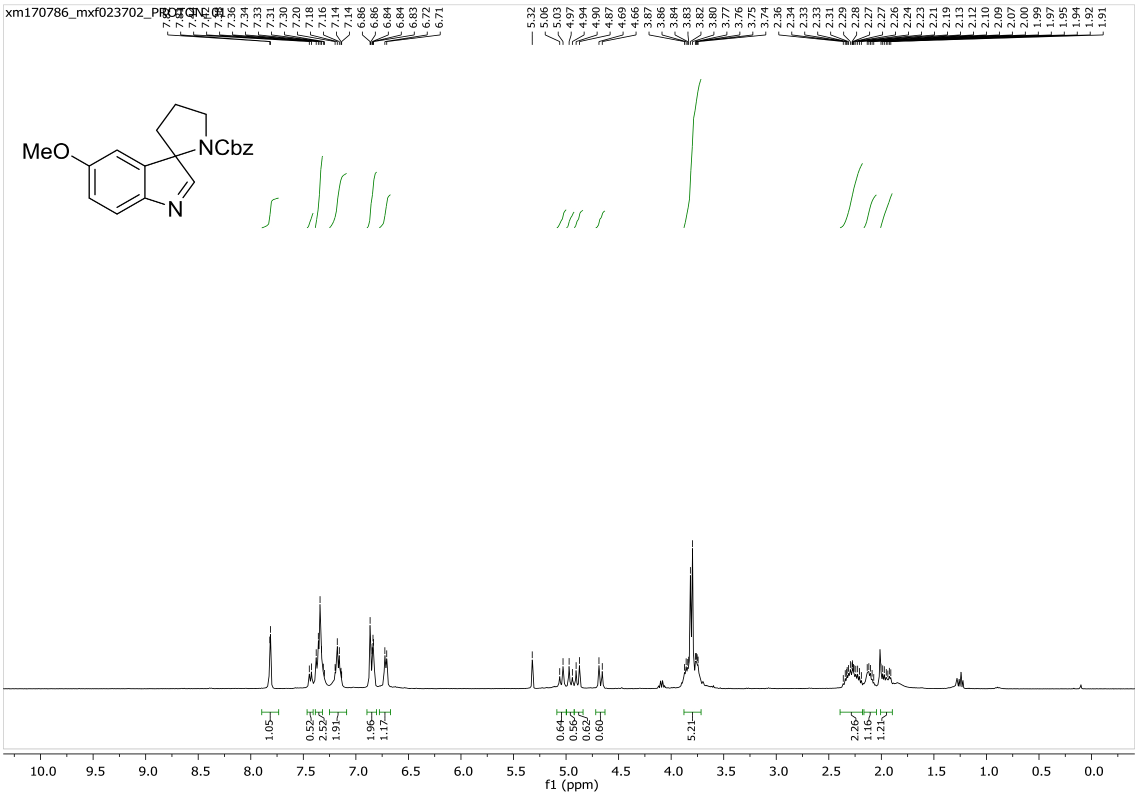

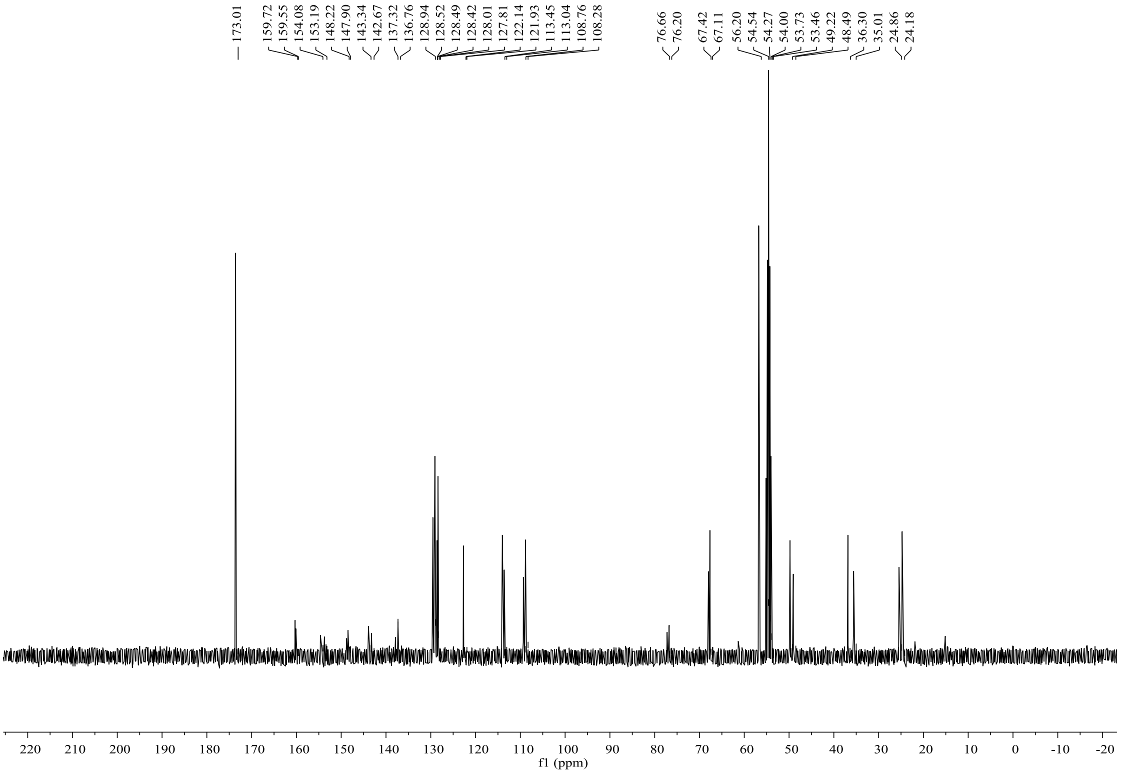

Benzyl 5-methylspiro[indole-3,2'-pyrrolidine]-1'-carboxylate (4h)

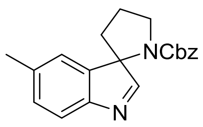

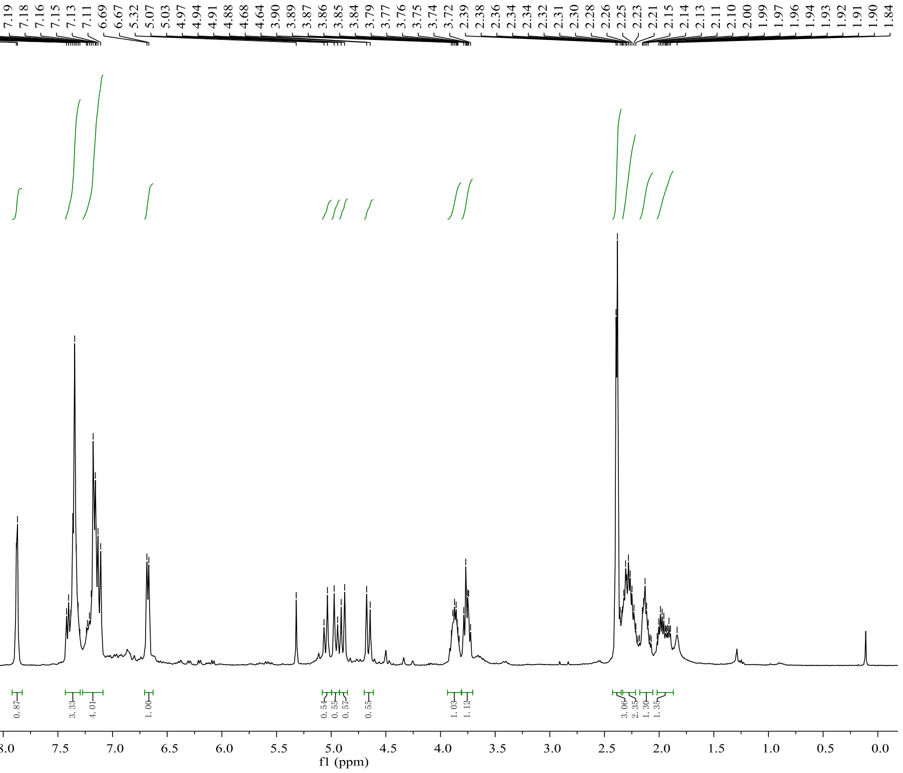

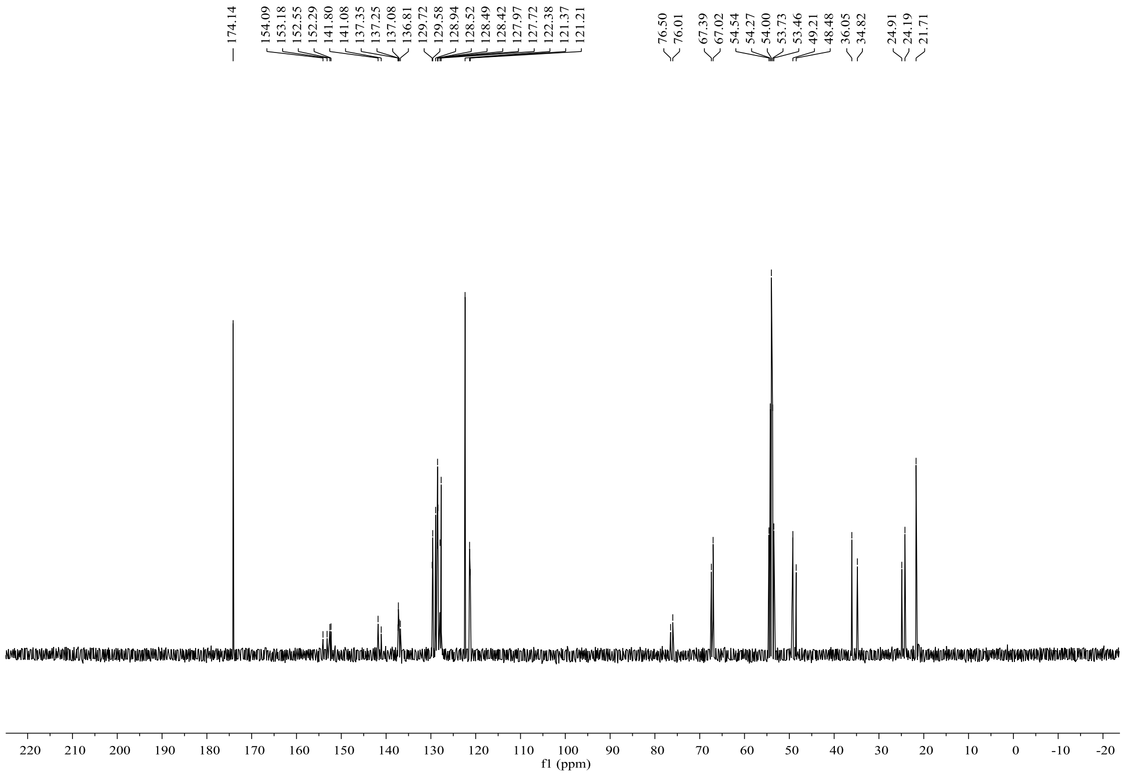

Benzyl 5-fluorospiro[indole-3,2'-pyrrolidine]-1'-carboxylate (4i)

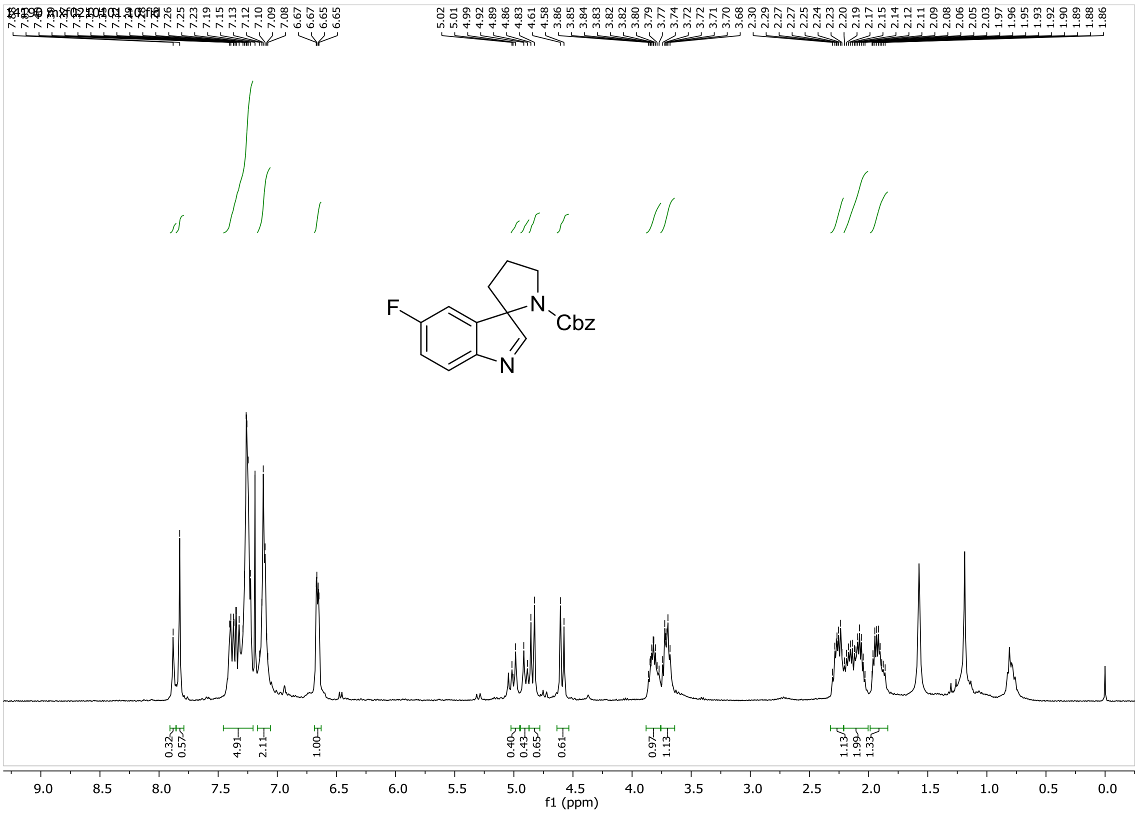

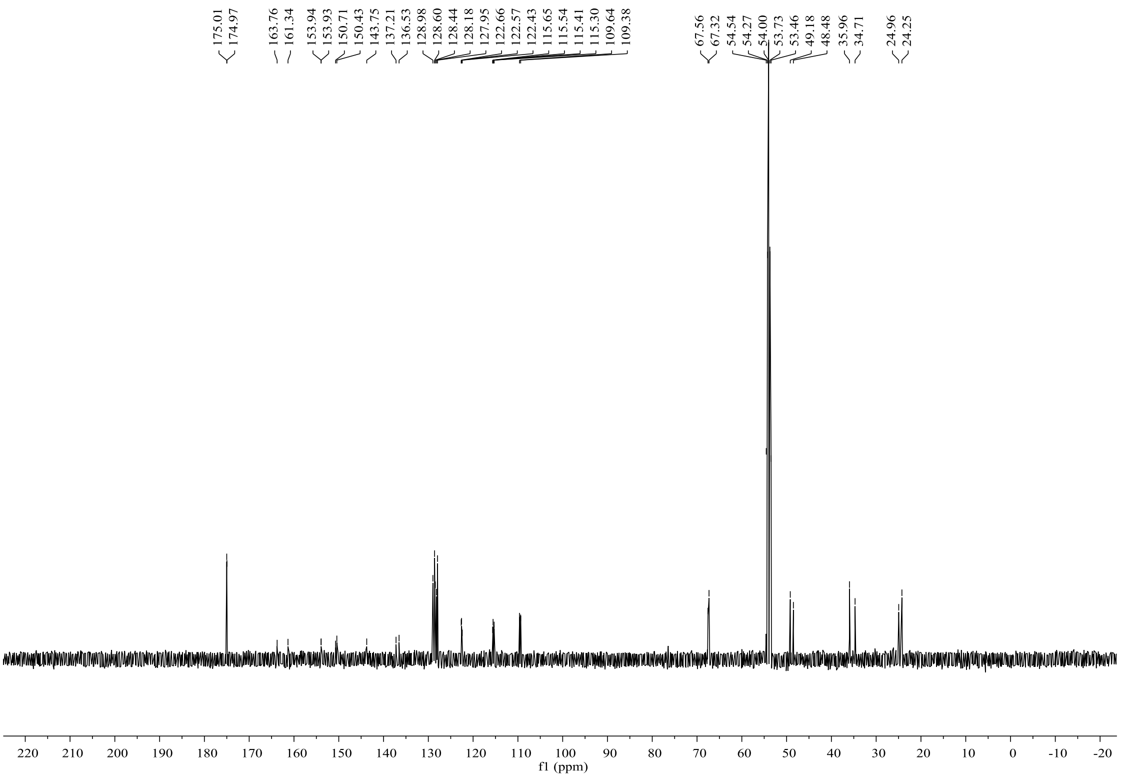

tert-Butyl 5-fluorospiro[indole-3,2'-pyrrolidine]-1'-carboxylate (4j)

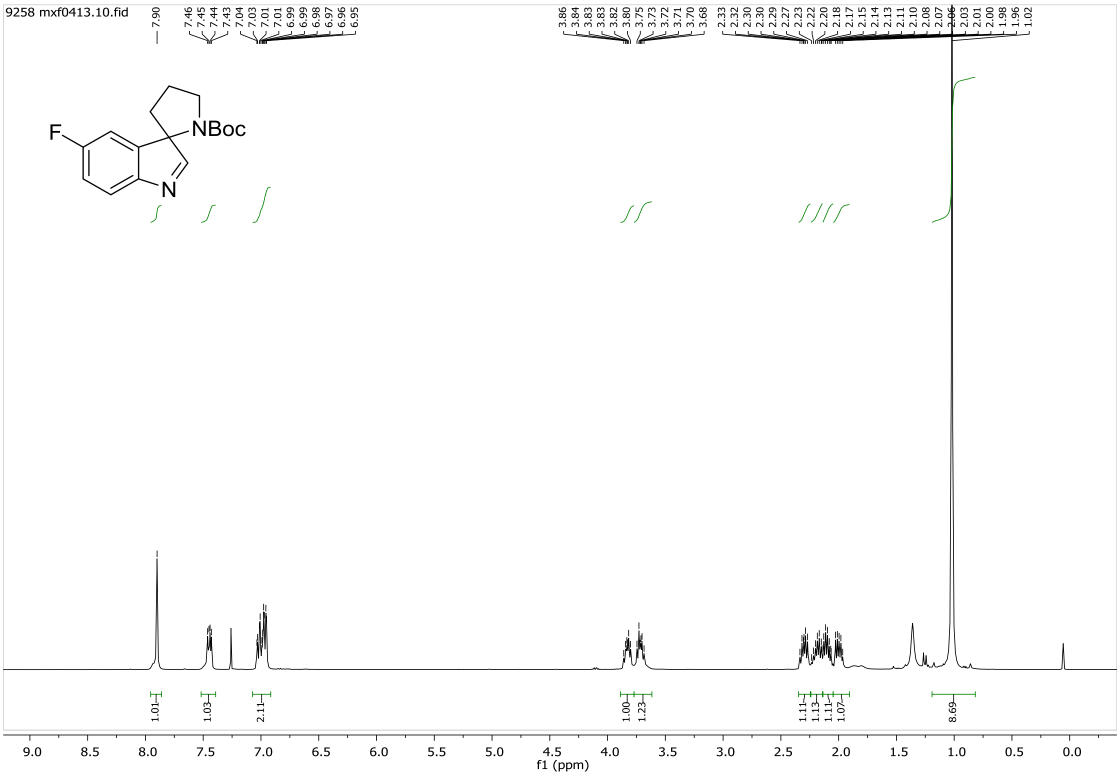

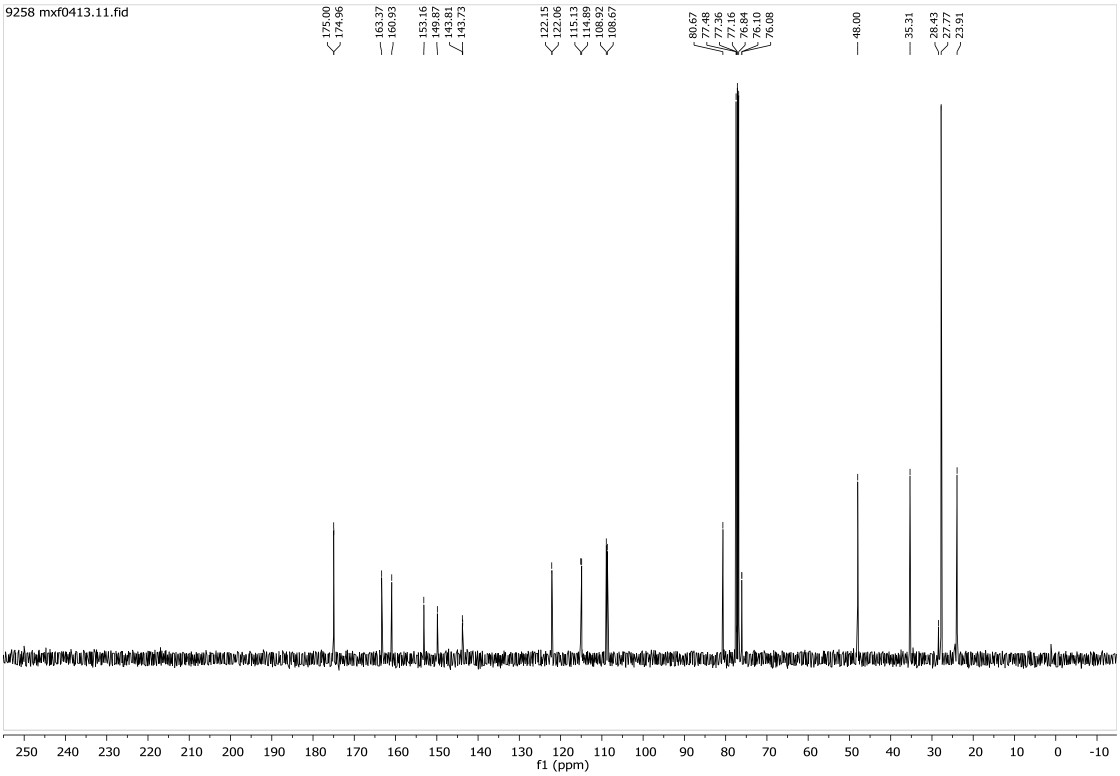

Benzyl 5-chlorospiro[indole-3,2'-pyrrolidine]-1'-carboxylate (4k)

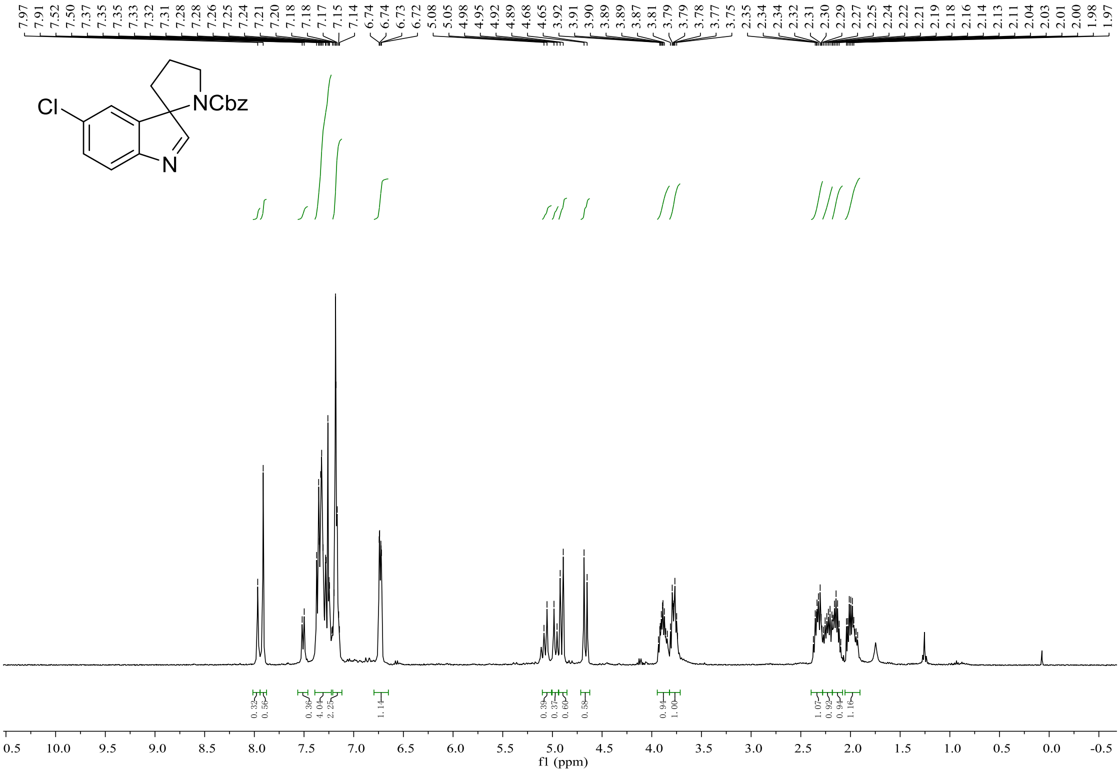

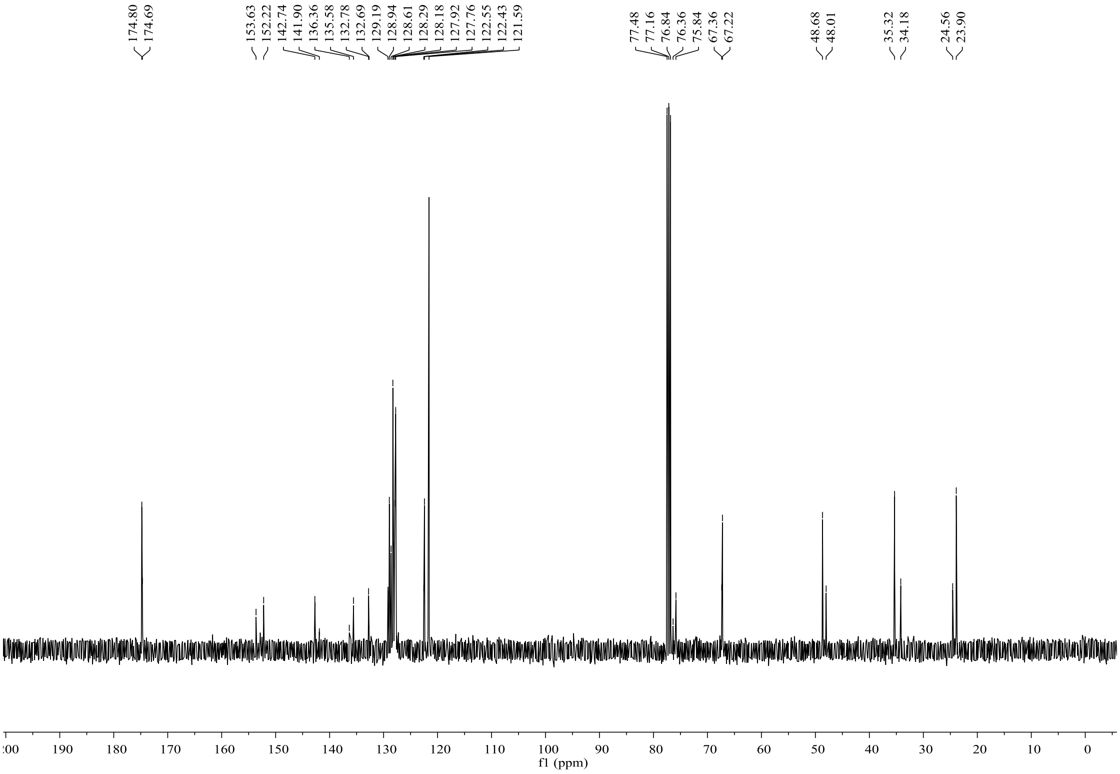

Benzyl 5-bromospiro[indole-3,2'-pyrrolidine]-1'-carboxylate (4l)

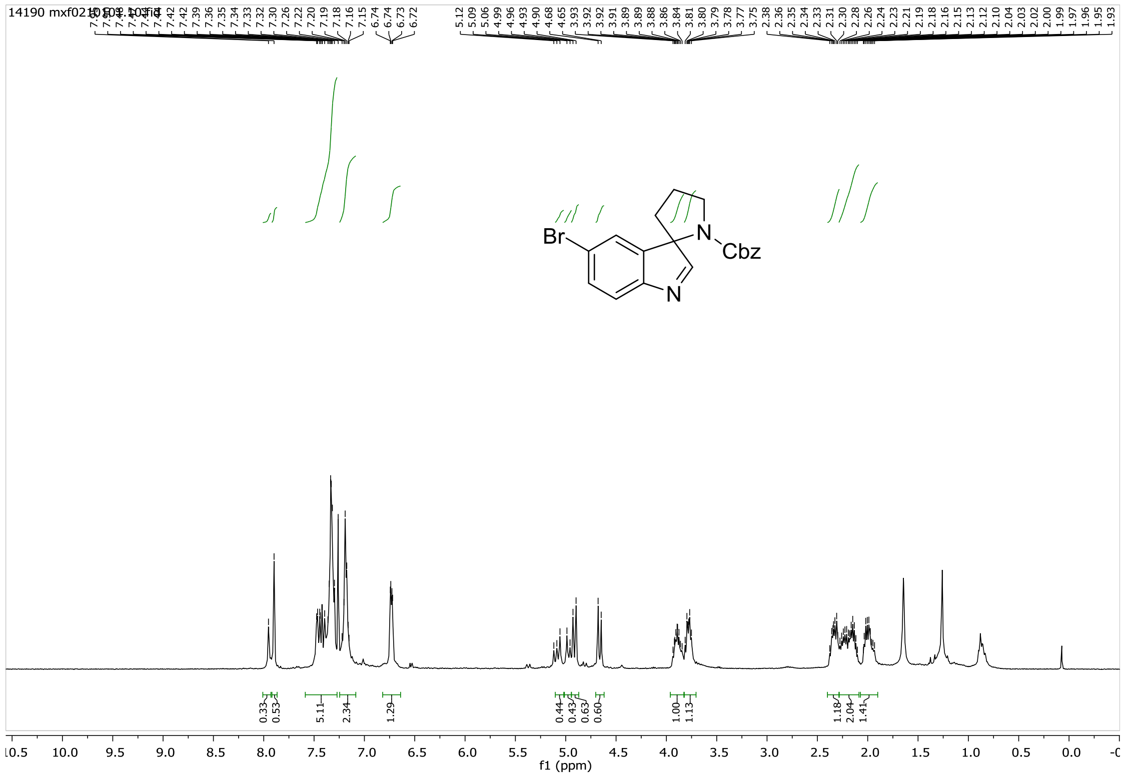

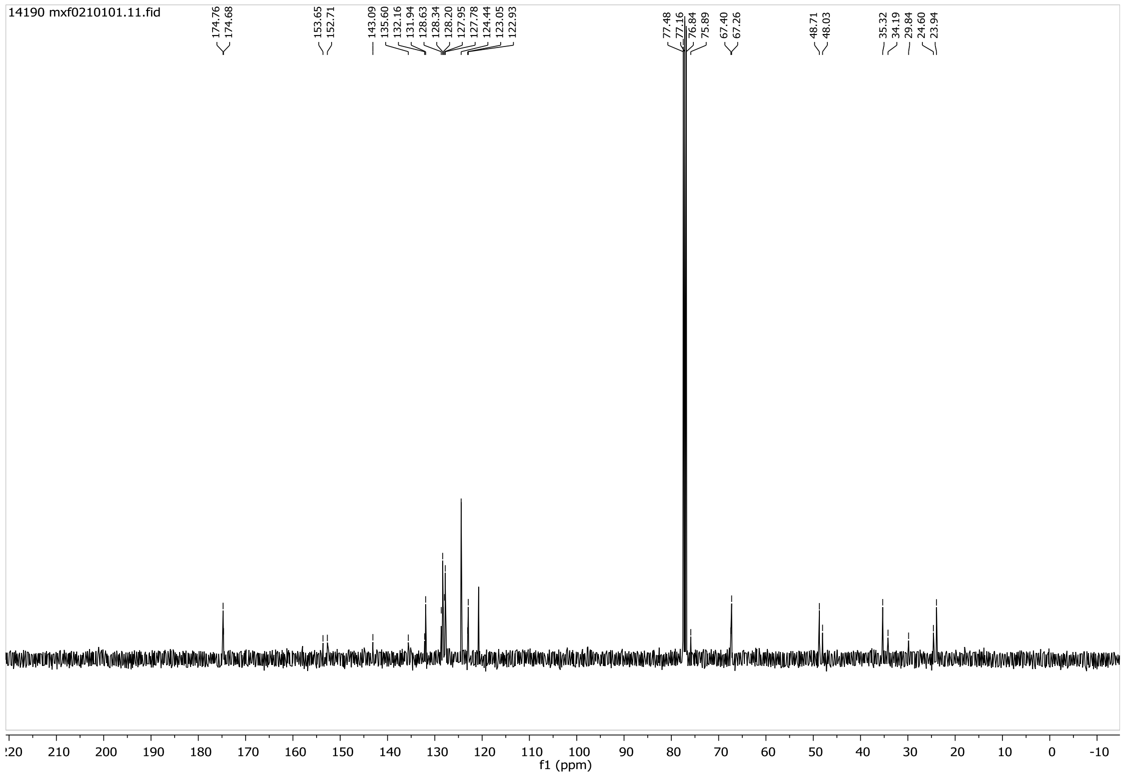

tert-Butyl 5-iodospiro[indole-3,2'-pyrrolidine]-1'-carboxylate (4m)

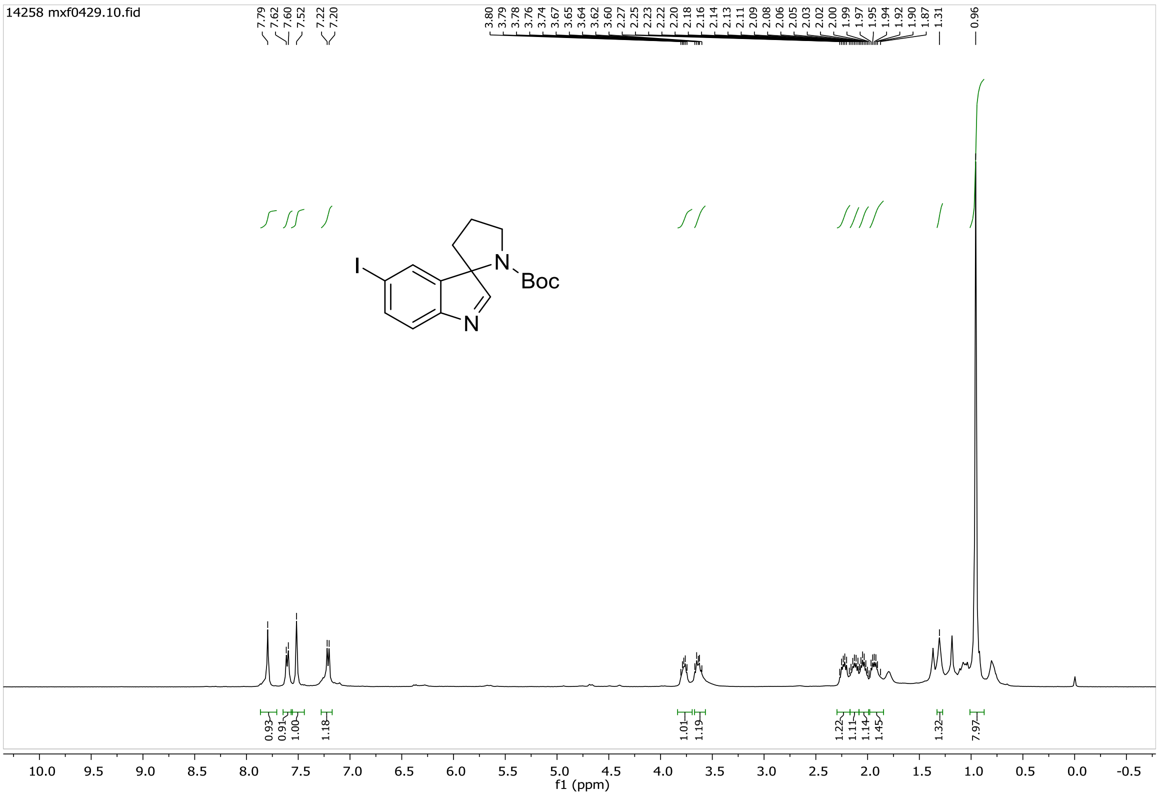

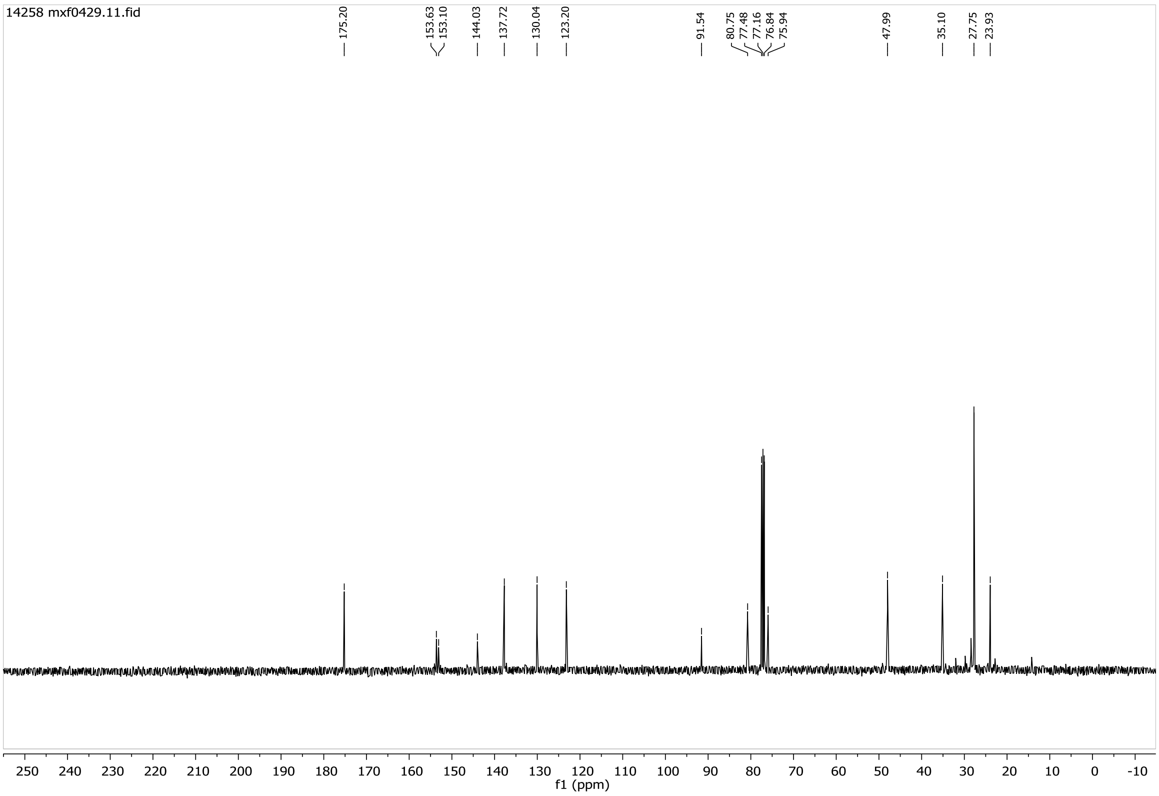

Benzyl 2-methylspiro[indole-3,2'-pyrrolidine]-1'-carboxylate (4n)

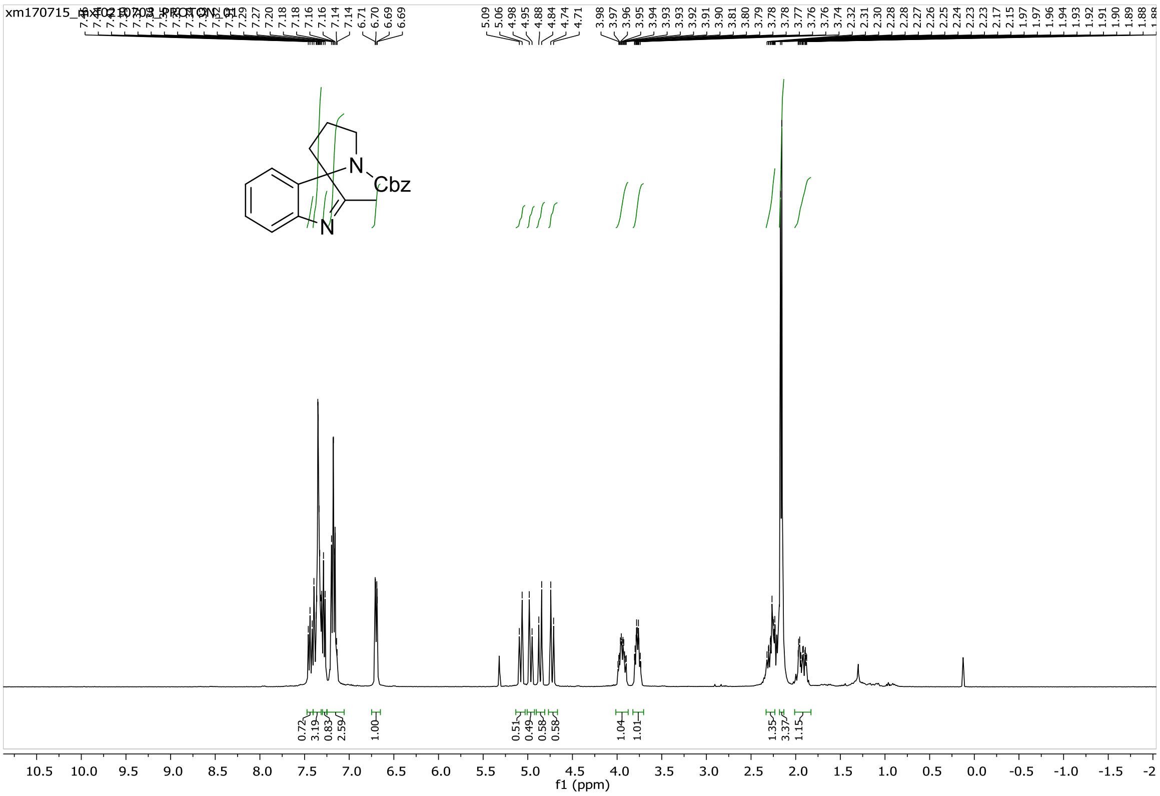

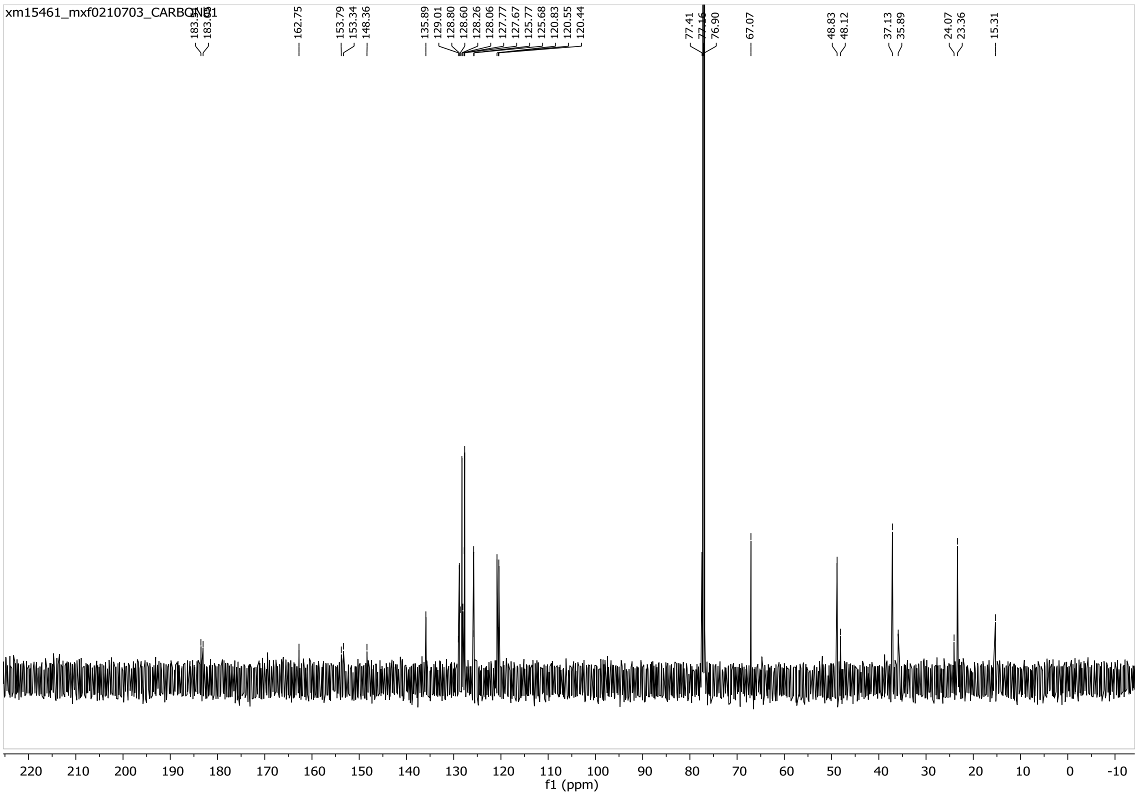

Benzyl 5,7-dimethylspiro[indole-3,2'-pyrrolidine]-1'-carboxylate (4o)

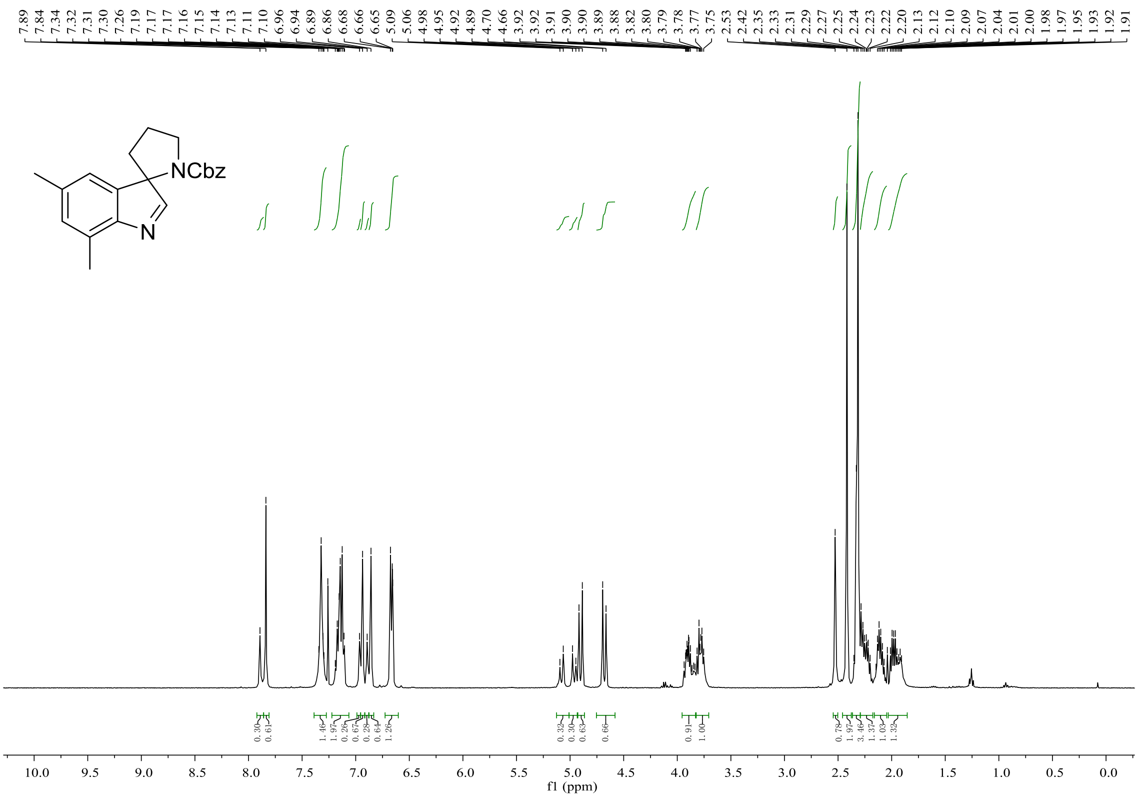

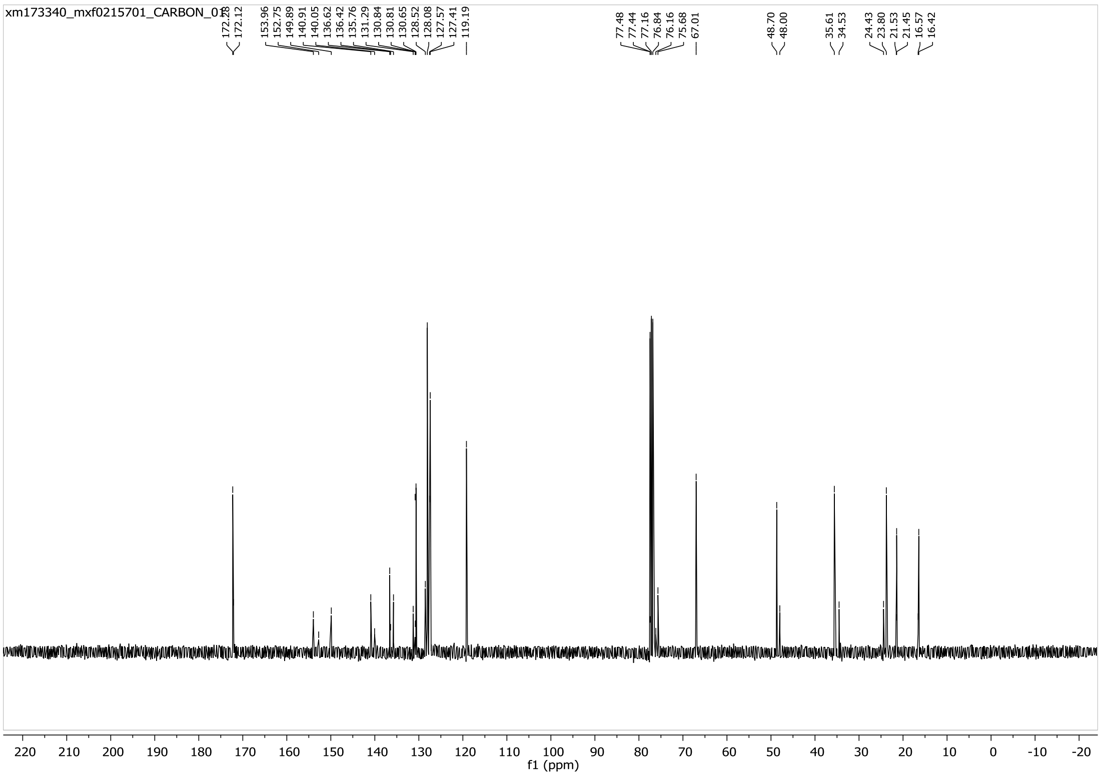

Benzyl 7-methylspiro[indole-3,2'-pyrrolidine]-1'-carboxylate (4p)

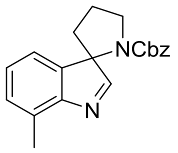

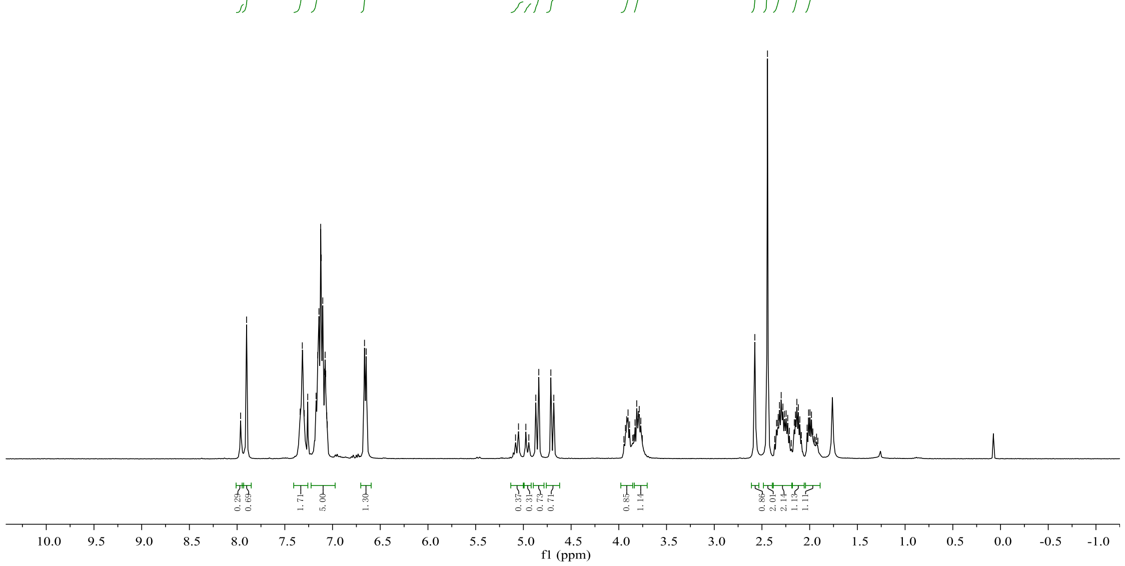

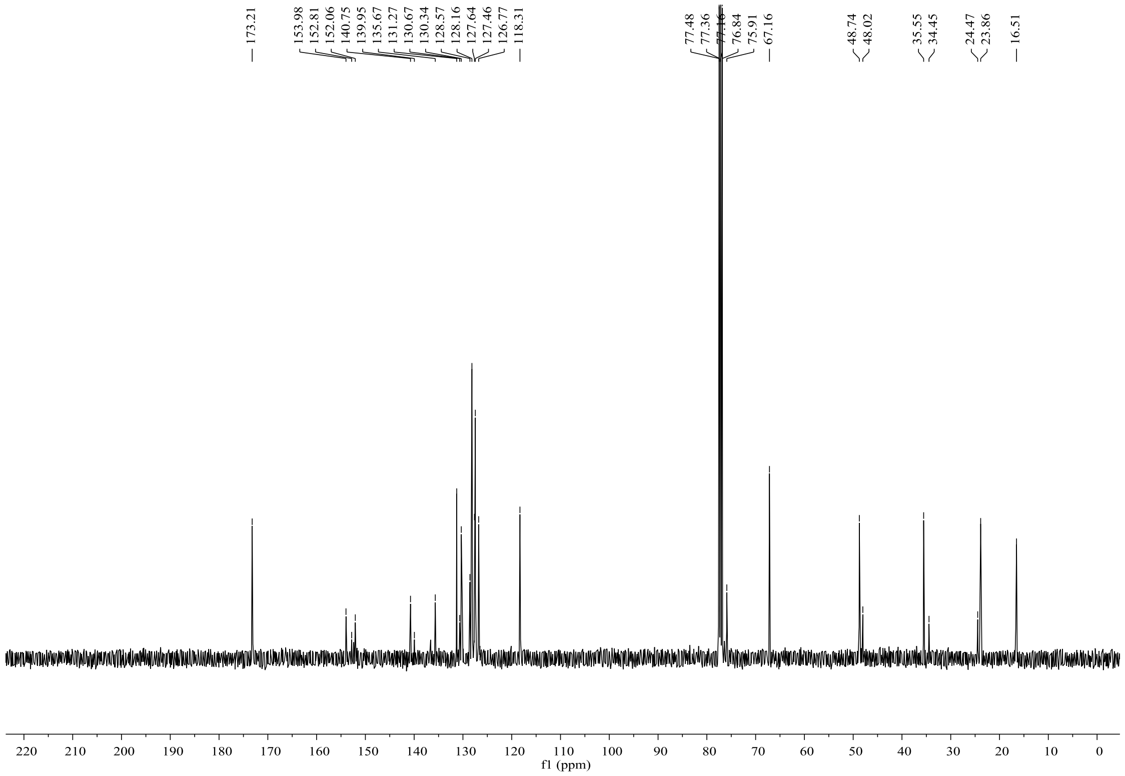

tert-Butyl 4'-methylspiro[indole-3,2'-pyrrolidine]-1'-carboxylate (4q)

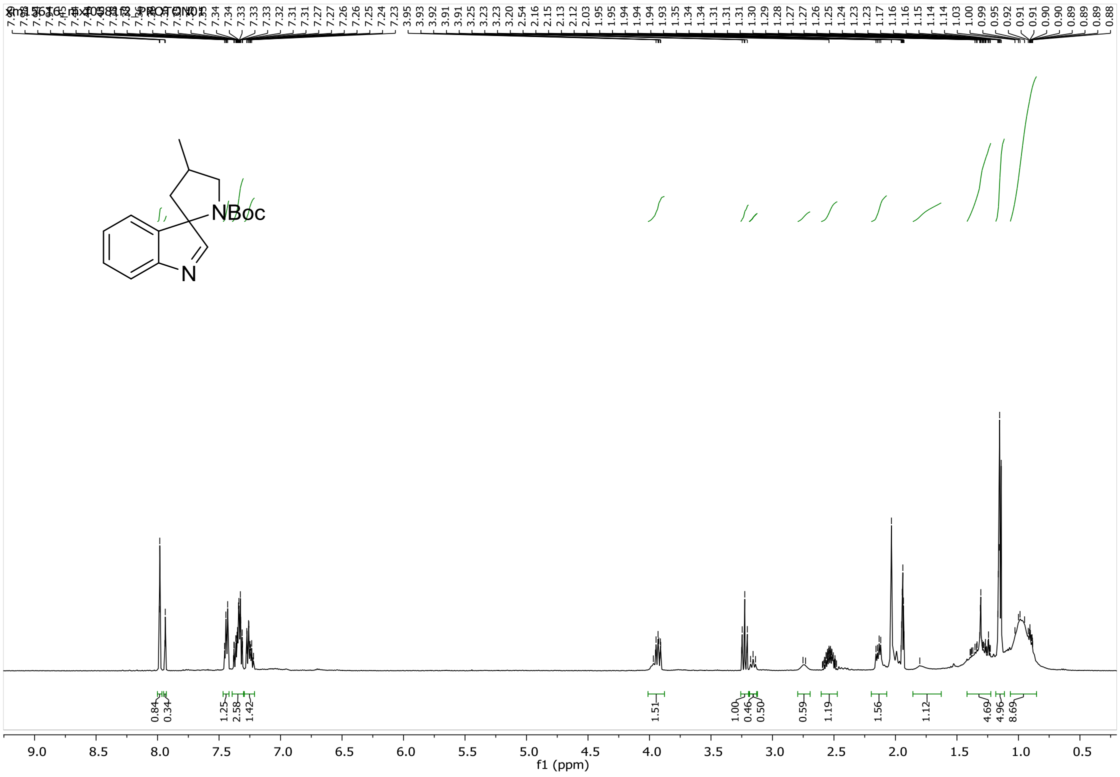

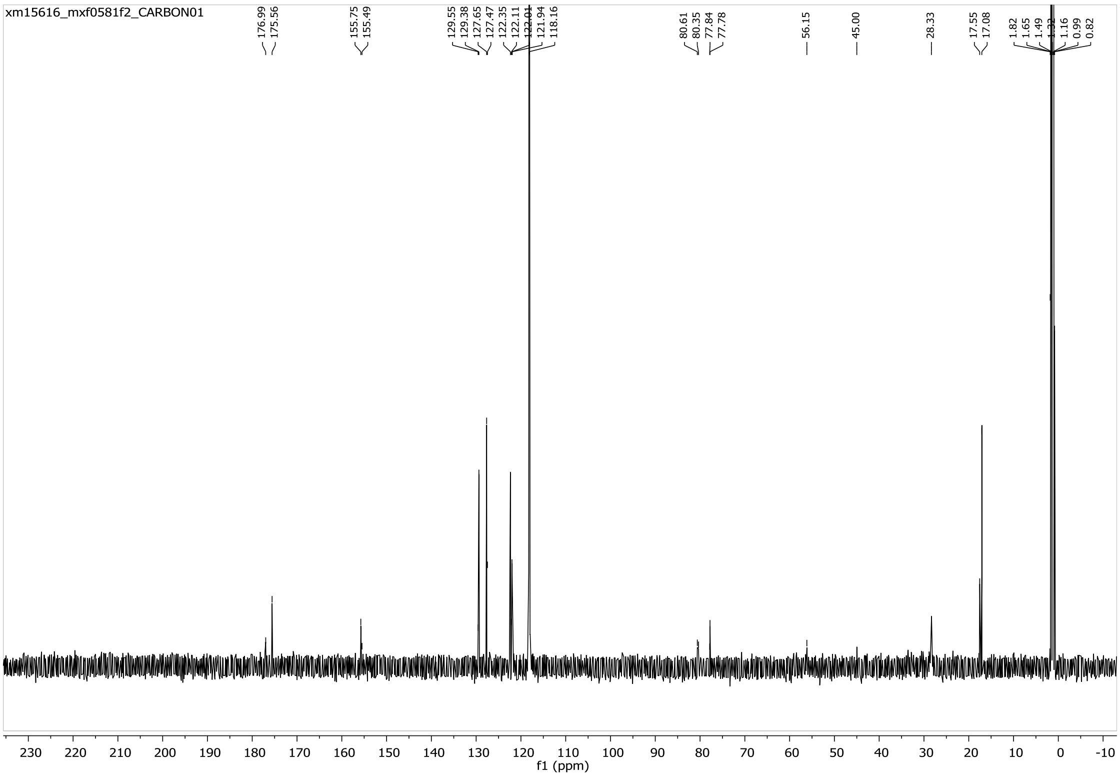

3-(*1H*-Indol-3-yl)-2-methylpropyl 4-methylbenzenesulfonate

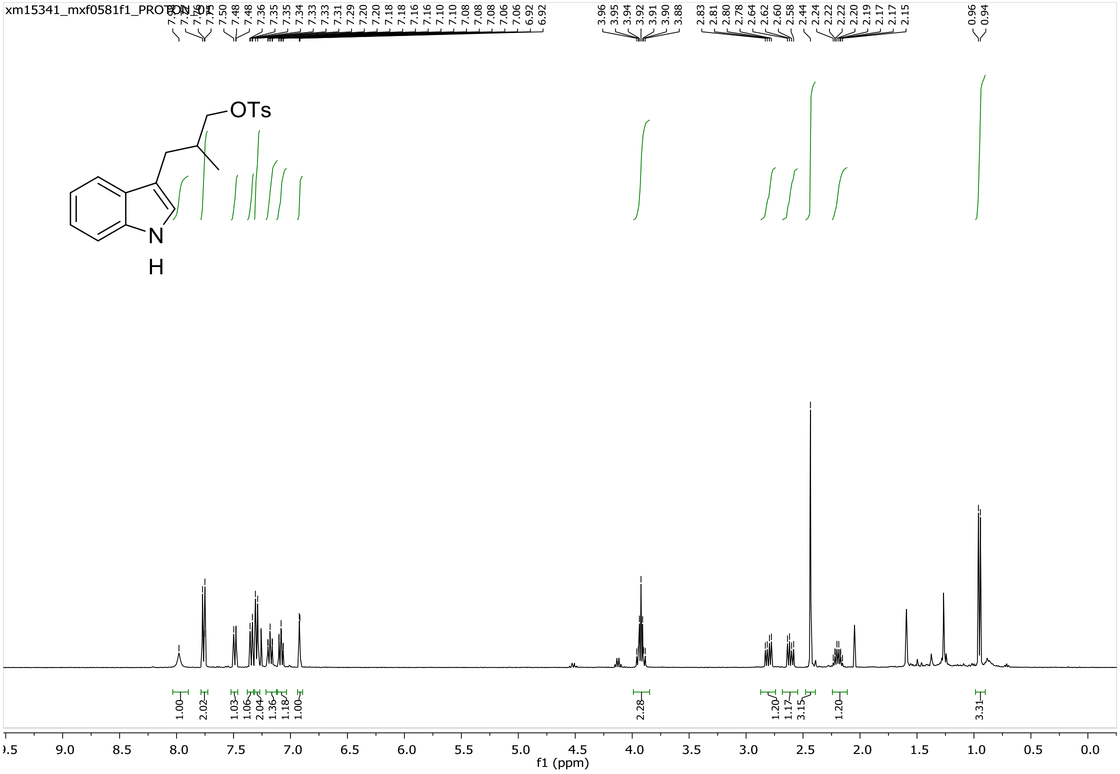

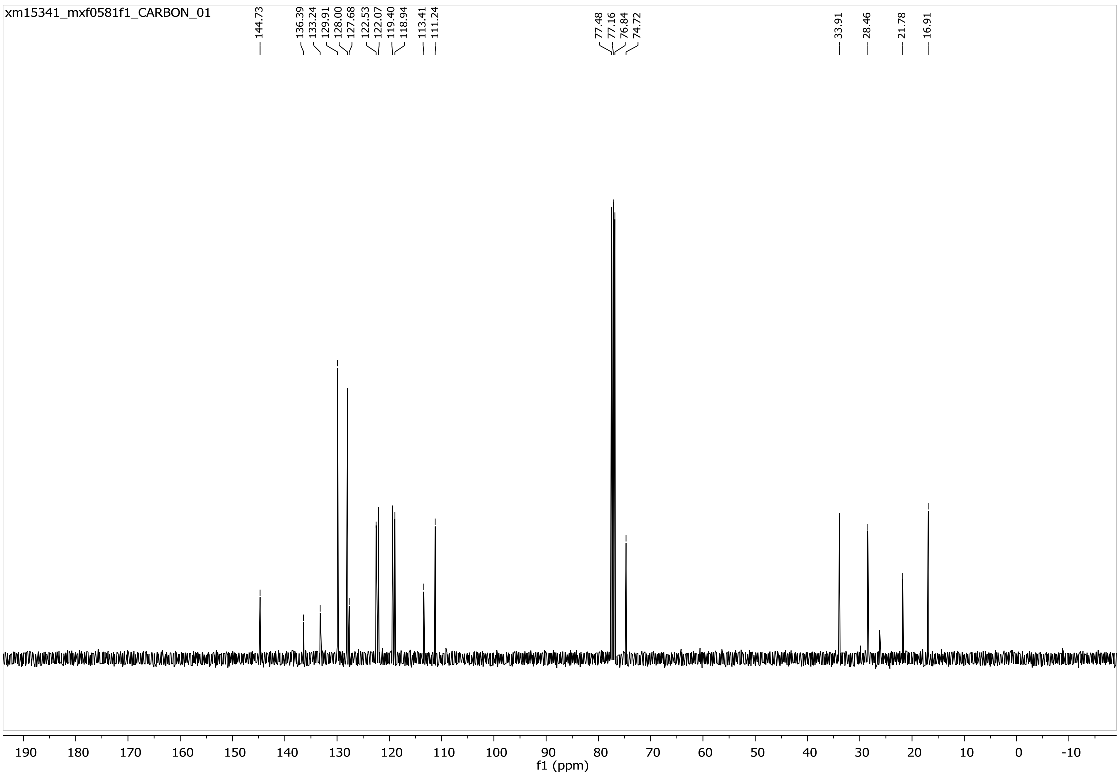

tert-Butyl (3_S*_,5'_R*_)-5'-methylspiro[indole-3,2'-pyrrolidine]-1'-carboxylate (4r)

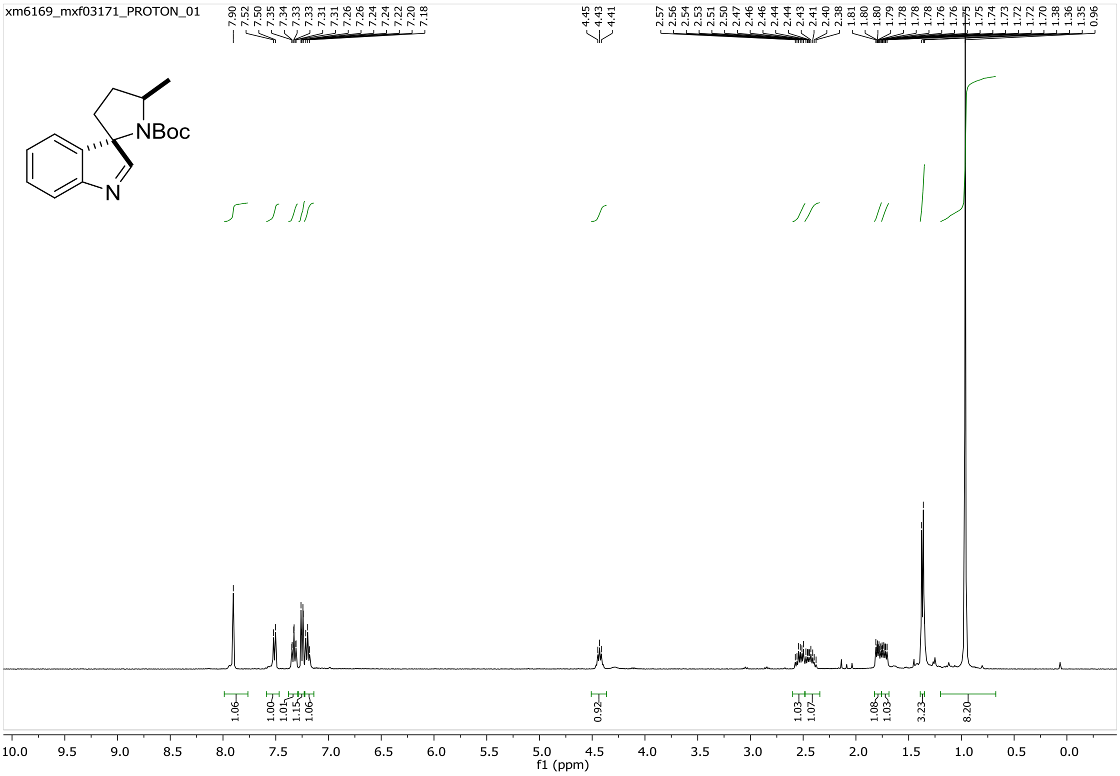

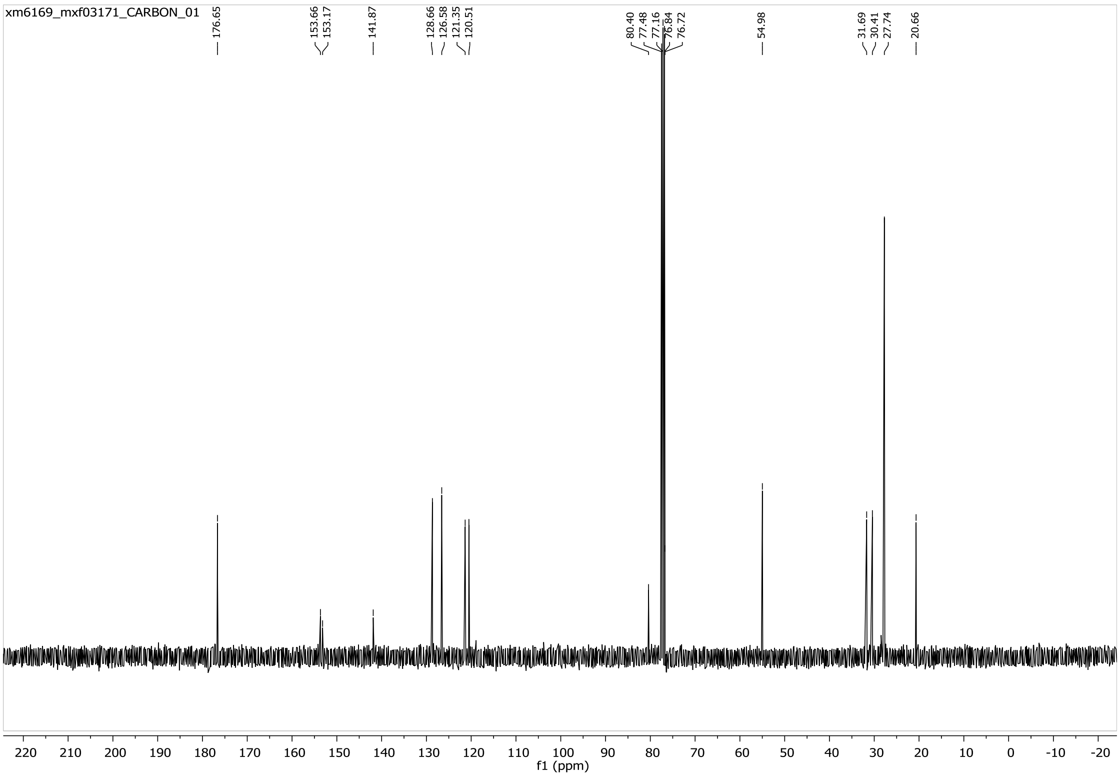

***tert*-Butyl (3*S*,3'*S*)-3'-methylspiro[indole-3,2'-pyrrolidine]-1'-carboxylate (4s)**

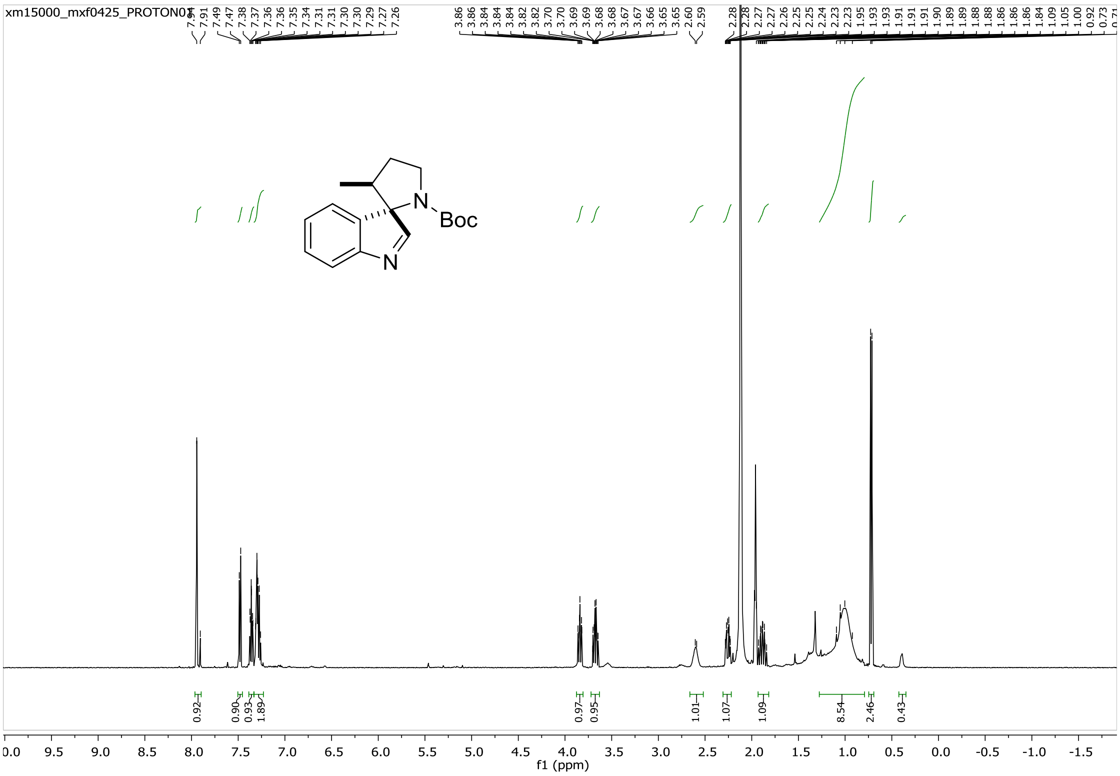

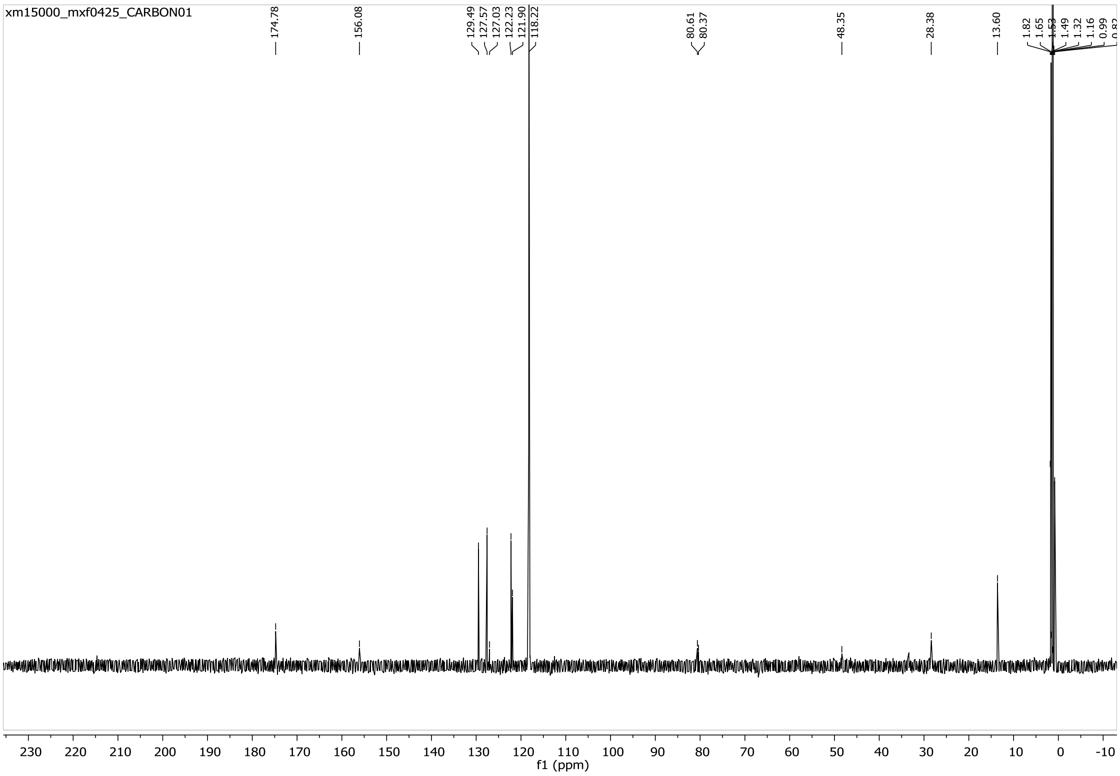

tert-Butyl (3_S_*,3'_S_*)-3'-isopropylspiro[indole-3,2'-pyrrolidine]-1'-carboxylate (4t)

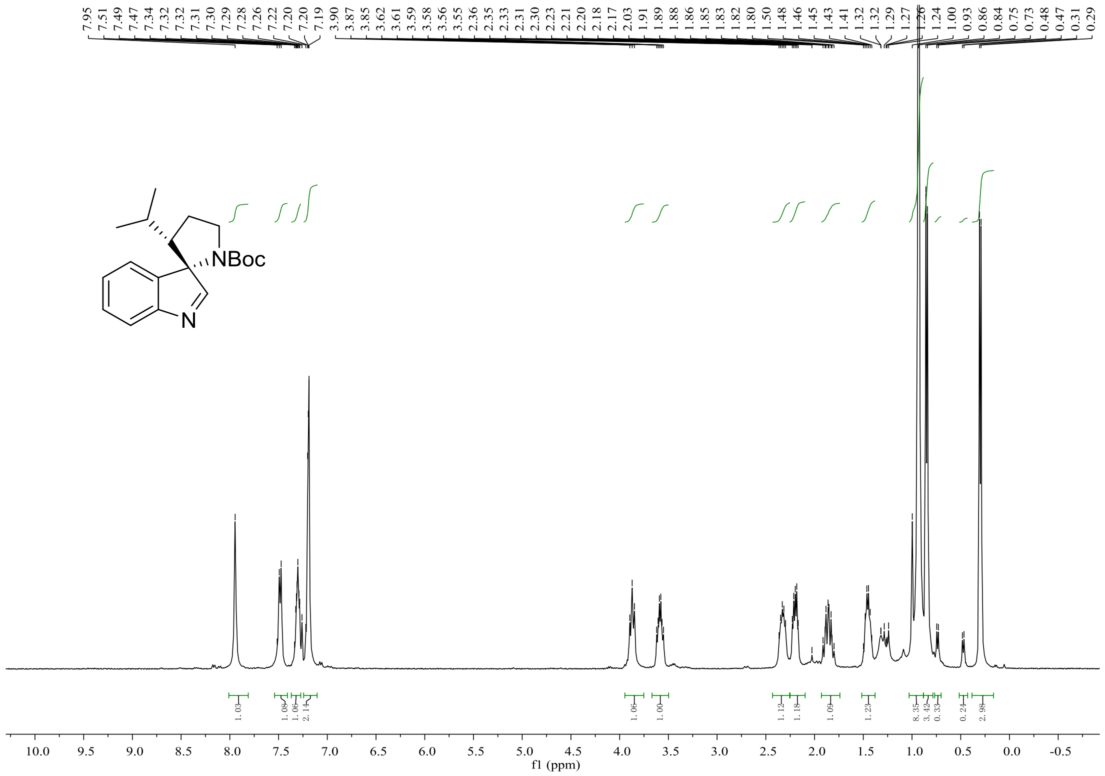

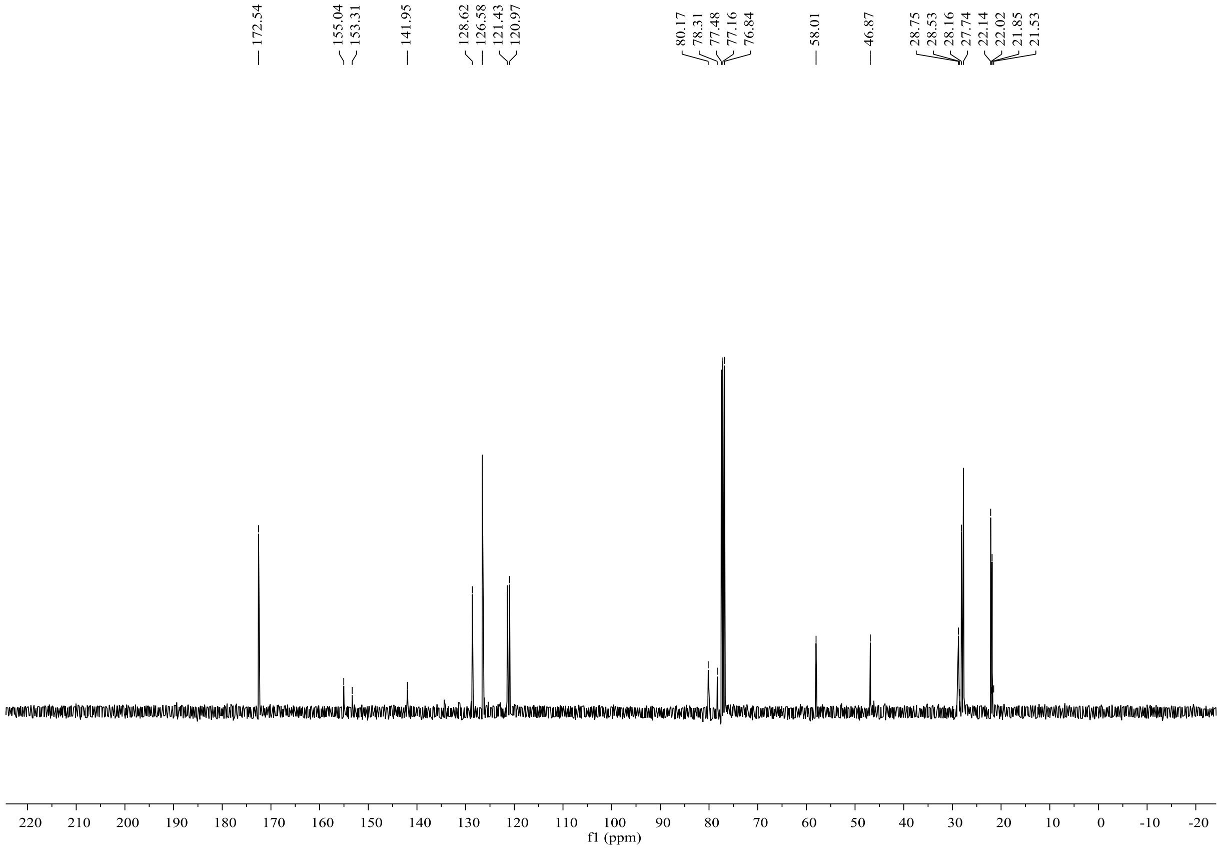

tert-Butyl (3S*,3'R*)-3'-phenylspiro[indole-3,2'-pyrrolidine]-1'-carboxylate (4u)

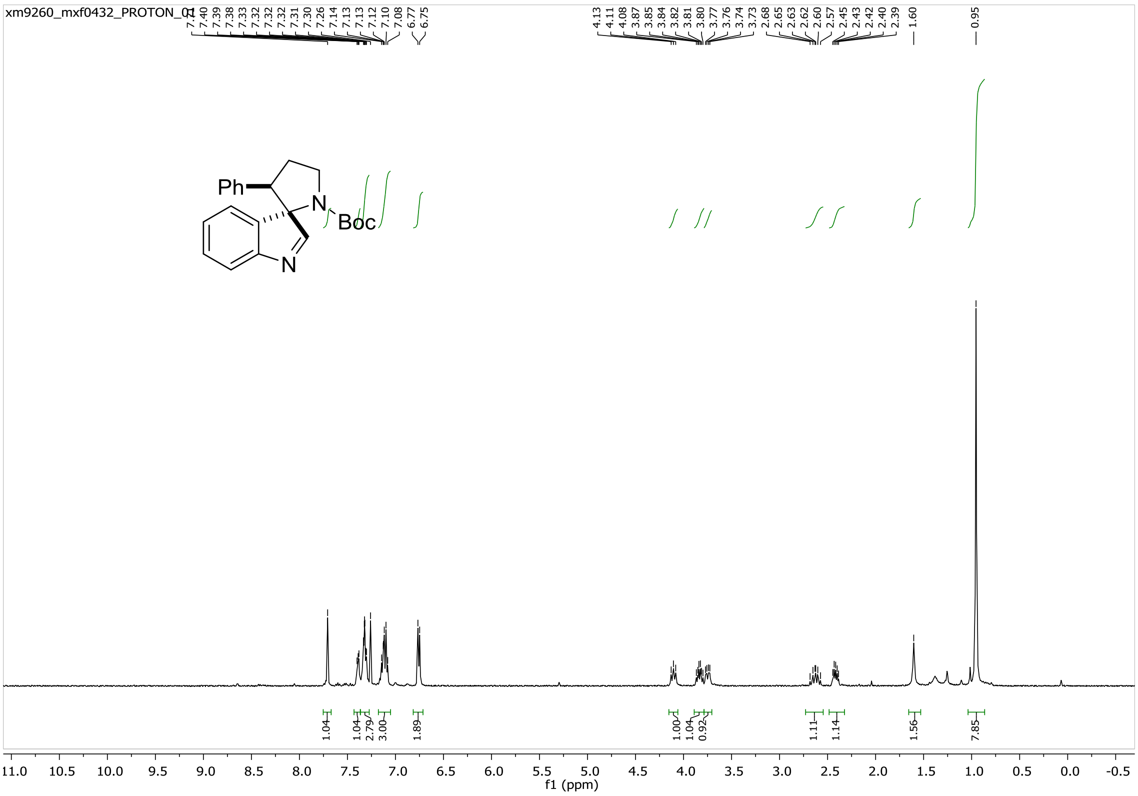

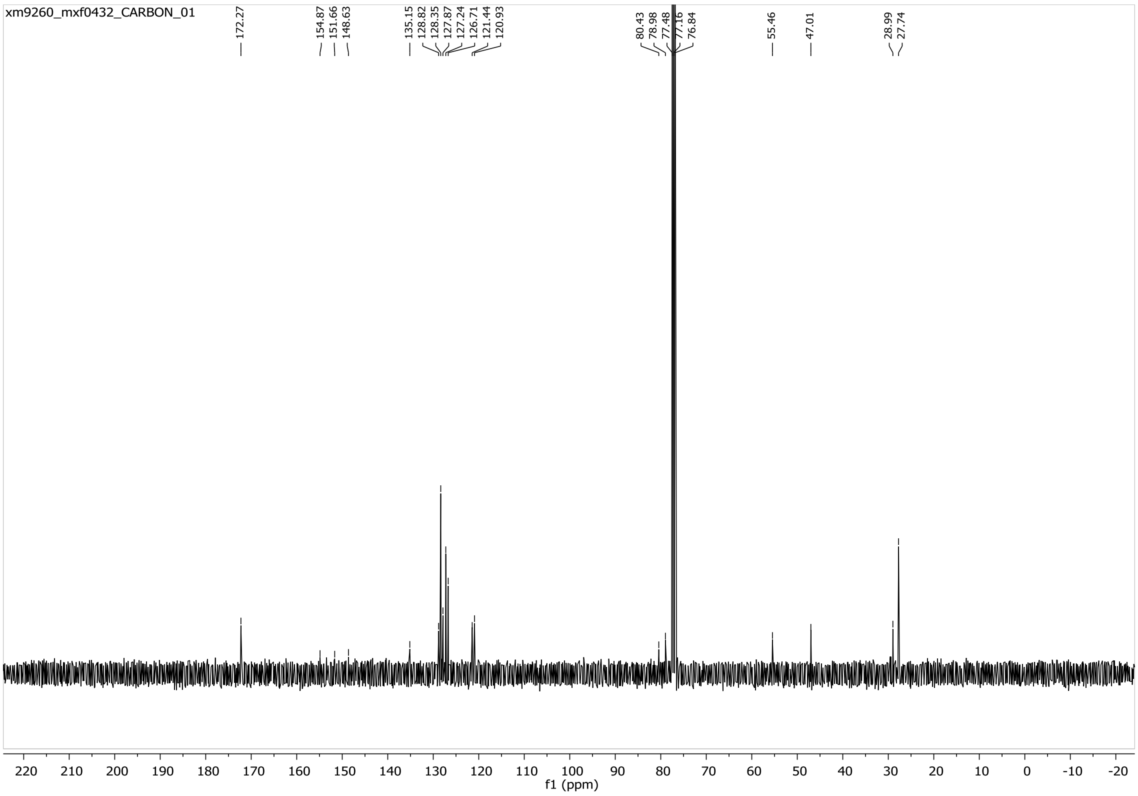

3-(4-((*tert*-Butyldimethylsilyl)oxy)phenyl)propanoic acid

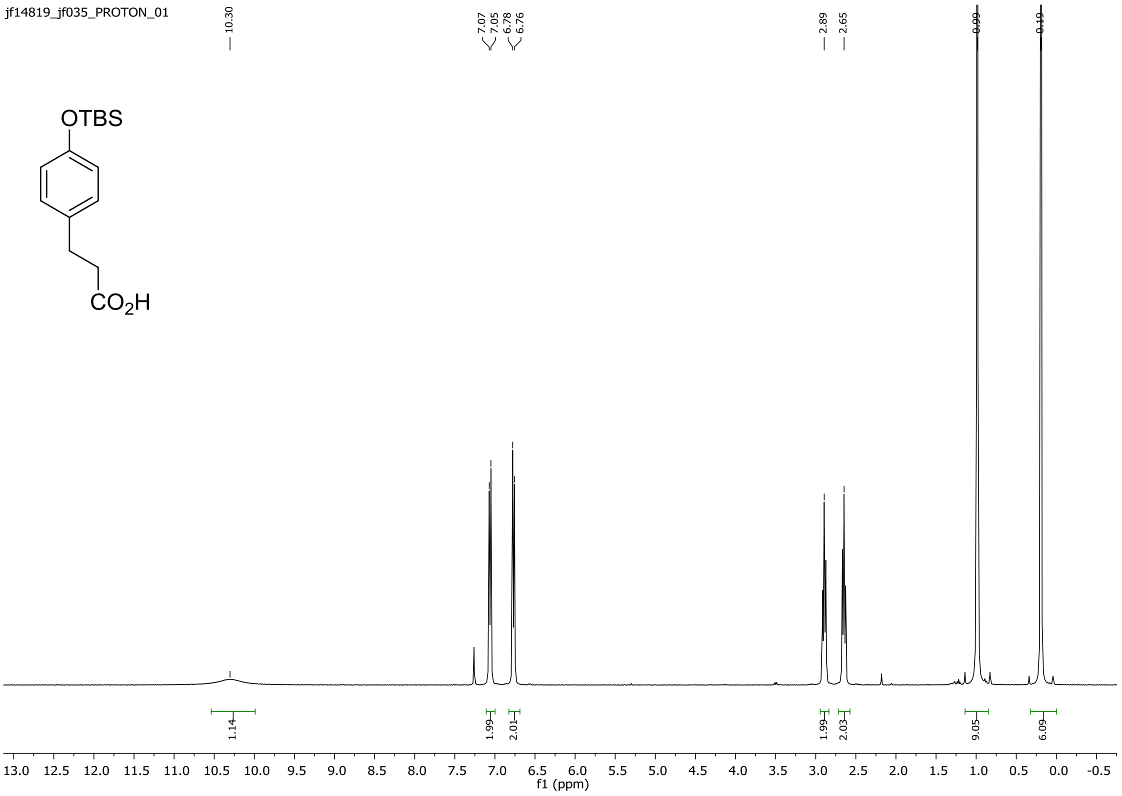

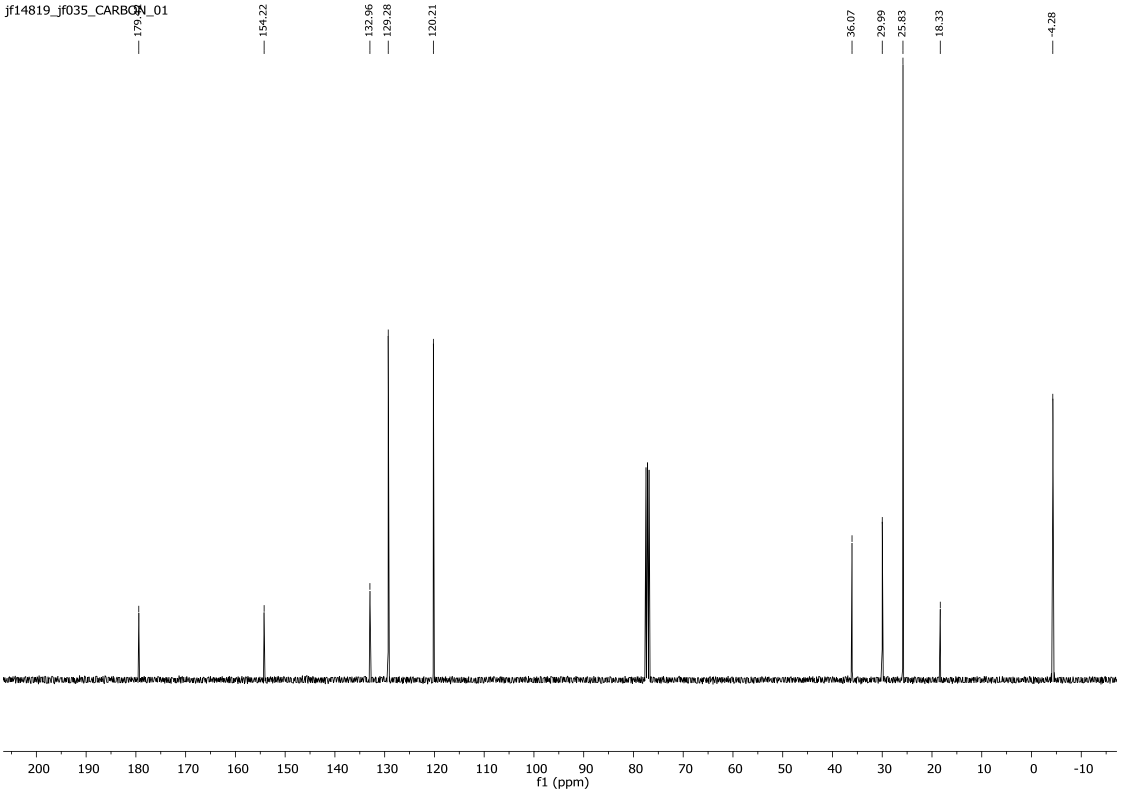

***tert*-Butyl (3-(4-((*tert*-butyldimethylsilyl)oxy)phenyl)propyl)(tosyloxy)carbamate**

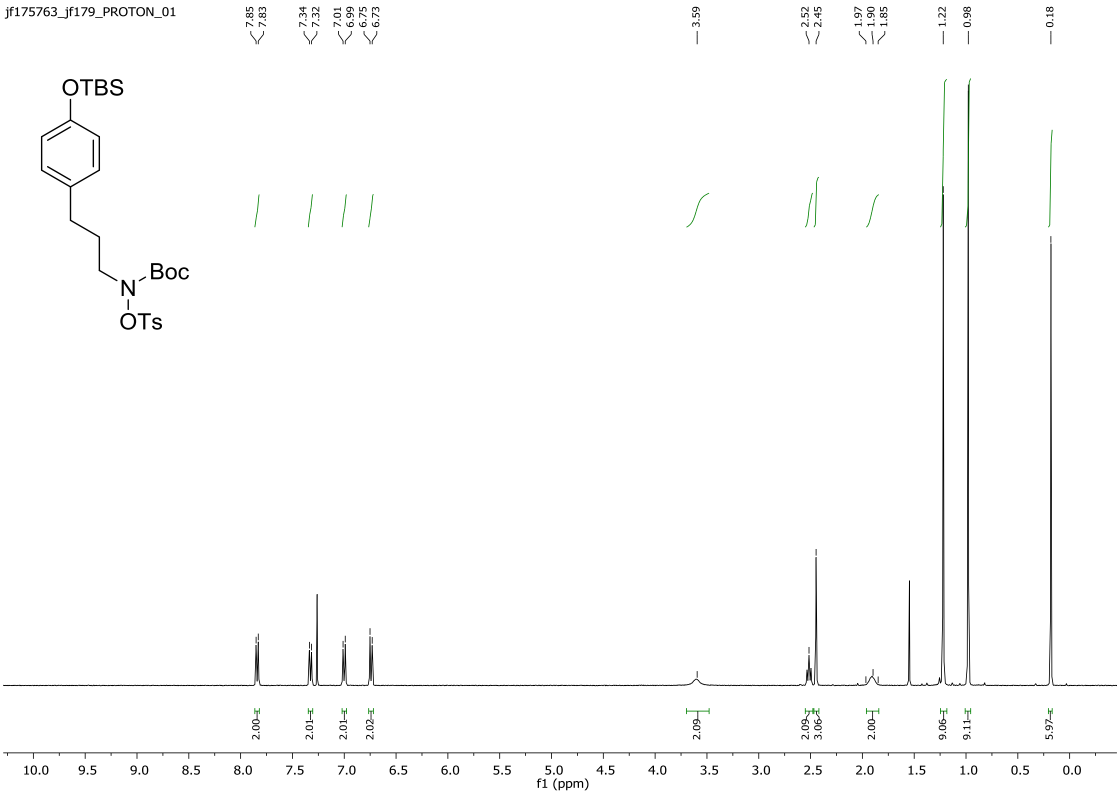

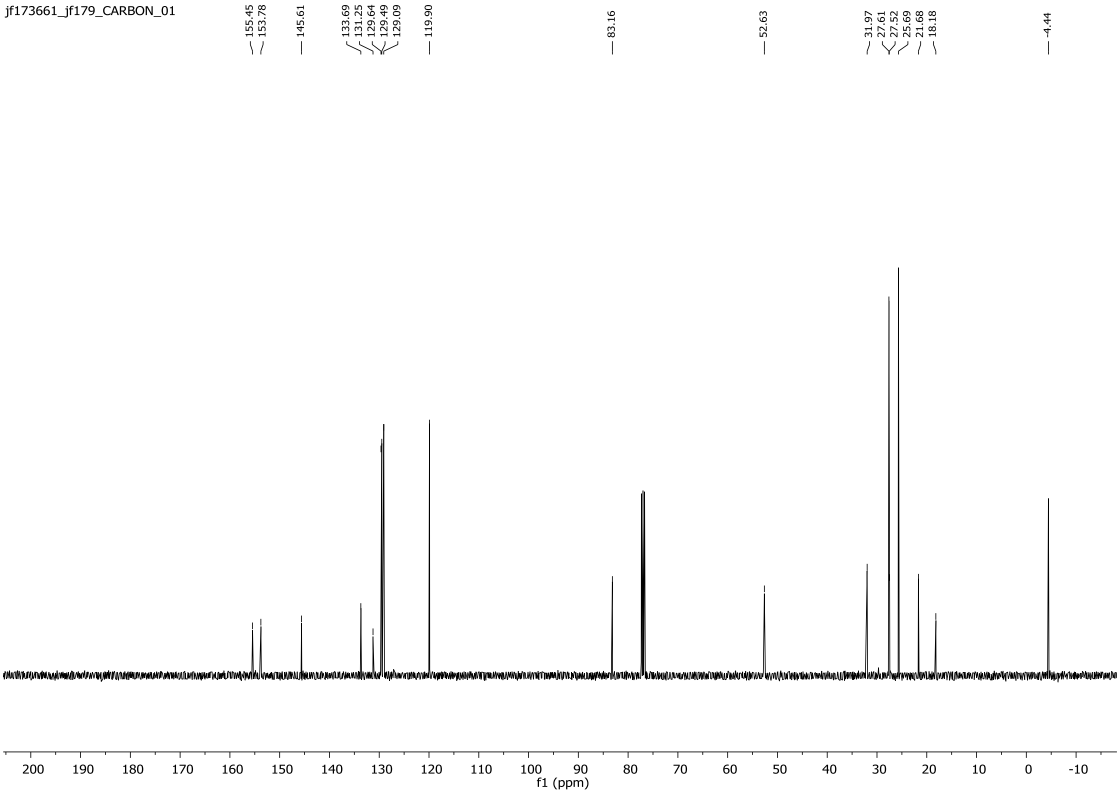

***tert*-Butyl (3-(4-hydroxyphenyl)propyl)(tosyloxy)carbamate (5a)**

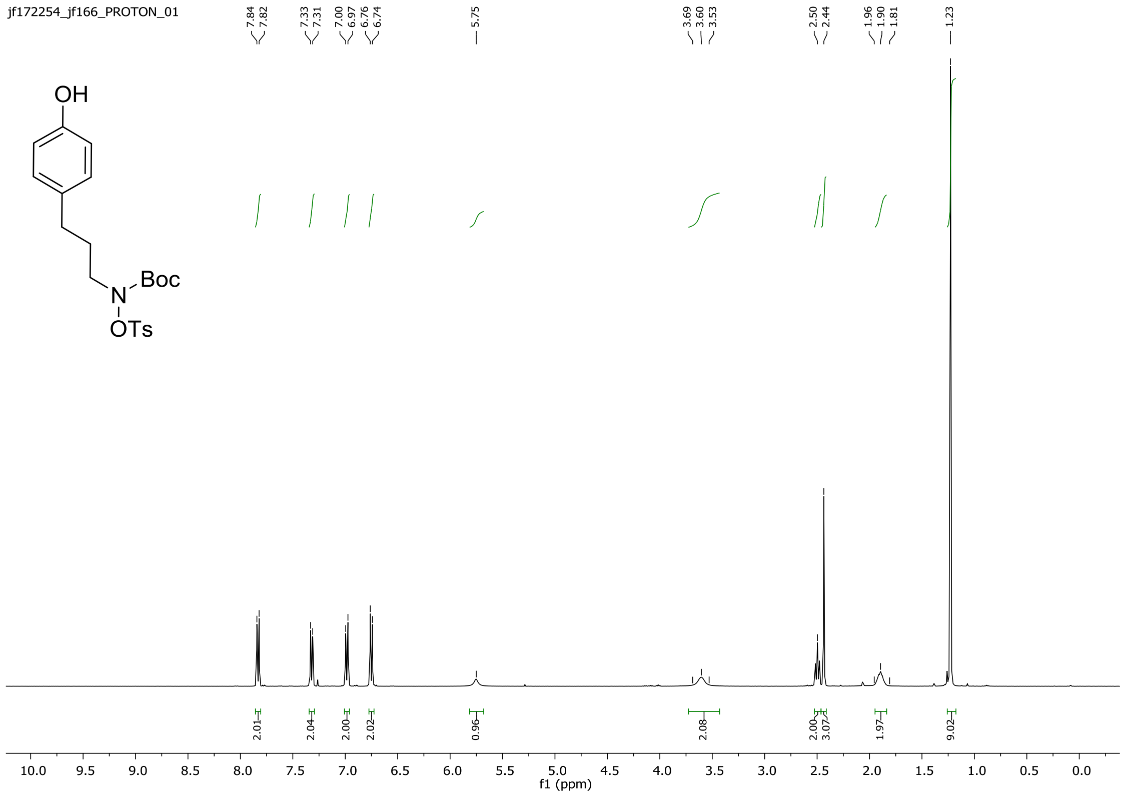

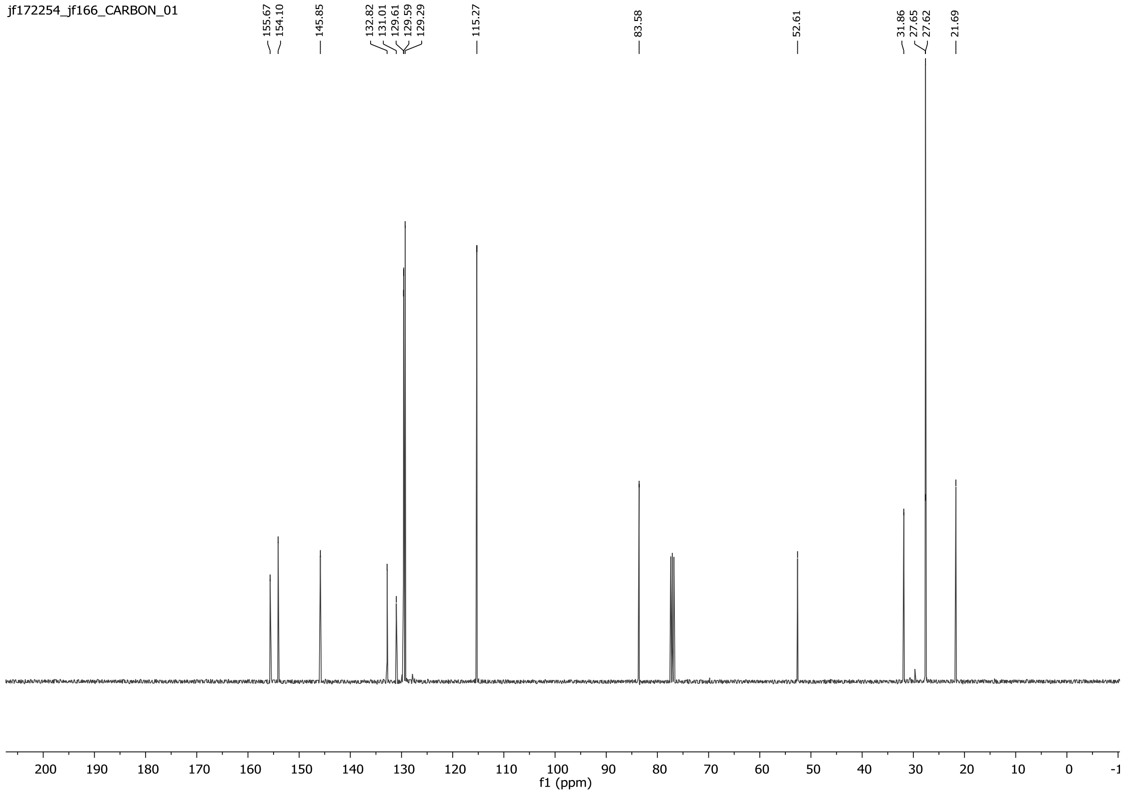

Methyl (*E*)-3-(4-(benzyloxy)-3,5-dimethylphenyl)acrylate

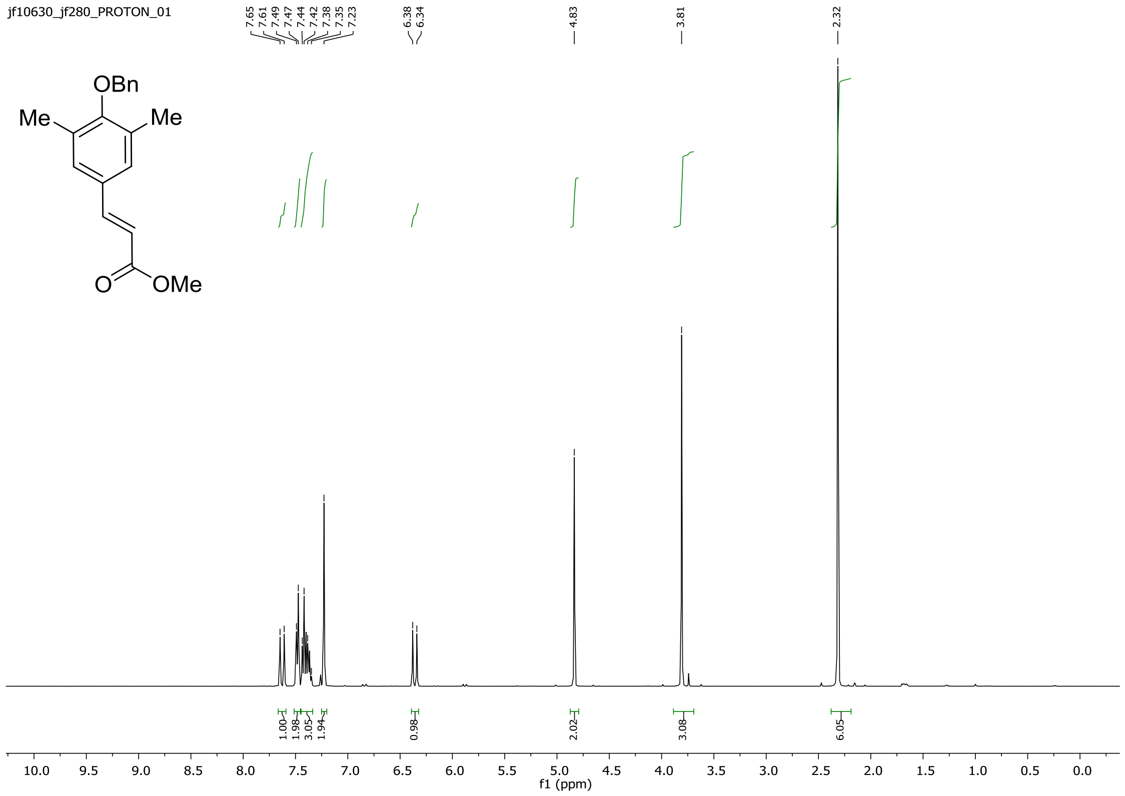

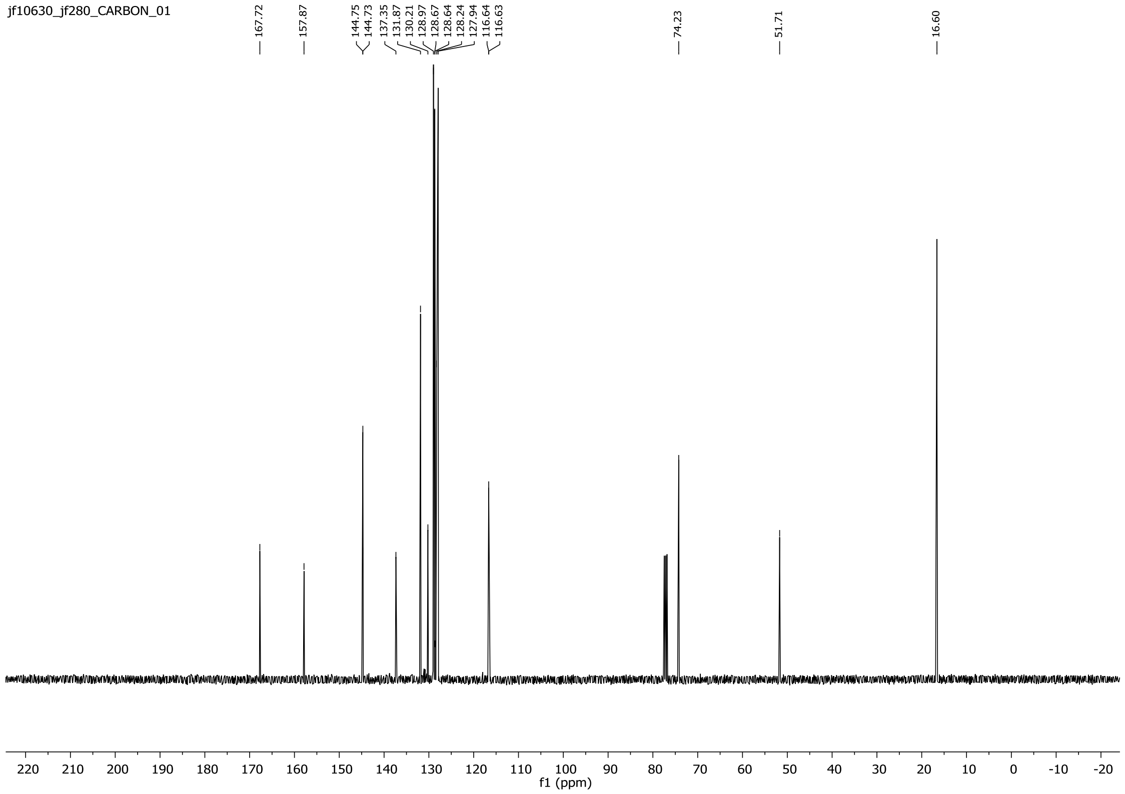

Methyl 3-(4-hydroxy-3,5-dimethylphenyl)propanoate

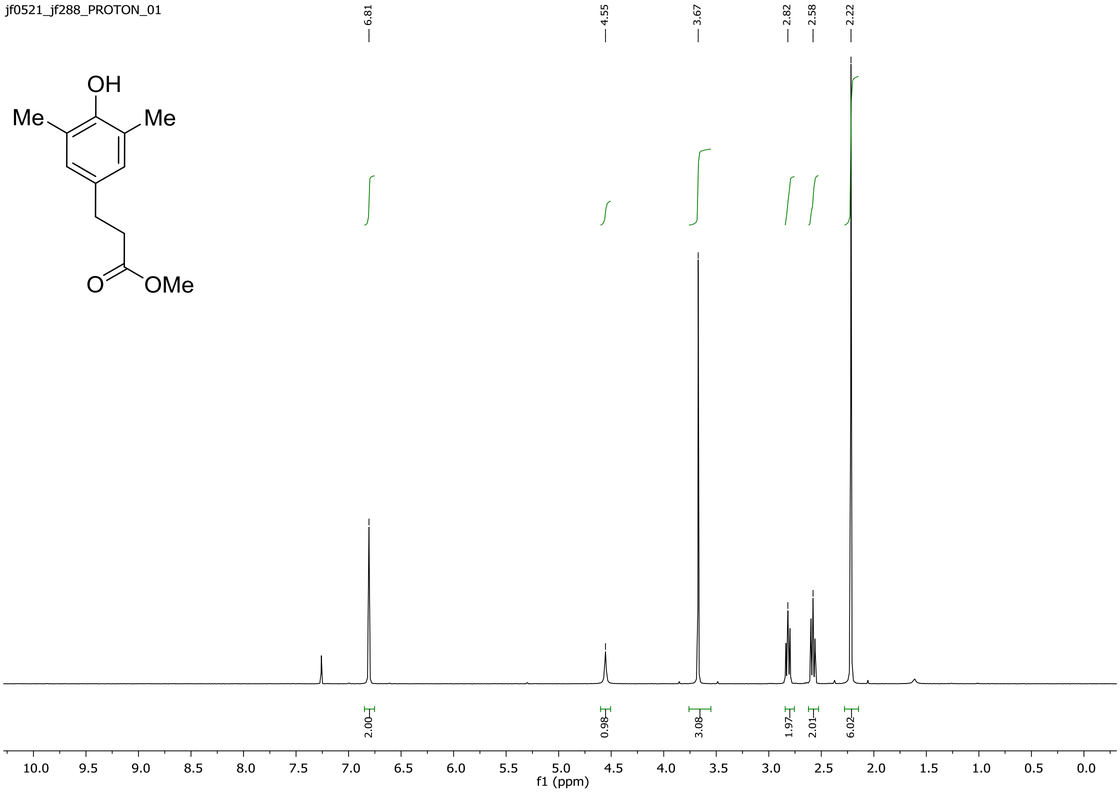

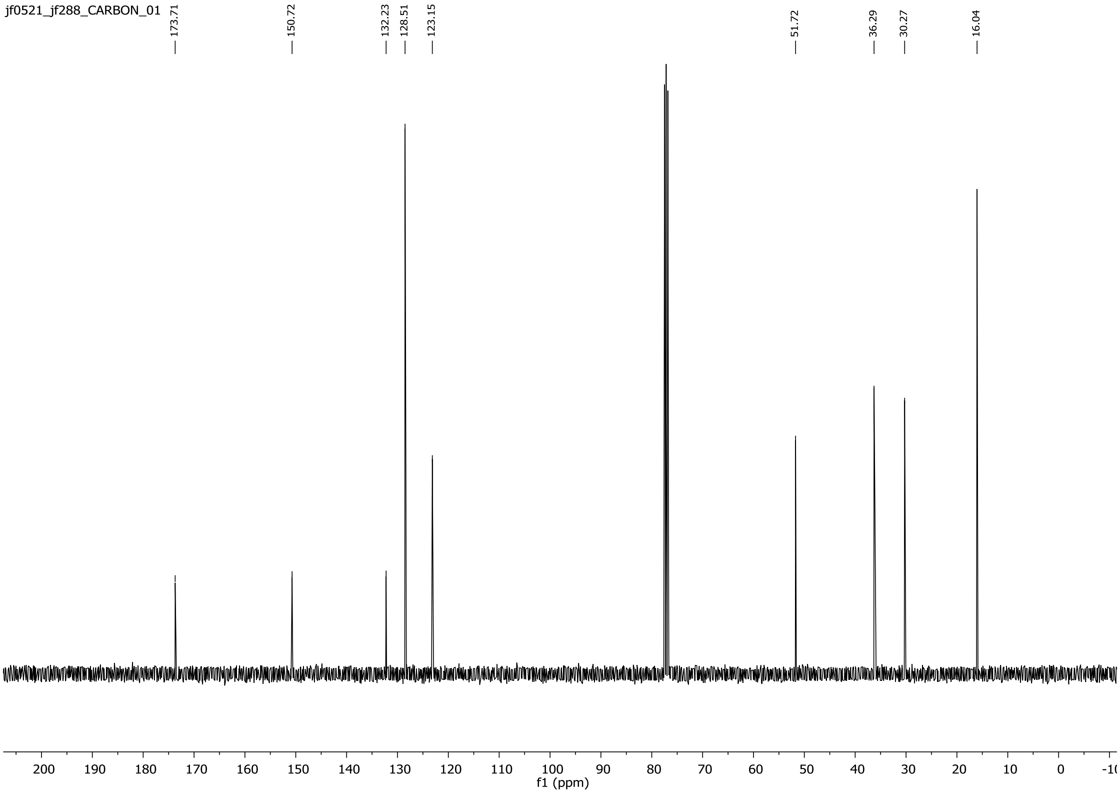

Methyl 3-(4-((*tert*-butyldimethylsilyl)oxy)-3,5-dimethylphenyl)propanoate

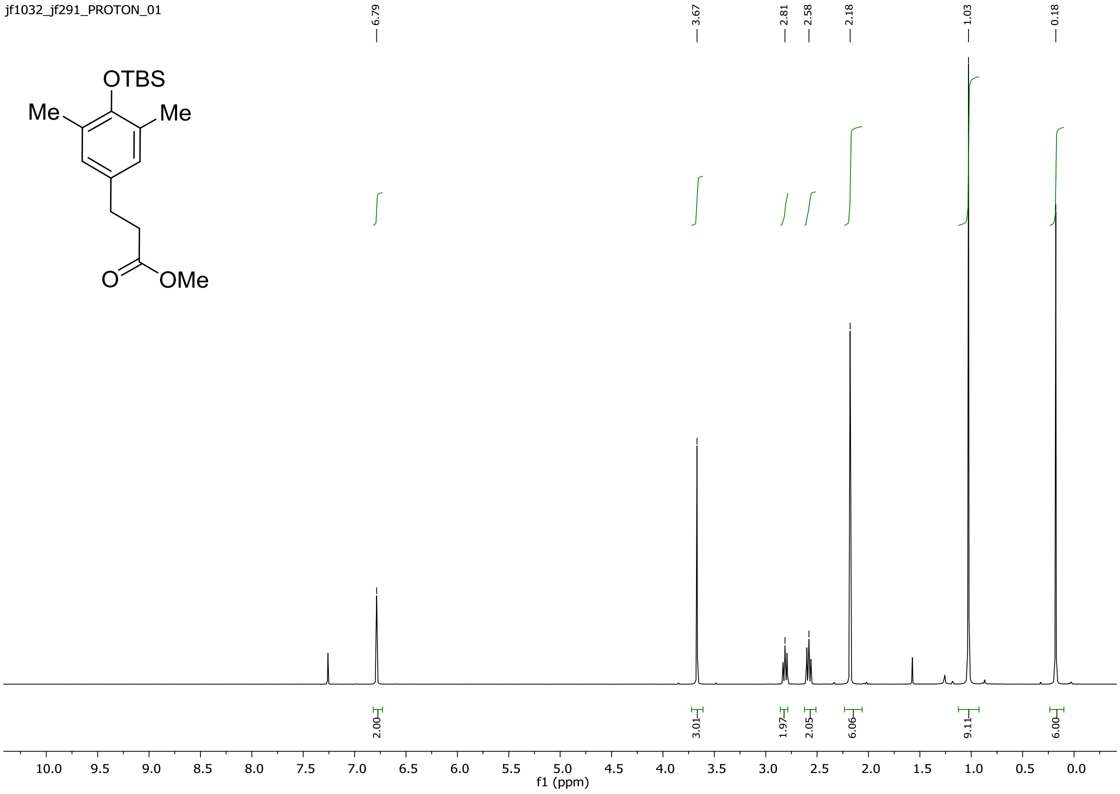

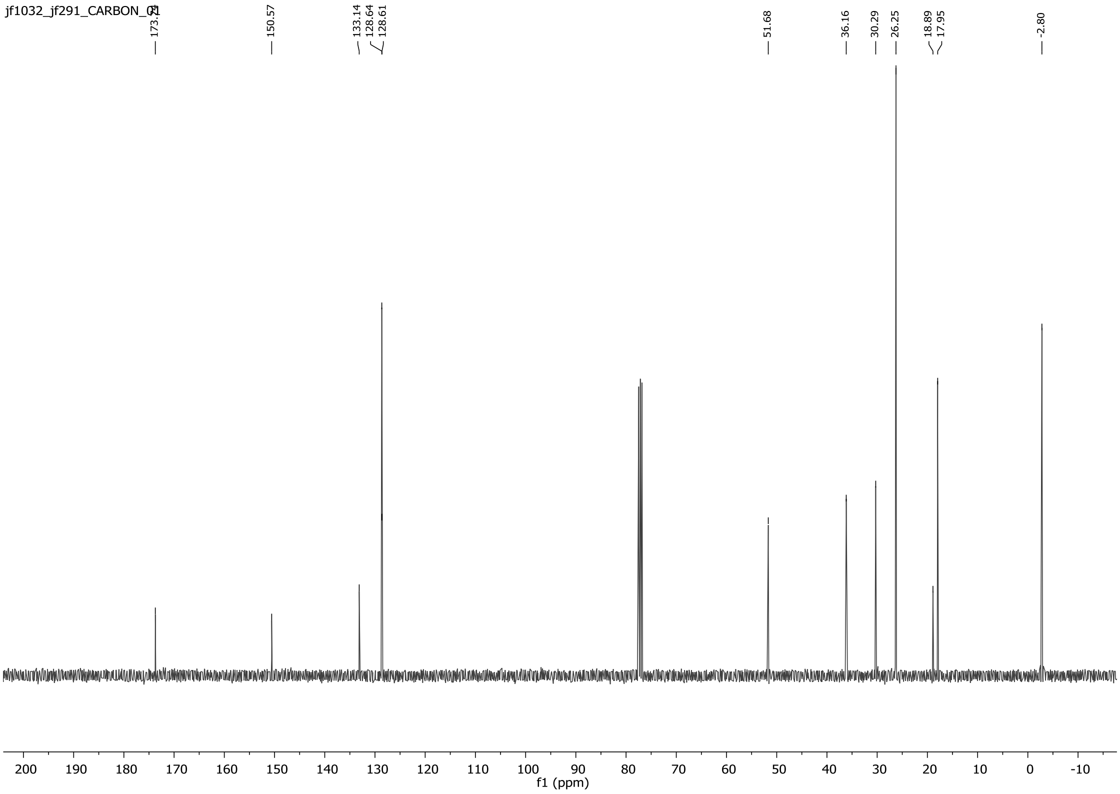

tert-Butyl(3-(4-((*tert*-butyldimethylsilyl)oxy)-3,5-dimethylphenyl)propyl)(tosyloxy) carbamate

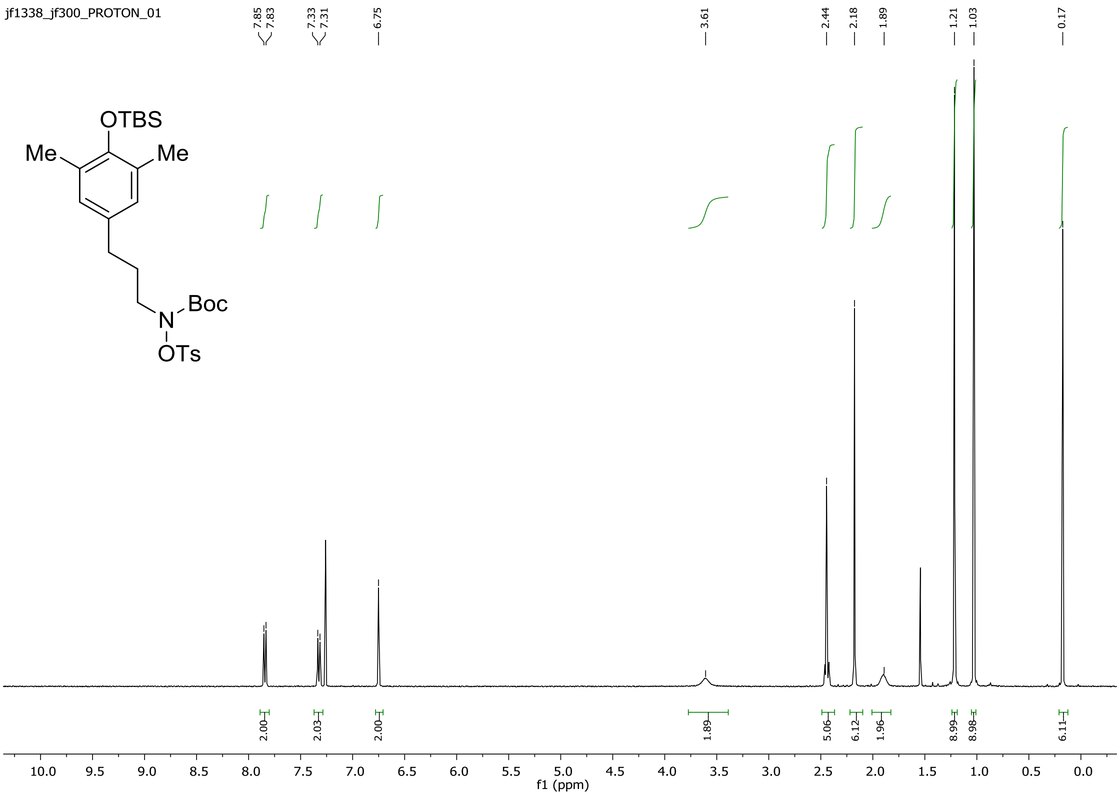

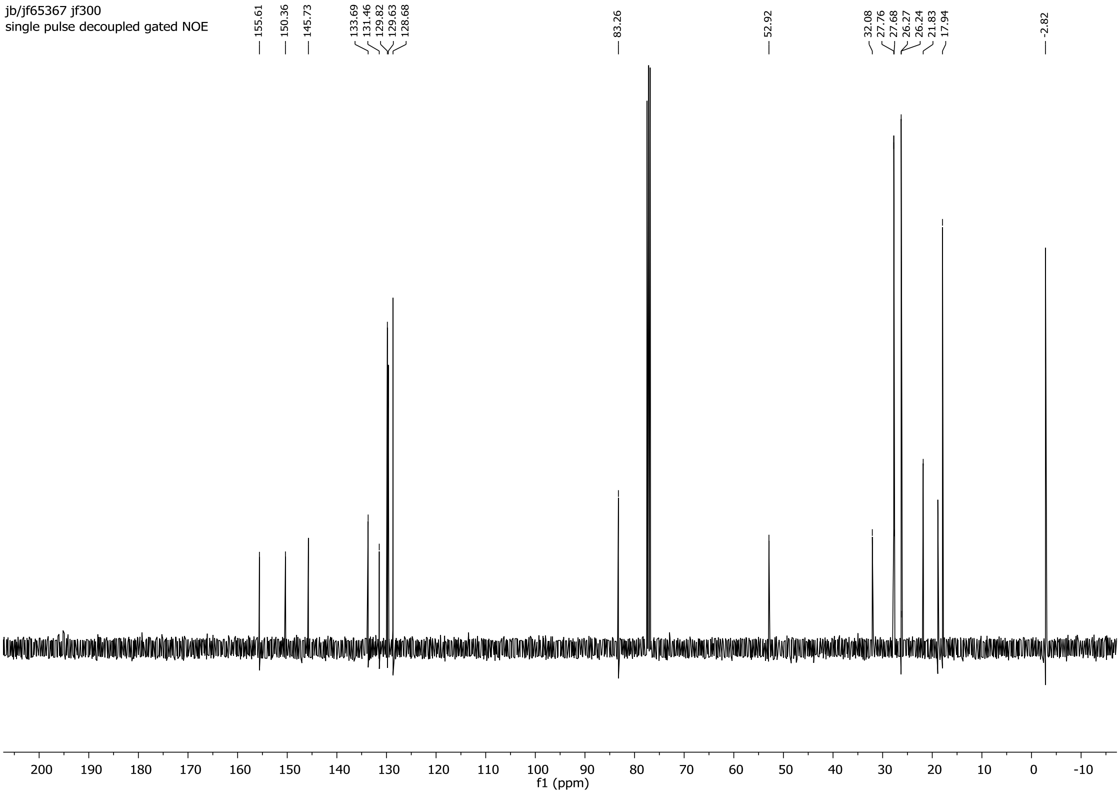

***tert*-Butyl (3-(4-hydroxy-3,5-dimethylphenyl)propyl)(tosyloxy)carbamate (5b)**

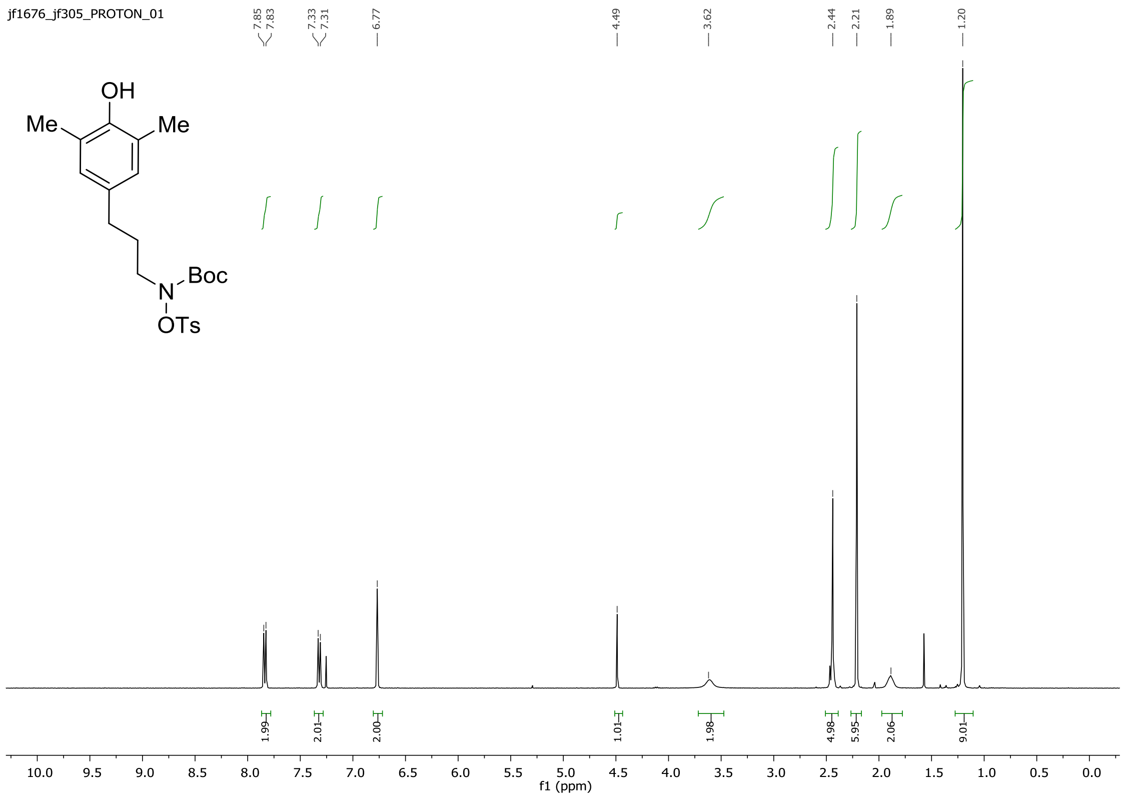

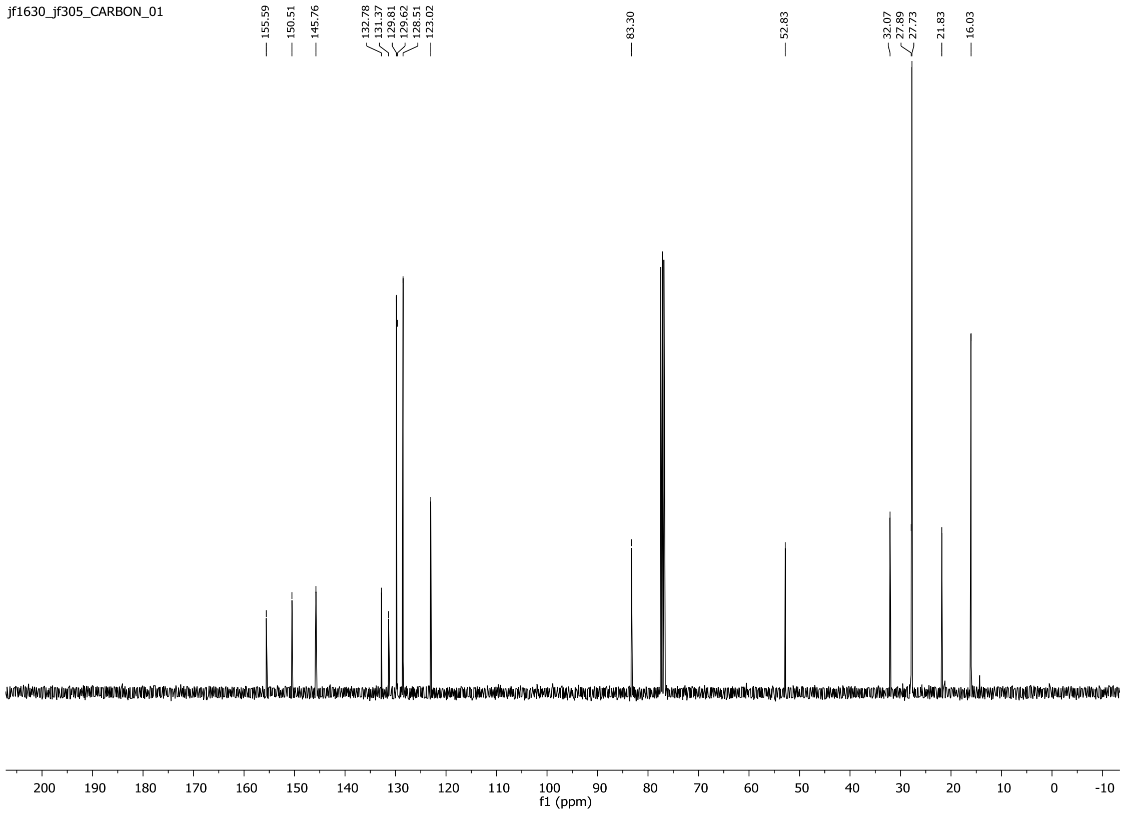

Ethyl 3-(3-bromo-4-((*tert*-butyldimethylsilyl)oxy)phenyl)propanoate

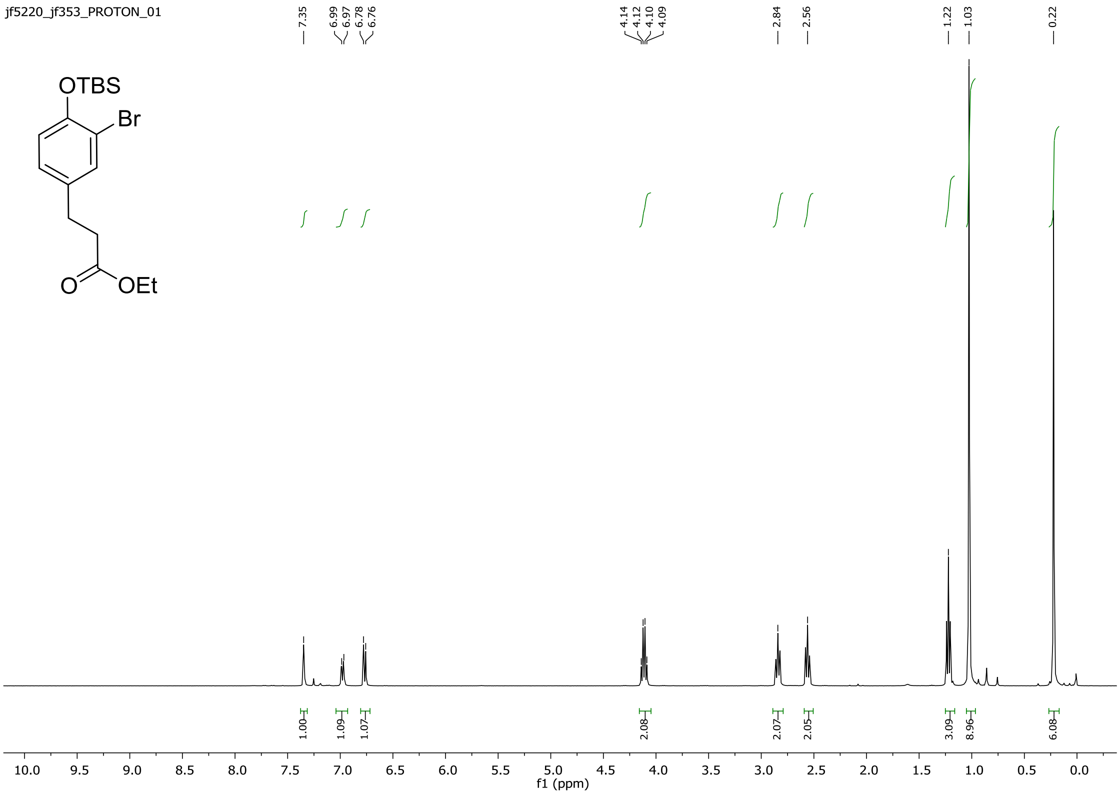

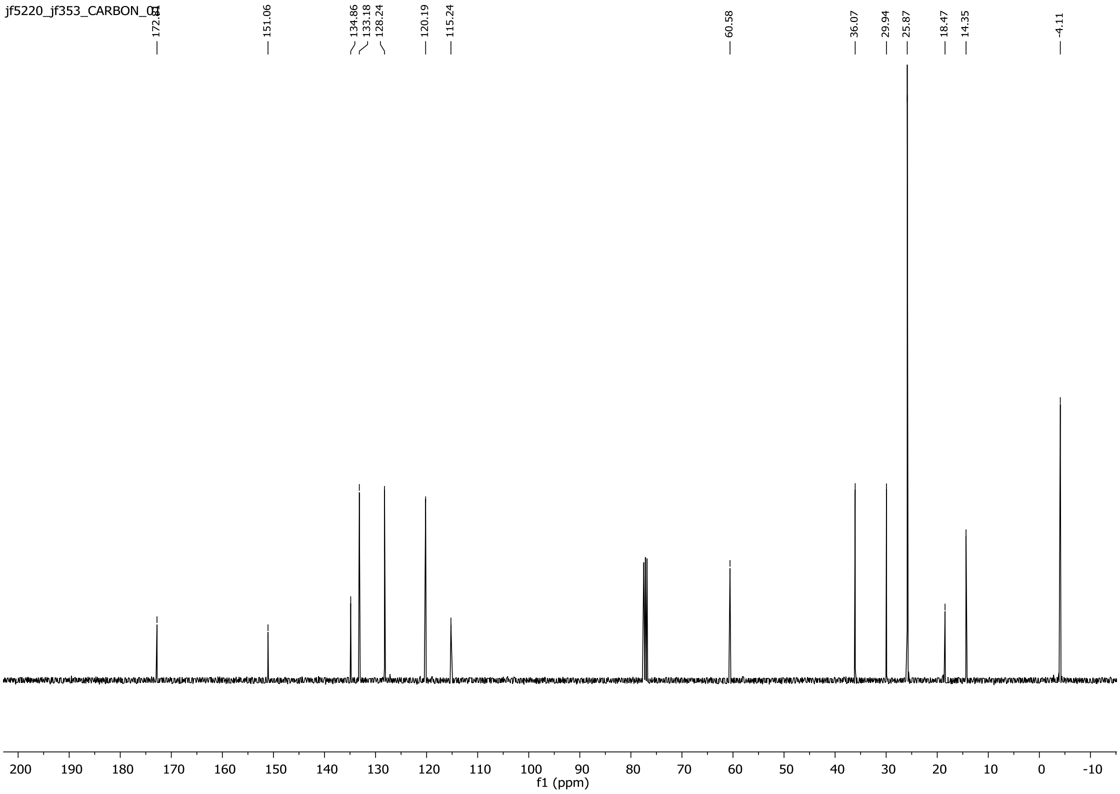

3-(3-Bromo-4-((*tert*-butyldimethylsilyl)oxy)phenyl)propan-1-ol

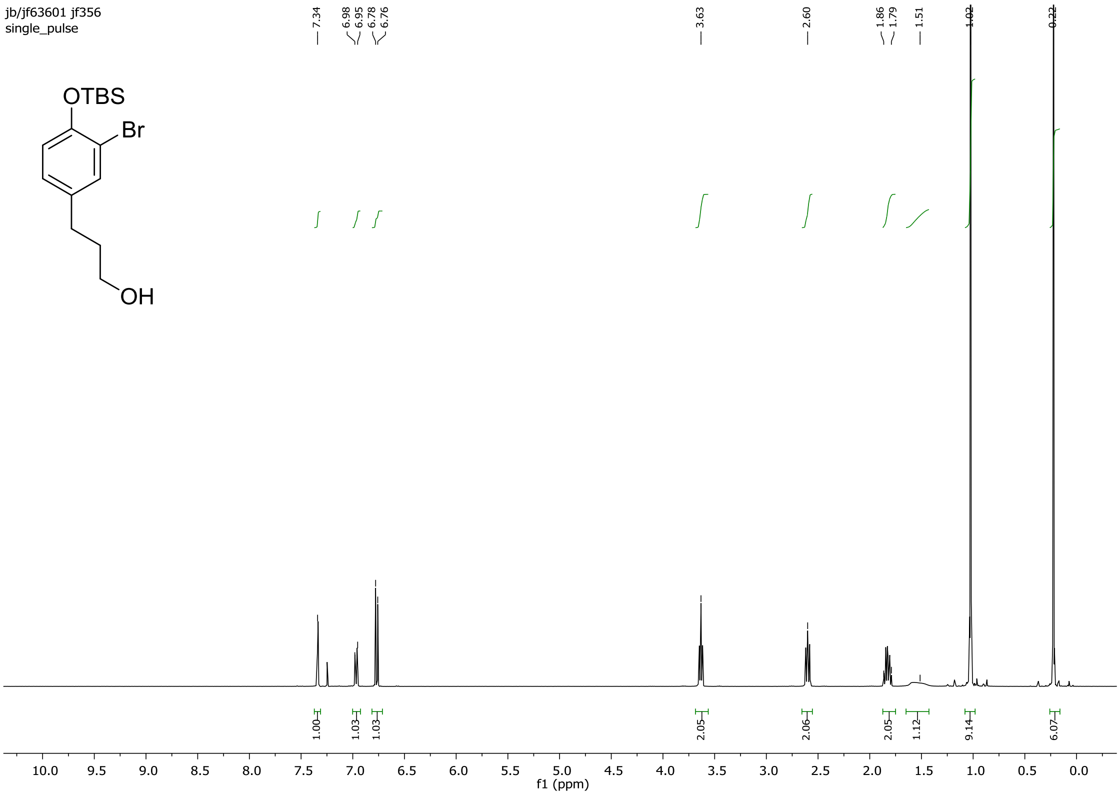

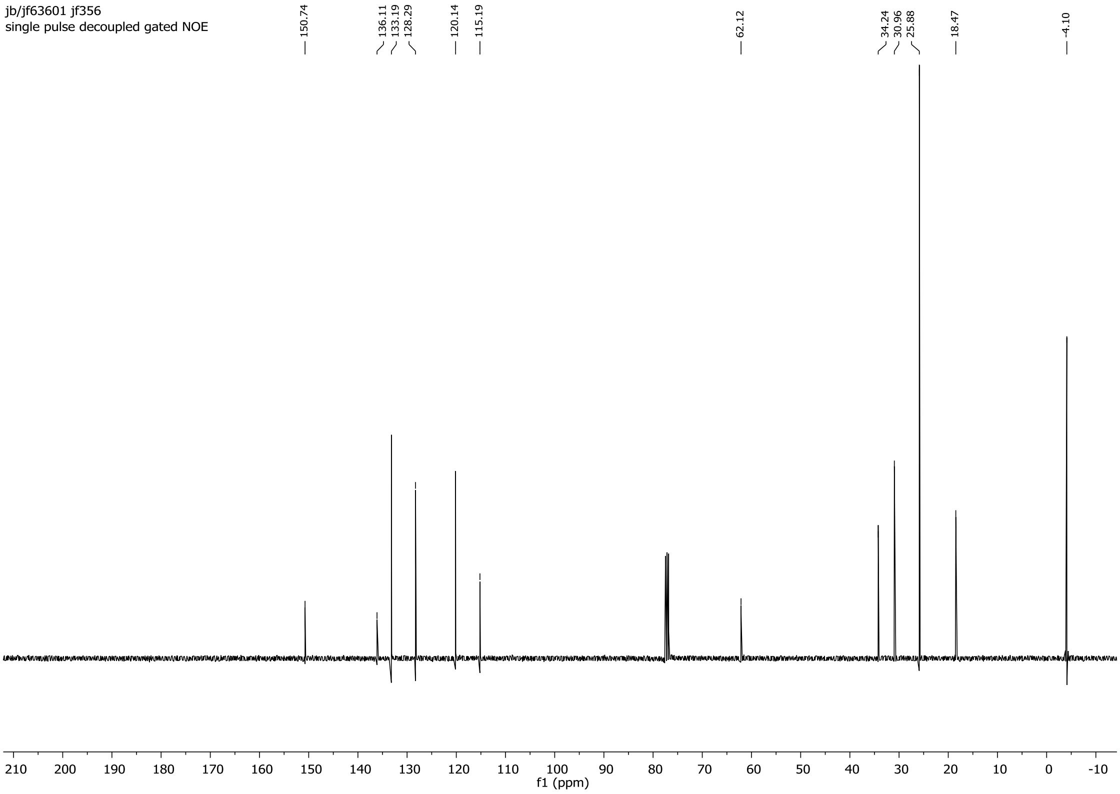

tert-Butyl(3-(3-bromo-4-((_tert_-butyldimethylsilyl)oxy)phenyl)propyl)(tosyloxy) carbamate

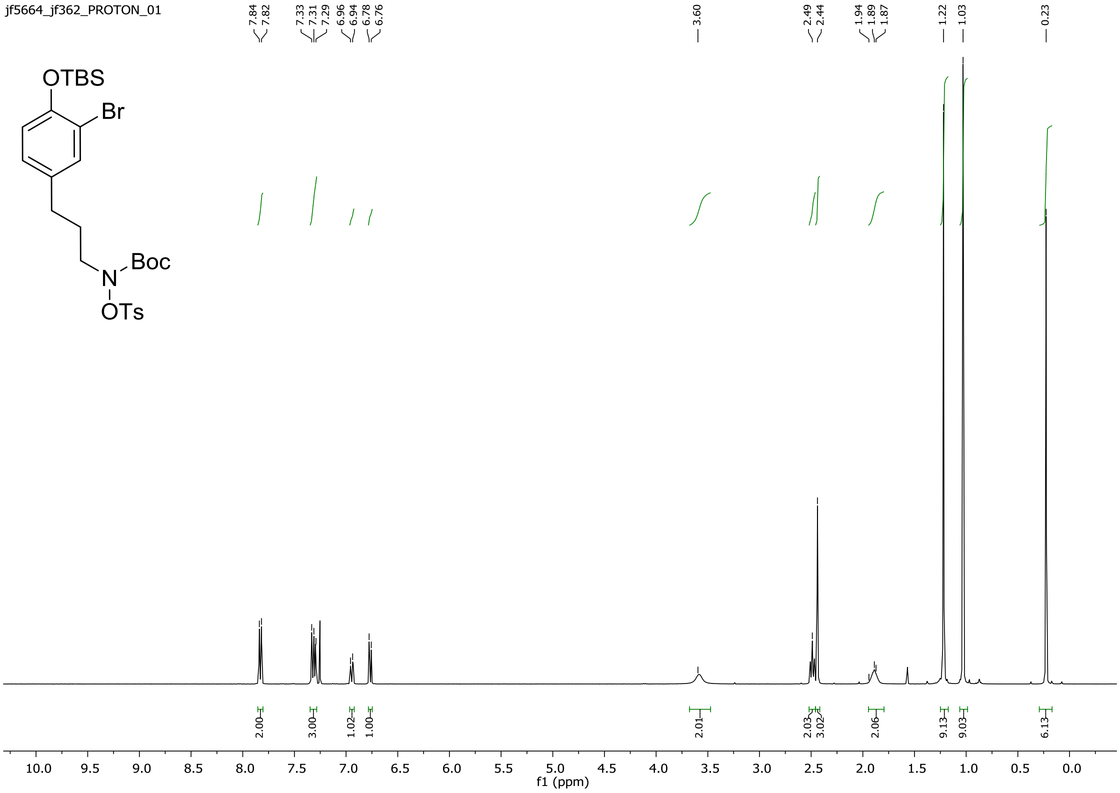

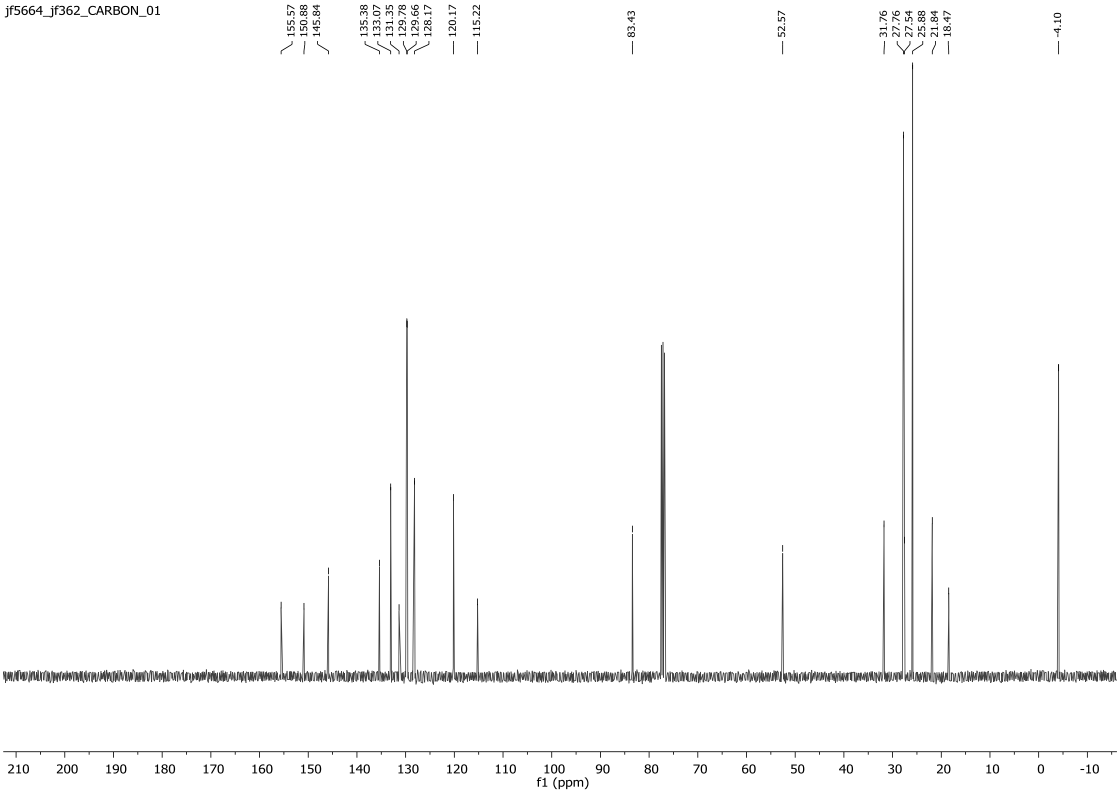

***tert*-Butyl (3-(3-bromo-4-hydroxyphenyl)propyl)(tosyloxy)carbamate (5c)**

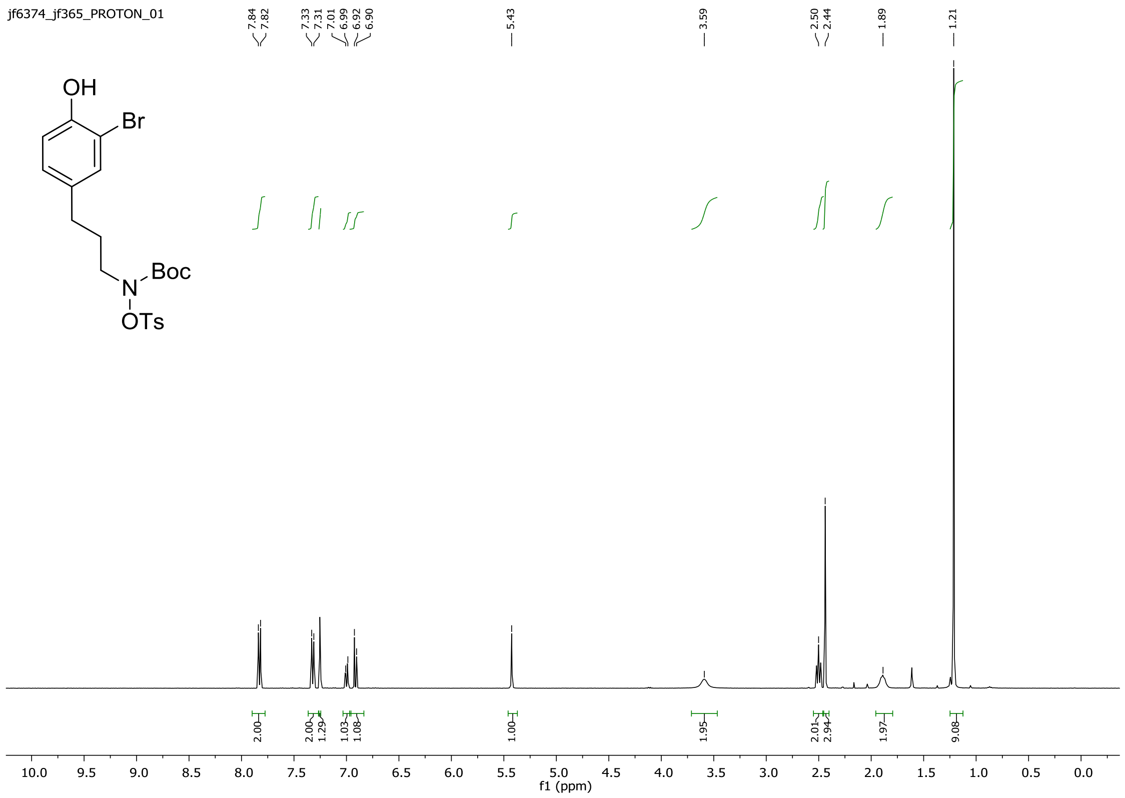

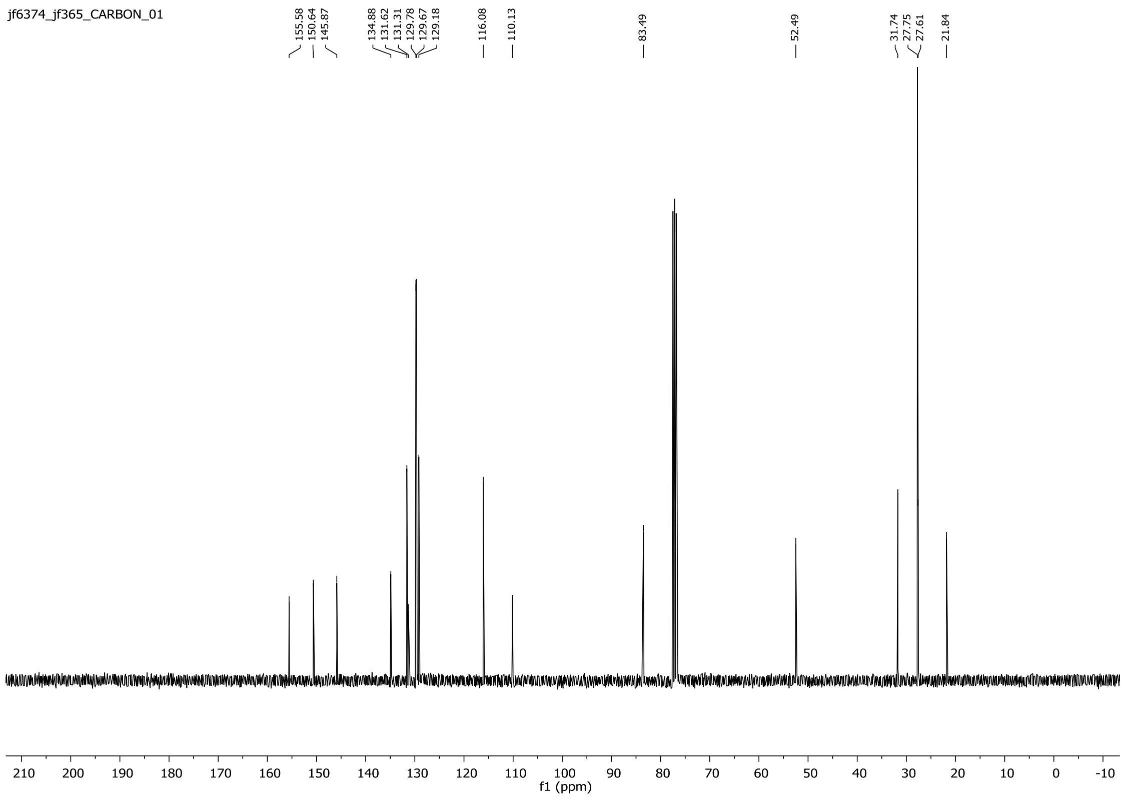

Ethyl 3-(4-((*tert*-butyldimethylsilyl)oxy)phenyl)pentanoate

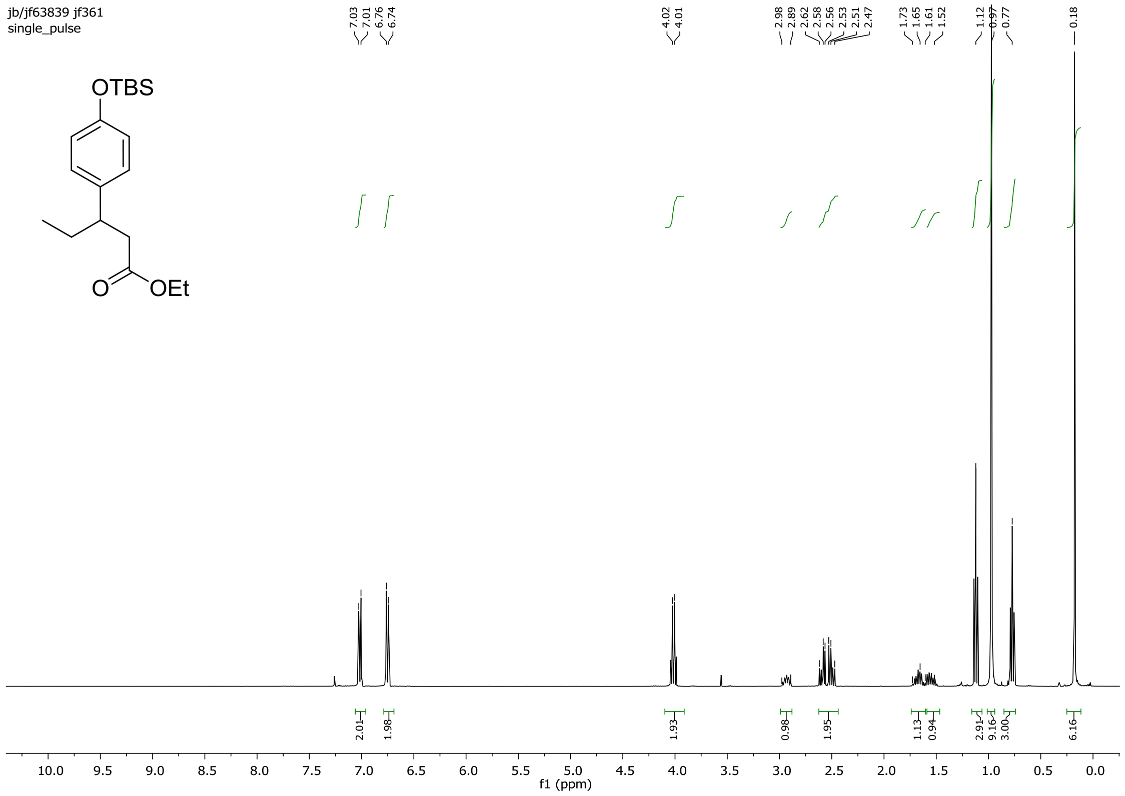

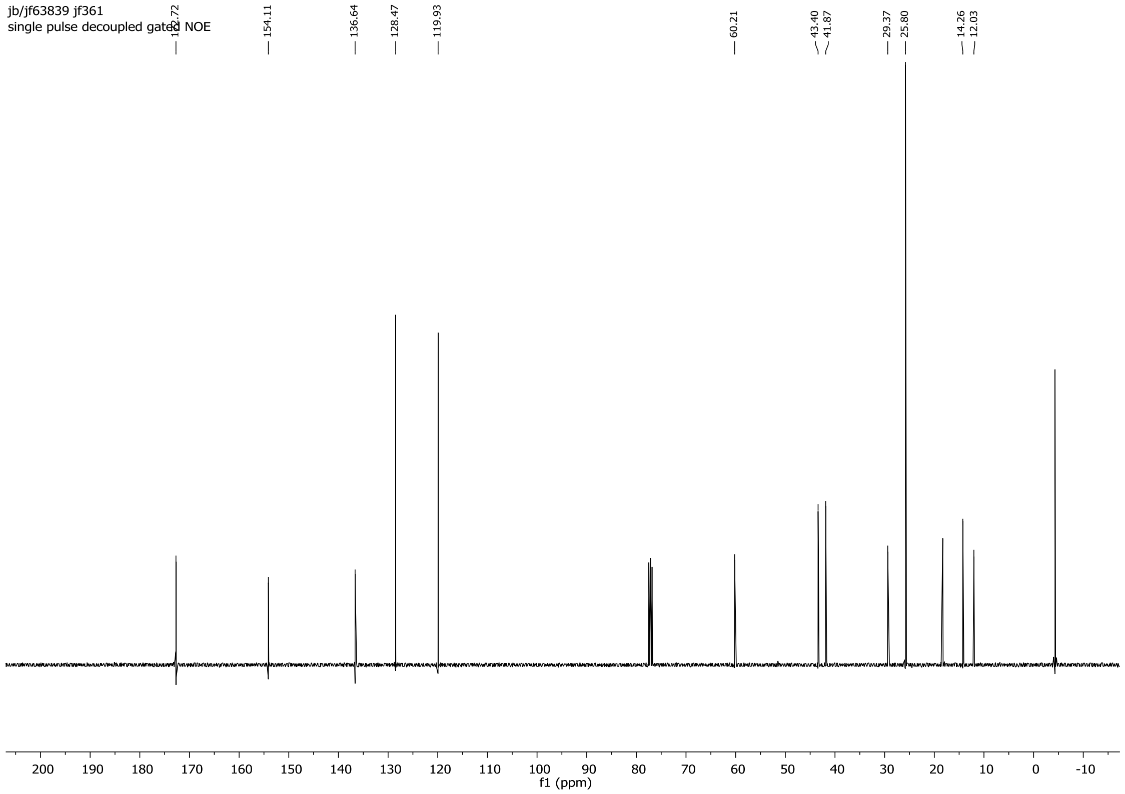

3-(4-((*tert*-Butyldimethylsilyl)oxy)phenyl)pentan-1-ol

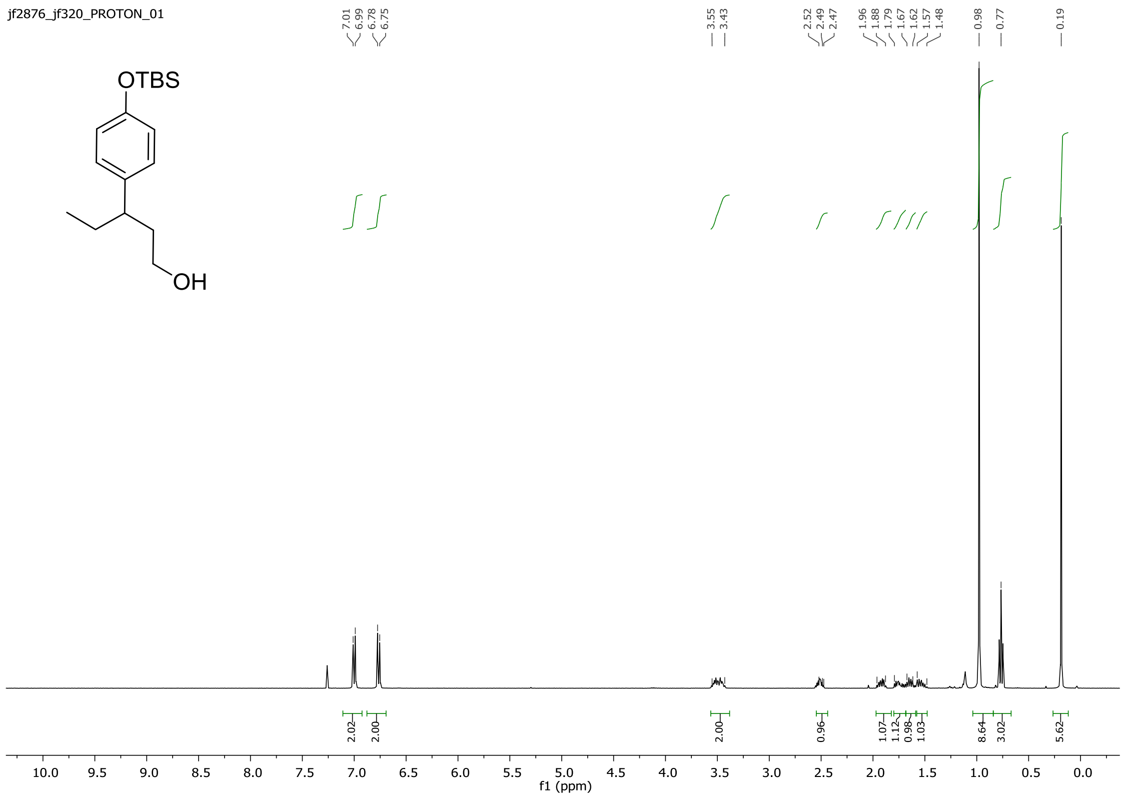

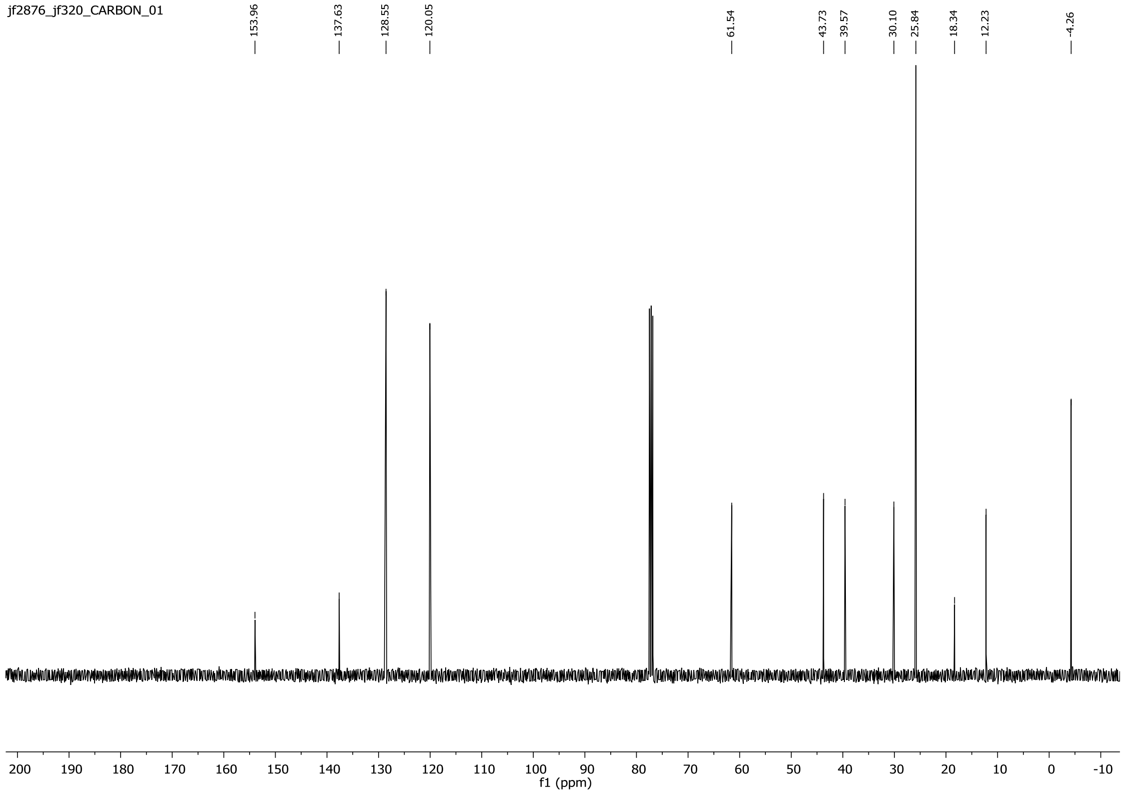

tert-Butyl (3-(4-((*tert*-butyldimethylsilyl)oxy)phenyl)pentyl)(tosyloxy)carbamate

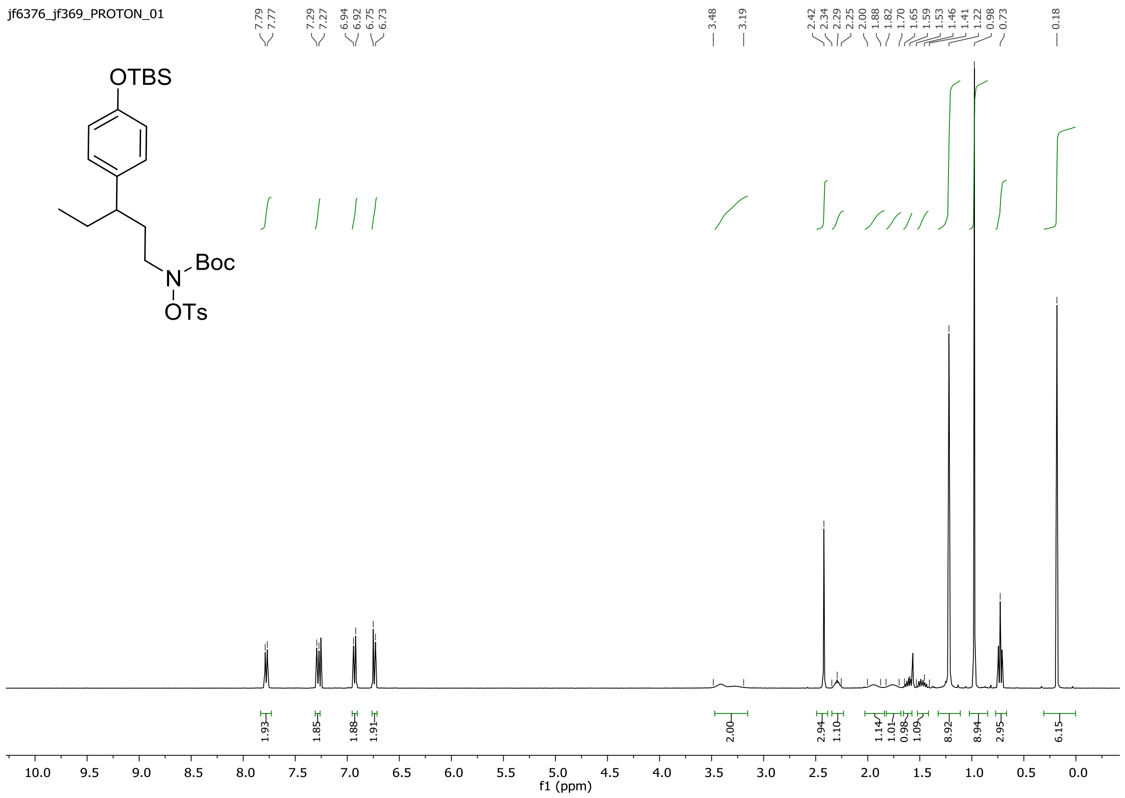

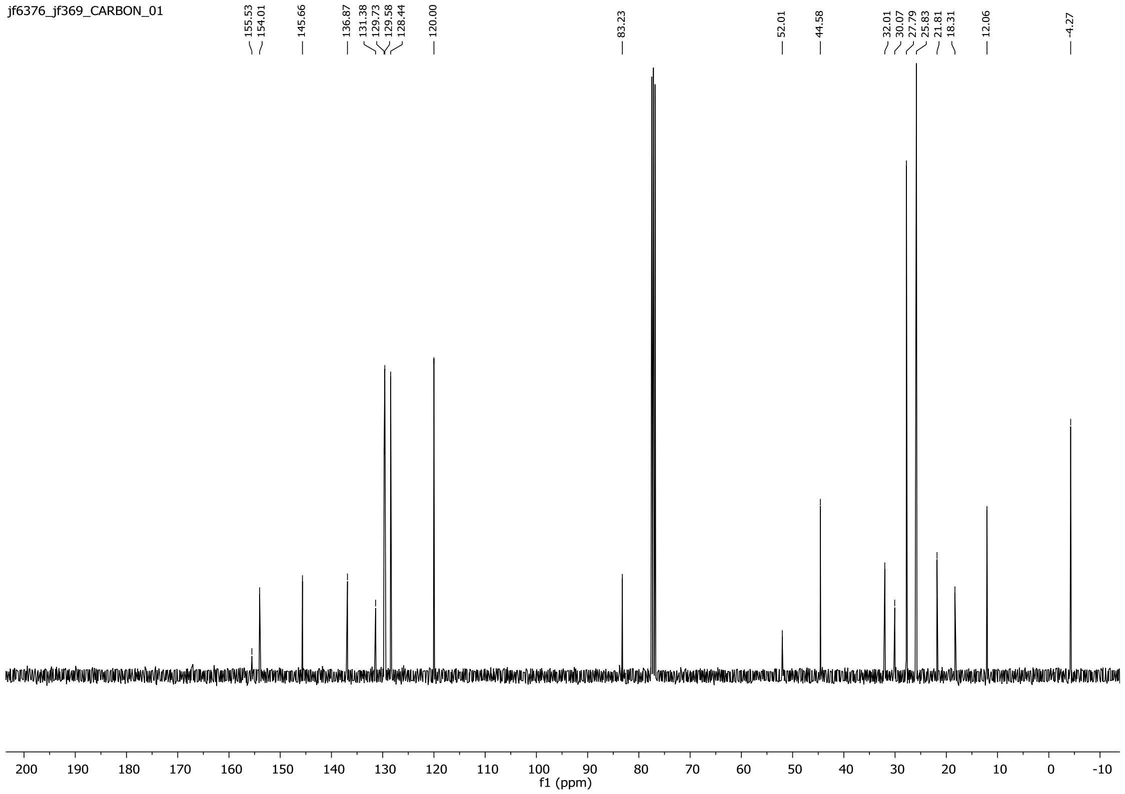

***tert*-Butyl (3-(4-hydroxyphenyl)pentyl)(tosyloxy)carbamate (5d)**

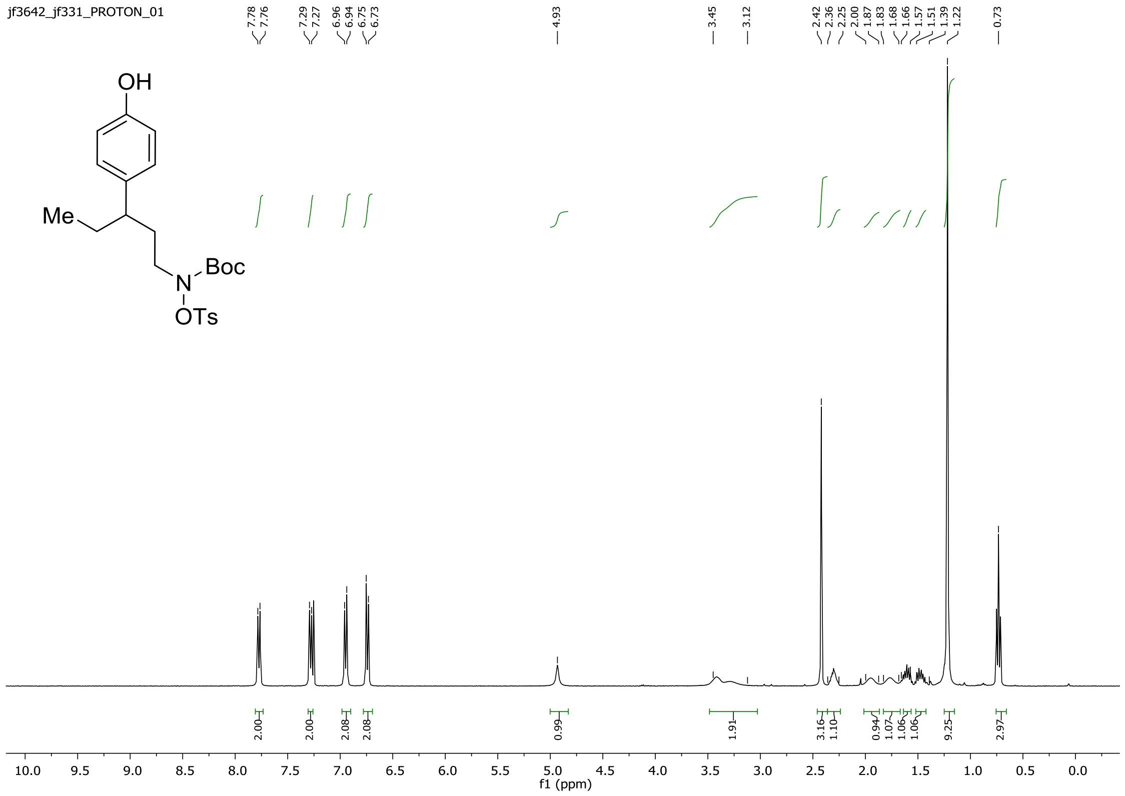

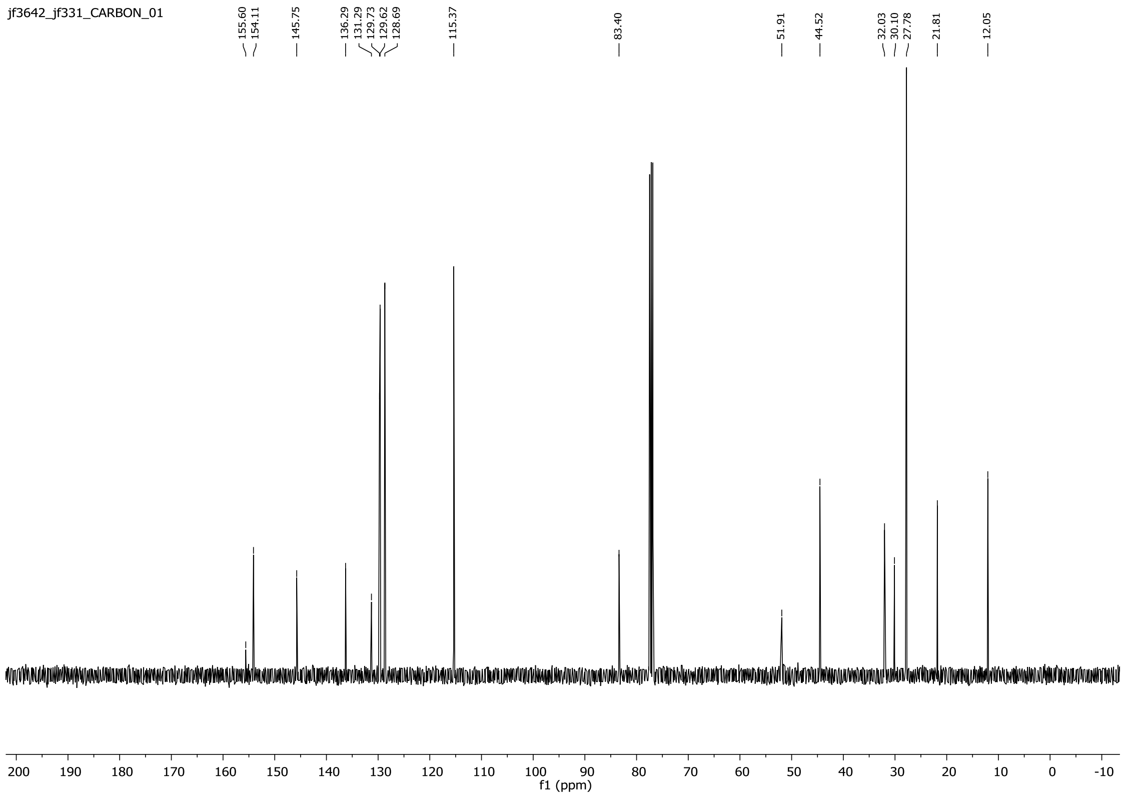

3-(4-((*tert*-Butyldimethylsilyl)oxy)naphthalen-1-yl)propan-1-ol

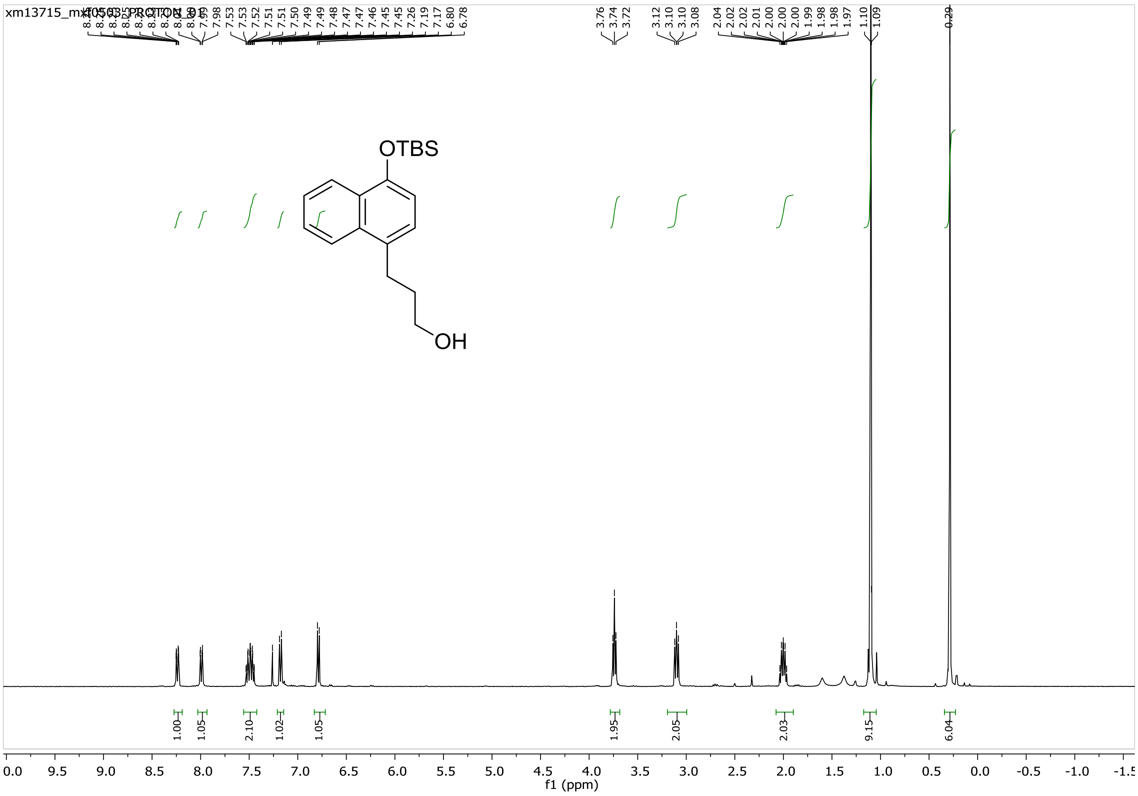

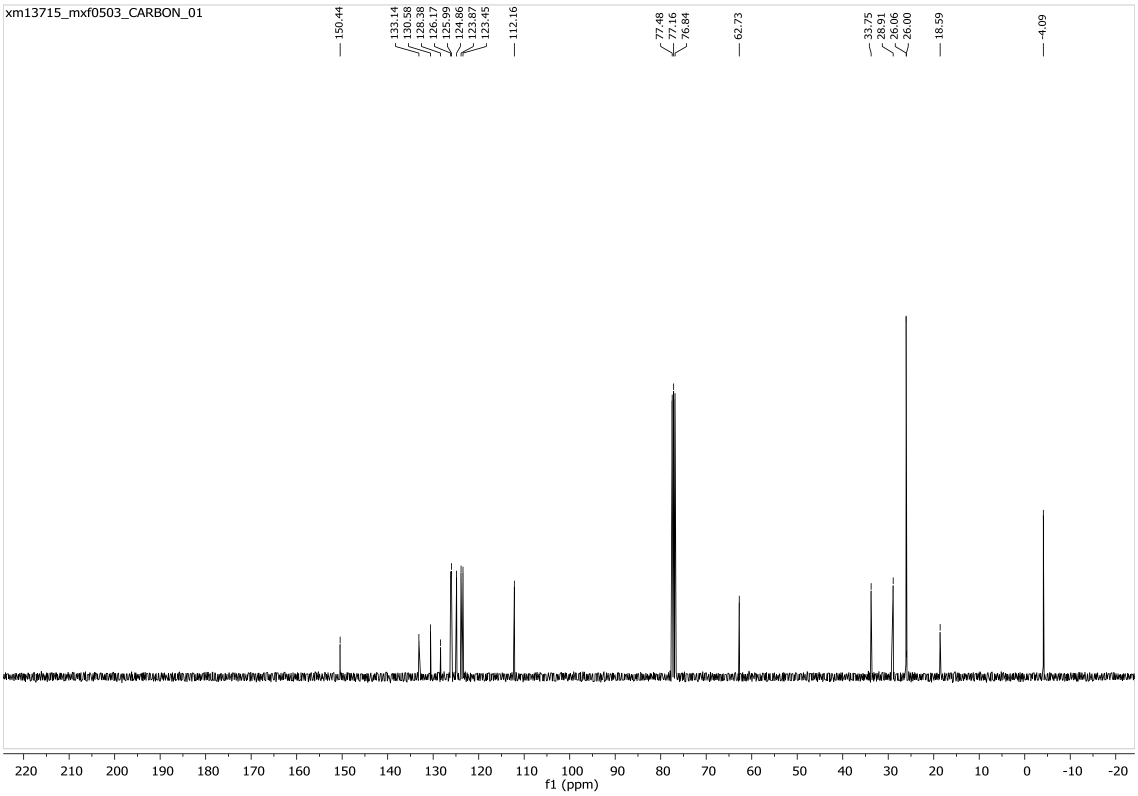

***tert*-Butyl (3-(4-((*tert*-butyldimethylsilyl)oxy)naphthalen-1-yl)propyl)(tosyloxy) carbamate**

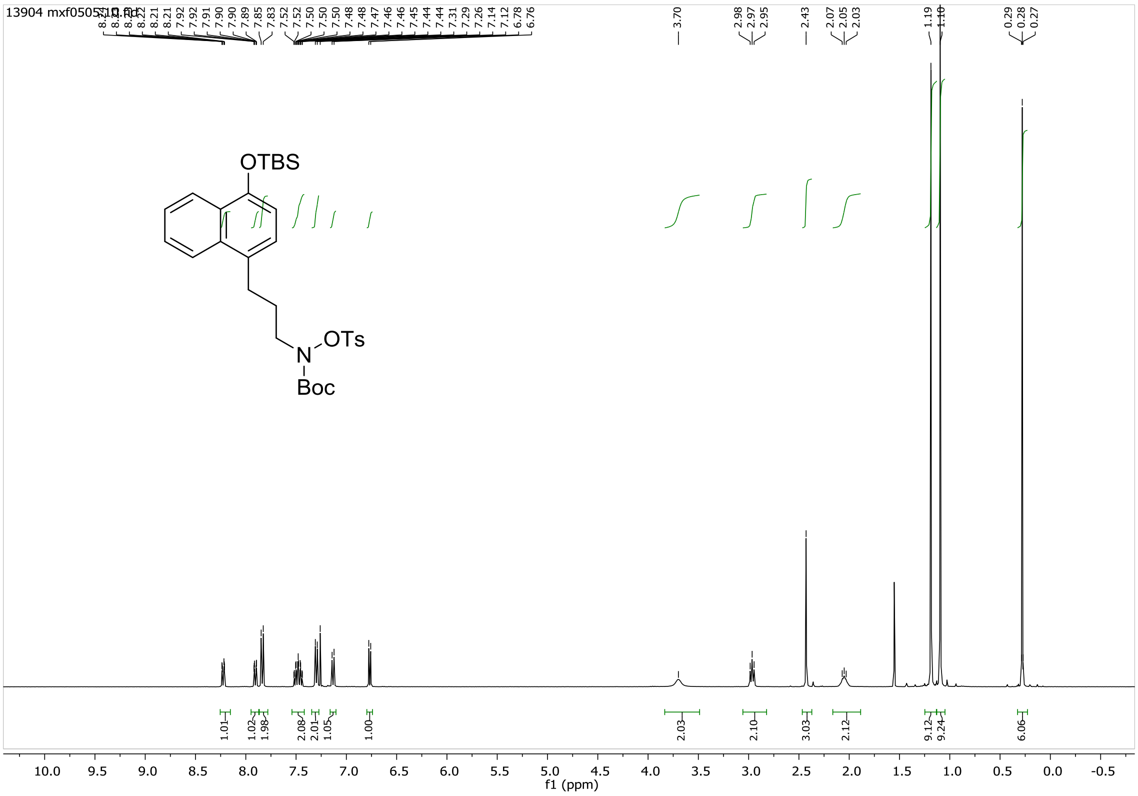

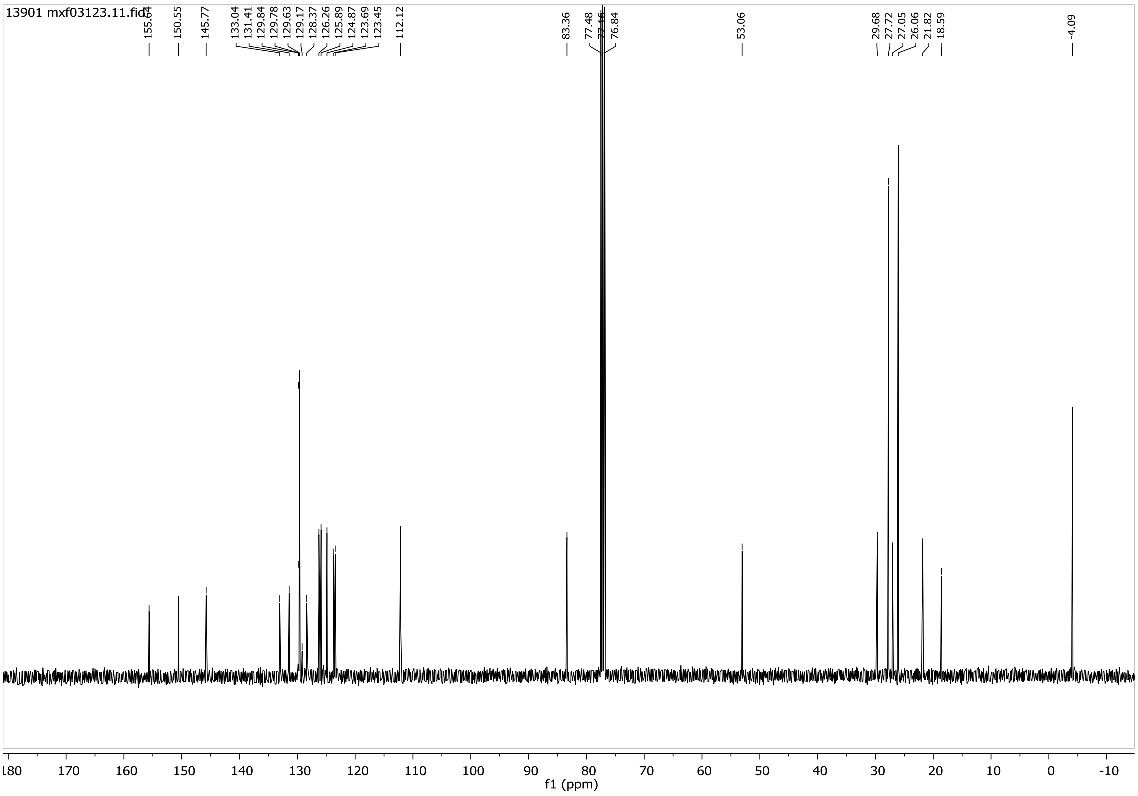

tert-Butyl (3-(4-hydroxynaphthalen-1-yl)propyl)(tosyloxy)carbamate (5e)

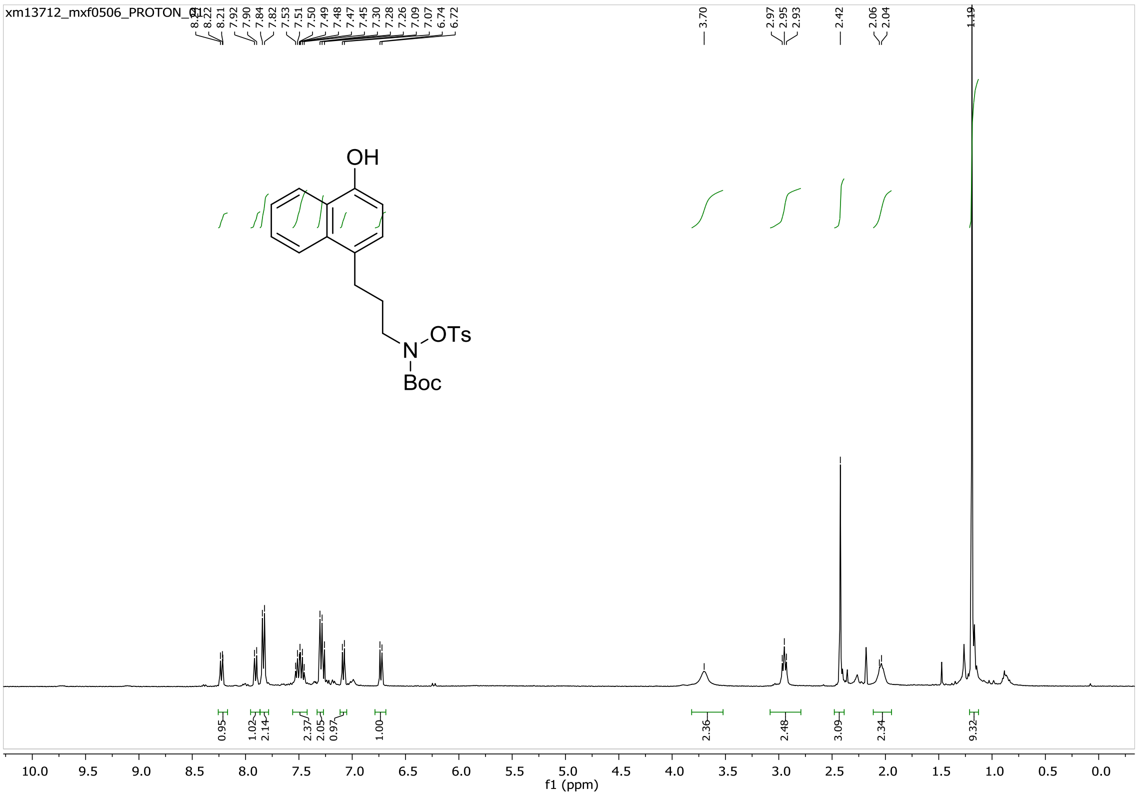

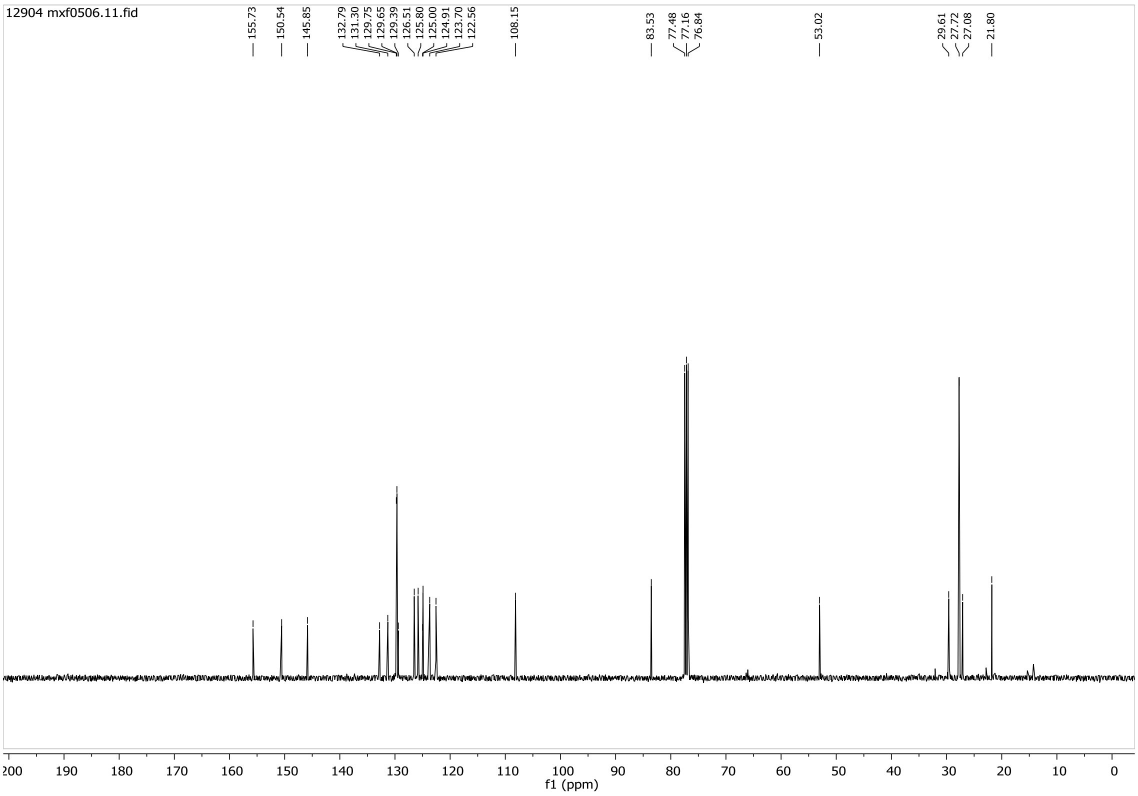

Ethyl 3-(2-((*tert*-butyldimethylsilyl)oxy)-4-methoxyphenyl)propanoate

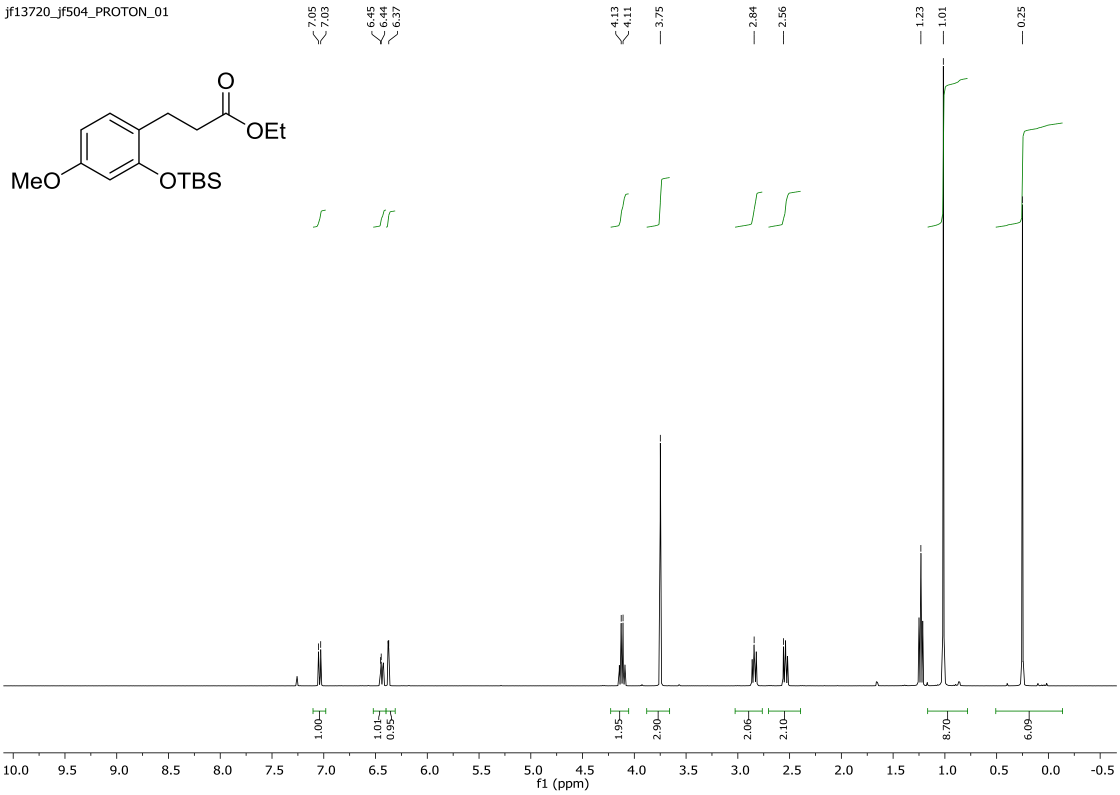

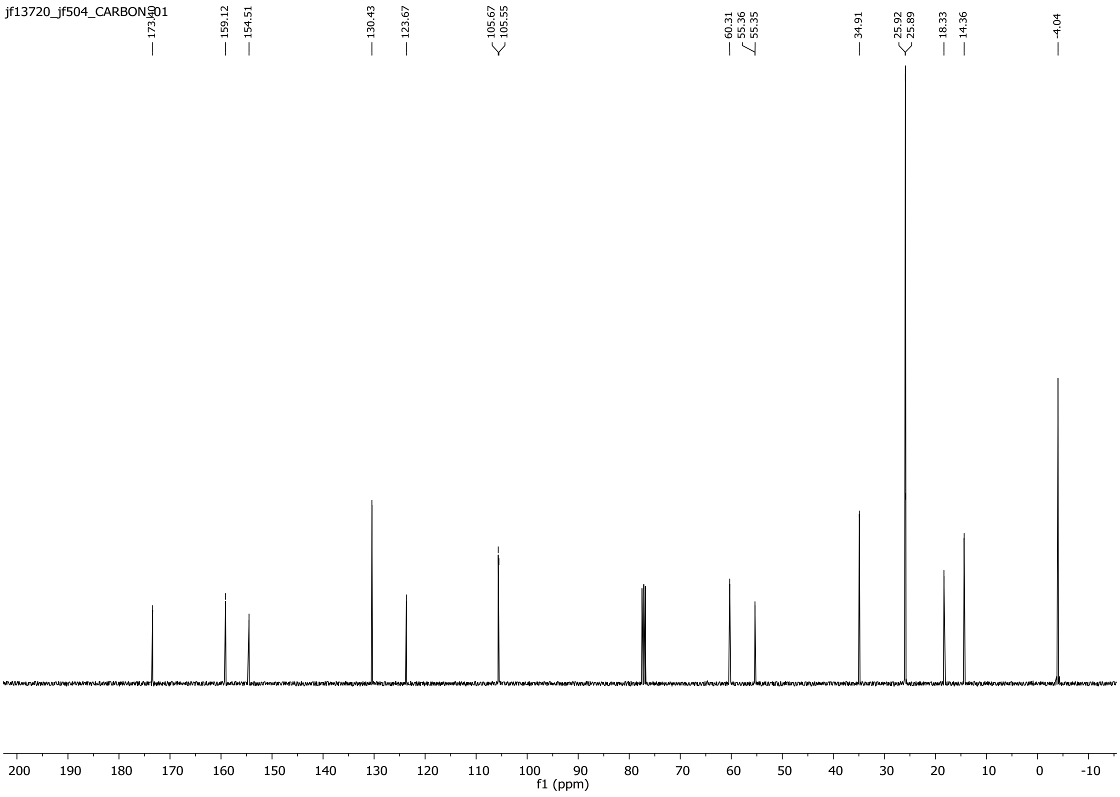

***tert*-Butyl (3-(2-((*tert*-butyldimethylsilyl)oxy)-4-methoxyphenyl)propyl)(tosyloxy) carbamate**

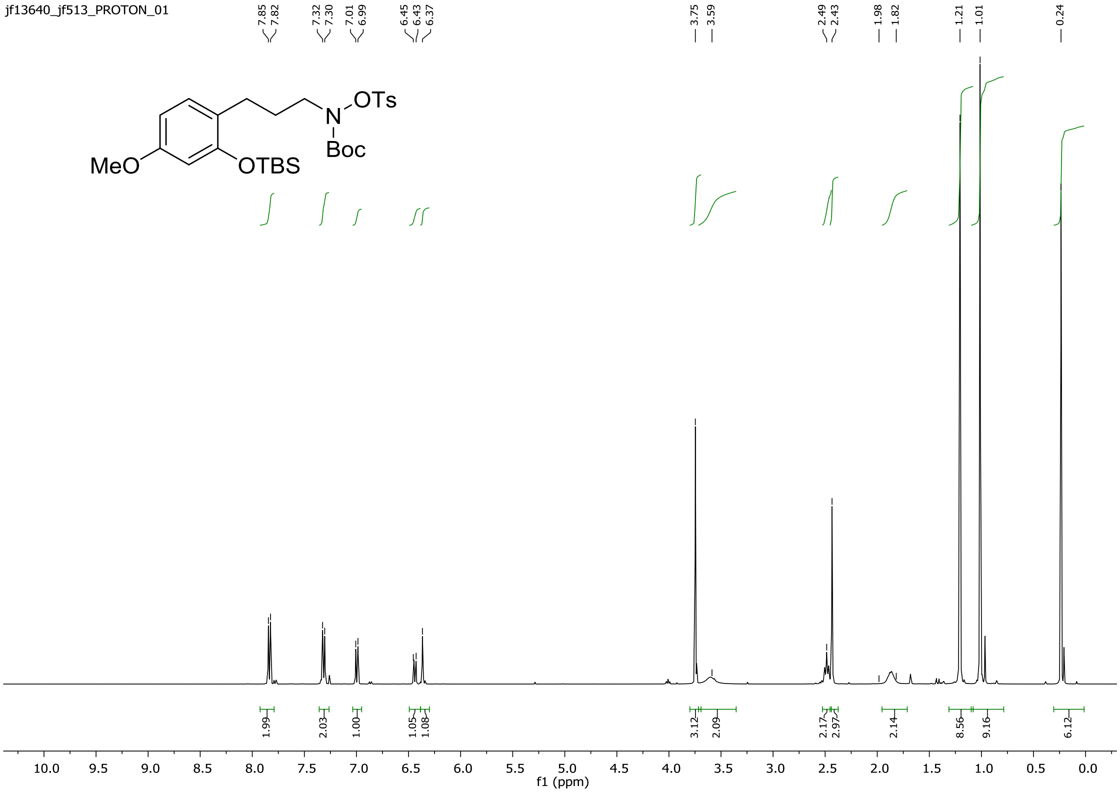

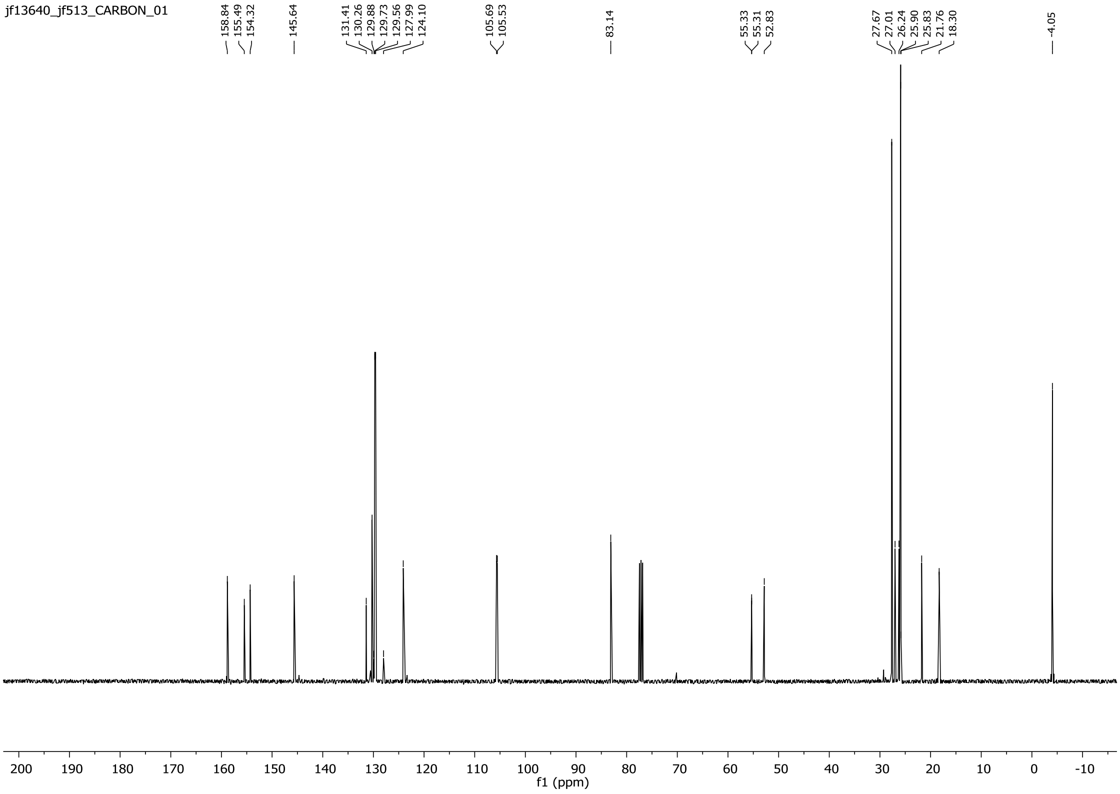

tert-Butyl (3-(2-hydroxy-4-methoxyphenyl)propyl)(tosyloxy)carbamate (5f)

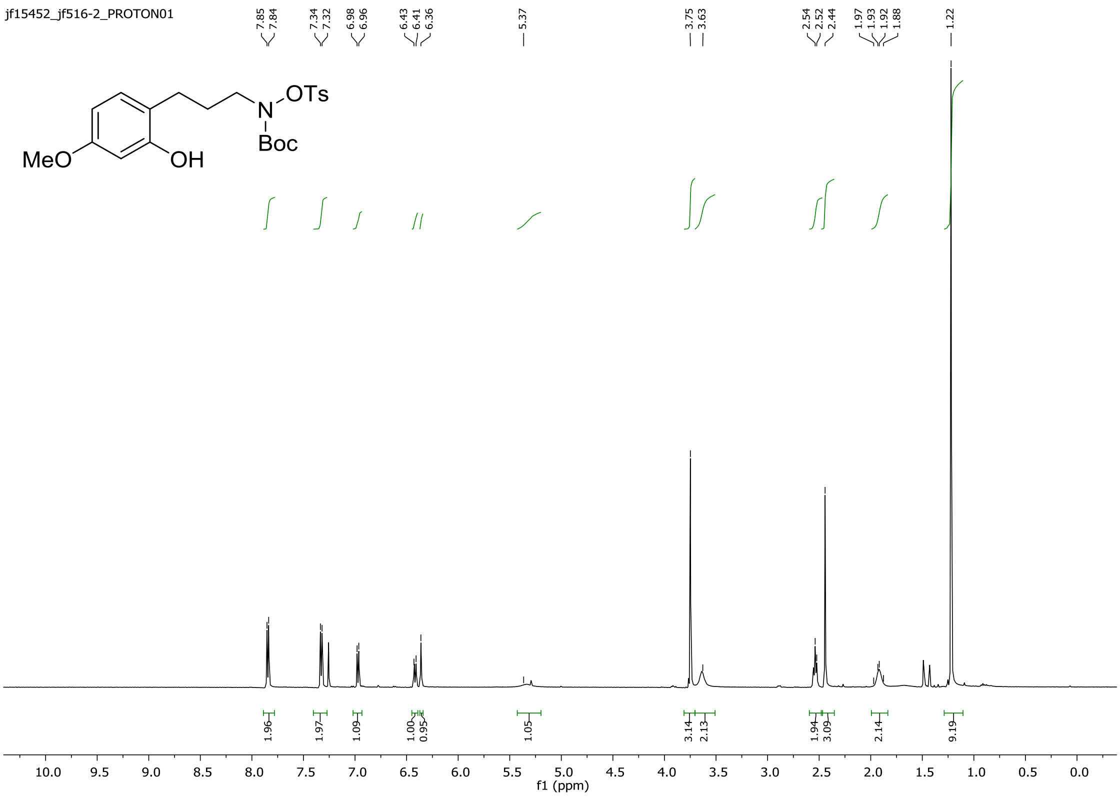

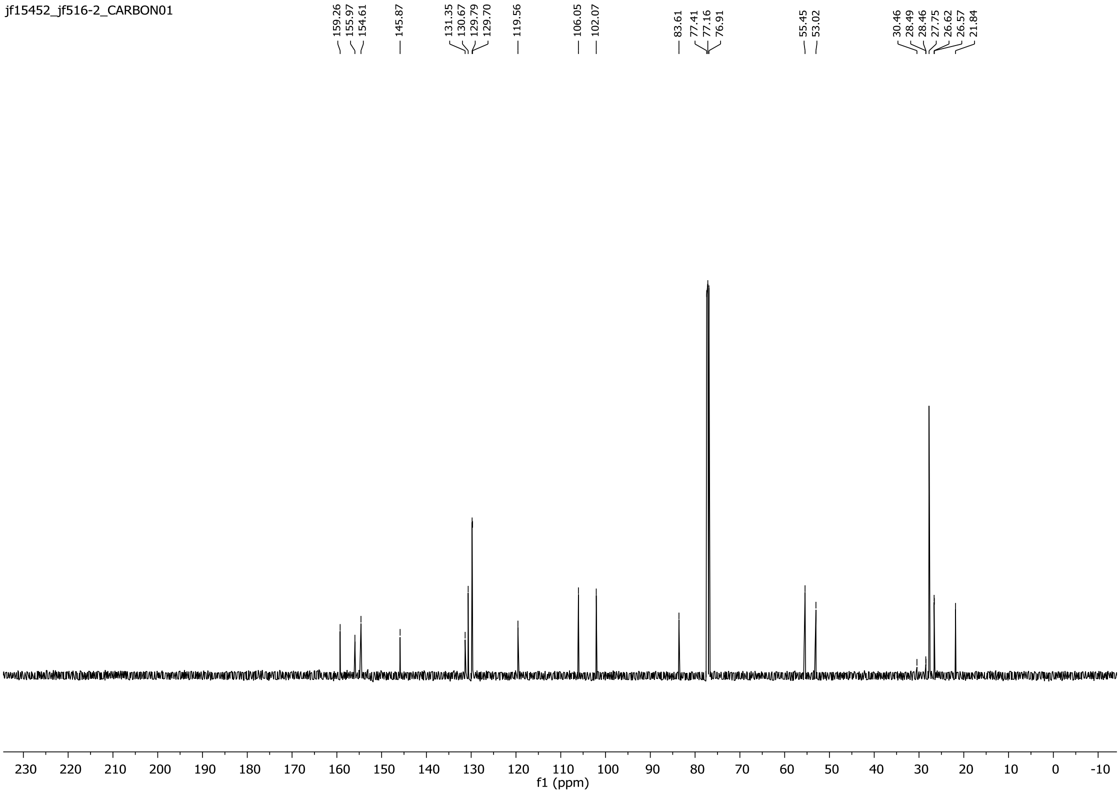

3-(2-((*tert*-Butyldimethylsilyl)oxy)naphthalen-1-yl)propanoic acid

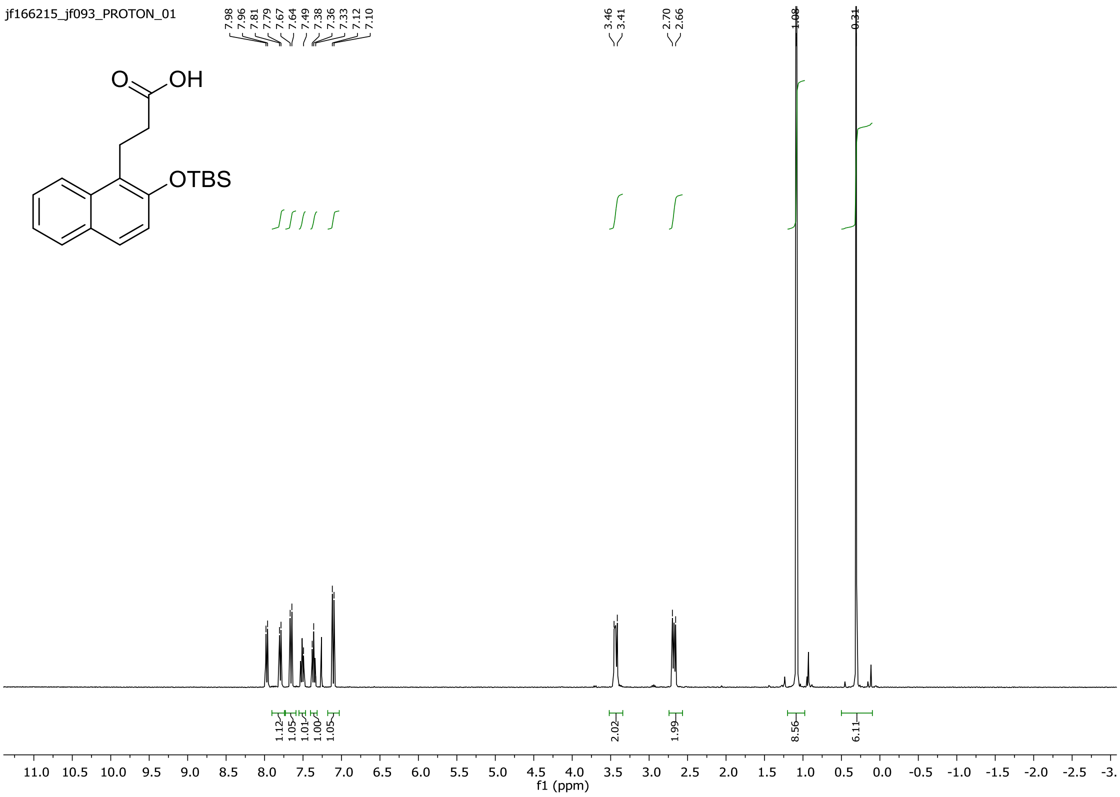

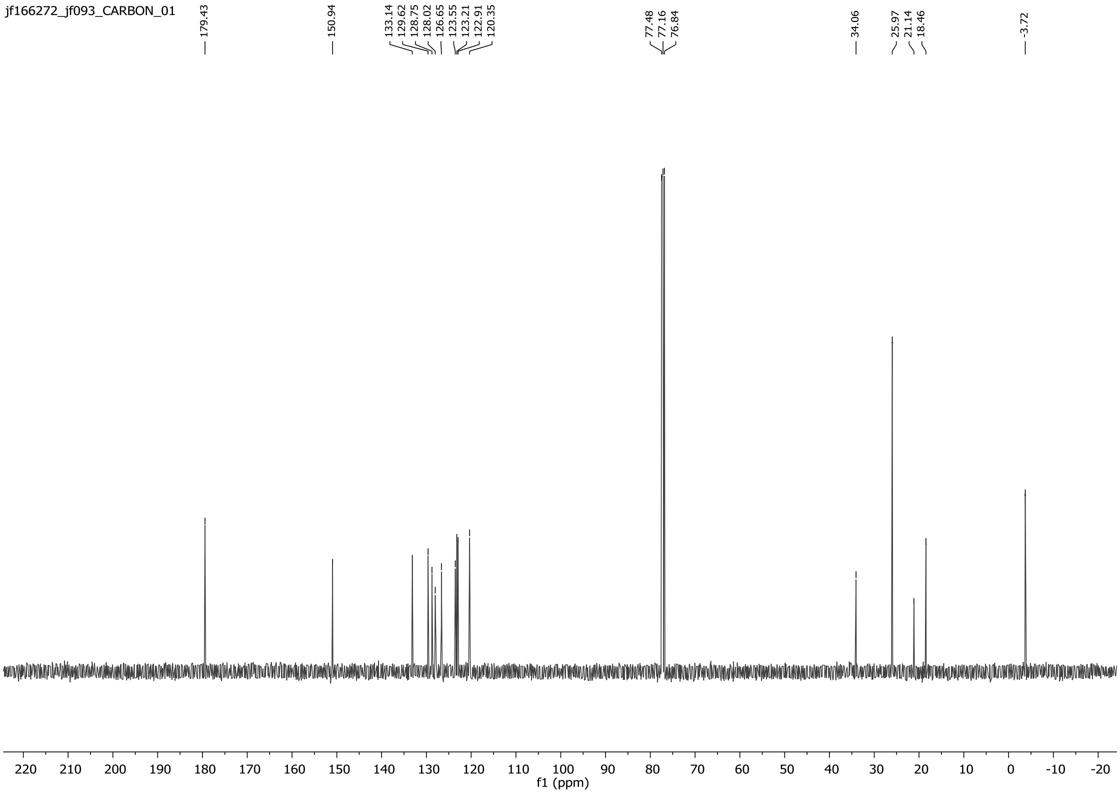

tert-Butyl (3-(2-((tert-butyldimethylsilyl)oxy)naphthalen-1-yl)propyl)(tosyloxy) carbamate

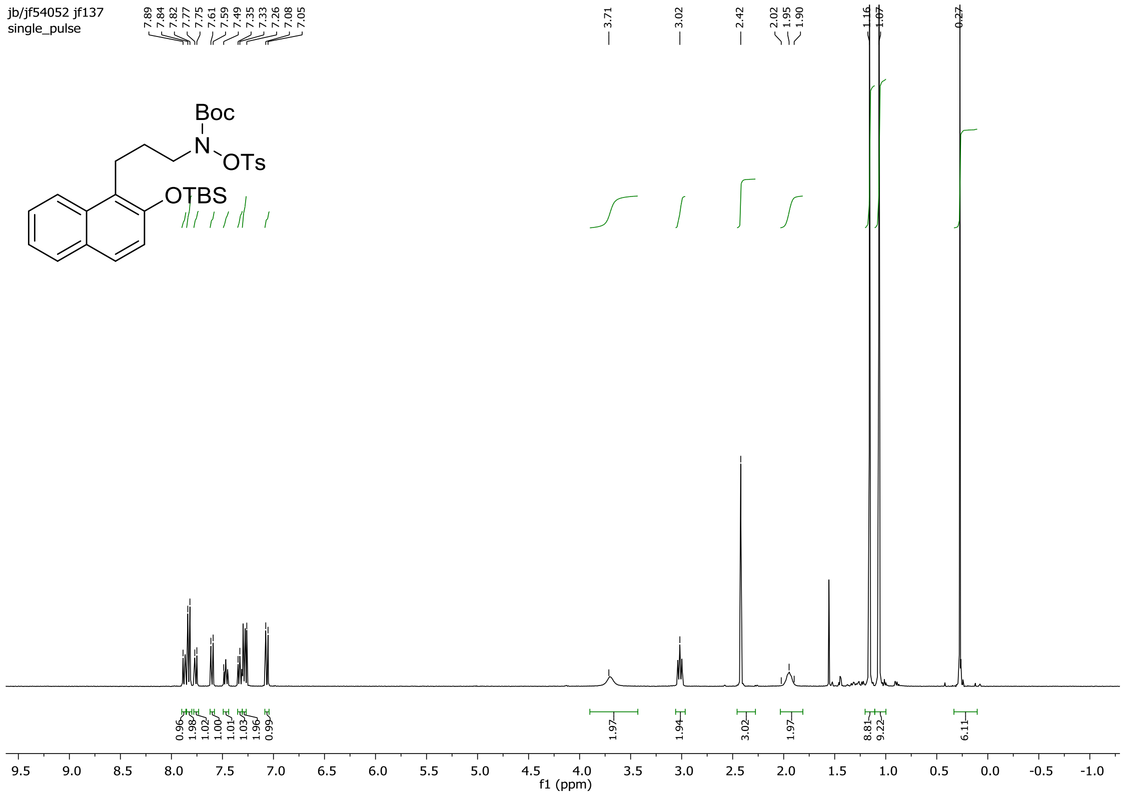

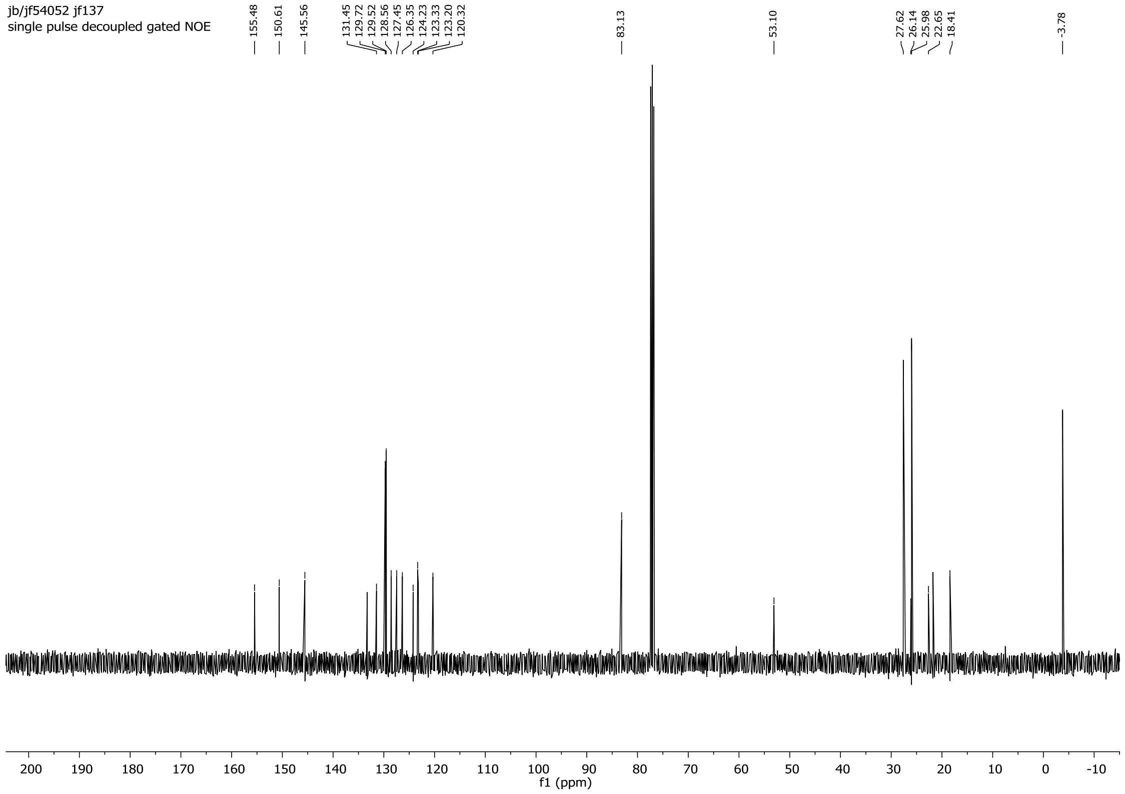

***tert*-Butyl (3-(2-hydroxynaphthalen-1-yl)propyl)(tosyloxy)carbamate (5g)**

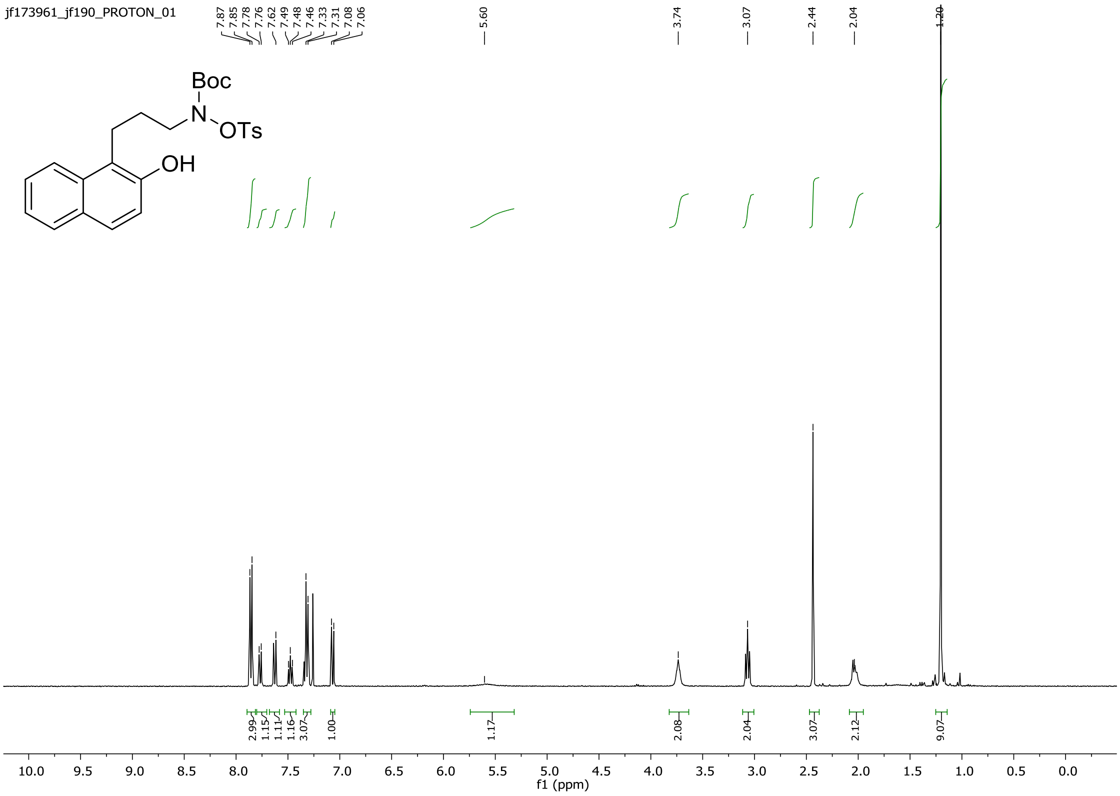

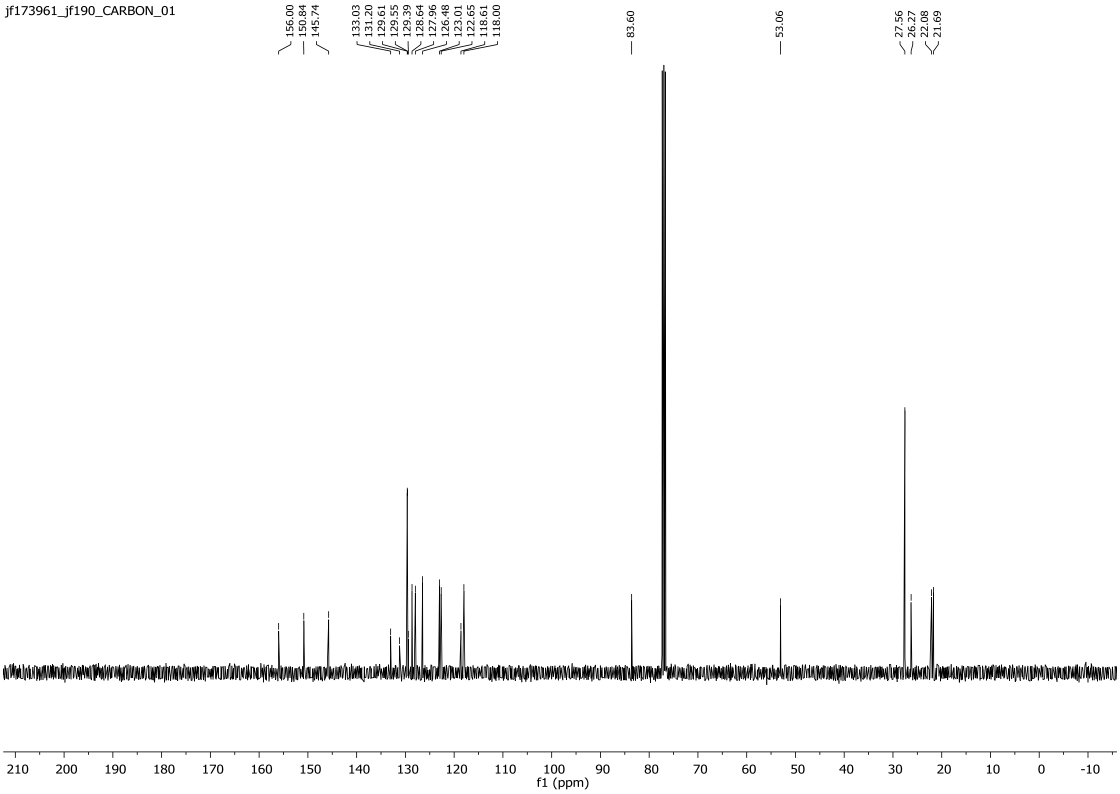

tert-Butyldimethyl((1-(1-phenylallyl)naphthalen-2-yl)oxy)silane

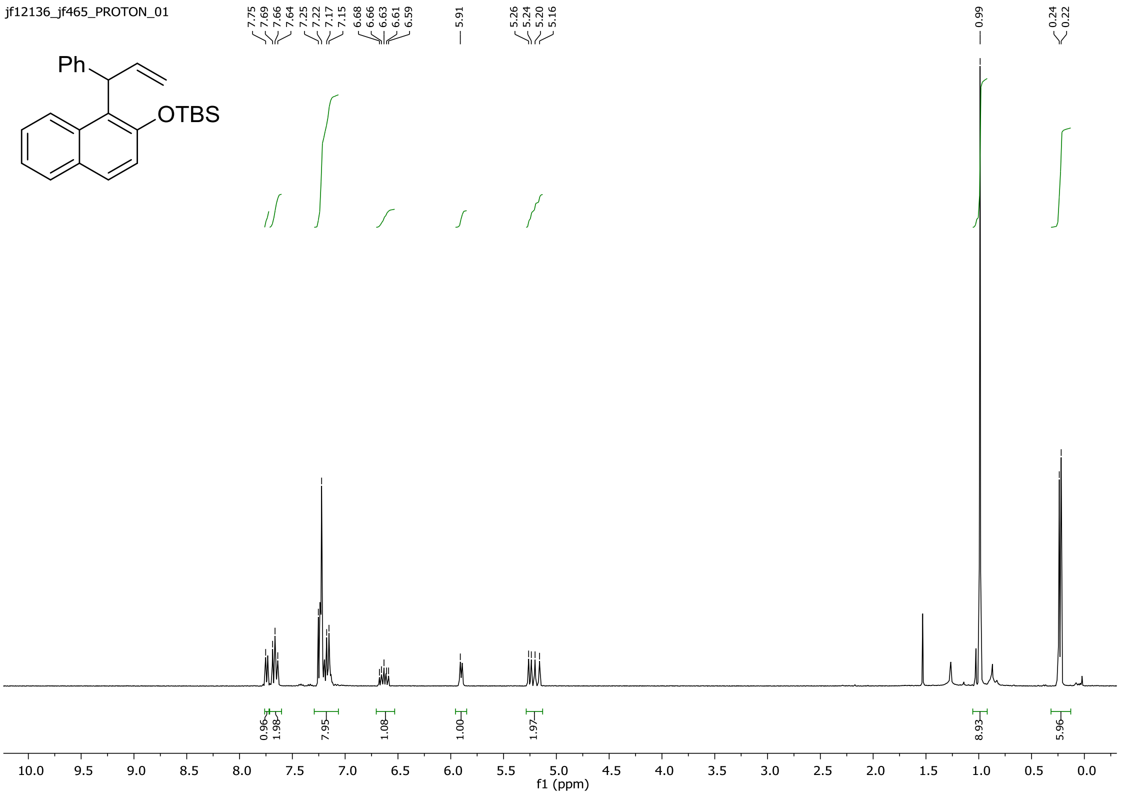

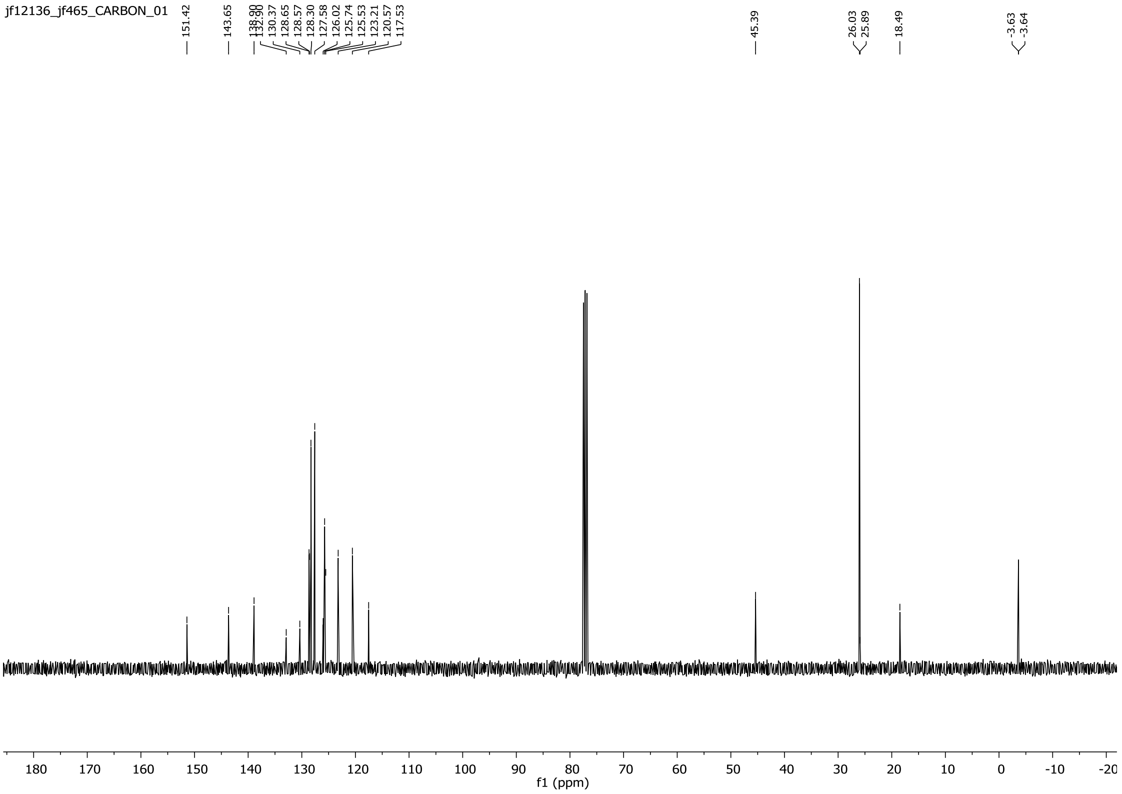

3-(2-((*tert*-Butyldimethylsilyl)oxy)naphthalen-1-yl)-3-phenylpropan-1-ol

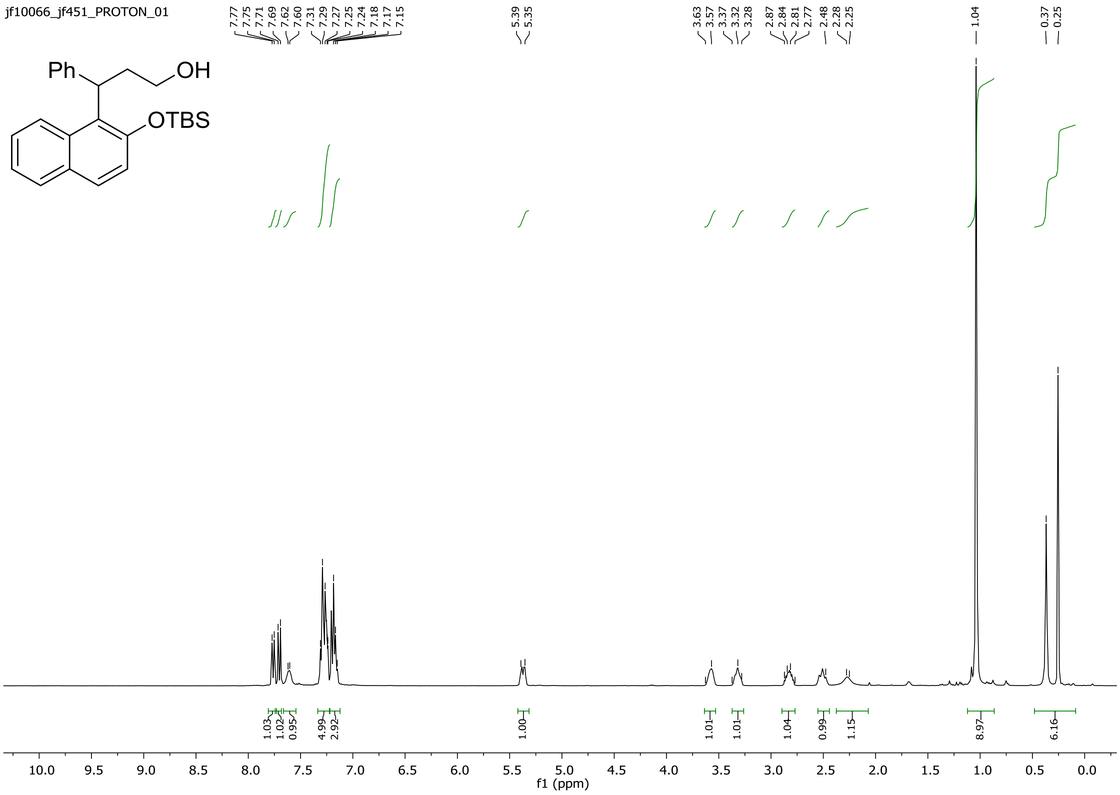

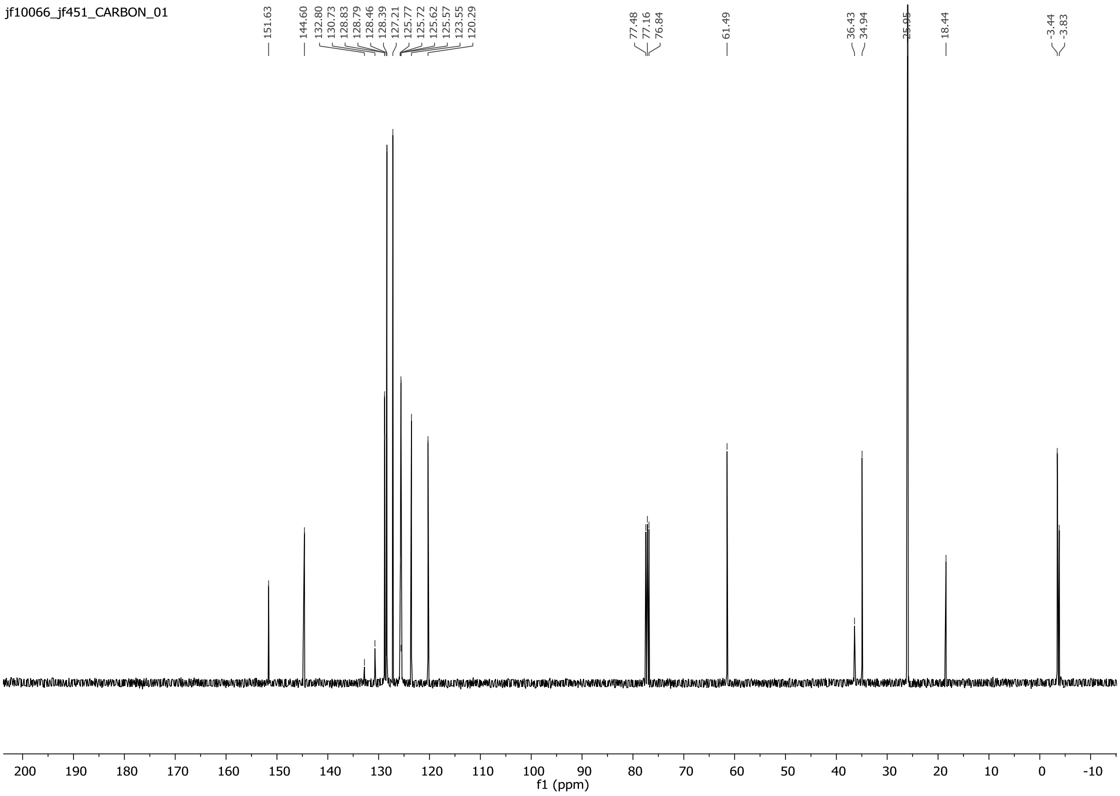

tert-Butyl (3-(2-hydroxynaphthalen-1-yl)-3-phenylpropyl)(tosyloxy)carbamate (5h)

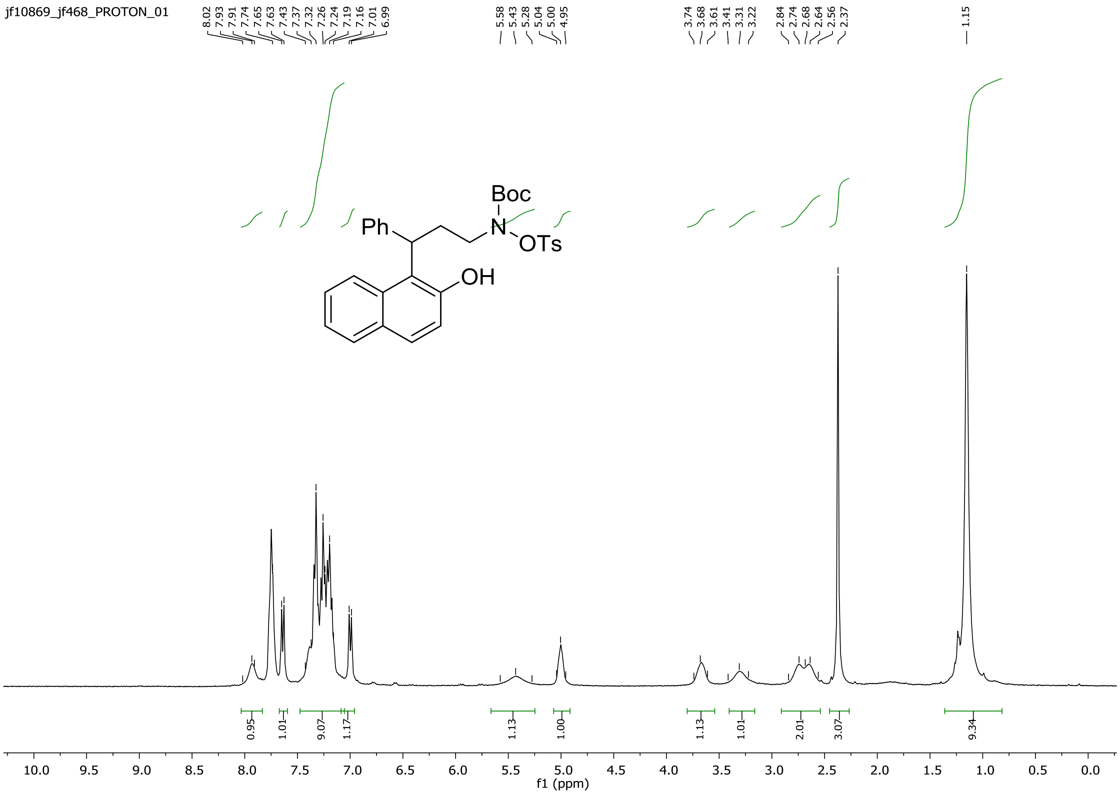

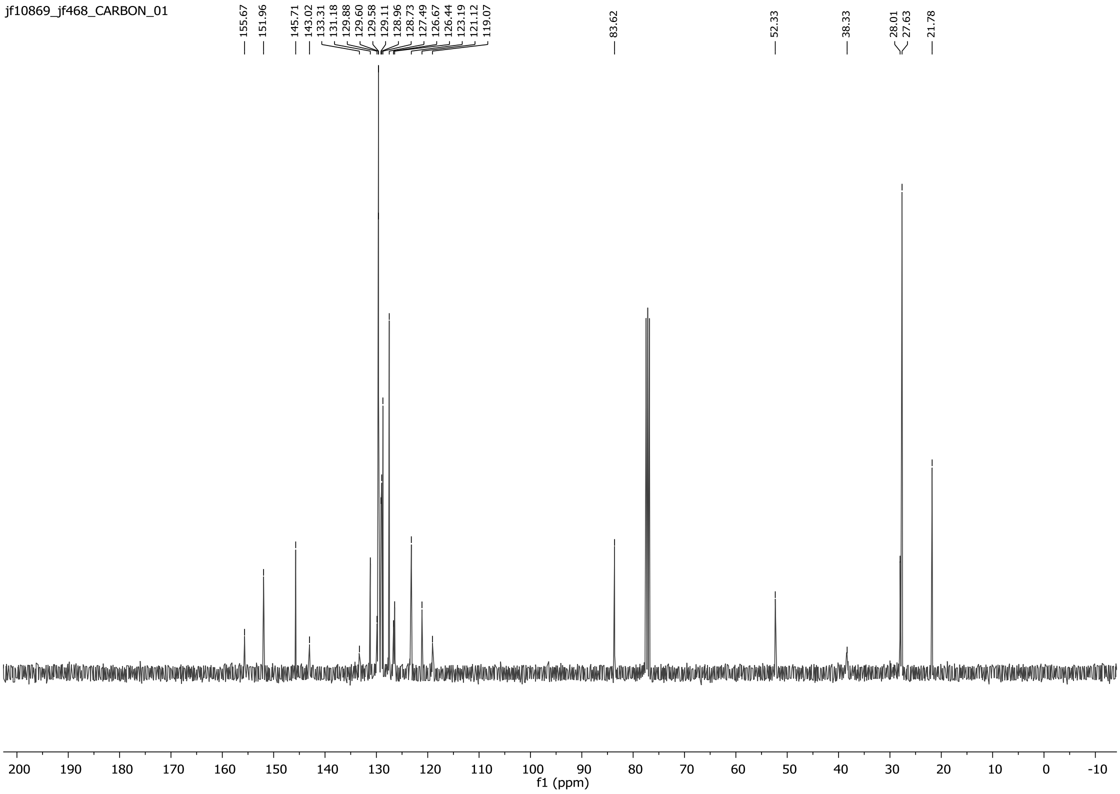

tert-Butyl 8-oxo-1-azaspiro[4.5]deca-6,9-diene-1-carboxylate (6a)

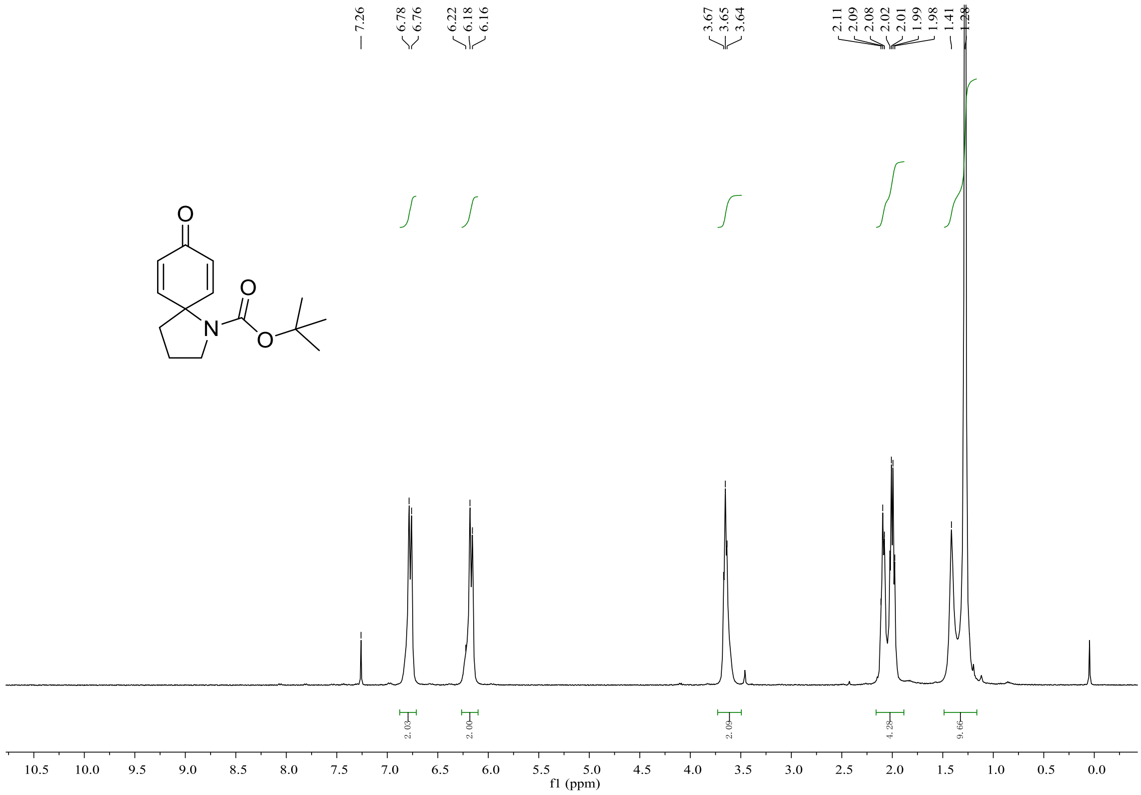

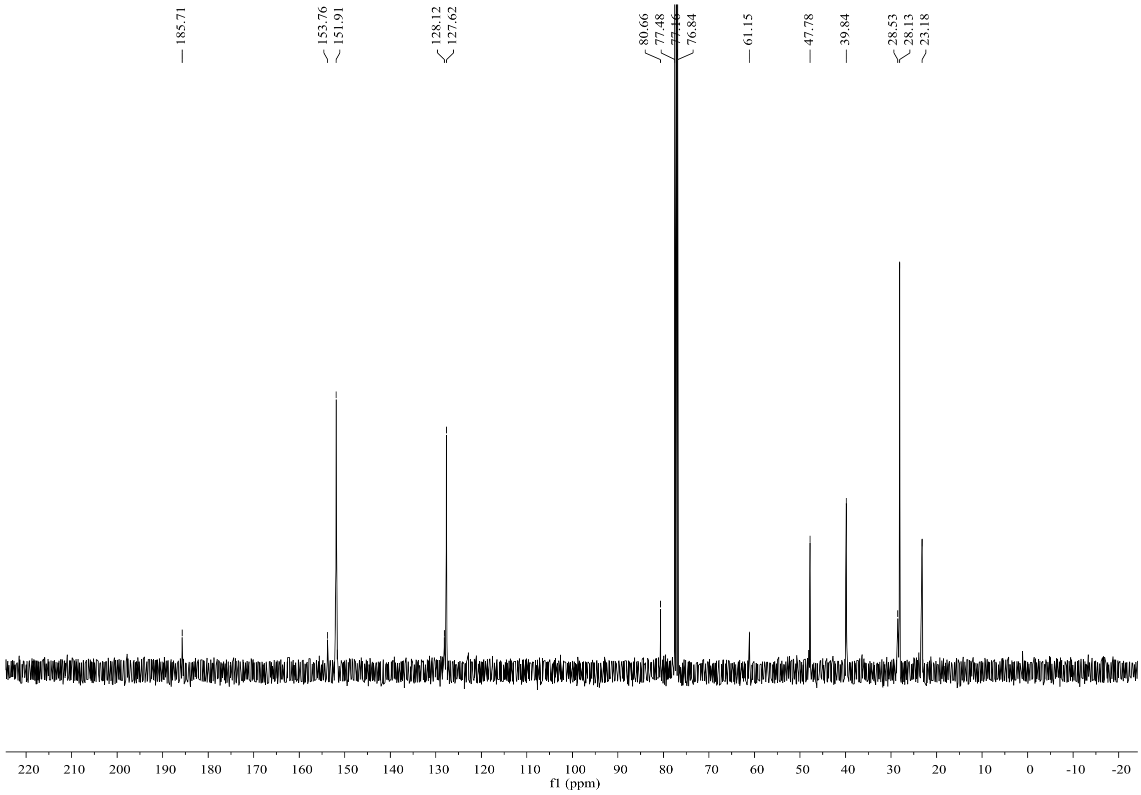

***tert*-Butyl 7,9-dimethyl-8-oxo-1-azaspiro[4.5]deca-6,9-diene-1-carboxylate (6b)**

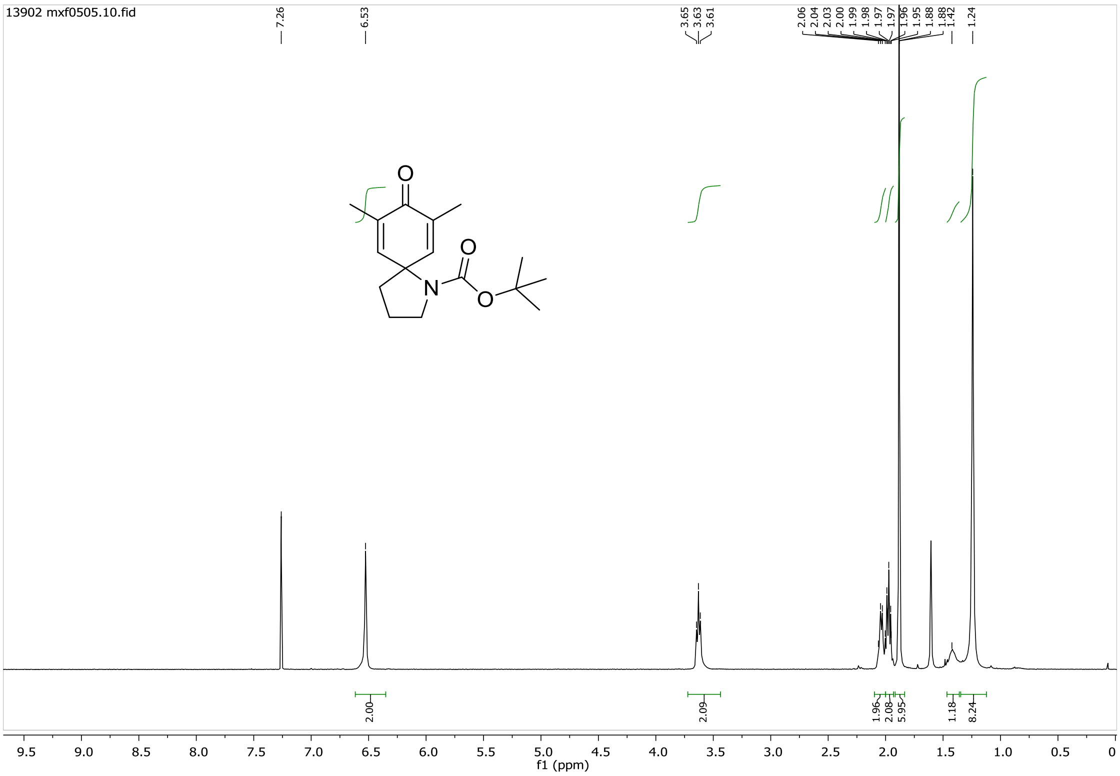

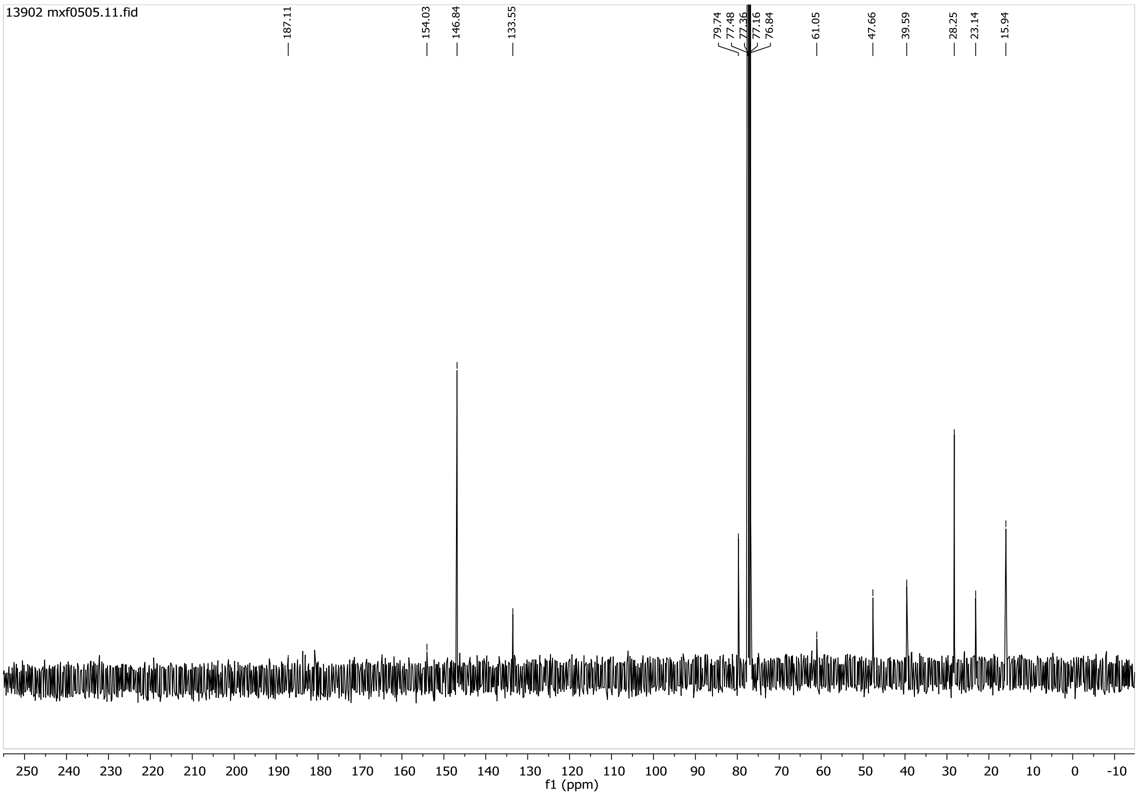

tert-Butyl 7-bromo-8-oxo-1-azaspiro[4.5]deca-6,9-diene-1-carboxylate (6c)

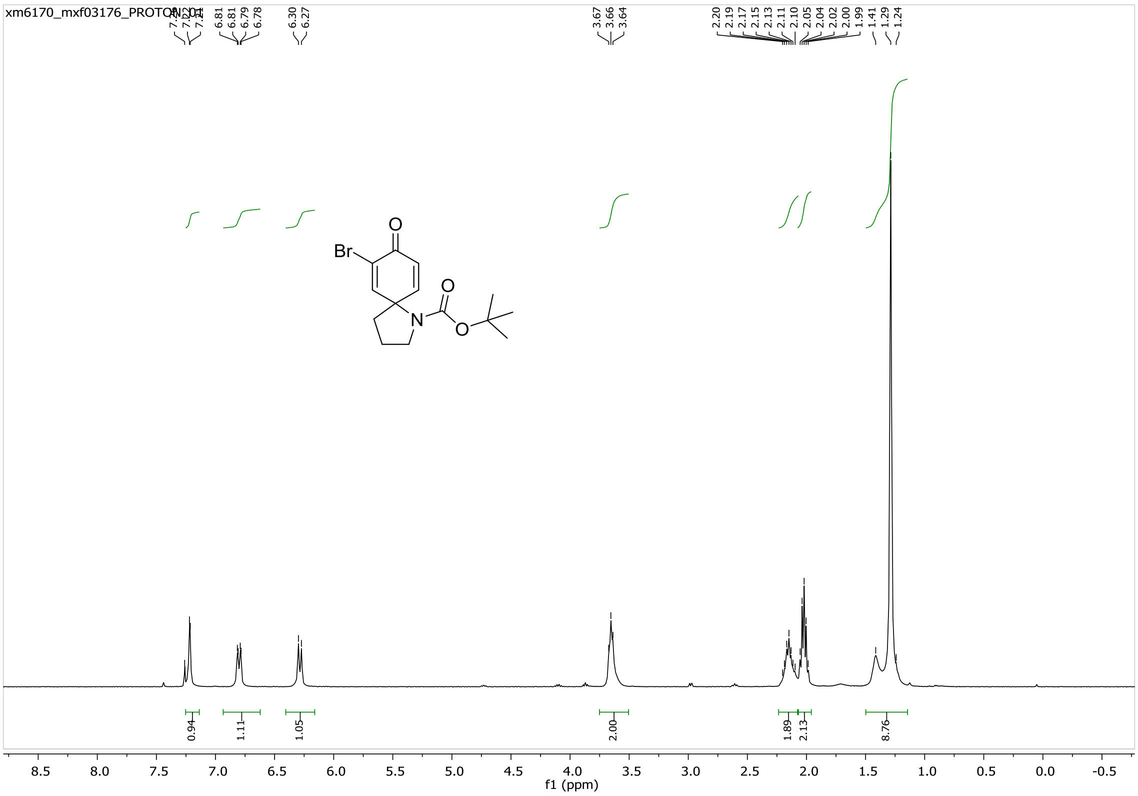

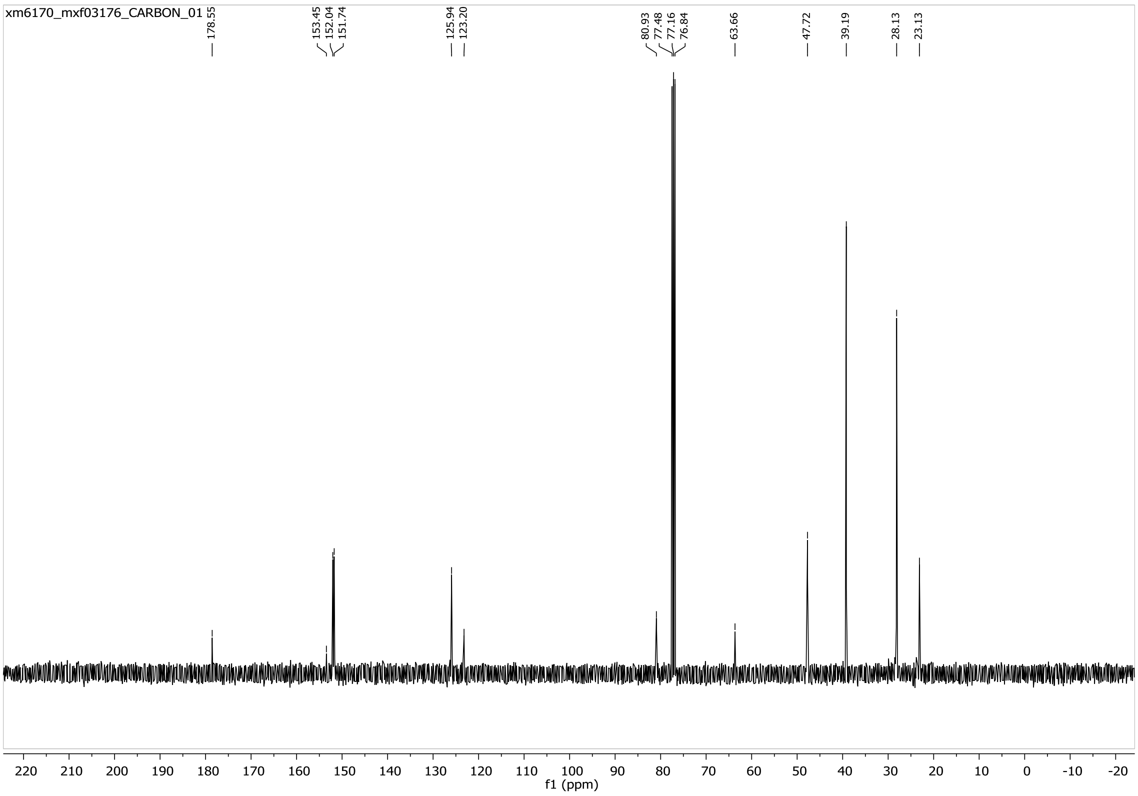

***tert*-Butyl 4-ethyl-8-oxo-1-azaspiro[4.5]deca-6,9-diene-1-carboxylate (6d)**

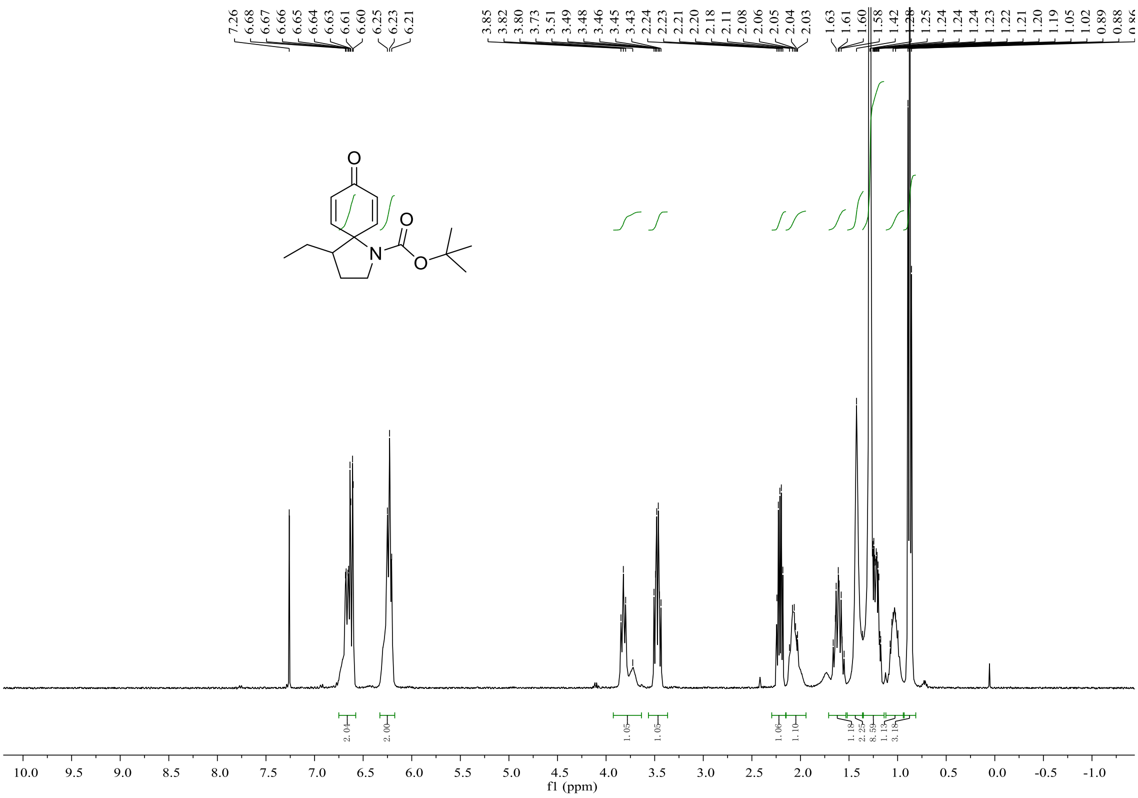

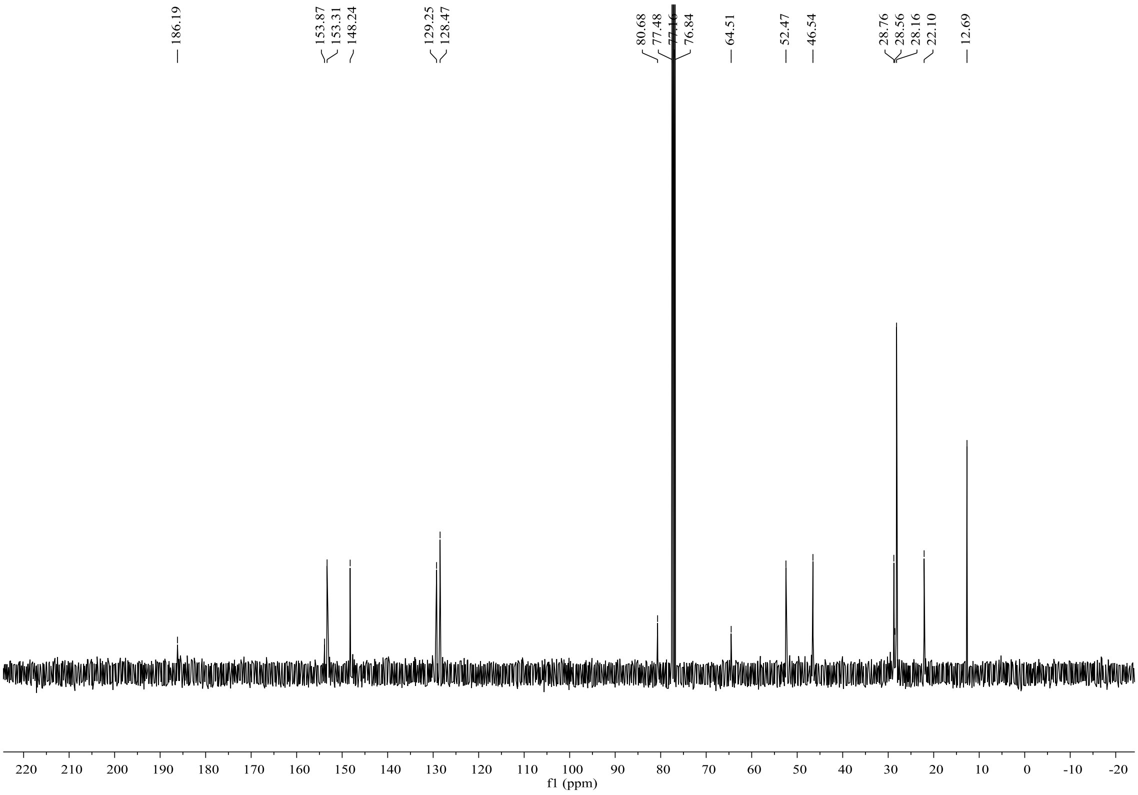

***tert*-Butyl 4-oxo-4*H*-spiro[naphthalene-1,2'-pyrrolidine]-1'-carboxylate (6e)**

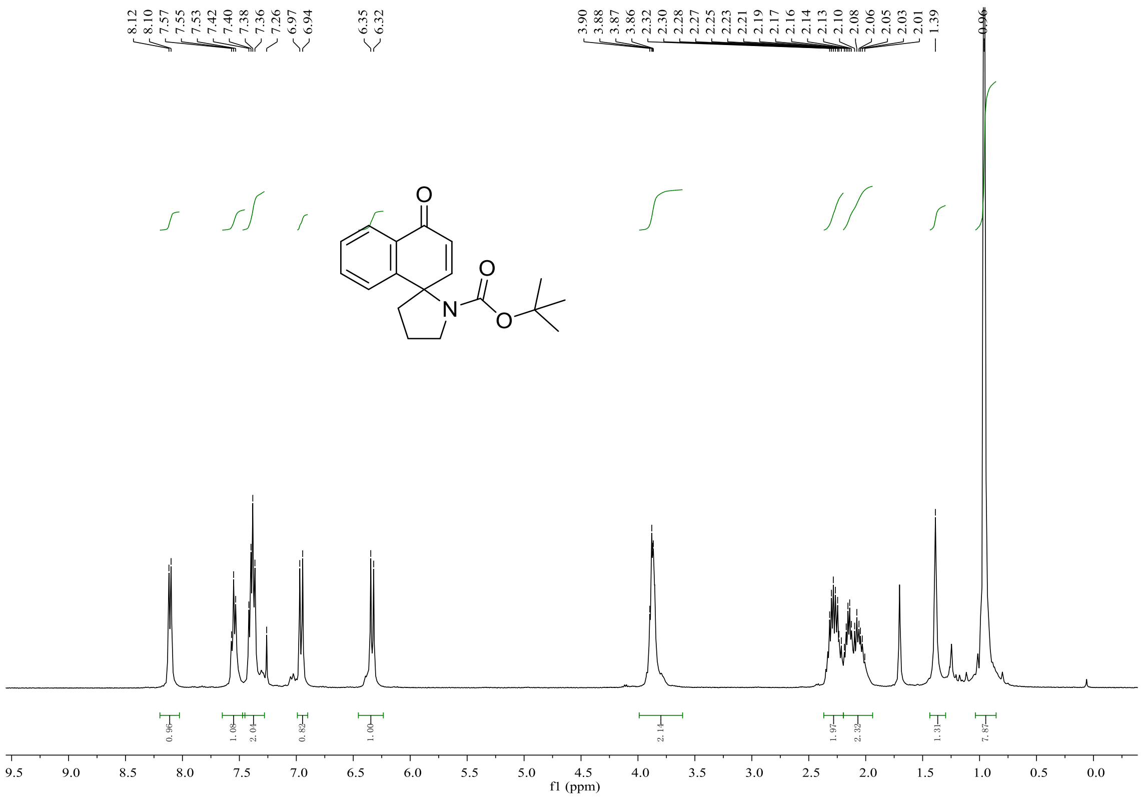

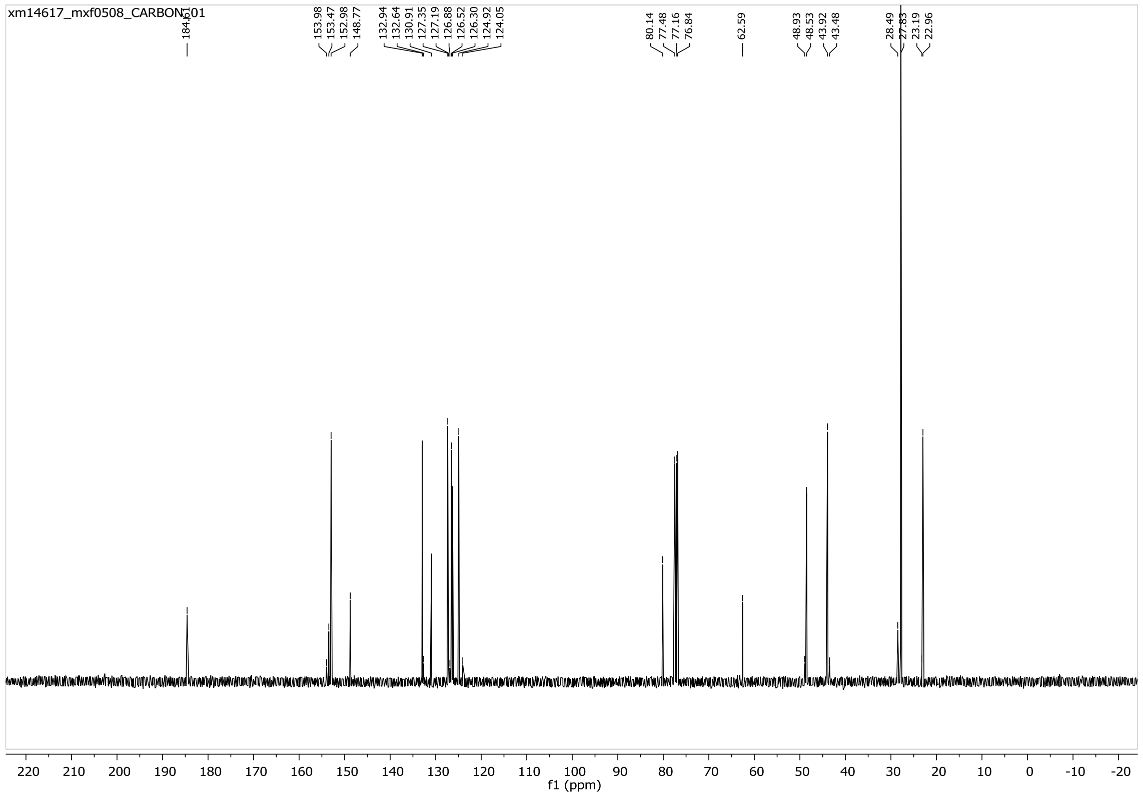

tert-Butyl 8-methoxy-10-oxo-1-azaspiro[4.5]deca-6,8-diene-1-carboxylate (6f)

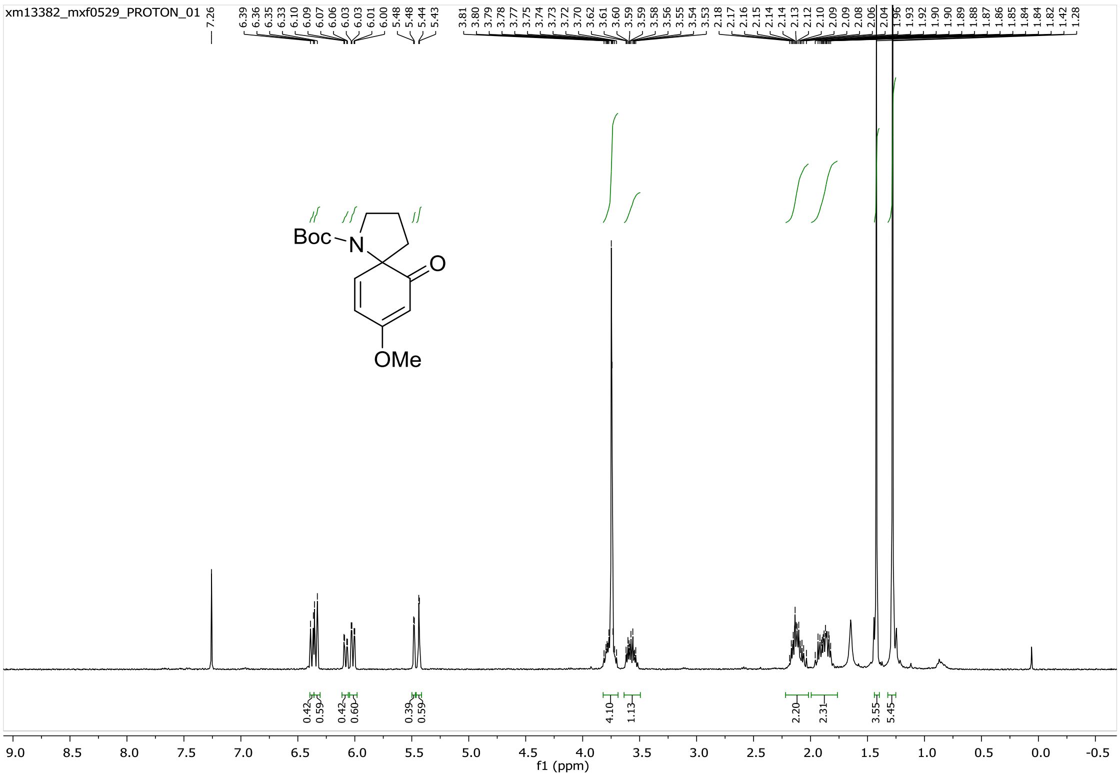

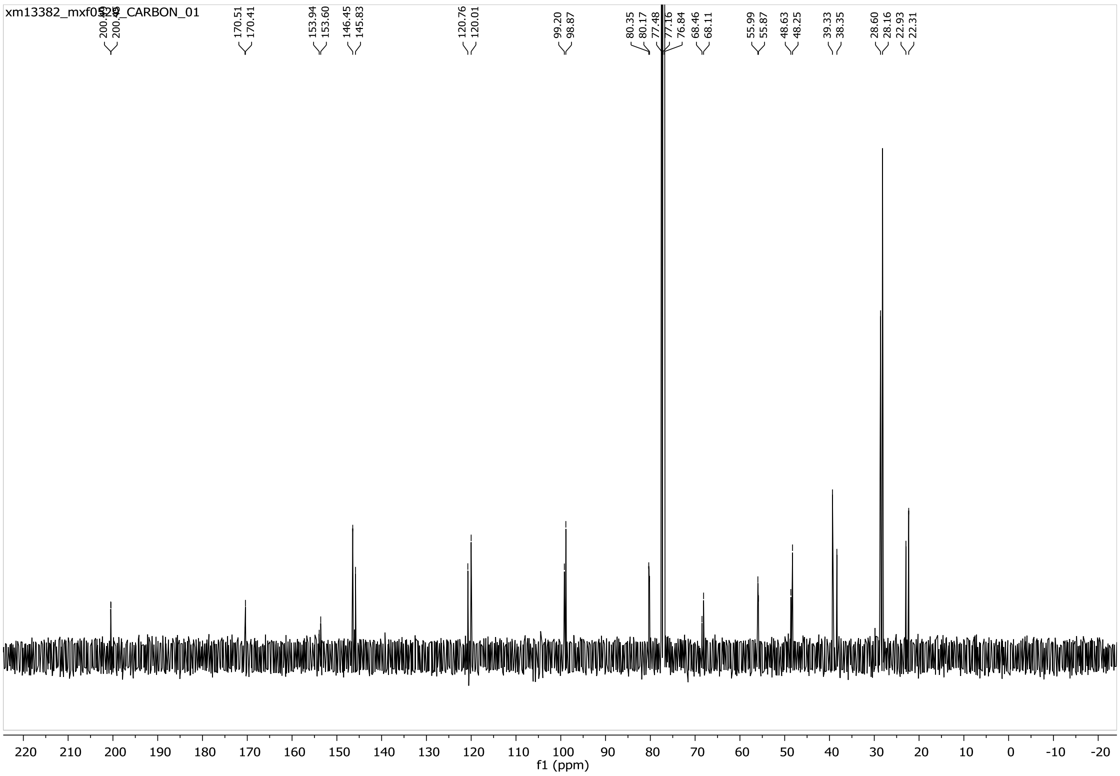

tert-Butyl 2-oxo-2H-spiro[naphthalene-1,2'-pyrrolidine]-1'-carboxylate (6g)

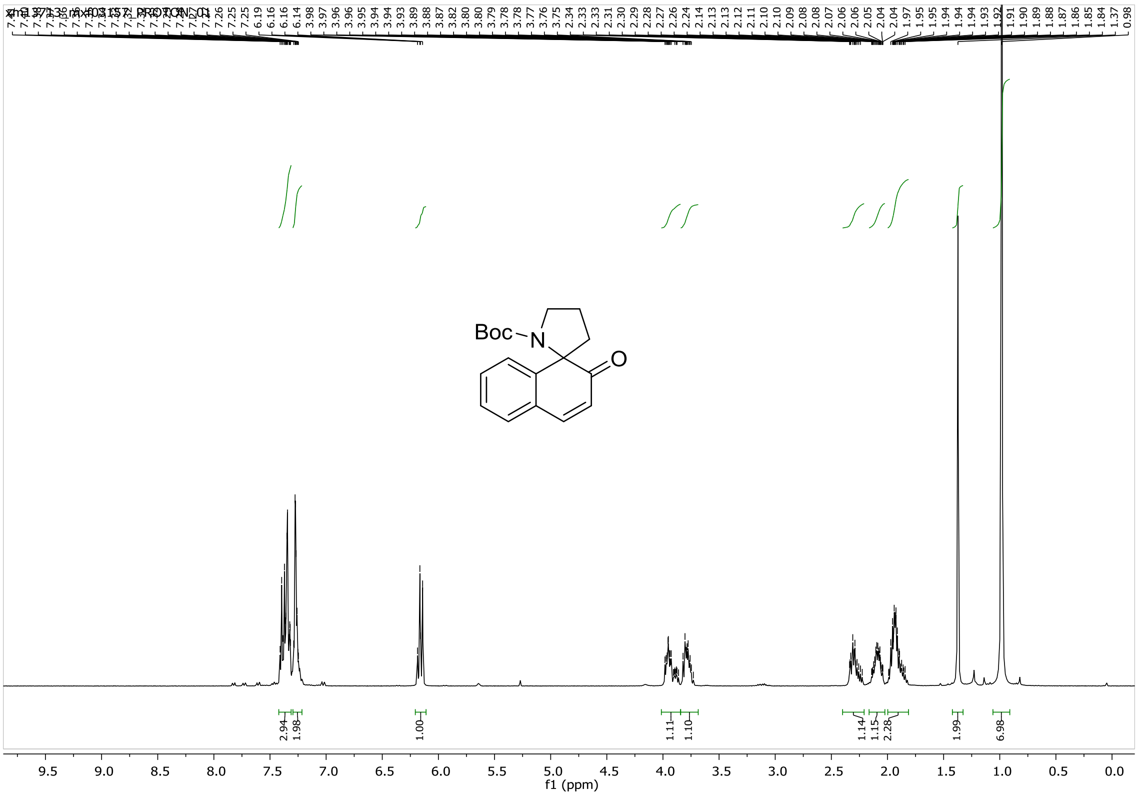

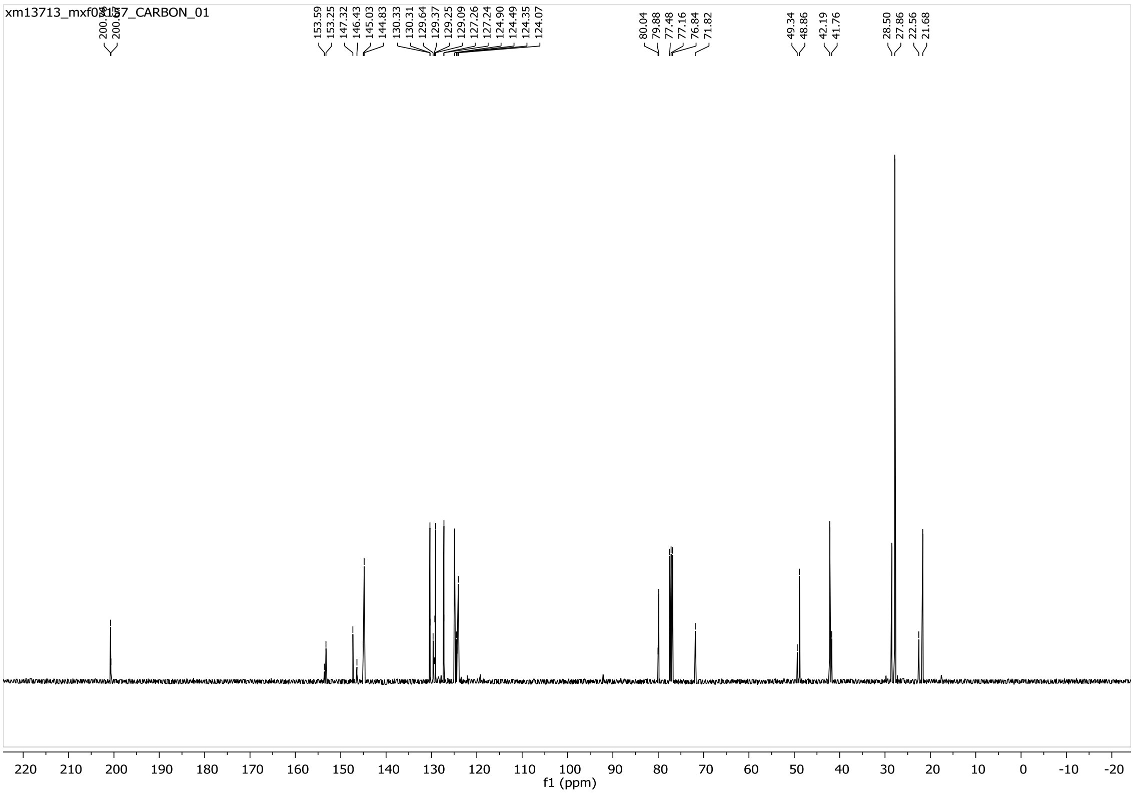

tert-Butyl (1_R_*,3'_R_*)-2-oxo-3'-phenyl-2_H_-spiro[naphthalene-1,2'-pyrrolidine]-1'-carboxylate (6h)

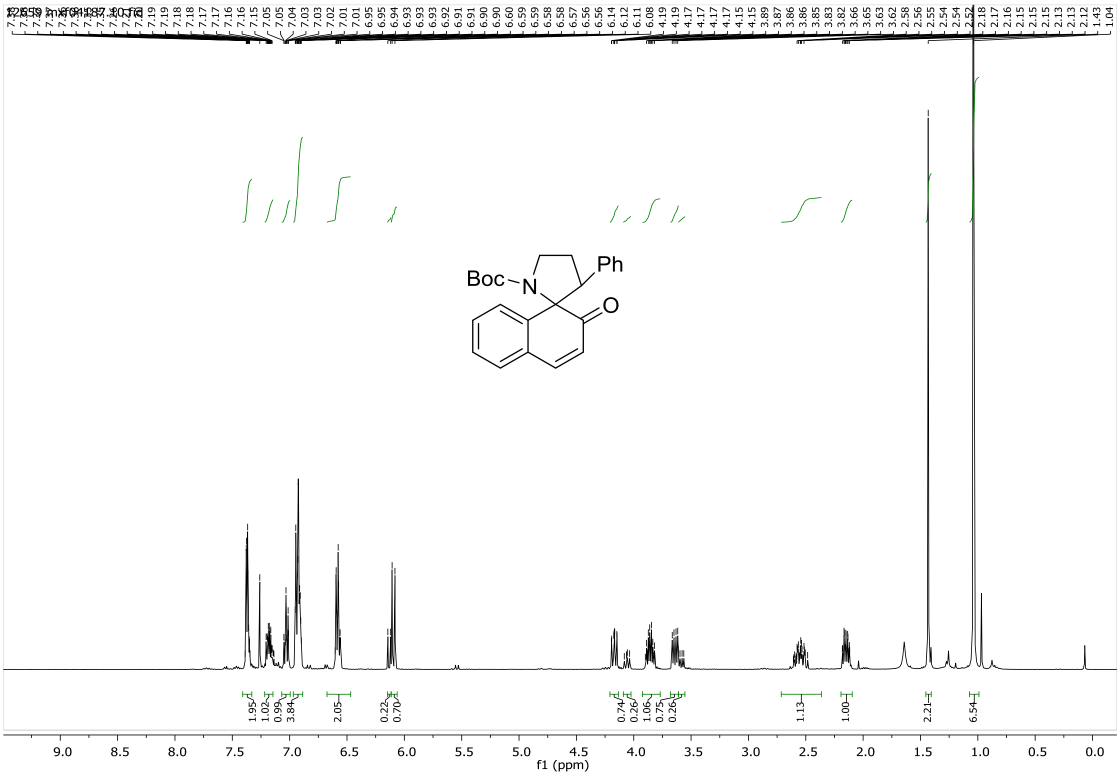

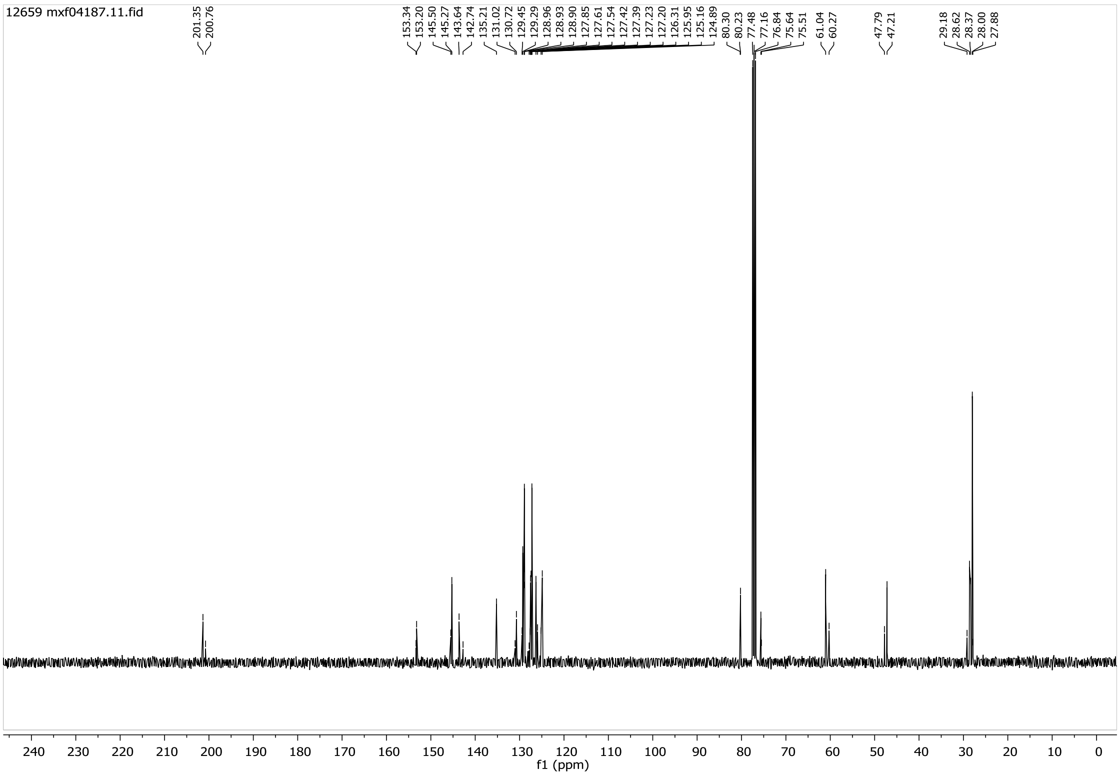

tert-Butyl (3*S**,3'*R**)-3'-phenylspiro[indoline-3,2'-pyrrolidine]-1'-carboxylate (7)

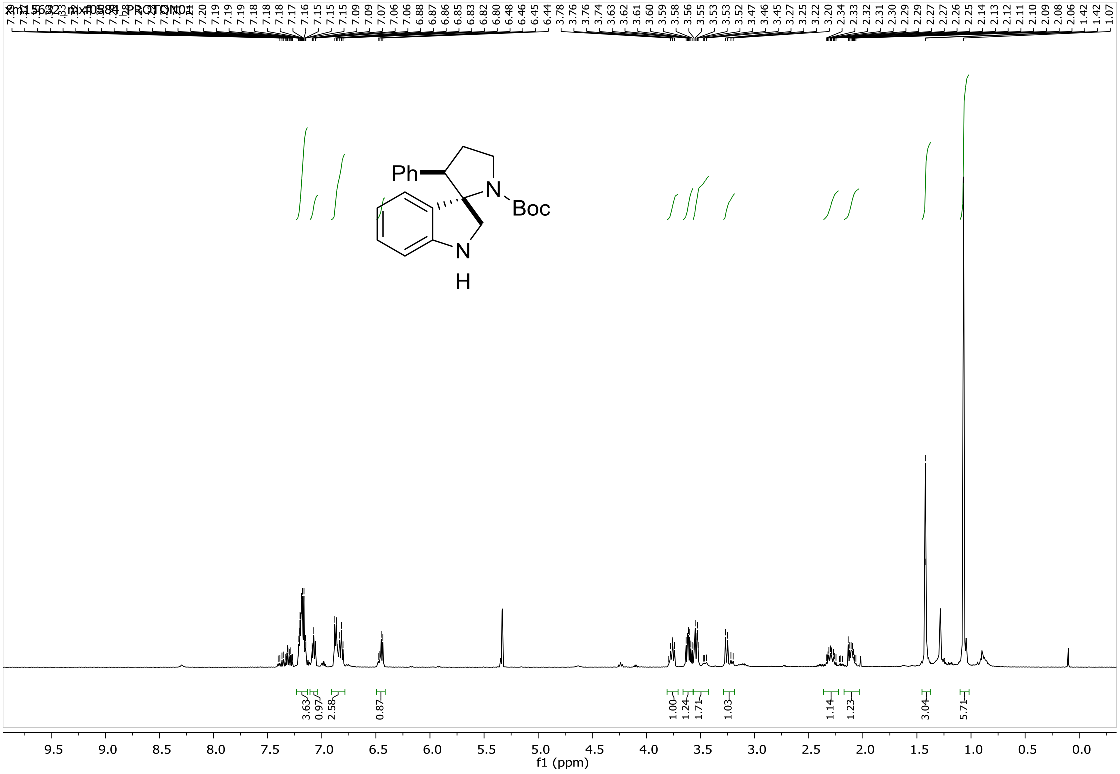

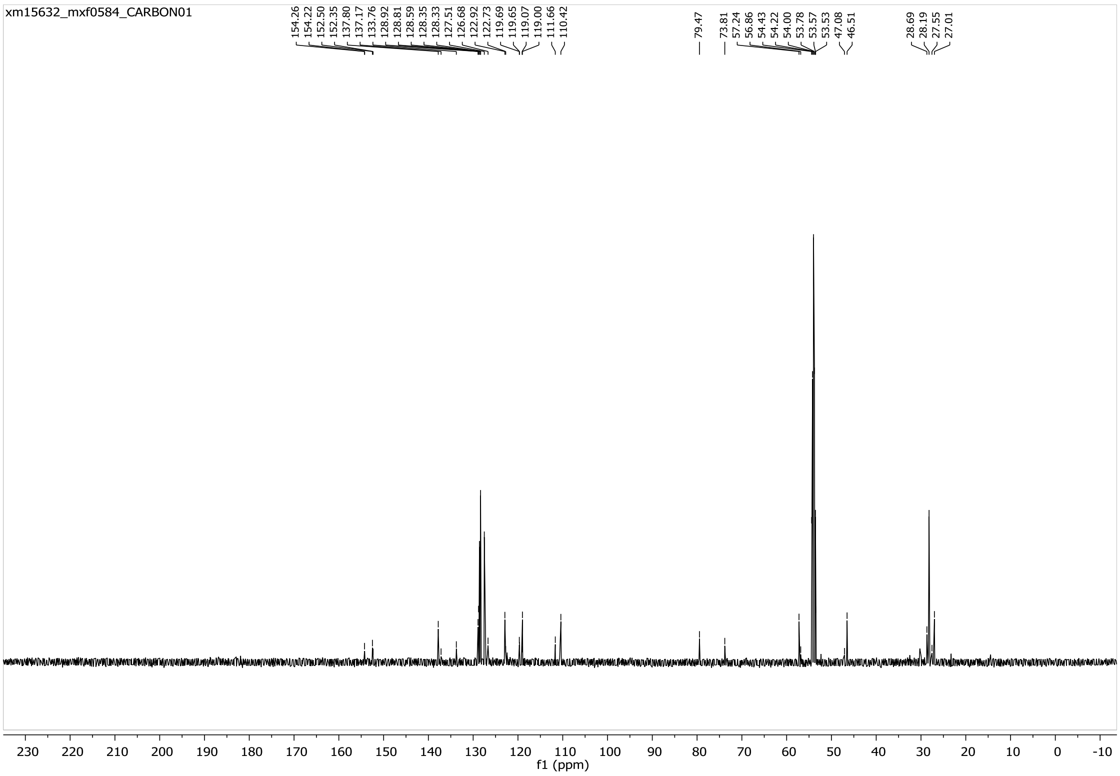

***tert*-Butyl (2*S**,3*S**,3'*R**)-2-allyl-3'-phenylspiro[indoline-3,2'-pyrrolidine]-1'-carboxylate (8)**

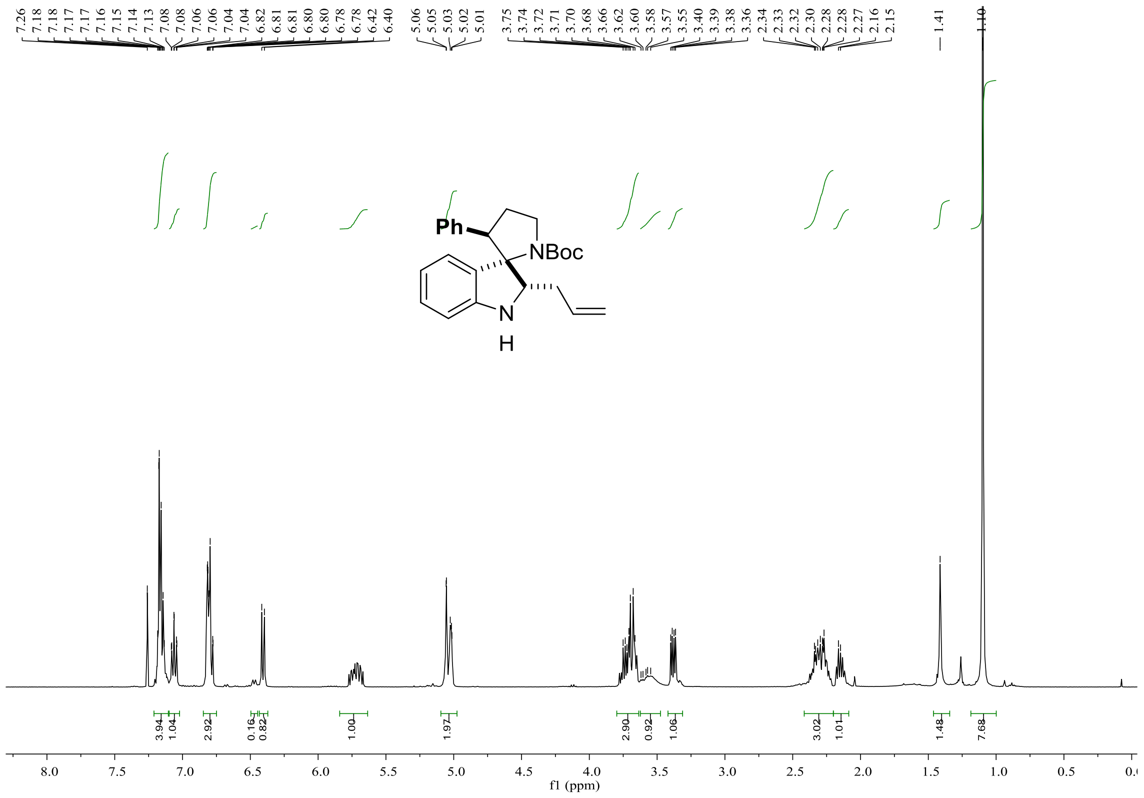

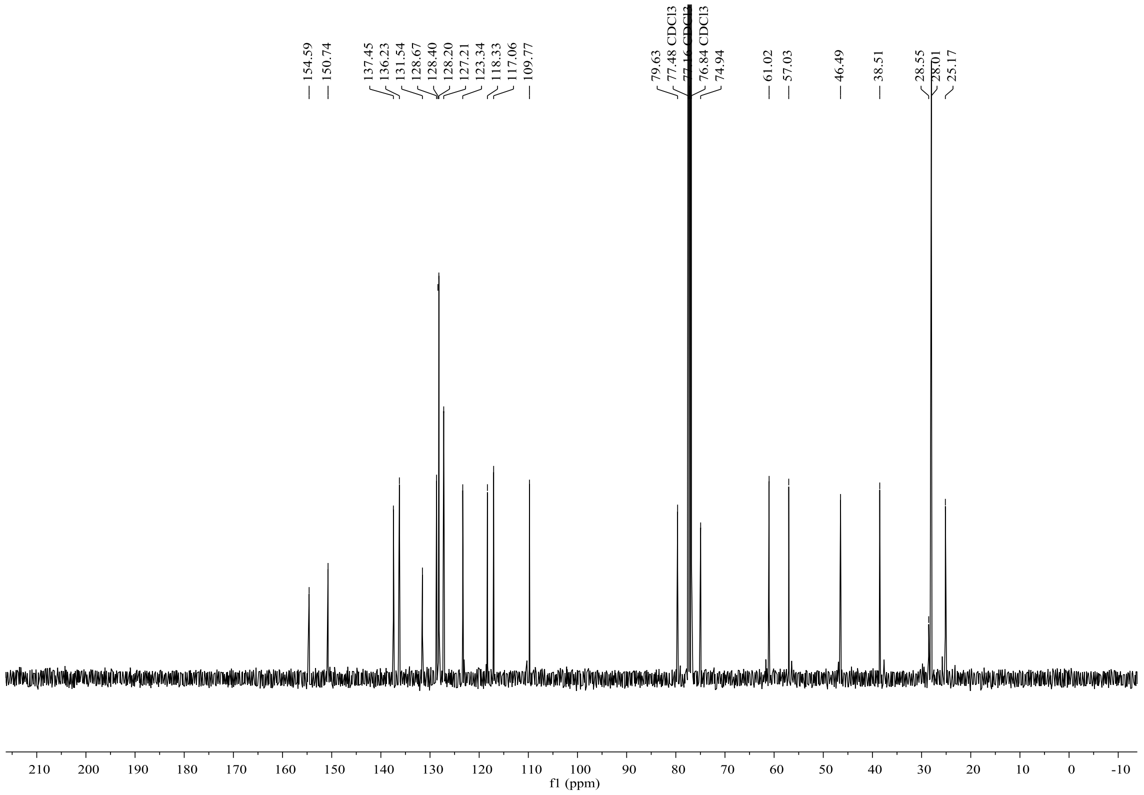

tert-Butyl 4-oxo-2-vinyl-3,4-dihydro-2_H_-spiro[naphthalene-1,2'-pyrrolidine]-1'-carboxylate (9)

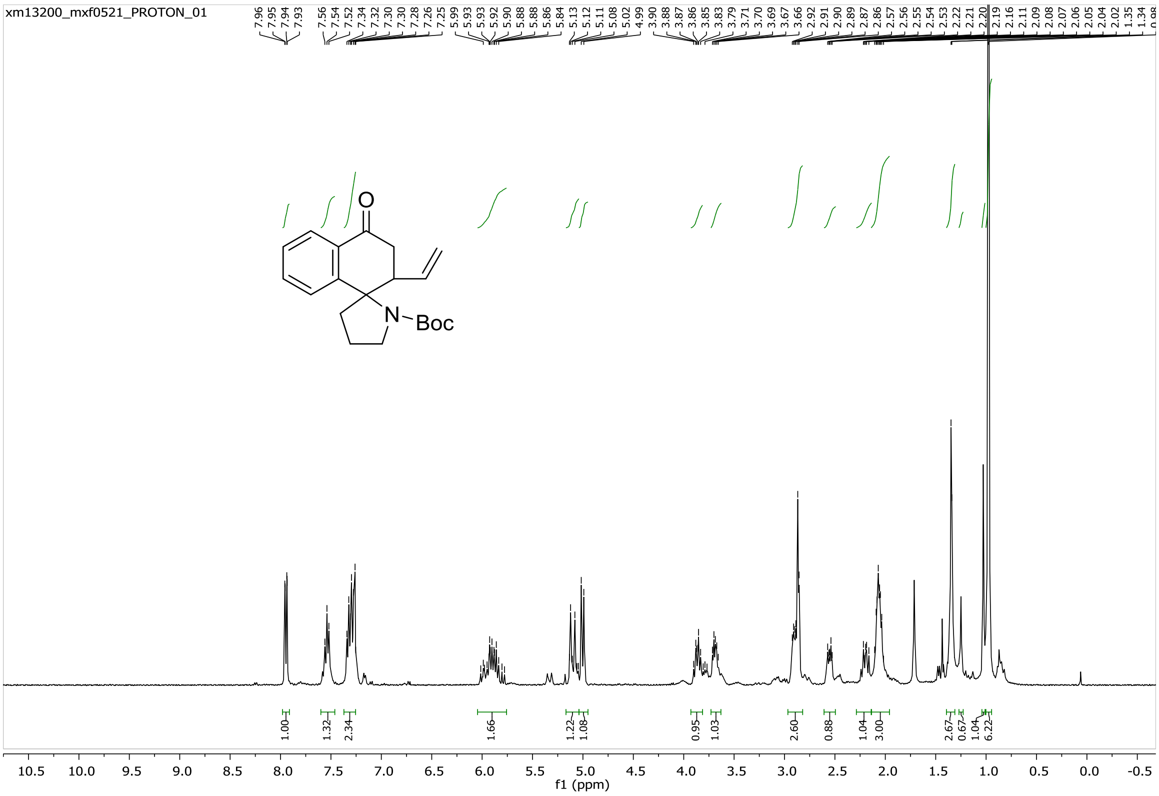

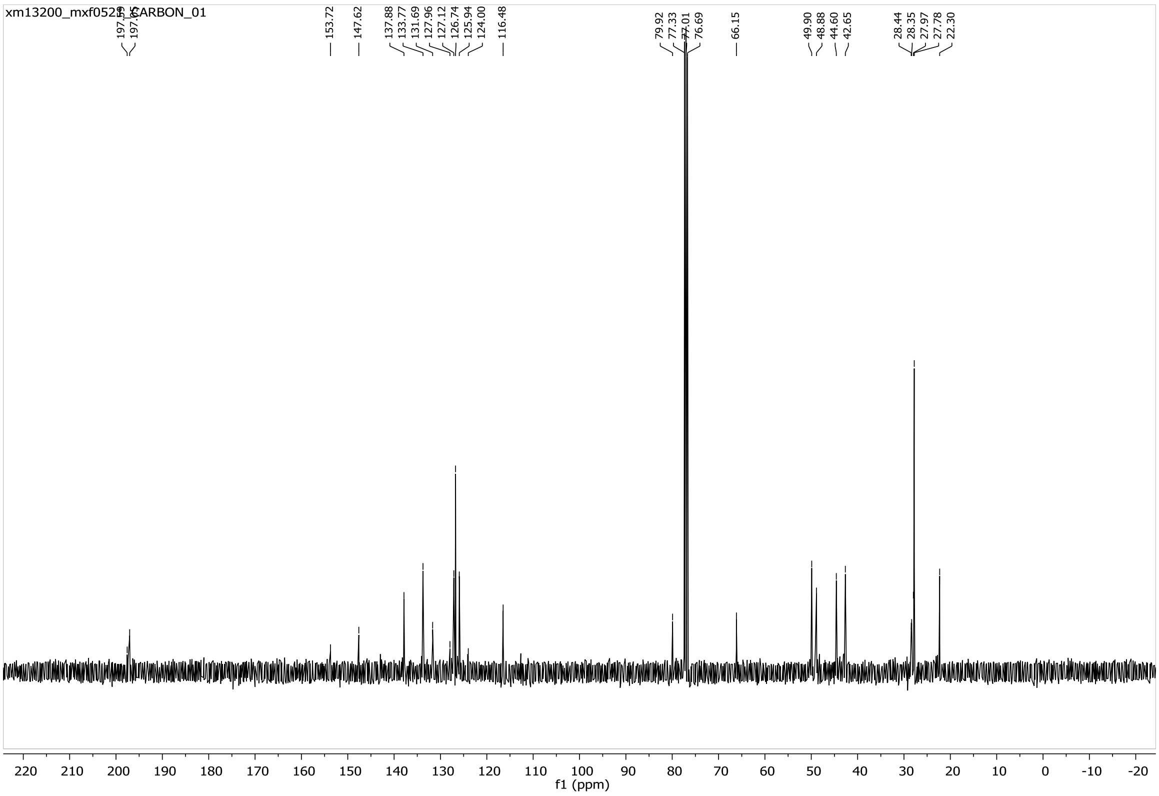

tert-Butyl (3-(1*H*-indol-3-yl)propyl)carbamate (10)

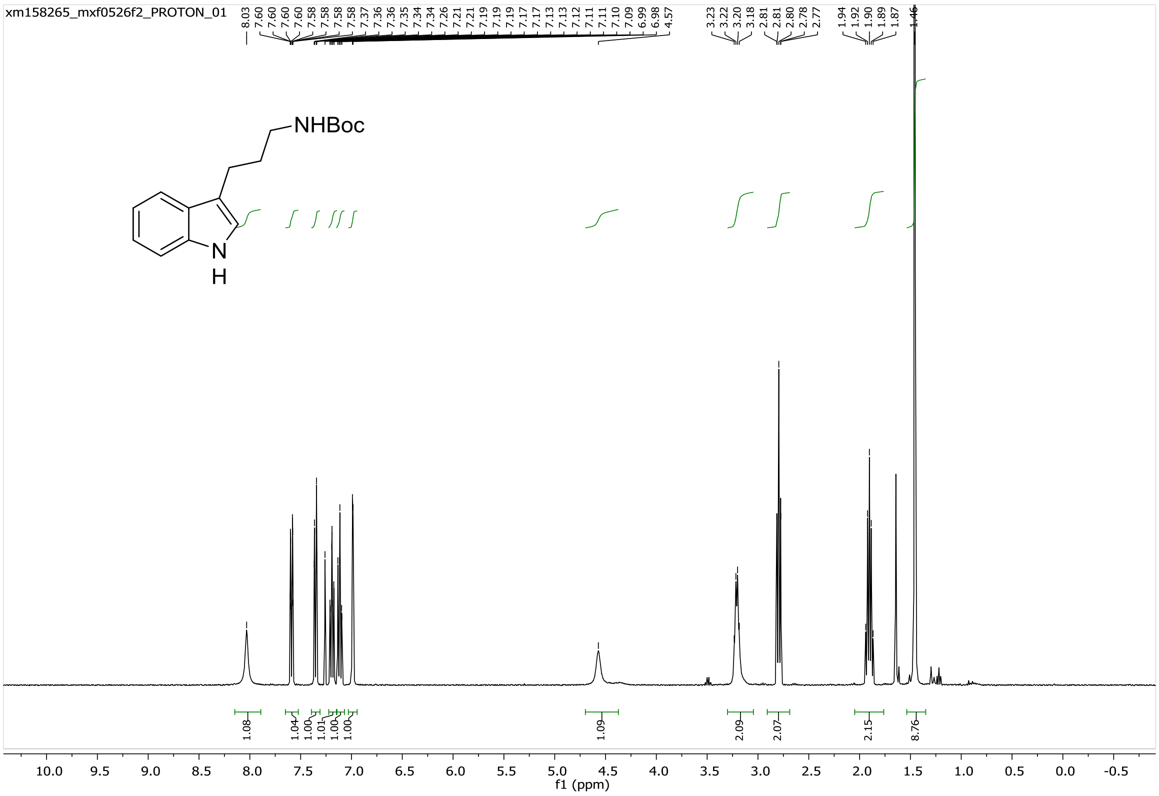

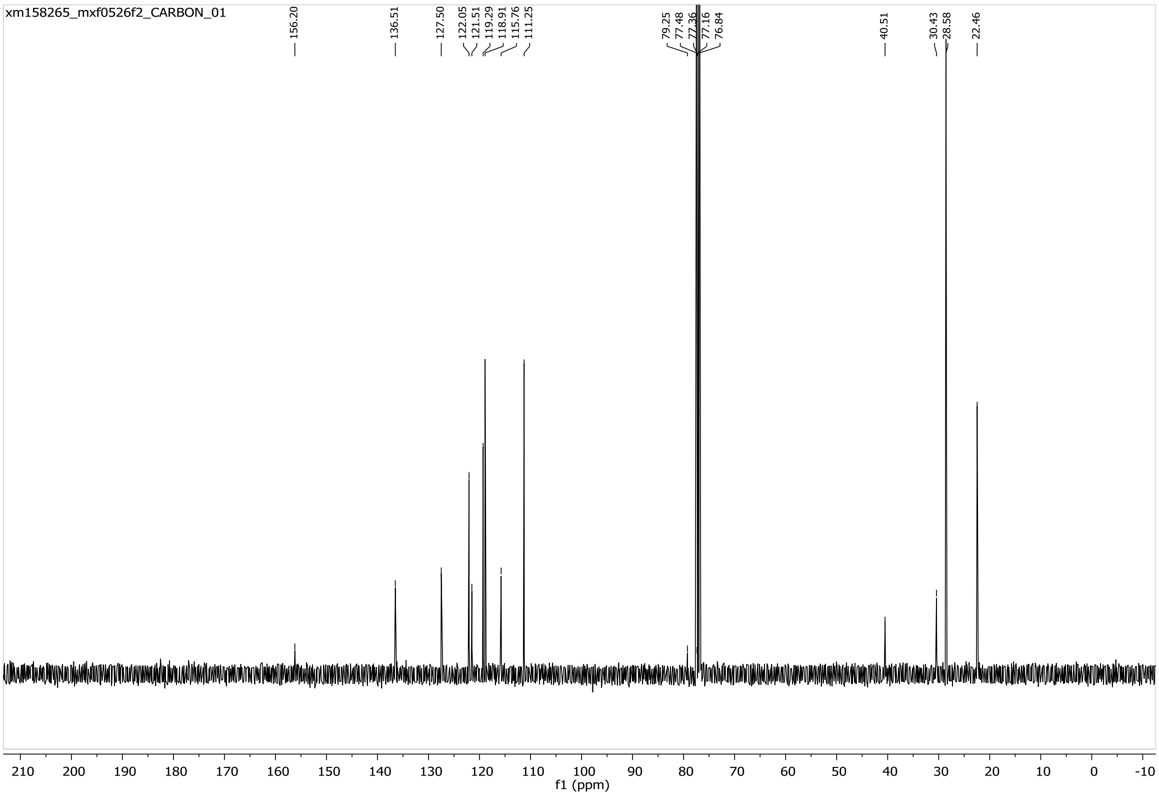

Benzyl (*E*)-hex-4-en-1-yl((pentafluorobenzoyl)oxy)carbamate (11a)

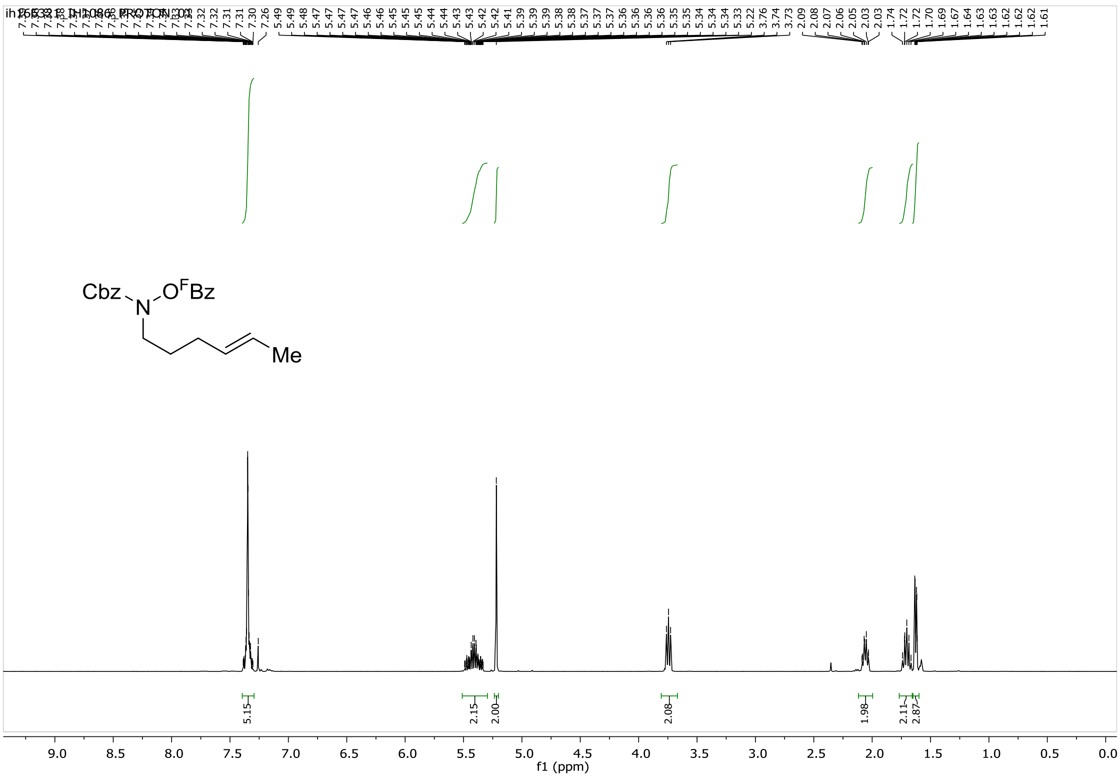

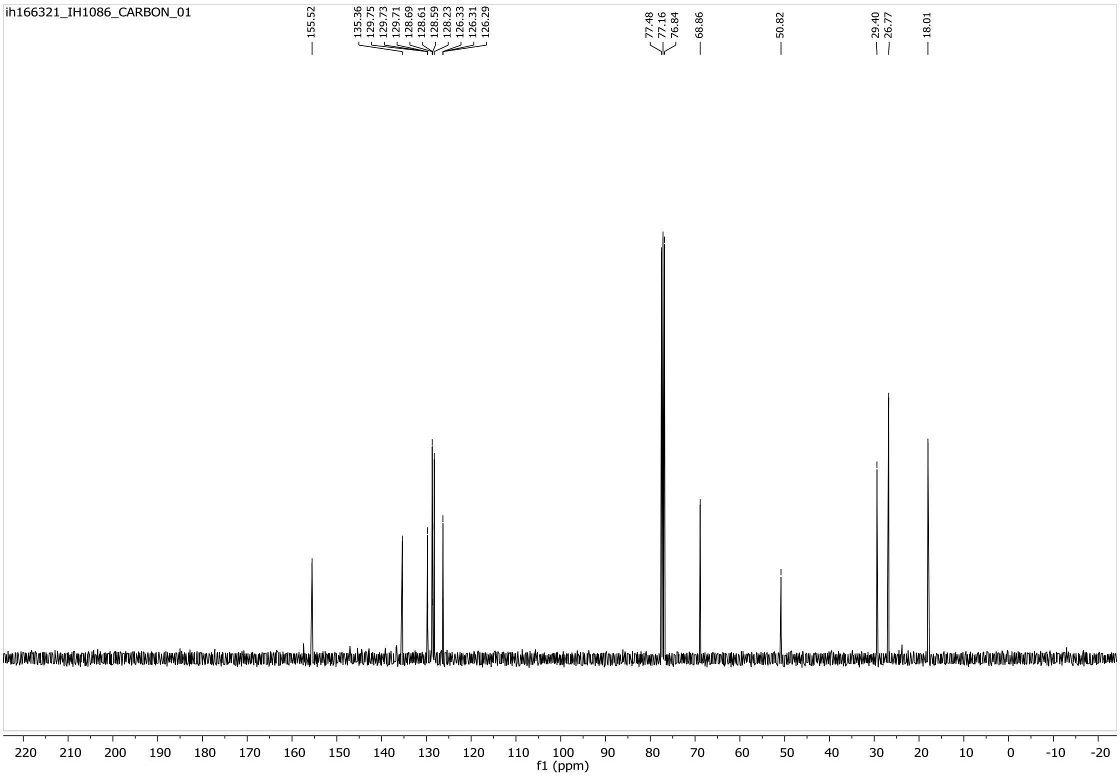

tert-Butyl (*E*)-hex-4-en-1-yl(tosyloxy)carbamate (11b)

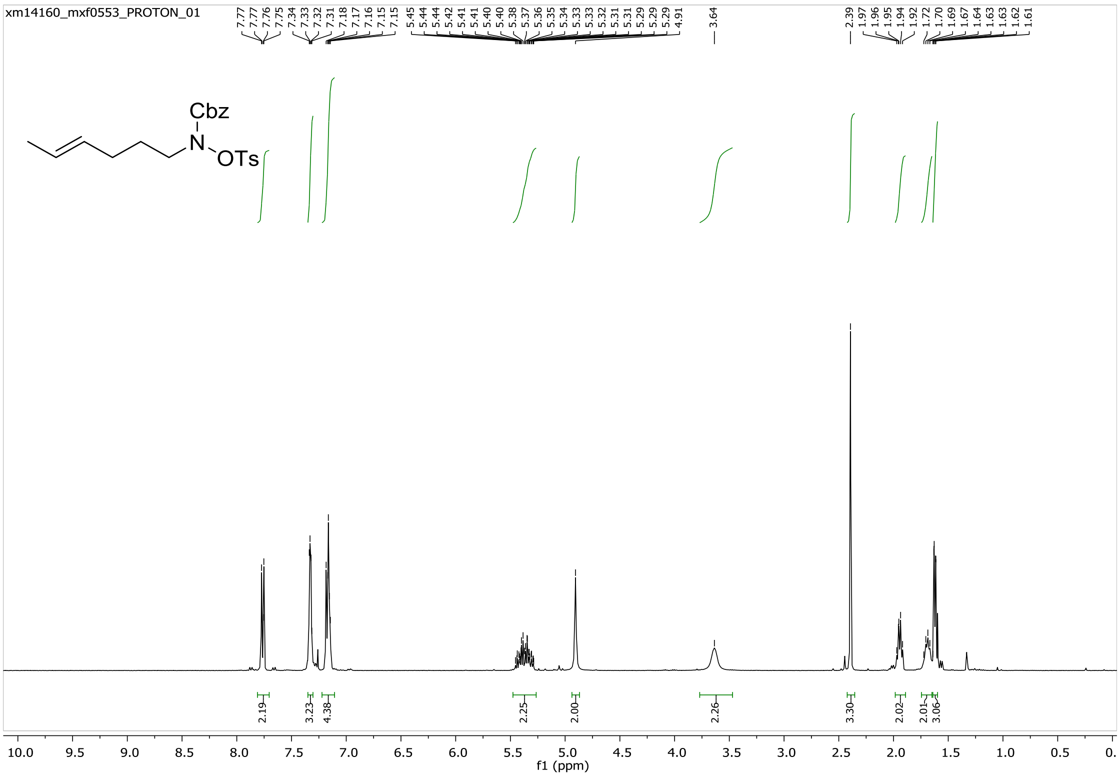

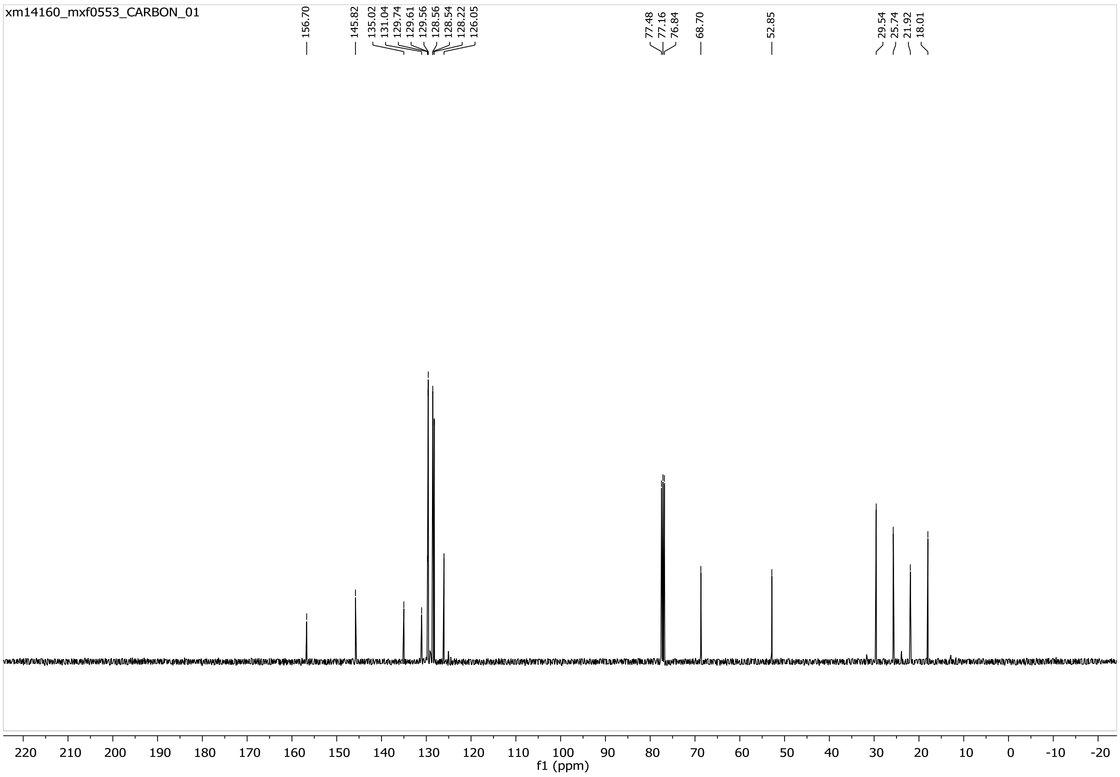

Methyl (3-(4-((*tert*-butyldimethylsilyl)oxy)phenyl)propyl)(tosyloxy)carbamate

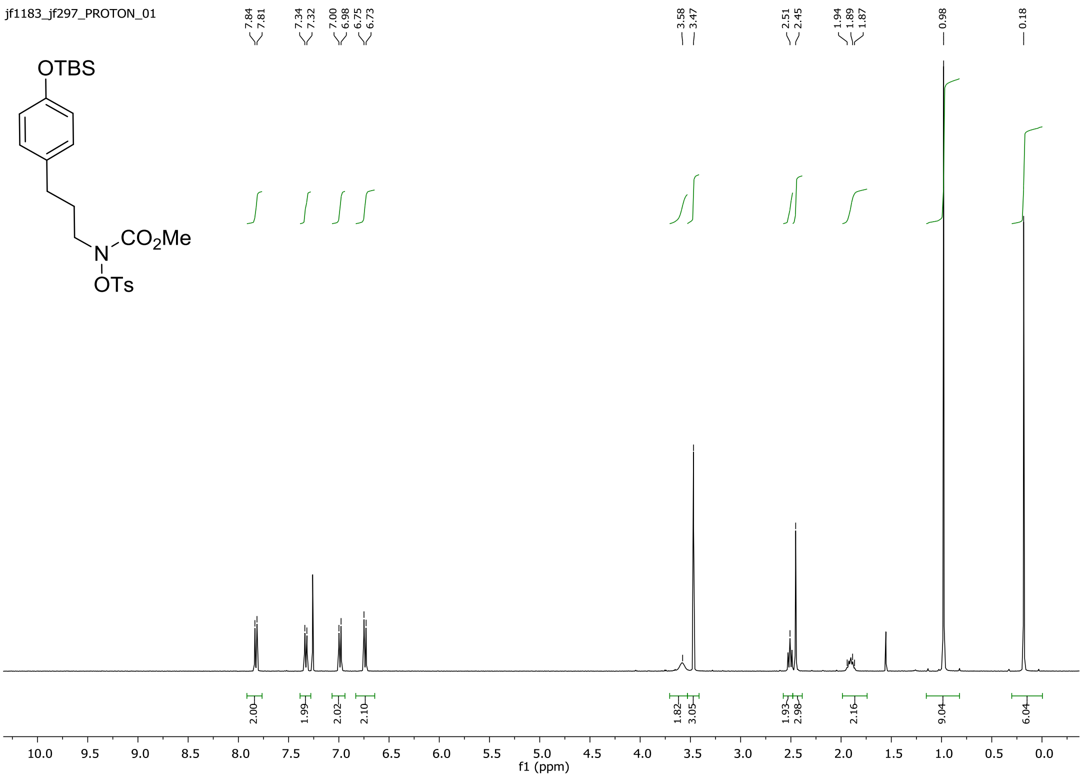

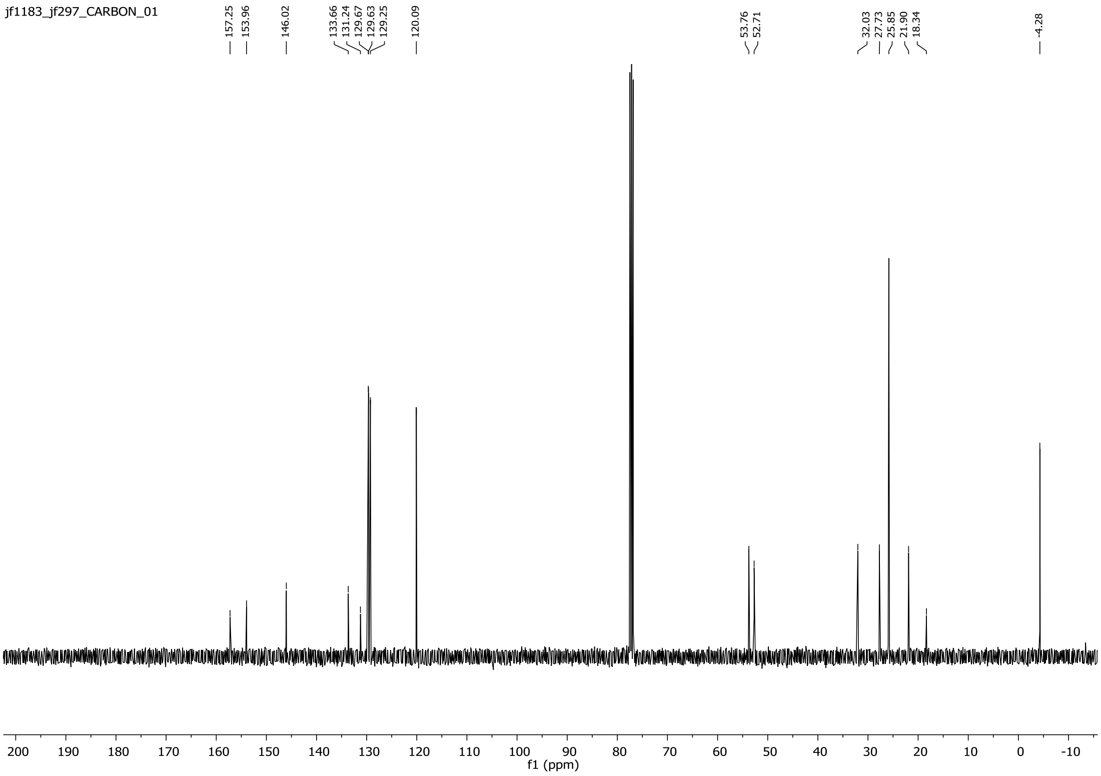

Methyl (3-(4-hydroxyphenyl)propyl)(tosyloxy)carbamate (5a-Me)

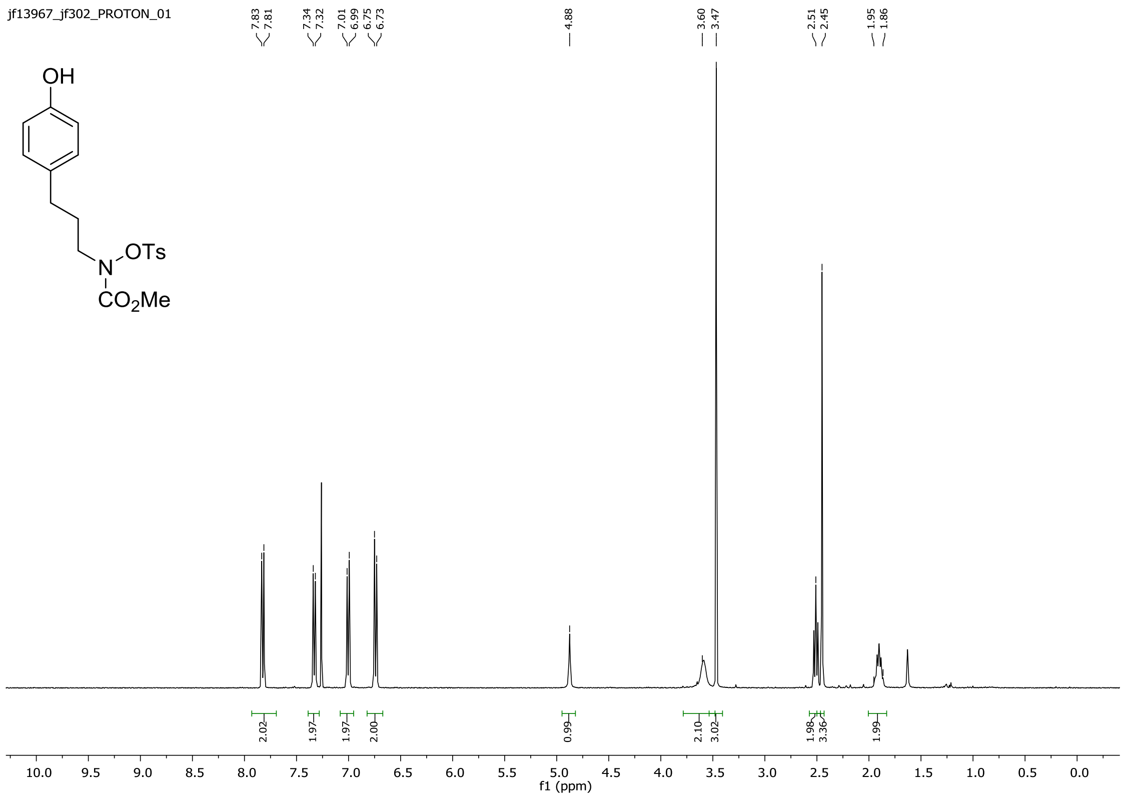

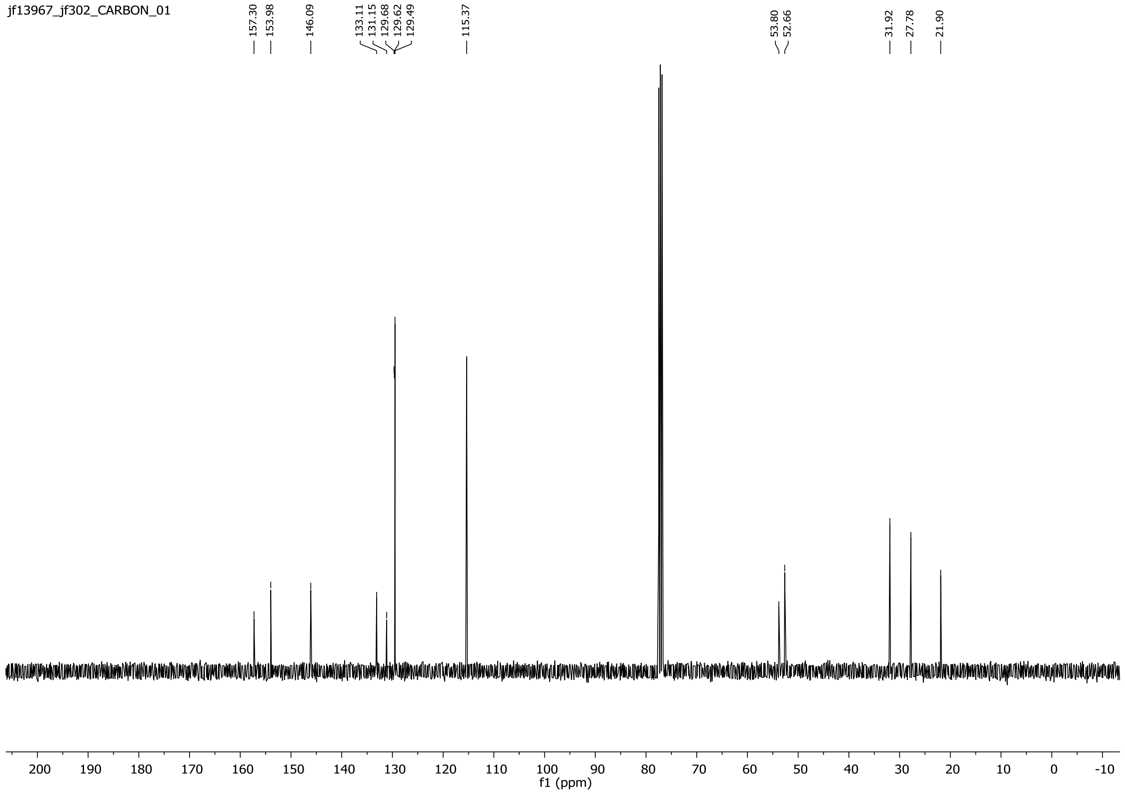

Methyl 8-oxo-1-azaspiro[4.5]deca-6,9-diene-1-carboxylate (6a-Me)

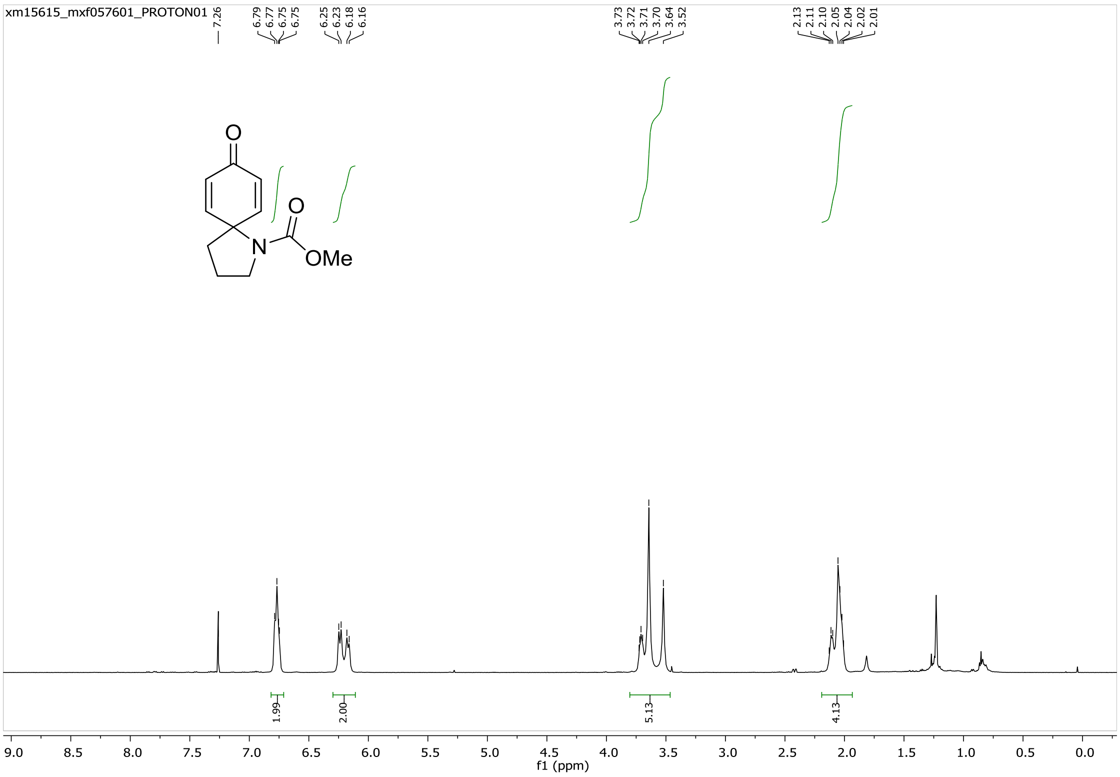

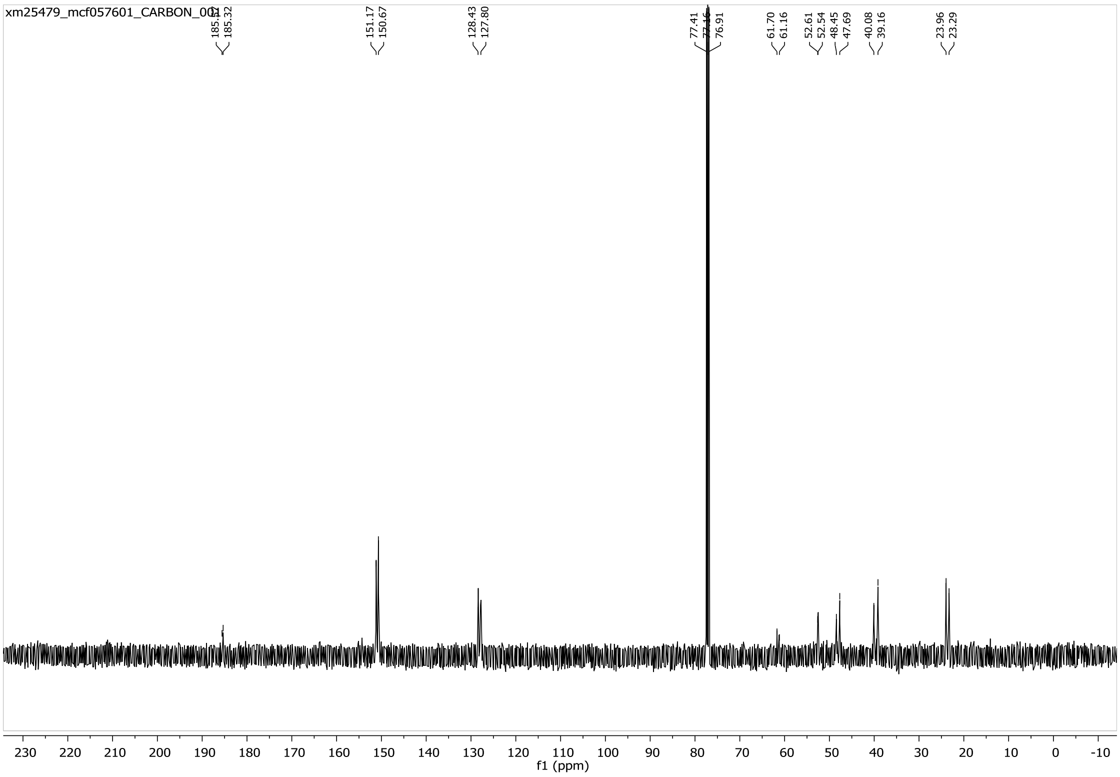

Supplement: Supplementary file 1 — Supplementary [file ANIE-56-14531-s001.pdf]
